# Supplementary material for: MEMPHIS: a smartphone app using psychological approaches for women with chronic pelvic pain presenting to gynaecology clinics: a randomised feasibility trial
Source: BMJ Open. 2020 Mar 12;10(3):e030164. doi: 10.1136/bmjopen-2019-030164 (PMC7069270; doi:10.1136/bmjopen-2019-030164)
Supplement: Supplementary data [file bmjopen-2019-030164supp001.pdf]

A smartphone app using psychological approaches for women with chronic pelvic pain (MEMPHIS): a randomised feasibility trial

**Short Title/Acronym**	MEMPHIS
**Sponsor**	Barts Health NHS Trust<br><br>Contact person of the above sponsor organisations is:<br><br>Dr Sally Burtles<br>Director of Research Services & Business Development<br>Joint Research Management Office<br>5 Walden Street<br>London<br>E1 2EF<br>Phone: 020 7882 7260<br>Email: *sponsorsrep@bartshealth.nhs.uk*
**REC Reference**	15/LO/1967
**Chief Investigator**	**Miss Elizabeth Ball**<br>Women and Children Department<br>Royal London Hospital<br>Barts Health NHS Trust<br>elizabeth.ball@bartshealth.nhs.uk<br>eball69@gmail.com

Forbes G, *et al. BMJ Open* 2020; 10:e030164. doi: 10.1136/bmjopen-2019-030164

Pragmatic Clinical Trials Unit

Blizard Institute, Centre for Primary Care and Public Health

Bart's and The London School Medicine and Dentistry

Queen Mary University of London

Yvonne Carter Building, 58 Turner St

Whitechapel, E1 2AB

PCTU Senior Trial statistician:

Mr Brennan Kahan

+44 207 882 2483

b.kahan@qmul.ac.uk

PCTU Junior Trial Statistician

Mr Neil Wright

+44 207 882 6564

n.wright@qmul.ac.uk

PCTU Data Manager:

Mr Mike Waring

+44 207 882 6914

m.r.waring@qmul.ac.uk

PCTU QA Manager:

Ms Anitha Manivannan

+44 207 882 6312

a.manivannan@qmul.ac.uk

Protocol development

Miss Elizabeth Ball

Chief Investigator

Forbes G, *et al. BMJ Open* 2020; 10:e030164. doi: 10.1136/bmjopen-2019-030164

elizabeth.ball@bartshealth.nhs.uk
eball69@gmail.com

Dr Julie Dodds
Senior Research Manager
j.dodds@qmul.ac.uk

Mr Brennan Kahan
PCTU Senior Trial Statistician
b.kahan@qmul.ac.uk

Mr Neil Wright
PCTU Junior Trial Statistician
n.wright@qmul.ac.uk

Dr Carol Rivas
Senior Research Fellow
C.A.Rivas@soton.ac.uk

Miss Ewelina Rogozinska
Co-investigator
e.a.rogozinska@qmul.ac.uk

PCTU QA Manager:
Ms Anitha Manivannan
+44 207 882 6312
a.manivannan@qmul.ac.uk

Forbes G, *et al. BMJ Open* 2020; 10:e030164. doi: 10.1136/bmjopen-2019-030164

Barts and The London
School of Medicine and Dentistry

CONTENTS PAGE

1.GLOSSARY OF TERMS AND ABBREVIATIONS	7
2.SIGNATURE PAGES	8
3.SUMMARY/SYNOPSIS	9
4.INTRODUCTION	11
4.1. Background	11
4.2. Effect of mindfulness meditation in chronic pain patients	12
4.3. On-going studies	12
4.4. Implications for the further development of clinical or public health practice	13
4.5. Potential impact on local policy making and improvement in service delivery	14
5.TRIAL OBJECTIVES	14
5.1. Aims and Objectives	14
5.2. Feasibility outcomes	15
5.3. App satisfaction questionnaires	16
5.4. Clinical Outcomes	17
6.METHODOLOGY	18
6.1. Inclusion Criteria	18
6.2. Exclusion Criteria	18
6.3. Study Design	19
6.4. Study Scheme Diagram	20
7. STUDY PROCEDURES	20
7.1.Informed Consent Procedures	20
7.2. Screening and enrolment	21
7.3. Randomisation Procedures	22
7.4 Blinding	22
7.5. Planned interventions	23
7.6. Concomitant Medications	24
7.7. Reasons for non progression to full trial	24

Forbes G, *et al. BMJ Open* 2020; 10:e030164. doi: 10.1136/bmjopen-2019-030164

7.8. Key risks to delivering this research and contingencies 24
7.9. Procedure for Collecting Data 25
7.10. Including Case Report Forms (CRFs) and storage 25
7.11. Follow-up Procedures 25
7.12. Subject withdrawal (including data collection / retention for withdrawn participants) 25
7.13. Continued app use after trial period and app use by treatment as usual group 26
7.14. Schedule of Assessment 27
7.15. Criteria for Early Termination of the study 28
7.16. End of Study Definition 28
8. STATISTICAL CONSIDERATIONS 28
8.1. Sample Size 28
8.2. Statistical Analysis 28
9. ETHICS 30
10. SAFETY CONSIDERATIONS 31
11. DATA HANDLING AND RECORD KEEPING 31
11.1 Confidentiality 31
11.2. Required Study Documents 32
11.3. Record Retention and Archiving 33
12. PRODUCTS, DEVICES, TECHNIQUES AND TOOLS 33
12.1. Devices 33
12.2. Techniques and intervention 34
13. SAFETY REPORTING 35
13.1. Adverse Events (AE) 35
13.2. Serious Adverse Event (SAE) 35
13.3. Urgent Safety Measures 36
13.4. Annual Safety Reporting 36
13.5. Overview of the Safety Reporting Responsibilities 37
14. MONITORING & AUDITING 37
14.1. Auditing 37
14.2. Summary Monitoring Plan 37

Forbes G, *et al. BMJ Open* 2020; 10:e030164. doi: 10.1136/bmjopen-2019-030164

14.3. Compliance 38
14.4. Non-Compliance 38
15. TRIAL COMMITTEES 39
15.1. Trial Steering Committee (TSC) 38
15.2. Trial Management Group (TMG) 39
15.3. Data Monitoring Committee (DMC) 39
16. FINANCE AND FUNDING 39
17. INDEMNITY 39
18. DISSEMINATION OF RESEARCH FINDINGS 40
REFERENCES 42
APPENDICES 46

Forbes G, *et al. BMJ Open* 2020; 10:e030164. doi: 10.1136/bmjopen-2019-030164

1. GLOSSARY of Terms and Abbreviations

AE	Adverse Event
CI	Chief Investigator
CPP	Chronic Pelvic Pain
CRF	Case Report Form
DMC	Data Monitoring Committee
GCP	Good Clinical Practice
HCP	Health Care Professional
ICF	Informed Consent Form
JRMO	Joint Research Management Office
KTN	Katherine Twining Network
MHRA	Medicines and Healthcare products Regulatory Agency
NHS REC	National Health Service Research Ethics Committee
NHS R&D	National Health Service Research & Development
NPT	Normalization Process Theory
Participant	An individual who takes part in a clinical trial
PCTU	Pragmatic Clinical Trial Unit
PI	Principal Investigator
PIS	Participant Information Sheet
PSM	Patient Self-Management
QOL	Quality Of Life
QC	Quality Control
RCT	Randomised Controlled Trial
REC	Research Ethics Committee
RfPB	Research for Patients Benefit
SAE	Serious Adverse Event
SOP	Standard Operating Procedure
SUS	System Usability Scale
TAU	Treatment As Usual
TMG	Trial Management Group
TSC	Trial Steering Committee

Forbes G, *et al. BMJ Open* 2020; 10:e030164. doi: 10.1136/bmjopen-2019-030164

Barts and The London
School of Medicine and Dentistry

2. SIGNATURE PAGES

Chief Investigator/Principal Investigator Agreement

The clinical study as detailed within this research protocol **(Version V8.0, dated 22 12 2016)**, or any subsequent amendments will be conducted in accordance with the Research Governance Framework for Health & Social Care (2005), the World Medical Association Declaration of Helsinki (1996) and the current applicable regulatory requirements and any subsequent amendments of the appropriate regulations.

Chief Investigator Name: Miss Elizabeth Ball
Chief Investigator Site: Barts and the London School of Medicine and Dentistry, Queen Mary University of London

Signature and Date: Elizabeth Ball **22.12.2016**

Statistician Agreement

The clinical study as detailed within this research protocol **(Version V8.0, 22 12 2016)**, or any subsequent amendments will be conducted in accordance with the Research Governance Framework for Health & Social Care (2005), the World Medical Association Declaration of Helsinki (1996) and the current applicable regulatory requirements and any subsequent amendments of the appropriate regulations.

Statistician Name: Mr Brennan Kahan
Statistician Site: Pragmatic Clinical Trials Unit, Queen Mary University of London

Signature and Date: **22.12.2016**

Forbes G, *et al. BMJ Open* 2020; 10:e030164. doi: 10.1136/bmjopen-2019-030164

Barts and The London
School of Medicine and Dentistry

3. SUMMARY/SYNOPSIS

**Short Title**	MEMPHIS
**Methodology**	A randomised feasibility trial
**Research Sites**	This trial will be conducted at the Royal London and Whipps Cross Hospitals
**Objectives/Aims**	The overall aim is to assess the feasibility of implementing a trial of a mindfulness meditation intervention delivered by a mobile phone app for patients with chronic pelvic pain (CPP). The primary objectives are:<br>1) To provide feasibility data for a large multicentre RCT aimed at rigorously testing mindfulness meditation in CPP<br>2) To determine whether this app can be seamlessly integrated into clinical practice, especially CPP pathways
**Number of Participants/Patients**	90 women with CPP will be recruited and each randomised into one of the three trial groups (meditation app, progressive muscle relaxation or no app).
**Main Inclusion Criteria**	To be eligible for the MEMPHIS study, the women must:<br>● Be age 18 or over<br>● Have either organic or non-organic chronic pelvic pain lasting for 6 months or more<br>● Have access to a personal computer or smartphone.<br>● Understand simple spoken English
**Statistical Methodology and Analysis (if applicable)**	Feasibility outcomes will be summarised using descriptive statistics. Clinical outcomes will be analysed using linear mixed-effects models, and results will be presented as a difference in means and a 95% confidence interval.

Forbes G, *et al. BMJ Open* 2020; 10:e030164. doi: 10.1136/bmjopen-2019-030164

	Usability and integration into clinical practice will be explored in focus groups or via telephone interviews with participants.<br>Some participants will be asked to elaborate about app satisfaction and also on clinical outcomes. Results will be analysed using content analysis including both thematic and text word analysis.
**Proposed Start Date**	November 2015
**Proposed End Date**	August 2017
**Study Duration**	22 months

Forbes G, *et al. BMJ Open* 2020; 10:e030164. doi: 10.1136/bmjopen-2019-030164

Barts and The London
School of Medicine and Dentistry

4. INTRODUCTION

4.1. Background

Chronic pelvic pain (CPP) is defined as intermittent or constant pain in the lower abdomen or pelvis of a woman for at least 6 months, not exclusively associated with menstruation, intercourse and not associated with pregnancy [1].
It affects up to 24% women worldwide [2], accounts for 20% of UK gynaecological clinic referrals [3], and has a considerable impact on patients' quality of life and their income. CPP costs the NHS € 3.3bn per year [4]. Despite costly interventions, CPP is often resistant to surgical and medical treatment. Multifactorial psychological and somatic causes require a multidimensional approach, which is not routinely offered in gynaecology clinics [5]. Evidence from randomised controlled trials (RCTs) suggests that psychological interventions may be superior to primary surgery [6]. Although psychological treatment is provided across the NHS, mostly in the context of the primary care programme *Improving Access to Psychological Therapies* there are problems with capacity, waiting times, and the overall number of patients being able to access services. Alternatively, patient self-management (PSM) is now recognised as a tool empowering patients to cope better with their condition [7]. Mindfulness meditation is a potentially valuable PSM tool in CPP. We conducted a systematic search of literature (07/2013, updated 12/2013) and found no RCTs of mindfulness meditation in CPP. However, we identified two small, non-randomised pilot trials investigating the effect of mindfulness meditation on pain (one in women with CPP and one in women with endometriosis) both of which showed promising results [8,9].

Because we identified no RCTs on mindfulness meditation in CPP in our systematic review, we included other chronic pain conditions which may have a similar patho-mechanism to pelvic pain, such as back pain, headache, fibromyalgia and diabetic neuropathy. We assume that any benefits of mindfulness meditation in these conditions may also be seen in CPP.
We found previous systematic reviews in these conditions had a number of limitations, such as not reporting effect sizes [10-12].

Forbes G, *et al. BMJ Open* 2020; 10:e030164. doi: 10.1136/bmjopen-2019-030164

Our systematic review conducted in lines with current standards [13] identified 472 relevant citations. Nine RCTs met fully the review's inclusion criteria [14,15,16-22]. Most studies were of moderate quality; but sample sizes were generally small (from 65 women for quality of life in mental health domain to 259 women for depression).

4.2. Effect of Mindfulness based meditation in chronic pain patients

Our results showed Mindfulness based meditation reduced depression levels in chronic pain patients (standardised mean difference (SMD) -0.28; 95% CI -0.53, -0.03; p = 0.03)). Patients who received Mindfulness meditation tended to cope better with anxiety (SMD -0.16, 95% CI -0.47, 0.15) and affective pain (the emotional reaction to pain) (SMD -0.13, 95% CI -0.42, 0.16). Women in the intervention arm had also higher Quality of life (QOL) scores (especially the mental health component SMD 0.65, 95% CI -0.27, 1.58) and higher pain acceptance (SMD 0.53, 95% CI -0.13, 1.19); although these results were not statistically significant. Only one of the included studies reported the important measure of pain acceptance.

Currently Mindfulness-based therapy is creating lively research interest. Two recent systematic reviews report positive effects on somatisation disorders [23] and psychological stress [24].

4.3. On-going studies

Although there are currently no on-going studies of Mindfulness in patients with CPP that we are aware of, there are other NIHR funded studies with overlapping themes**.**

Self help in CPP

The RFPB-funded study SUPPORT, which is currently in follow- up (MREC 10/H1005/24), is investigating an evidence-based self-care guidance in general practice for women with CPP. GPs received training to use the guidance in their consultations. Women were randomised to either receive the facilitated self-care guide or usual care. Results from SUPPORT will provide valuable information on how best to integrate a new patient self-help intervention into an existing patient pathway.

Forbes G, *et al. BMJ Open* 2020; 10:e030164. doi: 10.1136/bmjopen-2019-030164

Interactive mobile phone application to modify patient behaviour

The recently closed RFPB-funded feasibility study STARFISH (MREC 12/WS/0309) investigated the acceptability of a smartphone app that encourages stroke patients to become more physically active. The number of steps taken per day by the individual is monitored. Patients work in small groups and different goals can be set for different individuals in the group, along with goals for the whole group. It will be interesting to compare the reported obstacles and facilitators to using the app with MEMPHIS.

Web-based delivery of an intervention

Of particular interest, due to the similarities in study design to MEMPHIS, is a recently closed pilot study, MIMS (UKCRN ID 13105) that investigated adjustment to multiple sclerosis.

In MIMS, meditation teaching was delivered by videoconference. Web-based delivery has also been explored and shown to be feasible for reducing stress, anxiety and depression [25]; both options are lacking the flexibility of a smartphone app, which we are proposing.

4.4. Implications for the further development of clinical or public health practice

Our co-investigator Judy Birch is closely involved with the committee that produces national guidelines for CPP patient care pathways, which she helps to develop [26]. If the app were proven to be effective in a phase III trial, it would be possible for it to be incorporated in this pathway.

One outcome measure of MEMPHIS is to determine whether this app can be integrated into clinical practice, especially CPP pathways. If this is the case there would be benefit from studying how to extend the app to other pain conditions, such as headache, back pain and irritable bowel syndrome, in which face-to face delivered mindfulness meditation has had positive effects [23].

Forbes G, *et al. BMJ Open* 2020; 10:e030164. doi: 10.1136/bmjopen-2019-030164

If this app is shown to be effective in a phase III trial, we will collaborate closely with Headspace, our local Health and Education Cluster and Queen Mary to implement this app both locally and nationally.

4.5. Potential impact on local policy making and improvement in service delivery

Chronic pelvic pain patients would benefit from multiple treatment approaches [6] but currently most gynaecological departments only offer medical and/or surgical treatment [5]. Although psychological treatment is provided across the NHS, mostly in the context of the primary care programme *Improving Access to Psychological Therapies* there are problems with capacity, waiting times, and the overall number of patients being able to access services. If the app is proven to be useful in a phase III RCT this gap could be filled, without having to employ more psychologists, because the interventions would be largely app delivered. Locally this would help our concerns about access to psychological treatment for CPP. Given the ubiquity of the app, greater compliance with treatment and less wastage from patients not attending appointments is expected. The use of the app in local primary, secondary and tertiary care settings would be introduced in collaboration with GP commissioning groups through local guidelines and protocols.

5. TRIAL OBJECTIVES

5.1. Aims and Objectives

The overall aim is to assess the feasibility of implementing a trial of a mindfulness meditation intervention delivered by a mobile phone app for patients with chronic pelvic pain (CPP). The primary objectives are:

- To provide feasibility data for a large multicentre RCT aimed at rigorously testing Mindfulness meditation in patients with CPP. The full-scale trial will assess the effectiveness of the mindfulness meditation app in patients with chronic pelvic pain in a national multicentre RCT

Forbes G, *et al. BMJ Open* 2020; 10:e030164. doi: 10.1136/bmjopen-2019-030164

- To determine whether this app can be seamlessly integrated into clinical practice, especially CPP pathways. In cooperation with the Pelvic Pain Support Network, which is instrumental in the initiative on implementing nationwide pathways for patients with CPP, we will review the data on feasibility, especially the patient feedback and process analysis to answer this question to find out if the app, if it has been shown to be effective could be incorporated straight away into a national clinical pathway for CPP patients

5.2. Feasibility outcomes

5.2.1. Feasibility outcomes collected from participants

- Duration of recruitment (measured from the day recruitment opens until the day the 90th patient is randomised).
- Estimates to be used for the sample size calculation of the phase III RCT (the estimated SD for pain acceptance, and the dropout rate).
- Patient adherence to app use will be measured by the following outcomes:
    - Number of days (within the first 60 days from randomisation) a patient has used the app (with app use defined as having completed at least 90% of a session).
    - Whether the patient has used the app on 22 or more days within the first 60 days from randomisation.
    - Number of weeks (within the first eight weeks from randomisation) a patient has used the app on three or more days.
    - Whether the patient has used the app on three or more days in 6 or more weeks (within the first eight weeks from randomisation).
    - Whether the patient has used the app on 22 or more days within the first 60 days from randomisation, AND used the app on three or more days in 6 or more weeks within the first eight weeks from randomisation.
- Reasons for patient non-adherence to app use.

Forbes G, *et al. BMJ Open* 2020; 10:e030164. doi: 10.1136/bmjopen-2019-030164

5.2.2. Feasibility outcomes collected from participant focus groups

- Usability and integration into clinical practice will be explored in two focus groups post-intervention with approximately 15 app participants, who have completed the 60 day follow up. Alternatively, participants unable to attend focus groups will be given the chance to answer a questionnaire over the phone with a research nurse.
- Discussions will be recorded and literal themes on integration and usability will be evaluated for in depth information. This information will be considered as well as adherence to the app as an indirect measure of acceptability. In cooperation with the Pelvic Pain Support Network, which is instrumental in the initiative on implementing nationwide pathways for patients with CPP, we will review the data on feasibility, especially the patient feedback and process analysis to answer this question to find out if the app, If it has been shown to be effective could be incorporated straight away into a national clinical pathway for CPP patients.
- We will determine primary and secondary outcomes of interest from the perspective of patients, for a full-scale trial. This will involve asking participants who were randomised to the app groups to discuss and prioritise outcomes.
- Obstacles to recruitment will also be explored.

5.2.3. Feasibility outcomes collected from health care practitioner focus groups

- A purpose made topic guide will be used to structure a focus group with service providers and based on the NPT toolkit [27] and the Diffusion of Innovations Theory [28] as a prompt for the facilitator.

  The service providers will be asked to consider their role and their organisation and to suggest and discuss any issues to integration, and also – unlike conventional qualitative research focus groups – to suggest potential solutions. Discussions will be based around Diffusion of Innovations Theory, that is, we will consider:

  - Relative advantage vs. existing practices

- Compatibility with existing practices
- Simplicity and ease of integration
- Trialability and reinvention of the process
- Feedback (e.g. can clinicians see that patients benefit?)
- Peer to peer networking

We will use our findings to develop our integration approach to be further explored in the subsequent full trial.

- Obstacles to recruitment will also be explored.

5.3. Clinical outcomes

- Quality of life score, Physical Functioning subscale (as measured by the RAND Short Form (36) Health Survey (SF-36))
- Quality of life score, Social Functioning subscale (as measured by the RAND SF-36)
- Quality of life score, Pain subscale (as measured by the RAND SF-36)
- Quality of life score, General Health subscale (as measured by the RAND SF-36)
- Depression score (as measured by the Hospital Anxiety and Depression Scale (HADS))
- Anxiety score (as measured by HADS)
- Mindfulness score (as measure by the Cognitive and Mindfulness - Revised (CAMS – R) scale)
- Pain related disability score (as measured by the Chronic Pain Grade (CPG) disability subscale)
- Self efficacy score (as measured by the Pain Self-Efficacy Questionnaire (PSEQ))
- Pain acceptance score (as measured Chronic Pain Acceptance Questionnaire (CPAQ-8))
- Sexual Health Outcomes score (as measured by Sexual Health Outcomes in

Women Questionnaire (SHOW-Q))
- Subjective outcome score (as measured by Measure Yourself Medical Outcome Profile (MYMOP))

All clinical outcomes will be analysed at 60 days, 3 months, and 6 months post-randomisation.

6. METHODOLOGY

6.1. Inclusion Criteria

To be eligible for the MEMPHIS study, the women must meet the following criteria:

- Aged 18 or over
- Women with organic and non-organic chronic pelvic pain lasting for six months or more
- Be capable of understanding the information provided, with use of an interpreter if required and being able to understand simple English as is used in the app
- Give written informed consent

6.2. Exclusion Criteria

Patients who mee**t the following criteria are** ineligible to participate:

- No access to a Personal computer or smartphone
- Current users of the Headspace app content available to the public

6.3. Study Design

MEMPHIS is a randomised, single centre feasibility trial. All eligible women referred to the chronic pelvic pain clinics at the Royal London and Whipps Cross Hospitals (both new and existing patients) will be approached to take part in the study. A study leaflet will be given to them, providing brief information of the study and informing them that they are invited to participate. After informed consent, we will randomise

eligible women in a 1:1:1 ratio (30 participants in each group) to one of the three treatment groups:

Group A - "Intervention": 60 days of the app delivering mindfulness meditation content (in addition to usual care). See section 7.4 for a detailed description.

Group B - "Active control": 60 days of the app delivering progressive muscle relaxation content (in addition to usual care). See section 7.4 for a detailed description.

Group C - Treatment as usual (TAU): Usual care

Setting: NHS Tertiary care hospital

6.4. Study Scheme Diagram

Figure 1

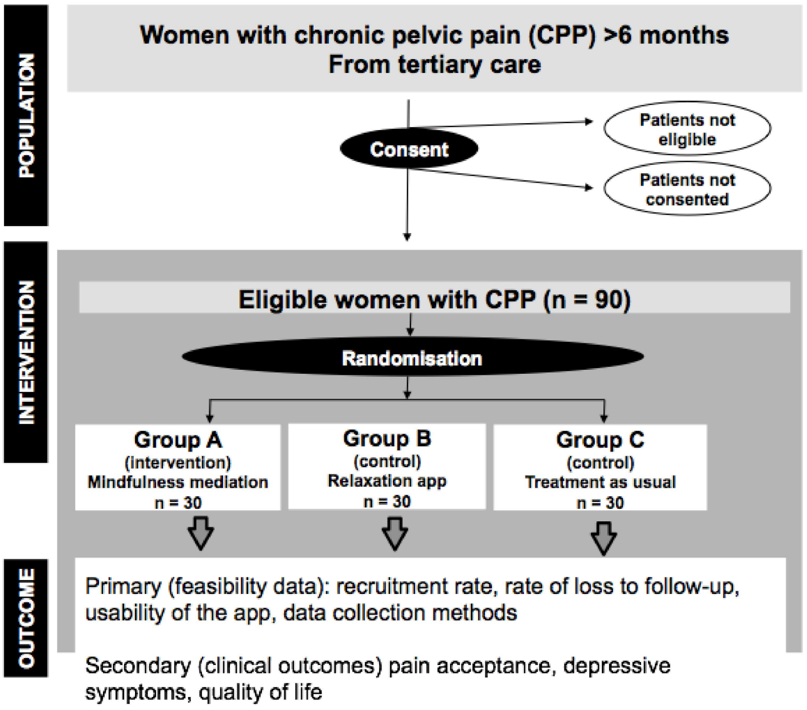

Forbes G, *et al. BMJ Open* 2020; 10:e030164. doi: 10.1136/bmjopen-2019-030164

7. STUDY PROCEDURES

7.1. Informed Consent Procedures

Women will be made aware of the study by a health care professional and through promotional material. Potentially eligible patients will receive the PIS along with their hospital appointment invitation to ensure they have adequate time (at least 24 hours) to consider the trial. The PIS will be accompanied with a letter from the PI informing the women that they may be approached about the study at their appointment. Eligible patients who are seen in clinics other than pelvic pain and endometriosis clinics will be given the PIS and contact details for the research practitioner so they can benefit from participating in MEMPHIS should they wish so.

The PIS will be reviewed and the patient will have the opportunity to ask any questions. All eligible participants willing to consent will be asked to sign the consent form. Women will be provided with the contact details of the researcher, and informed that they have the right to withdraw their consent at any stage. Some women may be asked for permission to be contacted by a research practitioner at a later stage for enrolment if there are time constraints.

Only those on the delegation log will be able to consent for the intervention. The consenting staff will have thorough knowledge of research governance issues surrounding consent and will be fully conversant with the protocol.

If they are eligible but do not wish to consent, this will be recorded. For the full scale trial we need to understand how many eligible patients need to be approached to reach the recruitment target. We also would like to identify if eligible women opt out of the study due to a rectifiable issue.

Women who give their approval will be randomised. The investigator (or another qualified person) will explain to the potential participant that they are free to refuse any involvement within the study or alternatively withdraw their consent at any point during the study and for any reason.

Forbes G, *et al. BMJ Open* 2020; 10:e030164. doi: 10.1136/bmjopen-2019-030164

If there is any further safety information, which may result in significant changes in the risk/benefit analysis, the PIS and Informed Consent Form (ICF) will be reviewed and updated accordingly. All participants who are actively enrolled on the study will be informed of the updated information and given a revised copy of the PIS/ICF in order to confirm their wish to continue on the study (if feasible), if it may change their willingness to participate. A copy of the consent form will be given to the participant; one will be kept in the hospital notes and the original will be placed in the Investigator Site File.

7.2. Screening and enrolment

New referrals and existing patients at the pelvic pain clinic are equally eligible. Through links with the Katherine Twining network and UCL partners we have established networks that can advertise recruitment. Based on these circumstances we are confident that we can achieve successful recruitment in the given timeframe.

Patients will be sent the Patient Information Sheet (PIS) in advance to ensure they have adequate time to consider the trial. The PIS will be accompanied with a letter from the PI informing patients that they may be approached about the study at their appointment.

At the appointment, the research practitioner will assess the women according to the inclusion/exclusion criteria detailed above and explain the nature of the intervention. The PIS will be reviewed and the patient will have the opportunity to ask any questions. All eligible participants willing to consent will be asked to sign the consent form. If a woman has not read or received the PIS before their appointment, the research team will go through the PIS with the individual in person. Women will be giving as much time as they want to consider the study before consent is taken. Women will be provided with the contact details of the researcher, and informed that they have the right to withdraw their consent at any stage.

Forbes G, *et al. BMJ Open* 2020; 10:e030164. doi: 10.1136/bmjopen-2019-030164

Barts and The London
School of Medicine and Dentistry

7.3. Randomisation Procedures

After informed consent, patients will be randomised in a 1:1:1 ratio to one of the three treatment groups, using permuted blocks without stratification. Randomisation will be performed using a centralised internet service, hosted by the Pragmatic Clinical Trials Unit. The schedule of intervention with timeline is detailed below.

7.4. Blinding

When a participant is randomised the randomisation system will only display whether they have been allocated to an "app" treatment group (either the "Intervention" or "Active Control" group, but not which one) or the "Treatment as usual" group. If a participant is randomised to either "app" treatment group, then the randomisation system will supply an alphanumeric token which is redeemed when registering to receive the app. This will ensure that the correct content (mindfulness meditation or progressive muscle relaxation) is delivered to each participant. Therefore, the participant and recruiting staff will NOT be blinded to allocation of the "Treatment as usual" or "app" groups. However, at randomisation they will be blinded to whether allocation is to "Intervention" or "Active Control" group.

To preserve blinding of participants as much as possible, "Intervention" and "Active Control" groups will be using the same app, and hearing instructions for the same duration, delivered by the same narrator. Only the content of the instructions will differ. In addition, the Patient Information Sheet and consent form do not explicitly refer to "mindfulness meditation" or "progressive muscle relaxation".

Outcomes are collected in paper questionnaires completed by participants. The 6 month questionnaire includes a question to determine whether the participants randomised to the app have been unblinded to the "Intervention" app or "control" app. The researcher will answer a short questionnaire after recruiting each participant to determine if they have been unblinded to the "Intervention" app or "control" app, for participants randomised to an app.

Statisticians will be blinded to individual treatment allocations until required for the final analysis. If necessary, an independent statistician will perform any interim analysis which require unblinding of the data.

Forbes G, et al. BMJ Open 2020; 10:e030164. doi: 10.1136/bmjopen-2019-030164

It is not anticipated that any emergency unbinding will be necessary.

7.5. Planned interventions

After eligible women have been allocated to one of the 3 groups, the participants in the Intervention and the Active Control group (progressive muscle relaxation app) will receive a face-to-face introduction to using the app. After that, the Intervention group will use the app over 60 days.

The meditation content is a structured and progressive course, layering in new techniques and concepts over successive sessions. The course was created and narrated by a former monk - Andy Puddicombe - drawing on a secularised version of the techniques he was taught over 10 years' experience in monasteries around the world.

The techniques used in the Intervention are shown in the table below. The first 30 days cover basic techniques, assuming no previous experience of meditation. The second 30 days focus specifically on the use of these techniques with respect to pain. The duration of individual sessions builds over time. Days 1-10 are 10 minutes in duration, days 11-20 are 15 minutes in duration, and days 21-60 are 20 minutes in duration.

The Active Control group will use the same app, but the app will be configured so that they will hear a series of non-meditative progressive muscle relaxation instructions, also narrated by Andy Puddicombe. These sessions will be identical every day, except that their duration will increase to mirror the increasing duration of the meditation content being listened to by the Intervention group.
In this way, both Intervention and Control groups will be using the same app, and hearing instructions for the same duration, delivered by the same narrator. Only the content of the instructions will differ.

Barts and The London
School of Medicine and Dentistry

Series	Techniques involved
Take 10/Foundation 1 (first 10 days)	Open monitoring, body scan, breath as anchor
Foundation 2 (days 11-20)	As above, plus intention and altruism
Foundation 3 (days 21-30)	As above, plus integration of mindfulness with daily activities
Pain series (days, 31-60)	As above, plus visualisation and enquiry (insight/Tibetan vipassana)

7.6. Concomitant Medications

Patients are able to receive any concomitant medications that they would as part of usual care.

7.7. Reasons for non progression to full trial

- Insurmountable problems with recruitment
- Extremely high rates of loss-to-follow-up
- Extremely low rates of adherence to the intervention
- Unacceptability of intervention for patients

7.8. Key risks to delivering this research and contingencies:

- Recruitment of 90 patients between May 2016 and October 2016 not achieved – regular monitoring throughout recruitment period to identify and resolve problems (e.g. open new centres/extend recruitment period)
- We will monitor regularly if patients have not downloaded apps and offer further one-to-one support
- Data collection issues will be monitored and addressed early where possible; this will inform the full-scale RCT design

Forbes G, et al. BMJ Open 2020; 10:e030164. doi: 10.1136/bmjopen-2019-030164

- Issues relating to the other milestones (ethics, personnel, app availability) and deliverables will be rectified, but potentially delay the start of MEMPHIS/full-scale trial. Contamination was not thought likely by the patient group

7.9. Procedure for Collecting Data

Patients will enter the data on paper questionnaires, which will be transferred into a purpose-built electronic database.

1.) Scales for clinical outcomes
2.) App satisfaction questionnaire, which includes open comment boxes and tick-boxes based on published questionnaires [30].

As an incentive to complete and return the patient questionnaires, a £5 shopping voucher will be sent in the post with each follow up questionnaire alongside a stamped addressed envelope.
In the case that a questionnaire is not received, participants will be sent a text reminder. Non-responders will then be contacted by telephone in order to collect a smaller dataset.

7.10. Including Case Report Forms (CRFs) and storage

In line with GCP guidance we will keep the data stored for 20 years following the close of the study to allow for verification and any further data sharing e.g. individual patient data meta-analysis.

We will follow the PCTU's standard operating procedures for legacy archiving.
Queen Mary University of London will act as custodians of the data.

7.11. Follow-up Procedures

Some of the participants will be asked for permission to elaborate on the open comment boxes about app satisfaction and also on clinical outcomes in two focus

groups to be held after the 6 month follow up point finisheswith participants asked to discuss and prioritise outcomes. Alternatively, participants unable to attend focus groups will be given the option to answer a questionnaire over the phone with a researcher.

7.12. Subject withdrawal (including data collection / retention for withdrawn participants)

A participant can be withdrawn from the trial if, in the opinion of the investigator or the care providing clinician or clinical team, it is medically necessary to do so.

With any post randomisation exclusions, the study personnel will make every effort to obtain, and record, information about the reasons for violation, any adverse events and to follow-up the women for all safety and efficacy outcomes, as appropriate. If a woman decides after randomisation she does not wish to participate any further in the MEMPHIS trial, she may withdraw herself from the trial. We will aim to document the reason for self-withdrawal. Clear distinction will be made as to whether the participant is withdrawing from trial whilst allowing further follow-up, or whether the participant refuses any follow-up. If a participant explicitly withdraws consent to have any further data recorded their decision will be respected and recorded on the final study form. All communication surrounding the withdrawal will be noted in the study records and no further data will be collected for that participant. They will be returned to the NHS standard practice for follow up care.

If a woman loses their ability to consent during participation in the trial, they will be withdrawn from the trial and no further data will be collected from the participant unless consent for this was explicitly obtained prior to the loss of capacity.

7.13. Continued app use after trial period and app use by treatment as usual group

It was decided to permit continued app use to the end of the study to reflect the situation in real life. Duration of use will be recorded through the app without using patient identifiable data.

Forbes G, et al. BMJ Open 2020; 10:e030164. doi: 10.1136/bmjopen-2019-030164

Barts and The London
School of Medicine and Dentistry

Consideration was given to inform patients in the 'treatment as usual' arm at the beginning that they will be able to access the meditation app at the end of the study, but this was abandoned due to concerns that this could lead to bias. Research has shown [31] that in those circumstances patients may decide to 'wait' until the end of the intervention before trying to improve, and as a consequence, they tend to improve less, leading to overestimating the effect of the intervention. It is possible that without the offer of delayed app use recruitment may be slower, which is something we would like to determine in the feasibility study. However, if after close involvement with the PPI this appears to be not acceptable to patients as compromise such as telling control patients after the end of the study that they are now allowed to use the app may be offered.

7.14. Schedule of Assessment

Health outcome measures are collected at baseline. The delivery of the intervention or control will occur for 60 days. Health outcome measures are collected immediately after the intervention at 60 days, and again at 3 and 6 months. App satisfaction/usability measures will be collected immediately after the intervention at 60 days from app participants.

The usability and clinical outcome focus groups will take place after the 6 month follow up point.

Assessment	Baseline	During intervention	60 days post randomisation	3 months post randomisation	6 months post randomisation
Questions about participants pain	V				
History of pain treatment	V				
Personal details	V				
Adherence to app use		V			
Clinical outcome questionnaires	V		V	V	V

Forbes G, *et al. BMJ Open* 2020; 10:e030164. doi: 10.1136/bmjopen-2019-030164

Barts and The London School of Medicine and Dentistry

App satisfaction questionnaires			V		
Interview/focus group with recruiters, nurses, patients, other stakeholders on usability and integration into practice					V
HCP and patient focus groups on clinical outcomes					V

Forbes G, *et al. BMJ Open* 2020; 10:e030164. doi: 10.1136/bmjopen-2019-030164

7.15. Criteria for Early Termination of the study

The nature of the intervention and follow−up makes it unlikely that any new information will impact an individual participant. If the TSC committee, REC, CI or sponsor determine it is within the best interests of the participants or trial to terminate the study, written notification will be given to the CI. This may be due to, but not limited to; safety concerns, proof of efficacy or non-compliance/serious breaches. If the study is terminated participants will be returned to the NHS normal follow up and routine care.

7.16. End of Study Definition

When the last enrolled participant has completed follow up, the REC will be notified of the trial completion. The final study report will be completed within 12 months after the trial completion.

8. STATISTICAL CONSIDERATIONS

8.1. Sample Size

30 participants will be recruited to each of the three treatment groups, giving a total of 90 participants. As this is a feasibility study, we have not performed a sample size calculation based upon the power to detect a significant treatment effect on a clinical outcome. However, 90 participants should provide a reliable estimate for the standard deviation of the primary clinical outcome (likely to be pain acceptance) [32, 33], which can be used to inform the sample size calculation of the main trial.

8.2. Statistical Analysis

A full analysis plan will be developed and agreed prior to any analysis or unblinding of the data.

Forbes G, et al. BMJ Open 2020; 10:e030164. doi: 10.1136/bmjopen-2019-030164

Barts and The London
School of Medicine and Dentistry

Baseline

Baseline variables will be presented for each treatment group as the mean (SD) or median (IQR) for continuous variables, and the number (%) for categorical variables.

Analysis of Feasibility Outcomes

Feasibility outcomes will be presented for each treatment group as the mean (SD) or median (IQR) for continuous variables, and the number (%) for categorical variables.

Duration of recruitment will be calculated as the number of days from the beginning to the end of recruitment. The number of participants recruited per month will be presented.

The proportion of patients in each treatment group who have returned data at each follow-up time point (60 days, 3 months, and 6 months post-randomisation) will be presented. Summaries of baseline variables will be presented separately for patients who have and have not returned data at each at the 6 month time point.

Adherence outcomes will be summarised separately for the intervention and active control treatment groups. Adherence outcomes will be presented as the mean (SD) or median (IQR) for continuous variables, and the number (%) for categorical variables.

An estimate of the standard deviation of pain acceptance (CPAQ) in each treatment group at each follow up time point (60 days, 3 months, and 6 months) will be presented.

Analysis of Clinical Outcomes

For each clinical outcome we will present the following information:

- The number of patients in each treatment group with an observed outcome at each follow-up time point.

- The mean (SD) in each treatment group at each follow-up time point.
- The estimated treatment effect at each follow-up time point, with a 95% confidence interval.

Estimates of treatment effect will be presented comparing the intervention group (mindfulness meditation app) to the control (treatment as usual) group, the intervention group to the active control (progressive muscle relaxation app) group, and the active control group to the control (treatment as usual) group. Outcomes will be analysed using linear mixed-effects models to account for the correlation between patient outcomes at different follow-up time points [34], and adjusted for baseline measure of the outcome [35]. Patient data will be analysed according to the treatment group to which they were randomised (intention-to-treat). All patients with an observed outcome for at least one of the three follow-up time points (60 days, 3 months, or 6 months) will be included in the analysis [36].

Analysis of usability and integration of app

- Obstacles to recruitment will be summarised
- The integration of the app into existing and emerging patient pathways will be investigated using questionnaires developed from social contagion theory and Normalisation Process Theory (NPT) as described in section 5.3. The maximum total score using NPT is 64. The maximum total score using the Diffusion of Innovations questionnaire is 200.
  The System Usability Scale (SUS) [28] has a maximum score of 50.

9. ETHICS

The Investigator to an Independent Research Ethics Committee will submit this protocol and any subsequent amendments, along with any accompanying material provided to the participant in addition to any advertising material. Written Approval from the Committee will be obtained and subsequently submitted to the JRMO to

Forbes G, *et al. BMJ Open* 2020; 10:e030164. doi: 10.1136/bmjopen-2019-030164

obtain Final R&D approval. The trial can only start after approval from a Research Ethics Committee and the local R&D "Sign-off" from the participating centre. If there is any further safety information, which may result in significant changes in the risk/benefit analysis, the Patient Information Sheet (PIS) and Informed Consent Form (ICF) will be amended accordingly and submitted to REC for revision and approval. All participants that are actively enrolled on the study will be informed of the updated information and given a revised copy of the PIS/ICF in order to confirm their wish to continue on the study (if feasible), if it may change their willingness to participate.

10. SAFETY CONSIDERATIONS

There are no known side effects arising from mindfulness meditation.

11. DATA HANDLING AND RECORD KEEPING

11.1. Confidentiality

Patient anonymity is protected and maintained. This applies to data collected on paper or via the headspace database.

We will ensure that patient identities are protected from any unauthorised parties. Information with regards to study patients will be kept confidential and managed in accordance with data Protection Act, NHS Caldicott Guardian, The research Governance Framework for Health and Social care and Research Ethics Committee Approval.

The trial will collect personal data and sensitive information about the participants either directly or from their clinical team. Participants will be informed about the transfer of this information to the study office and will be asked to consent to this. The data will be entered onto a secure computer database, either by trials unit staff or directly via a secure Internet connection. Any data to be processed will be anonymised. All personal information obtained for the trial will be held securely and treated as (strictly) confidential. All staff, at the hospital or the trials unit shares the

Forbes G, *et al. BMJ Open* 2020; 10:e030164. doi: 10.1136/bmjopen-2019-030164

same duty of care to prevent unauthorised disclosure of personal information. No data that could be used to identify an individual will be published.

In relation to the data collected by Headspace the following applies:

Headspace will not collect any clinical data, but data on app usage. Details collected on the headspace database will be confidential. Details about the individual's use of Headspace tools will never be seen by or shared with anyone outside the research team and the company. Individual usage and demographic information will only be used by Headspace in accordance with the standard Headspace user terms and conditions. No data will be shared with any other organizations, unless with prior agreement, and all data is kept confidential. App usage data will be transferred to the research team via a securely encrypted file.

The Chief investigator, Miss Elizabeth Ball is the "custodian" of the data.

11.2. Required Study Documents

- A signed protocol and any subsequent amendments
- PCTU self-monitoring template for the trial team to complete on a regular basis as detailed by the Trial Monitoring section
- Current and Superseded Patient Information Sheets
- Current and Superseded Consent Forms
- Current and Superseded GP letters
- Current and Superseded Posters
- Current and Superseded CRFs
- Indemnity documentation from sponsor
- Conditions of Sponsorship from sponsor
- Conditional/Final R&D Approval
- Signed site agreements
- Ethics submissions/approvals/correspondence
- CVs and GCP certificates of CI and site staff

Forbes G, *et al. BMJ Open* 2020; 10:e030164. doi: 10.1136/bmjopen-2019-030164

- Laboratory accreditation letter, certification and normal ranges for all laboratories to be utilised in the study
- Delegation log
- Staff training log
- Identification log
- Enrolment log
- Monitoring visit log
- Correspondence relating to the trial
- SAE reporting plan for the study

11.3. Record Retention and Archiving

During the course of research, all records are the responsibility of the Chief Investigator and must be kept in secure conditions. When the trial is complete, it is a requirement of the Research Governance Framework and Trust Policy that the records are kept for a further 20 years. For trials involving Barts Health Trust patients, undertaken by Trust staff, or sponsored by Barts Health trust or QMUL, the approved repository for long-term storage of local records is the Trust Modern Records centre, which is based at 9 Prescott Street.

12. PRODUCTS, DEVICES, TECHNIQUES AND TOOLS

12.1. Devices

The Medicines and Healthcare products Regulatory Agency (MHRA) states that some apps can be classified as medical devices. [37]

However, apps with software that provides general information but does not provide personalised advice, although it may be targeted to a particular user group, is unlikely to be considered a medical device. We believe that neither the mindfulness meditation nor the progressive muscle relaxation content in the app fulfil the criteria for medical devices.

12.2. Techniques and interventions

Intervention (mindfulness meditation content):

60 days of guided meditation content. The first 30 days cover basic techniques, assuming no previous experience of meditation. The second 30 days focus specifically on the use of these techniques with respect to pain. The duration of individual sessions builds over time. The first 10 days are each 10 minutes in duration. The next 10 days are each 15 minutes in duration. All following days are 20 minutes in duration. The minimum usage of app should be for at least 22 out of 60 days.

It was decided to permit continued app use to the end of the study to reflect the situation in real life. Duration of use will be recorded through the app without using patient identifiable data.

Control:

1) Treatment as usual (watch and wait, medication and/or surgery) to investigate if any app intervention makes a difference to wellbeing and to ascertain dropout rates for the full-scale trial in patients who perceive that they are getting no intervention

2) 60 days of progressive muscle relaxation content: This group will use the same app as the Intervention group, but the app will be configured so that they will hear a series of non-meditative progressive muscle relaxation instructions. These sessions will be identical every day, except that their duration will increase to mirror the increasing duration of the meditation content being listened to by the Intervention group (10 minutes a day for 10 days, then 15 minutes a day for 10 days, then 20 minutes a day thereafter.)

App satisfaction questionnaires

- Purpose made questionnaire (Carol Rivas)
- The System Usability Scale (SUS) [28]

Forbes G, *et al. BMJ Open* 2020; 10:e030164. doi: 10.1136/bmjopen-2019-030164

13. SAFETY REPORTING

13.1. Adverse Events (AE)

An AE is any untoward medical occurrence in a subject to whom a medicinal product has been administered, including occurrences which are not necessarily caused by or related to that product. An AE can therefore be any unfavourable and unintended sign (including an abnormal laboratory finding), symptom or disease temporarily associated with study activities.
We do not expect SAEs related to use of the mindfulness or the progressive muscle relaxation app.

Notification and reporting Adverse Events or Reactions
If the AE is not defined as SERIOUS, the AE is recorded in the study file and the participant is followed up by the research team. The AE is documented in the participants' medical notes (where appropriate) and the CRF.

13.2. Serious Adverse Event (SAE)

A serious adverse event (SAE) is defined as an untoward occurrence that:

(a) results in death;
(b) is life-threatening;
(c) requires hospitalisation or prolongation of existing hospitalisation;
(d) results in persistent or significant disability or incapacity;
(e) consists of a congenital anomaly or birth defect; or
(f) is otherwise considered medically significant by the investigator.

An SAE occurring to a research participant should be reported to the main REC where in the opinion of the Chief Investigator the event was:

• Related – that is, it resulted from administration of any of the research procedures, and

Forbes G, et al. BMJ Open 2020; 10:e030164. doi: 10.1136/bmjopen-2019-030164

• Unexpected – that is, the type of event is not listed in the protocol as an expected occurrence.

Notification and Reporting of Serious Adverse Events

Serious Adverse Event (SAEs) that are considered to be 'related' and 'unexpected' are to be reported to the sponsor within 24 hours of learning of the event and to the Main REC within 15 days in line with the required timeframe. For further guidance on this matter, please refer to NRES website and JRMO SOPs

13.3. Urgent Safety Measures

The CI may take urgent safety measures to ensure the safety and protection of the clinical trial subjects from any immediate hazard to their health and safety,. The measures should be taken immediately. In this instance, the approval of the REC prior to implementing these safety measures is not required. However, it is the responsibility of the CI to inform the sponsor and Main Research Ethics Committee (via telephone) of this event immediately.

The CI has an obligation to inform both the Main REC in writing within 3 days, in the form of a substantial amendment. The sponsor (Joint Research Management Office (JRMO)) must be sent a copy of the correspondence with regards to this matter. For further guidance on this matter, please refer to NRES website and JRMO SOPs.

13.4. Annual Safety Reporting

The CI will send the Annual Progress Report to the main REC using the NRES template (the anniversary date is the date on the MREC "favourable opinion" letter from the MREC) and to the sponsor. Please see NRES website and JRMO SOP for further information

Forbes G, *et al. BMJ Open* 2020; 10:e030164. doi: 10.1136/bmjopen-2019-030164

Barts and The London
School of Medicine and Dentistry

13. 5. Overview of the Safety Reporting responsibilities

The CI/PI has the overall pharmaco-vigilance oversight responsibility. The CI/PI has a duty to ensure that safety monitoring and reporting is conducted in accordance with the sponsor's requirements.

14. MONITORING & AUDITING

14.1. Auditing

Definition: "A systematic and independent examination of trial related activities and documents to determine whether the evaluated trial related activities were conducted, and the data were recorded, analysed and accurately reported according to the protocol, sponsor's standard operating procedures (SOPs), Good Clinical Practice (GCP), and the applicable regulatory requirement(s)."

A study may be identified for audit by any method listed below:

1. A project may be identified via the risk assessment process.
2. An individual investigator or department may request an audit.
3. A project may be identified via an allegation of research misconduct or fraud or a suspected breach of regulations.
4. Projects may be selected at random. The Department of Health states that Trusts should be auditing a minimum of 10% of all research projects.
5. Projects may be randomly selected for audit by an external organisation.

Internal audits may be conducted by a sponsor's or funder representative.

14.2. Summary Monitoring Plan

Investigators and their host Trusts will be required to permit study-related monitoring and audits to take place, providing direct access to source data and documents as requested. Trusts may also be subject to inspection by the Research and Development Manager and should do everything requested by the Chief Investigator in order to

Forbes G, *et al. BMJ Open* 2020; 10:e030164. doi: 10.1136/bmjopen-2019-030164

prepare and contribute to any inspection or audit. Study participants will be made aware of the possibility of external audit of data they provide in the participant information sheet.

14.3. Compliance

The CI will ensure that the trial is conducted in compliance with the principles of the Declaration of Helsinki (1996), and in accordance with all applicable regulatory requirements including but not limited to the Research Governance Framework, GCP, Trust and Research Office policies and procedures and any subsequent amendments.

14.4. Non-Compliance

Definition: A noted systematic lack of both the CI and the study staff adhering to the principles of the Declaration of Helsinki (1996), applicable regulatory requirements including but not limited to the Research Governance Framework, GCP, Trust and Research Office policies and procedures and any subsequent amendments, which leads to prolonged collection of deviations, breaches or suspected fraud.

These non-compliances may be captured from a variety of different sources including monitoring visits, CRFs, communications and updates. The sponsor will maintain a log of the non-compliances to ascertain if there are any trends developing or escalating. The sponsor will assess the non-compliances and action a timeframe in which they need to be dealt with. Each action will be given a different timeframe dependent on the severity. If the actions are not dealt with accordingly, the sponsor will agree an appropriate action, including an on-site audit.

15. TRIAL COMMITTEES

15.1. Trial Steering Committee (TSC)

The TSC provides independent supervision for the trial, providing advice to the Chief and Co-Investigators and the Sponsor on all aspects of the trial and affording

Forbes G, *et al. BMJ Open* 2020; 10:e030164. doi: 10.1136/bmjopen-2019-030164

protection for patients by ensuring the trial is conducted according to the principles of Good Clinical Practice in Clinical Trials. If the Chief and Co-Investigators are unable to resolve any concern satisfactorily, Principal Investigators, and all others associated with the trial, may write through the Trial Unit to the chairman of the TSC, drawing attention to any concerns they may have about the possibility of particular side-effects, or of particular categories of patient requiring special study, or about any other matters thought relevant.

15.2. Trial Management Group (TMG)

The trial management group will meet regularly to discuss operational issues. This will include the chief investigator, trial co-ordinator, senior research manager, statistician, data manager, QA manager and research administrator.

15.3. Data Monitoring Committee (DMC)

Based on the short duration of recruitment (expected to be 6 months) and the safety profile of the intervention, a DMC will not be used.

16. FINANCE AND FUNDING

-This study is funded by the Research for Patients Benefit national programme (RfPB).
- Headspace is donating subscriptions at no charge as part of their research initiative.

17. INDEMNITY

Queen Mary, University of London will act as a Sponsor, as defined by the Research Governance Framework for Health and Social Care (April 2005) for the project. The project will also be covered by the sponsor's insurance brokers on a "No Faults Compensation for Clinical Trials and/or Human Volunteer Studies". This policy will

Forbes G, et al. BMJ Open 2020; 10:e030164. doi: 10.1136/bmjopen-2019-030164

indemnify/cover the insured in respect of their legal liabilities arising out of the insured's activities.

18. DISSEMINATION OF RESEARCH FINDINGS

The research findings of the feasibility study will be disseminated judiciously to avoid biasing the full-scale trial. In both trials we will disseminate our findings to:

1) Study participants through a dedicated website and newsletters at the end of the feasibility and full scale study, guided by our lay advisers

2) Participating health care professionals through the dedicated website and electronic newsletters

4) Professional groups via peer-reviewed journals and scientific meetings. Post-trial workshops run in collaboration with PPI group

5) Health service commissioners via the study website and an electronic newsletter

6) The wider public through local and national media and via dedicated website

7) Patients and relatives through PPI group
Applicants have links for dissemination via these organisations: Cochrane reviews, NICE, Pelvic pain support network (Judy Birch), Katherine Twining Network (KTN), BJOG (Khalid Khan), BSGE (Elizabeth Ball) Communications experts at our higher education institutions and the NIHR Collaboration for Leadership in Applied Health Research and Care North Thames will support our dissemination strategy through Twitter, Facebook and press coverage.

A particular strength of our application is our close links with:

Forbes G, *et al. BMJ Open* 2020; 10:e030164. doi: 10.1136/bmjopen-2019-030164

1) KTN, dedicated to research and education in the UK and abroad via the East London International Women's Health Appeal, who will be able to disseminate this low cost-intervention in developing countries with high incidence of CPP [2]

2) UCL partners, whose focus is on patient-led population-focused delivery of research innovations.

Forbes G, *et al. BMJ Open* 2020; 10:e030164. doi: 10.1136/bmjopen-2019-030164

Barts and The London
School of Medicine and Dentistry

REFERENCES

1. Royal College of Obstetricians and Gynaecologists, green top guidelines (2012) Available at: https://www.rcog.org.uk/en/guidelines-research-services/guidelines/gtg41 (Accessed: 13 May 2015)

2. Latthe, P., Latthe, M., Say, L., Gülmezoglu, M., Khan, K.S., (2006), 'WHO systematic review of prevalence of chronic pelvic pain: a neglected reproductive health entity', BMC Public Health. , 6(6), pp. 177.

3. Howard, F.M., (1993), 'The role of laparoscopy in chronic pelvic pain: promise and pitfalls', Obstetrical and Gynecological Survey, 48(6), pp. 357-387.

4. Simoens, S., et al., (2012), 'The burden of endometriosis: costs and quality of life of women with endometriosis and treated in referral centres', Human Reproduction, 27(5) pp. 1292-1299

5. Stones, R.W. and Price, C., (2002), 'Health services for women with chronic pelvic pain', Journal of the Royal Society of Medicine, 95, pp. 531-535

6. Peters, A.A., et al., (1991), 'A randomized clinical trial to compare two different approaches in women with chronic pelvic pain', Obstetrics and Gynecology, 77(5), pp. 740-4

7. Warsi, A., et al., (2004), 'Self-management education programs in chronic disease: a systematic review and methodological critique of the literature', Archives of Internal Medicine, 164(15), pp. 1641-9.

8. Fox, S.D., Flynn, E., Allen, R.H., (2011), 'Mindfulness meditation for women with chronic pelvic pain: a pilot study', The Journal of reproductive medicine, 56(3-4), pp. 158-162.

9. Kold, M., et al., (2012), 'Mindfulness-based psychological intervention for coping with pain in endometriosis', Nordic Psychology, 64(1), pp. 2-16.

10. Cramer, H., et al., (2012), 'Mindfulness-based stress reduction for low back pain. A systematic review', BMC Complementary & Alternative Medicine, 12, pp. 162.

11. Kozasa, E.H., et al., (2012), 'The effects of meditation-based interventions on the treatment of fibromyalgia', Current Pain & Headache Reports, 16(5), pp. 383-7.

12. Veehof, M.M., et al., (2011), 'Acceptance-based interventions for the treatment of chronic pain: A systematic review and meta-analysis', Pain, 152(3), pp. 533-42

Forbes G, *et al. BMJ Open* 2020; 10:e030164. doi: 10.1136/bmjopen-2019-030164

13. Higgins, J.P.T., Green, S., (2011) 'Cochrane handbook for systematic reviews of interventions', version 5.1.0. Cochrane Collaboration. Available at: www.cochrane-handbook.org (Accessed: 13 May 2015)

14. Brown, C.A. and Jones, A.K., (2013), 'Psychobiological correlates of improved mental health in patients with musculoskeletal pain after a mindfulness-based pain management program', Clinical Journal of Pain, 29(3), pp. 233-44.

15. Carson, J., et al., (2005), 'Loving-kindness meditation for chronic low back pain: results from a pilot trial', Journal of Holistic Nursing, 23(3), pp. 287-304.

16. Morone, N.E., Greco, C.M., Weiner, D.K., (2008), 'Mindfulness meditation for the treatment of chronic low back pain in older adults: a randomized controlled pilot study', Pain, 134(3), pp. 310-9.

17. Nash-Mc Feron, D.E., (2006), 'Mindfulness in the treatment of chronic headache pain', Dissertation Abstracts International: Section B: The Sciences and Engineering, 67(5-B), pp. 2841.

18. Plews-Ogan, M., et al., (2005), 'A pilot study evaluating mindfulness-based stress reduction and massage for the management of chronic pain', Journal of General Internal Medicine, 20(12), pp. 1136-8.

19. Sagula, D. and Rice, K.G., (2004), 'The effectiveness of mindfulness training on the grieving process and emotional well-being of chronic pain patients', Journal of Clinical Psychology in Medical Settings, 11(4), pp. 333-342.

20. Schmidt, S., et al., (2010), 'Assessing a dynamical EEG pattern related to chronic pain-Results from a controlled evaluation of back pain patients as well as from an observational trial with a mindfulness based intervention', European Journal of Integrative Medicine, 2 (4), pp. 196.

21. Sephton, S.E., et al., (2007), 'Mindfulness meditation alleviates depressive symptoms in women with fibromyalgia: Results of a randomized clinical trial', Arthritis Care and Research, 57(1), pp. 77-85.

22. Teixeira, E., (2010), 'The effect of mindfulness meditation on painful diabetic peripheral neuropathy in adults older than 50 years', Holistic Nursing Practice, 24(5), pp. 277-83.

23. Lakhan, S.E. and Schofield, K.L., (2014), 'Mindfulness-Based Therapies in the Treatment of Somatization Disorders: A Systematic Review and Meta-Analysis', PLOS One, 8(8): e71834

Forbes G, *et al. BMJ Open* 2020; 10:e030164. doi: 10.1136/bmjopen-2019-030164

24. Goyal, M., et al., (2014), 'Meditation Programs for Psychological Stress and Well-being', JAMA Internal Medicine., 174(3), pp. 357-368

25. Krusche, A., Cyhlarova, E., Williams, J.M., (2013), 'Mindfulness online: an evaluation of the feasibility of a web-based mindfulness course for stress, anxiety and depression', BMJ Open., 3(11): pp. e003498.

26. http://bps.mapofmedicine.com/evidence/bps/chronic_pelvic_pain_for_men_and_women_1.html

27. May, C., Sibley, A., Hunt, K., (2013) 'The nursing work of hospital-based clinical practice guideline implementation: an explanatory systematic review using normalisation process theory', International Journal of Nursing studies, 51(2), pp.289-99

28. Rogers, E.M., (2003), Diffusion of Innovations, Fifth Edition, Free Press, New York, pp.221

29. Measuring Usability With The System Usability Scale (SUS), (2011) Available at: http://www.measuringu.com/sus.php, (Accessed: 13 May 2015)

30. Spook, J.E., et al., (2013), 'Monitoring Dietary Intake and Physical Activity Electronically: Feasibility, Usability, and Ecological Validity of a Mobile-Based Ecological Momentary Assessment Tool', Journal of Medical Internet Research, 15(9), pp. e214.

31. Cunningham, J.A., Kypros, K., McCambridge, J., (2013), 'Exploratory randomized controlled trial evaluating the impact of a waiting list control design', BMC Medical Research Methodology, 13(150)

32. Lancaster, G.A., Dodd, S., Williamson, PR., (2004), 'Design and analysis of pilot studies: recommendations for good practice', Journal of Evaluation in Clinical Practice, 10(2), pp.307-12

33. Teare, MD., Dimairo, M., Shephard, N., Hayman, A., Whitehead, A., Walters, SJ., (2014), ´Sample size requirements to estimate key design parameters from external pilot randomised controlled trials: a simulation study´, Trials, 15(1), pp.264.

34. Rabe-Hesketh, S., Skrondal, A., (2012), Multilevel and Longitudinal Modeling Using Stata, Third Edition, College Station, Texas: Stata Press.

Forbes G, *et al. BMJ Open* 2020; 10:e030164. doi: 10.1136/bmjopen-2019-030164

35. Kahan BC, Jairath V, Dore CJ, Morris TP., (2014), ´The risks and rewards of covariate adjustment in randomized trials: an assessment of 12 outcomes from 8 studies´, Trials, 15, pp.139.

36. White, IR., Horton, NJ., Carpenter, J., Pocock, SJ., (2011), ´Strategy for intention to treat analysis in randomised trials with missing outcome data`, BMJ, 342:d40.

37. Medicines and Healthcare products Regulatory Agency (2014) Medical device stand-alone software including apps. Available at: https://www.gov.uk/government/publications/medical-devices-software-applications-apps/medical-device-stand-alone-software-including-apps (Accessed: 13 May 2015)

Forbes G, *et al. BMJ Open* 2020; 10:e030164. doi: 10.1136/bmjopen-2019-030164

APPENDICES

Appendix 1 – Information with regards to Safety Reporting in Non-CTIMP Research

	Who	When	How	To Whom
**SUSAR**	Chief Investigator	Report to the Sponsor, and QA manager within 24 hours<br>MREC within 15 days of learning of the event	SAE Report form for Non-CTIMPs, available from NRES website.	Sponsor and MREC
**Urgent Safety Measures**	Chief Investigator	Contact the Sponsor and MREC Immediately<br><br>Within 3 days	By phone<br><br>Substantial amendment form giving notice in writing setting out the reasons for the urgent safety measures and the plan for future action.	Main REC and Sponsor<br><br>Main REC with a copy also sent to the sponsor. The MREC will acknowledge this within 30 days of receipt.
**Progress Reports**	Chief Investigator	Annually (starting 12 months after the date of favourable opinion)	Annual Progress Report Form (non-CTIMPs) available from the NRES website	Main REC and Sponsor
**Declaration of the conclusion or early termination of the study**	Chief Investigator	Within 90 days (conclusion)<br><br>Within 15 days (early termination)<br><br>*The end of study should be defined in the protocol*	End of Study Declaration form available from the NRES website	Main REC with a copy to be sent to the sponsor
**Summary of final Report**	Chief Investigator	Within one year of conclusion of the Research	Where the study has met its objectives, the main findings and arrangements for publication or dissemination including feedback to participants	Main REC with a copy to be sent to the sponsor

Forbes G, *et al. BMJ Open* 2020; 10:e030164. doi: 10.1136/bmjopen-2019-030164
