# Supplementary material for: MEMPHIS: a smartphone app using psychological approaches for women with chronic pelvic pain presenting to gynaecology clinics: a randomised feasibility trial
Source: BMJ Open. 2020 Mar 12;10(3):e030164. doi: 10.1136/bmjopen-2019-030164 (PMC7069270; doi:10.1136/bmjopen-2019-030164)
Supplement: Supplementary data [file bmjopen-2019-030164supp002.pdf]

MEMPHIS

Statistical Analysis Plan

Version: 3.0
Date: 26/Jan/2017

Person(s) contributing to the analysis plan	
Name(s) and position(s)	Neil Wright (Statistician)<br>Brennan Kahan (Statistician)<br>Elizabeth Ball (CI)<br>Gordon Forbes (Statistician)
Authorisation	
Position	**Chief or principal investigator**
Name	Elizabeth Ball
Signature	
Date	26/01/2017
Position	**Senior trial statistician**
Name	Brennan Kahan
Signature	
Date	26/01/2017
Position	**Independent statistician**
Name	Gian Luca Di Tanna
Tick once reviewed	✓
Date	30/01/2017

Forbes G, *et al. BMJ Open* 2020; 10:e030164. doi: 10.1136/bmjopen-2019-030164

1. INTRODUCTION 3
2. STUDY METHODS 6
3. STUDY OUTCOMES 8
4. DATA COLLECTION 10
5. DERIVED VARIABLES 14
6. STATISTICAL ANALYSIS 15
7. GRAPHS AND FIGURES TO BE PRODUCED 19
8. REFERENCES 21
9. APPENDIX A: DERIVED AND COMPUTED VARIABLES 23
10. APPENDIX B: STATA CODE FOR GENERATING ADHERENCE OUTCOMES 29
11. APPENDIX C: STATA CODE FOR STATISTICAL ANALYSIS OF CLINICAL OUTCOMES ... 32
12. APPENDIX D: DRAFT TABLES 33
13. APPENDIX E: DATA / FILE MANAGEMENT 53

Forbes G, *et al. BMJ Open* 2020; 10:e030164. doi: 10.1136/bmjopen-2019-030164

1. INTRODUCTION

1.1. Purpose of statistical analysis plan

The purpose of this document is to provide details of the statistical analyses and presentation of results to be reported within the principal paper(s) of the MEMPHIS trial. Any exploratory, post hoc or unplanned analyses will be clearly identified in the respective study analysis report. This document does not detail the qualitative analysis, and so aims and outcomes that are collected for qualitative analyses only are not included.

This document has been developed prior to examination of trial data and will not be implemented prior to final approval. Statisticians will be blinded to individual treatment allocations until this statistical analysis plan has been approved, all trial data has been collected and the trial is complete.

This document is based on protocol version 8.0 (December 2016)

1.2. Members of the writing committee

Neil Wright (Statistician) was primarily responsible for writing the Statistical Analysis Plan, with input from Brennan Kahan (Senior Statistician). Neil Wright was responsible for writing the computer code to implement the analysis strategy. Elizabeth Ball (CI) and Julie Dodds also contributed to this Statistical Analysis Plan.

1.3. Summary

Short Title	MEMPHIS
Methodology	A randomised feasibility trial
Research Sites	This trial will be conducted at the Royal London and Whipps Cross Hospitals
Objectives/Aims	The overall aim is to assess the feasibility of implementing a trial of a mindfulness meditation intervention delivered by a mobile phone app for patients with chronic pelvic pain (CPP). The primary objectives are:<br><br>To provide feasibility data for a large multicentre RCT aimed at rigorously testing mindfulness meditation in CPP<br><br>To determine whether this app can be seamlessly integrated into clinical practice, especially CPP pathways
Number of Participants/Patients	90 women with CPP will be recruited and each randomised into one of the three trial groups (meditation app, progressive muscle relaxation or no app).

Forbes G, *et al. BMJ Open* 2020; 10:e030164. doi: 10.1136/bmjopen-2019-030164

Main Inclusion Criteria	To be eligible for the MEMPHIS study, the women must:<br>Be age 18 or over<br>Have either organic or non-organic chronic pelvic pain lasting for 6 months or more<br>Have access to a personal computer or smartphone.<br>Understand simple spoken English
Statistical Methodology and Analysis	Feasibility outcomes will be summarised using descriptive statistics. Clinical outcomes will be analysed using linear mixed-effects models, and results will be presented as a difference in means and a 95% confidence interval.

1.4. Changes from planned analysis in the protocol

- In the protocol, the dropout rate is a feasibility outcome but is not defined. In this analysis plan, we define two feasibility outcomes as "the number and proportion of participants who never return or answer a follow-up questionnaire at 6 months post-randomisation" and "the number and proportion of participants who do not return a follow-up questionnaire, but do answer the questionnaire by phone at 6 month post-randomisation".

- In the protocol, duration of recruitment is described as "the number of days from the beginning to the end of recruitment". In this analysis plan, duration of recruitment is defined as "the number of days from the day recruitment opens until the day the 90th patient is randomised (inclusive of both end days)".

- In the protocol, "Sexual Health Outcomes score (as measured by Sexual Health Outcomes in Women Questionnaire (SHOW-Q))" is given as a clinical outcome. In this analysis plan, this is replaced by the SHOW-Q global score, for sexually active participants, and by the SHOW-Q pelvic interference score, for all participants.

1.5. Changes from SAP v1.0

- In section 1.4 of version 1.0 of the SAP we stated *"In the protocol, "Quality of life score (as measured by the RAND Short form (36) Health Survey (SF-36))" is given as a clinical outcome. In this analysis plan, this is replaced by four of the RAND SF-36 subscales: physical functioning, general health, social functioning, and pain."* This has now been removed from the SAP as the protocol has been updated to reflect the change in the way quality of life score is being measured.

- The definition of app use has been changed from "having completed at least 50% of a session" to "having completed at least 90% of a session" (section 3.1). The change was

Forbes G, *et al. BMJ Open* 2020; 10:e030164. doi: 10.1136/bmjopen-2019-030164

made due to Headspace, the data provider of the app usage data, only collecting data on sessions which were at least 90% complete.

1.6. Changes from SAP v2.0

- Added clarification to section 4.3 that data collected outside the recommended window for follow-up will still be included in analysis.
- In section 6.5.1, specified that the number of CRFs returned within the follow-up windows specified in section 4.3 will be summarised.
- Corrected scoring of CPAQ in Appendix A.
- Amended scoring of MYMOP in Appendix A so item scores are missing if the symptoms or activities are entered differently at follow up time points.

Forbes G, *et al. BMJ Open* 2020; 10:e030164. doi: 10.1136/bmjopen-2019-030164

2. STUDY METHODS

2.1. Study objectives

The overall aim is to assess the feasibility of implementing a trial of a mindfulness meditation intervention delivered by a mobile phone app for patients with chronic pelvic pain (CPP). The primary objectives are:

- To provide feasibility data for a large multicentre RCT aimed at rigorously testing Mindfulness meditation in patients with CPP.
- To determine whether this app can be seamlessly integrated into clinical practice, especially CPP pathways.

2.2. Overall study design and plan

MEMPHIS is a randomised feasibility trial. Eligible women will be randomised to one of the three treatment groups:

- Intervention: 60 days of the app delivering mindfulness meditation content (in addition to usual care).
- Active control: 60 days of the app delivering progressive muscle relaxation content (in addition to usual care).
- Treatment as usual: Usual care

2.3. Selection of study population

2.3.1. Inclusion Criteria

To be eligible for the MEMPHIS study, the women must meet the following criteria:

- Aged 18 or over
- Women with organic and non-organic chronic pelvic pain lasting for six months or more
- Be capable of understanding the information provided, with use of an interpreter if required and being able to understand simple English as is used in the app
- Give written informed consent

2.3.2. Exclusion Criteria

Patients who meet the following criteria are ineligible to participate:

- No access to a Personal computer or smartphone

Forbes G, *et al. BMJ Open* 2020; 10:e030164. doi: 10.1136/bmjopen-2019-030164

2.4. Method of treatment assignment and randomisation

After informed consent, patients will be randomised using a central, web-based system in a 1:1:1 ratio to one of the three treatment groups, using permuted blocks (of sizes 27, 30, 33) without stratification.

2.5. Sample size determination

30 participants will be recruited to each of the three treatment groups, giving a total of 90 participants. As this is a feasibility study, we have not performed a sample size calculation based upon the power to detect a significant treatment effect on a clinical outcome. However, 90 participants should provide a reliable estimate for the standard deviation of the primary clinical outcome (likely to be pain acceptance) [1, 2], which can be used to inform the sample size calculation of the main trial.

Forbes G, *et al. BMJ Open* 2020; 10:e030164. doi: 10.1136/bmjopen-2019-030164

3. STUDY OUTCOMES

3.1. Feasibility outcomes

- Duration of recruitment (measured from the day recruitment opens until the day the 90th patient is randomised)
- Estimates to be used for the sample size calculation of the phase III RCT:
  - The estimated SD at 60 days, 3 months, and 6 months post-randomisation for pain acceptance (as measured by the Chronic Pain Acceptance Questionnaire (CPAQ-8))
  - The number and proportion of participants who never return or answer a follow-up questionnaire at 6 months post-randomisation.
  - The number and proportion of participants who do not return a follow-up questionnaire, but do answer the questionnaire by phone at 6 month post-randomisation.
- Patient adherence to app use measured by the following outcomes:
  - Number of days (within the first 60 days from randomisation) a patient has used the app (with app use defined as having completed at least 90%% of a session).
  - Whether the patient has used the app on 22 or more days within the first 60 days from randomisation.
  - Number of weeks (within the first eight weeks from randomisation) a patient has used the app on three or more days.
  - Whether the patient has used the app on three or more days in 6 or more weeks (within the first eight weeks from randomisation).
  - Whether the patient has used the app on 22 or more days within the first 60 days from randomisation, AND used the app on three or more days in 6 or more weeks within the first eight weeks from randomisation.

3.2. App satisfaction questionnaires

At 60 days post-randomisation:

- System Usability Scale (SUS) score (0 [worst] – 100 [best])
- Reponses to the purpose made app satisfaction questionnaire

Forbes G, *et al. BMJ Open* 2020; 10:e030164. doi: 10.1136/bmjopen-2019-030164

3.3. Clinical outcomes

The following clinical outcomes at 60 days, 3 months, and 6 months post-randomisation:

- Pain acceptance score (as measured by the Chronic Pain Acceptance Questionnaire (CPAQ-8)) (0 [worst] – 48 [best])
- RAND Short form (36) Health Survey (RAND SF-36) scales:
  - Physical functioning (0 [worst] – 100 [best])
  - Pain (0 [worst] – 100 [best])
  - General health (0 [worst] – 100 [best])
  - Social functioning (0 [worst] – 100 [best])
- Depression score (as measured by the Hospital Anxiety and Depression Scale (HADS)) (0 [best] – 21 [worst])
- Anxiety score (as measured by HADS) (0 [best] – 21 [worst])
- Mindfulness score (as measure by the Cognitive and Mindfulness - Revised (CAMS – R) scale) (12 [worst] – 48 [best])
- Pain related disability score (as measured by the Chronic Pain Grade (CPG) disability subscale) (0 [best] – 100 [worst])
- Self efficacy score (as measured by the Pain Self-Efficacy Questionnaire (PSEQ)) (0 [worst] – 60 [best])
- Sexual Health Outcomes scores (as measured by the Sexual Health Outcomes in Women Questionnaire (SHOW-Q)):
  - SHOW-Q global score, for sexually active participants (0 [worst] – 100 [best])
  - SHOW-Q pelvic interference score, for all participants (0 [best] – 100 [worst])
- Subjective outcome score (as measured by the Measure Yourself Medical Outcome Profile (MYMOP)) (0 [best] – 6 [worst])

The following qualitative outcomes are not included in the Statistical Analysis Plan:

- Reasons for patient non-adherence to app use
- Obstacles to recruitment from participants and recruiting staff
- Usability/integration etc
- Determining primary/secondary outcomes of interest
- App satisfaction questionnaires for service providers

Forbes G, *et al. BMJ Open* 2020; 10:e030164. doi: 10.1136/bmjopen-2019-030164

4. DATA COLLECTION

This section describes the variables that will be collected during the trial to be used in the analysis described by this plan.

4.1. Collected at baseline only

The following variables will be collected for each participant at baseline only.

Demographic:

- Age
- Weight
- Height
- Living arrangements (Alone, With others)
- Employment status (Employed (full or part time, including self-employment), Unemployed and looking for work, At school or in full time education, Unable to work due to long term sickness, Looking after your home/family, Retired from paid work, Other)
- Age left full time education (I did not receive a formal education, Age 12 or less, Age 13 to 16, Age 17 to 19, Age 20 or over, I am still in full time education, Other)
- Ethnic group (White, Black, Central Asian, Middle Eastern, Southern Asian, Mixed, Other ethnic group, Do not wish to say)
- Do you smoke (Yes, No)
- Number of cigarettes per week
- Do you drink alcohol (Yes, No)
- Number of alcohol units per week

Prior and concurrent treatment:

- Treatment used in last six months: Acupuncture; Gabapentin; Amitriptyline; Biofeedback; Botox injection; Contraceptive pills/patch/ring; Exercise, yoga or pilates; Injections to suppress ovaries (e.g. Prostap, Zoladex); Herbal Medicine; Meditation or relaxation exercises; Massage; Nutrition/diet; Codeine or Morphine type painkillers; Nerve blocks; Over the counter medication; Physiotherapy; Psychological (talking) therapy; Transcutaneous Electrical Nerve Stimulation (TENS); Surgery; Other. (One variable for each: Yes, No.)
- Currently using pain treatment (Yes, No)

Participants' pain:

Forbes G, *et al. BMJ Open* 2020; 10:e030164. doi: 10.1136/bmjopen-2019-030164

- Length of pain (0-6 months, 7-12 months, 1-2 years, 3-5 years, 6-10 years, More than 10 years)
- Pain over the past week (0 [No pain] to 10 [Pain as bad as could be])

4.2. Randomisation details

The following variables for each participant will be held in the randomisation database.

- Date of randomisation
- Treatment group allocation

4.3. Collected at baseline and follow up

The following clinical outcome variables will be collected for each participant at baseline, 60 days, 3 months, and 6 months post-randomisation. We aim to collect 60 day follow up data between 46 and 74 days from randomisation, 3 month follow up date between 76 and 104 days and 6 month follow up data between 159 and 201 days. However, data collected outside these day ranges will be included in the analysis.

- Pain acceptance (as measured Chronic Pain Acceptance Questionnaire (CPAQ-8)) (4 variables)
- Short form (36) Health Survey (SF-36) (36 variables)
- Depression (as measured by the Hospital Anxiety and Depression Scale (HADS)) (7 variables)
- Anxiety (as measured by HADS) (7 variables)
- Mindfulness (as measure by the Cognitive and Mindfulness - Revised (CAMS – R) scale) (12 variables)
- Pain related disability (as measured by the Chronic Pain Grade (CPG) disability subscale) (3 variables)
- Self efficacy (as measured by the Pain Self-Efficacy Questionnaire (PSEQ)) (10 variables)
- Sexual Health Outcomes (as measured by Sexual Health Outcomes in Women Questionnaire (SHOW-Q)) (12 variables)
- Subjective outcome (as measured by Measure Yourself Medical Outcome Profile (MYMOP)) (4 variables)

Forbes G, *et al. BMJ Open* 2020; 10:e030164. doi: 10.1136/bmjopen-2019-030164

Date of visit / date completed and method of collection (return of postal questionnaire or via telephone) for each follow-up questionnaire will also be collected. When the follow-up questionnaire is answered via telephone, the variables for the Short form (36) Health Survey (SF-36), Self efficacy (as measured by the Pain Self-Efficacy Questionnaire (PSEQ)), and Sexual Health Outcomes (as measured by Sexual Health Outcomes in Women Questionnaire (SHOW-Q)) are not collected.

4.4. App usage data

App usage data will be received from Headspace, for all participants randomised to the Intervention or Active Control arms. The data will include variables for participant login token, duration of session, filename of session, date and time of completion. Each observation represents one user completing (at least 90% of) a mindfulness meditation or muscle relaxation session.

4.5. App satisfaction questionnaires

The following variables will be collected for participants randomised to an app arm, at 60 days post-randomisation:

- System Usability Scale (SUS) (10 variables)
- Purpose made questionnaire responses:
    - Nine statements with categorical response. (Totally disagree, Somewhat disagree, Neither agree nor disagree, Somewhat agree, Totally agree) (9 variables)
    - One question (Did you use the app every day? (Yes, No))

4.6. Unintentional unblinding of randomised treatment

After the participant has been randomised, the following variables will be collected from the researcher:

- Was the participant randomised to the app treatment arm? (Yes, No)
- If the participant was allocated to the app treatment arm, which app treatment do you believe the participant was randomised to? (Intervention app, Control app, Don't know)

At 6 months (between 159 and 201 days) post-randomisation, the following variables will be collected from the participant:

- Did you use the smartphone app for MEMPHIS? (Yes, No)

Forbes G, *et al. BMJ Open* 2020; 10:e030164. doi: 10.1136/bmjopen-2019-030164

- Do you think you received the new treatment or comparison treatment? (New Treatment, Comparison Treatment, Don't Know)

PCTU

Forbes G, et al. BMJ Open 2020; 10:e030164. doi: 10.1136/bmjopen-2019-030164

5. DERIVED VARIABLES

5.1. Feasibility outcomes

A participant is counted as never having returned follow-up questionnaire at 6 months post-randomisation if date of visit / date completed and all other fields in the follow-up questionnaire are missing.

The patient adherence to app use outcomes listed in Section 3.1 will be calculated from the app usage data described in Section 4.4. Completing a session that is at least ten minutes on a day counts as having used the app on that day. Sample Stata code showing the calculation of these outcome variables is given in APPENDIX B: STATA CODE FOR GENERATING ADHERENCE OUTCOMES.

In the app usage data, date and timestamps will be provided in Coordinated Universal Time (UTC). These will be converted to UK time (BST/GMT as appropriate) before outcomes are derived.

5.2. Clinical outcomes

Details for how the clinical outcome scores list in Section 3.3 are derived from question responses (Section 4.2) are given in APPENDIX A: DERIVED AND COMPUTED VARIABLES.

5.3. System Usability Score (SUS) score

Details for how the System Usability Scale (SUS) score is derived from question responses is given in APPENDIX A: DERIVED AND COMPUTED VARIABLES.

Forbes G, *et al. BMJ Open* 2020; 10:e030164. doi: 10.1136/bmjopen-2019-030164

6. STATISTICAL ANALYSIS

6.1. Analysis populations

All analyses will be carried out according to the intention-to-treat (ITT) principle: all patients with a non-missing outcome will be analysed according to the group to which they are randomised.

Summaries of patient adherence to app use will include all participants randomised to the intervention or active control treatment groups.

Sample means and SDs for clinical outcomes will include all participants with a non-missing outcome at that time point.

Analyses to estimate treatment effects for clinical outcomes (Section 6.4.2) will include all patients with a non-missing outcome for at least one of the three follow-up time points (60 days, 3 months, or 6 months) [3]. Patients with a missing outcome at all follow-up time points for a clinical outcome are excluded from the analysis of that clinical outcome. A clinical outcome is non-missing if there are recorded responses at that time point for all individual questions required for the derivation of the clinical outcome. (Note that for the Subjective outcome score (MYMOP profile score), only symptom 1 score and wellbeing score are required.)

6.2. Baseline variables

Demographic, prior and concurrent treatment, and participants' pain baseline variables are listed in Section 4.1. Each variable (plus body mass index instead of height and weight) will be summarised for each treatment group by the mean (SD) or median (IQR) for continuous variables, and the number (%) for categorical variables. Draft tables are given in APPENDIX D: DRAFT TABLES.

6.3. Analysis of feasibility outcomes

Duration of recruitment will be stated. It is the number of days from the day recruitment opens until the day the 90th patient is randomised (inclusive of both end days).

The number of participants randomised in each one month period from the day recruitment opens will be presented.

The estimated SD in each treatment group at each follow-up time point for pain acceptance (as measured by the Chronic Pain Acceptance Questionnaire (CPAQ-8)) will be presented.

Each patient adherence to app use outcome listed in Section 3.1 will be summarised separately for the intervention and active control treatment groups. Each outcome will be presented as the mean (SD) or median (IQR) for continuous variables, and the number (%) for categorical variables. Draft tables are given in APPENDIX D: DRAFT TABLES.

Forbes G, *et al. BMJ Open* 2020; 10:e030164. doi: 10.1136/bmjopen-2019-030164

6.4. Analysis of clinical outcomes

6.4.1. Descriptive statistics

For each clinical outcome listed in Section 3.3 we will present:

- The number of patients in each treatment group with a non-missing outcome at each time point.
- The mean (SD) in each treatment group at each time point.

6.4.2. Statistical analysis

For each clinical outcome we will present estimated treatment effects for each follow-up time point, with a 95% confidence interval. Estimates of treatment effects will be presented comparing the intervention group (mindfulness meditation app) to the control (treatment as usual) group, the intervention group to the active control (progressive muscle relaxation app) group, and the active control group to the control group.

Outcomes will be analysed using linear mixed-effects models with outcome measurement (at three follow-up time points) as the dependant variable. The model will include fixed time effects, a fixed effect for treatment, time treatment interactions for 3 months and 6 months follow-up time points, and an unstructured correlation matrix for the residuals [4]. The model will include baseline measure of the outcome as a covariate, assuming a linear relationship between baseline and outcome [5]. The model will be fitted using restricted maximum likelihood. Example Stata code for this analysis model is given in APPENDIX C: STATA CODE FOR STATISTICAL ANALYSIS OF CLINICAL OUTCOMES.

If there are missing values for baseline measure of a clinical outcome, they will be replaced by the mean of the observed baseline values for all participants in all treatment arms (mean imputation) [6]. Missing values of clinical outcomes at follow-up will not be imputed.

If the mixed effects models fail to converge, treatment effects will be estimated using separate linear regression models for each follow-up time point. Baseline measure of the outcome will be included as a covariate.

6.5. Other analyses

6.5.1. Comparison of losses to follow-up

The number and proportion of patients in each treatment group who have returned, answered by phone, or never returned the follow-up questionnaire will be presented for each follow-up time point (60 days, 3 months, and 6 months post-randomisation). A patient is counted as having returned data unless date of visit / date completed and all other fields in the follow-up questionnaire are missing. A draft table is given in APPENDIX D: DRAFT

Forbes G, et al. BMJ Open 2020; 10:e030164. doi: 10.1136/bmjopen-2019-030164

TABLES.Summaries of the following baseline variables will be presented separately for patients who have returned, answered by phone, or never returned the follow-up questionnaire at the 6 month time point:

- Age at randomisation
- Body mass index
- Living arrangements
- Employment status
- Age left full time education
- Ethnic group
- Do you smoke
- Number of cigarettes per week
- Do you drink alcohol
- Number of units of alcohol per week
- Length of pain
- Pain over the past week
- Baseline values of clinical outcomes:
    - Pain acceptance score
    - Depression score
    - Anxiety score
    - Pain related disability score

6.5.2. Unintentional unblinding of randomised treatment

For each participants in the intervention and active control arm, researcher response to the question "If the participant was allocated to the app treatment arm, which app treatment do you believe the participant was randomised to?" will be summarised by number and percentage.

For participants in the intervention and active control arms, response to the question "Do you think you received the new treatment or comparison treatment?" will be summarised by number and percentage. A draft table is given in APPENDIX D: DRAFT TABLES.

Forbes G, *et al. BMJ Open* 2020; 10:e030164. doi: 10.1136/bmjopen-2019-030164

6.5.3. Summarising missing data in clinical outcomes

For each clinical outcome variable we will present the number and proportion of individuals for whom the outcome is complete for at least one of the three follow-up time points (60 days, 3 months, or 6 months).

For each clinical outcome variable, we will also present the number and proportion of individuals for whom the outcome is not completed (either because the questionnaire was not returned, or because the participant left all variables for that outcome blank), partially completed (one or more, but not all, variables used in its derivation are missing), or complete (no variables used in its derivation are missing) at each time point.

Completely missing and partially missing outcomes will be summarised separately according to whether follow-up was completed via the mail-in questionnaire or over the phone.

6.5.1. Summarising data returned outside of target follow up periods

The number and proportion of patients in each treatment group who had follow up questionnaires completed within the time periods specified in section 4.3 will be presented for each follow up point. These are between 46 and 74 days for 60 days follow up, between 76 and 104 days for 3 month follow up, and between 159 and 201 days for 6 month follow up.

6.5.2. App usability

The mean (SD) of the System Usability Scale (SUS) score will be presented separately for the treatment app and active control app arms.

The number and proportion of each response for each question in the purpose made app satisfaction questionnaire will be presented separated for the treatment app and active control app arms. The number and proportion responding “Yes” to the question “Did you use the app every day?” will also be presented for each app arm.

6.5.3. Serious adverse events

We will present the number of reported serious adverse events in each treatment arm.

6.6. Analysis software

The analysis will be carried out using Stata.

Forbes G, *et al. BMJ Open* 2020; 10:e030164. doi: 10.1136/bmjopen-2019-030164

7. GRAPHS AND FIGURES TO BE PRODUCED

7.1. Participant flow

Participant throughput will be summarized in a CONSORT diagram:

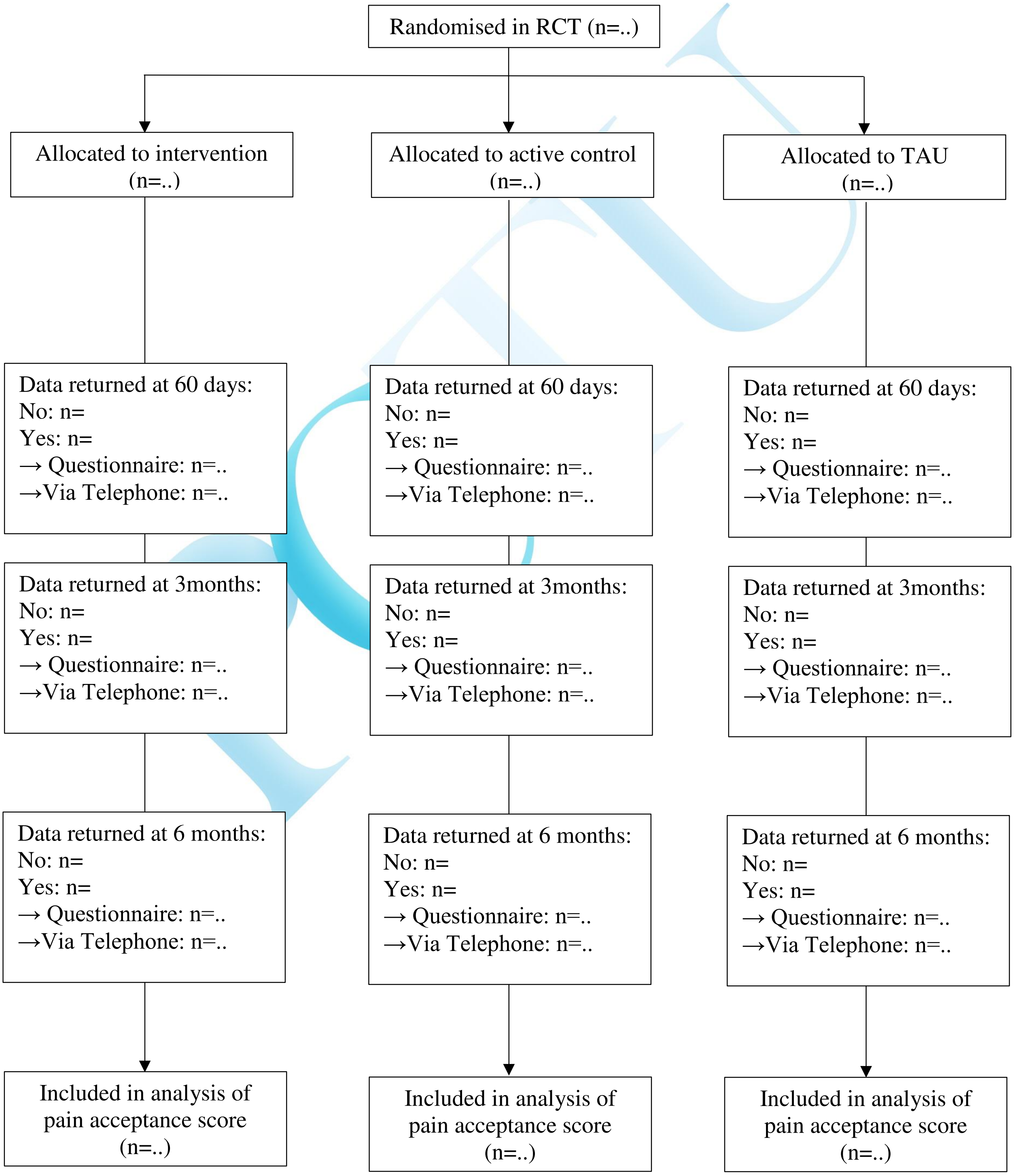

Forbes G, *et al. BMJ Open* 2020; 10:e030164. doi: 10.1136/bmjopen-2019-030164

7.2. Graphs

The following graphs will be created:

- Line graph showing mean CPAQ score at each time point for each treatment group. The graph will also include lines showing 95% confidence intervals for each mean CPAQ score.
- Line graph showing all estimated treatment effects (and 95% confidence intervals) on CPAQ score for each follow-up time point. (Estimates of treatment effects will be presented comparing the intervention group (mindfulness meditation app) to the control (treatment as usual) group, the intervention group to the active control (progressive muscle relaxation app) group, and the active control group to the control group.)
- Stacked bar chart showing the proportion of participants in each treatment group who have returned the follow-up questionnaire or answered the follow-up questionnaire by phone at each follow-up time point (60 days, 3 months, and 6 months post-randomisation).

Forbes G, et al. BMJ Open 2020; 10:e030164. doi: 10.1136/bmjopen-2019-030164

8. REFERENCES

[1] G. Lancaster, S. Dodd and P. Williamson, “Design and analysis of pilot studies: recommendations for good practice,” *Journal of Evaluation in Clinical Practice,* vol. 10, no. 2, pp. 307-12, 2004.

[2] M. Teare, M. Dimairo, N. Shephard, A. Hayman, A. Whitehead and S. Walters, “Sample size requirements to estimate key design parameters from external pilot randomised controlled trials: a simulation study,” *Trials,* vol. 15, p. 264, 2004.

[3] I. R. White, N. J. Horton, J. Carpenter and S. J. Pocock, “Strategy for intention to treat analysis in randomised trials with missing outcome data.,” *BMJ (Clinical research ed.),* vol. 342, p. d40, 2011.

[4] S. Rabe-Hesketh and A. Skrondal, Multilevel and Longitudinal Modeling Using Stata. Volume I: Continuous Responses, Stata Press, 2012.

[5] B. C. Kahan, V. Jairath, C. J. Doré and T. P. Morris, “The risks and rewards of covariate adjustment in randomized trials: an assessment of 12 outcomes from 8 studies,” *Trials,* vol. 15, p. 139, 2014.

[6] I. White and S. Thompson, “Adjusting for partially missing baseline measurements in randomized trials.,” *Statistics in Medicine,* vol. 24, no. 7, pp. 993-1007, 2005.

[7] “RAND 36-Item Short Form Survey (SF-36) Scoring Instructions,” [Online]. Available: https://www.rand.org/health/surveys_tools/mos/36-item-short-form/scoring.html.

[8] A. S. Zigmond and R. Snaith, “The hospital anxiety and depression scale.,” *Acta psychiatrica Scandinavica,* no. 6, pp. 361-370, 1983.

[9] G. Feldman, A. Hayes, S. Kumar, J. Greeson and J.-P. Laurenceau, “Mindfulness and Emotion Regulation: The Development and Initial Validation of the Cognitive and Affective Mindfulness Scale-Revised (CAMS-R),” *Journal of Psychopathology and Behavioral Assessment,* vol. 29, p. 177–190, 2007.

[10] M. V. Korff, J. Ormel, F. J. Keefe and S. F. Dworkin, “Grading the severity of chronic pain,” *Pain,* vol. 50, no. 2, pp. 133-149, 1992.

[11] M. K. Nicholas, “The pain self-efficacy questionnaire: Taking pain into account,” *European Journal of Pain,* vol. 11, p. 153–163, 2007.

[12] R. A. Fish, B. McGuire, M. Hogan, T. G. Morrison and I. Stewart, “Validation of the Chronic Pain Acceptance Questionnaire (CPAQ) in an Internet,” *Pain,* vol. 149, p. 435–443, 2010.

Forbes G, *et al. BMJ Open* 2020; 10:e030164. doi: 10.1136/bmjopen-2019-030164

[13] [Online]. Available: http://www.bris.ac.uk/primaryhealthcare/resources/mymop/general-information/.

[14] [Online]. Available: https://www.usability.gov/how-to-and-tools/methods/system-usability-scale.html.

Forbes G, *et al. BMJ Open* 2020; 10:e030164. doi: 10.1136/bmjopen-2019-030164

9. APPENDIX A: DERIVED AND COMPUTED VARIABLES

Unless otherwise stated, if an individual response variable used in the derivation of an outcome is missing then the outcome variable is missing.

Variables names used in the example code correspond to the field names specified in the trial database “Requirements Specification Document”.

Body mass index

BMI is calculated as a person’s weight (measured in kilograms) divided by the square of their height (measured in metres).

```
generate BMI = WEIGHT / ((HEIGHT / 100)^2)
```

RAND Short form (36) Health Survey (SF-36) scales scores [7]

Responses to individual questions are recoded as shown in the first table below. Each scale score is the average score for the questions in that scale, as shown in the second table below.

Item numbers	Original response code	Recode to
GH1, GH2, GH6, GH8, GH11b, GH11d	1	100
	2	75
	3	50
	4	25
	5	0
GH3a, GH3b, GH3c, GH3d, GH3e, GH3f, GH3g, GH3h, GH3i, GH3j	1	0
	2	50
	3	100
GH10, GH11a, GH11c	1	0
	2	25
	3	50
	4	75
	5	100
GH7	1	100
	2	80
	3	60
	4	40
	5	20
	6	0

Scale	After recoding, average the following items

Forbes G, *et al. BMJ Open* 2020; 10:e030164. doi: 10.1136/bmjopen-2019-030164

Physical functioning	GH3a, GH3b, GH3c, GH3d, GH3e, GH3f, GH3g, GH3h, GH3i, GH3j
Pain	GH7, GH8
General health	GH1, GH11a, GH11b, GH11c, GH11d
Social functioning	GH6, GH10

```
recode GH1 GH2 GH6 GH8 GH11b GH11d (1=100) (2=75) (3=50) (4=25) (5=0)
recode GH3a GH3b GH3c GH3d GH3e GH3f Gh3g GH3h GH3i Gh3j (1=0) (2=50) (3=100)
recode GH10 GH11a GH11c (1=0) (2=25) (3=50) (4=75) (5=100)
recode GH7 (1=100) (2=80) (3=60) (4=40) (5=20) (6=0)
generate SF36_PHYSICALFUNC = (GH3a + GH3b + GH3c + GH3d + GH3e + GH3f + GH3g + GH3h + GH3i + GH3j) / 10
generate SF36_SOCIALFUNC = (GH6 + GH10) / 2
generate SF36_PAIN = (GH7 + GH8) / 2
generate SF36_GENERALHEALTH = (GH1 + GH11a + GH11b + GH11c + GH11d) / 5
```

<u>Depression score (as measured by the Hospital Anxiety and Depression Scale (HADS))</u> [7]

After appropriate recoding, the HADS depression score is the sum of scores for questions 2, 4, 6, 8, 10, 12 and 14.

```
recode HADS02 HADS04 HADS12 HADS14 (1=0) (2=1) (3=2) (4=3)
recode HADS06 HADS08 HADS10 (1=3) (2=2) (3=1) (4=0)
generate HADS_DEPRESSION = HADS02 + HADS04 +HADS06 + HADS08 + HADS10 + HADS12 + HADS14
```

<u>Anxiety score (as measured by HADS)</u> [7]

After appropriate recoding, the HADS anxiety score is the sum of scores for questions 1, 3, 5, 7, 9, 11 and 13.

```
recode HADS01 HADS03 HADS05 HADS11 HADS13 (1=3) (2=2) (3=1) (4=0)
recode HADS07 HADS09 (1=0) (2=1) (3=2) (4=3)
generate HADS_ANXIETY = HADS01 + HADS03 + HADS05 + HADS07 + HADS09 + HADS11 + HADS13
```

<u>Mindfulness score (as measure by the Cognitive and Mindfulness - Revised (CAMS – R) scale)</u> [8]

Forbes G, *et al. BMJ Open* 2020; 10:e030164. doi: 10.1136/bmjopen-2019-030164

After appropriate recording, the CAMS-R mindfulness score is the sum of scores for all questions 1 to 12.

```
recode CAMSR02 CAMSR06 CAMSR07 (1=4) (2=3) (3=2) (4=1)
generate CAMSR_SCORE = CAMSR01 + CAMSR02 + CAMSR03 + CAMSR04 +
CAMSR05 + CAMSR06 + CAMSR07 + CAMSR08 + CAMSR09 + CAMSR10 +
CAMSR11 + CAMSR12
```

Pain related disability score (as measured by the Chronic Pain Grade (CPG) disability subscale) [9]

THE CPG pain related disability score is the mean of the daily activities, social activities, and work activities scores, multiplied by 10.

```
generate CPG_DISABILITYSCORE = [(CPGd1 + CPGd2 + CPGd3) / 3 ] *
10
```

Self efficacy score (as measured by the Pain Self-Efficacy Questionnaire (PSEQ)) [10]

The PSEQ self efficacy score is the sum of scores for all questions 1 to 10.

```
generate PSEQ_SCORE = PSEQ01 + PSEQ02 + PSEQ03 + PSEQ04 + PSEQ05
+ PSEQ06 + PSEQ07 + PSEQ08 + PSEQ09 + PSEQ10
```

Pain acceptance score (as measured Chronic Pain Acceptance Questionnaire (CPAQ-8)) [12]

After reverse scoring, the CPAQ-8 pain willingness score is the sum of scores from questions 4, 5, 7 and 8. The CPAQ-8 activity engagement score is the sum of scores from questions 1, 2, 3, 5 and 6. The CPAQ-8 total score is the sum of the pain willingness score and the activity engagement score.

```
recode CPAQ CPAQ4 CPAQ5 CPAQ7 CPAQ8 (0=6) (1=5) (2=4) (3=3)
(4=2) (5=1) (6=0)
generate CPAQ_PAINWILL = CPAQ4 + CPAQ5 + CPAQ7 + CPAQ8
generate CPAQ_ACTIVITYENG = CPAQ1 + CPAQ2 + CPAQ3 + CPAQ6
generate CPAQ_TOTAL = CPAQ_PAINWILL + CPAQ_ACTIVITYENG
```

Sexual Health Outcomes score (as measured by Sexual Health Outcomes in Women

Forbes G, *et al. BMJ Open* 2020; 10:e030164. doi: 10.1136/bmjopen-2019-030164

Questionnaire (SHOW-Q))

Each response is rescaled to a score 0 to 100, with higher scores reflecting higher sexual functioning or fewer sexual problems. For a 5 response item, the scores are 0, 25, 50, 75 or 100. For a 4 response item, the scores are 0, 33.3, 66.7 or 100. The scoring for each question is shown in the table below.

If a participant answers “I don’t have a partner” or “I don’t have sex without a partner” to question 2 or “I did not have sexual activity” to any of questions 3, 4, 6, 7 or 9, then the participant is classed as sexually inactive. Otherwise, the participant is classed as sexually active.

For sexually active participants, the SHOW-Q global score is calculated as the mean of all rescaled scores. Higher scores reflect higher sexual functioning or fewer sexual problems.

For all participants, the SHOW-Q pelvic problem interference score is the mean of response scores to questions 10, 11 and 12 after they are reverse scored. Higher scores reflect more interference.

Item number	Response text	Original response code	Recode to
SHOWQ01, SHOWQ02	Very satisfied	1	100
	Somewhat satisfied	2	75
	Neither satisfied nor dissatisfied	3	50
	Somewhat dissatisfied	4	25
	Very dissatisfied	5	0
SHOWQ10, SHOWQ11, SHOWQ12	Not at all	1	100
	Slightly	2	75
	Moderately	3	50
	Quite a bit	4	25
	Extremely	5	0
SHOWQ03, SHOWQ04	Never	1	0
	Rarely	2	25
	Sometimes	3	50
	Most of the time	4	75
	All of the time	5	100
SHOWQ08	Never	1	0
	Once or twice	2	25
	3-4 times	3	50
	5-6 times	4	75
	More than 6 times	5	100
SHOWQ05	Did not experience any orgasms	1	0
	Mild	2	33.3
	Moderate	3	66.7
	Strong	4	100
SHOWQ06,	Not a problem	1	100

Forbes G, *et al. BMJ Open* 2020; 10:e030164. doi: 10.1136/bmjopen-2019-030164

SHOWQ07, SHOWQ09	Little of a problem	2	66.7
	Somewhat of a problem	3	33.3
	Very much of a problem	4	0

```
generate SHOWQ_ACTIVE = 1
replace SHOWQ_ACTIVE = 0 if SHOWQ02==6 | SHOWQ02==7 | SHOWQ03==6
| SHOW04==6 | SHOWQ06==5 | SHOWQ07==5 | SHOWQ09== 5
recode SHOWQ01 SHOWQ02 SHOW10 SHOWQ11 SHOWQ12 (1=100) (2=75)
(3=50) (4=25) (5=0)
recode SHOWQ03 SHOWQ04 SHOWQ08 (1=0) (2=25) (3=50) (4=75)
(5=100)
recode SHOWQ05 (1=0) (2=33.3) (3=66.7) (4=100)
recode SHOWQ06 SHOWQ07 SHOWQ09 (1=100) (2=66.7) (3=33.3) (4=0)
generate SHOWQ_GLOBAL = (SHOWQ01 + SHOWQ02 + SHOWQ03 + SHOWQ04
+ SHOWQ05 + SHOWQ06 + SHOWQ07 + SHOWQ08 + SHOWQ09 + SHOWQ10 +
SHOWQ11 + SHOWQ12)/12 if SHOWQ_ACTIVE == 1
generate SHOWQ_PELVPROBLEM = ((100 - SHOWQ10) + (100 - SHOWQ11)
+ (100 - SHOWQ12))/3
```

Subjective outcome score (as measured by Measure Yourself Medical Outcome Profile (MYMOP)) [12]

If the description for symptom 1, symptom 2, symptom 3 or activity does not match the description given for the corresponding symptom or activity at baseline then the score for that symptom or activity is missing.

If symptom 1 score or wellbeing score are missing, then MYMOP profile score is missing.The MYMOP profile score is the mean of the symptom 1 score, symptom 2 score, activity score, wellbeing score, and symptom 3 score. (Symptom 2 score, activity score and symptom 3 score are only included if they are not missing)

```
egen MYMOP_PROFILE = rowmean(SYMSCORE1, SYMSCORE2, ACTSCORE,
WELLBEING, SYMSCORE3)
```

System Usability Scale (SUS) score [13]

Forbes G, et al. BMJ Open 2020; 10:e030164. doi: 10.1136/bmjopen-2019-030164

For questions 1, 3, 5, 7, and 9 the score contribution is the response number minus 1. For questions 2, 4, 6, 8, and 10 the score contribution is 5 minus the response number. The SUS score is the sum of all score contributions multiplied by 2.5

```
recode SUS01 SUS03 SYS05 SUS07 SUS09 (1 = 0) (2 = 1) (3 = 2) (4
= 3) (5 = 4)
recode SUS02 SUS04 SUS06 SUS08 SUS10 (1 = 4) (2 = 3) (3 = 2) (4
= 1) (5 = 0)
generate SUS_SCORE  = 2.5 * (SUS01 + SUS02 + SUS03 + SUS04 +
SUS05 +SUS06 + SUS07 + SUS08 + SUS9 + SUS10)
```

<u>Adherence outcomes</u>

countin60days	Number of days (within the first 60 days from randomisation) a patient has used the app (with app use defined as having completed at least 90% of a session).	
numberofweeksthreeplus	Number of weeks (within the first eight weeks from randomisation) a patient has used the app on three or more days.	
adhere_countin60days	Whether the patient has used the app on 22 or more days within the first 60 days from randomisation.	1 = Yes<br>0 = No
adhere_numberofweeksthreeplus	Whether the patient has used the app on three or more days in 6 or more weeks (within the first eight weeks from randomisation).	1 = Yes<br>0 = No

Sample Stata code showing the calculation of these outcome variables is given in APPENDIX B: STATA CODE FOR GENERATING ADHERENCE OUTCOMES.

Forbes G, *et al. BMJ Open* 2020; 10:e030164. doi: 10.1136/bmjopen-2019-030164

10. APPENDIX B: STATA CODE FOR GENERATING ADHERENCE OUTCOMES

Sample of Stata code for generating adherence outcomes from app usage data supplied by Headspace:

```
gen date_completed = date(datecompleted, "DMY")

format date_completed %td


gen date_rand = date(dateofrandomisation, "DMY")

format date_rand %td


gen date_fromrand = date_completed-date_rand


***


* Drop sessions which are not part of intervention (i.e. short duration)

drop if duration<5


* Remove multiple sessions in same day

duplicates report id date_fromrand
```


Forbes G, et al. BMJ Open 2020; 10:e030164. doi: 10.1136/bmjopen-2019-030164

```
duplicates drop id date_fromrand , force


gen in60days = 1 if date_fromrand<61

bysort id: egen countin60days = count(in60days)


gen numberofweeksthreeplus = 0


forvalues week=1/8 {

     gen inweek`week' = 1 if date_fromrand>7*(`week'-1) & date_fromrand<7*`week'+1

     gen threeplusinweek`week' = 0

     bysort id: egen countinweek`week' = count(inweek`week')

     assert countinweek`week'<8

     bysort id: replace threeplusinweek`week' = 1 if countinweek`week'>2

     bysort id: replace numberofweeksthreeplus = numberofweeksthreeplus +1 if countinweek`week'>2

}
```


Forbes G, *et al. BMJ Open* 2020; 10:e030164. doi: 10.1136/bmjopen-2019-030164

```
bysort id: keep if _n==1

keep id countin60days numberofweeksthreeplus threeplusinweek* countinweek*


gen adhere_countin60days = 0

replace adhere_countin60days = 1 if countin60days>21

gen adhere_numberofweeksthreeplus = 0

replace adhere_numberofweeksthreeplus = 1 if numberofweeksthreeplus>5


tab adhere_countin60days adhere_numberofweeksthreeplus
```


Forbes G, et al. BMJ Open 2020; 10:e030164. doi: 10.1136/bmjopen-2019-030164

11. APPENDIX C: STATA CODE FOR STATISTICAL ANALYSIS OF CLINICAL OUTCOMES

The following Stata shows the model that will be used to estimate treatment effects on clinical outcomes:

```
xtmixed outcome time##treat baseline || id: , noconstant
residuals(unstructured, t(time)) var reml
```

Estimates of treatment effects for each treatment arm comparison and time point will then be obtained using:

```
lincom 1.treat + 1.time#1.treat

lincom 1.treat + 2.time#1.treat

lincom 1.treat + 3.time#1.treat

lincom 2.treat + 1.time#2.treat

lincom 2.treat + 2.time#2.treat

lincom 2.treat + 3.time#2.treat

lincom 2.treat + 1.time#2.treat - 1.treat + 1.time#1.treat

lincom 2.treat + 2.time#2.treat - 1.treat + 2.time#1.treat

lincom 2.treat + 3.time#2.treat - 1.treat + 3.time#1.treat
```


Forbes G, *et al. BMJ Open* 2020; 10:e030164. doi: 10.1136/bmjopen-2019-030164

Barts and The London School of Medicine and Dentistry PCTU

12. APPENDIX D: DRAFT TABLES

12.1.1. Baseline demographics and medical history

Figures are mean (SD) unless stated otherwise.

	Intervention (n=…)	Active control (n=…)	Usual care (n=…)
**Demographics**			
Age at randomisation (Years)	XX (XX)	XX (XX)	XX (XX)
Body mass index	XX (XX)	XX (XX)	XX (XX)
Living arrangements – no. (%)			
Alone	XX (XX)	XX (XX)	XX (XX)
With others	XX (XX)	XX (XX)	XX (XX)
Employment status – no. (%)			
Employed	XX (XX)	XX (XX)	XX (XX)
Unemployed and looking for work	XX (XX)	XX (XX)	XX (XX)
At school or in full time education	XX (XX)	XX (XX)	XX (XX)
Unable to work due to long term sickness	XX (XX)	XX (XX)	XX (XX)
Look after your home/family	XX (XX)	XX (XX)	XX (XX)
Retired from paid work	XX (XX)	XX (XX)	XX (XX)
Other	XX (XX)	XX (XX)	XX (XX)
Age left full time education – no. (%)			
I did not receive a formal education	XX (XX)	XX (XX)	XX (XX)
Age 12 or less	XX (XX)	XX (XX)	XX (XX)
Age 13 to 16	XX (XX)	XX (XX)	XX (XX)
Age 17 to 19	XX (XX)	XX (XX)	XX (XX)
Age 20 or over	XX (XX)	XX (XX)	XX (XX)
I am still in full time education	XX (XX)	XX (XX)	XX (XX)
Other	XX (XX)	XX (XX)	XX (XX)
Ethnic group – no. (%)			
White	XX (XX)	XX (XX)	XX (XX)
Black	XX (XX)	XX (XX)	XX (XX)
Central Asian	XX (XX)	XX (XX)	XX (XX)
Middle Eastern	XX (XX)	XX (XX)	XX (XX)
Southern Asian	XX (XX)	XX (XX)	XX (XX)
Mixed	XX (XX)	XX (XX)	XX (XX)
Other ethnic group	XX (XX)	XX (XX)	XX (XX)
Do not wish to say	XX (XX)	XX (XX)	XX (XX)
Smoker – no. (%)			
Yes	XX (XX)	XX (XX)	XX (XX)
No	XX (XX)	XX (XX)	XX (XX)
If yes, number of cigarettes per week	XX (XX)	XX (XX)	XX (XX)
Drink alcohol – no. (%)			

Forbes G, *et al. BMJ Open* 2020; 10:e030164. doi: 10.1136/bmjopen-2019-030164

Yes	XX (XX)	XX (XX)	XX (XX)
No	XX (XX)	XX (XX)	XX (XX)
If yes, number of units of alcohol per week	XX (XX)	XX (XX)	XX (XX)
**Baseline medical history**			
Length of pain – no. (%)			
0-6 months	XX (XX)	XX (XX)	XX (XX)
7-12 months	XX (XX)	XX (XX)	XX (XX)
1-2 years	XX (XX)	XX (XX)	XX (XX)
3-5 years	XX (XX)	XX (XX)	XX (XX)
6-10 years	XX (XX)	XX (XX)	XX (XX)
More than 10 years	XX (XX)	XX (XX)	XX (XX)
Pain over the past week	XX (XX)	XX (XX)	XX (XX)

12.1.2. Prior and concurrent treatment

Figures are number (percentage).

	**Intervention (n=…)**	**Active control (n=…)**	**Usual care (n=…)**
Treatment used in last six months			
Acupuncture	XX (XX)	XX (XX)	XX (XX)
Gabapentin	XX (XX)	XX (XX)	XX (XX)
Amitriptyline	XX (XX)	XX (XX)	XX (XX)
Biofeedback	XX (XX)	XX (XX)	XX (XX)
Botox injection	XX (XX)	XX (XX)	XX (XX)
Contraceptive pills/patch/ring	XX (XX)	XX (XX)	XX (XX)
Exercise, yoga or pilates	XX (XX)	XX (XX)	XX (XX)
Injections to suppress ovaries (e.g. Prostap, Zoladex)	XX (XX)	XX (XX)	XX (XX)
Herbal Medicine	XX (XX)	XX (XX)	XX (XX)
Meditation or relaxation exercises	XX (XX)	XX (XX)	XX (XX)
Massage	XX (XX)	XX (XX)	XX (XX)
Nutrition/diet	XX (XX)	XX (XX)	XX (XX)
Codeine or Morphine type painkillers	XX (XX)	XX (XX)	XX (XX)
Nerve blocks	XX (XX)	XX (XX)	XX (XX)
Over the counter medication	XX (XX)	XX (XX)	XX (XX)
Physiotherapy	XX (XX)	XX (XX)	XX (XX)
Psychological (talking) therapy	XX (XX)	XX (XX)	XX (XX)
Transcutaneous Electrical Nerve Stimulation (TENS)	XX (XX)	XX (XX)	XX (XX)
Surgery	XX (XX)	XX (XX)	XX (XX)

Forbes G, *et al. BMJ Open* 2020; 10:e030164. doi: 10.1136/bmjopen-2019-030164

	Intervention	Active control	Usual care
Other	XX (XX)	XX (XX)	XX (XX)
Currently using pain treatment			
Yes	XX (XX)	XX (XX)	XX (XX)
No	XX (XX)	XX (XX)	XX (XX)

12.1.3. Baseline values of clinical outcomes

Figures are mean (SD) unless stated otherwise.

	**Intervention (n=…)**	**Active control (n=…)**	**Usual care (n=…)**
SF-36 scales:			
Physical functioning	XX (XX)	XX (XX)	XX (XX)
Pain	XX (XX)	XX (XX)	XX (XX)
General Health	XX (XX)	XX (XX)	XX (XX)
Social Functioning	XX (XX)	XX (XX)	XX (XX)
Depression score	XX (XX)	XX (XX)	XX (XX)
Anxiety score	XX (XX)	XX (XX)	XX (XX)
Mindfulness score	XX (XX)	XX (XX)	XX (XX)
Pain related disability score	XX (XX)	XX (XX)	XX (XX)
Self efficacy score	XX (XX)	XX (XX)	XX (XX)
Pain acceptance score	XX (XX)	XX (XX)	XX (XX)
Sexual health outcomes:			
SHOW-Q global score	XX (XX)	XX (XX)	XX (XX)
SHOW-Q pelvic problem interference score	XX (XX)	XX (XX)	XX (XX)
Subjective outcome score	XX (XX)	XX (XX)	XX (XX)

12.1.4. Loss to follow-up

	**Intervention (n=…)**	**Active control (n=…)**	**Usual care (n=…)**
Follow-up questionnaire returned – no. (%)			
60 days	XX (XX)	XX (XX)	XX (XX)
3 months	XX (XX)	XX (XX)	XX (XX)
6 months	XX (XX)	XX (XX)	XX (XX)
Follow-up questionnaire answered by phone – no. (%)			
60 days	XX (XX)	XX (XX)	XX (XX)
3 months	XX (XX)	XX (XX)	XX (XX)
6 months	XX (XX)	XX (XX)	XX (XX)
Follow-up questionnaire never returned – no. (%)			
60 days	XX (XX)	XX (XX)	XX (XX)
3 months	XX (XX)	XX (XX)	XX (XX)
6 months	XX (XX)	XX (XX)	XX (XX)

Forbes G, *et al. BMJ Open* 2020; 10:e030164. doi: 10.1136/bmjopen-2019-030164

12.1.5. Loss to follow-up

Figures are mean (SD) unless stated otherwise.

	6 months follow-up questionnaire returned (n=…)	6 months follow-up questionnaire answered by phone (n=…)	6 months follow-up questionnaire never returned (n=…)
**Demographics**			
Age at randomisation (Years)	XX (XX)	XX (XX)	XX (XX)
Body mass index	XX (XX)	XX (XX)	XX (XX)
Living arrangements – no. (%)			
Alone	XX (XX)	XX (XX)	XX (XX)
With others	XX (XX)	XX (XX)	XX (XX)
Employment status – no. (%)			
Employed	XX (XX)	XX (XX)	XX (XX)
Unemployed and looking for work	XX (XX)	XX (XX)	XX (XX)
At school or in full time education	XX (XX)	XX (XX)	XX (XX)
Unable to work due to long term sickness	XX (XX)	XX (XX)	XX (XX)
Look after your home/family	XX (XX)	XX (XX)	XX (XX)
Retired from paid work	XX (XX)	XX (XX)	XX (XX)
Other	XX (XX)	XX (XX)	XX (XX)
Age left full time education – no. (%)			
I did not receive a formal education	XX (XX)	XX (XX)	XX (XX)
Age 12 or less	XX (XX)	XX (XX)	XX (XX)
Age 13 to 16	XX (XX)	XX (XX)	XX (XX)
Age 17 to 19	XX (XX)	XX (XX)	XX (XX)
Age 20 or over	XX (XX)	XX (XX)	XX (XX)
I am still in full time education	XX (XX)	XX (XX)	XX (XX)
Other	XX (XX)	XX (XX)	XX (XX)
Ethnic group – no. (%)			
White	XX (XX)	XX (XX)	XX (XX)
Black	XX (XX)	XX (XX)	XX (XX)
Central Asian	XX (XX)	XX (XX)	XX (XX)
Middle Eastern	XX (XX)	XX (XX)	XX (XX)
Southern Asian	XX (XX)	XX (XX)	XX (XX)
Mixed	XX (XX)	XX (XX)	XX (XX)
Other ethnic group	XX (XX)	XX (XX)	XX (XX)
Do not wish to say	XX (XX)	XX (XX)	XX (XX)
Smoker – no. (%)			

Forbes G, *et al. BMJ Open* 2020; 10:e030164. doi: 10.1136/bmjopen-2019-030164

Barts and The London School of Medicine and Dentistry PCTU

Yes	XX	(XX)	XX	(XX)	XX	(XX)
No	XX	(XX)	XX	(XX)	XX	(XX)
If yes, number of cigarettes per week	XX	(XX)	XX	(XX)	XX	(XX)
Drink alcohol – no. (%)						
Yes	XX	(XX)	XX	(XX)	XX	(XX)
No	XX	(XX)	XX	(XX)	XX	(XX)
If yes, number of units of alcohol per week	XX	(XX)	XX	(XX)	XX	(XX)
**Baseline medical history**						
Length of pain – no. (%)						
0-6 months	XX	(XX)	XX	(XX)	XX	(XX)
7-12 months	XX	(XX)	XX	(XX)	XX	(XX)
1-2 years	XX	(XX)	XX	(XX)	XX	(XX)
3-5 years	XX	(XX)	XX	(XX)	XX	(XX)
6-10 years	XX	(XX)	XX	(XX)	XX	(XX)
More than 10 years	XX	(XX)	XX	(XX)	XX	(XX)
Pain over the past week	XX	(XX)	XX	(XX)	XX	(XX)
**Baseline values of clinical outcomes**						
Pain acceptance score (CPAQ-8)	XX	(XX)	XX	(XX)	XX	(XX)
Depression score (HADS)	XX	(XX)	XX	(XX)	XX	(XX)
Anxiety score (HADS)	XX	(XX)	XX	(XX)	XX	(XX)
Pain related disability score (CPG)	XX	(XX)	XX	(XX)	XX	(XX)

12.1.6. Follow up within target follow up period

	Intervention (n=…)	Active control (n=…)	Usual care (n=…)
Follow-up questionnaire returned or answered by phone within target follow up period– no. (%)			
60 days (46 and 74days)	XX (XX)	XX (XX)	XX (XX)
3 months (76 and 104 days)	XX (XX)	XX (XX)	XX (XX)
6 months (159 and 201 days)	XX (XX)	XX (XX)	XX (XX)

12.1.7. Adherence to app use

Figures are mean (SD) unless stated otherwise.

	Intervention (n=…)	Active control (n=…)
Number of days (within the first 60 days from randomisation) a patient has used the app	XX (XX)	XX (XX)

Forbes G, *et al. BMJ Open* 2020; 10:e030164. doi: 10.1136/bmjopen-2019-030164

Number of weeks (within the first eight weeks from randomisation) a patient has used the app on three or more days	XX (XX)	XX (XX)
Used the app on 22 or more days within the first 60 days from randomisation – no. (%)	XX (XX)	XX (XX)
Used the app on three or more days in 6 or more weeks (within the first eight weeks from randomisation) – no. (%)	XX (XX)	XX (XX)
Used the app on 22 or more days within the first 60 days from randomisation, AND used the app on three or more days in 6 or more weeks within the first eight weeks from randomisation – no. (%)	XX (XX)	XX (XX)

12.1.8. App usability questionnaire

Figures are number (percentage).

	Totally disagree	Somewhat disagree	Neither agree nor disagree	Somewhat agree	Totally agree	Not answered
It is easy to access the app whenever I wanted to use it						
Intervention:	XX (XX)	XX (XX)	XX (XX)	XX (XX)	XX (XX)	XX (XX)
Active control:	XX (XX)	XX (XX)	XX (XX)	XX (XX)	XX (XX)	XX (XX)
After being shown, I understood how the app would work						
Intervention:	XX (XX)	XX (XX)	XX (XX)	XX (XX)	XX (XX)	XX (XX)
Active control:	XX (XX)	XX (XX)	XX (XX)	XX (XX)	XX (XX)	XX (XX)
It was fun to work with the app						
Intervention:	XX (XX)	XX (XX)	XX (XX)	XX (XX)	XX (XX)	XX (XX)
Active control:	XX (XX)	XX (XX)	XX (XX)	XX (XX)	XX (XX)	XX (XX)
The app worked well						
Intervention:	XX (XX)	XX (XX)	XX (XX)	XX (XX)	XX (XX)	XX (XX)
Active control:	XX (XX)	XX (XX)	XX (XX)	XX (XX)	XX (XX)	XX (XX)
It was easy to work through the modules						
Intervention:	XX (XX)	XX (XX)	XX (XX)	XX (XX)	XX (XX)	XX (XX)
Active control:	XX (XX)	XX (XX)	XX (XX)	XX (XX)	XX (XX)	XX (XX)

Forbes G, *et al. BMJ Open* 2020; 10:e030164. doi: 10.1136/bmjopen-2019-030164

The number of modules was annoying						
Intervention:	XX (XX)	XX (XX)	XX (XX)	XX (XX)	XX (XX)	XX (XX)
Active control:	XX (XX)	XX (XX)	XX (XX)	XX (XX)	XX (XX)	XX (XX)
The modules were well-displayed on my smartphone						
Intervention:	XX (XX)	XX (XX)	XX (XX)	XX (XX)	XX (XX)	XX (XX)
Active control:	XX (XX)	XX (XX)	XX (XX)	XX (XX)	XX (XX)	XX (XX)
Using the app was difficult because of my daily activities						
Intervention:	XX (XX)	XX (XX)	XX (XX)	XX (XX)	XX (XX)	XX (XX)
Active control:	XX (XX)	XX (XX)	XX (XX)	XX (XX)	XX (XX)	XX (XX)
Using the app took too long						
Intervention:	XX (XX)	XX (XX)	XX (XX)	XX (XX)	XX (XX)	XX (XX)
Active control:	XX (XX)	XX (XX)	XX (XX)	XX (XX)	XX (XX)	XX (XX)

Forbes G, *et al. BMJ Open* 2020; 10:e030164. doi: 10.1136/bmjopen-2019-030164

Barts and The London School of Medicine and Dentistry PCTU

12.1.9. Clinical outcomes

	Intervention (n=…)				Active control (n=…)				Usual care (n=…)			
	**n**	**(%)**	**Mean**	**(SD)**	**n**	**(%)**	**Mean**	**(SD)**	**n**	**(%)**	**Mean**	**(SD)**
**Pain acceptance score**												
Baseline	XX	(XX)	XX	(XX)	XX	(XX)	XX	(XX)	XX	(XX)	XX	(XX)
60 days	XX	(XX)	XX	(XX)	XX	(XX)	XX	(XX)	XX	(XX)	XX	(XX)
3 months	XX	(XX)	XX	(XX)	XX	(XX)	XX	(XX)	XX	(XX)	XX	(XX)
6 months	XX	(XX)	XX	(XX)	XX	(XX)	XX	(XX)	XX	(XX)	XX	(XX)
Included in analysis †	XX	(XX)			XX	(XX)			XX	(XX)		
**Depression score**												
Baseline	XX	(XX)	XX	(XX)	XX	(XX)	XX	(XX)	XX	(XX)	XX	(XX)
60 days	XX	(XX)	XX	(XX)	XX	(XX)	XX	(XX)	XX	(XX)	XX	(XX)
3 months	XX	(XX)	XX	(XX)	XX	(XX)	XX	(XX)	XX	(XX)	XX	(XX)
6 months	XX	(XX)	XX	(XX)	XX	(XX)	XX	(XX)	XX	(XX)	XX	(XX)
Included in analysis †	XX	(XX)			XX	(XX)			XX	(XX)		
**Anxiety score**												
Baseline	XX	(XX)	XX	(XX)	XX	(XX)	XX	(XX)	XX	(XX)	XX	(XX)
60 days	XX	(XX)	XX	(XX)	XX	(XX)	XX	(XX)	XX	(XX)	XX	(XX)
3 months	XX	(XX)	XX	(XX)	XX	(XX)	XX	(XX)	XX	(XX)	XX	(XX)
6 months	XX	(XX)	XX	(XX)	XX	(XX)	XX	(XX)	XX	(XX)	XX	(XX)
Included in analysis †	XX	(XX)			XX	(XX)			XX	(XX)		

Forbes G, *et al. BMJ Open* 2020; 10:e030164. doi: 10.1136/bmjopen-2019-030164

**Mindfulness score**						
Baseline	XX (XX)	XX (XX)	XX (XX)	XX (XX)	XX (XX)	XX (XX)
60 days	XX (XX)	XX (XX)	XX (XX)	XX (XX)	XX (XX)	XX (XX)
3 months	XX (XX)	XX (XX)	XX (XX)	XX (XX)	XX (XX)	XX (XX)
6 months	XX (XX)	XX (XX)	XX (XX)	XX (XX)	XX (XX)	XX (XX)
Included in analysis †	XX (XX)		XX (XX)		XX (XX)	
**Pain related disability score**						
Baseline	XX (XX)	XX (XX)	XX (XX)	XX (XX)	XX (XX)	XX (XX)
60 days	XX (XX)	XX (XX)	XX (XX)	XX (XX)	XX (XX)	XX (XX)
3 months	XX (XX)	XX (XX)	XX (XX)	XX (XX)	XX (XX)	XX (XX)
6 months	XX (XX)	XX (XX)	XX (XX)	XX (XX)	XX (XX)	XX (XX)
Included in analysis †	XX (XX)		XX (XX)		XX (XX)	
**Self efficacy score**						
Baseline	XX (XX)	XX (XX)	XX (XX)	XX (XX)	XX (XX)	XX (XX)
60 days	XX (XX)	XX (XX)	XX (XX)	XX (XX)	XX (XX)	XX (XX)
3 months	XX (XX)	XX (XX)	XX (XX)	XX (XX)	XX (XX)	XX (XX)
6 months	XX (XX)	XX (XX)	XX (XX)	XX (XX)	XX (XX)	XX (XX)
Included in analysis †	XX (XX)		XX (XX)		XX (XX)	
**SHOW-Q global score, for sexually active participants**						
Baseline	XX (XX)	XX (XX)	XX (XX)	XX (XX)	XX (XX)	XX (XX)
60 days	XX (XX)	XX (XX)	XX (XX)	XX (XX)	XX (XX)	XX (XX)
3 months	XX (XX)	XX (XX)	XX (XX)	XX (XX)	XX (XX)	XX (XX)

6 months	XX	(XX)	XX	(XX)	XX	(XX)	XX	(XX)	XX	(XX)	XX	(XX)
Included in analysis †	XX	(XX)			XX	(XX)			XX	(XX)		
**SHOW-Q pelvic problem interference score, for all participants**												
Baseline	XX	(XX)	XX	(XX)	XX	(XX)	XX	(XX)	XX	(XX)	XX	(XX)
60 days	XX	(XX)	XX	(XX)	XX	(XX)	XX	(XX)	XX	(XX)	XX	(XX)
3 months	XX	(XX)	XX	(XX)	XX	(XX)	XX	(XX)	XX	(XX)	XX	(XX)
6 months	XX	(XX)	XX	(XX)	XX	(XX)	XX	(XX)	XX	(XX)	XX	(XX)
Included in analysis †	XX	(XX)			XX	(XX)			XX	(XX)		
**Subjective outcome score**												
Baseline	XX	(XX)	XX	(XX)	XX	(XX)	XX	(XX)	XX	(XX)	XX	(XX)
60 days	XX	(XX)	XX	(XX)	XX	(XX)	XX	(XX)	XX	(XX)	XX	(XX)
3 months	XX	(XX)	XX	(XX)	XX	(XX)	XX	(XX)	XX	(XX)	XX	(XX)
6 months	XX	(XX)	XX	(XX)	XX	(XX)	XX	(XX)	XX	(XX)	XX	(XX)
Included in analysis †	XX	(XX)			XX	(XX)			XX	(XX)		
**SF-36: Physical functioning**												
Baseline	XX	(XX)	XX	(XX)	XX	(XX)	XX	(XX)	XX	(XX)	XX	(XX)
60 days	XX	(XX)	XX	(XX)	XX	(XX)	XX	(XX)	XX	(XX)	XX	(XX)
3 months	XX	(XX)	XX	(XX)	XX	(XX)	XX	(XX)	XX	(XX)	XX	(XX)
6 months	XX	(XX)	XX	(XX)	XX	(XX)	XX	(XX)	XX	(XX)	XX	(XX)
Included in analysis †	XX	(XX)			XX	(XX)			XX	(XX)		
**SF-36: Pain**												
Baseline	XX	(XX)	XX	(XX)	XX	(XX)	XX	(XX)	XX	(XX)	XX	(XX)

Forbes G, *et al. BMJ Open* 2020; 10:e030164. doi: 10.1136/bmjopen-2019-030164

60 days	XX	(XX)	XX	(XX)	XX	(XX)	XX	(XX)	XX	(XX)	XX	(XX)
3 months	XX	(XX)	XX	(XX)	XX	(XX)	XX	(XX)	XX	(XX)	XX	(XX)
6 months	XX	(XX)	XX	(XX)	XX	(XX)	XX	(XX)	XX	(XX)	XX	(XX)
Included in analysis †	XX	(XX)			XX	(XX)			XX	(XX)		
**SF-36: General Health**												
Baseline	XX	(XX)	XX	(XX)	XX	(XX)	XX	(XX)	XX	(XX)	XX	(XX)
60 days	XX	(XX)	XX	(XX)	XX	(XX)	XX	(XX)	XX	(XX)	XX	(XX)
3 months	XX	(XX)	XX	(XX)	XX	(XX)	XX	(XX)	XX	(XX)	XX	(XX)
6 months	XX	(XX)	XX	(XX)	XX	(XX)	XX	(XX)	XX	(XX)	XX	(XX)
Included in analysis †	XX	(XX)			XX	(XX)			XX	(XX)		
**SF-36: Social Functioning**												
Baseline	XX	(XX)	XX	(XX)	XX	(XX)	XX	(XX)	XX	(XX)	XX	(XX)
60 days	XX	(XX)	XX	(XX)	XX	(XX)	XX	(XX)	XX	(XX)	XX	(XX)
3 months	XX	(XX)	XX	(XX)	XX	(XX)	XX	(XX)	XX	(XX)	XX	(XX)
6 months	XX	(XX)	XX	(XX)	XX	(XX)	XX	(XX)	XX	(XX)	XX	(XX)
Included in analysis †	XX	(XX)			XX	(XX)			XX	(XX)		

(† Included in analysis if outcome is available for at least one follow-up time point.)

Forbes G, *et al. BMJ Open* 2020; 10:e030164. doi: 10.1136/bmjopen-2019-030164

	Intervention vs. Active control Adjusted mean difference (95% CI)	Invention vs. Usual care Adjusted mean difference (95% CI)	Active control vs. Usual care Adjusted mean difference (95% CI)
**Pain acceptance score**			
60 days	XX (XX to XX)	XX (XX to XX)	XX (XX to XX)
3 months	XX (XX to XX)	XX (XX to XX)	XX (XX to XX)
6 months	XX (XX to XX)	XX (XX to XX)	XX (XX to XX)
**Depression score**			
60 days	XX (XX to XX)	XX (XX to XX)	XX (XX to XX)
3 months	XX (XX to XX)	XX (XX to XX)	XX (XX to XX)
6 months	XX (XX to XX)	XX (XX to XX)	XX (XX to XX)
**Anxiety score**			
60 days	XX (XX to XX)	XX (XX to XX)	XX (XX to XX)
3 months	XX (XX to XX)	XX (XX to XX)	XX (XX to XX)
6 months	XX (XX to XX)	XX (XX to XX)	XX (XX to XX)
**Mindfulness score**			
60 days	XX (XX to XX)	XX (XX to XX)	XX (XX to XX)
3 months	XX (XX to XX)	XX (XX to XX)	XX (XX to XX)
6 months	XX (XX to XX)	XX (XX to XX)	XX (XX to XX)
**Pain related disability score**			
60 days	XX (XX to XX)	XX (XX to XX)	XX (XX to XX)
3 months	XX (XX to XX)	XX (XX to XX)	XX (XX to XX)
6 months	XX (XX to XX)	XX (XX to XX)	XX (XX to XX)
**Self efficacy score**			
60 days	XX (XX to XX)	XX (XX to XX)	XX (XX to XX)
3 months	XX (XX to XX)	XX (XX to XX)	XX (XX to XX)
6 months	XX (XX to XX)	XX (XX to XX)	XX (XX to XX)
**SHOW-Q global score, for sexually active participants**			
60 days	XX (XX to XX)	XX (XX to XX)	XX (XX to XX)
3 months	XX (XX to XX)	XX (XX to XX)	XX (XX to XX)
6 months	XX (XX to XX)	XX (XX to XX)	XX (XX to XX)
**SHOW-Q pelvic problem interference score, for all participants**			
60 days	XX (XX to XX)	XX (XX to XX)	XX (XX to XX)
3 months	XX (XX to XX)	XX (XX to XX)	XX (XX to XX)

Forbes G, *et al. BMJ Open* 2020; 10:e030164. doi: 10.1136/bmjopen-2019-030164

6 months	XX	(XX to XX)	XX	(XX to XX)	XX	(XX to XX)
**Subjective outcome score**						
60 days	XX	(XX to XX)	XX	(XX to XX)	XX	(XX to XX)
3 months	XX	(XX to XX)	XX	(XX to XX)	XX	(XX to XX)
6 months	XX	(XX to XX)	XX	(XX to XX)	XX	(XX to XX)
**SF-36: Physical Functioning**						
60 days	XX	(XX to XX)	XX	(XX to XX)	XX	(XX to XX)
3 months	XX	(XX to XX)	XX	(XX to XX)	XX	(XX to XX)
6 months	XX	(XX to XX)	XX	(XX to XX)	XX	(XX to XX)
**SF-36: Pain**						
60 days	XX	(XX to XX)	XX	(XX to XX)	XX	(XX to XX)
3 months	XX	(XX to XX)	XX	(XX to XX)	XX	(XX to XX)
6 months	XX	(XX to XX)	XX	(XX to XX)	XX	(XX to XX)
**SF-36: General Health**						
60 days	XX	(XX to XX)	XX	(XX to XX)	XX	(XX to XX)
3 months	XX	(XX to XX)	XX	(XX to XX)	XX	(XX to XX)
6 months	XX	(XX to XX)	XX	(XX to XX)	XX	(XX to XX)
**SF-36: Social Functioning**						
60 days	XX	(XX to XX)	XX	(XX to XX)	XX	(XX to XX)
3 months	XX	(XX to XX)	XX	(XX to XX)	XX	(XX to XX)
6 months	XX	(XX to XX)	XX	(XX to XX)	XX	(XX to XX)

12.1.10. Unintentional unblinding of randomised treatment

Figures are number (%) unless stated otherwise.

	Intervention (n=…)		Active control (n=…)	
Researchers: Which app treatment do you believe the participant was randomised to?				
Intervention app	XX	(XX)	XX	(XX)
Control app	XX	(XX)	XX	(XX)
Don’t know	XX	(XX)	XX	(XX)
Participants: Do you think you received the new treatment or comparison treatment?				
New treatment	XX	(XX)	XX	(XX)
Comparison treatment	XX	(XX)	XX	(XX)
Don’t know	XX	(XX)	XX	(XX)

Forbes G, *et al. BMJ Open* 2020; 10:e030164. doi: 10.1136/bmjopen-2019-030164

12.1.11. Partially missing clinical outcomes

	Not completed *		Partially Completed **		Fully completed ***	
	n	(%)	n	(%)	n	(%)
**Pain acceptance score**						
Baseline	XX	(XX)	XX	(XX)	XX	(XX)
60 days	XX	(XX)	XX	(XX)	XX	(XX)
3 months	XX	(XX)	XX	(XX)	XX	(XX)
6 months	XX	(XX)	XX	(XX)	XX	(XX)
**Depression score**						
Baseline	XX	(XX)	XX	(XX)	XX	(XX)
60 days	XX	(XX)	XX	(XX)	XX	(XX)
3 months	XX	(XX)	XX	(XX)	XX	(XX)
6 months	XX	(XX)	XX	(XX)	XX	(XX)
**Anxiety score**						
Baseline	XX	(XX)	XX	(XX)	XX	(XX)
60 days	XX	(XX)	XX	(XX)	XX	(XX)
3 months	XX	(XX)	XX	(XX)	XX	(XX)
6 months	XX	(XX)	XX	(XX)	XX	(XX)
**Mindfulness score**						
Baseline	XX	(XX)	XX	(XX)	XX	(XX)
60 days	XX	(XX)	XX	(XX)	XX	(XX)
3 months	XX	(XX)	XX	(XX)	XX	(XX)
6 months	XX	(XX)	XX	(XX)	XX	(XX)
**Pain related disability score**						
Baseline	XX	(XX)	XX	(XX)	XX	(XX)
60 days	XX	(XX)	XX	(XX)	XX	(XX)
3 months	XX	(XX)	XX	(XX)	XX	(XX)
6 months	XX	(XX)	XX	(XX)	XX	(XX)
**Self efficacy score**						
Baseline	XX	(XX)	XX	(XX)	XX	(XX)
60 days	XX	(XX)	XX	(XX)	XX	(XX)
3 months	XX	(XX)	XX	(XX)	XX	(XX)
6 months	XX	(XX)	XX	(XX)	XX	(XX)

Forbes G, *et al. BMJ Open* 2020; 10:e030164. doi: 10.1136/bmjopen-2019-030164

**SHOW-Q global score, for sexually active participants**			
Baseline	XX (XX)	XX (XX)	XX (XX)
60 days	XX (XX)	XX (XX)	XX (XX)
3 months	XX (XX)	XX (XX)	XX (XX)
6 months	XX (XX)	XX (XX)	XX (XX)
**SHOW-Q pelvic problem interference score, for all participants**			
Baseline	XX (XX)	XX (XX)	XX (XX)
60 days	XX (XX)	XX (XX)	XX (XX)
3 months	XX (XX)	XX (XX)	XX (XX)
6 months	XX (XX)	XX (XX)	XX (XX)
**Subjective outcome score**			
Baseline	XX (XX)	XX (XX)	XX (XX)
60 days	XX (XX)	XX (XX)	XX (XX)
3 months	XX (XX)	XX (XX)	XX (XX)
6 months	XX (XX)	XX (XX)	XX (XX)
**SF-36: Physical Functioning**			
Baseline	XX (XX)	XX (XX)	XX (XX)
60 days	XX (XX)	XX (XX)	XX (XX)
3 months	XX (XX)	XX (XX)	XX (XX)
6 months	XX (XX)	XX (XX)	XX (XX)
**SF-36: Pain**			
Baseline	XX (XX)	XX (XX)	XX (XX)
60 days	XX (XX)	XX (XX)	XX (XX)
3 months	XX (XX)	XX (XX)	XX (XX)
6 months	XX (XX)	XX (XX)	XX (XX)
**SF-36: General Health**			
Baseline	XX (XX)	XX (XX)	XX (XX)
60 days	XX (XX)	XX (XX)	XX (XX)
3 months	XX (XX)	XX (XX)	XX (XX)
6 months	XX (XX)	XX (XX)	XX (XX)
**SF-36: Social Functioning**			
Baseline	XX (XX)	XX (XX)	XX (XX)
60 days	XX (XX)	XX (XX)	XX (XX)
3 months	XX (XX)	XX (XX)	XX (XX)
6 months	XX (XX)	XX (XX)	XX (XX)

Forbes G, *et al. BMJ Open* 2020; 10:e030164. doi: 10.1136/bmjopen-2019-030164

* Questionnaire not answered or all variables used in the derivation of the outcome are missing.
** One or more, but not all, variables used in the derivation of the outcome are missing.
*** No variables used in the derivation of the outcome are missing.

Forbes G, *et al. BMJ Open* 2020; 10:e030164. doi: 10.1136/bmjopen-2019-030164

	Questionnaire never returned	Questionnaire answered by telephone			Questionnaire returned		
		Not completed †	Partially completed ††	Fully completed †††	Not completed †	Partially completed ††	Fully completed †††
	n (%)	n (%)	n (%)	n (%)	n (%)	n (%)	n (%)
**Pain acceptance score**							
Baseline	XX (XX)	n/a	n/a	n/a	XX (XX)	XX (XX)	XX (XX)
60 days	XX (XX)	XX (XX)	XX (XX)	XX (XX)	XX (XX)	XX (XX)	XX (XX)
3 months	XX (XX)	XX (XX)	XX (XX)	XX (XX)	XX (XX)	XX (XX)	XX (XX)
6 months	XX (XX)	XX (XX)	XX (XX)	XX (XX)	XX (XX)	XX (XX)	XX (XX)
**Depression score**							
Baseline	XX (XX)	n/a	n/a	n/a	XX (XX)	XX (XX)	XX (XX)
60 days	XX (XX)	XX (XX)	XX (XX)	XX (XX)	XX (XX)	XX (XX)	XX (XX)
3 months	XX (XX)	XX (XX)	XX (XX)	XX (XX)	XX (XX)	XX (XX)	XX (XX)
6 months	XX (XX)	XX (XX)	XX (XX)	XX (XX)	XX (XX)	XX (XX)	XX (XX)
**Anxiety score**							
Baseline	XX (XX)	n/a	n/a	n/a	XX (XX)	XX (XX)	XX (XX)
60 days	XX (XX)	XX (XX)	XX (XX)	XX (XX)	XX (XX)	XX (XX)	XX (XX)
3 months	XX (XX)	XX (XX)	XX (XX)	XX (XX)	XX (XX)	XX (XX)	XX (XX)
6 months	XX (XX)	XX (XX)	XX (XX)	XX (XX)	XX (XX)	XX (XX)	XX (XX)
**Mindfulness score**							
Baseline	XX (XX)	n/a	n/a	n/a	XX (XX)	XX (XX)	XX (XX)

Forbes G, *et al. BMJ Open* 2020; 10:e030164. doi: 10.1136/bmjopen-2019-030164

60 days	XX (XX)	XX (XX)	XX (XX)	XX (XX)	XX (XX)	XX (XX)	XX (XX)
3 months	XX (XX)	XX (XX)	XX (XX)	XX (XX)	XX (XX)	XX (XX)	XX (XX)
6 months	XX (XX)	XX (XX)	XX (XX)	XX (XX)	XX (XX)	XX (XX)	XX (XX)
**Pain related disability score**							
Baseline	XX (XX)	n/a	n/a	n/a	XX (XX)	XX (XX)	XX (XX)
60 days	XX (XX)	XX (XX)	XX (XX)	XX (XX)	XX (XX)	XX (XX)	XX (XX)
3 months	XX (XX)	XX (XX)	XX (XX)	XX (XX)	XX (XX)	XX (XX)	XX (XX)
6 months	XX (XX)	XX (XX)	XX (XX)	XX (XX)	XX (XX)	XX (XX)	XX (XX)
**Self efficacy score**							
Baseline	XX (XX)	n/a	n/a	n/a	XX (XX)	XX (XX)	XX (XX)
60 days	XX (XX)	n/a	n/a	n/a	XX (XX)	XX (XX)	XX (XX)
3 months	XX (XX)	n/a	n/a	n/a	XX (XX)	XX (XX)	XX (XX)
6 months	XX (XX)	n/a	n/a	n/a	XX (XX)	XX (XX)	XX (XX)
**SHOW-Q global score, for sexually active participants**							
Baseline	XX (XX)	n/a	n/a	n/a	XX (XX)	XX (XX)	XX (XX)
60 days	XX (XX)	n/a	n/a	n/a	XX (XX)	XX (XX)	XX (XX)
3 months	XX (XX)	n/a	n/a	n/a	XX (XX)	XX (XX)	XX (XX)
6 months	XX (XX)	n/a	n/a	n/a	XX (XX)	XX (XX)	XX (XX)
**SHOW-Q pelvic problem interference score, for all participants**							
Baseline	XX (XX)	n/a	n/a	n/a	XX (XX)	XX (XX)	XX (XX)
60 days	XX (XX)	n/a	n/a	n/a	XX (XX)	XX (XX)	XX (XX)
3 months	XX (XX)	n/a	n/a	n/a	XX (XX)	XX (XX)	XX (XX)

Forbes G, *et al. BMJ Open* 2020; 10:e030164. doi: 10.1136/bmjopen-2019-030164

6 months	XX (XX)	n/a	n/a	n/a	XX (XX)	XX (XX)	XX (XX)
**Subjective outcome score**							
Baseline	XX (XX)	n/a	n/a	n/a	XX (XX)	XX (XX)	XX (XX)
60 days	XX (XX)	XX (XX)	XX (XX)	XX (XX)	XX (XX)	XX (XX)	XX (XX)
3 months	XX (XX)	XX (XX)	XX (XX)	XX (XX)	XX (XX)	XX (XX)	XX (XX)
6 months	XX (XX)	XX (XX)	XX (XX)	XX (XX)	XX (XX)	XX (XX)	XX (XX)
**SF-36: Physical Functioning**							
Baseline	XX (XX)	n/a	n/a	n/a	XX (XX)	XX (XX)	XX (XX)
60 days	XX (XX)	n/a	n/a	n/a	XX (XX)	XX (XX)	XX (XX)
3 months	XX (XX)	n/a	n/a	n/a	XX (XX)	XX (XX)	XX (XX)
6 months	XX (XX)	n/a	n/a	n/a	XX (XX)	XX (XX)	XX (XX)
**SF-36: Pain**							
Baseline	XX (XX)	n/a	n/a	n/a	XX (XX)	XX (XX)	XX (XX)
60 days	XX (XX)	n/a	n/a	n/a	XX (XX)	XX (XX)	XX (XX)
3 months	XX (XX)	n/a	n/a	n/a	XX (XX)	XX (XX)	XX (XX)
6 months	XX (XX)	n/a	n/a	n/a	XX (XX)	XX (XX)	XX (XX)
**SF-36: General Health**							
Baseline	XX (XX)	n/a	n/a	n/a	XX (XX)	XX (XX)	XX (XX)
60 days	XX (XX)	n/a	n/a	n/a	XX (XX)	XX (XX)	XX (XX)
3 months	XX (XX)	n/a	n/a	n/a	XX (XX)	XX (XX)	XX (XX)
6 months	XX (XX)	n/a	n/a	n/a	XX (XX)	XX (XX)	XX (XX)
**SF-36: Social Functioning**							

Forbes G, *et al. BMJ Open* 2020; 10:e030164. doi: 10.1136/bmjopen-2019-030164

Barts and The London School of Medicine and Dentistry PCTU

Baseline	XX	(XX)	n/a	n/a	n/a	XX	(XX)	XX	(XX)	XX	(XX)
60 days	XX	(XX)	n/a	n/a	n/a	XX	(XX)	XX	(XX)	XX	(XX)
3 months	XX	(XX)	n/a	n/a	n/a	XX	(XX)	XX	(XX)	XX	(XX)
6 months	XX	(XX)	n/a	n/a	n/a	XX	(XX)	XX	(XX)	XX	(XX)

† Questionnaire answered, but all variables used in the derivation of the outcome are missing.
†† One or more, but not all, variables used in the derivation of the outcome are missing.
††† No variables used in the derivation of the outcome are missing.

Forbes G, *et al. BMJ Open* 2020; 10:e030164. doi: 10.1136/bmjopen-2019-030164

13. APPENDIX E: DATA / FILE MANAGEMENT

13.1.1. Sources of data

Copies of CRFs are included in the Statistics Master File. Data is entered from these into a PCTU database. Extracts from the database are supplied by the data manager onto a secure environment.

App usage data will be received from Headspace.

13.1.2. Programming plan

The trial folder on secure environment will contain a folder for each analysis.

An analysis folder should contain the following folders (and their contents):

- analysis data (saved Stata data files for analysis)
- do files (Stata do files for data preparation and analysis)
- log files (Stata log files)
- output (any files output e.g. produced tables and graphs)
- raw data (data as extracted from database)
- temp (any temporary files needed during data preparation or analysis)

Folders containing do files should include a text directory explaining the role of each do file.

13.1.3. Data dictionary

Field names specified in the database "Requirements Specification Document" will be the variable names in the data files. Where a variable is collect on more than one occasion, suffixes will be added to variables names (e.g. "_BASELINE", "_60DAYS", "_3MONTHS", "_6MONTHS").

Details of derived variables are given in Section 5, APPENDIX A: DERIVED AND COMPUTED VARIABLES, and APPENDIX B: STATA CODE FOR GENERATING ADHERENCE OUTCOMES.

A complete data dictionary will be produced for the final analysis data set.

Forbes G, *et al. BMJ Open* 2020; 10:e030164. doi: 10.1136/bmjopen-2019-030164
