# Supplementary material for: MEMPHIS: a smartphone app using psychological approaches for women with chronic pelvic pain presenting to gynaecology clinics: a randomised feasibility trial
Source: BMJ Open. 2020 Mar 12;10(3):e030164. doi: 10.1136/bmjopen-2019-030164 (PMC7069270; doi:10.1136/bmjopen-2019-030164)
Supplement: Supplementary data [file bmjopen-2019-030164supp003.pdf]

Appendix 3: Supplementary tables

TABLE OF CONTENTS

Table 1. Prior and concurrent treatment ... *3*
Table 2. Baseline values of clinical outcomes ... *4*
Table 3. Baseline demographics of woman by 6 month questionnaire completion ... *5*

1. FEASIBILITY OUTCOMES ... 7

1.1. FOLLOW-UP ... 7
Table 4. Losses to follow up ... *7*
Figure 1. Proportion of participants answering follow up questionnaire ... 8
Table 5. Follow-up questionnaire returned or answered by phone within target follow up period ... *9*
1.2. STANDARD DEVIATION OF CPAQ ... 10
Table 6. Estimated standard deviation of CPAQ ... *10*
1.4. BLINDING ... 11
Table 7. Unintentional unbinding of randomised treatment ... *11*

2. APP SATISFACTION QUESTIONNAIRES ... 12

Table 8. System usability scale ... *12*
Table 9. App usability Questionnaire ... *13*

3. CLINICAL OUTCOMES ... 14

3.1. RANGES OF CLINICAL OUTCOMES ... 14
3.2. COMPLETENESS OF CLINICAL DATA ... 15
Table 10. Partially missing clinical outcomes ... *15*
Table 11. Partially missing clinical outcomes by method of questionnaire delivery ... *17*
3.3. RESULTS OF ANALYSIS OF CLINICAL OUTCOMES ... 20
Table 12. Descriptive statistics for clinical outcomes ... *20*
Table 13. Estimated treatment effects for clinical outcomes ... *23*

Forbes G, *et al. BMJ Open* 2020; 10:e030164. doi: 10.1136/bmjopen-2019-030164

Figure 2. Estimated treatment effects and 95% confidence intervals for CPAQ 25

Barts and The London School of Medicine and Dentistry

PCTU

Table 1. Prior and concurrent treatment

Figures are number (percentage).

	Summary measure			Missing data		
	Intervention (N=31)	Active control (N=30)	Usual care (N=29)	Intervention - no. (%)	Active control - no. (%)	Usual care - no. (%)
**Treatment used in the last six months**						
Acupuncture	2 (10.5)	5 (25.0)	1 (6.3)	12 (38.7)	10 (33.3)	13 (44.8)
Massage	11 (50.0)	8 (40.0)	7 (41.2)	9 (29.0)	10 (33.3)	12 (41.4)
Gabapentin	5 (26.3)	1 (5.9)	1 (6.3)	12 (38.7)	13 (43.3)	13 (44.8)
Nutrition/diet	14 (63.6)	14 (63.6)	18 (78.3)	9 (29.0)	8 (26.7)	6 (20.7)
Amitriptyline	5 (27.8)	4 (20.0)	4 (22.2)	13 (41.9)	10 (33.3)	11 (37.9)
Codeine or Morphine type painkillers	13 (56.5)	13 (59.1)	19 (76.0)	8 (25.8)	8 (26.7)	4 (13.8)
Biofeedback	0 (0.0)	0 (0.0)	0 (0.0)	13 (41.9)	12 (40.0)	13 (44.8)
Nerve blocks	0 (0.0)	2 (11.1)	0 (0.0)	14 (45.2)	12 (40.0)	12 (41.4)
Botox injection	0 (0.0)	0 (0.0)	0 (0.0)	14 (45.2)	13 (43.3)	13 (44.8)
Over the counter medication	17 (73.9)	9 (47.4)	17 (77.3)	8 (25.8)	11 (36.7)	7 (24.1)
Contraceptive pills/patch/ring	15 (68.2)	7 (36.8)	11 (52.4)	9 (29.0)	11 (36.7)	8 (27.6)
Physiotherapy	5 (26.3)	4 (20.0)	1 (6.7)	12 (38.7)	10 (33.3)	14 (48.3)
Exercise, yoga or Pilates	13 (59.1)	12 (60.0)	15 (78.9)	9 (29.0)	10 (33.3)	10 (34.5)
Psychological (talking) therapy	3 (16.7)	2 (11.1)	2 (13.3)	13 (41.9)	12 (40.0)	14 (48.3)
Injections to suppress ovaries (e.g. Prostap, Zoladex)	6 (33.3)	5 (25.0)	8 (38.1)	13 (41.9)	10 (33.3)	8 (27.6)
Transcutaneous Electrical Nerve Stimulation (TENS)	0 (0.0)	2 (11.1)	3 (17.6)	13 (41.9)	12 (40.0)	12 (41.4)
Herbal Medicine	4 (21.1)	5 (26.3)	8 (44.4)	12 (38.7)	11 (36.7)	11 (37.9)
Surgery	3 (16.7)	4 (23.5)	6 (31.6)	13 (41.9)	13 (43.3)	10 (34.5)
Meditation or relaxation exercises	11 (47.8)	7 (38.9)	10 (52.6)	8 (25.8)	12 (40.0)	10 (34.5)
Other	3 (37.5)	3 (33.3)	4 (44.4)	23 (74.2)	21 (70.0)	20 (69.0)
Currently using pain treatment				4 (12.9)	3 (10.0)	2 (6.9)
Yes	21 (77.8)	18 (66.7)	20 (74.1)			
No	6 (22.2)	9 (33.3)	7 (25.9)			

Forbes G, *et al. BMJ Open* 2020; 10:e030164. doi: 10.1136/bmjopen-2019-030164

Table 2. Baseline values of clinical outcomes

Figures are mean (SD)

	Summary measure			Missing data		
	**Intervention (N=31)**	**Active control (N=30)**	**Usual care (N=29)**	**Intervention - no. (%)**	**Active control - no. (%)**	**Usual care - no. (%)**
CPAQ pain acceptance score	21.9 (9.5)	22.7 (8.4)	23.8 (8.5)	2 (6.5)	3 (10.0)	1 (3.4)
HADS depression score	8.7 (5.1)	8.6 (5.0)	7.4 (3.6)	1 (3.2)	3 (10.0)	2 (6.9)
HADS anxiety score	12.6 (5.3)	12.0 (5.3)	10.9 (3.9)	1 (3.2)	4 (13.3)	1 (3.4)
CAMS-R mindfulness score	28.6 (6.1)	28.8 (7.1)	30.3 (5.4)	3 (9.7)	5 (16.7)	3 (10.3)
CPG disability score	60.6 (24.4)	64.6 (19.6)	59.2 (24.4)	1 (3.2)	3 (10.0)	1 (3.4)
PSEQ Self efficacy score	29.1 (14.7)	27.9 (14.6)	35.5 (10.6)	1 (3.2)	3 (10.0)	2 (6.9)
Sexual health outcomes:						
SHOW-Q global score*	45.4 (20.3)	50.9 (20.9)	58.1 (22.2)	5 (16.1)	7 (23.3)	3 (10.3)
SHOW-Q pelvic problem interference score	47.1 (29.0)	49.0 (32.7)	56.4 (25.9)	8 (25.8)	6 (20.0)	3 (10.3)
MYMOP subjective outcome score	4.1 (1.2)	3.9 (1.3)	3.9 (1.1)	1 (3.2)	3 (10.0)	2 (6.9)
SF-36 Scales:						
SF36 - Physical functioning	56.3 (30.2)	55.8 (32.2)	66.5 (30.4)	3 (9.7)	4 (13.3)	2 (6.9)
SF36 - Pain	35.1 (17.5)	34.7 (20.6)	37.6 (20.6)	1 (3.2)	3 (10.0)	1 (3.4)
SF36 - General Health	39.1 (20.3)	42.0 (19.8)	37.9 (21.4)	2 (6.5)	3 (10.0)	1 (3.4)
SF36 - Social functioning	37.5 (19.1)	38.0 (28.3)	50.4 (25.3)	1 (3.2)	3 (10.0)	1 (3.4)

*Show-Q global is only applicable for sexually active participants. At baseline there are 17 sexually active women in the intervention group, 22 in the active control group and 19 in the usual care group.

Forbes G, *et al. BMJ Open* 2020; 10:e030164. doi: 10.1136/bmjopen-2019-030164

Barts and The London School of Medicine and Dentistry PCTU

Table 3. Baseline demographics of woman by 6 month questionnaire completion

Figures are mean (SD) unless stated otherwise.

	6 month follow-up questionnaire returned (N=33)	6 month follow-up questionnaire answered by phone (N=24)	6 month follow-up questionnaire never returned (N=33)
**Demographics**			
Age (Years)	35.8 (8.0)	36.6 (9.2)	33.1 (7.5)
Body mass index (kg/m^2)	27.4 (7.1)	27.7 (6.5)	25.9 (4.5)
Living arrangements - no. (%)			
Alone	2 (6.3)	1 (4.2)	3 (10.7)
With others	30 (93.8)	23 (95.8)	25 (89.3)
Employment status - no. (%)			
Employed	26 (78.8)	13 (54.2)	17 (60.7)
Unemployed and looking for work	1 (3.0)	1 (4.2)	1 (3.6)
At school or in full time education	1 (3.0)	2 (8.3)	4 (14.3)
Unable to work due to long term sickness	3 (9.1)	4 (16.7)	3 (10.7)
Looking after your home/family	2 (6.1)	3 (12.5)	3 (10.7)
Retired from paid work	0 (0.0)	1 (4.2)	0 (0.0)
Age left full time education - no. (%)			
Age 12 or less	0 (0.0)	3 (12.5)	0 (0.0)
Age 13 to 16	2 (6.1)	6 (25.0)	8 (29.6)
Age 17 to 19	7 (21.2)	2 (8.3)	5 (18.5)
Age 20 or over	23 (69.7)	9 (37.5)	10 (37.0)
Still in education	1 (3.0)	4 (16.7)	4 (14.8)
Ethnic group - no. (%)			
White	18 (58.1)	9 (40.9)	8 (30.8)
Black	7 (22.6)	4 (18.2)	2 (7.7)
Central Asian	0 (0.0)	0 (0.0)	2 (7.7)
Middle Eastern	0 (0.0)	0 (0.0)	1 (3.8)
Southern Asian	5 (16.1)	6 (27.3)	7 (26.9)
Mixed	1 (3.2)	0 (0.0)	1 (3.8)
Other ethnic group	0 (0.0)	2 (9.1)	4 (15.4)
Do not wish to say	0 (0.0)	1 (4.5)	1 (3.8)

Forbes G, *et al. BMJ Open* 2020; 10:e030164. doi: 10.1136/bmjopen-2019-030164

	6 month follow-up questionnaire returned (N=33)	6 month follow-up questionnaire answered by phone (N=24)	6 month follow-up questionnaire never returned (N=33)
Smoker - no. (%)			
Yes	6 (18.8)	4 (18.2)	7 (25.9)
No	26 (81.3)	18 (81.8)	20 (74.1)
If yes, number of cigarettes per week	36.0 (24.1)	15.3 (12.5)	44.0 (30.8)
Drink alcohol - no. (%)			
Yes	18 (56.3)	6 (27.3)	10 (37.0)
No	14 (43.8)	16 (72.7)	17 (63.0)
If yes, number of units per week	8.9 (7.2)	5.8 (5.3)	5.2 (2.9)
**Baseline medical history**			
Duration of pain - no. (%)			
0 to 6 months	0 (0.0)	1 (4.2)	1 (3.6)
7 to 12 months	3 (9.1)	0 (0.0)	5 (17.9)
1 to 2 years	6 (18.2)	3 (12.5)	4 (14.3)
3 to 5 years	10 (30.3)	10 (41.7)	6 (21.4)
6 to 10 years	5 (15.2)	3 (12.5)	3 (10.7)
More than 10 years	9 (27.3)	7 (29.2)	9 (32.1)
Pain over the past week	6.0 (2.5)	6.0 (2.6)	7.5 (2.2)
**Baseline values of clinical outcomes**			
CPAQ pain acceptance score	25.3 (8.4)	20.8 (8.8)	21.4 (8.7)
HADS depression score	6.6 (3.6)	8.5 (4.9)	10.0 (4.9)
HADS anxiety score	10.3 (4.7)	12.2 (5.1)	13.5 (4.5)
CPG disability score	54.5 (18.8)	65.4 (20.7)	66.0 (27.4)

Forbes G, *et al. BMJ Open* 2020; 10:e030164. doi: 10.1136/bmjopen-2019-030164

1. Feasibility Outcomes

1.1. Follow-up

Table 4. Losses to follow up

	Intervention (N=31)	Active control (N=30)	Usual care (N=29)
**Follow-up questionnaire returned - no (%)**			
60 days	15 (48.4)	9 (30.0)	18 (62.1)
3 months	17 (54.8)	12 (40.0)	17 (58.6)
6 months	11 (35.5)	10 (33.3)	12 (41.4)
**Follow-up questionnaire answered by phone - no (%)**			
60 days	1 (3.2)	8 (26.7)	1 (3.4)
3 months	3 (9.7)	7 (23.3)	3 (10.3)
6 months	10 (32.3)	6 (20.0)	8 (27.6)
**Follow-up questionnaire never returned - no (%)**			
60 days	15 (48.4)	13 (43.3)	10 (34.5)
3 months	11 (35.5)	11 (36.7)	9 (31.0)
6 months	10 (32.3)	14 (46.7)	9 (31.0)

Forbes G, *et al. BMJ Open* 2020; 10:e030164. doi: 10.1136/bmjopen-2019-030164

Figure 1. Proportion of participants answering follow up questionnaire

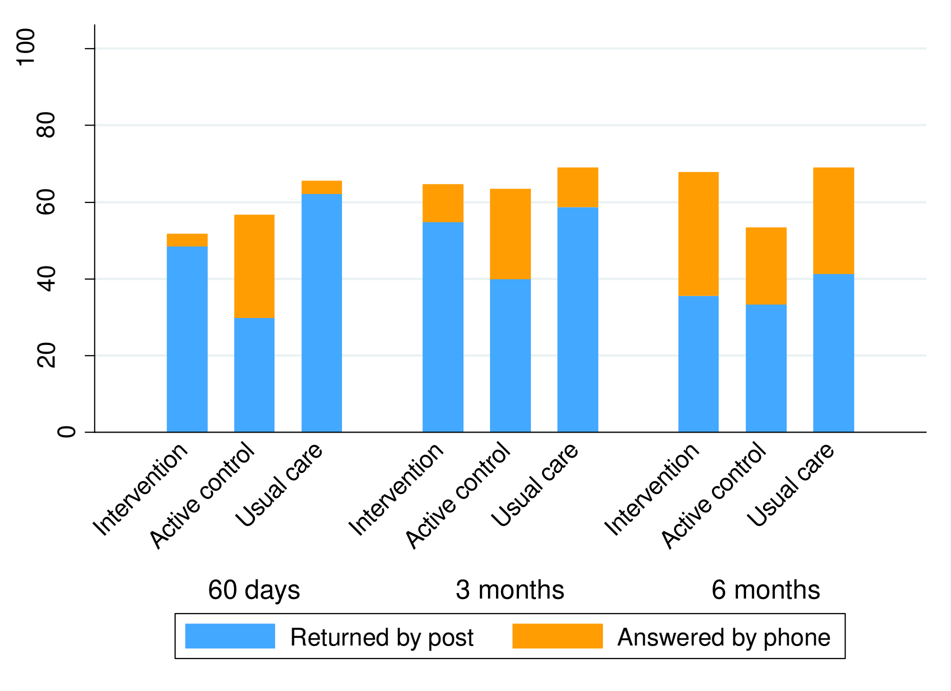

Forbes G, *et al. BMJ Open* 2020; 10:e030164. doi: 10.1136/bmjopen-2019-030164

Table 5. Follow-up questionnaire returned or answered by phone within target follow up period

Figures are no returning data on time/no. returning data questionnaire answering by phone (%)*.

	Intervention (N=31)	Active control (N=30)	Usual care (N=29)
60 days (47 and 74 days)	7/16 (43.8)	6/17 (35.3)	11/19 (57.9)
3 months (76 and 104 days)	7/20 (35.0)	6/19 (31.6)	11/20 (55.0)
6 months (159 and 201 days)	7/21 (33.3)	6/16 (37.5)	11/20 (55.0)

*Denominator for percentage is number returning data questionnaire answering by phone

Forbes G, et al. BMJ Open 2020; 10:e030164. doi: 10.1136/bmjopen-2019-030164

Barts and The London
School of Medicine and Dentistry

PCTU

1.2. Standard deviation of CPAQ

Table 6. Estimated standard deviation of CPAQ

	Number with complete outcome	Estimated standard deviation
60 days	50	9.6
3 months	55	8.1
6 months	56	9.6

Forbes G, *et al. BMJ Open* 2020; 10:e030164. doi: 10.1136/bmjopen-2019-030164

1.4. Blinding

Table 7. Unintentional unbinding of randomised treatment

Figures are number (%)

	Summary measure		Missing data	
	**Intervention (N=31)**	**Active control (N=30)**	**Intervention - no. (%)**	**Active control - no. (%)**
Researchers: Which app treatment do you believe the participant was randomised to?			2 (6.5)	3 (10.0)
Intervention app	0 (0.0)	1 (3.7)		
Control app	0 (0.0)	0 (0.0)		
Don't know	29 (100.0)	26 (96.3)		
Participants: Do you think you received the new treatment or comparison treatment?			15 (48.4)	19 (63.3)
New treatment	1 (6.3)	1 (9.1)		
Comparison treatment	0 (0.0)	1 (9.1)		
Don't know	15 (93.8)	9 (81.8)		

Forbes G, et al. BMJ Open 2020; 10:e030164. doi: 10.1136/bmjopen-2019-030164

2. App satisfaction questionnaires

Table 8. System usability scale

Figures are mean (sd).

	**Summary measure**		**Missing Data**	
	Intervention (N=31)	Active control (N=30)	Intervention - no. (%)	Active control - no. (%)
System usability scale	50.7 (6.6)	46.0 (12.0)	16 (51.6)	18 (60.0)

Forbes G, *et al. BMJ Open* 2020; 10:e030164. doi: 10.1136/bmjopen-2019-030164

Table 9. App usability Questionnaire

Figures are number (%).

	Totally disagree	Somewhat disagree	Neither agree nor disagree	Somewhat agree	Totally agree	Not answered
It is easy to use the app whenever I wanted to use it						
Intervention:	0 (0.0)	3 (9.7)	1 (3.2)	3 (9.7)	9 (29.0)	15 (48.4)
Active control:	0 (0.0)	0 (0.0)	0 (0.0)	4 (13.3)	8 (26.7)	18 (60.0)
After being shown, I understood how the app would work						
Intervention:	0 (0.0)	1 (3.2)	1 (3.2)	4 (12.9)	9 (29.0)	16 (51.6)
Active control:	0 (0.0)	2 (6.7)	0 (0.0)	2 (6.7)	8 (26.7)	18 (60.0)
It was fun to work with the app						
Intervention:	0 (0.0)	2 (6.5)	3 (9.7)	8 (25.8)	2 (6.5)	16 (51.6)
Active control:	0 (0.0)	3 (10.0)	3 (10.0)	5 (16.7)	1 (3.3)	18 (60.0)
The app worked well						
Intervention:	0 (0.0)	3 (9.7)	2 (6.5)	5 (16.1)	5 (16.1)	16 (51.6)
Active control:	0 (0.0)	2 (6.7)	2 (6.7)	4 (13.3)	4 (13.3)	18 (60.0)
It was easy to work through the modules						
Intervention:	0 (0.0)	4 (12.9)	0 (0.0)	5 (16.1)	6 (19.4)	16 (51.6)
Active control:	0 (0.0)	2 (6.7)	3 (10.0)	4 (13.3)	3 (10.0)	18 (60.0)
The number of modules was annoying						
Intervention:	1 (3.2)	4 (12.9)	6 (19.4)	5 (16.1)	0 (0.0)	15 (48.4)
Active control:	2 (6.7)	3 (10.0)	3 (10.0)	2 (6.7)	2 (6.7)	18 (60.0)
The modules were well-displayed on my smartphone						
Intervention:	0 (0.0)	2 (6.5)	1 (3.2)	8 (25.8)	5 (16.1)	15 (48.4)
Active control:	0 (0.0)	0 (0.0)	2 (6.7)	2 (6.7)	7 (23.3)	19 (63.3)
Using the app was difficult because of my daily activities						
Intervention:	2 (6.5)	2 (6.5)	3 (9.7)	9 (29.0)	0 (0.0)	15 (48.4)
Active control:	1 (3.3)	1 (3.3)	0 (0.0)	5 (16.7)	5 (16.7)	18 (60.0)
Using the app took too long						
Intervention:	2 (6.5)	4 (12.9)	3 (9.7)	7 (22.6)	0 (0.0)	15 (48.4)
Active control:	1 (3.3)	3 (10.0)	2 (6.7)	4 (13.3)	2 (6.7)	18 (60.0)

Forbes G, *et al. BMJ Open* 2020; 10:e030164. doi: 10.1136/bmjopen-2019-030164

3. Clinical Outcomes

3.1. Ranges of clinical outcomes

Pain acceptance score (as measured by the Chronic Pain Acceptance Questionnaire (CPAQ)):

- 0 (worst) – 48 (best)

Depression score (Hospital Anxiety and Depression Scale (HADS)):

- 0 (best) – 21 (worst)

Anxiety score (measured by HADS):

- 0 (best) – 21 (worst)

Mindfulness score (Cognitive and Mindfulness - Revised (CAMS – R) scale):

- 12 (worst) – 48 (best)

Pain related disability score (as measured by the Chronic Pain Grade (CPG) disability subscale):

- 0 (best) – 100 (worst)

Self efficacy score (as measured by the Pain Self-Efficacy Questionnaire (PSEQ)):

- 0 (worst) – 60 (best)

Sexual Health Outcomes scores (as measured by the Sexual Health Outcomes in Women Questionnaire (SHOW-Q))

- SHOW-Q global score, for sexually active participants: 0 (worst) – 100 (best)
- SHOW-Q pelvic interference score, for all participants: 0 (best) – 100 (worst)

Subjective outcome score (as measured by the Measure Yourself Medical Outcome Profile (MYMOP)):

- 0 (best) – 6)worst)

RAND Short form (36) Health Survey (RAND SF-36) scales:

- Physical functioning: 0 (worst) – 100)best)
- Pain: 0 (worst) – 100 (best)
- General health: 0 (worst) – 100 (best)
- Social functioning: 0 (worst) – 100 (best)

Forbes G, *et al. BMJ Open* 2020; 10:e030164. doi: 10.1136/bmjopen-2019-030164

3.2. Completeness of clinical data

Table 10. Partially missing clinical outcomes

Figures are number (%)

	Not completed* no. (%)	Partially completed** no. (%)	Fully completed*** no. (%)
**CPAQ pain acceptance score**			
Baseline	5 (5.6)	1 (1.1)	84 (93.3)
60 days	40 (44.4)	0 (0.0)	50 (55.6)
3 months	32 (35.6)	3 (3.3)	55 (61.1)
6 months	34 (37.8)	0 (0.0)	56 (62.2)
**HADS depression score**			
Baseline	5 (5.6)	1 (1.1)	84 (93.3)
60 days	40 (44.4)	1 (1.1)	49 (54.4)
3 months	32 (35.6)	0 (0.0)	58 (64.4)
6 months	33 (36.7)	0 (0.0)	57 (63.3)
**HADS anxiety score**			
Baseline	5 (5.6)	1 (1.1)	84 (93.3)
60 days	40 (44.4)	0 (0.0)	50 (55.6)
3 months	32 (35.6)	0 (0.0)	58 (64.4)
6 months	33 (36.7)	0 (0.0)	57 (63.3)
**CAMS-R mindfulness score**			
Baseline	5 (5.6)	6 (6.7)	79 (87.8)
60 days	40 (44.4)	0 (0.0)	50 (55.6)
3 months	32 (35.6)	2 (2.2)	56 (62.2)
6 months	33 (36.7)	0 (0.0)	57 (63.3)
**CPG disability score**			
Baseline	5 (5.6)	0 (0.0)	85 (94.4)
60 days	40 (44.4)	0 (0.0)	50 (55.6)
3 months	33 (36.7)	0 (0.0)	57 (63.3)
6 months	34 (37.8)	0 (0.0)	56 (62.2)
**PSEQ Self efficacy score**			
Baseline	5 (5.6)	1 (1.1)	84 (93.3)
60 days	50 (55.6)	1 (1.1)	39 (43.3)
3 months	45 (50.0)	0 (0.0)	45 (50.0)
6 months	57 (63.3)	0 (0.0)	33 (36.7)

* Questionnaire not answered or all variables used in the derivation of the outcome are missing.
** One or more, but not all, variables used in the derivation of the outcome are missing.
*** No variables used in the derivation of the outcome are missing.

Forbes G, *et al. BMJ Open* 2020; 10:e030164. doi: 10.1136/bmjopen-2019-030164

Barts and The London School of Medicine and Dentistry PCTU

	Not completed* no. (%)	Partially completed** no. (%)	Fully completed*** no. (%)
**SHOW-Q global score**			
Baseline	5 (5.6)	15 (16.7)	70 (77.8)
60 days	50 (55.6)	6 (6.7)	34 (37.8)
3 months	47 (52.2)	8 (8.9)	35 (38.9)
6 months	58 (64.4)	5 (5.6)	27 (30.0)
**SHOW-Q pelvic problem interference score**			
Baseline	9 (10.0)	8 (8.9)	73 (81.1)
60 days	51 (56.7)	3 (3.3)	36 (40.0)
3 months	49 (54.4)	3 (3.3)	38 (42.2)
6 months	60 (66.7)	1 (1.1)	29 (32.2)
**MYMOP subjective outcome score**			
Baseline	5 (5.6)	1 (1.1)	84 (93.3)
60 days	38 (42.2)	11 (12.2)	41 (45.6)
3 months	33 (36.7)	10 (11.1)	47 (52.2)
6 months	33 (36.7)	6 (6.7)	51 (56.7)
**SF36 - General Health**			
Baseline	5 (5.6)	1 (1.1)	84 (93.3)
60 days	38 (42.2)	11 (12.2)	41 (45.6)
3 months	31 (34.4)	14 (15.6)	45 (50.0)
6 months	33 (36.7)	24 (26.7)	33 (36.7)
**SF36 - Physical functioning**			
Baseline	5 (5.6)	4 (4.4)	81 (90.0)
60 days	48 (53.3)	3 (3.3)	39 (43.3)
3 months	45 (50.0)	2 (2.2)	43 (47.8)
6 months	57 (63.3)	3 (3.3)	30 (33.3)
**SF36 - Pain**			
Baseline	5 (5.6)	0 (0.0)	85 (94.4)
60 days	48 (53.3)	0 (0.0)	42 (46.7)
3 months	45 (50.0)	0 (0.0)	45 (50.0)
6 months	57 (63.3)	0 (0.0)	33 (36.7)
**SF36 - Social functioning**			
Baseline	5 (5.6)	0 (0.0)	85 (94.4)
60 days	48 (53.3)	1 (1.1)	41 (45.6)
3 months	45 (50.0)	1 (1.1)	44 (48.9)
6 months	57 (63.3)	1 (1.1)	32 (35.6)

* Questionnaire not answered or all variables used in the derivation of the outcome are missing.
** One or more, but not all, variables used in the derivation of the outcome are missing.
*** No variables used in the derivation of the outcome are missing.

Forbes G, *et al. BMJ Open* 2020; 10:e030164. doi: 10.1136/bmjopen-2019-030164

Table 11. Partially missing clinical outcomes by method of questionnaire delivery

Figures are number (%)

	Questionnaire never returned no. (%)	Questionnaire answered by telephone: Not completed* no. (%)	Questionnaire answered by telephone: Partially completed** no. (%)	Questionnaire answered by telephone: Fully completed*** no. (%)	Questionnaire returned: Not completed* no. (%)	Questionnaire returned: Partially completed** no. (%)	Questionnaire returned: Fully completed*** no. (%)
**CPAQ pain acceptance score**							
Baseline	5 (5.6)	n/a	n/a	n/a	0 (0.0)	1 (1.1)	84 (93.3)
60 days	38 (42.2)	1 (1.1)	0 (0.0)	9 (10.0)	1 (1.1)	0 (0.0)	41 (45.6)
3 months	31 (34.4)	0 (0.0)	0 (0.0)	13 (14.4)	1 (1.1)	3 (3.3)	42 (46.7)
6 months	33 (36.7)	1 (1.1)	0 (0.0)	23 (25.6)	0 (0.0)	0 (0.0)	33 (36.7)
**HADS depression score**							
Baseline	5 (5.6)	n/a	n/a	n/a	0 (0.0)	1 (1.1)	84 (93.3)
60 days	38 (42.2)	1 (1.1)	0 (0.0)	9 (10.0)	1 (1.1)	1 (1.1)	40 (44.4)
3 months	31 (34.4)	0 (0.0)	0 (0.0)	13 (14.4)	1 (1.1)	0 (0.0)	45 (50.0)
6 months	33 (36.7)	0 (0.0)	0 (0.0)	24 (26.7)	0 (0.0)	0 (0.0)	33 (36.7)
**HADS anxiety score**							
Baseline	5 (5.6)	n/a	n/a	n/a	0 (0.0)	1 (1.1)	84 (93.3)
60 days	38 (42.2)	1 (1.1)	0 (0.0)	9 (10.0)	1 (1.1)	0 (0.0)	41 (45.6)
3 months	31 (34.4)	0 (0.0)	0 (0.0)	13 (14.4)	1 (1.1)	0 (0.0)	45 (50.0)
6 months	33 (36.7)	0 (0.0)	0 (0.0)	24 (26.7)	0 (0.0)	0 (0.0)	33 (36.7)
**CAMS-R mindfulness score**							
Baseline	5 (5.6)	n/a	n/a	n/a	0 (0.0)	6 (6.7)	79 (87.8)
60 days	38 (42.2)	1 (1.1)	0 (0.0)	9 (10.0)	1 (1.1)	0 (0.0)	41 (45.6)
3 months	31 (34.4)	0 (0.0)	0 (0.0)	13 (14.4)	1 (1.1)	2 (2.2)	43 (47.8)
6 months	33 (36.7)	0 (0.0)	0 (0.0)	24 (26.7)	0 (0.0)	0 (0.0)	33 (36.7)
**CPG disability score**							
Baseline	5 (5.6)	n/a	n/a	n/a	0 (0.0)	0 (0.0)	85 (94.4)
60 days	38 (42.2)	1 (1.1)	0 (0.0)	9 (10.0)	1 (1.1)	0 (0.0)	41 (45.6)
3 months	31 (34.4)	0 (0.0)	0 (0.0)	13 (14.4)	2 (2.2)	0 (0.0)	44 (48.9)
6 months	33 (36.7)	1 (1.1)	0 (0.0)	23 (25.6)	0 (0.0)	0 (0.0)	33 (36.7)

* Questionnaire not answered or all variables used in the derivation of the outcome are missing.
** One or more, but not all, variables used in the derivation of the outcome are missing.
*** No variables used in the derivation of the outcome are missing.

Forbes G, *et al. BMJ Open* 2020; 10:e030164. doi: 10.1136/bmjopen-2019-030164

	Questionnaire never returned no. (%)	Questionnaire answered by telephone: Not completed* no. (%)	Questionnaire answered by telephone: Partially completed** no. (%)	Questionnaire answered by telephone: Fully completed*** no. (%)	Questionnaire returned: Not completed* no. (%)	Questionnaire returned: Partially completed** no. (%)	Questionnaire returned: Fully completed*** no. (%)
**PSEQ Self efficacy score**							
Baseline	5 (5.6)	n/a	n/a	n/a	0 (0.0)	1 (1.1)	84 (93.3)
60 days	38 (42.2)	10 (11.1)	0 (0.0)	0 (0.0)	2 (2.2)	1 (1.1)	39 (43.3)
3 months	31 (34.4)	13 (14.4)	0 (0.0)	0 (0.0)	1 (1.1)	0 (0.0)	45 (50.0)
6 months	33 (36.7)	24 (26.7)	0 (0.0)	0 (0.0)	0 (0.0)	0 (0.0)	33 (36.7)
**SHOW-Q global score**							
Baseline	5 (5.6)	n/a	n/a	n/a	0 (0.0)	15 (16.7)	70 (77.8)
60 days	38 (42.2)	10 (11.1)	0 (0.0)	0 (0.0)	2 (2.2)	6 (6.7)	34 (37.8)
3 months	31 (34.4)	13 (14.4)	0 (0.0)	0 (0.0)	3 (3.3)	8 (8.9)	35 (38.9)
6 months	33 (36.7)	24 (26.7)	0 (0.0)	0 (0.0)	1 (1.1)	5 (5.6)	27 (30.0)
**SHOW-Q pelvic problem interference score**							
Baseline	5 (5.6)	n/a	n/a	n/a	4 (4.4)	8 (8.9)	73 (81.1)
60 days	38 (42.2)	10 (11.1)	0 (0.0)	0 (0.0)	3 (3.3)	3 (3.3)	36 (40.0)
3 months	31 (34.4)	13 (14.4)	0 (0.0)	0 (0.0)	5 (5.6)	3 (3.3)	38 (42.2)
6 months	33 (36.7)	24 (26.7)	0 (0.0)	0 (0.0)	3 (3.3)	1 (1.1)	29 (32.2)
**MYMOP subjective outcome score**							
Baseline	5 (5.6)	n/a	n/a	n/a	0 (0.0)	1 (1.1)	84 (93.3)
60 days	38 (42.2)	0 (0.0)	1 (1.1)	9 (10.0)	0 (0.0)	10 (11.1)	32 (35.6)
3 months	31 (34.4)	0 (0.0)	1 (1.1)	12 (13.3)	2 (2.2)	9 (10.0)	35 (38.9)
6 months	33 (36.7)	0 (0.0)	2 (2.2)	22 (24.4)	0 (0.0)	4 (4.4)	29 (32.2)

* Questionnaire not answered or all variables used in the derivation of the outcome are missing.
** One or more, but not all, variables used in the derivation of the outcome are missing.
*** No variables used in the derivation of the outcome are missing.

Forbes G, *et al. BMJ Open* 2020; 10:e030164. doi: 10.1136/bmjopen-2019-030164

Barts and The London School of Medicine and Dentistry PCTU

	Questionnaire never returned no. (%)	Questionnaire answered by telephone: Not completed* no. (%)	Questionnaire answered by telephone: Partially completed** no. (%)	Questionnaire answered by telephone: Fully completed*** no. (%)	Questionnaire returned: Not completed* no. (%)	Questionnaire returned: Partially completed** no. (%)	Questionnaire returned: Fully completed*** no. (%)
**SF36 - General Health**							
Baseline	5 (5.6)	n/a	n/a	n/a	0 (0.0)	1 (1.1)	84 (93.3)
60 days	38 (42.2)	10 (11.1)	0 (0.0)	0 (0.0)	0 (0.0)	1 (1.1)	41 (45.6)
3 months	31 (34.4)	13 (14.4)	0 (0.0)	0 (0.0)	0 (0.0)	1 (1.1)	45 (50.0)
6 months	33 (36.7)	24 (26.7)	0 (0.0)	0 (0.0)	0 (0.0)	0 (0.0)	33 (36.7)
**SF36 - Physical functioning**							
Baseline	5 (5.6)	n/a	n/a	n/a	0 (0.0)	4 (4.4)	81 (90.0)
60 days	38 (42.2)	10 (11.1)	0 (0.0)	0 (0.0)	0 (0.0)	3 (3.3)	39 (43.3)
3 months	31 (34.4)	13 (14.4)	0 (0.0)	0 (0.0)	1 (1.1)	2 (2.2)	43 (47.8)
6 months	33 (36.7)	24 (26.7)	0 (0.0)	0 (0.0)	0 (0.0)	3 (3.3)	30 (33.3)
**SF36 - Pain**							
Baseline	5 (5.6)	n/a	n/a	n/a	0 (0.0)	0 (0.0)	85 (94.4)
60 days	38 (42.2)	10 (11.1)	0 (0.0)	0 (0.0)	0 (0.0)	0 (0.0)	42 (46.7)
3 months	31 (34.4)	13 (14.4)	0 (0.0)	0 (0.0)	1 (1.1)	0 (0.0)	45 (50.0)
6 months	33 (36.7)	24 (26.7)	0 (0.0)	0 (0.0)	0 (0.0)	0 (0.0)	33 (36.7)
**SF36 - Social functioning**							
Baseline	5 (5.6)	n/a	n/a	n/a	0 (0.0)	0 (0.0)	85 (94.4)
60 days	38 (42.2)	10 (11.1)	0 (0.0)	0 (0.0)	0 (0.0)	1 (1.1)	41 (45.6)
3 months	31 (34.4)	13 (14.4)	0 (0.0)	0 (0.0)	1 (1.1)	1 (1.1)	44 (48.9)
6 months	33 (36.7)	24 (26.7)	0 (0.0)	0 (0.0)	0 (0.0)	1 (1.1)	32 (35.6)

* Questionnaire not answered or all variables used in the derivation of the outcome are missing.
** One or more, but not all, variables used in the derivation of the outcome are missing.
*** No variables used in the derivation of the outcome are missing.

Forbes G, *et al. BMJ Open* 2020; 10:e030164. doi: 10.1136/bmjopen-2019-030164

3.3. Results of analysis of clinical outcomes

Table 12. Descriptive statistics for clinical outcomes

	Intervention (N=31)		Active control (N=30)		Usual care (N=29)	
	no. (%)	mean (sd)	no. (%)	mean (sd)	no. (%)	mean (sd)
**CPAQ pain acceptance score**						
Baseline	29 (93.5)	21.9 (9.5)	27 (90.0)	22.7 (8.4)	28 (96.6)	23.8 (8.5)
60 days	15 (48.4)	21.5 (10.2)	16 (53.3)	22.9 (8.5)	19 (65.5)	24.3 (10.2)
3 months	18 (58.1)	20.8 (7.2)	18 (60.0)	22.9 (8.5)	19 (65.5)	25.0 (8.4)
6 months	21 (67.7)	22.7 (10.1)	16 (53.3)	24.0 (11.2)	19 (65.5)	25.8 (7.6)
Included in analysis*	27 (87.1)		23 (76.7)		25 (86.2)	
**HADS depression score**						
Baseline	30 (96.8)	8.7 (5.1)	27 (90.0)	8.6 (5.0)	27 (93.1)	7.4 (3.6)
60 days	14 (45.2)	7.1 (5.2)	16 (53.3)	8.4 (4.0)	19 (65.5)	8.2 (2.9)
3 months	20 (64.5)	8.7 (3.9)	19 (63.3)	8.2 (5.0)	19 (65.5)	6.8 (3.6)
6 months	21 (67.7)	7.0 (4.9)	16 (53.3)	6.1 (4.4)	20 (69.0)	7.0 (4.6)
Included in analysis*	27 (87.1)		23 (76.7)		26 (89.7)	
**HADS anxiety score**						
Baseline	30 (96.8)	12.6 (5.3)	26 (86.7)	12.0 (5.3)	28 (96.6)	10.9 (3.9)
60 days	15 (48.4)	12.5 (5.6)	16 (53.3)	9.5 (4.1)	19 (65.5)	10.7 (4.1)
3 months	20 (64.5)	12.2 (4.1)	19 (63.3)	9.7 (5.6)	19 (65.5)	10.2 (4.0)
6 months	21 (67.7)	10.1 (4.9)	16 (53.3)	8.4 (5.5)	20 (69.0)	9.1 (4.7)
Included in analysis*	27 (87.1)		23 (76.7)		26 (89.7)	
**CAMS-R mindfulness score**						
Baseline	28 (90.3)	28.6 (6.1)	25 (83.3)	28.8 (7.1)	26 (89.7)	30.3 (5.4)
60 days	15 (48.4)	27.4 (5.6)	16 (53.3)	30.6 (8.4)	19 (65.5)	29.7 (7.6)
3 months	19 (61.3)	29.2 (5.2)	19 (63.3)	30.9 (8.8)	18 (62.1)	31.4 (6.4)
6 months	21 (67.7)	29.0 (7.6)	16 (53.3)	31.0 (7.3)	20 (69.0)	32.0 (8.5)
Included in analysis*	27 (87.1)		23 (76.7)		26 (89.7)	

* Included in analysis if outcome is available for at least one follow-up time point.

Forbes G, *et al. BMJ Open* 2020; 10:e030164. doi: 10.1136/bmjopen-2019-030164

	Intervention (N=31)		Active control (N=30)		Usual care (N=29)	
	no. (%)	mean (sd)	no. (%)	mean (sd)	no. (%)	mean (sd)
**CPG disability score**						
Baseline	30 (96.8)	60.6 (24.4)	27 (90.0)	64.6 (19.6)	28 (96.6)	59.2 (24.4)
60 days	15 (48.4)	56.7 (19.8)	16 (53.3)	54.8 (25.0)	19 (65.5)	54.7 (22.9)
3 months	19 (61.3)	61.1 (17.3)	19 (63.3)	52.5 (27.5)	19 (65.5)	52.8 (23.5)
6 months	21 (67.7)	48.3 (28.1)	16 (53.3)	48.5 (24.4)	19 (65.5)	54.2 (23.7)
Included in analysis*	27 (87.1)		23 (76.7)		25 (86.2)	
**PSEQ Self efficacy score**						
Baseline	30 (96.8)	29.1 (14.7)	27 (90.0)	27.9 (14.6)	27 (93.1)	35.5 (10.6)
60 days	14 (45.2)	32.4 (13.9)	9 (30.0)	30.9 (15.9)	16 (55.2)	34.5 (13.1)
3 months	17 (54.8)	28.9 (11.8)	12 (40.0)	30.2 (14.2)	16 (55.2)	39.3 (9.7)
6 months	11 (35.5)	34.3 (12.5)	10 (33.3)	33.7 (17.7)	12 (41.4)	40.2 (13.1)
Included in analysis*	21 (67.7)		18 (60.0)		21 (72.4)	
**SHOW-Q global score**						
Baseline	17 (54.8)	45.4 (20.3)	20 (66.7)	50.9 (20.9)	19 (65.5)	58.1 (22.2)
60 days	4 (12.9)	69.3 (13.3)	8 (26.7)	54.1 (18.0)	13 (44.8)	53.7 (24.5)
3 months	5 (16.1)	51.1 (26.6)	11 (36.7)	44.9 (19.4)	10 (34.5)	61.2 (24.8)
6 months	7 (22.6)	52.3 (15.6)	4 (13.3)	60.9 (14.3)	7 (24.1)	58.5 (26.4)
Included in analysis*	9 (29.0)		14 (46.7)		16 (55.2)	
**SHOW-Q pelvic problem interference score**						
Baseline	23 (74.2)	47.1 (29.0)	24 (80.0)	49.0 (32.7)	26 (89.7)	56.4 (25.9)
60 days	12 (38.7)	60.4 (33.7)	9 (30.0)	60.2 (27.9)	15 (51.7)	51.7 (28.9)
3 months	12 (38.7)	54.9 (34.0)	11 (36.7)	50.0 (25.3)	15 (51.7)	69.4 (32.8)
6 months	9 (29.0)	65.7 (22.2)	9 (30.0)	59.3 (33.4)	11 (37.9)	57.6 (32.8)
Included in analysis*	16 (51.6)		17 (56.7)		20 (69.0)	
**MYMOP subjective outcome score**						
Baseline	30 (96.8)	4.1 (1.2)	27 (90.0)	3.9 (1.3)	27 (93.1)	3.9 (1.1)
60 days	13 (41.9)	3.2 (1.4)	14 (46.7)	3.5 (1.3)	14 (48.3)	3.6 (1.2)
3 months	15 (48.4)	3.4 (1.3)	16 (53.3)	3.1 (1.6)	16 (55.2)	2.9 (1.4)
6 months	18 (58.1)	3.0 (1.4)	15 (50.0)	3.0 (1.5)	18 (62.1)	3.1 (1.5)
Included in analysis*	25 (80.6)		21 (70.0)		24 (82.8)	

* Included in analysis if outcome is available for at least one follow-up time point.

Barts and The London School of Medicine and Dentistry PCTU

	Intervention (N=31)		Active control (N=30)		Usual care (N=29)	
	no. (%)	mean (sd)	no. (%)	mean (sd)	no. (%)	mean (sd)
**SF36 - General Health**						
Baseline	29 (93.5)	39.1 (20.3)	27 (90.0)	42.0 (19.8)	28 (96.6)	37.9 (21.4)
60 days	15 (48.4)	45.0 (21.2)	9 (30.0)	51.1 (19.2)	17 (58.6)	37.6 (19.9)
3 months	17 (54.8)	44.1 (21.7)	12 (40.0)	42.1 (23.2)	16 (55.2)	40.3 (19.4)
6 months	11 (35.5)	54.5 (19.0)	10 (33.3)	54.5 (24.2)	12 (41.4)	40.0 (27.8)
Included in analysis*	21 (67.7)		18 (60.0)		22 (75.9)	
**SF36 - Physical functioning**						
Baseline	28 (90.3)	56.3 (30.2)	26 (86.7)	55.8 (32.2)	27 (93.1)	66.5 (30.4)
60 days	13 (41.9)	61.2 (27.1)	9 (30.0)	60.6 (25.7)	17 (58.6)	66.5 (30.0)
3 months	15 (48.4)	58.3 (24.0)	12 (40.0)	54.6 (30.7)	16 (55.2)	69.1 (27.5)
6 months	10 (32.3)	66.0 (26.5)	10 (33.3)	72.0 (28.6)	10 (34.5)	63.5 (37.4)
Included in analysis*	20 (64.5)		18 (60.0)		22 (75.9)	
**SF36 - Pain**						
Baseline	30 (96.8)	35.1 (17.5)	27 (90.0)	34.7 (20.6)	28 (96.6)	37.6 (20.6)
60 days	15 (48.4)	39.0 (19.2)	9 (30.0)	43.1 (33.0)	18 (62.1)	40.0 (24.5)
3 months	17 (54.8)	43.7 (17.6)	12 (40.0)	46.7 (22.7)	16 (55.2)	49.5 (25.9)
6 months	11 (35.5)	50.0 (17.8)	10 (33.3)	61.0 (19.9)	12 (41.4)	48.3 (24.8)
Included in analysis*	21 (67.7)		18 (60.0)		22 (75.9)	
**SF36 - Social functioning**						
Baseline	30 (96.8)	37.5 (19.1)	27 (90.0)	38.0 (28.3)	28 (96.6)	50.4 (25.3)
60 days	15 (48.4)	45.8 (27.4)	9 (30.0)	55.6 (29.4)	17 (58.6)	51.5 (28.9)
3 months	17 (54.8)	50.7 (20.9)	12 (40.0)	49.0 (30.4)	15 (51.7)	57.5 (29.0)
6 months	11 (35.5)	54.5 (21.8)	10 (33.3)	56.3 (27.8)	11 (37.9)	59.1 (34.0)
Included in analysis*	21 (67.7)		18 (60.0)		22 (75.9)	

* Included in analysis if outcome is available for at least one follow-up time point.

Forbes G, *et al. BMJ Open* 2020; 10:e030164. doi: 10.1136/bmjopen-2019-030164

Table 13. Estimated treatment effects for clinical outcomes

	Intervention vs Active control adjusted mean difference (95% CI)	Intervention vs Usual care adjusted mean difference (95% CI)	Active control vs Usual care adjusted mean difference (95% CI)
**CPAQ pain acceptance score**			
60 days	-2.3 (-6.6, 2.0)	-4.0 (-8.1, 0.1)	-1.7 (-5.8, 2.4)
3 months	-3.0 (-6.8, 0.7)	-4.5 (-8.2, -0.9)	-1.5 (-5.2, 2.2)
6 months	-1.4 (-5.8, 3.0)	-4.0 (-8.2, 0.2)	-2.5 (-7.0, 2.0)
**HADS depression score**			
60 days	-0.7 (-2.7, 1.2)	-1.2 (-3.1, 0.6)	-0.5 (-2.3, 1.3)
3 months	0.5 (-1.6, 2.6)	1.2 (-0.9, 3.4)	0.8 (-1.4, 2.9)
6 months	0.5 (-1.7, 2.6)	0.4 (-1.7, 2.4)	-0.1 (-2.3, 2.1)
**HADS anxiety score**			
60 days	2.0 (-0.1, 4.1)	1.0 (-1.1, 3.0)	-1.0 (-3.0, 1.0)
3 months	1.9 (-0.3, 4.0)	1.5 (-0.6, 3.6)	-0.4 (-2.5, 1.7)
6 months	0.1 (-2.3, 2.5)	0.3 (-2.0, 2.6)	0.2 (-2.2, 2.6)
**CAMS-R mindfulness score**			
60 days	-3.5 (-7.3, 0.4)	-2.2 (-5.9, 1.4)	1.2 (-2.5, 4.9)
3 months	-2.5 (-5.8, 0.8)	-2.3 (-5.5, 1.0)	0.2 (-3.1, 3.5)
6 months	-1.4 (-4.9, 2.2)	-2.9 (-6.3, 0.4)	-1.6 (-5.1, 2.0)
**CPG disability score**			
60 days	5.1 (-7.2, 17.5)	3.8 (-8.1, 15.7)	-1.4 (-13.1, 10.4)
3 months	8.8 (-3.4, 21.0)	7.6 (-4.5, 19.7)	-1.2 (-13.4, 10.9)
6 months	1.9 (-12.1, 16.0)	1.0 (-12.6, 14.5)	-1.0 (-15.3, 13.4)
**PSEQ Self efficacy score**			
60 days	0.1 (-8.2, 8.4)	-0.2 (-7.4, 6.9)	-0.3 (-8.4, 7.8)
3 months	-3.6 (-9.8, 2.6)	-7.1 (-12.9, -1.2)	-3.5 (-9.8, 2.9)
6 months	-5.9 (-14.8, 3.0)	-8.7 (-17.1, -0.2)	-2.8 (-11.6, 5.9)

Forbes G, et al. BMJ Open 2020; 10:e030164. doi: 10.1136/bmjopen-2019-030164

Barts and The London
School of Medicine and Dentistry

PCTU

	Intervention vs Active control adjusted mean difference (95% CI)	Intervention vs Usual care adjusted mean difference (95% CI)	Active control vs Usual care adjusted mean difference (95% CI)
**SHOW-Q global score**			
60 days	7.0 (-7.2, 21.2)	8.3 (-5.2, 21.8)	1.3 (-9.8, 12.4)
3 months	3.5 (-13.9, 20.9)	-4.8 (-22.0, 12.3)	-8.3 (-23.2, 6.6)
6 months	-11.5 (-27.7, 4.8)	-10.7 (-25.8, 4.3)	0.7 (-14.5, 15.9)
**SHOW-Q pelvic problem interference score**			
60 days	-7.2 (-28.0, 13.5)	3.6 (-14.7, 21.9)	10.9 (-8.9, 30.7)
3 months	-1.2 (-25.1, 22.8)	-10.2 (-32.5, 12.1)	-9.0 (-31.9, 13.8)
6 months	3.3 (-21.3, 27.9)	4.7 (-18.7, 28.1)	1.4 (-22.1, 24.8)
**MYMOP subjective outcome score**			
60 days	0.0 (-0.7, 0.8)	-0.3 (-1.1, 0.4)	-0.4 (-1.1, 0.4)
3 months	0.6 (-0.2, 1.5)	0.6 (-0.2, 1.4)	-0.0 (-0.9, 0.8)
6 months	-0.2 (-1.1, 0.7)	0.2 (-0.7, 1.1)	0.4 (-0.6, 1.3)
**SF36 - General Health**			
60 days	-8.8 (-19.4, 1.8)	-0.9 (-10.0, 8.3)	7.9 (-2.5, 18.3)
3 months	2.0 (-7.3, 11.3)	-5.6 (-14.5, 3.3)	-7.6 (-17.1, 1.9)
6 months	-4.6 (-18.2, 8.9)	-1.9 (-14.9, 11.0)	2.7 (-10.8, 16.2)
**SF36 - Physical functioning**			
60 days	0.1 (-16.0, 16.2)	-6.5 (-20.9, 7.9)	-6.6 (-22.2, 9.0)
3 months	-4.9 (-19.0, 9.3)	-7.7 (-20.8, 5.4)	-2.8 (-16.8, 11.1)
6 months	-2.4 (-24.7, 19.9)	6.3 (-15.7, 28.2)	8.6 (-13.6, 30.9)
**SF36 - Pain**			
60 days	-3.7 (-19.8, 12.3)	0.5 (-12.9, 13.9)	4.2 (-11.4, 19.8)
3 months	-6.4 (-20.7, 7.9)	-7.3 (-20.8, 6.2)	-0.9 (-15.3, 13.6)
6 months	-8.5 (-22.8, 5.8)	0.7 (-13.0, 14.4)	9.2 (-5.0, 23.4)
**SF36 - Social functioning**			
60 days	-17.1 (-33.4, -0.7)	5.2 (-8.8, 19.1)	22.2 (5.7, 38.8)
3 months	-8.2 (-26.5, 10.1)	4.3 (-13.2, 21.8)	12.5 (-6.5, 31.5)
6 months	0.3 (-18.9, 19.6)	3.9 (-15.0, 22.8)	3.5 (-16.0, 23.1)

Forbes G, *et al. BMJ Open* 2020; 10:e030164. doi: 10.1136/bmjopen-2019-030164

Figure 2. Estimated treatment effects and 95% confidence intervals for CPAQ

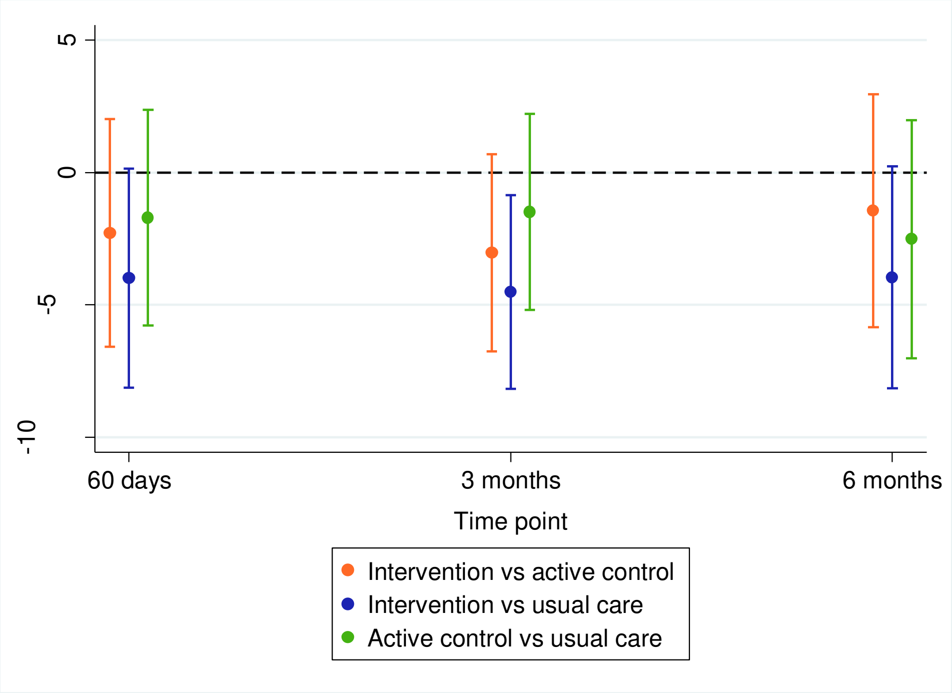

Forbes G, *et al. BMJ Open* 2020; 10:e030164. doi: 10.1136/bmjopen-2019-030164
