# Supplementary material for: MEMPHIS: a smartphone app using psychological approaches for women with chronic pelvic pain presenting to gynaecology clinics: a randomised feasibility trial
Source: BMJ Open. 2020 Mar 12;10(3):e030164. doi: 10.1136/bmjopen-2019-030164 (PMC7069270; doi:10.1136/bmjopen-2019-030164)
Supplement: Author's manuscript [file bmjopen-2019-030164.draft_revisions.pdf]

BMJ Open is committed to open peer review. As part of this commitment we make the peer review history of every article we publish publicly available.

When an article is published we post the peer reviewers' comments and the authors' responses online. We also post the versions of the paper that were used during peer review. These are the versions that the peer review comments apply to.

The versions of the paper that follow are the versions that were submitted during the peer review process. They are not the versions of record or the final published versions. They should not be cited or distributed as the published version of this manuscript.

BMJ Open is an open access journal and the full, final, typeset and author-corrected version of record of the manuscript is available on our site with no access controls, subscription charges or pay-per-view fees (http://bmjopen.bmj.com).

If you have any questions on BMJ Open's open peer review process please email info.bmjopen@bmj.com

BMJ Open

A smartphone app using psychological approaches for women with chronic pelvic pain (MEMPHIS): a randomised feasibility trial

Journal:	*BMJ Open*
Manuscript ID	bmjopen-2019-030164
Article Type:	Research
Date Submitted by the Author:	21-Mar-2019

Complete List of Authors:	Forbes, G; Queen Mary University of London, Centre for primary care and population health<br>Newton, Sian; Queen Mary University of London, Centre for Primary Care and Population Health<br>Cantalapiedra Calvete, Clara; Barts Health NHS Trust, United Kingdom, Department of Obstetrics and Gynaecology<br>Birch, Judy; Pelvic Pain Support Network<br>Dodds, Julie; Barts and The London School of Medicine and Dentistry, Women's Health Research Unit<br>Steed, Liz; Barts and The London School of Medicine and Dentistry, Centre for Primary Care and Public Health<br>Rivas, Carol; University of Southamptom, Faculty of Health Sciences<br>Khan, Khalid; Queen Mary, University of London, Centre for Primary Care and Public Health<br>Röhricht, Frank; East London NHS Foundation Trust<br>Taylor, Stephanie; Queen Mary University of London, Centre for Primary Care and Public Health<br>Kahan, Brennan; University of London, Pragmatic Clinical Trials Unit<br>Ball, ELizabeth; Queen Mary University of London - Whitechapel Campus, Yvonne Carter Building

Keywords:	chronic pain, meditation, mindfulness, mobile applications, pelvic pain, randomized controlled trial

SCHOLARONE™
Manuscripts

I, the Submitting Author has the right to grant and does grant on behalf of all authors of the Work (as defined in the below author licence), an exclusive licence and/or a non-exclusive licence for contributions from authors who are: i) UK Crown employees; ii) where BMJ has agreed a CC-BY licence shall apply, and/or iii) in accordance with the terms applicable for US Federal Government officers or employees acting as part of their official duties; on a worldwide, perpetual, irrevocable, royalty-free basis to BMJ Publishing Group Ltd ("BMJ") its licensees and where the relevant Journal is co-owned by BMJ to the co-owners of the Journal, to publish the Work in this journal and any other BMJ products and to exploit all rights, as set out in our licence.

The Submitting Author accepts and understands that any supply made under these terms is made by BMJ to the Submitting Author unless you are acting as an employee on behalf of your employer or a postgraduate student of an affiliated institution which is paying any applicable article publishing charge ("APC") for Open Access articles. Where the Submitting Author wishes to make the Work available on an Open Access basis (and intends to pay the relevant APC), the terms of reuse of such Open Access shall be governed by a Creative Commons licence – details of these licences and which Creative Commons licence will apply to this Work are set out in our licence referred to above.

Other than as permitted in any relevant BMJ Author's Self Archiving Policies, I confirm this Work has not been accepted for publication elsewhere, is not being considered for publication elsewhere and does not duplicate material already published. I confirm all authors consent to publication of this Work and authorise the granting of this licence.

For peer review only

A smartphone app using psychological approaches for women with chronic pelvic pain (MEMPHIS): a randomised feasibility trial

Gordon Forbes
Pragmatic Clinical Trials Unit, Centre for Primary Care and Population Health, Queen Mary University of London, United Kingdom

Newton, Sian
Centre for Primary Care and Population Health, Queen Mary University of London, United Kingdom

Cantalapiedra, Clara
Department of Obstetrics and Gynaecology, Barts Health NHS Trust, United Kingdom

Birch, Judy
Pelvic Pain Support Network, UK

Dodds, Julie
Women's Health Research Unit, Barts and the London School of Medicine and Dentistry, Queen Mary University of London, United Kingdom

Steed, Elizabeth
Centre for Primary Care and Population Health, Queen Mary University of London, United Kingdom

Rivas, Carol
Social Science Research Unit, University College London, United Kingdom

Khan, Khalid S.
Women's Health Research Unit, Barts and the London School of Medicine and Dentistry, Queen Mary University of London, United Kingdom

Rohricht, Frank
East London NHS Foundation Trust, United Kingdom

Taylor, Stephanie JC
Centre for Primary Care and Population Health, Queen Mary University of London, United Kingdom

Brennan C, Kahan
Pragmatic Clinical Trials Unit, Centre for Primary Care and Population Health, Queen Mary University of London, United Kingdom

Ball, Elizabeth
Department of Obstetrics and Gynaecology, Barts Health NHS Trust, United Kingdom

Corresponding Author: <Elizabeth Ball, *e.ball69@gmail.com* >

Objectives

To evaluate the feasibility of a randomised trial of a pre-existing modified smartphone app to teach mindfulness meditation intervention for women with chronic pelvic pain.

Methods

We conducted a three arm randomised feasibility trial comparing mindfulness meditation delivered by smartphone app, an active control app which delivered muscle relaxation techniques, and usual care without app delivering the interventions over a 60 day period. Women, recruited via two gynaecology clinics, were eligible if they had been experiencing organic or non-organic chronic pelvic pain for six months or more. Outcomes included length of recruitment, follow up rates, level of adherence to the app interventions, and clinical outcomes measured at baseline, two, three and six months.

Results

The target sample size of 90 women was reached after 145 days of recruitment. Adherence to the app interventions was extremely low (mean 1.8 days mindfulness meditation group, mean 7.0 days active control). No women in the mindfulness meditation group and 2 (7%) used the app on 22 or more days during the intervention period. Follow-up rates were adequate, with 57 (63%) women completing 6-month follow-up, and 75 (83%) women completing at least one post-randomisation follow-up. The 95% confidence intervals for clinical outcomes generally ruled out any meaningful clinical benefits from the mindfulness meditation app; for example, the estimated mean differences in pain acceptance scores at 60 days (where higher scores are better) were -2.3 (mindfulness meditation vs. usual care, 95% CI: -6.6, 2.0) and -4.0 (mindfulness meditation vs. active control, 95% CI: -8.1, 0.1).

Conclusions

Despite high recruitment and adequate follow-up rates, demonstrating feasibility, the extremely low adherence suggests that a definitive randomised trial of mindfulness meditation app used in this study is not warranted. Future research should focus on improving patient engagement.

ClinicalTrials.gov registration: ISRCTN 10925965

Funding: This research was supported by the UK National Institute of Health Research, Research for Patient Benefit programme (RfPB PB-PG-1013-32025).

Strengths and limitations of this study

- This is a randomised feasibility study designed specifically to test whether evaluation of the intervention is viable in a full scale randomised trial
- The trial achieved target recruitment demonstrating feasibility of recruiting patients to trials of apps for women experiencing chronic pelvic pain.
- Measures of adherence to the app interventions were robust and complete as they relied on system generated data
- This trial evaluated only one app provided by a leading developer of mindfulness meditation apps

BACKGROUND

Chronic pelvic pain is defined as intermittent or constant pain in the lower abdomen or pelvis for 6 or more months, and affects more than 24% of women worldwide (1). Chronic pelvic pain has a large impact on patients and can be difficult to treat. Chronic pelvic pain has both physical and psychological contributors (2). Health outcomes can be improved by psychological and lifestyle interventions (3) but these are often not provided (4, 5) due to difficult access or service shortages.

Mindfulness mediation is characterised as a mind-body-intervention, with the potantial to help in somatisation syndromes, which can be assocoated with chronic pelvic pain (6). Mindfulness meditation depends on activating the psychological trait or state of mindfulness. This refers to an awareness that emerges by way of paying attention intentionally and non-judgementally, in the present moment, to the unfolding of the moment-by-moment experience.

Systematic reviews of randomised controlled trials have found that mindfulness meditation may have positive effects on depression, quality of life and pain symptoms in patients with chronic pain (7, 8). Small uncontrolled studies comparing pre- and post-treatment outcomes have suggested there may be a benefit in women with chronic pelvic pain (9, 10), however the effect of mindfulness meditation on chronic pelvic pain has not been examined in an randomised controlled trial before (7).

Mindfulness meditation can be resource-intensive, typically require multiple face-to-face visit over a period of weeks or months. If effective, delivery of mindfulness meditation via smartphone app to women with chronic pelvic pain could provide a new treatment option for this patient group, without requiring an increase in resources for healthcare systems. No studies have evaluated mindfulness mediation via smartphone app. We therefore conducted a randomised feasibility trial to assess the feasibility of a future full scale, multi-centre randomised trial to test effectiveness of a mindfulness meditation intervention delivered by the Headspace smartphone app (Headspace Ltd) for patients with chronic pelvic pain. Specifically, we assessed feasibility of recruitment, levels of adherence to the intervention, and estimates parameters required for the sample size calculation for a full trial. This article reports quantitative findings; qualitative findings will be published separately (11).

METHODS

Study design and participants

This three arm parallel group randomised feasibility trial was conducted at two gynecology clinics within Barts Health NHS trust. Eligible patients were aged 18 years or over, had been experiencing organic or non-organic chronic pelvic pain for six months or more, and understood simple English. Patients were excluded from the trial if they had no access to a personal computer or smartphone, or were current users of the publicly available Headspace app. Patients were recruited via pelvic pain or endometriosis clinics at participating sites as well as at other routine appointments. The study protocol has been published (12) and the final version is given in Appendix 1.

Interventions

Full details of the interventions are available in the published protocol(12). All participants received usual care, which included watch and wait, medication and/or surgery. Women in the mindfulness meditation group received access to a 60 day progressive mindfulness meditation course layering in new techniques and concepts over successive sessions delivered via the Headspace app. The active control group received access to a series of muscle relaxation sessions. These sessions were identical every day, except that their duration increased to mirror the increasing duration of the meditation content being listened to by the intervention group. Women in mindfulness mediation group and active control group were given instructions on how to install the app. No further face-to-face induction was given on how to carry out the techniques taught in the apps. To maintain blinding between the mindfulness meditation group and active control, both groups accessed their intervention via the same app, and received instructions for the same duration, delivered by the same narrator. Only the content of the instructions differed.

We chose to evaluate an existing commercial app teaching mindfulness by guided meditation (Headspace Ltd) as it is quicker and cheaper than designing an app from scratch. The Headspace app was adapted for use by chronic pelvic pain patients by augmenting the existing app with a novel module on chronic pain, which could be accessed after completing ten days of basic training in mindfulness meditation.

Randomisation and blinding

Women were randomly allocated 1:1:1 to the active intervention app, active control app, or treatment as usual using random permuted blocks (block size 27, 30, 33) without stratification using a centralised web based service with strict allocation concealment. The randomisation list was generated using the Pragmatic Clinical Trials Unit's randomisation system using a random number generator. Following randomisation, participants, recruiting staff, and researchers conducting follow-up interviews were not

blinded to whether allocation was to the treatment as usual group or to one of the app groups (mindfulness meditation or active control); however, for allocation to an app group they were blinded to which specific app group this was (mindfulness meditation or active control). The trial statisticians remained blinded to allocation until the statistical analysis plan had been signed off, all data collection was completed, and the dataset was finalised.

Data collection

Data on patient adherence to the app was collected by Headspace Ltd. Data collection was performed automatically by the app and recorded every time a participant completed more than 90% of a session with the app. No data was collected on sessions that were less than 90% complete. Clinical outcome measures were collected in person at baseline prior to randomisation and via postal questionnaires or telephone at 2, 3 and 6 months post-randomisation. App satisfaction and usability questionnaires were collected via postal questionnaires or telephone. Shopping vouchers (£5), text reminders and phone calls were introduced to improve follow up rates three months after recruitment began: shopping vouchers were sent in the post with each follow up questionnaire; participants were sent text reminders and up to three attempts were made to contact participants by phone if questionnaire responses were not received within 10 days.

Outcomes

Feasibility outcomes were: time to recruit 90 patients to the study; standard deviation of chronic pain acceptance questionnaire (CPAQ-8) (13) (as this was likely to be the primary outcome for a future full-scale trial); proportion of participants completing a follow-up questionnaire at 6 months post randomisation; and proportion of participants not returning a follow up questionnaire by post but who answered a telephone questionnaire at 6 months. App usability was measured using the system usability scale (14) and a purpose made, non-validated questionnaire developed from PPI discussion. Adherence to the app interventions were measured in the following ways:

(a) number of days a patient has used the app within 60 days of randomisation;

(b) Number of weeks a patient has used the app on three or more days within the first eight weeks from randomisation;

(c) whether the patient has used the app on at least 22 days within 60 days of randomisation (binary outcome);

(d) whether the patient has used the app on three or more days in 6 or more weeks within the first eight weeks of randomisation (binary outcome);
(e) whether the patient has used the app on 22 or more days within the first 60 days from randomisation and used the app on three or more days in 6 or more weeks within the first eight weeks from randomisation (binary outcome).

App use was defined as having completed at least 90% of a session. This definition of app use was changed after the trial started recruiting but before any data were analysed due to a change in the way data on app use were collected by Headspace. The original definition of app use was for patients to have completed at least 50% of a session.

The following clinical outcomes were measured at baseline, 60 days, 3 months and 6 months post randomisation:
a) Pain acceptance score (measured by the chronic pain acceptance questionnaire [CPAQ-8]) (13);
b) pain related disability (chronic pain grade [CPG] – disability subscale) (15);
c) quality of life subscales (measured by the RAND short form 36 health survey [SF-36]): social functioning subscale, pain functioning subscale, and general health subscale (16);
d) the depression and anxiety subscales of the Hospital Anxiety and Depression Scale [HADS] (17)
e) mindfulness (cognitive and mindfulness - revised scale [CAMS-R]) (18);
f) self-efficacy (pain self-efficacy questionnaire [PSEQ]) (19);
g) sexual health amongst sexually active participants (sexual health outcomes in women questionnaire [SHOW-Q]) (20);
h) sexual health pelvic problem interference score (SHOW-Q pelvic problem subscale) (20);
i) an individualised outcome (Measure yourself medical outcome profile [MYMOP]) (21).

No primary outcome was specified for this study because this was a feasibility study, however it was anticipated that chronic pain acceptance would be the primary outcome for any future study assessing effectiveness.

Statistical analysis

A sample size of 90 participants was chosen as it would provide a reliable estimate for the standard deviation of the primary clinical outcome (likely to be pain acceptance) (22, 23), which could be used to inform the sample size calculation of a subsequent full-scale trial.

Feasibility outcomes and baseline data were summarised using descriptive statistics. Clinical outcomes were analysed using a linear mixed-effects models with outcome measurement (at three follow-up time points) as the dependent variable and an unstructured correlation matrix for the residuals (24). The model included fixed effects for time, treatment arm, time-by-treatment interactions and baseline measure of the outcome (25). Analysis was by intention-to-treat; all patients with an observed outcome for at least one of the three follow-up time points were included in the analysis (26), and were analysed according to their randomised group. Missing baseline clinical measures were handled using mean imputation (27). See appendix 2 for a full statistical analysis plan.

Patient and Public Involvement (PPI).

The study design and intervention was discussed with a PPI group formed of 15 women who attended the recruiting clinics. A basic version of the app by Headspace Ltd. was made available to the group for testing. A patient sat on the TMG, who bought their own experience and acted as a representative for a charity supporting those with CPP.

Ethics was granted by Camden and Kings Cross Research Ethics Committee on 1st February 2016.

RESULTS

Feasibility Outcomes

Ninety women were recruited to the trial in 145 days between May 2016 and September 2016. A CONSORT diagram is shown in figure 1, Follow up at 6 months was 68% in the mindfulness meditation group, 53% in the active control group and 69% in the usual care group. Follow up rates by method of follow up (phone or questionnaire) and at different time, points are given in appendix 3.

App use was low in both groups, but was higher in the active control group than the intervention group (mean 1.8 days intervention vs 7.0 active control – table 2). Few women used the app on more than 22 days across the entire 6 month follow up period (0 intervention vs 2 active control). Adherence to the app intervention was low or entirely absent across all other measures of app use (table 2). Daily app use

within 60 days of randomisation is summarised in figure 2. The results from the app usability questionnaire are shown in appendix 3.

Clinical outcomes

Baseline characteristics are shown in Table 1. We included 27 (87%) women from the intervention group, 23 (77%) from the active control group and 25 (86%) from the usual care group in the analysis of pain acceptance score. The 95% confidence intervals for CPAQ (figure 3) exclude a clinically meaningful effect of the intervention compared to either the active control group, or usual care group at any time point (higher CPAQ corresponds to better outcomes). The results for other clinical outcomes are consistent with no effect of the intervention (full results of clinical outcomes are shown in appendix 3).

DISCUSSION

This trial shows that it is feasible to recruit women to a trial of a mindfulness meditation app. Follow up rates were adequate, and including data across all time points meant that a relatively a high proportion of participants could be included in the analysis. This study provides estimates to inform sample size calculations for future research.

Most participants either did not complete any sessions on the apps or used them extremely infrequently. The analyses of clinical outcomes are consistent with no differences in health outcome between the three study arms. An effective intervention requires both engagement from those receiving it and the ability to change the targeted clinical outcome (28). As engagement with the mindfulness meditation app evaluated in this study was very low it is unlikely it would be an effective intervention in the routine clinical setting.

An important lesson from this trial for future researchers was that intermediate follow up points allowed for more participants to be included in the analysis of clinical outcomes than were followed up at the final time point. This demonstrates that utilising intermediate follow up time points may help to minimise potential bias from missing data in trials.

Strengths of this study include randomisation of participants, which eliminates bias inherent in other designs such as before-after studies. We also blinded patients, recruiters, and data collectors to which app group patients were allocated to. We used system generated app data and therefore were able to obtain complete adherence data for all participants. One drawback to this method of data collection was that sessions of the app were only recorded as being complete if a participant listened to 90% of the

session. This means this study may have underestimated app use if participants were only partially completing sessions. Levels of app use were so low however that this is unlikely to have had a material impact on the study's results. A second limitation is that recruitment was limited to two hospitals in one area of London, this may limit the generalisability of the results to settings where there is very high engagement with smartphone apps.

In conclusion, this study had high recruitment and adequate follow-up rates, demonstrating that it is feasible to conduct randomised trials in this patient population. However, due to extremely low adherence, further randomised trials to evaluate the benefit of the Headspace mindfulness meditation app for women with chronic pelvic pain are not warranted at this time.

Data availability statements

Anonymised participant data is available upon reasonable request. Please contact pctu-data-sharing@qmul.ac.uk with any data sharing requests.

References

1. Latthe P, Latthe M, Say L, Gulmezoglu M, Khan KS. WHO systematic review of prevalence of chronic pelvic pain: a neglected reproductive health morbidity. BMC Public Health. 2006;6:177.
2. Moore SJ KS. Green Top Guideline No 41: the initial management of Chronic Pelvic Pain. In: Gynaecologists TrcoOa, editor. 2012.
3. Peters AA, van Dorst E, Jellis B, van Zuuren E, Hermans J, Trimbos JB. A randomized clinical trial to compare two different approaches in women with chronic pelvic pain. Obstet Gynecol. 1991;77(5):740-4.
4. Romao APMS, Gorayeb R, Romao GS, Poli-Neto OB, dos Reis FJC, Rosa-e-Silva JC, et al. High levels of anxiety and depression have a negative effect on quality of life of women with chronic pelvic pain. Int J Clin Pract. 2009;63(5):707-11.
5. Stones RW, Price C. Health services for women with chronic pelvic pain. J R Soc Med. 2002;95(11):531-5.
6. Wahbeh H, Elsas SM, Oken BS. Mind-body interventions: applications in neurology. Neurology. 2008;70(24):2321-8.
7. Ball EF, Sharizan ENSM, Franklin G, Rogozinska E. Does mindfulness meditation improve chronic pain? A systematic review. Curr Opin Obstet Gynecol. 2017;29(6):359-66.
8. Hilton L, Hempel S, Ewing BA, Apaydin E, Xenakis L, Newberry S, et al. Mindfulness Meditation for Chronic Pain: Systematic Review and Meta-analysis. Ann Behav Med. 2017;51(2):199-213.
9. Kold M, Hansen T, Vedsted-Hansen H, Forman A. Mindfulness-based psychological intervention for coping with pain in endometriosis. Nordic Psychology. 2012;64(1):2-16.
10. Fox SD, Flynn E, Allen RH. Mindfulness Meditation for Women with Chronic Pelvic Pain A Pilot Study. J Reprod Med. 2011;56(3-4):158-62.
11. Ball E, Newton S, Kahan BC, Forbes G, Rohricht F, Steed E, et al. Using a Mindfulness App for women with chronic pelvic pain: Qualitative data of user experience and lessons learnt Submitted. 2018.
12. Ball E, Newton S, Kahan BC, Forbes G, Wright N, Calvete CC, et al. Smartphone App Using Mindfulness Meditation for Women With Chronic Pelvic Pain (MEMPHIS): Protocol for a Randomized Feasibility Trial. Jmir Research Protocols. 2018;7(1).
13. Fish RA, McGuire B, Hogan M, Morrison TG, Stewart I. Validation of the Chronic Pain Acceptance Questionnaire (CPAQ) in an Internet sample and development and preliminary validation of the CPAQ-8. Pain. 2010;149(3):435-43.
14. Brooke J. SUS-A quick and dirty usability scale. Usability evaluation in industry. 1996;189(194):4-7.
15. Von Korff M, Ormel J, Keefe FJ, Dworkin SF. Grading the severity of chronic pain. Pain. 1992;50(2):133-49.
16. Ware JE, Sherbourne CD. The Mos 36-Item Short-Form Health Survey (Sf-36) .1. Conceptual-Framework and Item Selection. Med Care. 1992;30(6):473-83.
17. Zigmond AS, Snaith RP. The Hospital Anxiety and Depression Scale. Acta Psychiatr Scand. 1983;67(6):361-70.
18. Feldman G, Hayes A, Kumar S, Greeson J, Laurenceau JP. Mindfulness and emotion regulation: The development and initial validation of the Cognitive and Affective Mindfulness Scale-Revised (CAMS-R). Journal of Psychopathology and Behavioral Assessment. 2007;29(3):177-90.
19. Nicholas MK. The pain self-efficacy questionnaire: Taking pain into account. European Journal of Pain. 2007;11(2):153-63.

20. Learman LA, Huang AJ, Nakagawa S, Gregorich SE, Kuppermann M. Development and validation of a sexual functioning measure for use in diverse women's health outcome studies. Am J Obstet Gynecol. 2008;198(6).
21. Paterson C. Measuring outcomes in primary care: A patient generated measure, MYMOP, compared with the SF-36 health survey. Br Med J. 1996;312(7037):1016-20.
22. Lancaster GA, Dodd S, Williamson PR. Design and analysis of pilot studies: recommendations for good practice. J Eval Clin Pract. 2004;10(2):307-12.
23. Teare MD, Dimairo M, Shephard N, Hayman A, Whitehead A, Walters SJ. Sample size requirements to estimate key design parameters from external pilot randomised controlled trials: a simulation study. Trials. 2014;15.
24. Rabe-Hesketh S, Skrondal A. Multilevel and longitudinal modeling using Stata. 3rd ed. College Station, Tex.: Stata Press Publication; 2012.
25. Kahan BC, Jairath V, Dore CJ, Morris TP. The risks and rewards of covariate adjustment in randomized trials: an assessment of 12 outcomes from 8 studies. Trials. 2014;15.
26. White IR, Horton NJ, Carpenter J, Pocock SJ. Strategy for intention to treat analysis in randomised trials with missing outcome data. Bmj-British Medical Journal. 2011;342.
27. White IR, Thompson SG. Adjusting for partially missing baseline measurements in randomized trials. Stat Med. 2005;24(7):993-1007.
28. Barrera M, Castro FG. A heuristic framework for the cultural adaptation of interventions. Clinical Psychology-Science and Practice. 2006;13(4):311-6.

Tables

Table 1: Baseline demographics and medical history. Figures are mean (SD) unless stated otherwise.

	Summary measure		
	Intervention (N=31)	Active control (N=30)	Usual care (N=29)
**Demographics**			
Age (Years)	34.8 (9.9)	35.7 (5.7)	35.0 (8.6)
Body mass index (kg/m^2)	28.7 (7.0)	26.2 (5.5)	26.6 (6.3)
Living arrangements - no. (%)			
Alone	1 (3.3)	2 (7.4)	3 (11.1)
With others	29 (96.7)	25 (92.6)	24 (88.9)
Employment status - no. (%)			
Employed	19 (63.3)	18 (66.7)	19 (67.9)
Unemployed and looking for work	2 (6.7)	0 (0.0)	1 (3.6)
At school or in full time education	2 (6.7)	1 (3.7)	4 (14.3)
Unable to work due to long term sickness	4 (13.3)	5 (18.5)	1 (3.6)
Looking after your home/family	3 (10.0)	3 (11.1)	2 (7.1)
Retired from paid work	0 (0.0)	0 (0.0)	1 (3.6)
Age left full time education - no. (%)			
Age 12 or less	1 (3.3)	1 (3.8)	1 (3.6)
Age 13 to 16	9 (30.0)	4 (15.4)	3 (10.7)
Age 17 to 19	6 (20.0)	5 (19.2)	3 (10.7)
Age 20 or over	11 (36.7)	15 (57.7)	16 (57.1)
Still in education	3 (10.0)	1 (3.8)	5 (17.9)
Ethnic group - no. (%)			
White	10 (35.7)	10 (43.5)	15 (53.6)
Black	6 (21.4)	4 (17.4)	3 (10.7)
Cetral Asian	1 (3.6)	1 (4.3)	0 (0.0)
Middle Eastern	0 (0.0)	0 (0.0)	1 (3.6)
Southern Asian	8 (28.6)	7 (30.4)	3 (10.7)
Mixed	0 (0.0)	0 (0.0)	2 (7.1)
Other ethnic group	2 (7.1)	1 (4.3)	3 (10.7)
Do not wish to say	1 (3.6)	0 (0.0)	1 (3.6)
Smoker - no. (%)			
Yes	8 (27.6)	3 (12.5)	6 (21.4)
No	21 (72.4)	21 (87.5)	22 (78.6)
If yes, number of cigarettes per week	23.9 (20.3)	40.0 (20.0)	47.6 (35.6)
Drink alcohol - no. (%)			
Yes	10 (34.5)	9 (36.0)	15 (55.6)
No	19 (65.5)	16 (64.0)	12 (44.4)
If yes, number of units per week	5.7 (5.3)	8.3 (4.7)	7.7 (7.2)
**Baseline medical history**			
Duration of pain - no. (%)			
0 to 6 months	2 (6.7)	0 (0.0)	0 (0.0)
7 to 12 months	2 (6.7)	4 (14.8)	2 (7.1)
1 to 2 years	3 (10.0)	5 (18.5)	5 (17.9)
3 to 5 years	13 (43.3)	7 (25.9)	6 (21.4)
6 to 10 years	4 (13.3)	4 (14.8)	3 (10.7)
More than 10 years	6 (20.0)	7 (25.9)	12 (42.9)
Pain over the past week (scale of 0 to 10)	6.9 (2.3)	5.8 (2.8)	6.8 (2.3)

Table 2: App use
Figures are mean (SD) unless stated otherwise.

	**Intervention (N=31)**	**Active control (N=28)***
Number of days a patient has used the app (within 60 days of randomisation)	1.8 (4.3)	7.0 (10.5)
Number of weeks a patient has used the app on three or more days (within the first eight weeks from randomisation)	0.3 (0.8)	1.0 (1.6)
Used the app on 22 or more days within the first 60 days from randomisation - no. (%)	0 (0.0)	2 (7.1)
Used the app on three or more days in 6 or more weeks (within the first eight weeks from randomisation) - no. (%)	0 (0.0)	0 (0.0)
Used the app on 22 or more days within the first 60 days AND used the app on three or more days in 6 or more weeks within the first eight weeks from randomisation - no. (%)	0 (0.0)	0 (0.0)

*2 participants in the active control group withdrew permission for their data to be used and are excluded from this analysis.

Appendix 1: Final study protocol

Appendix 2: Statistical analysis plan

Appendix 3: Supplementary tables

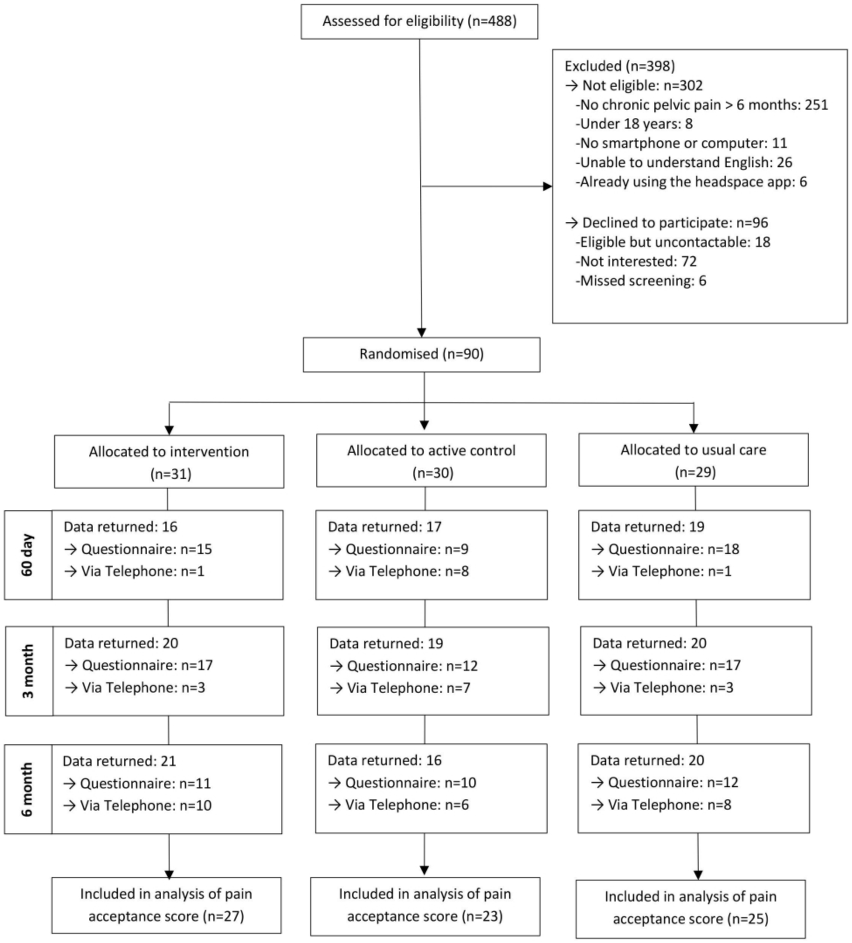

Figure 1: CONSORT diagram

592x837mm (96 x 96 DPI)

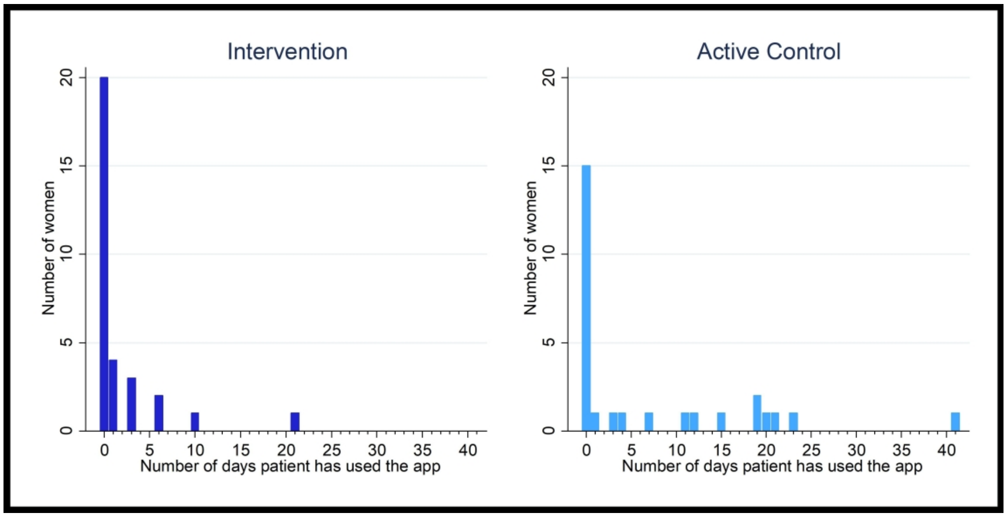

Figure 2: Daily app use (defined as completing >90% of a session) within 60 days of randomisation in the intervention and active control groups.

749x380mm (144 x 144 DPI)

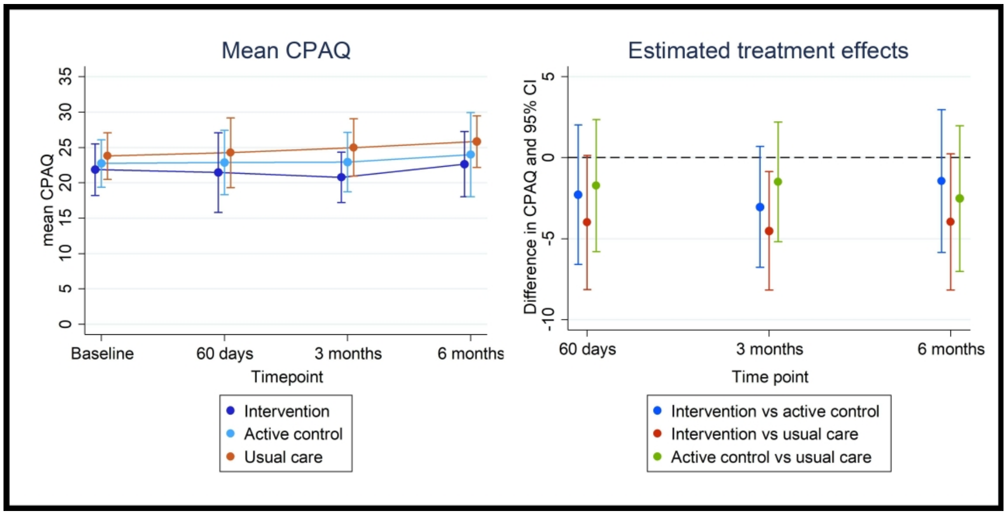

Figure 3: Mean (95% CI) chronic pain acceptance score (CPAQ) and estimated treatment effect (95% CI) at each follow-up time point. (CPAQ). Higher scores indicate better health outcomes.

854x433mm (144 x 144 DPI)

A smartphone app using psychological approaches for women with chronic pelvic pain (MEMPHIS): a randomised feasibility trial

**Short Title/Acronym**	MEMPHIS
**Sponsor**	Barts Health NHS Trust<br><br>Contact person of the above sponsor organisations is:<br><br>Dr Sally Burtles<br>Director of Research Services &<br>Business Development<br>Joint Research Management Office<br>5 Walden Street<br>London<br>E1 2EF<br>Phone: 020 7882 7260<br>Email: sponsorsrep@bartshealth.nhs.uk
**REC Reference**	15/LO/1967
**Chief Investigator**	**Miss Elizabeth Ball**<br>Women and Children Department<br>Royal London Hospital<br>Barts Health NHS Trust<br>elizabeth.ball@bartshealth.nhs.uk<br>eball69@gmail.com

For peer review only - http://bmjopen.bmj.com/site/about/guidelines.xhtml

Pragmatic Clinical Trials Unit

Blizard Institute, Centre for Primary Care and Public Health

Bart's and The London School Medicine and Dentistry

Queen Mary University of London

Yvonne Carter Building, 58 Turner St

Whitechapel, E1 2AB

PCTU Senior Trial statistician:

Mr Brennan Kahan

+44 207 882 2483

b.kahan@qmul.ac.uk

PCTU Junior Trial Statistician

Mr Neil Wright

+44 207 882 6564

n.wright@qmul.ac.uk

PCTU Data Manager:

Mr Mike Waring

+44 207 882 6914

m.r.waring@qmul.ac.uk

PCTU QA Manager:

Ms Anitha Manivannan

+44 207 882 6312

a.manivannan@qmul.ac.uk

Protocol development

Miss Elizabeth Ball

Chief Investigator

For peer review only - http://bmjopen.bmj.com/site/about/guidelines.xhtml

Barts and The London
School of Medicine and Dentistry

elizabeth.ball@bartshealth.nhs.uk

eball69@gmail.com

Dr Julie Dodds

Senior Research Manager

j.dodds@qmul.ac.uk

Mr Brennan Kahan

PCTU Senior Trial Statistician

b.kahan@qmul.ac.uk

Mr Neil Wright

PCTU Junior Trial Statistician

n.wright@qmul.ac.uk

Dr Carol Rivas

Senior Research Fellow

C.A.Rivas@soton.ac.uk

Miss Ewelina Rogozinska

Co-investigator

e.a.rogozinska@qmul.ac.uk

PCTU QA Manager:

Ms Anitha Manivannan

+44 207 882 6312

a.manivannan@qmul.ac.uk

For peer review only - http://bmjopen.bmj.com/site/about/guidelines.xhtml

Barts and The London
School of Medicine and Dentistry

CONTENTS PAGE

1.GLOSSARY OF TERMS AND ABBREVIATIONS	7
2.SIGNATURE PAGES	8
3.SUMMARY/SYNOPSIS	9
4.INTRODUCTION	11
4.1. Background	11
4.2. Effect of mindfulness meditation in chronic pain patients	12
4.3. On-going studies	12
4.4. Implications for the further development of clinical or public health practice	13
4.5. Potential impact on local policy making and improvement in service delivery	14
5.TRIAL OBJECTIVES	14
5.1. Aims and Objectives	14
5.2. Feasibility outcomes	15
5.3. App satisfaction questionnaires	16
5.4. Clinical Outcomes	17
6.METHODOLOGY	18
6.1. Inclusion Criteria	18
6.2. Exclusion Criteria	18
6.3. Study Design	19
6.4. Study Scheme Diagram	20
7. STUDY PROCEDURES	20
7.1.Informed Consent Procedures	20
7.2. Screening and enrolment	21
7.3. Randomisation Procedures	22
7.4 Blinding	22
7.5. Planned interventions	23
7.6. Concomitant Medications	24
7.7. Reasons for non progression to full trial	24

For peer review only - http://bmjopen.bmj.com/site/about/guidelines.xhtml

Barts and The London
School of Medicine and Dentistry

7.8. Key risks to delivering this research and contingencies 24
7.9. Procedure for Collecting Data 25
7.10. Including Case Report Forms (CRFs) and storage 25
7.11. Follow-up Procedures 25
7.12. Subject withdrawal (including data collection / retention for withdrawn participants) 25
7.13. Continued app use after trial period and app use by treatment as usual group 26
7.14. Schedule of Assessment 27
7.15. Criteria for Early Termination of the study 28
7.16. End of Study Definition 28
8. STATISTICAL CONSIDERATIONS 28
8.1. Sample Size 28
8.2. Statistical Analysis 28
9. ETHICS 30
10. SAFETY CONSIDERATIONS 31
11. DATA HANDLING AND RECORD KEEPING 31
11.1 Confidentiality 31
11.2. Required Study Documents 32
11.3. Record Retention and Archiving 33
12. PRODUCTS, DEVICES, TECHNIQUES AND TOOLS 33
12.1. Devices 33
12.2. Techniques and intervention 34
13. SAFETY REPORTING 35
13.1. Adverse Events (AE) 35
13.2. Serious Adverse Event (SAE) 35
13.3. Urgent Safety Measures 36
13.4. Annual Safety Reporting 36
13.5. Overview of the Safety Reporting Responsibilities 37
14. MONITORING & AUDITING 37
14.1. Auditing 37
14.2. Summary Monitoring Plan 37

For peer review only - http://bmjopen.bmj.com/site/about/guidelines.xhtml

Barts and The London
School of Medicine and Dentistry

14.3. Compliance 38
14.4. Non-Compliance 38
15. TRIAL COMMITTEES 39
15.1. Trial Steering Committee (TSC) 38
15.2. Trial Management Group (TMG) 39
15.3. Data Monitoring Committee (DMC) 39
16. FINANCE AND FUNDING 39
17. INDEMNITY 39
18. DISSEMINATION OF RESEARCH FINDINGS 40
REFERENCES 42
APPENDICES 46

For peer review only - http://bmjopen.bmj.com/site/about/guidelines.xhtml

Barts and The London
School of Medicine and Dentistry

1. GLOSSARY of Terms and Abbreviations

AE	Adverse Event
CI	Chief Investigator
CPP	Chronic Pelvic Pain
CRF	Case Report Form
DMC	Data Monitoring Committee
GCP	Good Clinical Practice
HCP	Health Care Professional
ICF	Informed Consent Form
JRMO	Joint Research Management Office
KTN	Katherine Twining Network
MHRA	Medicines and Healthcare products Regulatory Agency
NHS REC	National Health Service Research Ethics Committee
NHS R&D	National Health Service Research & Development
NPT	Normalization Process Theory
Participant	An individual who takes part in a clinical trial
PCTU	Pragmatic Clinical Trial Unit
PI	Principal Investigator
PIS	Participant Information Sheet
PSM	Patient Self-Management
QOL	Quality Of Life
QC	Quality Control
RCT	Randomised Controlled Trial
REC	Research Ethics Committee
RfPB	Research for Patients Benefit
SAE	Serious Adverse Event
SOP	Standard Operating Procedure
SUS	System Usability Scale
TAU	Treatment As Usual
TMG	Trial Management Group
TSC	Trial Steering Committee

For peer review only - http://bmjopen.bmj.com/site/about/guidelines.xhtml

2. SIGNATURE PAGES

Chief Investigator/Principal Investigator Agreement

The clinical study as detailed within this research protocol **(Version V8.0, dated 22 12 2016)**, or any subsequent amendments will be conducted in accordance with the Research Governance Framework for Health & Social Care (2005), the World Medical Association Declaration of Helsinki (1996) and the current applicable regulatory requirements and any subsequent amendments of the appropriate regulations.

Chief Investigator Name: Miss Elizabeth Ball
Chief Investigator Site: Barts and the London School of Medicine and Dentistry, Queen Mary University of London

Signature and Date: **22.12.2016**

Statistician Agreement

The clinical study as detailed within this research protocol **(Version V8.0, 22 12 2016)**, or any subsequent amendments will be conducted in accordance with the Research Governance Framework for Health & Social Care (2005), the World Medical Association Declaration of Helsinki (1996) and the current applicable regulatory requirements and any subsequent amendments of the appropriate regulations.

Statistician Name: Mr Brennan Kahan
Statistician Site: Pragmatic Clinical Trials Unit, Queen Mary University of London

Signature and Date: **22.12.2016**

For peer review only - http://bmjopen.bmj.com/site/about/guidelines.xhtml

Barts and The London
School of Medicine and Dentistry

3. SUMMARY/SYNOPSIS

**Short Title**	MEMPHIS
**Methodology**	A randomised feasibility trial
**Research Sites**	This trial will be conducted at the Royal London and Whipps Cross Hospitals
**Objectives/Aims**	The overall aim is to assess the feasibility of implementing a trial of a mindfulness meditation intervention delivered by a mobile phone app for patients with chronic pelvic pain (CPP). The primary objectives are:<br>1) To provide feasibility data for a large multicentre RCT aimed at rigorously testing mindfulness meditation in CPP<br>2) To determine whether this app can be seamlessly integrated into clinical practice, especially CPP pathways
**Number of Participants/Patients**	90 women with CPP will be recruited and each randomised into one of the three trial groups (meditation app, progressive muscle relaxation or no app).
**Main Inclusion Criteria**	To be eligible for the MEMPHIS study, the women must:<br>● Be age 18 or over<br>● Have either organic or non-organic chronic pelvic pain lasting for 6 months or more<br>● Have access to a personal computer or smartphone.<br>● Understand simple spoken English
**Statistical Methodology and Analysis (if applicable)**	Feasibility outcomes will be summarised using descriptive statistics. Clinical outcomes will be analysed using linear mixed-effects models, and results will be presented as a difference in means and a 95% confidence interval.

Barts and The London
School of Medicine and Dentistry

	Usability and integration into clinical practice will be explored in focus groups or via telephone interviews with participants.<br>Some participants will be asked to elaborate about app satisfaction and also on clinical outcomes. Results will be analysed using content analysis including both thematic and text word analysis.
**Proposed Start Date**	November 2015
**Proposed End Date**	August 2017
**Study Duration**	22 months

For peer review only - http://bmjopen.bmj.com/site/about/guidelines.xhtml

Barts and The London
School of Medicine and Dentistry

4. INTRODUCTION

4.1. Background

Chronic pelvic pain (CPP) is defined as intermittent or constant pain in the lower abdomen or pelvis of a woman for at least 6 months, not exclusively associated with menstruation, intercourse and not associated with pregnancy [1].

It affects up to 24% women worldwide [2], accounts for 20% of UK gynaecological clinic referrals [3], and has a considerable impact on patients' quality of life and their income. CPP costs the NHS € 3.3bn per year [4]. Despite costly interventions, CPP is often resistant to surgical and medical treatment. Multifactorial psychological and somatic causes require a multidimensional approach, which is not routinely offered in gynaecology clinics [5]. Evidence from randomised controlled trials (RCTs) suggests that psychological interventions may be superior to primary surgery [6]. Although psychological treatment is provided across the NHS, mostly in the context of the primary care programme *Improving Access to Psychological Therapies* there are problems with capacity, waiting times, and the overall number of patients being able to access services. Alternatively, patient self-management (PSM) is now recognised as a tool empowering patients to cope better with their condition [7]. Mindfulness meditation is a potentially valuable PSM tool in CPP. We conducted a systematic search of literature (07/2013, updated 12/2013) and found no RCTs of mindfulness meditation in CPP. However, we identified two small, non-randomised pilot trials investigating the effect of mindfulness meditation on pain (one in women with CPP and one in women with endometriosis) both of which showed promising results [8,9].

Because we identified no RCTs on mindfulness meditation in CPP in our systematic review, we included other chronic pain conditions which may have a similar patho-mechanism to pelvic pain, such as back pain, headache, fibromyalgia and diabetic neuropathy. We assume that any benefits of mindfulness meditation in these conditions may also be seen in CPP.

We found previous systematic reviews in these conditions had a number of limitations, such as not reporting effect sizes [10-12].

For peer review only - http://bmjopen.bmj.com/site/about/guidelines.xhtml

Barts and The London
School of Medicine and Dentistry

Our systematic review conducted in lines with current standards [13] identified 472 relevant citations. Nine RCTs met fully the review's inclusion criteria [14,15,16-22]. Most studies were of moderate quality; but sample sizes were generally small (from 65 women for quality of life in mental health domain to 259 women for depression).

4.2. Effect of Mindfulness based meditation in chronic pain patients

Our results showed Mindfulness based meditation reduced depression levels in chronic pain patients (standardised mean difference (SMD) -0.28; 95% CI -0.53, -0.03; p = 0.03)). Patients who received Mindfulness meditation tended to cope better with anxiety (SMD -0.16, 95% CI -0.47, 0.15) and affective pain (the emotional reaction to pain) (SMD -0.13, 95% CI -0.42, 0.16). Women in the intervention arm had also higher Quality of life (QOL) scores (especially the mental health component SMD 0.65, 95% CI -0.27, 1.58) and higher pain acceptance (SMD 0.53, 95% CI -0.13, 1.19); although these results were not statistically significant. Only one of the included studies reported the important measure of pain acceptance.

Currently Mindfulness-based therapy is creating lively research interest. Two recent systematic reviews report positive effects on somatisation disorders [23] and psychological stress [24].

4.3. On-going studies

Although there are currently no on-going studies of Mindfulness in patients with CPP that we are aware of, there are other NIHR funded studies with overlapping themes**.**

Self help in CPP

The RFPB-funded study SUPPORT, which is currently in follow- up (MREC 10/H1005/24), is investigating an evidence-based self-care guidance in general practice for women with CPP. GPs received training to use the guidance in their consultations. Women were randomised to either receive the facilitated self-care guide or usual care. Results from SUPPORT will provide valuable information on how best to integrate a new patient self-help intervention into an existing patient pathway.

For peer review only - http://bmjopen.bmj.com/site/about/guidelines.xhtml

Interactive mobile phone application to modify patient behaviour

The recently closed RFPB-funded feasibility study STARFISH (MREC 12/WS/0309) investigated the acceptability of a smartphone app that encourages stroke patients to become more physically active. The number of steps taken per day by the individual is monitored. Patients work in small groups and different goals can be set for different individuals in the group, along with goals for the whole group. It will be interesting to compare the reported obstacles and facilitators to using the app with MEMPHIS.

Web-based delivery of an intervention

Of particular interest, due to the similarities in study design to MEMPHIS, is a recently closed pilot study, MIMS (UKCRN ID 13105) that investigated adjustment to multiple sclerosis.

In MIMS, meditation teaching was delivered by videoconference. Web-based delivery has also been explored and shown to be feasible for reducing stress, anxiety and depression [25]; both options are lacking the flexibility of a smartphone app, which we are proposing.

4.4. Implications for the further development of clinical or public health practice

Our co-investigator Judy Birch is closely involved with the committee that produces national guidelines for CPP patient care pathways, which she helps to develop [26]. If the app were proven to be effective in a phase III trial, it would be possible for it to be incorporated in this pathway.

One outcome measure of MEMPHIS is to determine whether this app can be integrated into clinical practice, especially CPP pathways. If this is the case there would be benefit from studying how to extend the app to other pain conditions, such as headache, back pain and irritable bowel syndrome, in which face-to face delivered mindfulness meditation has had positive effects [23].

For peer review only - http://bmjopen.bmj.com/site/about/guidelines.xhtml

If this app is shown to be effective in a phase III trial, we will collaborate closely with Headspace, our local Health and Education Cluster and Queen Mary to implement this app both locally and nationally.

4.5. Potential impact on local policy making and improvement in service delivery

Chronic pelvic pain patients would benefit from multiple treatment approaches [6] but currently most gynaecological departments only offer medical and/or surgical treatment [5]. Although psychological treatment is provided across the NHS, mostly in the context of the primary care programme *Improving Access to Psychological Therapies* there are problems with capacity, waiting times, and the overall number of patients being able to access services. If the app is proven to be useful in a phase III RCT this gap could be filled, without having to employ more psychologists, because the interventions would be largely app delivered. Locally this would help our concerns about access to psychological treatment for CPP. Given the ubiquity of the app, greater compliance with treatment and less wastage from patients not attending appointments is expected. The use of the app in local primary, secondary and tertiary care settings would be introduced in collaboration with GP commissioning groups through local guidelines and protocols.

5. TRIAL OBJECTIVES

5.1. Aims and Objectives

The overall aim is to assess the feasibility of implementing a trial of a mindfulness meditation intervention delivered by a mobile phone app for patients with chronic pelvic pain (CPP). The primary objectives are:

- To provide feasibility data for a large multicentre RCT aimed at rigorously testing Mindfulness meditation in patients with CPP. The full-scale trial will assess the effectiveness of the mindfulness meditation app in patients with chronic pelvic pain in a national multicentre RCT

For peer review only - http://bmjopen.bmj.com/site/about/guidelines.xhtml

- To determine whether this app can be seamlessly integrated into clinical practice, especially CPP pathways. In cooperation with the Pelvic Pain Support Network, which is instrumental in the initiative on implementing nationwide pathways for patients with CPP, we will review the data on feasibility, especially the patient feedback and process analysis to answer this question to find out if the app, if it has been shown to be effective could be incorporated straight away into a national clinical pathway for CPP patients

5.2. Feasibility outcomes

5.2.1. Feasibility outcomes collected from participants

- Duration of recruitment (measured from the day recruitment opens until the day the 90th patient is randomised).
- Estimates to be used for the sample size calculation of the phase III RCT (the estimated SD for pain acceptance, and the dropout rate).
- Patient adherence to app use will be measured by the following outcomes:
    - Number of days (within the first 60 days from randomisation) a patient has used the app (with app use defined as having completed at least 90% of a session).
    - Whether the patient has used the app on 22 or more days within the first 60 days from randomisation.
    - Number of weeks (within the first eight weeks from randomisation) a patient has used the app on three or more days.
    - Whether the patient has used the app on three or more days in 6 or more weeks (within the first eight weeks from randomisation).
    - Whether the patient has used the app on 22 or more days within the first 60 days from randomisation, AND used the app on three or more days in 6 or more weeks within the first eight weeks from randomisation.
- Reasons for patient non-adherence to app use.

For peer review only - http://bmjopen.bmj.com/site/about/guidelines.xhtml

Barts and The London
School of Medicine and Dentistry

5.2.2. Feasibility outcomes collected from participant focus groups

- Usability and integration into clinical practice will be explored in two focus groups post-intervention with approximately 15 app participants, who have completed the 60 day follow up. Alternatively, participants unable to attend focus groups will be given the chance to answer a questionnaire over the phone with a research nurse.
- Discussions will be recorded and literal themes on integration and usability will be evaluated for in depth information. This information will be considered as well as adherence to the app as an indirect measure of acceptability. In cooperation with the Pelvic Pain Support Network, which is instrumental in the initiative on implementing nationwide pathways for patients with CPP, we will review the data on feasibility, especially the patient feedback and process analysis to answer this question to find out if the app, If it has been shown to be effective could be incorporated straight away into a national clinical pathway for CPP patients.
- We will determine primary and secondary outcomes of interest from the perspective of patients, for a full-scale trial. This will involve asking participants who were randomised to the app groups to discuss and prioritise outcomes.
- Obstacles to recruitment will also be explored.

5.2.3. Feasibility outcomes collected from health care practitioner focus groups

- A purpose made topic guide will be used to structure a focus group with service providers and based on the NPT toolkit [27] and the Diffusion of Innovations Theory [28] as a prompt for the facilitator.

The service providers will be asked to consider their role and their organisation and to suggest and discuss any issues to integration, and also – unlike conventional qualitative research focus groups – to suggest potential solutions. Discussions will be based around Diffusion of Innovations Theory, that is, we will consider:

- Relative advantage vs. existing practices

For peer review only - http://bmjopen.bmj.com/site/about/guidelines.xhtml

Barts and The London School of Medicine and Dentistry

- Compatibility with existing practices
- Simplicity and ease of integration
- Trialability and reinvention of the process
- Feedback (e.g. can clinicians see that patients benefit?)
- Peer to peer networking

We will use our findings to develop our integration approach to be further explored in the subsequent full trial.

- Obstacles to recruitment will also be explored.

5.3. Clinical outcomes

- Quality of life score, Physical Functioning subscale (as measured by the RAND Short Form (36) Health Survey (SF-36))
- Quality of life score, Social Functioning subscale (as measured by the RAND SF-36)
- Quality of life score, Pain subscale (as measured by the RAND SF-36)
- Quality of life score, General Health subscale (as measured by the RAND SF-36)
- Depression score (as measured by the Hospital Anxiety and Depression Scale (HADS))
- Anxiety score (as measured by HADS)
- Mindfulness score (as measure by the Cognitive and Mindfulness - Revised (CAMS – R) scale)
- Pain related disability score (as measured by the Chronic Pain Grade (CPG) disability subscale)
- Self efficacy score (as measured by the Pain Self-Efficacy Questionnaire (PSEQ))
- Pain acceptance score (as measured Chronic Pain Acceptance Questionnaire (CPAQ-8))
- Sexual Health Outcomes score (as measured by Sexual Health Outcomes in

For peer review only - http://bmjopen.bmj.com/site/about/guidelines.xhtml

Barts and The London
School of Medicine and Dentistry

Women Questionnaire (SHOW-Q))

- Subjective outcome score (as measured by Measure Yourself Medical Outcome Profile (MYMOP))

All clinical outcomes will be analysed at 60 days, 3 months, and 6 months post-randomisation.

6. METHODOLOGY

6.1. Inclusion Criteria

To be eligible for the MEMPHIS study, the women must meet the following criteria:

- Aged 18 or over
- Women with organic and non-organic chronic pelvic pain lasting for six months or more
- Be capable of understanding the information provided, with use of an interpreter if required and being able to understand simple English as is used in the app
- Give written informed consent

6.2. Exclusion Criteria

Patients who mee**t the following criteria are** ineligible to participate:

- No access to a Personal computer or smartphone
- Current users of the Headspace app content available to the public

6.3. Study Design

MEMPHIS is a randomised, single centre feasibility trial. All eligible women referred to the chronic pelvic pain clinics at the Royal London and Whipps Cross Hospitals (both new and existing patients) will be approached to take part in the study. A study leaflet will be given to them, providing brief information of the study and informing them that they are invited to participate. After informed consent, we will randomise

For peer review only - http://bmjopen.bmj.com/site/about/guidelines.xhtml

eligible women in a 1:1:1 ratio (30 participants in each group) to one of the three treatment groups:

Group A - "Intervention": 60 days of the app delivering mindfulness meditation content (in addition to usual care). See section 7.4 for a detailed description.

Group B - "Active control": 60 days of the app delivering progressive muscle relaxation content (in addition to usual care). See section 7.4 for a detailed description.

Group C - Treatment as usual (TAU): Usual care

Setting: NHS Tertiary care hospital

6.4. Study Scheme Diagram

Figure 1

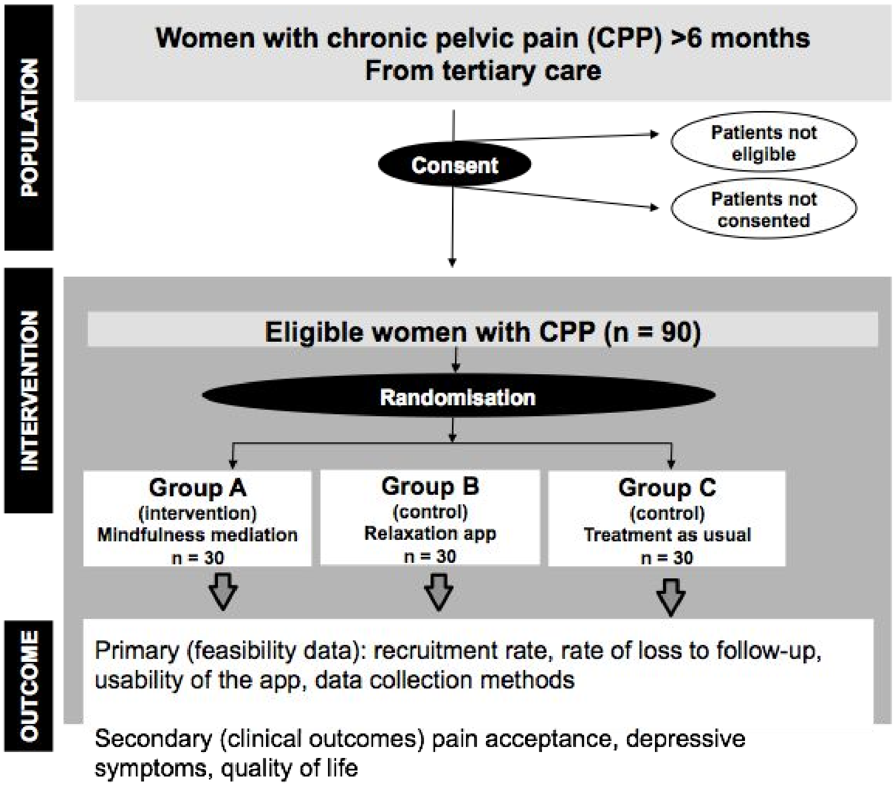

For peer review only - http://bmjopen.bmj.com/site/about/guidelines.xhtml

7. STUDY PROCEDURES

7.1. Informed Consent Procedures

Women will be made aware of the study by a health care professional and through promotional material. Potentially eligible patients will receive the PIS along with their hospital appointment invitation to ensure they have adequate time (at least 24 hours) to consider the trial. The PIS will be accompanied with a letter from the PI informing the women that they may be approached about the study at their appointment. Eligible patients who are seen in clinics other than pelvic pain and endometriosis clinics will be given the PIS and contact details for the research practitioner so they can benefit from participating in MEMPHIS should they wish so.

The PIS will be reviewed and the patient will have the opportunity to ask any questions. All eligible participants willing to consent will be asked to sign the consent form. Women will be provided with the contact details of the researcher, and informed that they have the right to withdraw their consent at any stage. Some women may be asked for permission to be contacted by a research practitioner at a later stage for enrolment if there are time constraints.

Only those on the delegation log will be able to consent for the intervention. The consenting staff will have thorough knowledge of research governance issues surrounding consent and will be fully conversant with the protocol.

If they are eligible but do not wish to consent, this will be recorded. For the full scale trial we need to understand how many eligible patients need to be approached to reach the recruitment target. We also would like to identify if eligible women opt out of the study due to a rectifiable issue.

Women who give their approval will be randomised. The investigator (or another qualified person) will explain to the potential participant that they are free to refuse any involvement within the study or alternatively withdraw their consent at any point during the study and for any reason.

For peer review only - http://bmjopen.bmj.com/site/about/guidelines.xhtml

If there is any further safety information, which may result in significant changes in the risk/benefit analysis, the PIS and Informed Consent Form (ICF) will be reviewed and updated accordingly. All participants who are actively enrolled on the study will be informed of the updated information and given a revised copy of the PIS/ICF in order to confirm their wish to continue on the study (if feasible), if it may change their willingness to participate. A copy of the consent form will be given to the participant; one will be kept in the hospital notes and the original will be placed in the Investigator Site File.

7.2. Screening and enrolment

New referrals and existing patients at the pelvic pain clinic are equally eligible. Through links with the Katherine Twining network and UCL partners we have established networks that can advertise recruitment. Based on these circumstances we are confident that we can achieve successful recruitment in the given timeframe.

Patients will be sent the Patient Information Sheet (PIS) in advance to ensure they have adequate time to consider the trial. The PIS will be accompanied with a letter from the PI informing patients that they may be approached about the study at their appointment.

At the appointment, the research practitioner will assess the women according to the inclusion/exclusion criteria detailed above and explain the nature of the intervention. The PIS will be reviewed and the patient will have the opportunity to ask any questions. All eligible participants willing to consent will be asked to sign the consent form. If a woman has not read or received the PIS before their appointment, the research team will go through the PIS with the individual in person. Women will be giving as much time as they want to consider the study before consent is taken. Women will be provided with the contact details of the researcher, and informed that they have the right to withdraw their consent at any stage.

For peer review only - http://bmjopen.bmj.com/site/about/guidelines.xhtml

Barts and The London
School of Medicine and Dentistry

7.3. Randomisation Procedures

After informed consent, patients will be randomised in a 1:1:1 ratio to one of the three treatment groups, using permuted blocks without stratification. Randomisation will be performed using a centralised internet service, hosted by the Pragmatic Clinical Trials Unit. The schedule of intervention with timeline is detailed below.

7.4. Blinding

When a participant is randomised the randomisation system will only display whether they have been allocated to an "app" treatment group (either the "Intervention" or "Active Control" group, but not which one) or the "Treatment as usual" group. If a participant is randomised to either "app" treatment group, then the randomisation system will supply an alphanumeric token which is redeemed when registering to receive the app. This will ensure that the correct content (mindfulness meditation or progressive muscle relaxation) is delivered to each participant. Therefore, the participant and recruiting staff will NOT be blinded to allocation of the "Treatment as usual" or "app" groups. However, at randomisation they will be blinded to whether allocation is to "Intervention" or "Active Control" group.

To preserve blinding of participants as much as possible, "Intervention" and "Active Control" groups will be using the same app, and hearing instructions for the same duration, delivered by the same narrator. Only the content of the instructions will differ. In addition, the Patient Information Sheet and consent form do not explicitly refer to "mindfulness meditation" or "progressive muscle relaxation".

Outcomes are collected in paper questionnaires completed by participants. The 6 month questionnaire includes a question to determine whether the participants randomised to the app have been unblinded to the "Intervention" app or "control" app. The researcher will answer a short questionnaire after recruiting each participant to determine if they have been unblinded to the "Intervention" app or "control" app, for participants randomised to an app.

Statisticians will be blinded to individual treatment allocations until required for the final analysis. If necessary, an independent statistician will perform any interim analysis which require unblinding of the data.

For peer review only - http://bmjopen.bmj.com/site/about/guidelines.xhtml

Barts and The London
School of Medicine and Dentistry

It is not anticipated that any emergency unbinding will be necessary.

7.5. Planned interventions

After eligible women have been allocated to one of the 3 groups, the participants in the Intervention and the Active Control group (progressive muscle relaxation app) will receive a face-to-face introduction to using the app. After that, the Intervention group will use the app over 60 days.

The meditation content is a structured and progressive course, layering in new techniques and concepts over successive sessions. The course was created and narrated by a former monk - Andy Puddicombe - drawing on a secularised version of the techniques he was taught over 10 years' experience in monasteries around the world.

The techniques used in the Intervention are shown in the table below. The first 30 days cover basic techniques, assuming no previous experience of meditation. The second 30 days focus specifically on the use of these techniques with respect to pain. The duration of individual sessions builds over time. Days 1-10 are 10 minutes in duration, days 11-20 are 15 minutes in duration, and days 21-60 are 20 minutes in duration.

The Active Control group will use the same app, but the app will be configured so that they will hear a series of non-meditative progressive muscle relaxation instructions, also narrated by Andy Puddicombe. These sessions will be identical every day, except that their duration will increase to mirror the increasing duration of the meditation content being listened to by the Intervention group.
In this way, both Intervention and Control groups will be using the same app, and hearing instructions for the same duration, delivered by the same narrator. Only the content of the instructions will differ.

For peer review only - http://bmjopen.bmj.com/site/about/guidelines.xhtml

Barts and The London
School of Medicine and Dentistry

Series	Techniques involved
Take 10/Foundation 1 (first 10 days)	Open monitoring, body scan, breath as anchor
Foundation 2 (days 11-20)	As above, plus intention and altruism
Foundation 3 (days 21-30)	As above, plus integration of mindfulness with daily activities
Pain series (days, 31-60)	As above, plus visualisation and enquiry (insight/Tibetan vipassana)

7.6. Concomitant Medications

Patients are able to receive any concomitant medications that they would as part of usual care.

7.7. Reasons for non progression to full trial

- Insurmountable problems with recruitment
- Extremely high rates of loss-to-follow-up
- Extremely low rates of adherence to the intervention
- Unacceptability of intervention for patients

7.8. Key risks to delivering this research and contingencies:

- Recruitment of 90 patients between May 2016 and October 2016 not achieved – regular monitoring throughout recruitment period to identify and resolve problems (e.g. open new centres/extend recruitment period)
- We will monitor regularly if patients have not downloaded apps and offer further one-to-one support
- Data collection issues will be monitored and addressed early where possible; this will inform the full-scale RCT design

For peer review only - http://bmjopen.bmj.com/site/about/guidelines.xhtml

- Issues relating to the other milestones (ethics, personnel, app availability) and deliverables will be rectified, but potentially delay the start of MEMPHIS/full-scale trial. Contamination was not thought likely by the patient group

7.9. Procedure for Collecting Data

Patients will enter the data on paper questionnaires, which will be transferred into a purpose-built electronic database.

1.) Scales for clinical outcomes
2.) App satisfaction questionnaire, which includes open comment boxes and tick-boxes based on published questionnaires [30].

As an incentive to complete and return the patient questionnaires, a £5 shopping voucher will be sent in the post with each follow up questionnaire alongside a stamped addressed envelope.
In the case that a questionnaire is not received, participants will be sent a text reminder. Non-responders will then be contacted by telephone in order to collect a smaller dataset.

7.10. Including Case Report Forms (CRFs) and storage

In line with GCP guidance we will keep the data stored for 20 years following the close of the study to allow for verification and any further data sharing e.g. individual patient data meta-analysis.

We will follow the PCTU's standard operating procedures for legacy archiving.
Queen Mary University of London will act as custodians of the data.

7.11. Follow-up Procedures

Some of the participants will be asked for permission to elaborate on the open comment boxes about app satisfaction and also on clinical outcomes in two focus

For peer review only - http://bmjopen.bmj.com/site/about/guidelines.xhtml

Barts and The London School of Medicine and Dentistry

groups to be held after the 6 month follow up point finisheswith participants asked to discuss and prioritise outcomes. Alternatively, participants unable to attend focus groups will be given the option to answer a questionnaire over the phone with a researcher.

7.12. Subject withdrawal (including data collection / retention for withdrawn participants)

A participant can be withdrawn from the trial if, in the opinion of the investigator or the care providing clinician or clinical team, it is medically necessary to do so.

With any post randomisation exclusions, the study personnel will make every effort to obtain, and record, information about the reasons for violation, any adverse events and to follow-up the women for all safety and efficacy outcomes, as appropriate. If a woman decides after randomisation she does not wish to participate any further in the MEMPHIS trial, she may withdraw herself from the trial. We will aim to document the reason for self-withdrawal. Clear distinction will be made as to whether the participant is withdrawing from trial whilst allowing further follow-up, or whether the participant refuses any follow-up. If a participant explicitly withdraws consent to have any further data recorded their decision will be respected and recorded on the final study form. All communication surrounding the withdrawal will be noted in the study records and no further data will be collected for that participant. They will be returned to the NHS standard practice for follow up care.

If a woman loses their ability to consent during participation in the trial, they will be withdrawn from the trial and no further data will be collected from the participant unless consent for this was explicitly obtained prior to the loss of capacity.

7.13. Continued app use after trial period and app use by treatment as usual group

It was decided to permit continued app use to the end of the study to reflect the situation in real life. Duration of use will be recorded through the app without using patient identifiable data.

For peer review only - http://bmjopen.bmj.com/site/about/guidelines.xhtml

Barts and The London School of Medicine and Dentistry

Consideration was given to inform patients in the 'treatment as usual' arm at the beginning that they will be able to access the meditation app at the end of the study, but this was abandoned due to concerns that this could lead to bias. Research has shown [31] that in those circumstances patients may decide to 'wait' until the end of the intervention before trying to improve, and as a consequence, they tend to improve less, leading to overestimating the effect of the intervention. It is possible that without the offer of delayed app use recruitment may be slower, which is something we would like to determine in the feasibility study. However, if after close involvement with the PPI this appears to be not acceptable to patients as compromise such as telling control patients after the end of the study that they are now allowed to use the app may be offered.

7.14. Schedule of Assessment

Health outcome measures are collected at baseline. The delivery of the intervention or control will occur for 60 days. Health outcome measures are collected immediately after the intervention at 60 days, and again at 3 and 6 months. App satisfaction/usability measures will be collected immediately after the intervention at 60 days from app participants.

The usability and clinical outcome focus groups will take place after the 6 month follow up point.

Assessment	Baseline	During intervention	60 days post randomisation	3 months post randomisation	6 months post randomisation
Questions about participants pain	V				
History of pain treatment	V				
Personal details	V				
Adherence to app use		V			
Clinical outcome questionnaires	V		V	V	V

Barts and The London
School of Medicine and Dentistry

App satisfaction questionnaires			V		
Interview/focus group with recruiters, nurses, patients, other stakeholders on usability and integration into practice					V
HCP and patient focus groups on clinical outcomes					V

For peer review only - http://bmjopen.bmj.com/site/about/guidelines.xhtml

7.15. Criteria for Early Termination of the study

The nature of the intervention and follow–up makes it unlikely that any new information will impact an individual participant. If the TSC committee, REC, CI or sponsor determine it is within the best interests of the participants or trial to terminate the study, written notification will be given to the CI. This may be due to, but not limited to; safety concerns, proof of efficacy or non-compliance/serious breaches. If the study is terminated participants will be returned to the NHS normal follow up and routine care.

7.16. End of Study Definition

When the last enrolled participant has completed follow up, the REC will be notified of the trial completion. The final study report will be completed within 12 months after the trial completion.

8. STATISTICAL CONSIDERATIONS

8.1. Sample Size

30 participants will be recruited to each of the three treatment groups, giving a total of 90 participants. As this is a feasibility study, we have not performed a sample size calculation based upon the power to detect a significant treatment effect on a clinical outcome. However, 90 participants should provide a reliable estimate for the standard deviation of the primary clinical outcome (likely to be pain acceptance) [32, 33], which can be used to inform the sample size calculation of the main trial.

8.2. Statistical Analysis

A full analysis plan will be developed and agreed prior to any analysis or unblinding of the data.

For peer review only - http://bmjopen.bmj.com/site/about/guidelines.xhtml

Barts and The London
School of Medicine and Dentistry

Baseline

Baseline variables will be presented for each treatment group as the mean (SD) or median (IQR) for continuous variables, and the number (%) for categorical variables.

Analysis of Feasibility Outcomes

Feasibility outcomes will be presented for each treatment group as the mean (SD) or median (IQR) for continuous variables, and the number (%) for categorical variables.

Duration of recruitment will be calculated as the number of days from the beginning to the end of recruitment. The number of participants recruited per month will be presented.

The proportion of patients in each treatment group who have returned data at each follow-up time point (60 days, 3 months, and 6 months post-randomisation) will be presented. Summaries of baseline variables will be presented separately for patients who have and have not returned data at each at the 6 month time point.

Adherence outcomes will be summarised separately for the intervention and active control treatment groups. Adherence outcomes will be presented as the mean (SD) or median (IQR) for continuous variables, and the number (%) for categorical variables.

An estimate of the standard deviation of pain acceptance (CPAQ) in each treatment group at each follow up time point (60 days, 3 months, and 6 months) will be presented.

Analysis of Clinical Outcomes

For each clinical outcome we will present the following information:

- The number of patients in each treatment group with an observed outcome at each follow-up time point.

For peer review only - http://bmjopen.bmj.com/site/about/guidelines.xhtml

Barts and The London School of Medicine and Dentistry

- The mean (SD) in each treatment group at each follow-up time point.
- The estimated treatment effect at each follow-up time point, with a 95% confidence interval.

Estimates of treatment effect will be presented comparing the intervention group (mindfulness meditation app) to the control (treatment as usual) group, the intervention group to the active control (progressive muscle relaxation app) group, and the active control group to the control (treatment as usual) group. Outcomes will be analysed using linear mixed-effects models to account for the correlation between patient outcomes at different follow-up time points [34], and adjusted for baseline measure of the outcome [35]. Patient data will be analysed according to the treatment group to which they were randomised (intention-to-treat). All patients with an observed outcome for at least one of the three follow-up time points (60 days, 3 months, or 6 months) will be included in the analysis [36].

Analysis of usability and integration of app

- Obstacles to recruitment will be summarised
- The integration of the app into existing and emerging patient pathways will be investigated using questionnaires developed from social contagion theory and Normalisation Process Theory (NPT) as described in section 5.3. The maximum total score using NPT is 64. The maximum total score using the Diffusion of Innovations questionnaire is 200.
  The System Usability Scale (SUS) [28] has a maximum score of 50.

9. ETHICS

The Investigator to an Independent Research Ethics Committee will submit this protocol and any subsequent amendments, along with any accompanying material provided to the participant in addition to any advertising material. Written Approval from the Committee will be obtained and subsequently submitted to the JRMO to

For peer review only - http://bmjopen.bmj.com/site/about/guidelines.xhtml

Barts and The London
School of Medicine and Dentistry

obtain Final R&D approval. The trial can only start after approval from a Research Ethics Committee and the local R&D "Sign-off" from the participating centre. If there is any further safety information, which may result in significant changes in the risk/benefit analysis, the Patient Information Sheet (PIS) and Informed Consent Form (ICF) will be amended accordingly and submitted to REC for revision and approval. All participants that are actively enrolled on the study will be informed of the updated information and given a revised copy of the PIS/ICF in order to confirm their wish to continue on the study (if feasible), if it may change their willingness to participate.

10. SAFETY CONSIDERATIONS

There are no known side effects arising from mindfulness meditation.

11. DATA HANDLING AND RECORD KEEPING

11.1. Confidentiality

Patient anonymity is protected and maintained. This applies to data collected on paper or via the headspace database.

We will ensure that patient identities are protected from any unauthorised parties. Information with regards to study patients will be kept confidential and managed in accordance with data Protection Act, NHS Caldicott Guardian, The research Governance Framework for Health and Social care and Research Ethics Committee Approval.

The trial will collect personal data and sensitive information about the participants either directly or from their clinical team. Participants will be informed about the transfer of this information to the study office and will be asked to consent to this. The data will be entered onto a secure computer database, either by trials unit staff or directly via a secure Internet connection. Any data to be processed will be anonymised. All personal information obtained for the trial will be held securely and treated as (strictly) confidential. All staff, at the hospital or the trials unit shares the

For peer review only - http://bmjopen.bmj.com/site/about/guidelines.xhtml

Barts and The London
School of Medicine and Dentistry

same duty of care to prevent unauthorised disclosure of personal information. No data that could be used to identify an individual will be published.

In relation to the data collected by Headspace the following applies:

Headspace will not collect any clinical data, but data on app usage. Details collected on the headspace database will be confidential. Details about the individual's use of Headspace tools will never be seen by or shared with anyone outside the research team and the company. Individual usage and demographic information will only be used by Headspace in accordance with the standard Headspace user terms and conditions. No data will be shared with any other organizations, unless with prior agreement, and all data is kept confidential. App usage data will be transferred to the research team via a securely encrypted file.

The Chief investigator, Miss Elizabeth Ball is the "custodian" of the data.

11.2. Required Study Documents

- A signed protocol and any subsequent amendments
- PCTU self-monitoring template for the trial team to complete on a regular basis as detailed by the Trial Monitoring section
- Current and Superseded Patient Information Sheets
- Current and Superseded Consent Forms
- Current and Superseded GP letters
- Current and Superseded Posters
- Current and Superseded CRFs
- Indemnity documentation from sponsor
- Conditions of Sponsorship from sponsor
- Conditional/Final R&D Approval
- Signed site agreements
- Ethics submissions/approvals/correspondence
- CVs and GCP certificates of CI and site staff

For peer review only - http://bmjopen.bmj.com/site/about/guidelines.xhtml

Barts and The London School of Medicine and Dentistry

- Laboratory accreditation letter, certification and normal ranges for all laboratories to be utilised in the study
- Delegation log
- Staff training log
- Identification log
- Enrolment log
- Monitoring visit log
- Correspondence relating to the trial
- SAE reporting plan for the study

11.3. Record Retention and Archiving

During the course of research, all records are the responsibility of the Chief Investigator and must be kept in secure conditions. When the trial is complete, it is a requirement of the Research Governance Framework and Trust Policy that the records are kept for a further 20 years. For trials involving Barts Health Trust patients, undertaken by Trust staff, or sponsored by Barts Health trust or QMUL, the approved repository for long-term storage of local records is the Trust Modern Records centre, which is based at 9 Prescott Street.

12. PRODUCTS, DEVICES, TECHNIQUES AND TOOLS

12.1. Devices

The Medicines and Healthcare products Regulatory Agency (MHRA) states that some apps can be classified as medical devices. [37]

However, apps with software that provides general information but does not provide personalised advice, although it may be targeted to a particular user group, is unlikely to be considered a medical device. We believe that neither the mindfulness meditation nor the progressive muscle relaxation content in the app fulfil the criteria for medical devices.

For peer review only - http://bmjopen.bmj.com/site/about/guidelines.xhtml

Barts and The London School of Medicine and Dentistry

12.2. Techniques and interventions

Intervention (mindfulness meditation content):

60 days of guided meditation content. The first 30 days cover basic techniques, assuming no previous experience of meditation. The second 30 days focus specifically on the use of these techniques with respect to pain. The duration of individual sessions builds over time. The first 10 days are each 10 minutes in duration. The next 10 days are each 15 minutes in duration. All following days are 20 minutes in duration. The minimum usage of app should be for at least 22 out of 60 days.

It was decided to permit continued app use to the end of the study to reflect the situation in real life. Duration of use will be recorded through the app without using patient identifiable data.

Control:

1) Treatment as usual (watch and wait, medication and/or surgery) to investigate if any app intervention makes a difference to wellbeing and to ascertain dropout rates for the full-scale trial in patients who perceive that they are getting no intervention

2) 60 days of progressive muscle relaxation content: This group will use the same app as the Intervention group, but the app will be configured so that they will hear a series of non-meditative progressive muscle relaxation instructions. These sessions will be identical every day, except that their duration will increase to mirror the increasing duration of the meditation content being listened to by the Intervention group (10 minutes a day for 10 days, then 15 minutes a day for 10 days, then 20 minutes a day thereafter.)

App satisfaction questionnaires

- Purpose made questionnaire (Carol Rivas)
- The System Usability Scale (SUS) [28]

For peer review only - http://bmjopen.bmj.com/site/about/guidelines.xhtml

Barts and The London
School of Medicine and Dentistry

13. SAFETY REPORTING

13.1. Adverse Events (AE)

An AE is any untoward medical occurrence in a subject to whom a medicinal product has been administered, including occurrences which are not necessarily caused by or related to that product. An AE can therefore be any unfavourable and unintended sign (including an abnormal laboratory finding), symptom or disease temporarily associated with study activities.

We do not expect SAEs related to use of the mindfulness or the progressive muscle relaxation app.

Notification and reporting Adverse Events or Reactions

If the AE is not defined as SERIOUS, the AE is recorded in the study file and the participant is followed up by the research team. The AE is documented in the participants' medical notes (where appropriate) and the CRF.

13.2. Serious Adverse Event (SAE)

A serious adverse event (SAE) is defined as an untoward occurrence that:

(a) results in death;
(b) is life-threatening;
(c) requires hospitalisation or prolongation of existing hospitalisation;
(d) results in persistent or significant disability or incapacity;
(e) consists of a congenital anomaly or birth defect; or
(f) is otherwise considered medically significant by the investigator.

An SAE occurring to a research participant should be reported to the main REC where in the opinion of the Chief Investigator the event was:

• Related – that is, it resulted from administration of any of the research procedures, and

For peer review only - http://bmjopen.bmj.com/site/about/guidelines.xhtml

Barts and The London
School of Medicine and Dentistry

• Unexpected – that is, the type of event is not listed in the protocol as an expected occurrence.

Notification and Reporting of Serious Adverse Events

Serious Adverse Event (SAEs) that are considered to be 'related' and 'unexpected' are to be reported to the sponsor within 24 hours of learning of the event and to the Main REC within 15 days in line with the required timeframe. For further guidance on this matter, please refer to NRES website and JRMO SOPs

13.3. Urgent Safety Measures

The CI may take urgent safety measures to ensure the safety and protection of the clinical trial subjects from any immediate hazard to their health and safety,. The measures should be taken immediately. In this instance, the approval of the REC prior to implementing these safety measures is not required. However, it is the responsibility of the CI to inform the sponsor and Main Research Ethics Committee (via telephone) of this event immediately.

The CI has an obligation to inform both the Main REC in writing within 3 days, in the form of a substantial amendment. The sponsor (Joint Research Management Office (JRMO)) must be sent a copy of the correspondence with regards to this matter. For further guidance on this matter, please refer to NRES website and JRMO SOPs.

13.4. Annual Safety Reporting

The CI will send the Annual Progress Report to the main REC using the NRES template (the anniversary date is the date on the MREC "favourable opinion" letter from the MREC) and to the sponsor. Please see NRES website and JRMO SOP for further information

For peer review only - http://bmjopen.bmj.com/site/about/guidelines.xhtml

Barts and The London School of Medicine and Dentistry

13. 5. Overview of the Safety Reporting responsibilities

The CI/PI has the overall pharmaco-vigilance oversight responsibility. The CI/PI has a duty to ensure that safety monitoring and reporting is conducted in accordance with the sponsor's requirements.

14. MONITORING & AUDITING

14.1. Auditing

Definition: "A systematic and independent examination of trial related activities and documents to determine whether the evaluated trial related activities were conducted, and the data were recorded, analysed and accurately reported according to the protocol, sponsor's standard operating procedures (SOPs), Good Clinical Practice (GCP), and the applicable regulatory requirement(s)."

A study may be identified for audit by any method listed below:

1. A project may be identified via the risk assessment process.
2. An individual investigator or department may request an audit.
3. A project may be identified via an allegation of research misconduct or fraud or a suspected breach of regulations.
4. Projects may be selected at random. The Department of Health states that Trusts should be auditing a minimum of 10% of all research projects.
5. Projects may be randomly selected for audit by an external organisation.

Internal audits may be conducted by a sponsor's or funder representative.

14.2. Summary Monitoring Plan

Investigators and their host Trusts will be required to permit study-related monitoring and audits to take place, providing direct access to source data and documents as requested. Trusts may also be subject to inspection by the Research and Development Manager and should do everything requested by the Chief Investigator in order to

For peer review only - http://bmjopen.bmj.com/site/about/guidelines.xhtml

prepare and contribute to any inspection or audit. Study participants will be made aware of the possibility of external audit of data they provide in the participant information sheet.

14.3. Compliance

The CI will ensure that the trial is conducted in compliance with the principles of the Declaration of Helsinki (1996), and in accordance with all applicable regulatory requirements including but not limited to the Research Governance Framework, GCP, Trust and Research Office policies and procedures and any subsequent amendments.

14.4. Non-Compliance

Definition: A noted systematic lack of both the CI and the study staff adhering to the principles of the Declaration of Helsinki (1996), applicable regulatory requirements including but not limited to the Research Governance Framework, GCP, Trust and Research Office policies and procedures and any subsequent amendments, which leads to prolonged collection of deviations, breaches or suspected fraud.

These non-compliances may be captured from a variety of different sources including monitoring visits, CRFs, communications and updates. The sponsor will maintain a log of the non-compliances to ascertain if there are any trends developing or escalating. The sponsor will assess the non-compliances and action a timeframe in which they need to be dealt with. Each action will be given a different timeframe dependent on the severity. If the actions are not dealt with accordingly, the sponsor will agree an appropriate action, including an on-site audit.

15. TRIAL COMMITTEES

15.1. Trial Steering Committee (TSC)

The TSC provides independent supervision for the trial, providing advice to the Chief and Co-Investigators and the Sponsor on all aspects of the trial and affording

For peer review only - http://bmjopen.bmj.com/site/about/guidelines.xhtml

Barts and The London
School of Medicine and Dentistry

protection for patients by ensuring the trial is conducted according to the principles of Good Clinical Practice in Clinical Trials. If the Chief and Co-Investigators are unable to resolve any concern satisfactorily, Principal Investigators, and all others associated with the trial, may write through the Trial Unit to the chairman of the TSC, drawing attention to any concerns they may have about the possibility of particular side-effects, or of particular categories of patient requiring special study, or about any other matters thought relevant.

15.2. Trial Management Group (TMG)

The trial management group will meet regularly to discuss operational issues. This will include the chief investigator, trial co-ordinator, senior research manager, statistician, data manager, QA manager and research administrator.

15.3. Data Monitoring Committee (DMC)

Based on the short duration of recruitment (expected to be 6 months) and the safety profile of the intervention, a DMC will not be used.

16. FINANCE AND FUNDING

-This study is funded by the Research for Patients Benefit national programme (RfPB).
- Headspace is donating subscriptions at no charge as part of their research initiative.

17. INDEMNITY

Queen Mary, University of London will act as a Sponsor, as defined by the Research Governance Framework for Health and Social Care (April 2005) for the project. The project will also be covered by the sponsor's insurance brokers on a "No Faults Compensation for Clinical Trials and/or Human Volunteer Studies". This policy will

For peer review only - http://bmjopen.bmj.com/site/about/guidelines.xhtml

indemnify/cover the insured in respect of their legal liabilities arising out of the insured's activities.

18. DISSEMINATION OF RESEARCH FINDINGS

The research findings of the feasibility study will be disseminated judiciously to avoid biasing the full-scale trial. In both trials we will disseminate our findings to:

1) Study participants through a dedicated website and newsletters at the end of the feasibility and full scale study, guided by our lay advisers

2) Participating health care professionals through the dedicated website and electronic newsletters

4) Professional groups via peer-reviewed journals and scientific meetings. Post-trial workshops run in collaboration with PPI group

5) Health service commissioners via the study website and an electronic newsletter

6) The wider public through local and national media and via dedicated website

7) Patients and relatives through PPI group

Applicants have links for dissemination via these organisations: Cochrane reviews, NICE, Pelvic pain support network (Judy Birch), Katherine Twining Network (KTN), BJOG (Khalid Khan), BSGE (Elizabeth Ball) Communications experts at our higher education institutions and the NIHR Collaboration for Leadership in Applied Health Research and Care North Thames will support our dissemination strategy through Twitter, Facebook and press coverage.

A particular strength of our application is our close links with:

For peer review only - http://bmjopen.bmj.com/site/about/guidelines.xhtml

1) KTN, dedicated to research and education in the UK and abroad via the East London International Women's Health Appeal, who will be able to disseminate this low cost-intervention in developing countries with high incidence of CPP [2]

2) UCL partners, whose focus is on patient-led population-focused delivery of research innovations.

For peer review only - http://bmjopen.bmj.com/site/about/guidelines.xhtml

Barts and The London
School of Medicine and Dentistry

REFERENCES

1. Royal College of Obstetricians and Gynaecologists, green top guidelines (2012) Available at: https://www.rcog.org.uk/en/guidelines-research-services/guidelines/gtg41 (Accessed: 13 May 2015)

2. Latthe, P., Latthe, M., Say, L., Gülmezoglu, M., Khan, K.S., (2006), 'WHO systematic review of prevalence of chronic pelvic pain: a neglected reproductive health entity', BMC Public Health. , 6(6), pp. 177.

3. Howard, F.M., (1993), 'The role of laparoscopy in chronic pelvic pain: promise and pitfalls', Obstetrical and Gynecological Survey, 48(6), pp. 357-387.

4. Simoens, S., et al., (2012), 'The burden of endometriosis: costs and quality of life of women with endometriosis and treated in referral centres', Human Reproduction, 27(5) pp. 1292-1299

5. Stones, R.W. and Price, C., (2002), 'Health services for women with chronic pelvic pain', Journal of the Royal Society of Medicine, 95, pp. 531-535

6. Peters, A.A., et al., (1991), 'A randomized clinical trial to compare two different approaches in women with chronic pelvic pain', Obstetrics and Gynecology, 77(5), pp. 740-4

7. Warsi, A., et al., (2004), 'Self-management education programs in chronic disease: a systematic review and methodological critique of the literature', Archives of Internal Medicine, 164(15), pp. 1641-9.

8. Fox, S.D., Flynn, E., Allen, R.H., (2011), 'Mindfulness meditation for women with chronic pelvic pain: a pilot study', The Journal of reproductive medicine, 56(3-4), pp. 158-162.

9. Kold, M., et al., (2012), 'Mindfulness-based psychological intervention for coping with pain in endometriosis', Nordic Psychology, 64(1), pp. 2-16.

10. Cramer, H., et al., (2012), 'Mindfulness-based stress reduction for low back pain. A systematic review', BMC Complementary & Alternative Medicine, 12, pp. 162.

11. Kozasa, E.H., et al., (2012), 'The effects of meditation-based interventions on the treatment of fibromyalgia', Current Pain & Headache Reports, 16(5), pp. 383-7.

12. Veehof, M.M., et al., (2011), 'Acceptance-based interventions for the treatment of chronic pain: A systematic review and meta-analysis', Pain, 152(3), pp. 533-42

For peer review only - http://bmjopen.bmj.com/site/about/guidelines.xhtml

13. Higgins, J.P.T., Green, S., (2011) 'Cochrane handbook for systematic reviews of interventions', version 5.1.0. Cochrane Collaboration. Available at: www.cochrane-handbook.org (Accessed: 13 May 2015)

14. Brown, C.A. and Jones, A.K., (2013), 'Psychobiological correlates of improved mental health in patients with musculoskeletal pain after a mindfulness-based pain management program', Clinical Journal of Pain, 29(3), pp. 233-44.

15. Carson, J., et al., (2005), 'Loving-kindness meditation for chronic low back pain: results from a pilot trial', Journal of Holistic Nursing, 23(3), pp. 287-304.

16. Morone, N.E., Greco, C.M., Weiner, D.K., (2008), 'Mindfulness meditation for the treatment of chronic low back pain in older adults: a randomized controlled pilot study', Pain, 134(3), pp. 310-9.

17. Nash-Mc Feron, D.E., (2006), 'Mindfulness in the treatment of chronic headache pain', Dissertation Abstracts International: Section B: The Sciences and Engineering, 67(5-B), pp. 2841.

18. Plews-Ogan, M., et al., (2005), 'A pilot study evaluating mindfulness-based stress reduction and massage for the management of chronic pain', Journal of General Internal Medicine, 20(12), pp. 1136-8.

19. Sagula, D. and Rice, K.G., (2004), 'The effectiveness of mindfulness training on the grieving process and emotional well-being of chronic pain patients', Journal of Clinical Psychology in Medical Settings, 11(4), pp. 333-342.

20. Schmidt, S., et al., (2010), 'Assessing a dynamical EEG pattern related to chronic pain-Results from a controlled evaluation of back pain patients as well as from an observational trial with a mindfulness based intervention', European Journal of Integrative Medicine, 2 (4), pp. 196.

21. Sephton, S.E., et al., (2007), 'Mindfulness meditation alleviates depressive symptoms in women with fibromyalgia: Results of a randomized clinical trial', Arthritis Care and Research, 57(1), pp. 77-85.

22. Teixeira, E., (2010), 'The effect of mindfulness meditation on painful diabetic peripheral neuropathy in adults older than 50 years', Holistic Nursing Practice, 24(5), pp. 277-83.

23. Lakhan, S.E. and Schofield, K.L., (2014), 'Mindfulness-Based Therapies in the Treatment of Somatization Disorders: A Systematic Review and Meta-Analysis', PLOS One, 8(8): e71834

For peer review only - http://bmjopen.bmj.com/site/about/guidelines.xhtml

24. Goyal, M., et al., (2014), ‘Meditation Programs for Psychological Stress and Well-being’, JAMA Internal Medicine., 174(3), pp. 357-368

25. Krusche, A., Cyhlarova, E., Williams, J.M., (2013), ‘Mindfulness online: an evaluation of the feasibility of a web-based mindfulness course for stress, anxiety and depression’, BMJ Open., 3(11): pp. e003498.

26. http://bps.mapofmedicine.com/evidence/bps/chronic_pelvic_pain_for_men_and_women_1.html

27. May, C., Sibley, A., Hunt, K., (2013) ’The nursing work of hospital-based clinical practice guideline implementation: an explanatory systematic review using normalisation process theory’, International Journal of Nursing studies, 51(2), pp.289-99

28. Rogers, E.M., (2003), Diffusion of Innovations, Fifth Edition, Free Press, New York, pp.221

29. Measuring Usability With The System Usability Scale (SUS), (2011) Available at: http://www.measuringu.com/sus.php, (Accessed: 13 May 2015)

30. Spook, J.E., et al., (2013), ‘Monitoring Dietary Intake and Physical Activity Electronically: Feasibility, Usability, and Ecological Validity of a Mobile-Based Ecological Momentary Assessment Tool’, Journal of Medical Internet Research, 15(9), pp. e214.

31. Cunningham, J.A., Kypros, K., McCambridge, J., (2013), ‘Exploratory randomized controlled trial evaluating the impact of a waiting list control design’, BMC Medical Research Methodology, 13(150)

32. Lancaster, G.A., Dodd, S., Williamson, PR., (2004), ‘Design and analysis of pilot studies: recommendations for good practice’, Journal of Evaluation in Clinical Practice, 10(2), pp.307-12

33. Teare, MD., Dimairo, M., Shephard, N., Hayman, A., Whitehead, A., Walters, SJ., (2014), ′Sample size requirements to estimate key design parameters from external pilot randomised controlled trials: a simulation study′, Trials, 15(1), pp.264.

34. Rabe-Hesketh, S., Skrondal, A., (2012), Multilevel and Longitudinal Modeling Using Stata, Third Edition, College Station, Texas: Stata Press.

For peer review only - http://bmjopen.bmj.com/site/about/guidelines.xhtml

Barts and The London
School of Medicine and Dentistry

35. Kahan BC, Jairath V, Dore CJ, Morris TP., (2014), ´The risks and rewards of covariate adjustment in randomized trials: an assessment of 12 outcomes from 8 studies´, Trials, 15, pp.139.

36. White, IR., Horton, NJ., Carpenter, J., Pocock, SJ., (2011), ´Strategy for intention to treat analysis in randomised trials with missing outcome data`, BMJ, 342:d40.

37. Medicines and Healthcare products Regulatory Agency (2014) Medical device stand-alone software including apps. Available at: https://www.gov.uk/government/publications/medical-devices-software-applications-apps/medical-device-stand-alone-software-including-apps (Accessed: 13 May 2015)

For peer review only - http://bmjopen.bmj.com/site/about/guidelines.xhtml

Barts and The London
School of Medicine and Dentistry

APPENDICES

Appendix 1 – Information with regards to Safety Reporting in Non-CTIMP Research

	Who	When	How	To Whom
**SUSAR**	Chief Investigator	Report to the Sponsor, and QA manager within 24 hours<br>MREC within 15 days of learning of the event	SAE Report form for Non-CTIMPs, available from NRES website.	Sponsor and MREC
**Urgent Safety Measures**	Chief Investigator	Contact the Sponsor and MREC Immediately<br><br>Within 3 days	By phone<br><br>Substantial amendment form giving notice in writing setting out the reasons for the urgent safety measures and the plan for future action.	Main REC and Sponsor<br><br>Main REC with a copy also sent to the sponsor. The MREC will acknowledge this within 30 days of receipt.
**Progress Reports**	Chief Investigator	Annually (starting 12 months after the date of favourable opinion)	Annual Progress Report Form (non-CTIMPs) available from the NRES website	Main REC and Sponsor
**Declaration of the conclusion or early termination of the study**	Chief Investigator	Within 90 days (conclusion)<br><br>Within 15 days (early termination)<br><br>*The end of study should be defined in the protocol*	End of Study Declaration form available from the NRES website	Main REC with a copy to be sent to the sponsor
**Summary of final Report**	Chief Investigator	Within one year of conclusion of the Research	Where the study has met its objectives, the main findings and arrangements for publication or dissemination including feedback to participants	Main REC with a copy to be sent to the sponsor

For peer review only - http://bmjopen.bmj.com/site/about/guidelines.xhtml

MEMPHIS

MEMPHIS

Statistical Analysis Plan

Version: 3.0
Date: 26/Jan/2017

Person(s) contributing to the analysis plan	
Name(s) and position(s)	Neil Wright (Statistician)<br>Brennan Kahan (Statistician)<br>Elizabeth Ball (CI)<br>Gordon Forbes (Statistician)
Authorisation	
Position	**Chief or principal investigator**
Name	Elizabeth Ball
Signature	
Date	26/01/2017
Position	**Senior trial statistician**
Name	Brennan Kahan
Signature	
Date	26/01/2017
Position	**Independent statistician**
Name	Gian Luca Di Tanna
Tick once reviewed	✓
Date	30/01/2017

For peer review only - http://bmjopen.bmj.com/site/about/guidelines.xhtml

1. INTRODUCTION 3
2. STUDY METHODS..... 6
3. STUDY OUTCOMES..... 8
4. DATA COLLECTION 10
5. DERIVED VARIABLES..... 14
6. STATISTICAL ANALYSIS 15
7. GRAPHS AND FIGURES TO BE PRODUCED 19
8. REFERENCES..... 21
9. APPENDIX A: DERIVED AND COMPUTED VARIABLES 23
10. APPENDIX B: STATA CODE FOR GENERATING ADHERENCE OUTCOMES 29
11. APPENDIX C: STATA CODE FOR STATISTICAL ANALYSIS OF CLINICAL OUTCOMES ... 32
12. APPENDIX D: DRAFT TABLES 33
13. APPENDIX E: DATA / FILE MANAGEMENT 53

For peer review only - http://bmjopen.bmj.com/site/about/guidelines.xhtml

1. INTRODUCTION

1.1. Purpose of statistical analysis plan

The purpose of this document is to provide details of the statistical analyses and presentation of results to be reported within the principal paper(s) of the MEMPHIS trial. Any exploratory, post hoc or unplanned analyses will be clearly identified in the respective study analysis report. This document does not detail the qualitative analysis, and so aims and outcomes that are collected for qualitative analyses only are not included.

This document has been developed prior to examination of trial data and will not be implemented prior to final approval. Statisticians will be blinded to individual treatment allocations until this statistical analysis plan has been approved, all trial data has been collected and the trial is complete.

This document is based on protocol version 8.0 (December 2016)

1.2. Members of the writing committee

Neil Wright (Statistician) was primarily responsible for writing the Statistical Analysis Plan, with input from Brennan Kahan (Senior Statistician). Neil Wright was responsible for writing the computer code to implement the analysis strategy. Elizabeth Ball (CI) and Julie Dodds also contributed to this Statistical Analysis Plan.

1.3. Summary

Short Title	MEMPHIS
Methodology	A randomised feasibility trial
Research Sites	This trial will be conducted at the Royal London and Whipps Cross Hospitals
Objectives/Aims	The overall aim is to assess the feasibility of implementing a trial of a mindfulness meditation intervention delivered by a mobile phone app for patients with chronic pelvic pain (CPP). The primary objectives are:<br><br>To provide feasibility data for a large multicentre RCT aimed at rigorously testing mindfulness meditation in CPP<br><br>To determine whether this app can be seamlessly integrated into clinical practice, especially CPP pathways
Number of Participants/Patients	90 women with CPP will be recruited and each randomised into one of the three trial groups (meditation app, progressive muscle relaxation or no app).

For peer review only - http://bmjopen.bmj.com/site/about/guidelines.xhtml

Main Inclusion Criteria	To be eligible for the MEMPHIS study, the women must:<br>Be age 18 or over<br>Have either organic or non-organic chronic pelvic pain lasting for 6 months or more<br>Have access to a personal computer or smartphone.<br>Understand simple spoken English
Statistical Methodology and Analysis	Feasibility outcomes will be summarised using descriptive statistics. Clinical outcomes will be analysed using linear mixed-effects models, and results will be presented as a difference in means and a 95% confidence interval.

1.4. Changes from planned analysis in the protocol

- In the protocol, the dropout rate is a feasibility outcome but is not defined. In this analysis plan, we define two feasibility outcomes as "the number and proportion of participants who never return or answer a follow-up questionnaire at 6 months post-randomisation" and "the number and proportion of participants who do not return a follow-up questionnaire, but do answer the questionnaire by phone at 6 month post-randomisation".

- In the protocol, duration of recruitment is described as "the number of days from the beginning to the end of recruitment". In this analysis plan, duration of recruitment is defined as "the number of days from the day recruitment opens until the day the 90th patient is randomised (inclusive of both end days)".

- In the protocol, "Sexual Health Outcomes score (as measured by Sexual Health Outcomes in Women Questionnaire (SHOW-Q))" is given as a clinical outcome. In this analysis plan, this is replaced by the SHOW-Q global score, for sexually active participants, and by the SHOW-Q pelvic interference score, for all participants.

1.5. Changes from SAP v1.0

- In section 1.4 of version 1.0 of the SAP we stated *"In the protocol, "Quality of life score (as measured by the RAND Short form (36) Health Survey (SF-36))" is given as a clinical outcome. In this analysis plan, this is replaced by four of the RAND SF-36 subscales: physical functioning, general health, social functioning, and pain."* This has now been removed from the SAP as the protocol has been updated to reflect the change in the way quality of life score is being measured.

- The definition of app use has been changed from "having completed at least 50% of a session" to "having completed at least 90% of a session" (section 3.1). The change was

made due to Headspace, the data provider of the app usage data, only collecting data on sessions which were at least 90% complete.

1.6. Changes from SAP v2.0

- Added clarification to section 4.3 that data collected outside the recommended window for follow-up will still be included in analysis.
- In section 6.5.1, specified that the number of CRFs returned within the follow-up windows specified in section 4.3 will be summarised.
- Corrected scoring of CPAQ in Appendix A.
- Amended scoring of MYMOP in Appendix A so item scores are missing if the symptoms or activities are entered differently at follow up time points.

For peer review only - http://bmjopen.bmj.com/site/about/guidelines.xhtml

2. STUDY METHODS

2.1. Study objectives

The overall aim is to assess the feasibility of implementing a trial of a mindfulness meditation intervention delivered by a mobile phone app for patients with chronic pelvic pain (CPP). The primary objectives are:

- To provide feasibility data for a large multicentre RCT aimed at rigorously testing Mindfulness meditation in patients with CPP.
- To determine whether this app can be seamlessly integrated into clinical practice, especially CPP pathways.

2.2. Overall study design and plan

MEMPHIS is a randomised feasibility trial. Eligible women will be randomised to one of the three treatment groups:

- Intervention: 60 days of the app delivering mindfulness meditation content (in addition to usual care).
- Active control: 60 days of the app delivering progressive muscle relaxation content (in addition to usual care).
- Treatment as usual: Usual care

2.3. Selection of study population

2.3.1. Inclusion Criteria

To be eligible for the MEMPHIS study, the women must meet the following criteria:

- Aged 18 or over
- Women with organic and non-organic chronic pelvic pain lasting for six months or more
- Be capable of understanding the information provided, with use of an interpreter if required and being able to understand simple English as is used in the app
- Give written informed consent

2.3.2. Exclusion Criteria

Patients who meet the following criteria are ineligible to participate:

- No access to a Personal computer or smartphone

For peer review only - http://bmjopen.bmj.com/site/about/guidelines.xhtml

2.4. Method of treatment assignment and randomisation

After informed consent, patients will be randomised using a central, web-based system in a 1:1:1 ratio to one of the three treatment groups, using permuted blocks (of sizes 27, 30, 33) without stratification.

2.5. Sample size determination

30 participants will be recruited to each of the three treatment groups, giving a total of 90 participants. As this is a feasibility study, we have not performed a sample size calculation based upon the power to detect a significant treatment effect on a clinical outcome. However, 90 participants should provide a reliable estimate for the standard deviation of the primary clinical outcome (likely to be pain acceptance) [1, 2], which can be used to inform the sample size calculation of the main trial.

For peer review only - http://bmjopen.bmj.com/site/about/guidelines.xhtml

3. STUDY OUTCOMES

3.1. Feasibility outcomes

- Duration of recruitment (measured from the day recruitment opens until the day the 90th patient is randomised)
- Estimates to be used for the sample size calculation of the phase III RCT:
  - The estimated SD at 60 days, 3 months, and 6 months post-randomisation for pain acceptance (as measured by the Chronic Pain Acceptance Questionnaire (CPAQ-8))
  - The number and proportion of participants who never return or answer a follow-up questionnaire at 6 months post-randomisation.
  - The number and proportion of participants who do not return a follow-up questionnaire, but do answer the questionnaire by phone at 6 month post-randomisation.
- Patient adherence to app use measured by the following outcomes:
  - Number of days (within the first 60 days from randomisation) a patient has used the app (with app use defined as having completed at least 90%% of a session).
  - Whether the patient has used the app on 22 or more days within the first 60 days from randomisation.
  - Number of weeks (within the first eight weeks from randomisation) a patient has used the app on three or more days.
  - Whether the patient has used the app on three or more days in 6 or more weeks (within the first eight weeks from randomisation).
  - Whether the patient has used the app on 22 or more days within the first 60 days from randomisation, AND used the app on three or more days in 6 or more weeks within the first eight weeks from randomisation.

3.2. App satisfaction questionnaires

At 60 days post-randomisation:

- System Usability Scale (SUS) score (0 [worst] – 100 [best])
- Reponses to the purpose made app satisfaction questionnaire

For peer review only - http://bmjopen.bmj.com/site/about/guidelines.xhtml

3.3. Clinical outcomes

The following clinical outcomes at 60 days, 3 months, and 6 months post-randomisation:

- Pain acceptance score (as measured by the Chronic Pain Acceptance Questionnaire (CPAQ-8)) (0 [worst] – 48 [best])
- RAND Short form (36) Health Survey (RAND SF-36) scales:
    - Physical functioning (0 [worst] – 100 [best])
    - Pain (0 [worst] – 100 [best])
    - General health (0 [worst] – 100 [best])
    - Social functioning (0 [worst] – 100 [best])
- Depression score (as measured by the Hospital Anxiety and Depression Scale (HADS)) (0 [best] – 21 [worst])
- Anxiety score (as measured by HADS) (0 [best] – 21 [worst])
- Mindfulness score (as measure by the Cognitive and Mindfulness - Revised (CAMS – R) scale) (12 [worst] – 48 [best])
- Pain related disability score (as measured by the Chronic Pain Grade (CPG) disability subscale) (0 [best] – 100 [worst])
- Self efficacy score (as measured by the Pain Self-Efficacy Questionnaire (PSEQ)) (0 [worst] – 60 [best])
- Sexual Health Outcomes scores (as measured by the Sexual Health Outcomes in Women Questionnaire (SHOW-Q)):
    - SHOW-Q global score, for sexually active participants (0 [worst] – 100 [best])
    - SHOW-Q pelvic interference score, for all participants (0 [best] – 100 [worst])
- Subjective outcome score (as measured by the Measure Yourself Medical Outcome Profile (MYMOP)) (0 [best] – 6 [worst])

The following qualitative outcomes are not included in the Statistical Analysis Plan:

- Reasons for patient non-adherence to app use
- Obstacles to recruitment from participants and recruiting staff
- Usability/integration etc
- Determining primary/secondary outcomes of interest
- App satisfaction questionnaires for service providers

For peer review only - http://bmjopen.bmj.com/site/about/guidelines.xhtml

4. DATA COLLECTION

This section describes the variables that will be collected during the trial to be used in the analysis described by this plan.

4.1. Collected at baseline only

The following variables will be collected for each participant at baseline only.

Demographic:

- Age
- Weight
- Height
- Living arrangements (Alone, With others)
- Employment status (Employed (full or part time, including self-employment), Unemployed and looking for work, At school or in full time education, Unable to work due to long term sickness, Looking after your home/family, Retired from paid work, Other)
- Age left full time education (I did not receive a formal education, Age 12 or less, Age 13 to 16, Age 17 to 19, Age 20 or over, I am still in full time education, Other)
- Ethnic group (White, Black, Central Asian, Middle Eastern, Southern Asian, Mixed, Other ethnic group, Do not wish to say)
- Do you smoke (Yes, No)
- Number of cigarettes per week
- Do you drink alcohol (Yes, No)
- Number of alcohol units per week

Prior and concurrent treatment:

- Treatment used in last six months: Acupuncture; Gabapentin; Amitriptyline; Biofeedback; Botox injection; Contraceptive pills/patch/ring; Exercise, yoga or pilates; Injections to suppress ovaries (e.g. Prostap, Zoladex); Herbal Medicine; Meditation or relaxation exercises; Massage; Nutrition/diet; Codeine or Morphine type painkillers; Nerve blocks; Over the counter medication; Physiotherapy; Psychological (talking) therapy; Transcutaneous Electrical Nerve Stimulation (TENS); Surgery; Other. (One variable for each: Yes, No.)
- Currently using pain treatment (Yes, No)

Participants' pain:

For peer review only - http://bmjopen.bmj.com/site/about/guidelines.xhtml

- Length of pain (0-6 months, 7-12 months, 1-2 years, 3-5 years, 6-10 years, More than 10 years)
- Pain over the past week (0 [No pain] to 10 [Pain as bad as could be])

4.2. Randomisation details

The following variables for each participant will be held in the randomisation database.

- Date of randomisation
- Treatment group allocation

4.3. Collected at baseline and follow up

The following clinical outcome variables will be collected for each participant at baseline, 60 days, 3 months, and 6 months post-randomisation. We aim to collect 60 day follow up data between 46 and 74 days from randomisation, 3 month follow up date between 76 and 104 days and 6 month follow up data between 159 and 201 days. However, data collected outside these day ranges will be included in the analysis.

- Pain acceptance (as measured Chronic Pain Acceptance Questionnaire (CPAQ-8)) (4 variables)
- Short form (36) Health Survey (SF-36) (36 variables)
- Depression (as measured by the Hospital Anxiety and Depression Scale (HADS)) (7 variables)
- Anxiety (as measured by HADS) (7 variables)
- Mindfulness (as measure by the Cognitive and Mindfulness - Revised (CAMS – R) scale) (12 variables)
- Pain related disability (as measured by the Chronic Pain Grade (CPG) disability subscale) (3 variables)
- Self efficacy (as measured by the Pain Self-Efficacy Questionnaire (PSEQ)) (10 variables)
- Sexual Health Outcomes (as measured by Sexual Health Outcomes in Women Questionnaire (SHOW-Q)) (12 variables)
- Subjective outcome (as measured by Measure Yourself Medical Outcome Profile (MYMOP)) (4 variables)

For peer review only - http://bmjopen.bmj.com/site/about/guidelines.xhtml

Date of visit / date completed and method of collection (return of postal questionnaire or via telephone) for each follow-up questionnaire will also be collected. When the follow-up questionnaire is answered via telephone, the variables for the Short form (36) Health Survey (SF-36), Self efficacy (as measured by the Pain Self-Efficacy Questionnaire (PSEQ)), and Sexual Health Outcomes (as measured by Sexual Health Outcomes in Women Questionnaire (SHOW-Q)) are not collected.

4.4. App usage data

App usage data will be received from Headspace, for all participants randomised to the Intervention or Active Control arms. The data will include variables for participant login token, duration of session, filename of session, date and time of completion. Each observation represents one user completing (at least 90% of) a mindfulness meditation or muscle relaxation session.

4.5. App satisfaction questionnaires

The following variables will be collected for participants randomised to an app arm, at 60 days post-randomisation:

- System Usability Scale (SUS) (10 variables)
- Purpose made questionnaire responses:
  - Nine statements with categorical response. (Totally disagree, Somewhat disagree, Neither agree nor disagree, Somewhat agree, Totally agree) (9 variables)
  - One question (Did you use the app every day? (Yes, No))

4.6. Unintentional unblinding of randomised treatment

After the participant has been randomised, the following variables will be collected from the researcher:

- Was the participant randomised to the app treatment arm? (Yes, No)
- If the participant was allocated to the app treatment arm, which app treatment do you believe the participant was randomised to? (Intervention app, Control app, Don’t know)

At 6 months (between 159 and 201 days) post-randomisation, the following variables will be collected from the participant:

- Did you use the smartphone app for MEMPHIS? (Yes, No)

For peer review only - http://bmjopen.bmj.com/site/about/guidelines.xhtml

- Do you think you received the new treatment or comparison treatment? (New Treatment, Comparison Treatment, Don't Know)

For peer review only - http://bmjopen.bmj.com/site/about/guidelines.xhtml

5. DERIVED VARIABLES

5.1. Feasibility outcomes

A participant is counted as never having returned follow-up questionnaire at 6 months post-randomisation if date of visit / date completed and all other fields in the follow-up questionnaire are missing.

The patient adherence to app use outcomes listed in Section 3.1 will be calculated from the app usage data described in Section 4.4. Completing a session that is at least ten minutes on a day counts as having used the app on that day. Sample Stata code showing the calculation of these outcome variables is given in APPENDIX B: STATA CODE FOR GENERATING ADHERENCE OUTCOMES.

In the app usage data, date and timestamps will be provided in Coordinated Universal Time (UTC). These will be converted to UK time (BST/GMT as appropriate) before outcomes are derived.

5.2. Clinical outcomes

Details for how the clinical outcome scores list in Section 3.3 are derived from question responses (Section 4.2) are given in APPENDIX A: DERIVED AND COMPUTED VARIABLES.

5.3. System Usability Score (SUS) score

Details for how the System Usability Scale (SUS) score is derived from question responses is given in APPENDIX A: DERIVED AND COMPUTED VARIABLES.

For peer review only - http://bmjopen.bmj.com/site/about/guidelines.xhtml

6. STATISTICAL ANALYSIS

6.1. Analysis populations

All analyses will be carried out according to the intention-to-treat (ITT) principle: all patients with a non-missing outcome will be analysed according to the group to which they are randomised.

Summaries of patient adherence to app use will include all participants randomised to the intervention or active control treatment groups.

Sample means and SDs for clinical outcomes will include all participants with a non-missing outcome at that time point.

Analyses to estimate treatment effects for clinical outcomes (Section 6.4.2) will include all patients with a non-missing outcome for at least one of the three follow-up time points (60 days, 3 months, or 6 months) [3]. Patients with a missing outcome at all follow-up time points for a clinical outcome are excluded from the analysis of that clinical outcome. A clinical outcome is non-missing if there are recorded responses at that time point for all individual questions required for the derivation of the clinical outcome. (Note that for the Subjective outcome score (MYMOP profile score), only symptom 1 score and wellbeing score are required.)

6.2. Baseline variables

Demographic, prior and concurrent treatment, and participants' pain baseline variables are listed in Section 4.1. Each variable (plus body mass index instead of height and weight) will be summarised for each treatment group by the mean (SD) or median (IQR) for continuous variables, and the number (%) for categorical variables. Draft tables are given in APPENDIX D: DRAFT TABLES.

6.3. Analysis of feasibility outcomes

Duration of recruitment will be stated. It is the number of days from the day recruitment opens until the day the 90th patient is randomised (inclusive of both end days).

The number of participants randomised in each one month period from the day recruitment opens will be presented.

The estimated SD in each treatment group at each follow-up time point for pain acceptance (as measured by the Chronic Pain Acceptance Questionnaire (CPAQ-8)) will be presented.

Each patient adherence to app use outcome listed in Section 3.1 will be summarised separately for the intervention and active control treatment groups. Each outcome will be presented as the mean (SD) or median (IQR) for continuous variables, and the number (%) for categorical variables. Draft tables are given in APPENDIX D: DRAFT TABLES.

For peer review only - http://bmjopen.bmj.com/site/about/guidelines.xhtml

Barts and The London School of Medicine and Dentistry

PCTU

6.4. Analysis of clinical outcomes

6.4.1. Descriptive statistics

For each clinical outcome listed in Section 3.3 we will present:

- The number of patients in each treatment group with a non-missing outcome at each time point.
- The mean (SD) in each treatment group at each time point.

6.4.2. Statistical analysis

For each clinical outcome we will present estimated treatment effects for each follow-up time point, with a 95% confidence interval. Estimates of treatment effects will be presented comparing the intervention group (mindfulness meditation app) to the control (treatment as usual) group, the intervention group to the active control (progressive muscle relaxation app) group, and the active control group to the control group.

Outcomes will be analysed using linear mixed-effects models with outcome measurement (at three follow-up time points) as the dependant variable. The model will include fixed time effects, a fixed effect for treatment, time treatment interactions for 3 months and 6 months follow-up time points, and an unstructured correlation matrix for the residuals [4]. The model will include baseline measure of the outcome as a covariate, assuming a linear relationship between baseline and outcome [5]. The model will be fitted using restricted maximum likelihood. Example Stata code for this analysis model is given in APPENDIX C: STATA CODE FOR STATISTICAL ANALYSIS OF CLINICAL OUTCOMES.

If there are missing values for baseline measure of a clinical outcome, they will be replaced by the mean of the observed baseline values for all participants in all treatment arms (mean imputation) [6]. Missing values of clinical outcomes at follow-up will not be imputed.

If the mixed effects models fail to converge, treatment effects will be estimated using separate linear regression models for each follow-up time point. Baseline measure of the outcome will be included as a covariate.

6.5. Other analyses

6.5.1. Comparison of losses to follow-up

The number and proportion of patients in each treatment group who have returned, answered by phone, or never returned the follow-up questionnaire will be presented for each follow-up time point (60 days, 3 months, and 6 months post-randomisation). A patient is counted as having returned data unless date of visit / date completed and all other fields in the follow-up questionnaire are missing. A draft table is given in APPENDIX D: DRAFT

For peer review only - http://bmjopen.bmj.com/site/about/guidelines.xhtml

TABLES.Summaries of the following baseline variables will be presented separately for patients who have returned, answered by phone, or never returned the follow-up questionnaire at the 6 month time point:

- Age at randomisation
- Body mass index
- Living arrangements
- Employment status
- Age left full time education
- Ethnic group
- Do you smoke
- Number of cigarettes per week
- Do you drink alcohol
- Number of units of alcohol per week
- Length of pain
- Pain over the past week
- Baseline values of clinical outcomes:
  - Pain acceptance score
  - Depression score
  - Anxiety score
  - Pain related disability score

6.5.2. Unintentional unblinding of randomised treatment

For each participants in the intervention and active control arm, researcher response to the question "If the participant was allocated to the app treatment arm, which app treatment do you believe the participant was randomised to?" will be summarised by number and percentage.

For participants in the intervention and active control arms, response to the question "Do you think you received the new treatment or comparison treatment?" will be summarised by number and percentage. A draft table is given in APPENDIX D: DRAFT TABLES.

For peer review only - http://bmjopen.bmj.com/site/about/guidelines.xhtml

6.5.3. Summarising missing data in clinical outcomes

For each clinical outcome variable we will present the number and proportion of individuals for whom the outcome is complete for at least one of the three follow-up time points (60 days, 3 months, or 6 months).

For each clinical outcome variable, we will also present the number and proportion of individuals for whom the outcome is not completed (either because the questionnaire was not returned, or because the participant left all variables for that outcome blank), partially completed (one or more, but not all, variables used in its derivation are missing), or complete (no variables used in its derivation are missing) at each time point.

Completely missing and partially missing outcomes will be summarised separately according to whether follow-up was completed via the mail-in questionnaire or over the phone.

6.5.1. Summarising data returned outside of target follow up periods

The number and proportion of patients in each treatment group who had follow up questionnaires completed within the time periods specified in section 4.3 will be presented for each follow up point. These are between 46 and 74 days for 60 days follow up, between 76 and 104 days for 3 month follow up, and between 159 and 201 days for 6 month follow up.

6.5.2. App usability

The mean (SD) of the System Usability Scale (SUS) score will be presented separately for the treatment app and active control app arms.

The number and proportion of each response for each question in the purpose made app satisfaction questionnaire will be presented separated for the treatment app and active control app arms. The number and proportion responding "Yes" to the question "Did you use the app every day?" will also be presented for each app arm.

6.5.3. Serious adverse events

We will present the number of reported serious adverse events in each treatment arm.

6.6. Analysis software

The analysis will be carried out using Stata.

For peer review only - http://bmjopen.bmj.com/site/about/guidelines.xhtml

7. GRAPHS AND FIGURES TO BE PRODUCED

7.1. Participant flow

Participant throughput will be summarized in a CONSORT diagram:

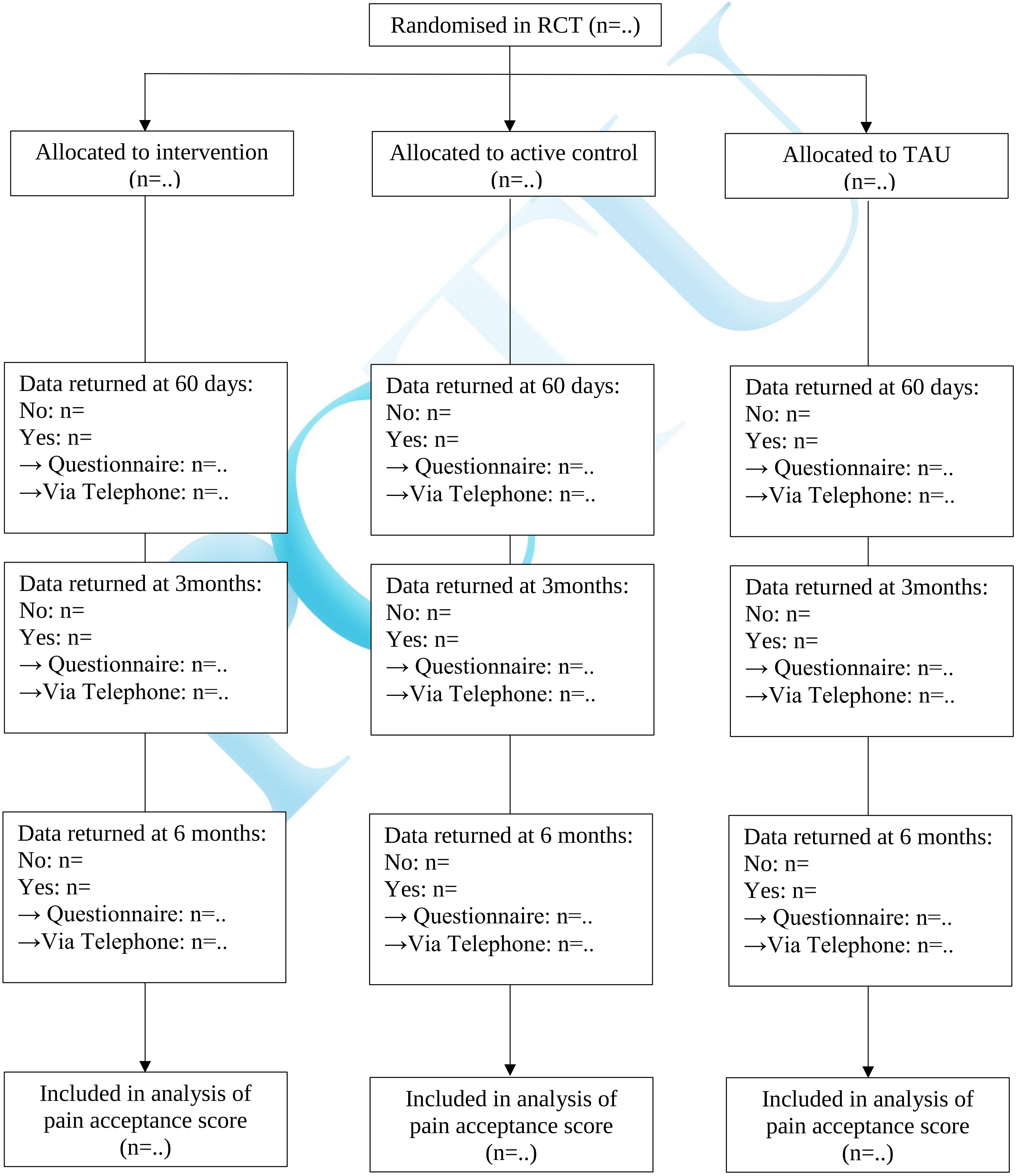

For peer review only - http://bmjopen.bmj.com/site/about/guidelines.xhtml

Barts and The London
School of Medicine and Dentistry

PCTU

7.2. Graphs

The following graphs will be created:

- Line graph showing mean CPAQ score at each time point for each treatment group. The graph will also include lines showing 95% confidence intervals for each mean CPAQ score.

- Line graph showing all estimated treatment effects (and 95% confidence intervals) on CPAQ score for each follow-up time point. (Estimates of treatment effects will be presented comparing the intervention group (mindfulness meditation app) to the control (treatment as usual) group, the intervention group to the active control (progressive muscle relaxation app) group, and the active control group to the control group.)

- Stacked bar chart showing the proportion of participants in each treatment group who have returned the follow-up questionnaire or answered the follow-up questionnaire by phone at each follow-up time point (60 days, 3 months, and 6 months post-randomisation).

For peer review only - http://bmjopen.bmj.com/site/about/guidelines.xhtml

Barts and The London
School of Medicine and Dentistry

PCTU

8. REFERENCES

[1] G. Lancaster, S. Dodd and P. Williamson, "Design and analysis of pilot studies: recommendations for good practice," *Journal of Evaluation in Clinical Practice,* vol. 10, no. 2, pp. 307-12, 2004.

[2] M. Teare, M. Dimairo, N. Shephard, A. Hayman, A. Whitehead and S. Walters, "Sample size requirements to estimate key design parameters from external pilot randomised controlled trials: a simulation study," *Trials*, vol. 15, p. 264, 2004.

[3] I. R. White, N. J. Horton, J. Carpenter and S. J. Pocock, "Strategy for intention to treat analysis in randomised trials with missing outcome data.," *BMJ (Clinical research ed.),* vol. 342, p. d40, 2011.

[4] S. Rabe-Hesketh and A. Skrondal, Multilevel and Longitudinal Modeling Using Stata. Volume I: Continuous Responses, Stata Press, 2012.

[5] B. C. Kahan, V. Jairath, C. J. Doré and T. P. Morris, "The risks and rewards of covariate adjustment in randomized trials: an assessment of 12 outcomes from 8 studies," *Trials,* vol. 15, p. 139, 2014.

[6] I. White and S. Thompson, "Adjusting for partially missing baseline measurements in randomized trials.," *Statistics in Medicine,* vol. 24, no. 7, pp. 993-1007, 2005.

[7] "RAND 36-Item Short Form Survey (SF-36) Scoring Instructions," [Online]. Available: https://www.rand.org/health/surveys_tools/mos/36-item-short-form/scoring.html.

[8] A. S. Zigmond and R. Snaith, "The hospital anxiety and depression scale.," *Acta psychiatrica Scandinavica,* no. 6, pp. 361-370, 1983.

[9] G. Feldman, A. Hayes, S. Kumar, J. Greeson and J.-P. Laurenceau, "Mindfulness and Emotion Regulation: The Development and Initial Validation of the Cognitive and Affective Mindfulness Scale-Revised (CAMS-R)," *Journal of Psychopathology and Behavioral Assessment,* vol. 29, p. 177–190, 2007.

[10] M. V. Korff, J. Ormel, F. J. Keefe and S. F. Dworkin, "Grading the severity of chronic pain," *Pain,* vol. 50, no. 2, pp. 133-149, 1992.

[11] M. K. Nicholas, "The pain self-efficacy questionnaire: Taking pain into account," *European Journal of Pain,* vol. 11, p. 153–163, 2007.

[12] R. A. Fish, B. McGuire, M. Hogan, T. G. Morrison and I. Stewart, "Validation of the Chronic Pain Acceptance Questionnaire (CPAQ) in an Internet," *Pain,* vol. 149, p. 435–443, 2010.

For peer review only - http://bmjopen.bmj.com/site/about/guidelines.xhtml

[13] [Online]. Available: http://www.bris.ac.uk/primaryhealthcare/resources/mymop/general-information/.

[14] [Online]. Available: https://www.usability.gov/how-to-and-tools/methods/system-usability-scale.html.

For peer review only - http://bmjopen.bmj.com/site/about/guidelines.xhtml

9. APPENDIX A: DERIVED AND COMPUTED VARIABLES

Unless otherwise stated, if an individual response variable used in the derivation of an outcome is missing then the outcome variable is missing.

Variables names used in the example code correspond to the field names specified in the trial database "Requirements Specification Document".

Body mass index

BMI is calculated as a person's weight (measured in kilograms) divided by the square of their height (measured in metres).

```
generate BMI = WEIGHT / ((HEIGHT / 100)^2)
```

RAND Short form (36) Health Survey (SF-36) scales scores [7]

Responses to individual questions are recoded as shown in the first table below. Each scale score is the average score for the questions in that scale, as shown in the second table below.

Item numbers	Original response code	Recode to
GH1, GH2, GH6, GH8, GH11b, GH11d	1	100
	2	75
	3	50
	4	25
	5	0
GH3a, GH3b, GH3c, GH3d, GH3e, GH3f, GH3g, GH3h, GH3i, GH3j	1	0
	2	50
	3	100
GH10, GH11a, GH11c	1	0
	2	25
	3	50
	4	75
	5	100
GH7	1	100
	2	80
	3	60
	4	40
	5	20
	6	0

Scale	After recoding, average the following items

For peer review only - http://bmjopen.bmj.com/site/about/guidelines.xhtml

Physical functioning	GH3a, GH3b, GH3c, GH3d, GH3e, GH3f, GH3g, GH3h, GH3i, GH3j
Pain	GH7, GH8
General health	GH1, GH11a, GH11b, GH11c, GH11d
Social functioning	GH6, GH10

```
recode GH1 GH2 GH6 GH8 GH11b GH11d (1=100) (2=75) (3=50) (4=25)
(5=0)
recode GH3a GH3b GH3c GH3d GH3e GH3f Gh3g GH3h GH3i Gh3j (1=0)
(2=50) (3=100)
recode GH10 GH11a GH11c (1=0) (2=25) (3=50) (4=75) (5=100)
recode GH7 (1=100) (2=80) (3=60) (4=40) (5=20) (6=0)
generate SF36_PHYSICALFUNC = (GH3a + GH3b + GH3c + GH3d + GH3e
+ GH3f + GH3g + GH3h + GH3i + GH3j) / 10
generate SF36_SOCIALFUNC = (GH6 + GH10) / 2
generate SF36_PAIN = (GH7 + GH8) / 2
generate SF36_GENERALHEALTH = (GH1 + GH11a + GH11b + GH11c +
GH11d) / 5
```

Depression score (as measured by the Hospital Anxiety and Depression Scale (HADS)) [7]

After appropriate recoding, the HADS depression score is the sum of scores for questions 2, 4, 6, 8, 10, 12 and 14.

```
recode HADS02 HADS04 HADS12 HADS14 (1=0) (2=1) (3=2) (4=3)
recode HADS06 HADS08 HADS10 (1=3) (2=2) (3=1) (4=0)
generate HADS_DEPRESSION = HADS02 + HADS04 +HADS06 + HADS08 +
HADS10 + HADS12 + HADS14
```

Anxiety score (as measured by HADS) [7]

After appropriate recoding, the HADS anxiety score is the sum of scores for questions 1, 3, 5, 7, 9, 11 and 13.

```
recode HADS01 HADS03 HADS05 HADS11 HADS13 (1=3) (2=2) (3=1)
(4=0)
recode HADS07 HADS09 (1=0) (2=1) (3=2) (4=3)
generate HADS_ANXIETY = HADS01 + HADS03 + HADS05 + HADS07 +
HADS09 + HADS11 + HADS13
```

Mindfulness score (as measure by the Cognitive and Mindfulness - Revised (CAMS – R) scale) [8]

Barts and The London School of Medicine and Dentistry PCTU

After appropriate recording, the CAMS-R mindfulness score is the sum of scores for all questions 1 to 12.

```
recode CAMSR02 CAMSR06 CAMSR07 (1=4) (2=3) (3=2) (4=1)
generate CAMSR_SCORE = CAMSR01 + CAMSR02 + CAMSR03 + CAMSR04 +
CAMSR05 + CAMSR06 + CAMSR07 + CAMSR08 + CAMSR09 + CAMSR10 +
CAMSR11 + CAMSR12
```

Pain related disability score (as measured by the Chronic Pain Grade (CPG) disability subscale) [9]

THE CPG pain related disability score is the mean of the daily activities, social activities, and work activities scores, multiplied by 10.

```
generate CPG_DISABILITYSCORE = [(CPGd1 + CPGd2 + CPGd3) / 3 ] *
10
```

Self efficacy score (as measured by the Pain Self-Efficacy Questionnaire (PSEQ)) [10]

The PSEQ self efficacy score is the sum of scores for all questions 1 to 10.

```
generate PSEQ_SCORE = PSEQ01 + PSEQ02 + PSEQ03 + PSEQ04 + PSEQ05
+ PSEQ06 + PSEQ07 + PSEQ08 + PSEQ09 + PSEQ10
```

Pain acceptance score (as measured Chronic Pain Acceptance Questionnaire (CPAQ-8)) [12]

After reverse scoring, the CPAQ-8 pain willingness score is the sum of scores from questions 4, 5, 7 and 8. The CPAQ-8 activity engagement score is the sum of scores from questions 1, 2, 3, 5 and 6. The CPAQ-8 total score is the sum of the pain willingness score and the activity engagement score.

```
recode CPAQ CPAQ4 CPAQ5 CPAQ7 CPAQ8 (0=6) (1=5) (2=4) (3=3)
(4=2) (5=1) (6=0)
generate CPAQ_PAINWILL = CPAQ4 + CPAQ5 + CPAQ7 + CPAQ8
generate CPAQ_ACTIVITYENG = CPAQ1 + CPAQ2 + CPAQ3 + CPAQ6
generate CPAQ_TOTAL = CPAQ_PAINWILL + CPAQ_ACTIVITYENG
```

Sexual Health Outcomes score (as measured by Sexual Health Outcomes in Women

Questionnaire (SHOW-Q))

Each response is rescaled to a score 0 to 100, with higher scores reflecting higher sexual functioning or fewer sexual problems. For a 5 response item, the scores are 0, 25, 50, 75 or 100. For a 4 response item, the scores are 0, 33.3, 66.7 or 100. The scoring for each question is shown in the table below.

If a participant answers "I don't have a partner" or "I don't have sex without a partner" to question 2 or "I did not have sexual activity" to any of questions 3, 4, 6, 7 or 9, then the participant is classed as sexually inactive. Otherwise, the participant is classed as sexually active.

For sexually active participants, the SHOW-Q global score is calculated as the mean of all rescaled scores. Higher scores reflect higher sexual functioning or fewer sexual problems.

For all participants, the SHOW-Q pelvic problem interference score is the mean of response scores to questions 10, 11 and 12 after they are reverse scored. Higher scores reflect more interference.

Item number	Response text	Original response code	Recode to
SHOWQ01, SHOWQ02	Very satisfied	1	100
	Somewhat satisfied	2	75
	Neither satisfied nor dissatisfied	3	50
	Somewhat dissatisfied	4	25
	Very dissatisfied	5	0
SHOWQ10, SHOWQ11, SHOWQ12	Not at all	1	100
	Slightly	2	75
	Moderately	3	50
	Quite a bit	4	25
	Extremely	5	0
SHOWQ03, SHOWQ04	Never	1	0
	Rarely	2	25
	Sometimes	3	50
	Most of the time	4	75
	All of the time	5	100
SHOWQ08	Never	1	0
	Once or twice	2	25
	3-4 times	3	50
	5-6 times	4	75
	More than 6 times	5	100
SHOWQ05	Did not experience any orgasms	1	0
	Mild	2	33.3
	Moderate	3	66.7
	Strong	4	100
SHOWQ06,	Not a problem	1	100

For peer review only - http://bmjopen.bmj.com/site/about/guidelines.xhtml

SHOWQ07, SHOWQ09	Little of a problem	2	66.7
	Somewhat of a problem	3	33.3
	Very much of a problem	4	0

```
generate SHOWQ_ACTIVE = 1
replace SHOWQ_ACTIVE = 0 if SHOWQ02==6 | SHOWQ02==7 | SHOWQ03==6
| SHOW04==6 | SHOWQ06==5 | SHOWQ07==5 | SHOWQ09== 5
recode SHOWQ01 SHOWQ02 SHOW10 SHOWQ11 SHOWQ12 (1=100) (2=75)
(3=50) (4=25) (5=0)
recode SHOWQ03 SHOWQ04 SHOWQ08 (1=0) (2=25) (3=50) (4=75)
(5=100)
recode SHOWQ05 (1=0) (2=33.3) (3=66.7) (4=100)
recode SHOWQ06 SHOWQ07 SHOWQ09 (1=100) (2=66.7) (3=33.3) (4=0)
generate SHOWQ_GLOBAL = (SHOWQ01 + SHOWQ02 + SHOWQ03 + SHOWQ04
+ SHOWQ05 + SHOWQ06 + SHOWQ07 + SHOWQ08 + SHOWQ09 + SHOWQ10 +
SHOWQ11 + SHOWQ12)/12 if SHOWQ_ACTIVE == 1
generate SHOWQ_PELVPROBLEM = ((100 - SHOWQ10) + (100 - SHOWQ11)
+ (100 - SHOWQ12))/3
```

Subjective outcome score (as measured by Measure Yourself Medical Outcome Profile (MYMOP)) [12]

If the description for symptom 1, symptom 2, symptom 3 or activity does not match the description given for the corresponding symptom or activity at baseline then the score for that symptom or activity is missing.

If symptom 1 score or wellbeing score are missing, then MYMOP profile score is missing.The MYMOP profile score is the mean of the symptom 1 score, symptom 2 score, activity score, wellbeing score, and symptom 3 score. (Symptom 2 score, activity score and symptom 3 score are only included if they are not missing)

```
egen MYMOP_PROFILE = rowmean(SYMSCORE1, SYMSCORE2, ACTSCORE,
WELLBEING, SYMSCORE3)
```

System Usability Scale (SUS) score [13]

For questions 1, 3, 5, 7, and 9 the score contribution is the response number minus 1. For questions 2, 4, 6, 8, and 10 the score contribution is 5 minus the response number. The SUS score is the sum of all score contributions multiplied by 2.5

```
recode SUS01 SUS03 SYS05 SUS07 SUS09 (1 = 0) (2 = 1) (3 = 2) (4 = 3) (5 = 4)
recode SUS02 SUS04 SUS06 SUS08 SUS10 (1 = 4) (2 = 3) (3 = 2) (4 = 1) (5 = 0)
generate SUS_SCORE  = 2.5 * (SUS01 + SUS02 + SUS03 + SUS04 + SUS05 +SUS06 + SUS07 + SUS08 + SUS9 + SUS10)
```

Adherence outcomes

countin60days	Number of days (within the first 60 days from randomisation) a patient has used the app (with app use defined as having completed at least 90% of a session).	
numberofweeksthreeplus	Number of weeks (within the first eight weeks from randomisation) a patient has used the app on three or more days.	
adhere_countin60days	Whether the patient has used the app on 22 or more days within the first 60 days from randomisation.	1 = Yes<br>0 = No
adhere_numberofweeksthreeplus	Whether the patient has used the app on three or more days in 6 or more weeks (within the first eight weeks from randomisation).	1 = Yes<br>0 = No

Sample Stata code showing the calculation of these outcome variables is given in APPENDIX B: STATA CODE FOR GENERATING ADHERENCE OUTCOMES.

10. APPENDIX B: STATA CODE FOR GENERATING ADHERENCE OUTCOMES

Sample of Stata code for generating adherence outcomes from app usage data supplied by Headspace:

```
gen date_completed = date(datecompleted, "DMY")

format date_completed %td


gen date_rand = date(dateofrandomisation, "DMY")

format date_rand %td


gen date_fromrand = date_completed-date_rand


***


* Drop sessions which are not part of intervention (i.e. short duration)

drop if duration<5


* Remove multiple sessions in same day

duplicates report id date_fromrand
```

For peer review only - http://bmjopen.bmj.com/site/about/guidelines.xhtml

Barts and The London
School of Medicine and Dentistry

PCTU

```
duplicates drop id date_fromrand , force


gen in60days = 1 if date_fromrand<61

bysort id: egen countin60days = count(in60days)


gen numberofweeksthreeplus = 0


forvalues week=1/8 {

     gen inweek`week' = 1 if date_fromrand>7*(`week'-1) & date_fromrand<7*`week'+1

     gen threeplusinweek`week' = 0

     bysort id: egen countinweek`week' = count(inweek`week')

     assert countinweek`week'<8

     bysort id: replace threeplusinweek`week' = 1 if countinweek`week'>2

     bysort id: replace numberofweeksthreeplus = numberofweeksthreeplus +1 if countinweek`week'>2

}
```

PCTU only

For peer review only - http://bmjopen.bmj.com/site/about/guidelines.xhtml

```
bysort id: keep if _n==1

keep id countin60days numberofweeksthreeplus threeplusinweek* countinweek*



gen adhere_countin60days = 0

replace adhere_countin60days = 1 if countin60days>21

gen adhere_numberofweeksthreeplus = 0

replace adhere_numberofweeksthreeplus = 1 if numberofweeksthreeplus>5



tab adhere_countin60days adhere_numberofweeksthreeplus
```

For peer review only - http://bmjopen.bmj.com/site/about/guidelines.xhtml

11. APPENDIX C: STATA CODE FOR STATISTICAL ANALYSIS OF CLINICAL OUTCOMES

The following Stata shows the model that will be used to estimate treatment effects on clinical outcomes:

```
xtmixed outcome time##treat baseline || id: , noconstant
residuals(unstructured, t(time)) var reml
```

Estimates of treatment effects for each treatment arm comparison and time point will then be obtained using:

```
lincom 1.treat + 1.time#1.treat

lincom 1.treat + 2.time#1.treat

lincom 1.treat + 3.time#1.treat

lincom 2.treat + 1.time#2.treat

lincom 2.treat + 2.time#2.treat

lincom 2.treat + 3.time#2.treat

lincom 2.treat + 1.time#2.treat - 1.treat + 1.time#1.treat

lincom 2.treat + 2.time#2.treat - 1.treat + 2.time#1.treat

lincom 2.treat + 3.time#2.treat - 1.treat + 3.time#1.treat
```

For peer review only - http://bmjopen.bmj.com/site/about/guidelines.xhtml

Barts and The London School of Medicine and Dentistry

PCTU

12. APPENDIX D: DRAFT TABLES

12.1.1. Baseline demographics and medical history

Figures are mean (SD) unless stated otherwise.

	Intervention (n=…)	Active control (n=…)	Usual care (n=…)
**Demographics**			
Age at randomisation (Years)	XX (XX)	XX (XX)	XX (XX)
Body mass index	XX (XX)	XX (XX)	XX (XX)
Living arrangements – no. (%)			
Alone	XX (XX)	XX (XX)	XX (XX)
With others	XX (XX)	XX (XX)	XX (XX)
Employment status – no. (%)			
Employed	XX (XX)	XX (XX)	XX (XX)
Unemployed and looking for work	XX (XX)	XX (XX)	XX (XX)
At school or in full time education	XX (XX)	XX (XX)	XX (XX)
Unable to work due to long term sickness	XX (XX)	XX (XX)	XX (XX)
Look after your home/family	XX (XX)	XX (XX)	XX (XX)
Retired from paid work	XX (XX)	XX (XX)	XX (XX)
Other	XX (XX)	XX (XX)	XX (XX)
Age left full time education – no. (%)			
I did not receive a formal education	XX (XX)	XX (XX)	XX (XX)
Age 12 or less	XX (XX)	XX (XX)	XX (XX)
Age 13 to 16	XX (XX)	XX (XX)	XX (XX)
Age 17 to 19	XX (XX)	XX (XX)	XX (XX)
Age 20 or over	XX (XX)	XX (XX)	XX (XX)
I am still in full time education	XX (XX)	XX (XX)	XX (XX)
Other	XX (XX)	XX (XX)	XX (XX)
Ethnic group – no. (%)			
White	XX (XX)	XX (XX)	XX (XX)
Black	XX (XX)	XX (XX)	XX (XX)
Central Asian	XX (XX)	XX (XX)	XX (XX)
Middle Eastern	XX (XX)	XX (XX)	XX (XX)
Southern Asian	XX (XX)	XX (XX)	XX (XX)
Mixed	XX (XX)	XX (XX)	XX (XX)
Other ethnic group	XX (XX)	XX (XX)	XX (XX)
Do not wish to say	XX (XX)	XX (XX)	XX (XX)
Smoker – no. (%)			
Yes	XX (XX)	XX (XX)	XX (XX)
No	XX (XX)	XX (XX)	XX (XX)
If yes, number of cigarettes per week	XX (XX)	XX (XX)	XX (XX)
Drink alcohol – no. (%)			

For peer review only - http://bmjopen.bmj.com/site/about/guidelines.xhtml

Barts and The London School of Medicine and Dentistry PCTU

	Intervention		Active control		Usual care	
Yes	XX	(XX)	XX	(XX)	XX	(XX)
No	XX	(XX)	XX	(XX)	XX	(XX)
If yes, number of units of alcohol per week	XX	(XX)	XX	(XX)	XX	(XX)
**Baseline medical history**						
Length of pain – no. (%)						
0-6 months	XX	(XX)	XX	(XX)	XX	(XX)
7-12 months	XX	(XX)	XX	(XX)	XX	(XX)
1-2 years	XX	(XX)	XX	(XX)	XX	(XX)
3-5 years	XX	(XX)	XX	(XX)	XX	(XX)
6-10 years	XX	(XX)	XX	(XX)	XX	(XX)
More than 10 years	XX	(XX)	XX	(XX)	XX	(XX)
Pain over the past week	XX	(XX)	XX	(XX)	XX	(XX)

12.1.2. Prior and concurrent treatment

Figures are number (percentage).

	Intervention (n=…)		Active control (n=…)		Usual care (n=…)	
Treatment used in last six months						
Acupuncture	XX	(XX)	XX	(XX)	XX	(XX)
Gabapentin	XX	(XX)	XX	(XX)	XX	(XX)
Amitriptyline	XX	(XX)	XX	(XX)	XX	(XX)
Biofeedback	XX	(XX)	XX	(XX)	XX	(XX)
Botox injection	XX	(XX)	XX	(XX)	XX	(XX)
Contraceptive pills/patch/ring	XX	(XX)	XX	(XX)	XX	(XX)
Exercise, yoga or pilates	XX	(XX)	XX	(XX)	XX	(XX)
Injections to suppress ovaries (e.g. Prostap, Zoladex)	XX	(XX)	XX	(XX)	XX	(XX)
Herbal Medicine	XX	(XX)	XX	(XX)	XX	(XX)
Meditation or relaxation exercises	XX	(XX)	XX	(XX)	XX	(XX)
Massage	XX	(XX)	XX	(XX)	XX	(XX)
Nutrition/diet	XX	(XX)	XX	(XX)	XX	(XX)
Codeine or Morphine type painkillers	XX	(XX)	XX	(XX)	XX	(XX)
Nerve blocks	XX	(XX)	XX	(XX)	XX	(XX)
Over the counter medication	XX	(XX)	XX	(XX)	XX	(XX)
Physiotherapy	XX	(XX)	XX	(XX)	XX	(XX)
Psychological (talking) therapy	XX	(XX)	XX	(XX)	XX	(XX)
Transcutaneous Electrical Nerve Stimulation (TENS)	XX	(XX)	XX	(XX)	XX	(XX)
Surgery	XX	(XX)	XX	(XX)	XX	(XX)

For peer review only - http://bmjopen.bmj.com/site/about/guidelines.xhtml

	Intervention		Active control		Usual care	
Other	XX	(XX)	XX	(XX)	XX	(XX)
Currently using pain treatment						
Yes	XX	(XX)	XX	(XX)	XX	(XX)
No	XX	(XX)	XX	(XX)	XX	(XX)

12.1.3. Baseline values of clinical outcomes

Figures are mean (SD) unless stated otherwise.

	Intervention (n=…)		Active control (n=…)		Usual care (n=…)	
SF-36 scales:						
Physical functioning	XX	(XX)	XX	(XX)	XX	(XX)
Pain	XX	(XX)	XX	(XX)	XX	(XX)
General Health	XX	(XX)	XX	(XX)	XX	(XX)
Social Functioning	XX	(XX)	XX	(XX)	XX	(XX)
Depression score	XX	(XX)	XX	(XX)	XX	(XX)
Anxiety score	XX	(XX)	XX	(XX)	XX	(XX)
Mindfulness score	XX	(XX)	XX	(XX)	XX	(XX)
Pain related disability score	XX	(XX)	XX	(XX)	XX	(XX)
Self efficacy score	XX	(XX)	XX	(XX)	XX	(XX)
Pain acceptance score	XX	(XX)	XX	(XX)	XX	(XX)
Sexual health outcomes:						
SHOW-Q global score	XX	(XX)	XX	(XX)	XX	(XX)
SHOW-Q pelvic problem interference score	XX	(XX)	XX	(XX)	XX	(XX)
Subjective outcome score	XX	(XX)	XX	(XX)	XX	(XX)

12.1.4. Loss to follow-up

	Intervention (n=…)		Active control (n=…)		Usual care (n=…)	
Follow-up questionnaire returned – no. (%)						
60 days	XX	(XX)	XX	(XX)	XX	(XX)
3 months	XX	(XX)	XX	(XX)	XX	(XX)
6 months	XX	(XX)	XX	(XX)	XX	(XX)
Follow-up questionnaire answered by phone – no. (%)						
60 days	XX	(XX)	XX	(XX)	XX	(XX)
3 months	XX	(XX)	XX	(XX)	XX	(XX)
6 months	XX	(XX)	XX	(XX)	XX	(XX)
Follow-up questionnaire never returned – no. (%)						
60 days	XX	(XX)	XX	(XX)	XX	(XX)
3 months	XX	(XX)	XX	(XX)	XX	(XX)
6 months	XX	(XX)	XX	(XX)	XX	(XX)

For peer review only - http://bmjopen.bmj.com/site/about/guidelines.xhtml

Barts and The London School of Medicine and Dentistry

PCTU

12.1.5. Loss to follow-up

Figures are mean (SD) unless stated otherwise.

	6 months follow-up questionnaire returned (n=…)	6 months follow-up questionnaire answered by phone (n=…)	6 months follow-up questionnaire never returned (n=…)
**Demographics**			
Age at randomisation (Years)	XX (XX)	XX (XX)	XX (XX)
Body mass index	XX (XX)	XX (XX)	XX (XX)
Living arrangements – no. (%)			
Alone	XX (XX)	XX (XX)	XX (XX)
With others	XX (XX)	XX (XX)	XX (XX)
Employment status – no. (%)			
Employed	XX (XX)	XX (XX)	XX (XX)
Unemployed and looking for work	XX (XX)	XX (XX)	XX (XX)
At school or in full time education	XX (XX)	XX (XX)	XX (XX)
Unable to work due to long term sickness	XX (XX)	XX (XX)	XX (XX)
Look after your home/family	XX (XX)	XX (XX)	XX (XX)
Retired from paid work	XX (XX)	XX (XX)	XX (XX)
Other	XX (XX)	XX (XX)	XX (XX)
Age left full time education – no. (%)			
I did not receive a formal education	XX (XX)	XX (XX)	XX (XX)
Age 12 or less	XX (XX)	XX (XX)	XX (XX)
Age 13 to 16	XX (XX)	XX (XX)	XX (XX)
Age 17 to 19	XX (XX)	XX (XX)	XX (XX)
Age 20 or over	XX (XX)	XX (XX)	XX (XX)
I am still in full time education	XX (XX)	XX (XX)	XX (XX)
Other	XX (XX)	XX (XX)	XX (XX)
Ethnic group – no. (%)			
White	XX (XX)	XX (XX)	XX (XX)
Black	XX (XX)	XX (XX)	XX (XX)
Central Asian	XX (XX)	XX (XX)	XX (XX)
Middle Eastern	XX (XX)	XX (XX)	XX (XX)
Southern Asian	XX (XX)	XX (XX)	XX (XX)
Mixed	XX (XX)	XX (XX)	XX (XX)
Other ethnic group	XX (XX)	XX (XX)	XX (XX)
Do not wish to say	XX (XX)	XX (XX)	XX (XX)
Smoker – no. (%)			

For peer review only - http://bmjopen.bmj.com/site/about/guidelines.xhtml

Yes	XX (XX)	XX (XX)	XX (XX)
No	XX (XX)	XX (XX)	XX (XX)
If yes, number of cigarettes per week	XX (XX)	XX (XX)	XX (XX)
Drink alcohol – no. (%)			
Yes	XX (XX)	XX (XX)	XX (XX)
No	XX (XX)	XX (XX)	XX (XX)
If yes, number of units of alcohol per week	XX (XX)	XX (XX)	XX (XX)
**Baseline medical history**			
Length of pain – no. (%)			
0-6 months	XX (XX)	XX (XX)	XX (XX)
7-12 months	XX (XX)	XX (XX)	XX (XX)
1-2 years	XX (XX)	XX (XX)	XX (XX)
3-5 years	XX (XX)	XX (XX)	XX (XX)
6-10 years	XX (XX)	XX (XX)	XX (XX)
More than 10 years	XX (XX)	XX (XX)	XX (XX)
Pain over the past week	XX (XX)	XX (XX)	XX (XX)
**Baseline values of clinical outcomes**			
Pain acceptance score (CPAQ-8)	XX (XX)	XX (XX)	XX (XX)
Depression score (HADS)	XX (XX)	XX (XX)	XX (XX)
Anxiety score (HADS)	XX (XX)	XX (XX)	XX (XX)
Pain related disability score (CPG)	XX (XX)	XX (XX)	XX (XX)

12.1.6. Follow up within target follow up period

	Intervention (n=…)	Active control (n=…)	Usual care (n=…)
Follow-up questionnaire returned or answered by phone within target follow up period– no. (%)			
60 days (46 and 74days)	XX (XX)	XX (XX)	XX (XX)
3 months (76 and 104 days)	XX (XX)	XX (XX)	XX (XX)
6 months (159 and 201 days)	XX (XX)	XX (XX)	XX (XX)

12.1.7. Adherence to app use

Figures are mean (SD) unless stated otherwise.

	Intervention (n=…)	Active control (n=…)
Number of days (within the first 60 days from randomisation) a patient has used the app	XX (XX)	XX (XX)

For peer review only - http://bmjopen.bmj.com/site/about/guidelines.xhtml

Barts and The London School of Medicine and Dentistry

PCTU

Number of weeks (within the first eight weeks from randomisation) a patient has used the app on three or more days	XX (XX)	XX (XX)
Used the app on 22 or more days within the first 60 days from randomisation – no. (%)	XX (XX)	XX (XX)
Used the app on three or more days in 6 or more weeks (within the first eight weeks from randomisation) – no. (%)	XX (XX)	XX (XX)
Used the app on 22 or more days within the first 60 days from randomisation, AND used the app on three or more days in 6 or more weeks within the first eight weeks from randomisation – no. (%)	XX (XX)	XX (XX)

12.1.8. App usability questionnaire

Figures are number (percentage).

	Totally disagree	Somewhat disagree	Neither agree nor disagree	Somewhat agree	Totally agree	Not answered
It is easy to access the app whenever I wanted to use it						
Intervention:	XX (XX)	XX (XX)	XX (XX)	XX (XX)	XX (XX)	XX (XX)
Active control:	XX (XX)	XX (XX)	XX (XX)	XX (XX)	XX (XX)	XX (XX)
After being shown, I understood how the app would work						
Intervention:	XX (XX)	XX (XX)	XX (XX)	XX (XX)	XX (XX)	XX (XX)
Active control:	XX (XX)	XX (XX)	XX (XX)	XX (XX)	XX (XX)	XX (XX)
It was fun to work with the app						
Intervention:	XX (XX)	XX (XX)	XX (XX)	XX (XX)	XX (XX)	XX (XX)
Active control:	XX (XX)	XX (XX)	XX (XX)	XX (XX)	XX (XX)	XX (XX)
The app worked well						
Intervention:	XX (XX)	XX (XX)	XX (XX)	XX (XX)	XX (XX)	XX (XX)
Active control:	XX (XX)	XX (XX)	XX (XX)	XX (XX)	XX (XX)	XX (XX)
It was easy to work through the modules						
Intervention:	XX (XX)	XX (XX)	XX (XX)	XX (XX)	XX (XX)	XX (XX)
Active control:	XX (XX)	XX (XX)	XX (XX)	XX (XX)	XX (XX)	XX (XX)

For peer review only - http://bmjopen.bmj.com/site/about/guidelines.xhtml

Barts and The London School of Medicine and Dentistry PCTU

The number of modules was annoying						
Intervention:	XX (XX)	XX (XX)	XX (XX)	XX (XX)	XX (XX)	XX (XX)
Active control:	XX (XX)	XX (XX)	XX (XX)	XX (XX)	XX (XX)	XX (XX)
The modules were well-displayed on my smartphone						
Intervention:	XX (XX)	XX (XX)	XX (XX)	XX (XX)	XX (XX)	XX (XX)
Active control:	XX (XX)	XX (XX)	XX (XX)	XX (XX)	XX (XX)	XX (XX)
Using the app was difficult because of my daily activities						
Intervention:	XX (XX)	XX (XX)	XX (XX)	XX (XX)	XX (XX)	XX (XX)
Active control:	XX (XX)	XX (XX)	XX (XX)	XX (XX)	XX (XX)	XX (XX)
Using the app took too long						
Intervention:	XX (XX)	XX (XX)	XX (XX)	XX (XX)	XX (XX)	XX (XX)
Active control:	XX (XX)	XX (XX)	XX (XX)	XX (XX)	XX (XX)	XX (XX)

For peer review only - http://bmjopen.bmj.com/site/about/guidelines.xhtml

Barts and The London School of Medicine and Dentistry PCTU

12.1.9. Clinical outcomes

	Intervention (n=…)				Active control (n=…)				Usual care (n=…)			
	n	(%)	Mean	(SD)	n	(%)	Mean	(SD)	n	(%)	Mean	(SD)
**Pain acceptance score**												
Baseline	XX	(XX)	XX	(XX)	XX	(XX)	XX	(XX)	XX	(XX)	XX	(XX)
60 days	XX	(XX)	XX	(XX)	XX	(XX)	XX	(XX)	XX	(XX)	XX	(XX)
3 months	XX	(XX)	XX	(XX)	XX	(XX)	XX	(XX)	XX	(XX)	XX	(XX)
6 months	XX	(XX)	XX	(XX)	XX	(XX)	XX	(XX)	XX	(XX)	XX	(XX)
Included in analysis †	XX	(XX)			XX	(XX)			XX	(XX)		
**Depression score**												
Baseline	XX	(XX)	XX	(XX)	XX	(XX)	XX	(XX)	XX	(XX)	XX	(XX)
60 days	XX	(XX)	XX	(XX)	XX	(XX)	XX	(XX)	XX	(XX)	XX	(XX)
3 months	XX	(XX)	XX	(XX)	XX	(XX)	XX	(XX)	XX	(XX)	XX	(XX)
6 months	XX	(XX)	XX	(XX)	XX	(XX)	XX	(XX)	XX	(XX)	XX	(XX)
Included in analysis †	XX	(XX)			XX	(XX)			XX	(XX)		
**Anxiety score**												
Baseline	XX	(XX)	XX	(XX)	XX	(XX)	XX	(XX)	XX	(XX)	XX	(XX)
60 days	XX	(XX)	XX	(XX)	XX	(XX)	XX	(XX)	XX	(XX)	XX	(XX)
3 months	XX	(XX)	XX	(XX)	XX	(XX)	XX	(XX)	XX	(XX)	XX	(XX)
6 months	XX	(XX)	XX	(XX)	XX	(XX)	XX	(XX)	XX	(XX)	XX	(XX)
Included in analysis †	XX	(XX)			XX	(XX)			XX	(XX)		

For peer review only - http://bmjopen.bmj.com/site/about/guidelines.xhtml

Barts and The London School of Medicine and Dentistry PCTU

**Mindfulness score**						
Baseline	XX (XX)	XX (XX)	XX (XX)	XX (XX)	XX (XX)	XX (XX)
60 days	XX (XX)	XX (XX)	XX (XX)	XX (XX)	XX (XX)	XX (XX)
3 months	XX (XX)	XX (XX)	XX (XX)	XX (XX)	XX (XX)	XX (XX)
6 months	XX (XX)	XX (XX)	XX (XX)	XX (XX)	XX (XX)	XX (XX)
Included in analysis †	XX (XX)		XX (XX)		XX (XX)	
**Pain related disability score**						
Baseline	XX (XX)	XX (XX)	XX (XX)	XX (XX)	XX (XX)	XX (XX)
60 days	XX (XX)	XX (XX)	XX (XX)	XX (XX)	XX (XX)	XX (XX)
3 months	XX (XX)	XX (XX)	XX (XX)	XX (XX)	XX (XX)	XX (XX)
6 months	XX (XX)	XX (XX)	XX (XX)	XX (XX)	XX (XX)	XX (XX)
Included in analysis †	XX (XX)		XX (XX)		XX (XX)	
**Self efficacy score**						
Baseline	XX (XX)	XX (XX)	XX (XX)	XX (XX)	XX (XX)	XX (XX)
60 days	XX (XX)	XX (XX)	XX (XX)	XX (XX)	XX (XX)	XX (XX)
3 months	XX (XX)	XX (XX)	XX (XX)	XX (XX)	XX (XX)	XX (XX)
6 months	XX (XX)	XX (XX)	XX (XX)	XX (XX)	XX (XX)	XX (XX)
Included in analysis †	XX (XX)		XX (XX)		XX (XX)	
**SHOW-Q global score, for sexually active participants**						
Baseline	XX (XX)	XX (XX)	XX (XX)	XX (XX)	XX (XX)	XX (XX)
60 days	XX (XX)	XX (XX)	XX (XX)	XX (XX)	XX (XX)	XX (XX)
3 months	XX (XX)	XX (XX)	XX (XX)	XX (XX)	XX (XX)	XX (XX)

PCTU only

6 months	XX	(XX)	XX	(XX)	XX	(XX)	XX	(XX)	XX	(XX)	XX	(XX)
Included in analysis †	XX	(XX)			XX	(XX)			XX	(XX)		
**SHOW-Q pelvic problem interference score, for all participants**												
Baseline	XX	(XX)	XX	(XX)	XX	(XX)	XX	(XX)	XX	(XX)	XX	(XX)
60 days	XX	(XX)	XX	(XX)	XX	(XX)	XX	(XX)	XX	(XX)	XX	(XX)
3 months	XX	(XX)	XX	(XX)	XX	(XX)	XX	(XX)	XX	(XX)	XX	(XX)
6 months	XX	(XX)	XX	(XX)	XX	(XX)	XX	(XX)	XX	(XX)	XX	(XX)
Included in analysis †	XX	(XX)			XX	(XX)			XX	(XX)		
**Subjective outcome score**												
Baseline	XX	(XX)	XX	(XX)	XX	(XX)	XX	(XX)	XX	(XX)	XX	(XX)
60 days	XX	(XX)	XX	(XX)	XX	(XX)	XX	(XX)	XX	(XX)	XX	(XX)
3 months	XX	(XX)	XX	(XX)	XX	(XX)	XX	(XX)	XX	(XX)	XX	(XX)
6 months	XX	(XX)	XX	(XX)	XX	(XX)	XX	(XX)	XX	(XX)	XX	(XX)
Included in analysis †	XX	(XX)			XX	(XX)			XX	(XX)		
**SF-36: Physical functioning**												
Baseline	XX	(XX)	XX	(XX)	XX	(XX)	XX	(XX)	XX	(XX)	XX	(XX)
60 days	XX	(XX)	XX	(XX)	XX	(XX)	XX	(XX)	XX	(XX)	XX	(XX)
3 months	XX	(XX)	XX	(XX)	XX	(XX)	XX	(XX)	XX	(XX)	XX	(XX)
6 months	XX	(XX)	XX	(XX)	XX	(XX)	XX	(XX)	XX	(XX)	XX	(XX)
Included in analysis †	XX	(XX)			XX	(XX)			XX	(XX)		
**SF-36: Pain**												
Baseline	XX	(XX)	XX	(XX)	XX	(XX)	XX	(XX)	XX	(XX)	XX	(XX)

For peer review only - http://bmjopen.bmj.com/site/about/guidelines.xhtml

60 days	XX (XX)	XX (XX)	XX (XX)	XX (XX)	XX (XX)	XX (XX)
3 months	XX (XX)	XX (XX)	XX (XX)	XX (XX)	XX (XX)	XX (XX)
6 months	XX (XX)	XX (XX)	XX (XX)	XX (XX)	XX (XX)	XX (XX)
Included in analysis †	XX (XX)		XX (XX)		XX (XX)	
**SF-36: General Health**						
Baseline	XX (XX)	XX (XX)	XX (XX)	XX (XX)	XX (XX)	XX (XX)
60 days	XX (XX)	XX (XX)	XX (XX)	XX (XX)	XX (XX)	XX (XX)
3 months	XX (XX)	XX (XX)	XX (XX)	XX (XX)	XX (XX)	XX (XX)
6 months	XX (XX)	XX (XX)	XX (XX)	XX (XX)	XX (XX)	XX (XX)
Included in analysis †	XX (XX)		XX (XX)		XX (XX)	
**SF-36: Social Functioning**						
Baseline	XX (XX)	XX (XX)	XX (XX)	XX (XX)	XX (XX)	XX (XX)
60 days	XX (XX)	XX (XX)	XX (XX)	XX (XX)	XX (XX)	XX (XX)
3 months	XX (XX)	XX (XX)	XX (XX)	XX (XX)	XX (XX)	XX (XX)
6 months	XX (XX)	XX (XX)	XX (XX)	XX (XX)	XX (XX)	XX (XX)
Included in analysis †	XX (XX)		XX (XX)		XX (XX)	

(† Included in analysis if outcome is available for at least one follow-up time point.)

For peer review only - http://bmjopen.bmj.com/site/about/guidelines.xhtml

Barts and The London School of Medicine and Dentistry

PCTU

	Intervention vs. Active control Adjusted mean difference (95% CI)	Invention vs. Usual care Adjusted mean difference (95% CI)	Active control vs. Usual care Adjusted mean difference (95% CI)
**Pain acceptance score**			
60 days	XX (XX to XX)	XX (XX to XX)	XX (XX to XX)
3 months	XX (XX to XX)	XX (XX to XX)	XX (XX to XX)
6 months	XX (XX to XX)	XX (XX to XX)	XX (XX to XX)
**Depression score**			
60 days	XX (XX to XX)	XX (XX to XX)	XX (XX to XX)
3 months	XX (XX to XX)	XX (XX to XX)	XX (XX to XX)
6 months	XX (XX to XX)	XX (XX to XX)	XX (XX to XX)
**Anxiety score**			
60 days	XX (XX to XX)	XX (XX to XX)	XX (XX to XX)
3 months	XX (XX to XX)	XX (XX to XX)	XX (XX to XX)
6 months	XX (XX to XX)	XX (XX to XX)	XX (XX to XX)
**Mindfulness score**			
60 days	XX (XX to XX)	XX (XX to XX)	XX (XX to XX)
3 months	XX (XX to XX)	XX (XX to XX)	XX (XX to XX)
6 months	XX (XX to XX)	XX (XX to XX)	XX (XX to XX)
**Pain related disability score**			
60 days	XX (XX to XX)	XX (XX to XX)	XX (XX to XX)
3 months	XX (XX to XX)	XX (XX to XX)	XX (XX to XX)
6 months	XX (XX to XX)	XX (XX to XX)	XX (XX to XX)
**Self efficacy score**			
60 days	XX (XX to XX)	XX (XX to XX)	XX (XX to XX)
3 months	XX (XX to XX)	XX (XX to XX)	XX (XX to XX)
6 months	XX (XX to XX)	XX (XX to XX)	XX (XX to XX)
**SHOW-Q global score, for sexually active participants**			
60 days	XX (XX to XX)	XX (XX to XX)	XX (XX to XX)
3 months	XX (XX to XX)	XX (XX to XX)	XX (XX to XX)
6 months	XX (XX to XX)	XX (XX to XX)	XX (XX to XX)
**SHOW-Q pelvic problem interference score, for all participants**			
60 days	XX (XX to XX)	XX (XX to XX)	XX (XX to XX)
3 months	XX (XX to XX)	XX (XX to XX)	XX (XX to XX)

For peer review only - http://bmjopen.bmj.com/site/about/guidelines.xhtml

6 months	XX	(XX to XX)	XX	(XX to XX)	XX	(XX to XX)
**Subjective outcome score**						
60 days	XX	(XX to XX)	XX	(XX to XX)	XX	(XX to XX)
3 months	XX	(XX to XX)	XX	(XX to XX)	XX	(XX to XX)
6 months	XX	(XX to XX)	XX	(XX to XX)	XX	(XX to XX)
**SF-36: Physical Functioning**						
60 days	XX	(XX to XX)	XX	(XX to XX)	XX	(XX to XX)
3 months	XX	(XX to XX)	XX	(XX to XX)	XX	(XX to XX)
6 months	XX	(XX to XX)	XX	(XX to XX)	XX	(XX to XX)
**SF-36: Pain**						
60 days	XX	(XX to XX)	XX	(XX to XX)	XX	(XX to XX)
3 months	XX	(XX to XX)	XX	(XX to XX)	XX	(XX to XX)
6 months	XX	(XX to XX)	XX	(XX to XX)	XX	(XX to XX)
**SF-36: General Health**						
60 days	XX	(XX to XX)	XX	(XX to XX)	XX	(XX to XX)
3 months	XX	(XX to XX)	XX	(XX to XX)	XX	(XX to XX)
6 months	XX	(XX to XX)	XX	(XX to XX)	XX	(XX to XX)
**SF-36: Social Functioning**						
60 days	XX	(XX to XX)	XX	(XX to XX)	XX	(XX to XX)
3 months	XX	(XX to XX)	XX	(XX to XX)	XX	(XX to XX)
6 months	XX	(XX to XX)	XX	(XX to XX)	XX	(XX to XX)

12.1.10. Unintentional unblinding of randomised treatment

Figures are number (%) unless stated otherwise.

	Intervention (n=…)		Active control (n=…)	
Researchers: Which app treatment do you believe the participant was randomised to?				
Intervention app	XX	(XX)	XX	(XX)
Control app	XX	(XX)	XX	(XX)
Don’t know	XX	(XX)	XX	(XX)
Participants: Do you think you received the new treatment or comparison treatment?				
New treatment	XX	(XX)	XX	(XX)
Comparison treatment	XX	(XX)	XX	(XX)
Don’t know	XX	(XX)	XX	(XX)

For peer review only - http://bmjopen.bmj.com/site/about/guidelines.xhtml

Barts and The London School of Medicine and Dentistry PCTU

12.1.11. Partially missing clinical outcomes

	Not completed *		Partially Completed **		Fully completed ***	
	n	(%)	n	(%)	n	(%)
**Pain acceptance score**						
Baseline	XX	(XX)	XX	(XX)	XX	(XX)
60 days	XX	(XX)	XX	(XX)	XX	(XX)
3 months	XX	(XX)	XX	(XX)	XX	(XX)
6 months	XX	(XX)	XX	(XX)	XX	(XX)
**Depression score**						
Baseline	XX	(XX)	XX	(XX)	XX	(XX)
60 days	XX	(XX)	XX	(XX)	XX	(XX)
3 months	XX	(XX)	XX	(XX)	XX	(XX)
6 months	XX	(XX)	XX	(XX)	XX	(XX)
**Anxiety score**						
Baseline	XX	(XX)	XX	(XX)	XX	(XX)
60 days	XX	(XX)	XX	(XX)	XX	(XX)
3 months	XX	(XX)	XX	(XX)	XX	(XX)
6 months	XX	(XX)	XX	(XX)	XX	(XX)
**Mindfulness score**						
Baseline	XX	(XX)	XX	(XX)	XX	(XX)
60 days	XX	(XX)	XX	(XX)	XX	(XX)
3 months	XX	(XX)	XX	(XX)	XX	(XX)
6 months	XX	(XX)	XX	(XX)	XX	(XX)
**Pain related disability score**						
Baseline	XX	(XX)	XX	(XX)	XX	(XX)
60 days	XX	(XX)	XX	(XX)	XX	(XX)
3 months	XX	(XX)	XX	(XX)	XX	(XX)
6 months	XX	(XX)	XX	(XX)	XX	(XX)
**Self efficacy score**						
Baseline	XX	(XX)	XX	(XX)	XX	(XX)
60 days	XX	(XX)	XX	(XX)	XX	(XX)
3 months	XX	(XX)	XX	(XX)	XX	(XX)
6 months	XX	(XX)	XX	(XX)	XX	(XX)

For peer review only - http://bmjopen.bmj.com/site/about/guidelines.xhtml

Barts and The London School of Medicine and Dentistry

PCTU

**SHOW-Q global score, for sexually active participants**			
Baseline	XX (XX)	XX (XX)	XX (XX)
60 days	XX (XX)	XX (XX)	XX (XX)
3 months	XX (XX)	XX (XX)	XX (XX)
6 months	XX (XX)	XX (XX)	XX (XX)
**SHOW-Q pelvic problem interference score, for all participants**			
Baseline	XX (XX)	XX (XX)	XX (XX)
60 days	XX (XX)	XX (XX)	XX (XX)
3 months	XX (XX)	XX (XX)	XX (XX)
6 months	XX (XX)	XX (XX)	XX (XX)
**Subjective outcome score**			
Baseline	XX (XX)	XX (XX)	XX (XX)
60 days	XX (XX)	XX (XX)	XX (XX)
3 months	XX (XX)	XX (XX)	XX (XX)
6 months	XX (XX)	XX (XX)	XX (XX)
**SF-36: Physical Functioning**			
Baseline	XX (XX)	XX (XX)	XX (XX)
60 days	XX (XX)	XX (XX)	XX (XX)
3 months	XX (XX)	XX (XX)	XX (XX)
6 months	XX (XX)	XX (XX)	XX (XX)
**SF-36: Pain**			
Baseline	XX (XX)	XX (XX)	XX (XX)
60 days	XX (XX)	XX (XX)	XX (XX)
3 months	XX (XX)	XX (XX)	XX (XX)
6 months	XX (XX)	XX (XX)	XX (XX)
**SF-36: General Health**			
Baseline	XX (XX)	XX (XX)	XX (XX)
60 days	XX (XX)	XX (XX)	XX (XX)
3 months	XX (XX)	XX (XX)	XX (XX)
6 months	XX (XX)	XX (XX)	XX (XX)
**SF-36: Social Functioning**			
Baseline	XX (XX)	XX (XX)	XX (XX)
60 days	XX (XX)	XX (XX)	XX (XX)
3 months	XX (XX)	XX (XX)	XX (XX)
6 months	XX (XX)	XX (XX)	XX (XX)

For peer review only - http://bmjopen.bmj.com/site/about/guidelines.xhtml

* Questionnaire not answered or all variables used in the derivation of the outcome are missing.
** One or more, but not all, variables used in the derivation of the outcome are missing.
*** No variables used in the derivation of the outcome are missing.

For peer review only - http://bmjopen.bmj.com/site/about/guidelines.xhtml

Barts and The London School of Medicine and Dentistry

PCTU

			Questionnaire answered by telephone						Questionnaire returned					
	Questionnaire never returned		Not completed †		Partially completed ††		Fully completed †††		Not completed †		Partially completed ††		Fully completed †††	
	n	(%)	n	(%)	n	(%)	n	(%)	n	(%)	n	(%)	n	(%)
**Pain acceptance score**														
Baseline	XX	(XX)	n/a		n/a		n/a		XX	(XX)	XX	(XX)	XX	(XX)
60 days	XX	(XX)	XX	(XX)	XX	(XX)	XX	(XX)	XX	(XX)	XX	(XX)	XX	(XX)
3 months	XX	(XX)	XX	(XX)	XX	(XX)	XX	(XX)	XX	(XX)	XX	(XX)	XX	(XX)
6 months	XX	(XX)	XX	(XX)	XX	(XX)	XX	(XX)	XX	(XX)	XX	(XX)	XX	(XX)
**Depression score**														
Baseline	XX	(XX)	n/a		n/a		n/a		XX	(XX)	XX	(XX)	XX	(XX)
60 days	XX	(XX)	XX	(XX)	XX	(XX)	XX	(XX)	XX	(XX)	XX	(XX)	XX	(XX)
3 months	XX	(XX)	XX	(XX)	XX	(XX)	XX	(XX)	XX	(XX)	XX	(XX)	XX	(XX)
6 months	XX	(XX)	XX	(XX)	XX	(XX)	XX	(XX)	XX	(XX)	XX	(XX)	XX	(XX)
**Anxiety score**														
Baseline	XX	(XX)	n/a		n/a		n/a		XX	(XX)	XX	(XX)	XX	(XX)
60 days	XX	(XX)	XX	(XX)	XX	(XX)	XX	(XX)	XX	(XX)	XX	(XX)	XX	(XX)
3 months	XX	(XX)	XX	(XX)	XX	(XX)	XX	(XX)	XX	(XX)	XX	(XX)	XX	(XX)
6 months	XX	(XX)	XX	(XX)	XX	(XX)	XX	(XX)	XX	(XX)	XX	(XX)	XX	(XX)
**Mindfulness score**														
Baseline	XX	(XX)	n/a		n/a		n/a		XX	(XX)	XX	(XX)	XX	(XX)

For peer review only - http://bmjopen.bmj.com/site/about/guidelines.xhtml

60 days	XX (XX)	XX (XX)	XX (XX)	XX (XX)	XX (XX)	XX (XX)	XX (XX)
3 months	XX (XX)	XX (XX)	XX (XX)	XX (XX)	XX (XX)	XX (XX)	XX (XX)
6 months	XX (XX)	XX (XX)	XX (XX)	XX (XX)	XX (XX)	XX (XX)	XX (XX)
**Pain related disability score**							
Baseline	XX (XX)	n/a	n/a	n/a	XX (XX)	XX (XX)	XX (XX)
60 days	XX (XX)	XX (XX)	XX (XX)	XX (XX)	XX (XX)	XX (XX)	XX (XX)
3 months	XX (XX)	XX (XX)	XX (XX)	XX (XX)	XX (XX)	XX (XX)	XX (XX)
6 months	XX (XX)	XX (XX)	XX (XX)	XX (XX)	XX (XX)	XX (XX)	XX (XX)
**Self efficacy score**							
Baseline	XX (XX)	n/a	n/a	n/a	XX (XX)	XX (XX)	XX (XX)
60 days	XX (XX)	n/a	n/a	n/a	XX (XX)	XX (XX)	XX (XX)
3 months	XX (XX)	n/a	n/a	n/a	XX (XX)	XX (XX)	XX (XX)
6 months	XX (XX)	n/a	n/a	n/a	XX (XX)	XX (XX)	XX (XX)
**SHOW-Q global score, for sexually active participants**							
Baseline	XX (XX)	n/a	n/a	n/a	XX (XX)	XX (XX)	XX (XX)
60 days	XX (XX)	n/a	n/a	n/a	XX (XX)	XX (XX)	XX (XX)
3 months	XX (XX)	n/a	n/a	n/a	XX (XX)	XX (XX)	XX (XX)
6 months	XX (XX)	n/a	n/a	n/a	XX (XX)	XX (XX)	XX (XX)
**SHOW-Q pelvic problem interference score, for all participants**							
Baseline	XX (XX)	n/a	n/a	n/a	XX (XX)	XX (XX)	XX (XX)
60 days	XX (XX)	n/a	n/a	n/a	XX (XX)	XX (XX)	XX (XX)
3 months	XX (XX)	n/a	n/a	n/a	XX (XX)	XX (XX)	XX (XX)

For peer review only - http://bmjopen.bmj.com/site/about/guidelines.xhtml

6 months	XX (XX)	n/a	n/a	n/a	XX (XX)	XX (XX)	XX (XX)
**Subjective outcome score**							
Baseline	XX (XX)	n/a	n/a	n/a	XX (XX)	XX (XX)	XX (XX)
60 days	XX (XX)	XX (XX)	XX (XX)	XX (XX)	XX (XX)	XX (XX)	XX (XX)
3 months	XX (XX)	XX (XX)	XX (XX)	XX (XX)	XX (XX)	XX (XX)	XX (XX)
6 months	XX (XX)	XX (XX)	XX (XX)	XX (XX)	XX (XX)	XX (XX)	XX (XX)
**SF-36: Physical Functioning**							
Baseline	XX (XX)	n/a	n/a	n/a	XX (XX)	XX (XX)	XX (XX)
60 days	XX (XX)	n/a	n/a	n/a	XX (XX)	XX (XX)	XX (XX)
3 months	XX (XX)	n/a	n/a	n/a	XX (XX)	XX (XX)	XX (XX)
6 months	XX (XX)	n/a	n/a	n/a	XX (XX)	XX (XX)	XX (XX)
**SF-36: Pain**							
Baseline	XX (XX)	n/a	n/a	n/a	XX (XX)	XX (XX)	XX (XX)
60 days	XX (XX)	n/a	n/a	n/a	XX (XX)	XX (XX)	XX (XX)
3 months	XX (XX)	n/a	n/a	n/a	XX (XX)	XX (XX)	XX (XX)
6 months	XX (XX)	n/a	n/a	n/a	XX (XX)	XX (XX)	XX (XX)
**SF-36: General Health**							
Baseline	XX (XX)	n/a	n/a	n/a	XX (XX)	XX (XX)	XX (XX)
60 days	XX (XX)	n/a	n/a	n/a	XX (XX)	XX (XX)	XX (XX)
3 months	XX (XX)	n/a	n/a	n/a	XX (XX)	XX (XX)	XX (XX)
6 months	XX (XX)	n/a	n/a	n/a	XX (XX)	XX (XX)	XX (XX)
**SF-36: Social Functioning**							

For peer review only - http://bmjopen.bmj.com/site/about/guidelines.xhtml

Baseline	XX (XX)	n/a	n/a	n/a	XX (XX)	XX (XX)	XX (XX)
60 days	XX (XX)	n/a	n/a	n/a	XX (XX)	XX (XX)	XX (XX)
3 months	XX (XX)	n/a	n/a	n/a	XX (XX)	XX (XX)	XX (XX)
6 months	XX (XX)	n/a	n/a	n/a	XX (XX)	XX (XX)	XX (XX)

† Questionnaire answered, but all variables used in the derivation of the outcome are missing.
†† One or more, but not all, variables used in the derivation of the outcome are missing.
††† No variables used in the derivation of the outcome are missing.

For peer review only - http://bmjopen.bmj.com/site/about/guidelines.xhtml

Barts and The London School of Medicine and Dentistry PCTU

13. APPENDIX E: DATA / FILE MANAGEMENT

13.1.1. Sources of data

Copies of CRFs are included in the Statistics Master File. Data is entered from these into a PCTU database. Extracts from the database are supplied by the data manager onto a secure environment.

App usage data will be received from Headspace.

13.1.2. Programming plan

The trial folder on secure environment will contain a folder for each analysis.

An analysis folder should contain the following folders (and their contents):

- analysis data (saved Stata data files for analysis)
- do files (Stata do files for data preparation and analysis)
- log files (Stata log files)
- output (any files output e.g. produced tables and graphs)
- raw data (data as extracted from database)
- temp (any temporary files needed during data preparation or analysis)

Folders containing do files should include a text directory explaining the role of each do file.

13.1.3. Data dictionary

Field names specified in the database "Requirements Specification Document" will be the variable names in the data files. Where a variable is collect on more than one occasion, suffixes will be added to variables names (e.g. "_BASELINE", "_60DAYS", "_3MONTHS", "_6MONTHS").

Details of derived variables are given in Section 5, APPENDIX A: DERIVED AND COMPUTED VARIABLES, and APPENDIX B: STATA CODE FOR GENERATING ADHERENCE OUTCOMES.

A complete data dictionary will be produced for the final analysis data set.

For peer review only - http://bmjopen.bmj.com/site/about/guidelines.xhtml

Barts and The London
School of Medicine and Dentistry

PCTU

Appendix 1: Supplementary tables

TABLE OF CONTENTS

Table 1. Prior and concurrent treatment.....3
Table 2. Baseline values of clinical outcomes.....4
Table 3. Baseline demographics of woman by 6 month questionnaire completion.....5
1. FEASIBILITY OUTCOMES.....**7**
1.1. FOLLOW-UP.....7
Table 4. Losses to follow up.....7
Figure 1. Figure 1: Proportion of participants answering follow up questionnaire.....8
Table 5. Follow-up questionnaire returned or answered by phone within target follow up period.....9
1.2. STANDARD DEVIATION OF CPAQ.....10
Table 6. Estimated standard deviation of CPAQ.....10
1.4. BLINDING.....11
Table 7. Unintentional unbinding of randomised treatment.....11
2. APP SATISFACTION QUESTIONNAIRES.....**12**
Table 8. System usability scale.....12
Table 9. App usability Questionnaire.....13
3. CLINICAL OUTCOMES.....**14**
3.1. RANGES OF CLINICAL OUTCOMES.....14
3.2. COMPLETENESS OF CLINICAL DATA.....15
Table 10. Partially missing clinical outcomes.....15
Table 11. Partially missing clinical outcomes by method of questionnaire delivery.....17
3.3. RESULTS OF ANALYSIS OF CLINICAL OUTCOMES.....20
Table 12. Descriptive statistics for clinical outcomes.....20
Table 13. Estimated treatment effects for clinical outcomes.....23

For peer review only - http://bmjopen.bmj.com/site/about/guidelines.xhtml

Figure 2. Figure 3: Estimated treatment effects and 95% confidence intervals for CPAQ......25

PCTU

For peer review only

For peer review only - http://bmjopen.bmj.com/site/about/guidelines.xhtml

Barts and The London School of Medicine and Dentistry

PCTU

Table 1. Prior and concurrent treatment

Figures are number (percentage).

	Summary measure			Missing data		
	Intervention (N=31)	Active control (N=30)	Usual care (N=29)	Intervention - no. (%)	Active control - no. (%)	Usual care - no. (%)
**Treatment used in the last six months**						
Acupuncture	2 (10.5)	5 (25.0)	1 (6.3)	12 (38.7)	10 (33.3)	13 (44.8)
Massage	11 (50.0)	8 (40.0)	7 (41.2)	9 (29.0)	10 (33.3)	12 (41.4)
Gabapentin	5 (26.3)	1 (5.9)	1 (6.3)	12 (38.7)	13 (43.3)	13 (44.8)
Nutrition/diet	14 (63.6)	14 (63.6)	18 (78.3)	9 (29.0)	8 (26.7)	6 (20.7)
Amitriptyline	5 (27.8)	4 (20.0)	4 (22.2)	13 (41.9)	10 (33.3)	11 (37.9)
Codeine or Morphine type painkillers	13 (56.5)	13 (59.1)	19 (76.0)	8 (25.8)	8 (26.7)	4 (13.8)
Biofeedback	0 (0.0)	0 (0.0)	0 (0.0)	13 (41.9)	12 (40.0)	13 (44.8)
Nerve blocks	0 (0.0)	2 (11.1)	0 (0.0)	14 (45.2)	12 (40.0)	12 (41.4)
Botox injection	0 (0.0)	0 (0.0)	0 (0.0)	14 (45.2)	13 (43.3)	13 (44.8)
Over the counter medication	17 (73.9)	9 (47.4)	17 (77.3)	8 (25.8)	11 (36.7)	7 (24.1)
Contraceptive pills/patch/ring	15 (68.2)	7 (36.8)	11 (52.4)	9 (29.0)	11 (36.7)	8 (27.6)
Physiotherapy	5 (26.3)	4 (20.0)	1 (6.7)	12 (38.7)	10 (33.3)	14 (48.3)
Exercise, yoga or Pilates	13 (59.1)	12 (60.0)	15 (78.9)	9 (29.0)	10 (33.3)	10 (34.5)
Psychological (talking) therapy	3 (16.7)	2 (11.1)	2 (13.3)	13 (41.9)	12 (40.0)	14 (48.3)
Injections to suppress ovaries (e.g. Prostap, Zoladex)	6 (33.3)	5 (25.0)	8 (38.1)	13 (41.9)	10 (33.3)	8 (27.6)
Transcutaneous Electrical Nerve Stimulation (TENS)	0 (0.0)	2 (11.1)	3 (17.6)	13 (41.9)	12 (40.0)	12 (41.4)
Herbal Medicine	4 (21.1)	5 (26.3)	8 (44.4)	12 (38.7)	11 (36.7)	11 (37.9)
Surgery	3 (16.7)	4 (23.5)	6 (31.6)	13 (41.9)	13 (43.3)	10 (34.5)
Meditation or relaxation exercises	11 (47.8)	7 (38.9)	10 (52.6)	8 (25.8)	12 (40.0)	10 (34.5)
Other	3 (37.5)	3 (33.3)	4 (44.4)	23 (74.2)	21 (70.0)	20 (69.0)
Currently using pain treatment				4 (12.9)	3 (10.0)	2 (6.9)
Yes	21 (77.8)	18 (66.7)	20 (74.1)			
No	6 (22.2)	9 (33.3)	7 (25.9)			

For peer review only - http://bmjopen.bmj.com/site/about/guidelines.xhtml

Barts and The London
School of Medicine and Dentistry

PCTU

Table 2. Baseline values of clinical outcomes

Figures are mean (SD)

	Summary measure			Missing data		
	**Intervention (N=31)**	**Active control (N=30)**	**Usual care (N=29)**	**Intervention - no. (%)**	**Active control - no. (%)**	**Usual care - no. (%)**
CPAQ pain acceptance score	21.9 (9.5)	22.7 (8.4)	23.8 (8.5)	2 (6.5)	3 (10.0)	1 (3.4)
HADS depression score	8.7 (5.1)	8.6 (5.0)	7.4 (3.6)	1 (3.2)	3 (10.0)	2 (6.9)
HADS anxiety score	12.6 (5.3)	12.0 (5.3)	10.9 (3.9)	1 (3.2)	4 (13.3)	1 (3.4)
CAMS-R mindfulness score	28.6 (6.1)	28.8 (7.1)	30.3 (5.4)	3 (9.7)	5 (16.7)	3 (10.3)
CPG disability score	60.6 (24.4)	64.6 (19.6)	59.2 (24.4)	1 (3.2)	3 (10.0)	1 (3.4)
PSEQ Self efficacy score	29.1 (14.7)	27.9 (14.6)	35.5 (10.6)	1 (3.2)	3 (10.0)	2 (6.9)
Sexual health outcomes:						
SHOW-Q global score*	45.4 (20.3)	50.9 (20.9)	58.1 (22.2)	5 (16.1)	7 (23.3)	3 (10.3)
SHOW-Q pelvic problem interference score	47.1 (29.0)	49.0 (32.7)	56.4 (25.9)	8 (25.8)	6 (20.0)	3 (10.3)
MYMOP subjective outcome score	4.1 (1.2)	3.9 (1.3)	3.9 (1.1)	1 (3.2)	3 (10.0)	2 (6.9)
SF-36 Scales:						
SF36 - Physical functioning	56.3 (30.2)	55.8 (32.2)	66.5 (30.4)	3 (9.7)	4 (13.3)	2 (6.9)
SF36 - Pain	35.1 (17.5)	34.7 (20.6)	37.6 (20.6)	1 (3.2)	3 (10.0)	1 (3.4)
SF36 - General Health	39.1 (20.3)	42.0 (19.8)	37.9 (21.4)	2 (6.5)	3 (10.0)	1 (3.4)
SF36 - Social functioning	37.5 (19.1)	38.0 (28.3)	50.4 (25.3)	1 (3.2)	3 (10.0)	1 (3.4)

*Show-Q global is only applicable for sexually active participants. At baseline there are 17 sexually active women in the intervention group, 22 in the active control group and 19 in the usual care group.

For peer review only - http://bmjopen.bmj.com/site/about/guidelines.xhtml

Table 3. Baseline demographics of woman by 6 month questionnaire completion

Figures are mean (SD) unless stated otherwise.

	6 month follow-up questionnaire returned (N=33)	6 month follow-up questionnaire answered by phone (N=24)	6 month follow-up questionnaire never returned (N=33)
**Demographics**			
Age (Years)	35.8 (8.0)	36.6 (9.2)	33.1 (7.5)
Body mass index (kg/m^2)	27.4 (7.1)	27.7 (6.5)	25.9 (4.5)
Living arrangements - no. (%)			
Alone	2 (6.3)	1 (4.2)	3 (10.7)
With others	30 (93.8)	23 (95.8)	25 (89.3)
Employment status - no. (%)			
Employed	26 (78.8)	13 (54.2)	17 (60.7)
Unemployed and looking for work	1 (3.0)	1 (4.2)	1 (3.6)
At school or in full time education	1 (3.0)	2 (8.3)	4 (14.3)
Unable to work due to long term sickness	3 (9.1)	4 (16.7)	3 (10.7)
Looking after your home/family	2 (6.1)	3 (12.5)	3 (10.7)
Retired from paid work	0 (0.0)	1 (4.2)	0 (0.0)
Age left full time education - no. (%)			
Age 12 or less	0 (0.0)	3 (12.5)	0 (0.0)
Age 13 to 16	2 (6.1)	6 (25.0)	8 (29.6)
Age 17 to 19	7 (21.2)	2 (8.3)	5 (18.5)
Age 20 or over	23 (69.7)	9 (37.5)	10 (37.0)
Still in education	1 (3.0)	4 (16.7)	4 (14.8)
Ethnic group - no. (%)			
White	18 (58.1)	9 (40.9)	8 (30.8)
Black	7 (22.6)	4 (18.2)	2 (7.7)
Central Asian	0 (0.0)	0 (0.0)	2 (7.7)
Middle Eastern	0 (0.0)	0 (0.0)	1 (3.8)
Southern Asian	5 (16.1)	6 (27.3)	7 (26.9)
Mixed	1 (3.2)	0 (0.0)	1 (3.8)
Other ethnic group	0 (0.0)	2 (9.1)	4 (15.4)
Do not wish to say	0 (0.0)	1 (4.5)	1 (3.8)

For peer review only - http://bmjopen.bmj.com/site/about/guidelines.xhtml

Barts and The London School of Medicine and Dentistry PCTU

	6 month follow-up questionnaire returned (N=33)	6 month follow-up questionnaire answered by phone (N=24)	6 month follow-up questionnaire never returned (N=33)
Smoker - no. (%)			
Yes	6 (18.8)	4 (18.2)	7 (25.9)
No	26 (81.3)	18 (81.8)	20 (74.1)
If yes, number of cigarettes per week	36.0 (24.1)	15.3 (12.5)	44.0 (30.8)
Drink alcohol - no. (%)			
Yes	18 (56.3)	6 (27.3)	10 (37.0)
No	14 (43.8)	16 (72.7)	17 (63.0)
If yes, number of units per week	8.9 (7.2)	5.8 (5.3)	5.2 (2.9)
**Baseline medical history**			
Duration of pain - no. (%)			
0 to 6 months	0 (0.0)	1 (4.2)	1 (3.6)
7 to 12 months	3 (9.1)	0 (0.0)	5 (17.9)
1 to 2 years	6 (18.2)	3 (12.5)	4 (14.3)
3 to 5 years	10 (30.3)	10 (41.7)	6 (21.4)
6 to 10 years	5 (15.2)	3 (12.5)	3 (10.7)
More than 10 years	9 (27.3)	7 (29.2)	9 (32.1)
Pain over the past week	6.0 (2.5)	6.0 (2.6)	7.5 (2.2)
**Baseline values of clinical outcomes**			
CPAQ pain acceptance score	25.3 (8.4)	20.8 (8.8)	21.4 (8.7)
HADS depression score	6.6 (3.6)	8.5 (4.9)	10.0 (4.9)
HADS anxiety score	10.3 (4.7)	12.2 (5.1)	13.5 (4.5)
CPG disability score	54.5 (18.8)	65.4 (20.7)	66.0 (27.4)

For peer review only - http://bmjopen.bmj.com/site/about/guidelines.xhtml

Barts and The London
School of Medicine and Dentistry

PCTU

1. Feasibility Outcomes

1.1. Follow-up

Table 4. Losses to follow up

	Intervention (N=31)	Active control (N=30)	Usual care (N=29)
**Follow-up questionnaire returned - no (%)**			
60 days	15 (48.4)	9 (30.0)	18 (62.1)
3 months	17 (54.8)	12 (40.0)	17 (58.6)
6 months	11 (35.5)	10 (33.3)	12 (41.4)
**Follow-up questionnaire answered by phone - no (%)**			
60 days	1 (3.2)	8 (26.7)	1 (3.4)
3 months	3 (9.7)	7 (23.3)	3 (10.3)
6 months	10 (32.3)	6 (20.0)	8 (27.6)
**Follow-up questionnaire never returned - no (%)**			
60 days	15 (48.4)	13 (43.3)	10 (34.5)
3 months	11 (35.5)	11 (36.7)	9 (31.0)
6 months	10 (32.3)	14 (46.7)	9 (31.0)

For peer review only - http://bmjopen.bmj.com/site/about/guidelines.xhtml

Figure 1. *Figure 1: Proportion of participants answering follow up questionnaire*

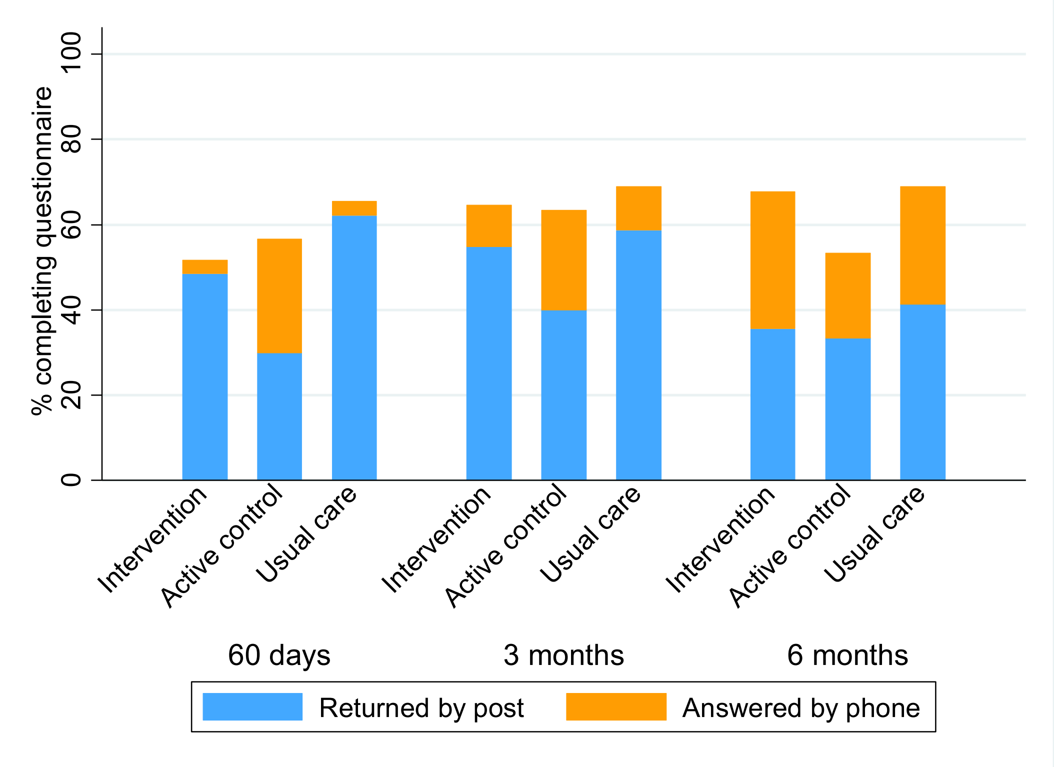

For peer review only - http://bmjopen.bmj.com/site/about/guidelines.xhtml

Barts and The London
School of Medicine and Dentistry

PCTU

Table 5. Follow-up questionnaire returned or answered by phone within target follow up period

Figures are no returning data on time/no. returning data questionnaire answering by phone (%)*.

	Intervention (N=31)	Active control (N=30)	Usual care (N=29)
60 days (47 and 74 days)	7/16 (43.8)	6/17 (35.3)	11/19 (57.9)
3 months (76 and 104 days)	7/20 (35.0)	6/19 (31.6)	11/20 (55.0)
6 months (159 and 201 days)	7/21 (33.3)	6/16 (37.5)	11/20 (55.0)

*Denominator for percentage is number returning data questionnaire answering by phone

For peer review only - http://bmjopen.bmj.com/site/about/guidelines.xhtml

Barts and The London
School of Medicine and Dentistry

PCTU

1.2. Standard deviation of CPAQ

Table 6. Estimated standard deviation of CPAQ

	Number with complete outcome	Estimated standard deviation
60 days	50	9.6
3 months	55	8.1
6 months	56	9.6

For peer review only - http://bmjopen.bmj.com/site/about/guidelines.xhtml

Barts and The London School of Medicine and Dentistry

PCTU

1.4. Blinding

Table 7. Unintentional unbinding of randomised treatment

Figures are number (%)

	Summary measure		Missing data	
	**Intervention (N=31)**	**Active control (N=30)**	**Intervention - no. (%)**	**Active control - no. (%)**
Researchers: Which app treatment do you believe the participant was randomised to?			2 (6.5)	3 (10.0)
Intervention app	0 (0.0)	1 (3.7)		
Control app	0 (0.0)	0 (0.0)		
Don't know	29 (100.0)	26 (96.3)		
Participants: Do you think you received the new treatment or comparison treatment?			15 (48.4)	19 (63.3)
New treatment	1 (6.3)	1 (9.1)		
Comparison treatment	0 (0.0)	1 (9.1)		
Don't know	15 (93.8)	9 (81.8)		

For peer review only - http://bmjopen.bmj.com/site/about/guidelines.xhtml

Barts and The London School of Medicine and Dentistry

PCTU

2. App satisfaction questionnaires

Table 8. System usability scale

Figures are mean (sd).

	**Summary measure**		**Missing Data**	
	Intervention (N=31)	Active control (N=30)	Intervention - no. (%)	Active control - no. (%)
System usability scale	50.7 (6.6)	46.0 (12.0)	16 (51.6)	18 (60.0)

For peer review only - http://bmjopen.bmj.com/site/about/guidelines.xhtml

Barts and The London
School of Medicine and Dentistry

PCTU

Table 9. App usability Questionnaire

Figures are number (%).

	Totally disagree	Somewhat disagree	Neither agree nor disagree	Somewhat agree	Totally agree	Not answered
It is easy to use the app whenever I wanted to use it						
Intervention:	0 (0.0)	3 (9.7)	1 (3.2)	3 (9.7)	9 (29.0)	15 (48.4)
Active control:	0 (0.0)	0 (0.0)	0 (0.0)	4 (13.3)	8 (26.7)	18 (60.0)
After being shown, I understood how the app would work						
Intervention:	0 (0.0)	1 (3.2)	1 (3.2)	4 (12.9)	9 (29.0)	16 (51.6)
Active control:	0 (0.0)	2 (6.7)	0 (0.0)	2 (6.7)	8 (26.7)	18 (60.0)
It was fun to work with the app						
Intervention:	0 (0.0)	2 (6.5)	3 (9.7)	8 (25.8)	2 (6.5)	16 (51.6)
Active control:	0 (0.0)	3 (10.0)	3 (10.0)	5 (16.7)	1 (3.3)	18 (60.0)
The app worked well						
Intervention:	0 (0.0)	3 (9.7)	2 (6.5)	5 (16.1)	5 (16.1)	16 (51.6)
Active control:	0 (0.0)	2 (6.7)	2 (6.7)	4 (13.3)	4 (13.3)	18 (60.0)
It was easy to work through the modules						
Intervention:	0 (0.0)	4 (12.9)	0 (0.0)	5 (16.1)	6 (19.4)	16 (51.6)
Active control:	0 (0.0)	2 (6.7)	3 (10.0)	4 (13.3)	3 (10.0)	18 (60.0)
The number of modules was annoying						
Intervention:	1 (3.2)	4 (12.9)	6 (19.4)	5 (16.1)	0 (0.0)	15 (48.4)
Active control:	2 (6.7)	3 (10.0)	3 (10.0)	2 (6.7)	2 (6.7)	18 (60.0)
The modules were well-displayed on my smartphone						
Intervention:	0 (0.0)	2 (6.5)	1 (3.2)	8 (25.8)	5 (16.1)	15 (48.4)
Active control:	0 (0.0)	0 (0.0)	2 (6.7)	2 (6.7)	7 (23.3)	19 (63.3)
Using the app was difficult because of my daily activities						
Intervention:	2 (6.5)	2 (6.5)	3 (9.7)	9 (29.0)	0 (0.0)	15 (48.4)
Active control:	1 (3.3)	1 (3.3)	0 (0.0)	5 (16.7)	5 (16.7)	18 (60.0)
Using the app took too long						
Intervention:	2 (6.5)	4 (12.9)	3 (9.7)	7 (22.6)	0 (0.0)	15 (48.4)
Active control:	1 (3.3)	3 (10.0)	2 (6.7)	4 (13.3)	2 (6.7)	18 (60.0)

For peer review only - http://bmjopen.bmj.com/site/about/guidelines.xhtml

Barts and The London School of Medicine and Dentistry

PCTU

3. Clinical Outcomes

3.1. Ranges of clinical outcomes

Pain acceptance score (as measured by the Chronic Pain Acceptance Questionnaire (CPAQ)):

- 0 (worst) – 48 (best)

Depression score (Hospital Anxiety and Depression Scale (HADS)):

- 0 (best) – 21 (worst)

Anxiety score (measured by HADS):

- 0 (best) – 21 (worst)

Mindfulness score (Cognitive and Mindfulness - Revised (CAMS – R) scale):

- 12 (worst) – 48 (best)

Pain related disability score (as measured by the Chronic Pain Grade (CPG) disability subscale):

- 0 (best) – 100 (worst)

Self efficacy score (as measured by the Pain Self-Efficacy Questionnaire (PSEQ)):

- 0 (worst) – 60 (best)

Sexual Health Outcomes scores (as measured by the Sexual Health Outcomes in Women Questionnaire (SHOW-Q))

- SHOW-Q global score, for sexually active participants: 0 (worst) – 100 (best)
- SHOW-Q pelvic interference score, for all participants: 0 (best) – 100 (worst)

Subjective outcome score (as measured by the Measure Yourself Medical Outcome Profile (MYMOP)):

- 0 (best) – 6)worst)

RAND Short form (36) Health Survey (RAND SF-36) scales:

- Physical functioning: 0 (worst) – 100)best)
- Pain: 0 (worst) – 100 (best)
- General health: 0 (worst) – 100 (best)
- Social functioning: 0 (worst) – 100 (best)

For peer review only - http://bmjopen.bmj.com/site/about/guidelines.xhtml

Barts and The London School of Medicine and Dentistry PCTU

3.2. Completeness of clinical data

Table 10. Partially missing clinical outcomes

Figures are number (%)

	Not completed* no. (%)	Partially completed** no. (%)	Fully completed*** no. (%)
**CPAQ pain acceptance score**			
Baseline	5 (5.6)	1 (1.1)	84 (93.3)
60 days	40 (44.4)	0 (0.0)	50 (55.6)
3 months	32 (35.6)	3 (3.3)	55 (61.1)
6 months	34 (37.8)	0 (0.0)	56 (62.2)
**HADS depression score**			
Baseline	5 (5.6)	1 (1.1)	84 (93.3)
60 days	40 (44.4)	1 (1.1)	49 (54.4)
3 months	32 (35.6)	0 (0.0)	58 (64.4)
6 months	33 (36.7)	0 (0.0)	57 (63.3)
**HADS anxiety score**			
Baseline	5 (5.6)	1 (1.1)	84 (93.3)
60 days	40 (44.4)	0 (0.0)	50 (55.6)
3 months	32 (35.6)	0 (0.0)	58 (64.4)
6 months	33 (36.7)	0 (0.0)	57 (63.3)
**CAMS-R mindfulness score**			
Baseline	5 (5.6)	6 (6.7)	79 (87.8)
60 days	40 (44.4)	0 (0.0)	50 (55.6)
3 months	32 (35.6)	2 (2.2)	56 (62.2)
6 months	33 (36.7)	0 (0.0)	57 (63.3)
**CPG disability score**			
Baseline	5 (5.6)	0 (0.0)	85 (94.4)
60 days	40 (44.4)	0 (0.0)	50 (55.6)
3 months	33 (36.7)	0 (0.0)	57 (63.3)
6 months	34 (37.8)	0 (0.0)	56 (62.2)
**PSEQ Self efficacy score**			
Baseline	5 (5.6)	1 (1.1)	84 (93.3)
60 days	50 (55.6)	1 (1.1)	39 (43.3)
3 months	45 (50.0)	0 (0.0)	45 (50.0)
6 months	57 (63.3)	0 (0.0)	33 (36.7)

* Questionnaire not answered or all variables used in the derivation of the outcome are missing.
** One or more, but not all, variables used in the derivation of the outcome are missing.
*** No variables used in the derivation of the outcome are missing.

For peer review only - http://bmjopen.bmj.com/site/about/guidelines.xhtml

Barts and The London
School of Medicine and Dentistry

PCTU

	Not completed* no. (%)	Partially completed** no. (%)	Fully completed*** no. (%)
**SHOW-Q global score**			
Baseline	5 (5.6)	15 (16.7)	70 (77.8)
60 days	50 (55.6)	6 (6.7)	34 (37.8)
3 months	47 (52.2)	8 (8.9)	35 (38.9)
6 months	58 (64.4)	5 (5.6)	27 (30.0)
**SHOW-Q pelvic problem interference score**			
Baseline	9 (10.0)	8 (8.9)	73 (81.1)
60 days	51 (56.7)	3 (3.3)	36 (40.0)
3 months	49 (54.4)	3 (3.3)	38 (42.2)
6 months	60 (66.7)	1 (1.1)	29 (32.2)
**MYMOP subjective outcome score**			
Baseline	5 (5.6)	1 (1.1)	84 (93.3)
60 days	38 (42.2)	11 (12.2)	41 (45.6)
3 months	33 (36.7)	10 (11.1)	47 (52.2)
6 months	33 (36.7)	6 (6.7)	51 (56.7)
**SF36 - General Health**			
Baseline	5 (5.6)	1 (1.1)	84 (93.3)
60 days	38 (42.2)	11 (12.2)	41 (45.6)
3 months	31 (34.4)	14 (15.6)	45 (50.0)
6 months	33 (36.7)	24 (26.7)	33 (36.7)
**SF36 - Physical functioning**			
Baseline	5 (5.6)	4 (4.4)	81 (90.0)
60 days	48 (53.3)	3 (3.3)	39 (43.3)
3 months	45 (50.0)	2 (2.2)	43 (47.8)
6 months	57 (63.3)	3 (3.3)	30 (33.3)
**SF36 - Pain**			
Baseline	5 (5.6)	0 (0.0)	85 (94.4)
60 days	48 (53.3)	0 (0.0)	42 (46.7)
3 months	45 (50.0)	0 (0.0)	45 (50.0)
6 months	57 (63.3)	0 (0.0)	33 (36.7)
**SF36 - Social functioning**			
Baseline	5 (5.6)	0 (0.0)	85 (94.4)
60 days	48 (53.3)	1 (1.1)	41 (45.6)
3 months	45 (50.0)	1 (1.1)	44 (48.9)
6 months	57 (63.3)	1 (1.1)	32 (35.6)

* Questionnaire not answered or all variables used in the derivation of the outcome are missing.
** One or more, but not all, variables used in the derivation of the outcome are missing.
*** No variables used in the derivation of the outcome are missing.

For peer review only - http://bmjopen.bmj.com/site/about/guidelines.xhtml

Barts and The London School of Medicine and Dentistry
PCTU

Table 11. Partially missing clinical outcomes by method of questionnaire delivery

Figures are number (%)

	Questionnaire never returned no. (%)	Questionnaire answered by telephone			Questionnaire returned		
		Not completed* no. (%)	Partially completed** no. (%)	Fully completed*** no. (%)	Not completed* no. (%)	Partially completed** no. (%)	Fully completed*** no. (%)
**CPAQ pain acceptance score**							
Baseline	5 (5.6)	n/a	n/a	n/a	0 (0.0)	1 (1.1)	84 (93.3)
60 days	38 (42.2)	1 (1.1)	0 (0.0)	9 (10.0)	1 (1.1)	0 (0.0)	41 (45.6)
3 months	31 (34.4)	0 (0.0)	0 (0.0)	13 (14.4)	1 (1.1)	3 (3.3)	42 (46.7)
6 months	33 (36.7)	1 (1.1)	0 (0.0)	23 (25.6)	0 (0.0)	0 (0.0)	33 (36.7)
**HADS depression score**							
Baseline	5 (5.6)	n/a	n/a	n/a	0 (0.0)	1 (1.1)	84 (93.3)
60 days	38 (42.2)	1 (1.1)	0 (0.0)	9 (10.0)	1 (1.1)	1 (1.1)	40 (44.4)
3 months	31 (34.4)	0 (0.0)	0 (0.0)	13 (14.4)	1 (1.1)	0 (0.0)	45 (50.0)
6 months	33 (36.7)	0 (0.0)	0 (0.0)	24 (26.7)	0 (0.0)	0 (0.0)	33 (36.7)
**HADS anxiety score**							
Baseline	5 (5.6)	n/a	n/a	n/a	0 (0.0)	1 (1.1)	84 (93.3)
60 days	38 (42.2)	1 (1.1)	0 (0.0)	9 (10.0)	1 (1.1)	0 (0.0)	41 (45.6)
3 months	31 (34.4)	0 (0.0)	0 (0.0)	13 (14.4)	1 (1.1)	0 (0.0)	45 (50.0)
6 months	33 (36.7)	0 (0.0)	0 (0.0)	24 (26.7)	0 (0.0)	0 (0.0)	33 (36.7)
**CAMS-R mindfulness score**							
Baseline	5 (5.6)	n/a	n/a	n/a	0 (0.0)	6 (6.7)	79 (87.8)
60 days	38 (42.2)	1 (1.1)	0 (0.0)	9 (10.0)	1 (1.1)	0 (0.0)	41 (45.6)
3 months	31 (34.4)	0 (0.0)	0 (0.0)	13 (14.4)	1 (1.1)	2 (2.2)	43 (47.8)
6 months	33 (36.7)	0 (0.0)	0 (0.0)	24 (26.7)	0 (0.0)	0 (0.0)	33 (36.7)
**CPG disability score**							
Baseline	5 (5.6)	n/a	n/a	n/a	0 (0.0)	0 (0.0)	85 (94.4)
60 days	38 (42.2)	1 (1.1)	0 (0.0)	9 (10.0)	1 (1.1)	0 (0.0)	41 (45.6)
3 months	31 (34.4)	0 (0.0)	0 (0.0)	13 (14.4)	2 (2.2)	0 (0.0)	44 (48.9)
6 months	33 (36.7)	1 (1.1)	0 (0.0)	23 (25.6)	0 (0.0)	0 (0.0)	33 (36.7)

* Questionnaire not answered or all variables used in the derivation of the outcome are missing.
** One or more, but not all, variables used in the derivation of the outcome are missing.
*** No variables used in the derivation of the outcome are missing.

For peer review only - http://bmjopen.bmj.com/site/about/guidelines.xhtml

Barts and The London School of Medicine and Dentistry PCTU

	Questionnaire never returned no. (%)	Questionnaire answered by telephone			Questionnaire returned		
		Not completed* no. (%)	Partially completed** no. (%)	Fully completed*** no. (%)	Not completed* no. (%)	Partially completed** no. (%)	Fully completed*** no. (%)
**PSEQ Self efficacy score**							
Baseline	5 (5.6)	n/a	n/a	n/a	0 (0.0)	1 (1.1)	84 (93.3)
60 days	38 (42.2)	10 (11.1)	0 (0.0)	0 (0.0)	2 (2.2)	1 (1.1)	39 (43.3)
3 months	31 (34.4)	13 (14.4)	0 (0.0)	0 (0.0)	1 (1.1)	0 (0.0)	45 (50.0)
6 months	33 (36.7)	24 (26.7)	0 (0.0)	0 (0.0)	0 (0.0)	0 (0.0)	33 (36.7)
**SHOW-Q global score**							
Baseline	5 (5.6)	n/a	n/a	n/a	0 (0.0)	15 (16.7)	70 (77.8)
60 days	38 (42.2)	10 (11.1)	0 (0.0)	0 (0.0)	2 (2.2)	6 (6.7)	34 (37.8)
3 months	31 (34.4)	13 (14.4)	0 (0.0)	0 (0.0)	3 (3.3)	8 (8.9)	35 (38.9)
6 months	33 (36.7)	24 (26.7)	0 (0.0)	0 (0.0)	1 (1.1)	5 (5.6)	27 (30.0)
**SHOW-Q pelvic problem interference score**							
Baseline	5 (5.6)	n/a	n/a	n/a	4 (4.4)	8 (8.9)	73 (81.1)
60 days	38 (42.2)	10 (11.1)	0 (0.0)	0 (0.0)	3 (3.3)	3 (3.3)	36 (40.0)
3 months	31 (34.4)	13 (14.4)	0 (0.0)	0 (0.0)	5 (5.6)	3 (3.3)	38 (42.2)
6 months	33 (36.7)	24 (26.7)	0 (0.0)	0 (0.0)	3 (3.3)	1 (1.1)	29 (32.2)
**MYMOP subjective outcome score**							
Baseline	5 (5.6)	n/a	n/a	n/a	0 (0.0)	1 (1.1)	84 (93.3)
60 days	38 (42.2)	0 (0.0)	1 (1.1)	9 (10.0)	0 (0.0)	10 (11.1)	32 (35.6)
3 months	31 (34.4)	0 (0.0)	1 (1.1)	12 (13.3)	2 (2.2)	9 (10.0)	35 (38.9)
6 months	33 (36.7)	0 (0.0)	2 (2.2)	22 (24.4)	0 (0.0)	4 (4.4)	29 (32.2)

* Questionnaire not answered or all variables used in the derivation of the outcome are missing.
** One or more, but not all, variables used in the derivation of the outcome are missing.
*** No variables used in the derivation of the outcome are missing.

For peer review only - http://bmjopen.bmj.com/site/about/guidelines.xhtml

Barts and The London
School of Medicine and Dentistry

PCTU

	Questionnaire never returned no. (%)	Questionnaire answered by telephone			Questionnaire returned		
		Not completed* no. (%)	Partially completed** no. (%)	Fully completed*** no. (%)	Not completed* no. (%)	Partially completed** no. (%)	Fully completed*** no. (%)
**SF36 - General Health**							
Baseline	5 (5.6)	n/a	n/a	n/a	0 (0.0)	1 (1.1)	84 (93.3)
60 days	38 (42.2)	10 (11.1)	0 (0.0)	0 (0.0)	0 (0.0)	1 (1.1)	41 (45.6)
3 months	31 (34.4)	13 (14.4)	0 (0.0)	0 (0.0)	0 (0.0)	1 (1.1)	45 (50.0)
6 months	33 (36.7)	24 (26.7)	0 (0.0)	0 (0.0)	0 (0.0)	0 (0.0)	33 (36.7)
**SF36 - Physical functioning**							
Baseline	5 (5.6)	n/a	n/a	n/a	0 (0.0)	4 (4.4)	81 (90.0)
60 days	38 (42.2)	10 (11.1)	0 (0.0)	0 (0.0)	0 (0.0)	3 (3.3)	39 (43.3)
3 months	31 (34.4)	13 (14.4)	0 (0.0)	0 (0.0)	1 (1.1)	2 (2.2)	43 (47.8)
6 months	33 (36.7)	24 (26.7)	0 (0.0)	0 (0.0)	0 (0.0)	3 (3.3)	30 (33.3)
**SF36 - Pain**							
Baseline	5 (5.6)	n/a	n/a	n/a	0 (0.0)	0 (0.0)	85 (94.4)
60 days	38 (42.2)	10 (11.1)	0 (0.0)	0 (0.0)	0 (0.0)	0 (0.0)	42 (46.7)
3 months	31 (34.4)	13 (14.4)	0 (0.0)	0 (0.0)	1 (1.1)	0 (0.0)	45 (50.0)
6 months	33 (36.7)	24 (26.7)	0 (0.0)	0 (0.0)	0 (0.0)	0 (0.0)	33 (36.7)
**SF36 - Social functioning**							
Baseline	5 (5.6)	n/a	n/a	n/a	0 (0.0)	0 (0.0)	85 (94.4)
60 days	38 (42.2)	10 (11.1)	0 (0.0)	0 (0.0)	0 (0.0)	1 (1.1)	41 (45.6)
3 months	31 (34.4)	13 (14.4)	0 (0.0)	0 (0.0)	1 (1.1)	1 (1.1)	44 (48.9)
6 months	33 (36.7)	24 (26.7)	0 (0.0)	0 (0.0)	0 (0.0)	1 (1.1)	32 (35.6)

* Questionnaire not answered or all variables used in the derivation of the outcome are missing.
** One or more, but not all, variables used in the derivation of the outcome are missing.
*** No variables used in the derivation of the outcome are missing.

For peer review only - http://bmjopen.bmj.com/site/about/guidelines.xhtml

Barts and The London School of Medicine and Dentistry

PCTU

3.3. Results of analysis of clinical outcomes

Table 12. Descriptive statistics for clinical outcomes

	Intervention (N=31)		Active control (N=30)		Usual care (N=29)	
	no. (%)	mean (sd)	no. (%)	mean (sd)	no. (%)	mean (sd)
**CPAQ pain acceptance score**						
Baseline	29 (93.5)	21.9 (9.5)	27 (90.0)	22.7 (8.4)	28 (96.6)	23.8 (8.5)
60 days	15 (48.4)	21.5 (10.2)	16 (53.3)	22.9 (8.5)	19 (65.5)	24.3 (10.2)
3 months	18 (58.1)	20.8 (7.2)	18 (60.0)	22.9 (8.5)	19 (65.5)	25.0 (8.4)
6 months	21 (67.7)	22.7 (10.1)	16 (53.3)	24.0 (11.2)	19 (65.5)	25.8 (7.6)
Included in analysis*	27 (87.1)		23 (76.7)		25 (86.2)	
**HADS depression score**						
Baseline	30 (96.8)	8.7 (5.1)	27 (90.0)	8.6 (5.0)	27 (93.1)	7.4 (3.6)
60 days	14 (45.2)	7.1 (5.2)	16 (53.3)	8.4 (4.0)	19 (65.5)	8.2 (2.9)
3 months	20 (64.5)	8.7 (3.9)	19 (63.3)	8.2 (5.0)	19 (65.5)	6.8 (3.6)
6 months	21 (67.7)	7.0 (4.9)	16 (53.3)	6.1 (4.4)	20 (69.0)	7.0 (4.6)
Included in analysis*	27 (87.1)		23 (76.7)		26 (89.7)	
**HADS anxiety score**						
Baseline	30 (96.8)	12.6 (5.3)	26 (86.7)	12.0 (5.3)	28 (96.6)	10.9 (3.9)
60 days	15 (48.4)	12.5 (5.6)	16 (53.3)	9.5 (4.1)	19 (65.5)	10.7 (4.1)
3 months	20 (64.5)	12.2 (4.1)	19 (63.3)	9.7 (5.6)	19 (65.5)	10.2 (4.0)
6 months	21 (67.7)	10.1 (4.9)	16 (53.3)	8.4 (5.5)	20 (69.0)	9.1 (4.7)
Included in analysis*	27 (87.1)		23 (76.7)		26 (89.7)	
**CAMS-R mindfulness score**						
Baseline	28 (90.3)	28.6 (6.1)	25 (83.3)	28.8 (7.1)	26 (89.7)	30.3 (5.4)
60 days	15 (48.4)	27.4 (5.6)	16 (53.3)	30.6 (8.4)	19 (65.5)	29.7 (7.6)
3 months	19 (61.3)	29.2 (5.2)	19 (63.3)	30.9 (8.8)	18 (62.1)	31.4 (6.4)
6 months	21 (67.7)	29.0 (7.6)	16 (53.3)	31.0 (7.3)	20 (69.0)	32.0 (8.5)
Included in analysis*	27 (87.1)		23 (76.7)		26 (89.7)	

* Included in analysis if outcome is available for at least one follow-up time point.

For peer review only - http://bmjopen.bmj.com/site/about/guidelines.xhtml

Barts and The London School of Medicine and Dentistry
PCTU

	Intervention (N=31)		Active control (N=30)		Usual care (N=29)	
	no. (%)	mean (sd)	no. (%)	mean (sd)	no. (%)	mean (sd)
**CPG disability score**						
Baseline	30 (96.8)	60.6 (24.4)	27 (90.0)	64.6 (19.6)	28 (96.6)	59.2 (24.4)
60 days	15 (48.4)	56.7 (19.8)	16 (53.3)	54.8 (25.0)	19 (65.5)	54.7 (22.9)
3 months	19 (61.3)	61.1 (17.3)	19 (63.3)	52.5 (27.5)	19 (65.5)	52.8 (23.5)
6 months	21 (67.7)	48.3 (28.1)	16 (53.3)	48.5 (24.4)	19 (65.5)	54.2 (23.7)
Included in analysis*	27 (87.1)		23 (76.7)		25 (86.2)	
**PSEQ Self efficacy score**						
Baseline	30 (96.8)	29.1 (14.7)	27 (90.0)	27.9 (14.6)	27 (93.1)	35.5 (10.6)
60 days	14 (45.2)	32.4 (13.9)	9 (30.0)	30.9 (15.9)	16 (55.2)	34.5 (13.1)
3 months	17 (54.8)	28.9 (11.8)	12 (40.0)	30.2 (14.2)	16 (55.2)	39.3 (9.7)
6 months	11 (35.5)	34.3 (12.5)	10 (33.3)	33.7 (17.7)	12 (41.4)	40.2 (13.1)
Included in analysis*	21 (67.7)		18 (60.0)		21 (72.4)	
**SHOW-Q global score**						
Baseline	17 (54.8)	45.4 (20.3)	20 (66.7)	50.9 (20.9)	19 (65.5)	58.1 (22.2)
60 days	4 (12.9)	69.3 (13.3)	8 (26.7)	54.1 (18.0)	13 (44.8)	53.7 (24.5)
3 months	5 (16.1)	51.1 (26.6)	11 (36.7)	44.9 (19.4)	10 (34.5)	61.2 (24.8)
6 months	7 (22.6)	52.3 (15.6)	4 (13.3)	60.9 (14.3)	7 (24.1)	58.5 (26.4)
Included in analysis*	9 (29.0)		14 (46.7)		16 (55.2)	
**SHOW-Q pelvic problem interference score**						
Baseline	23 (74.2)	47.1 (29.0)	24 (80.0)	49.0 (32.7)	26 (89.7)	56.4 (25.9)
60 days	12 (38.7)	60.4 (33.7)	9 (30.0)	60.2 (27.9)	15 (51.7)	51.7 (28.9)
3 months	12 (38.7)	54.9 (34.0)	11 (36.7)	50.0 (25.3)	15 (51.7)	69.4 (32.8)
6 months	9 (29.0)	65.7 (22.2)	9 (30.0)	59.3 (33.4)	11 (37.9)	57.6 (32.8)
Included in analysis*	16 (51.6)		17 (56.7)		20 (69.0)	
**MYMOP subjective outcome score**						
Baseline	30 (96.8)	4.1 (1.2)	27 (90.0)	3.9 (1.3)	27 (93.1)	3.9 (1.1)
60 days	13 (41.9)	3.2 (1.4)	14 (46.7)	3.5 (1.3)	14 (48.3)	3.6 (1.2)
3 months	15 (48.4)	3.4 (1.3)	16 (53.3)	3.1 (1.6)	16 (55.2)	2.9 (1.4)
6 months	18 (58.1)	3.0 (1.4)	15 (50.0)	3.0 (1.5)	18 (62.1)	3.1 (1.5)
Included in analysis*	25 (80.6)		21 (70.0)		24 (82.8)	

* Included in analysis if outcome is available for at least one follow-up time point.

PCTU only

For peer review only - http://bmjopen.bmj.com/site/about/guidelines.xhtml

Barts and The London School of Medicine and Dentistry PCTU

	Intervention (N=31)		Active control (N=30)		Usual care (N=29)	
	no. (%)	mean (sd)	no. (%)	mean (sd)	no. (%)	mean (sd)
**SF36 - General Health**						
Baseline	29 (93.5)	39.1 (20.3)	27 (90.0)	42.0 (19.8)	28 (96.6)	37.9 (21.4)
60 days	15 (48.4)	45.0 (21.2)	9 (30.0)	51.1 (19.2)	17 (58.6)	37.6 (19.9)
3 months	17 (54.8)	44.1 (21.7)	12 (40.0)	42.1 (23.2)	16 (55.2)	40.3 (19.4)
6 months	11 (35.5)	54.5 (19.0)	10 (33.3)	54.5 (24.2)	12 (41.4)	40.0 (27.8)
Included in analysis*	21 (67.7)		18 (60.0)		22 (75.9)	
**SF36 - Physical functioning**						
Baseline	28 (90.3)	56.3 (30.2)	26 (86.7)	55.8 (32.2)	27 (93.1)	66.5 (30.4)
60 days	13 (41.9)	61.2 (27.1)	9 (30.0)	60.6 (25.7)	17 (58.6)	66.5 (30.0)
3 months	15 (48.4)	58.3 (24.0)	12 (40.0)	54.6 (30.7)	16 (55.2)	69.1 (27.5)
6 months	10 (32.3)	66.0 (26.5)	10 (33.3)	72.0 (28.6)	10 (34.5)	63.5 (37.4)
Included in analysis*	20 (64.5)		18 (60.0)		22 (75.9)	
**SF36 - Pain**						
Baseline	30 (96.8)	35.1 (17.5)	27 (90.0)	34.7 (20.6)	28 (96.6)	37.6 (20.6)
60 days	15 (48.4)	39.0 (19.2)	9 (30.0)	43.1 (33.0)	18 (62.1)	40.0 (24.5)
3 months	17 (54.8)	43.7 (17.6)	12 (40.0)	46.7 (22.7)	16 (55.2)	49.5 (25.9)
6 months	11 (35.5)	50.0 (17.8)	10 (33.3)	61.0 (19.9)	12 (41.4)	48.3 (24.8)
Included in analysis*	21 (67.7)		18 (60.0)		22 (75.9)	
**SF36 - Social functioning**						
Baseline	30 (96.8)	37.5 (19.1)	27 (90.0)	38.0 (28.3)	28 (96.6)	50.4 (25.3)
60 days	15 (48.4)	45.8 (27.4)	9 (30.0)	55.6 (29.4)	17 (58.6)	51.5 (28.9)
3 months	17 (54.8)	50.7 (20.9)	12 (40.0)	49.0 (30.4)	15 (51.7)	57.5 (29.0)
6 months	11 (35.5)	54.5 (21.8)	10 (33.3)	56.3 (27.8)	11 (37.9)	59.1 (34.0)
Included in analysis*	21 (67.7)		18 (60.0)		22 (75.9)	

* Included in analysis if outcome is available for at least one follow-up time point.

PCTU only

For peer review only - http://bmjopen.bmj.com/site/about/guidelines.xhtml

Barts and The London School of Medicine and Dentistry

PCTU

Table 13. Estimated treatment effects for clinical outcomes

	Intervention vs Active control adjusted mean difference (95% CI)	Intervention vs Usual care adjusted mean difference (95% CI)	Active control vs Usual care adjusted mean difference (95% CI)
**CPAQ pain acceptance score**			
60 days	-2.3 (-6.6, 2.0)	-4.0 (-8.1, 0.1)	-1.7 (-5.8, 2.4)
3 months	-3.0 (-6.8, 0.7)	-4.5 (-8.2, -0.9)	-1.5 (-5.2, 2.2)
6 months	-1.4 (-5.8, 3.0)	-4.0 (-8.2, 0.2)	-2.5 (-7.0, 2.0)
**HADS depression score**			
60 days	-0.7 (-2.7, 1.2)	-1.2 (-3.1, 0.6)	-0.5 (-2.3, 1.3)
3 months	0.5 (-1.6, 2.6)	1.2 (-0.9, 3.4)	0.8 (-1.4, 2.9)
6 months	0.5 (-1.7, 2.6)	0.4 (-1.7, 2.4)	-0.1 (-2.3, 2.1)
**HADS anxiety score**			
60 days	2.0 (-0.1, 4.1)	1.0 (-1.1, 3.0)	-1.0 (-3.0, 1.0)
3 months	1.9 (-0.3, 4.0)	1.5 (-0.6, 3.6)	-0.4 (-2.5, 1.7)
6 months	0.1 (-2.3, 2.5)	0.3 (-2.0, 2.6)	0.2 (-2.2, 2.6)
**CAMS-R mindfulness score**			
60 days	-3.5 (-7.3, 0.4)	-2.2 (-5.9, 1.4)	1.2 (-2.5, 4.9)
3 months	-2.5 (-5.8, 0.8)	-2.3 (-5.5, 1.0)	0.2 (-3.1, 3.5)
6 months	-1.4 (-4.9, 2.2)	-2.9 (-6.3, 0.4)	-1.6 (-5.1, 2.0)
**CPG disability score**			
60 days	5.1 (-7.2, 17.5)	3.8 (-8.1, 15.7)	-1.4 (-13.1, 10.4)
3 months	8.8 (-3.4, 21.0)	7.6 (-4.5, 19.7)	-1.2 (-13.4, 10.9)
6 months	1.9 (-12.1, 16.0)	1.0 (-12.6, 14.5)	-1.0 (-15.3, 13.4)
**PSEQ Self efficacy score**			
60 days	0.1 (-8.2, 8.4)	-0.2 (-7.4, 6.9)	-0.3 (-8.4, 7.8)
3 months	-3.6 (-9.8, 2.6)	-7.1 (-12.9, -1.2)	-3.5 (-9.8, 2.9)
6 months	-5.9 (-14.8, 3.0)	-8.7 (-17.1, -0.2)	-2.8 (-11.6, 5.9)

For peer review only - http://bmjopen.bmj.com/site/about/guidelines.xhtml

Barts and The London School of Medicine and Dentistry PCTU

	Intervention vs Active control adjusted mean difference (95% CI)	Intervention vs Usual care adjusted mean difference (95% CI)	Active control vs Usual care adjusted mean difference (95% CI)
**SHOW-Q global score**			
60 days	7.0 (-7.2, 21.2)	8.3 (-5.2, 21.8)	1.3 (-9.8, 12.4)
3 months	3.5 (-13.9, 20.9)	-4.8 (-22.0, 12.3)	-8.3 (-23.2, 6.6)
6 months	-11.5 (-27.7, 4.8)	-10.7 (-25.8, 4.3)	0.7 (-14.5, 15.9)
**SHOW-Q pelvic problem interference score**			
60 days	-7.2 (-28.0, 13.5)	3.6 (-14.7, 21.9)	10.9 (-8.9, 30.7)
3 months	-1.2 (-25.1, 22.8)	-10.2 (-32.5, 12.1)	-9.0 (-31.9, 13.8)
6 months	3.3 (-21.3, 27.9)	4.7 (-18.7, 28.1)	1.4 (-22.1, 24.8)
**MYMOP subjective outcome score**			
60 days	0.0 (-0.7, 0.8)	-0.3 (-1.1, 0.4)	-0.4 (-1.1, 0.4)
3 months	0.6 (-0.2, 1.5)	0.6 (-0.2, 1.4)	-0.0 (-0.9, 0.8)
6 months	-0.2 (-1.1, 0.7)	0.2 (-0.7, 1.1)	0.4 (-0.6, 1.3)
**SF36 - General Health**			
60 days	-8.8 (-19.4, 1.8)	-0.9 (-10.0, 8.3)	7.9 (-2.5, 18.3)
3 months	2.0 (-7.3, 11.3)	-5.6 (-14.5, 3.3)	-7.6 (-17.1, 1.9)
6 months	-4.6 (-18.2, 8.9)	-1.9 (-14.9, 11.0)	2.7 (-10.8, 16.2)
**SF36 - Physical functioning**			
60 days	0.1 (-16.0, 16.2)	-6.5 (-20.9, 7.9)	-6.6 (-22.2, 9.0)
3 months	-4.9 (-19.0, 9.3)	-7.7 (-20.8, 5.4)	-2.8 (-16.8, 11.1)
6 months	-2.4 (-24.7, 19.9)	6.3 (-15.7, 28.2)	8.6 (-13.6, 30.9)
**SF36 - Pain**			
60 days	-3.7 (-19.8, 12.3)	0.5 (-12.9, 13.9)	4.2 (-11.4, 19.8)
3 months	-6.4 (-20.7, 7.9)	-7.3 (-20.8, 6.2)	-0.9 (-15.3, 13.6)
6 months	-8.5 (-22.8, 5.8)	0.7 (-13.0, 14.4)	9.2 (-5.0, 23.4)
**SF36 - Social functioning**			
60 days	-17.1 (-33.4, -0.7)	5.2 (-8.8, 19.1)	22.2 (5.7, 38.8)
3 months	-8.2 (-26.5, 10.1)	4.3 (-13.2, 21.8)	12.5 (-6.5, 31.5)
6 months	0.3 (-18.9, 19.6)	3.9 (-15.0, 22.8)	3.5 (-16.0, 23.1)

For peer review only - http://bmjopen.bmj.com/site/about/guidelines.xhtml

Barts and The London
School of Medicine and Dentistry

PCTU

Figure 2. Figure 3: Estimated treatment effects and 95% confidence intervals for CPAQ

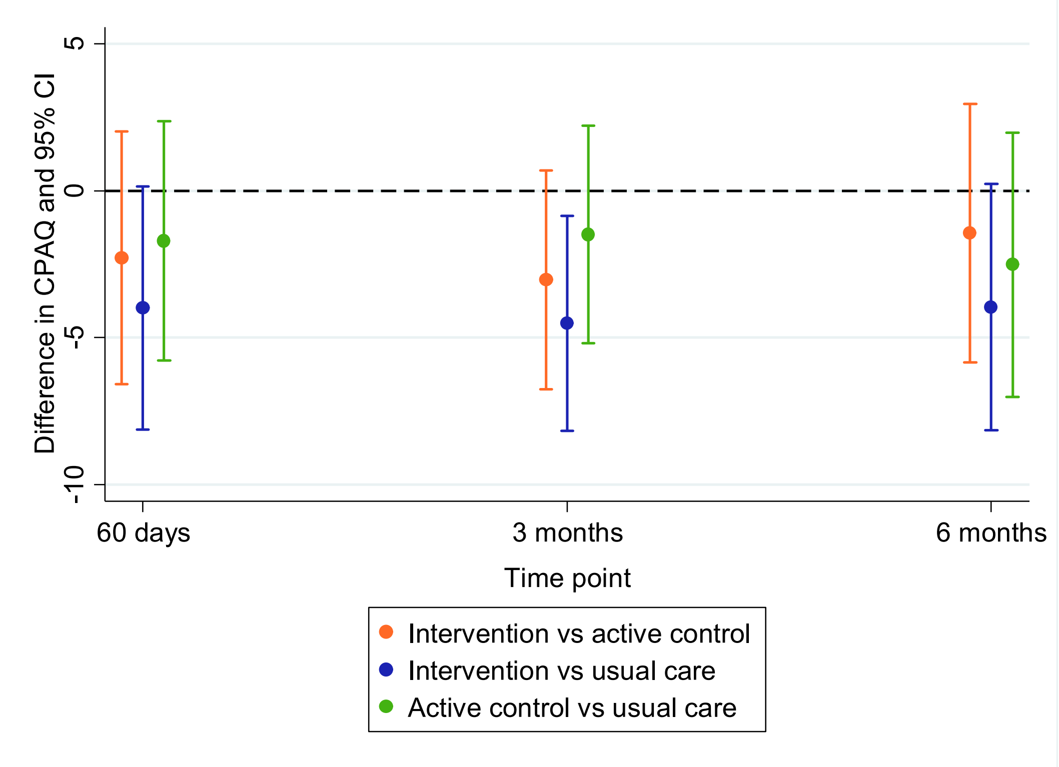

For peer review only - http://bmjopen.bmj.com/site/about/guidelines.xhtml

CONSORT 2010 checklist of information to include when reporting a pilot or feasibility trial*

Section/Topic	Item No	Checklist item	Reported on page No
**Title and abstract**			
	1a	Identification as a pilot or feasibility randomised trial in the title	1
	1b	Structured summary of pilot trial design, methods, results, and conclusions (for specific guidance see CONSORT abstract extension for pilot trials)	2
**Introduction**			
Background and objectives	2a	Scientific background and explanation of rationale for future definitive trial, and reasons for randomised pilot trial	4
	2b	Specific objectives or research questions for pilot trial	4
**Methods**			
Trial design	3a	Description of pilot trial design (such as parallel, factorial) including allocation ratio	5
	3b	Important changes to methods after pilot trial commencement (such as eligibility criteria), with reasons	n/a
Participants	4a	Eligibility criteria for participants	5
	4b	Settings and locations where the data were collected	6
	4c	How participants were identified and consented	5
Interventions	5	The interventions for each group with sufficient details to allow replication, including how and when they were actually administered	5
Outcomes	6a	Completely defined prespecified assessments or measurements to address each pilot trial objective specified in 2b, including how and when they were assessed	6-7
	6b	Any changes to pilot trial assessments or measurements after the pilot trial commenced, with reasons	7
	6c	If applicable, prespecified criteria used to judge whether, or how, to proceed with future definitive trial	n/a
Sample size	7a	Rationale for numbers in the pilot trial	8
	7b	When applicable, explanation of any interim analyses and stopping guidelines	n/a
Randomisation:			
Sequence generation	8a	Method used to generate the random allocation sequence	5
	8b	Type of randomisation(s); details of any restriction (such as blocking and block size)	5
Allocation concealment mechanism	9	Mechanism used to implement the random allocation sequence (such as sequentially numbered containers), describing any steps taken to conceal the sequence until interventions were assigned	5

For peer review only

Implementation	10	Who generated the random allocation sequence, who enrolled participants, and who assigned participants to interventions	5
Blinding	11a	If done, who was blinded after assignment to interventions (for example, participants, care providers, those assessing outcomes) and how	6
	11b	If relevant, description of the similarity of interventions	5
Statistical methods	12	Methods used to address each pilot trial objective whether qualitative or quantitative	8
**Results**			
Participant flow (a diagram is strongly recommended)	13a	For each group, the numbers of participants who were approached and/or assessed for eligibility, randomly assigned, received intended treatment, and were assessed for each objective	13
	13b	For each group, losses and exclusions after randomisation, together with reasons	13
Recruitment	14a	Dates defining the periods of recruitment and follow-up	8
	14b	Why the pilot trial ended or was stopped	8
Baseline data	15	A table showing baseline demographic and clinical characteristics for each group	15
Numbers analysed	16	For each objective, number of participants (denominator) included in each analysis. If relevant, these numbers should be by randomised group	Supplementary tables
Outcomes and estimation	17	For each objective, results including expressions of uncertainty (such as 95% confidence interval) for any estimates. If relevant, these results should be by randomised group	Supplementary tables
Ancillary analyses	18	Results of any other analyses performed that could be used to inform the future definitive trial	n/a
Harms	19	All important harms or unintended effects in each group (for specific guidance see CONSORT for harms)	Supplementary tables
	19a	If relevant, other important unintended consequences	n/a
**Discussion**			
Limitations	20	Pilot trial limitations, addressing sources of potential bias and remaining uncertainty about feasibility	9
Generalisability	21	Generalisability (applicability) of pilot trial methods and findings to future definitive trial and other studies	9
Interpretation	22	Interpretation consistent with pilot trial objectives and findings, balancing potential benefits and harms, and considering other relevant evidence	9
	22a	Implications for progression from pilot to future definitive trial, including any proposed amendments	9
**Other information**			
Registration	23	Registration number for pilot trial and name of trial registry	3
Protocol	24	Where the pilot trial protocol can be accessed, if available	Supplementary material
Funding	25	Sources of funding and other support (such as supply of drugs), role of funders	3

For peer review only

	26	Ethical approval or approval by research review committee, confirmed with reference number	8

Citation: Eldridge SM, Chan CL, Campbell MJ, Bond CM, Hopewell S, Thabane L, et al. CONSORT 2010 statement: extension to randomised pilot and feasibility trials. BMJ. 2016;355.
*We strongly recommend reading this statement in conjunction with the CONSORT 2010, extension to randomised pilot and feasibility trials, Explanation and Elaboration for important clarifications on all the items. If relevant, we also recommend reading CONSORT extensions for cluster randomised trials, non-inferiority and equivalence trials, non-pharmacological treatments, herbal interventions, and pragmatic trials. Additional extensions are forthcoming: for those and for up to date references relevant to this checklist, see www.consort-statement.org.

For peer review only

BMJ Open

A smartphone app using psychological approaches for women with chronic pelvic pain presenting to gynaecology clinics (MEMPHIS): a randomised feasibility trial

Journal:	*BMJ Open*
Manuscript ID	bmjopen-2019-030164.R1
Article Type:	Original research
Date Submitted by the Author:	16-Sep-2019

Complete List of Authors:	Forbes, Gordon; King's College London, IoPPN<br>Newton, Sian; Queen Mary University of London, Centre for Primary Care and Population Health<br>Cantalapiedra Calvete, Clara; Barts Health NHS Trust, United Kingdom, Department of Obstetrics and Gynaecology<br>Birch, Judy; Pelvic Pain Support Network<br>Dodds, Julie; Barts and The London School of Medicine and Dentistry, Women's Health Research Unit<br>Steed, Liz; Queen Mary University of London, Centre for Primary Care and Population Health<br>Rivas, Carol; University of Southamptom, Faculty of Health Sciences<br>Khan, Khalid; Barts and The London School of Medicine and Dentistry, Women's Health Research Unit<br>Röhricht, Frank; East London NHS Foundation Trust<br>Taylor, Stephanie; Queen Mary University of London, Centre for Primary Care and Public Health<br>Kahan, Brennan; Queen Mary University of London, Pragmatic Clinical Trials Unit<br>Ball, Elizabeth; Barts Health NHS Trust, Department of Obstetrics and Gynaecology

**Primary Subject Heading**:	Obstetrics and gynaecology
Secondary Subject Heading:	Obstetrics and gynaecology
Keywords:	chronic pain, meditation, mindfulness, mobile applications, pelvic pain, randomized controlled trial

SCHOLARONE™
Manuscripts

I, the Submitting Author has the right to grant and does grant on behalf of all authors of the Work (as defined in the below author licence), an exclusive licence and/or a non-exclusive licence for contributions from authors who are: i) UK Crown employees; ii) where BMJ has agreed a CC-BY licence shall apply, and/or iii) in accordance with the terms applicable for US Federal Government officers or employees acting as part of their official duties; on a worldwide, perpetual, irrevocable, royalty-free basis to BMJ Publishing Group Ltd ("BMJ") its licensees and where the relevant Journal is co-owned by BMJ to the co-owners of the Journal, to publish the Work in this journal and any other BMJ products and to exploit all rights, as set out in our licence.

The Submitting Author accepts and understands that any supply made under these terms is made by BMJ to the Submitting Author unless you are acting as an employee on behalf of your employer or a postgraduate student of an affiliated institution which is paying any applicable article publishing charge ("APC") for Open Access articles. Where the Submitting Author wishes to make the Work available on an Open Access basis (and intends to pay the relevant APC), the terms of reuse of such Open Access shall be governed by a Creative Commons licence – details of these licences and which Creative Commons licence will apply to this Work are set out in our licence referred to above.

Other than as permitted in any relevant BMJ Author's Self Archiving Policies, I confirm this Work has not been accepted for publication elsewhere, is not being considered for publication elsewhere and does not duplicate material already published. I confirm all authors consent to publication of this Work and authorise the granting of this licence.

For peer review only

A smartphone app using psychological approaches for women with chronic pelvic pain presenting to gynaecology clinics (MEMPHIS): a randomised feasibility trial

Gordon Forbes
King's College London, IoPPN, London, UK

Newton, Sian
Centre for Primary Care and Population Health, Queen Mary University of London, United Kingdom

Cantalapiedra, Clara
Department of Obstetrics and Gynaecology, Barts Health NHS Trust, United Kingdom

Birch, Judy
Pelvic Pain Support Network, UK

Dodds, Julie
Women's Health Research Unit, Barts and the London School of Medicine and Dentistry, Queen Mary University of London, United Kingdom

Steed, Elizabeth
Centre for Primary Care and Population Health, Queen Mary University of London, United Kingdom

Rivas, Carol
Social Science Research Unit, University College London, United Kingdom

Khan, Khalid S.
Women's Health Research Unit, Barts and the London School of Medicine and Dentistry, Queen Mary University of London, United Kingdom

Rohricht, Frank
East London NHS Foundation Trust, United Kingdom

Taylor, Stephanie JC
Centre for Primary Care and Population Health, Queen Mary University of London, United Kingdom

Brennan C, Kahan
Pragmatic Clinical Trials Unit, Centre for Primary Care and Population Health, Queen Mary University of London, United Kingdom

Ball, Elizabeth
Department of Obstetrics and Gynaecology, Barts Health NHS Trust, United Kingdom

Corresponding Author: elizabeth.ball9@nhs.net

Objectives

To evaluate the feasibility of a randomised trial of a modified, pre-existing, mindfulness meditation smartphone app for women with chronic pelvic pain.

Design

Three arm randomised feasibility trial.

Setting

Women were recruited at two gynaecology clinics in the UK. Interventions were delivered via smartphone or computer at a location of participants choosing.

Participants

Women were eligible for the study if they were over 18, had been experiencing organic or non-organic chronic pelvic pain for six months or more, and had access to a computer or smartphone. 90 women were randomised.

Interventions

Daily mindfulness meditation delivered by smartphone app, an active control app which delivered muscle relaxation techniques, and usual care without app. Interventions were delivered over 60-days.

Primary and secondary outcome measures

Outcomes included length of recruitment, follow up rates, adherence to the app interventions, and clinical outcomes measured at baseline, two, three and six months.

Results

The target sample size was recruited in 145 days. Adherence to the app interventions was extremely low (mean app use 1.8 days mindfulness meditation group, 7.0 days active control). Fifty-seven (63%) women completed 6-month follow-up, and 75 (83%) women completed at least one post-randomisation follow-up. The 95% confidence intervals for clinical outcomes were consistent with no benefit from the mindfulness meditation app; for example, mean differences in pain acceptance scores at 60 days (higher scores are better) were -2.3 (mindfulness meditation vs. usual care, 95% CI: -6.6, 2.0) and -4.0 (mindfulness meditation vs. active control, 95% CI: -8.1, 0.1).

Conclusions

Despite high recruitment and adequate follow-up rates, demonstrating feasibility, the extremely low adherence suggests a definitive randomised trial of the mindfulness meditation app used in this study is not warranted. Future research should focus on improving patient engagement.

ClinicalTrials.gov registration: NCT02721108, ISRCTN 10925965

Funding: This research was supported by the UK National Institute of Health Research, Research for Patient Benefit programme (RfPB PB-PG-1013-32025).

Strengths and limitations of this study

- This is a randomised feasibility study designed specifically to test whether evaluation of the intervention is viable in a full scale randomised trial
- The trial achieved target recruitment demonstrating feasibility of recruiting patients to trials of apps for women experiencing chronic pelvic pain.
- Measures of adherence to the app interventions were robust and complete as they relied on system generated data
- This trial evaluated only one app provided by a leading developer of mindfulness meditation apps

BACKGROUND

Chronic pelvic pain in women is defined as intermittent or constant pain in the lower abdomen or pelvis for six or more months, and affects more than 24% of women worldwide (1). It has considerable impact on patients' quality of life, including their mental health and their income due to loss of working days and diminished work capacity (2). Chronic pelvic pain may or may not have an identifiable pathology and has both physical and psychological contributors (3). Chronic pelvic pain is difficult to treat but health outcomes can be improved by psychological and lifestyle interventions (4, 5). However these are often not provided (6, 7) due to difficult access or service shortages.

Mindfulness is a form of meditation where the client attempts to maintain attention on their own breathing. Whenever attention wanders from the breath to thoughts and feelings, the client will simply take notice of them and let them go as attention is returned to the breath. There is an emphasis on simply taking notice of whatever the mind happens to wander to and accepting each object without making judgements about it or elaborating on its implications additional meaning or need for action. The client is further encouraged to use the same general approach outside of their formal meditation practice, bringing awareness back to the here and now, whenever they notices a general lack of awareness or that attention has become focused on streams of thoughts and worries (8)

Systematic reviews of randomised controlled trials evaluating mindfulness meditation have shown benefit in chronic pain conditions (positive effects on depression, quality of life and pain symptoms (9, 10). So far no randomised controlled trials of mindfulness meditation exist in chronic pelvic pain in women, but results from uncontrolled studies comparing pre- and post-treatment outcomes have suggested there may be a benefit (such as increased ability to control pain, improvements in mental health, emotional well-being, work and family life and social functioning) (11, 12).

Mindfulness meditation can be resource-intensive and typically requires multiple face-to-face visits over a period of weeks or months. If effective, delivery of mindfulness meditation via smartphone app to women with chronic pelvic pain could provide a new treatment option for this patient group, requiring a minimal increase in resources for healthcare systems. No studies have evaluated mindfulness mediation via smartphone app for women with chronic pelvic pain. We therefore conducted a randomised feasibility trial to assess the feasibility of a future full scale, multi-centre randomised trial to test

effectiveness of a mindfulness meditation intervention delivered by the Headspace smartphone app (Headspace Ltd) for patients with chronic pelvic pain.

The primary objective of the study was to assess the feasibility of implementing a randomised trial to assess the effectiveness of a mindfulness meditation intervention delivered by a smartphone app for women with chronic pelvic pain. Specifically, we assessed feasibility of recruitment, levels of adherence to the intervention, and estimated parameters required for the sample size calculation for a full trial. Secondary objectives were to measure the clinical outcomes that may be used in a future full scale trial. No primary outcome was specified because this was a feasibility study, however it was anticipated that chronic pain acceptance would be the primary outcome for any future study assessing effectiveness. Pain acceptance was chosen by the study group with input from pain patients and clinicians because has been shown to be a meaningful clinical outcome that was improved by mindfulness mediation in other pain conditions (9). This article reports quantitative findings; qualitative findings will be published separately (13).

METHODS

Study design and participants

This three arm parallel group randomised feasibility trial was conducted at two gynecology clinics within Barts Health NHS trust. Eligible patients were aged 18 years or over, had been experiencing chronic pelvic pain with or without identifiable pathology (i.e. organic or non-organic chronic pelvic pain) for six months or more, and understood simple English. Patients were excluded from the trial if they had no access to a personal computer or smartphone, or were current users of the publicly available Headspace app. Patients were recruited via pelvic pain or endometriosis clinics at participating sites as well as at other routine appointments. Prior to randomisation, all participants were provided with a patient information sheet and provided written informed consent. The study protocol has been published (14) and the final version is given in Appendix 1.

Interventions

Full details of the interventions are available in the published protocol (14). Patients were randomised to receive mindfulness meditation, an active control, or usual care only. All participants received usual care, which included watch and wait, medication and/or surgery.

Women in the mindfulness meditation group received access to a 60-day progressive mindfulness meditation course delivered via the Headspace app. The intervention consisted of daily, audio guided, mindfulness meditation sessions. The first 10 days of the course taught basics of mindfulness meditation. Following this, participants were able to access the module on meditation which was targeted at chronic pain. This module was specifically designed for the MEMPHIS trial. Session length was 10 minutes for the first 10 days, 15 minutes up to day 20 and 20 minutes up to day 60.

The active control group received access to a series of muscle relaxation sessions. These sessions were identical every day, except that their duration increased to mirror the increasing duration of the meditation content being listened to by the intervention group.

Women in the mindfulness mediation group and active control group were given instructions on how to install the app. No further face-to-face induction was given on how to carry out the techniques taught in the apps. To maintain blinding between the mindfulness meditation group and active control, both groups accessed their intervention via the same app, and received instructions for the same duration, delivered by the same narrator. Only the content of the instructions differed.

We chose to evaluate an existing commercial app teaching mindfulness by guided meditation (Headspace Ltd) as this approach was expected to save time and money compared to designing a new app from scratch. The Headspace app was adapted for use by chronic pelvic pain patients by augmenting the existing app with a novel module on chronic pain, which could be accessed after completing ten days of basic training in mindfulness meditation.

Randomisation and blinding

Women were randomly allocated 1:1:1 to the active intervention app, active control app, or treatment as usual using random permuted blocks (block size 27, 30, 33) without stratification using a centralised web based service with allocation concealment. The randomisation list was generated using the Pragmatic Clinical Trials Unit's randomisation system using a random number generator. Following randomisation, participants, recruiting staff, and researchers conducting follow-up interviews were not blinded to whether allocation was to the treatment as usual group or to one of the app groups (mindfulness meditation or active control); however, for allocation to an app group they were blinded to which specific app group this was (mindfulness meditation or active control). The trial statisticians

remained blinded to allocation until the statistical analysis plan had been signed off, all data collection was completed, and the dataset was finalised.

Data collection

Data on patient adherence to the app was collected by Headspace Ltd. Data collection was performed automatically by the app and recorded every time a participant completed more than 90% of a session with the app. No data was collected on sessions that were less than 90% complete. Headspace provided the trial team with a list of codes, which were linked to the randomisation system, and given to trial participants to access the app. At the end of the trial, data on completed sessions were transferred via a secure file transfer protocol (SFTP) from Headspace to the trial team. No data which could identify participants were included in this transfer. Clinical outcome measures were collected in person at baseline prior to randomisation and via postal questionnaires or telephone at 2, 3 and 6 months post-randomisation. App satisfaction and usability questionnaires were collected via postal questionnaires or telephone. Shopping vouchers (£5), text reminders and phone calls were introduced to improve follow up rates three months after recruitment began: shopping vouchers were sent in the post with each follow up questionnaire; participants were sent text reminders and up to three attempts were made to contact participants by phone if questionnaire responses were not received within 10 days.

Outcomes

Feasibility outcomes were: time to recruit 90 patients to the study; standard deviation of chronic pain acceptance questionnaire (CPAQ-8) (15) (as this was likely to be the primary outcome for a future full-scale trial); proportion of participants completing a follow-up questionnaire at 6 months post randomisation; and proportion of participants not returning a follow up questionnaire by post but who answered a telephone questionnaire at 6 months. Standard deviation of CPAQ was included as an outcome as this information would be required for the sample size calculation for a full trial. App usability was measured using the system usability scale (16) and a purpose made, non-validated questionnaire developed from PPI group discussion. Adherence to the app interventions was measured in the following ways:

(a) number of days a patient has used the app within 60 days of randomisation;

(b) Number of weeks a patient has used the app on three or more days within the first eight weeks from randomisation;

(c) whether the patient has used the app on at least 22 days within 60 days of randomisation (binary outcome);

(d) whether the patient has used the app on three or more days in 6 or more weeks within the first eight weeks of randomisation (binary outcome);

(e) whether the patient has used the app on 22 or more days within the first 60 days from randomisation and used the app on three or more days in 6 or more weeks within the first eight weeks from randomisation (binary outcome).

Measures of app use were chosen following discussion within the trial management group and trial steering group to give a complete picture of how participants were using the app. App use was defined as having completed at least 90% of a session. This definition of app use was changed after the trial started recruiting but before any data were analysed due to a change in the way data on app use were collected by Headspace. The original definition of app use was for patients to have completed at least 50% of a session.

The following clinical outcomes were measured at baseline, 60 days, 3 months and 6 months post randomisation:

a) Pain acceptance score (measured by the chronic pain acceptance questionnaire [CPAQ-8]) (15);

b) pain related disability (chronic pain grade [CPG] – disability subscale) (17);

c) quality of life subscales (measured by the RAND short form 36 health survey [SF-36]): social functioning subscale, pain functioning subscale, and general health subscale (18);

d) the depression and anxiety subscales of the Hospital Anxiety and Depression Scale [HADS] (19)

e) mindfulness (cognitive and mindfulness - revised scale [CAMS-R]) (20);

f) self-efficacy (pain self-efficacy questionnaire [PSEQ]) (21);

g) sexual health amongst sexually active participants (sexual health outcomes in women questionnaire [SHOW-Q]) (22);

h) sexual health pelvic problem interference score (SHOW-Q pelvic problem subscale) (22);

i) an individualised outcome (Measure yourself medical outcome profile [MYMOP]) (23).

Statistical analysis

A sample size of 90 participants was chosen as it would provide a precise estimate for the standard deviation of the primary clinical outcome (likely to be pain acceptance) (24, 25), which could be used to inform the sample size calculation of a subsequent full-scale trial. This sample size is also adequate to provide estimates of proportions for binary outcomes (25).

Feasibility outcomes and baseline data were summarised using descriptive statistics. Clinical outcomes were analysed using a linear mixed-effects models with outcome measurement (at three follow-up time points) as the dependent variable and an unstructured correlation matrix for the residuals (26). The model included fixed effects for time, treatment arm, time-by-treatment interactions and baseline measure of the outcome (27). Analysis was by intention-to-treat; all patients with an observed outcome for at least one of the three follow-up time points were included in the analysis (28), and were analysed according to their randomised group. Missing baseline clinical measures were handled using mean imputation (29). See appendix 2 for a full statistical analysis plan.

Patient and Public Involvement (PPI).

The study design and intervention was discussed with a PPI group formed of 15 women who attended the recruiting clinics. A basic version of the app by Headspace Ltd. was made available to the group for testing. A patient, who bought their own experience and acted as a representative for a charity supporting those with CPP, sat on the trial management group which oversaw the conduct of the trial.

Ethical Approval

Ethics approval was granted by Camden and Kings Cross Research Ethics Committee on 1st February 2016.

RESULTS

Feasibility Outcomes

Ninety women were recruited to the trial in 145 days between May 2016 and September 2016. A CONSORT diagram is shown in figure 1 and baseline characteristics are shown in table 1, with additional baseline data given in appendix 3, tables 1 and 2. Follow up at 6 months was 68% in the mindfulness meditation group, 53% in the active control group and 69% in the usual care group. Follow up rates by method of follow up (phone or questionnaire), at different time points, and a comparison of baseline

characteristics by questionnaire completion are given in appendix 3, tables 3-5 and appendix 3 figure 1. The standard deviation for CPAQ can be found in appendix 3, table 6. Unintentional unblinding of treatment for either participants or researchers collecting data was rare (Appendix 3, table 7).

App use was low in both groups, but was higher in the active control group than the intervention group (app used on mean 1.8 days intervention vs 7.0 active control – table 2). Few women used the app on more than 22 days within 60 days of randomisation (0 intervention vs 2 active control). Adherence to the app intervention was low or entirely absent across all other measures of app use (table 2). Daily app use within 60 days of randomisation is summarised in figure 2. The results from the app usability questionnaire are shown in appendix 3, tables 8 and 9.

Clinical outcomes

We included 27 (87%) women from the intervention group, 23 (77%) from the active control group and 25 (86%) from the usual care group in the analysis of pain acceptance score. The 95% confidence intervals for CPAQ (figure 3) rules out any strong benefit of the intervention compared to either the active control group, or usual care group at any time point (higher CPAQ corresponds to better outcomes). The results for other clinical outcomes are consistent with no effect of the intervention (full results of clinical outcomes are shown in appendix 3 tables 10-13 & figure 2).

DISCUSSION

This trial shows that it is feasible to recruit women to a trial of a mindfulness meditation app. Follow up rates were adequate and including data across all time points meant that a relatively a high proportion of participants could be included in the analysis. This study provides estimates to inform sample size calculations for future research.

Most participants either did not complete any sessions on the apps or used them extremely infrequently. The analyses of clinical outcomes are consistent with no differences in health outcome between the three study arms. For pain acceptance, which was considered to be a likely outcome for a future effectiveness trial, our results suggest a meaningful effect of the mindfulness meditation app, delivered as it is in this trial, is unlikely. An effective intervention requires both engagement from those receiving it and the ability to change the targeted clinical outcome (30). As engagement with the mindfulness meditation app evaluated in this study was very low it is unlikely it would be an effective intervention in

the routine clinical setting for women with chronic pelvic pain, unless delivered as part of an intervention which significantly enhanced rates of engagement.

In addition to the work described in this paper we carried out in-depth qualitative interviews in order to examine the reasons for low levels of user engagement. Suggestions are given for improving the intervention such as co- development, an approach to intervention that involves the users in the design of the intervention The findings are published in the companion paper describing the qualitative arm of this study (13).

An important lesson from this trial for future researchers was that intermediate follow up points allowed for more participants to be included in the analysis of clinical outcomes than were followed up at the final time point. This demonstrates that utilising intermediate follow up time points may help to minimise potential bias from missing data in trials.

Strengths of this study include randomisation of participants, which eliminates bias inherent in other designs such as before-after studies. We also blinded patients, recruiters, and data collectors to which app group patients were allocated to. We used system generated app data and therefore were able to obtain complete adherence data for all participants. One drawback to this method of data collection was that sessions of the app were only recorded as being complete if a participant listened to 90% of the session. This means this study may have underestimated app use if participants were only partially completing sessions. Levels of app use were so low however that this is unlikely to have had a material impact on the study's results. A second limitation is that recruitment was limited to two hospitals in one area of London, this may limit the generalisability of the results to settings where there is higher engagement with smartphone apps.

In conclusion, this study had high recruitment and adequate follow-up rates, demonstrating that it is feasible to conduct randomised trials in this patient population. However, due to extremely low adherence, further randomised trials to evaluate the benefit of the Headspace mindfulness meditation app for women with chronic pelvic pain are not warranted, unless additional steps to improve engagement with the app are included in the intervention. Further discussion of reasons for low engagement and what could be done to improve engagement may be found in the qualitative part of this study (13).

Data availability statements

Anonymised participant data is available upon reasonable request. Please contact pctu-data-sharing@qmul.ac.uk with any data sharing requests.

Competing interests

None declared

Author contributions

EB conceived of the research and lead the study. GF conducted the statistical analysis under supervision from BCK. GF drafted the manuscript. GF, SN, CC, JB, JD, ES, CR, KSK, FR, SJCT, BCK, and EB contributed to the design and conduct of the study, and discussed and reviewed the final manuscript.

References

1. Latthe P, Latthe M, Say L, Gulmezoglu M, Khan KS. WHO systematic review of prevalence of chronic pelvic pain: a neglected reproductive health morbidity. BMC Public Health. 2006;6:177.
2. Zondervan KT, Yudkin PL, Vessey MP, Jenkinson CP, Dawes MG, Barlow DH, et al. Chronic pelvic pain in the community - Symptoms, investigations, and diagnoses. Am J Obstet Gynecol. 2001;184(6):1149-55.
3. Daniels JP, Khan KS. Chronic pelvic pain in women. BMJ. 2010;341:c4834.
4. Moore SJ KS. Green Top Guideline No 41: the initial management of Chronic Pelvic Pain. In: Gynaecologists TrcoOa, editor. 2012.
5. Peters AA, van Dorst E, Jellis B, van Zuuren E, Hermans J, Trimbos JB. A randomized clinical trial to compare two different approaches in women with chronic pelvic pain. Obstet Gynecol. 1991;77(5):740-4.
6. Romao APMS, Gorayeb R, Romao GS, Poli-Neto OB, dos Reis FJC, Rosa-e-Silva JC, et al. High levels of anxiety and depression have a negative effect on quality of life of women with chronic pelvic pain. Int J Clin Pract. 2009;63(5):707-11.
7. Stones RW, Price C. Health services for women with chronic pelvic pain. J R Soc Med. 2002;95(11):531-5.
8. Bishop SR, Lau M, Shapiro S, Carlson L, Anderson ND, Carmody J, et al. Mindfulness: A proposed operational definition. Clinical Psychology-Science and Practice. 2004;11(3):230-41.
9. Ball EF, Sharizan ENSM, Franklin G, Rogozinska E. Does mindfulness meditation improve chronic pain? A systematic review. Curr Opin Obstet Gynecol. 2017;29(6):359-66.
10. Hilton L, Hempel S, Ewing BA, Apaydin E, Xenakis L, Newberry S, et al. Mindfulness Meditation for Chronic Pain: Systematic Review and Meta-analysis. Ann Behav Med. 2017;51(2):199-213.
11. Kold M, Hansen T, Vedsted-Hansen H, Forman A. Mindfulness-based psychological intervention for coping with pain in endometriosis. Nordic Psychology. 2012;64(1):2-16.
12. Fox SD, Flynn E, Allen RH. Mindfulness Meditation for Women with Chronic Pelvic Pain A Pilot Study. J Reprod Med. 2011;56(3-4):158-62.
13. Ball E, Newton S, Kahan BC, Forbes G, Rohricht F, Steed E, et al. Using a Mindfulness App for women with chronic pelvic pain: Qualitative data of user experience and lessons learnt Submitted. 2018.
14. Ball E, Newton S, Kahan BC, Forbes G, Wright N, Calvete CC, et al. Smartphone App Using Mindfulness Meditation for Women With Chronic Pelvic Pain (MEMPHIS): Protocol for a Randomized Feasibility Trial. Jmir Research Protocols. 2018;7(1).
15. Fish RA, McGuire B, Hogan M, Morrison TG, Stewart I. Validation of the Chronic Pain Acceptance Questionnaire (CPAQ) in an Internet sample and development and preliminary validation of the CPAQ-8. Pain. 2010;149(3):435-43.
16. Brooke J. SUS-A quick and dirty usability scale. Usability evaluation in industry. 1996;189(194):4-7.
17. Von Korff M, Ormel J, Keefe FJ, Dworkin SF. Grading the severity of chronic pain. Pain. 1992;50(2):133-49.
18. Ware JE, Sherbourne CD. The Mos 36-Item Short-Form Health Survey (Sf-36) .1. Conceptual-Framework and Item Selection. Med Care. 1992;30(6):473-83.
19. Zigmond AS, Snaith RP. The Hospital Anxiety and Depression Scale. Acta Psychiatr Scand. 1983;67(6):361-70.
20. Feldman G, Hayes A, Kumar S, Greeson J, Laurenceau JP. Mindfulness and emotion regulation: The development and initial validation of the Cognitive and Affective Mindfulness Scale-Revised (CAMS-R). Journal of Psychopathology and Behavioral Assessment. 2007;29(3):177-90.

21. Nicholas MK. The pain self-efficacy questionnaire: Taking pain into account. European Journal of Pain. 2007;11(2):153-63.
22. Learman LA, Huang AJ, Nakagawa S, Gregorich SE, Kuppermann M. Development and validation of a sexual functioning measure for use in diverse women's health outcome studies. Am J Obstet Gynecol. 2008;198(6).
23. Paterson C. Measuring outcomes in primary care: A patient generated measure, MYMOP, compared with the SF-36 health survey. Br Med J. 1996;312(7037):1016-20.
24. Lancaster GA, Dodd S, Williamson PR. Design and analysis of pilot studies: recommendations for good practice. J Eval Clin Pract. 2004;10(2):307-12.
25. Teare MD, Dimairo M, Shephard N, Hayman A, Whitehead A, Walters SJ. Sample size requirements to estimate key design parameters from external pilot randomised controlled trials: a simulation study. Trials. 2014;15.
26. Rabe-Hesketh S, Skrondal A. Multilevel and longitudinal modeling using Stata. 3rd ed. College Station, Tex.: Stata Press Publication; 2012.
27. Kahan BC, Jairath V, Dore CJ, Morris TP. The risks and rewards of covariate adjustment in randomized trials: an assessment of 12 outcomes from 8 studies. Trials. 2014;15.
28. White IR, Horton NJ, Carpenter J, Pocock SJ. Strategy for intention to treat analysis in randomised trials with missing outcome data. Bmj-British Medical Journal. 2011;342.
29. White IR, Thompson SG. Adjusting for partially missing baseline measurements in randomized trials. Stat Med. 2005;24(7):993-1007.
30. Barrera M, Castro FG. A heuristic framework for the cultural adaptation of interventions. Clinical Psychology-Science and Practice. 2006;13(4):311-6.

Figures

Figure 1: CONSORT diagram

Figure 2: Daily app use (defined as completing ≥90% of a session) within 60 days of randomisation in the intervention and active control groups.

Figure 3: Mean (95% CI) chronic pain acceptance score (CPAQ) and estimated treatment effect (95% CI) at each follow-up time point. (CPAQ). Higher scores indicate better health outcomes.

Tables

Table 1: Baseline demographics and medical history. Figures are mean (SD) unless stated otherwise.

	Summary measure		
	**Intervention (N=31)**	**Active control (N=30)**	**Usual care (N=29)**
**Demographics**			
Age (Years)	34.8 (9.9)	35.7 (5.7)	35.0 (8.6)
Body mass index (kg/m^2)	28.7 (7.0)	26.2 (5.5)	26.6 (6.3)
Living arrangements - no. (%)			
Alone	1 (3.3)	2 (7.4)	3 (11.1)
With others	29 (96.7)	25 (92.6)	24 (88.9)
Employment status - no. (%)			
Employed	19 (63.3)	18 (66.7)	19 (67.9)
Unemployed and looking for work	2 (6.7)	0 (0.0)	1 (3.6)
At school or in full time education	2 (6.7)	1 (3.7)	4 (14.3)
Unable to work due to long term sickness	4 (13.3)	5 (18.5)	1 (3.6)
Looking after your home/family	3 (10.0)	3 (11.1)	2 (7.1)
Retired from paid work	0 (0.0)	0 (0.0)	1 (3.6)
Age left full time education - no. (%)			
Age 12 or less	1 (3.3)	1 (3.8)	1 (3.6)
Age 13 to 16	9 (30.0)	4 (15.4)	3 (10.7)
Age 17 to 19	6 (20.0)	5 (19.2)	3 (10.7)
Age 20 or over	11 (36.7)	15 (57.7)	16 (57.1)
Still in education	3 (10.0)	1 (3.8)	5 (17.9)
Ethnic group - no. (%)			
White	10 (35.7)	10 (43.5)	15 (53.6)
Black	6 (21.4)	4 (17.4)	3 (10.7)
Cetral Asian	1 (3.6)	1 (4.3)	0 (0.0)
Middle Eastern	0 (0.0)	0 (0.0)	1 (3.6)
Southern Asian	8 (28.6)	7 (30.4)	3 (10.7)
Mixed	0 (0.0)	0 (0.0)	2 (7.1)
Other ethnic group	2 (7.1)	1 (4.3)	3 (10.7)
Do not wish to say	1 (3.6)	0 (0.0)	1 (3.6)
Smoker - no. (%)			
Yes	8 (27.6)	3 (12.5)	6 (21.4)
No	21 (72.4)	21 (87.5)	22 (78.6)
If yes, number of cigarettes per week	23.9 (20.3)	40.0 (20.0)	47.6 (35.6)
Drink alcohol - no. (%)			
Yes	10 (34.5)	9 (36.0)	15 (55.6)
No	19 (65.5)	16 (64.0)	12 (44.4)

If yes, number of units per week	5.7 (5.3)	8.3 (4.7)	7.7 (7.2)
**Baseline medical history**			
Duration of pain - no. (%)			
0 to 6 months	2 (6.7)	0 (0.0)	0 (0.0)
7 to 12 months	2 (6.7)	4 (14.8)	2 (7.1)
1 to 2 years	3 (10.0)	5 (18.5)	5 (17.9)
3 to 5 years	13 (43.3)	7 (25.9)	6 (21.4)
6 to 10 years	4 (13.3)	4 (14.8)	3 (10.7)
More than 10 years	6 (20.0)	7 (25.9)	12 (42.9)
Pain over the past week (scale of 0 to 10)	6.9 (2.3)	5.8 (2.8)	6.8 (2.3)

Table 2: App use

Figures are mean (SD) unless stated otherwise.

	**Intervention (N=31)**	**Active control (N=28)***
Number of days a patient has used the app (within 60 days of randomisation)	1.8 (4.3)	7.0 (10.5)
Number of weeks a patient has used the app on three or more days (within the first eight weeks from randomisation)	0.3 (0.8)	1.0 (1.6)
Used the app on 22 or more days within the first 60 days from randomisation - no. (%)	0 (0.0)	2 (7.1)
Used the app on three or more days in 6 or more weeks (within the first eight weeks from randomisation) - no. (%)	0 (0.0)	0 (0.0)
Used the app on 22 or more days within the first 60 days AND used the app on three or more days in 6 or more weeks within the first eight weeks from randomisation - no. (%)	0 (0.0)	0 (0.0)

*2 participants in the active control group withdrew permission for their data to be used and are excluded from this analysis.

Appendix 1: Final study protocol

Appendix 2: Statistical analysis plan

Appendix 3: Supplementary tables

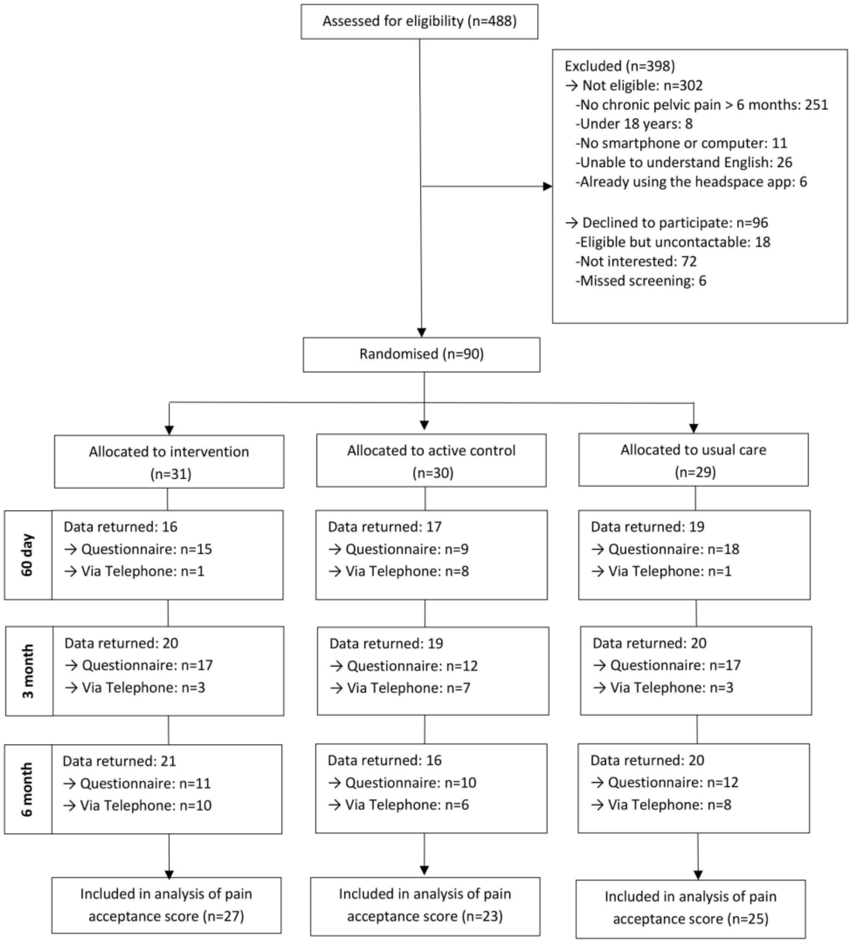

Figure 1: CONSORT diagram

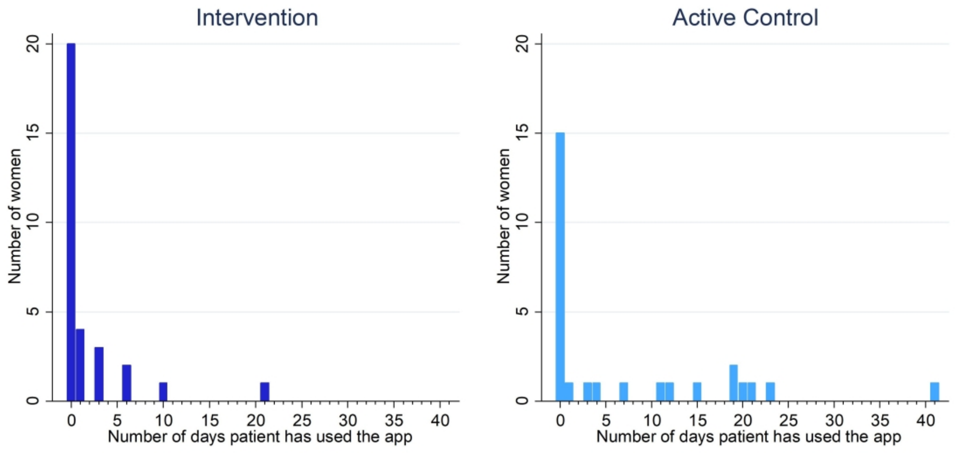

Figure 2: Daily app use (defined as completing >90% of a session) within 60 days of randomisation in the intervention and active control groups.

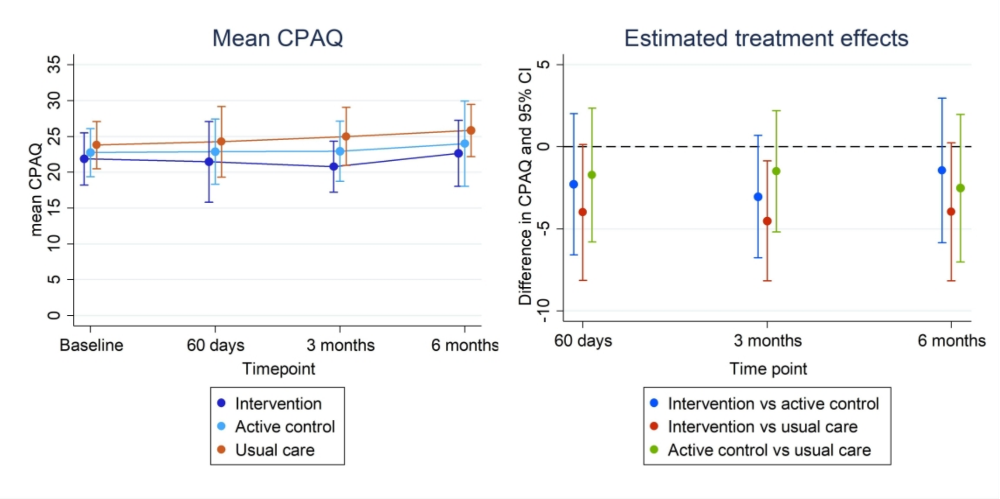

Figure 3: Mean (95% CI) chronic pain acceptance score (CPAQ) and estimated treatment effect (95% CI) at each follow-up time point. (CPAQ). Higher scores indicate better health outcomes.

A smartphone app using psychological approaches for women with chronic pelvic pain (MEMPHIS): a randomised feasibility trial

**Short Title/Acronym**	MEMPHIS
**Sponsor**	Barts Health NHS Trust<br><br>Contact person of the above sponsor organisations is:<br><br>Dr Sally Burtles<br>Director of Research Services & Business Development<br>Joint Research Management Office<br>5 Walden Street<br>London<br>E1 2EF<br>Phone: 020 7882 7260<br>Email: sponsorsrep@bartshealth.nhs.uk
**REC Reference**	15/LO/1967
**Chief Investigator**	**Miss Elizabeth Ball**<br>Women and Children Department<br>Royal London Hospital<br>Barts Health NHS Trust<br>elizabeth.ball@bartshealth.nhs.uk<br>eball69@gmail.com

For peer review only - http://bmjopen.bmj.com/site/about/guidelines.xhtml

Pragmatic Clinical Trials Unit

Blizard Institute, Centre for Primary Care and Public Health

Bart's and The London School Medicine and Dentistry

Queen Mary University of London

Yvonne Carter Building, 58 Turner St

Whitechapel, E1 2AB

PCTU Senior Trial statistician:

Mr Brennan Kahan

+44 207 882 2483

b.kahan@qmul.ac.uk

PCTU Junior Trial Statistician

Mr Neil Wright

+44 207 882 6564

n.wright@qmul.ac.uk

PCTU Data Manager:

Mr Mike Waring

+44 207 882 6914

m.r.waring@qmul.ac.uk

PCTU QA Manager:

Ms Anitha Manivannan

+44 207 882 6312

a.manivannan@qmul.ac.uk

Protocol development

Miss Elizabeth Ball

Chief Investigator

For peer review only - http://bmjopen.bmj.com/site/about/guidelines.xhtml

Barts and The London
School of Medicine and Dentistry

elizabeth.ball@bartshealth.nhs.uk

eball69@gmail.com

Dr Julie Dodds

Senior Research Manager

j.dodds@qmul.ac.uk

Mr Brennan Kahan

PCTU Senior Trial Statistician

b.kahan@qmul.ac.uk

Mr Neil Wright

PCTU Junior Trial Statistician

n.wright@qmul.ac.uk

Dr Carol Rivas

Senior Research Fellow

C.A.Rivas@soton.ac.uk

Miss Ewelina Rogozinska

Co-investigator

e.a.rogozinska@qmul.ac.uk

PCTU QA Manager:

Ms Anitha Manivannan

+44 207 882 6312

a.manivannan@qmul.ac.uk

For peer review only - http://bmjopen.bmj.com/site/about/guidelines.xhtml

CONTENTS PAGE

1.GLOSSARY OF TERMS AND ABBREVIATIONS 7
2.SIGNATURE PAGES 8
3.SUMMARY/SYNOPSIS 9
4.INTRODUCTION 11
4.1. Background 11
4.2. Effect of mindfulness meditation in chronic pain patients 12
4.3. On-going studies 12
4.4. Implications for the further development of clinical or public health practice 13
4.5. Potential impact on local policy making and improvement in service delivery 14
5.TRIAL OBJECTIVES 14
5.1. Aims and Objectives 14
5.2. Feasibility outcomes 15
5.3. App satisfaction questionnaires 16
5.4. Clinical Outcomes 17
6.METHODOLOGY 18
6.1. Inclusion Criteria 18
6.2. Exclusion Criteria 18
6.3. Study Design 19
6.4. Study Scheme Diagram 20
7. STUDY PROCEDURES 20
7.1.Informed Consent Procedures 20
7.2. Screening and enrolment 21
7.3. Randomisation Procedures 22
7.4 Blinding 22
7.5. Planned interventions 23
7.6. Concomitant Medications 24
7.7. Reasons for non progression to full trial 24

For peer review only - http://bmjopen.bmj.com/site/about/guidelines.xhtml

Barts and The London
School of Medicine and Dentistry

7.8. Key risks to delivering this research and contingencies 24
7.9. Procedure for Collecting Data 25
7.10. Including Case Report Forms (CRFs) and storage 25
7.11. Follow-up Procedures 25
7.12. Subject withdrawal (including data collection / retention for withdrawn participants) 25
7.13. Continued app use after trial period and app use by treatment as usual group 26
7.14. Schedule of Assessment 27
7.15. Criteria for Early Termination of the study 28
7.16. End of Study Definition 28
8. STATISTICAL CONSIDERATIONS 28
8.1. Sample Size 28
8.2. Statistical Analysis 28
9. ETHICS 30
10. SAFETY CONSIDERATIONS 31
11. DATA HANDLING AND RECORD KEEPING 31
11.1 Confidentiality 31
11.2. Required Study Documents 32
11.3. Record Retention and Archiving 33
12. PRODUCTS, DEVICES, TECHNIQUES AND TOOLS 33
12.1. Devices 33
12.2. Techniques and intervention 34
13. SAFETY REPORTING 35
13.1. Adverse Events (AE) 35
13.2. Serious Adverse Event (SAE) 35
13.3. Urgent Safety Measures 36
13.4. Annual Safety Reporting 36
13.5. Overview of the Safety Reporting Responsibilities 37
14. MONITORING & AUDITING 37
14.1. Auditing 37
14.2. Summary Monitoring Plan 37

For peer review only - http://bmjopen.bmj.com/site/about/guidelines.xhtml

14.3. Compliance 38
14.4. Non-Compliance 38
15. TRIAL COMMITTEES 39
15.1. Trial Steering Committee (TSC) 38
15.2. Trial Management Group (TMG) 39
15.3. Data Monitoring Committee (DMC) 39
16. FINANCE AND FUNDING 39
17. INDEMNITY 39
18. DISSEMINATION OF RESEARCH FINDINGS 40
REFERENCES 42
APPENDICES 46

For peer review only - http://bmjopen.bmj.com/site/about/guidelines.xhtml

Barts and The London
School of Medicine and Dentistry

1. GLOSSARY of Terms and Abbreviations

AE	Adverse Event
CI	Chief Investigator
CPP	Chronic Pelvic Pain
CRF	Case Report Form
DMC	Data Monitoring Committee
GCP	Good Clinical Practice
HCP	Health Care Professional
ICF	Informed Consent Form
JRMO	Joint Research Management Office
KTN	Katherine Twining Network
MHRA	Medicines and Healthcare products Regulatory Agency
NHS REC	National Health Service Research Ethics Committee
NHS R&D	National Health Service Research & Development
NPT	Normalization Process Theory
Participant	An individual who takes part in a clinical trial
PCTU	Pragmatic Clinical Trial Unit
PI	Principal Investigator
PIS	Participant Information Sheet
PSM	Patient Self-Management
QOL	Quality Of Life
QC	Quality Control
RCT	Randomised Controlled Trial
REC	Research Ethics Committee
RfPB	Research for Patients Benefit
SAE	Serious Adverse Event
SOP	Standard Operating Procedure
SUS	System Usability Scale
TAU	Treatment As Usual
TMG	Trial Management Group
TSC	Trial Steering Committee

For peer review only - http://bmjopen.bmj.com/site/about/guidelines.xhtml

Barts and The London
School of Medicine and Dentistry

2. SIGNATURE PAGES

Chief Investigator/Principal Investigator Agreement

The clinical study as detailed within this research protocol **(Version V8.0, dated 22 12 2016)**, or any subsequent amendments will be conducted in accordance with the Research Governance Framework for Health & Social Care (2005), the World Medical Association Declaration of Helsinki (1996) and the current applicable regulatory requirements and any subsequent amendments of the appropriate regulations.

Chief Investigator Name: Miss Elizabeth Ball
Chief Investigator Site: Barts and the London School of Medicine and Dentistry, Queen Mary University of London

Signature and Date: Elizabeth Ball **22.12.2016**

Statistician Agreement

The clinical study as detailed within this research protocol **(Version V8.0, 22 12 2016)**, or any subsequent amendments will be conducted in accordance with the Research Governance Framework for Health & Social Care (2005), the World Medical Association Declaration of Helsinki (1996) and the current applicable regulatory requirements and any subsequent amendments of the appropriate regulations.

Statistician Name: Mr Brennan Kahan
Statistician Site: Pragmatic Clinical Trials Unit, Queen Mary University of London

Signature and Date: **22.12.2016**

For peer review only - http://bmjopen.bmj.com/site/about/guidelines.xhtml

Barts and The London
School of Medicine and Dentistry

3. SUMMARY/SYNOPSIS

**Short Title**	MEMPHIS
**Methodology**	A randomised feasibility trial
**Research Sites**	This trial will be conducted at the Royal London and Whipps Cross Hospitals
**Objectives/Aims**	The overall aim is to assess the feasibility of implementing a trial of a mindfulness meditation intervention delivered by a mobile phone app for patients with chronic pelvic pain (CPP). The primary objectives are:<br>1) To provide feasibility data for a large multicentre RCT aimed at rigorously testing mindfulness meditation in CPP<br>2) To determine whether this app can be seamlessly integrated into clinical practice, especially CPP pathways
**Number of Participants/Patients**	90 women with CPP will be recruited and each randomised into one of the three trial groups (meditation app, progressive muscle relaxation or no app).
**Main Inclusion Criteria**	To be eligible for the MEMPHIS study, the women must:<br>● Be age 18 or over<br>● Have either organic or non-organic chronic pelvic pain lasting for 6 months or more<br>● Have access to a personal computer or smartphone.<br>● Understand simple spoken English
**Statistical Methodology and Analysis (if applicable)**	Feasibility outcomes will be summarised using descriptive statistics. Clinical outcomes will be analysed using linear mixed-effects models, and results will be presented as a difference in means and a 95% confidence interval.

For peer review only - http://bmjopen.bmj.com/site/about/guidelines.xhtml

	Usability and integration into clinical practice will be explored in focus groups or via telephone interviews with participants.<br>Some participants will be asked to elaborate about app satisfaction and also on clinical outcomes. Results will be analysed using content analysis including both thematic and text word analysis.
**Proposed Start Date**	November 2015
**Proposed End Date**	August 2017
**Study Duration**	22 months

For peer review only - http://bmjopen.bmj.com/site/about/guidelines.xhtml

Barts and The London
School of Medicine and Dentistry

4. INTRODUCTION

4.1. Background

Chronic pelvic pain (CPP) is defined as intermittent or constant pain in the lower abdomen or pelvis of a woman for at least 6 months, not exclusively associated with menstruation, intercourse and not associated with pregnancy [1].

It affects up to 24% women worldwide [2], accounts for 20% of UK gynaecological clinic referrals [3], and has a considerable impact on patients' quality of life and their income. CPP costs the NHS € 3.3bn per year [4]. Despite costly interventions, CPP is often resistant to surgical and medical treatment. Multifactorial psychological and somatic causes require a multidimensional approach, which is not routinely offered in gynaecology clinics [5]. Evidence from randomised controlled trials (RCTs) suggests that psychological interventions may be superior to primary surgery [6]. Although psychological treatment is provided across the NHS, mostly in the context of the primary care programme *Improving Access to Psychological Therapies* there are problems with capacity, waiting times, and the overall number of patients being able to access services. Alternatively, patient self-management (PSM) is now recognised as a tool empowering patients to cope better with their condition [7]. Mindfulness meditation is a potentially valuable PSM tool in CPP. We conducted a systematic search of literature (07/2013, updated 12/2013) and found no RCTs of mindfulness meditation in CPP. However, we identified two small, non-randomised pilot trials investigating the effect of mindfulness meditation on pain (one in women with CPP and one in women with endometriosis) both of which showed promising results [8,9].

Because we identified no RCTs on mindfulness meditation in CPP in our systematic review, we included other chronic pain conditions which may have a similar patho-mechanism to pelvic pain, such as back pain, headache, fibromyalgia and diabetic neuropathy. We assume that any benefits of mindfulness meditation in these conditions may also be seen in CPP.

We found previous systematic reviews in these conditions had a number of limitations, such as not reporting effect sizes [10-12].

For peer review only - http://bmjopen.bmj.com/site/about/guidelines.xhtml

Our systematic review conducted in lines with current standards [13] identified 472 relevant citations. Nine RCTs met fully the review's inclusion criteria [14,15,16-22]. Most studies were of moderate quality; but sample sizes were generally small (from 65 women for quality of life in mental health domain to 259 women for depression).

4.2. Effect of Mindfulness based meditation in chronic pain patients

Our results showed Mindfulness based meditation reduced depression levels in chronic pain patients (standardised mean difference (SMD) -0.28; 95% CI -0.53, -0.03; p = 0.03)). Patients who received Mindfulness meditation tended to cope better with anxiety (SMD -0.16, 95% CI -0.47, 0.15) and affective pain (the emotional reaction to pain) (SMD -0.13, 95% CI -0.42, 0.16). Women in the intervention arm had also higher Quality of life (QOL) scores (especially the mental health component SMD 0.65, 95% CI -0.27, 1.58) and higher pain acceptance (SMD 0.53, 95% CI -0.13, 1.19); although these results were not statistically significant. Only one of the included studies reported the important measure of pain acceptance.

Currently Mindfulness-based therapy is creating lively research interest. Two recent systematic reviews report positive effects on somatisation disorders [23] and psychological stress [24].

4.3. On-going studies

Although there are currently no on-going studies of Mindfulness in patients with CPP that we are aware of, there are other NIHR funded studies with overlapping themes**.**

Self help in CPP

The RFPB-funded study SUPPORT, which is currently in follow- up (MREC 10/H1005/24), is investigating an evidence-based self-care guidance in general practice for women with CPP. GPs received training to use the guidance in their consultations. Women were randomised to either receive the facilitated self-care guide or usual care. Results from SUPPORT will provide valuable information on how best to integrate a new patient self-help intervention into an existing patient pathway.

For peer review only - http://bmjopen.bmj.com/site/about/guidelines.xhtml

Barts and The London
School of Medicine and Dentistry

Interactive mobile phone application to modify patient behaviour

The recently closed RFPB-funded feasibility study STARFISH (MREC 12/WS/0309) investigated the acceptability of a smartphone app that encourages stroke patients to become more physically active. The number of steps taken per day by the individual is monitored. Patients work in small groups and different goals can be set for different individuals in the group, along with goals for the whole group. It will be interesting to compare the reported obstacles and facilitators to using the app with MEMPHIS.

Web-based delivery of an intervention

Of particular interest, due to the similarities in study design to MEMPHIS, is a recently closed pilot study, MIMS (UKCRN ID 13105) that investigated adjustment to multiple sclerosis.

In MIMS, meditation teaching was delivered by videoconference. Web-based delivery has also been explored and shown to be feasible for reducing stress, anxiety and depression [25]; both options are lacking the flexibility of a smartphone app, which we are proposing.

4.4. Implications for the further development of clinical or public health practice

Our co-investigator Judy Birch is closely involved with the committee that produces national guidelines for CPP patient care pathways, which she helps to develop [26]. If the app were proven to be effective in a phase III trial, it would be possible for it to be incorporated in this pathway.

One outcome measure of MEMPHIS is to determine whether this app can be integrated into clinical practice, especially CPP pathways. If this is the case there would be benefit from studying how to extend the app to other pain conditions, such as headache, back pain and irritable bowel syndrome, in which face-to face delivered mindfulness meditation has had positive effects [23].

For peer review only - http://bmjopen.bmj.com/site/about/guidelines.xhtml

Barts and The London School of Medicine and Dentistry

If this app is shown to be effective in a phase III trial, we will collaborate closely with Headspace, our local Health and Education Cluster and Queen Mary to implement this app both locally and nationally.

4.5. Potential impact on local policy making and improvement in service delivery

Chronic pelvic pain patients would benefit from multiple treatment approaches [6] but currently most gynaecological departments only offer medical and/or surgical treatment [5]. Although psychological treatment is provided across the NHS, mostly in the context of the primary care programme *Improving Access to Psychological Therapies* there are problems with capacity, waiting times, and the overall number of patients being able to access services. If the app is proven to be useful in a phase III RCT this gap could be filled, without having to employ more psychologists, because the interventions would be largely app delivered. Locally this would help our concerns about access to psychological treatment for CPP. Given the ubiquity of the app, greater compliance with treatment and less wastage from patients not attending appointments is expected. The use of the app in local primary, secondary and tertiary care settings would be introduced in collaboration with GP commissioning groups through local guidelines and protocols.

5. TRIAL OBJECTIVES

5.1. Aims and Objectives

The overall aim is to assess the feasibility of implementing a trial of a mindfulness meditation intervention delivered by a mobile phone app for patients with chronic pelvic pain (CPP). The primary objectives are:

- To provide feasibility data for a large multicentre RCT aimed at rigorously testing Mindfulness meditation in patients with CPP. The full-scale trial will assess the effectiveness of the mindfulness meditation app in patients with chronic pelvic pain in a national multicentre RCT

For peer review only - http://bmjopen.bmj.com/site/about/guidelines.xhtml

Barts and The London
School of Medicine and Dentistry

- To determine whether this app can be seamlessly integrated into clinical practice, especially CPP pathways. In cooperation with the Pelvic Pain Support Network, which is instrumental in the initiative on implementing nationwide pathways for patients with CPP, we will review the data on feasibility, especially the patient feedback and process analysis to answer this question to find out if the app, if it has been shown to be effective could be incorporated straight away into a national clinical pathway for CPP patients

5.2. Feasibility outcomes

5.2.1. Feasibility outcomes collected from participants

- Duration of recruitment (measured from the day recruitment opens until the day the 90th patient is randomised).
- Estimates to be used for the sample size calculation of the phase III RCT (the estimated SD for pain acceptance, and the dropout rate).
- Patient adherence to app use will be measured by the following outcomes:
    - Number of days (within the first 60 days from randomisation) a patient has used the app (with app use defined as having completed at least 90% of a session).
    - Whether the patient has used the app on 22 or more days within the first 60 days from randomisation.
    - Number of weeks (within the first eight weeks from randomisation) a patient has used the app on three or more days.
    - Whether the patient has used the app on three or more days in 6 or more weeks (within the first eight weeks from randomisation).
    - Whether the patient has used the app on 22 or more days within the first 60 days from randomisation, AND used the app on three or more days in 6 or more weeks within the first eight weeks from randomisation.
- Reasons for patient non-adherence to app use.

For peer review only - http://bmjopen.bmj.com/site/about/guidelines.xhtml

Barts and The London
School of Medicine and Dentistry

5.2.2. Feasibility outcomes collected from participant focus groups

- Usability and integration into clinical practice will be explored in two focus groups post-intervention with approximately 15 app participants, who have completed the 60 day follow up. Alternatively, participants unable to attend focus groups will be given the chance to answer a questionnaire over the phone with a research nurse.
- Discussions will be recorded and literal themes on integration and usability will be evaluated for in depth information. This information will be considered as well as adherence to the app as an indirect measure of acceptability. In cooperation with the Pelvic Pain Support Network, which is instrumental in the initiative on implementing nationwide pathways for patients with CPP, we will review the data on feasibility, especially the patient feedback and process analysis to answer this question to find out if the app, If it has been shown to be effective could be incorporated straight away into a national clinical pathway for CPP patients.
- We will determine primary and secondary outcomes of interest from the perspective of patients, for a full-scale trial. This will involve asking participants who were randomised to the app groups to discuss and prioritise outcomes.
- Obstacles to recruitment will also be explored.

5.2.3. Feasibility outcomes collected from health care practitioner focus groups

- A purpose made topic guide will be used to structure a focus group with service providers and based on the NPT toolkit [27] and the Diffusion of Innovations Theory [28] as a prompt for the facilitator.

The service providers will be asked to consider their role and their organisation and to suggest and discuss any issues to integration, and also – unlike conventional qualitative research focus groups – to suggest potential solutions. Discussions will be based around Diffusion of Innovations Theory, that is, we will consider:

- Relative advantage vs. existing practices

For peer review only - http://bmjopen.bmj.com/site/about/guidelines.xhtml

Barts and The London School of Medicine and Dentistry

- Compatibility with existing practices
- Simplicity and ease of integration
- Trialability and reinvention of the process
- Feedback (e.g. can clinicians see that patients benefit?)
- Peer to peer networking

We will use our findings to develop our integration approach to be further explored in the subsequent full trial.

- Obstacles to recruitment will also be explored.

5.3. Clinical outcomes

- Quality of life score, Physical Functioning subscale (as measured by the RAND Short Form (36) Health Survey (SF-36))
- Quality of life score, Social Functioning subscale (as measured by the RAND SF-36)
- Quality of life score, Pain subscale (as measured by the RAND SF-36)
- Quality of life score, General Health subscale (as measured by the RAND SF-36)
- Depression score (as measured by the Hospital Anxiety and Depression Scale (HADS))
- Anxiety score (as measured by HADS)
- Mindfulness score (as measure by the Cognitive and Mindfulness - Revised (CAMS – R) scale)
- Pain related disability score (as measured by the Chronic Pain Grade (CPG) disability subscale)
- Self efficacy score (as measured by the Pain Self-Efficacy Questionnaire (PSEQ))
- Pain acceptance score (as measured Chronic Pain Acceptance Questionnaire (CPAQ-8))
- Sexual Health Outcomes score (as measured by Sexual Health Outcomes in

For peer review only - http://bmjopen.bmj.com/site/about/guidelines.xhtml

Barts and The London
School of Medicine and Dentistry

Women Questionnaire (SHOW-Q))

- Subjective outcome score (as measured by Measure Yourself Medical Outcome Profile (MYMOP))

All clinical outcomes will be analysed at 60 days, 3 months, and 6 months post-randomisation.

6. METHODOLOGY

6.1. Inclusion Criteria

To be eligible for the MEMPHIS study, the women must meet the following criteria:

- Aged 18 or over
- Women with organic and non-organic chronic pelvic pain lasting for six months or more
- Be capable of understanding the information provided, with use of an interpreter if required and being able to understand simple English as is used in the app
- Give written informed consent

6.2. Exclusion Criteria

Patients who mee**t the following criteria are** ineligible to participate:

- No access to a Personal computer or smartphone
- Current users of the Headspace app content available to the public

6.3. Study Design

MEMPHIS is a randomised, single centre feasibility trial. All eligible women referred to the chronic pelvic pain clinics at the Royal London and Whipps Cross Hospitals (both new and existing patients) will be approached to take part in the study. A study leaflet will be given to them, providing brief information of the study and informing them that they are invited to participate. After informed consent, we will randomise

eligible women in a 1:1:1 ratio (30 participants in each group) to one of the three treatment groups:

Group A - "Intervention": 60 days of the app delivering mindfulness meditation content (in addition to usual care). See section 7.4 for a detailed description.

Group B - "Active control": 60 days of the app delivering progressive muscle relaxation content (in addition to usual care). See section 7.4 for a detailed description.

Group C - Treatment as usual (TAU): Usual care

Setting: NHS Tertiary care hospital

6.4. Study Scheme Diagram

Figure 1

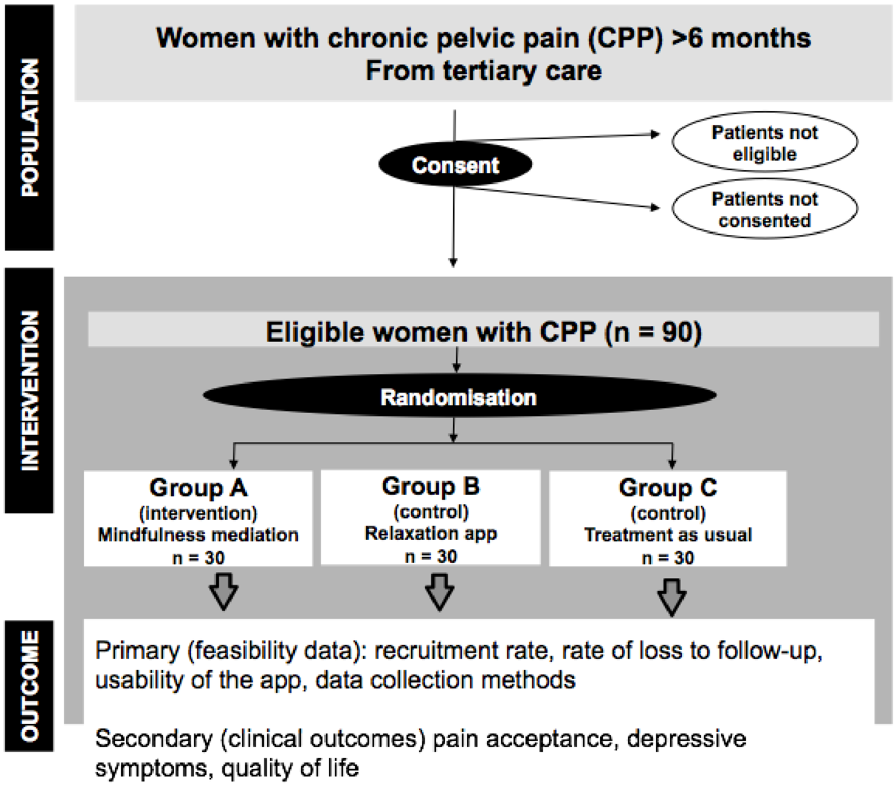

For peer review only - http://bmjopen.bmj.com/site/about/guidelines.xhtml

7. STUDY PROCEDURES

7.1. Informed Consent Procedures

Women will be made aware of the study by a health care professional and through promotional material. Potentially eligible patients will receive the PIS along with their hospital appointment invitation to ensure they have adequate time (at least 24 hours) to consider the trial. The PIS will be accompanied with a letter from the PI informing the women that they may be approached about the study at their appointment. Eligible patients who are seen in clinics other than pelvic pain and endometriosis clinics will be given the PIS and contact details for the research practitioner so they can benefit from participating in MEMPHIS should they wish so.

The PIS will be reviewed and the patient will have the opportunity to ask any questions. All eligible participants willing to consent will be asked to sign the consent form. Women will be provided with the contact details of the researcher, and informed that they have the right to withdraw their consent at any stage. Some women may be asked for permission to be contacted by a research practitioner at a later stage for enrolment if there are time constraints.

Only those on the delegation log will be able to consent for the intervention. The consenting staff will have thorough knowledge of research governance issues surrounding consent and will be fully conversant with the protocol.

If they are eligible but do not wish to consent, this will be recorded. For the full scale trial we need to understand how many eligible patients need to be approached to reach the recruitment target. We also would like to identify if eligible women opt out of the study due to a rectifiable issue.
Women who give their approval will be randomised. The investigator (or another qualified person) will explain to the potential participant that they are free to refuse any involvement within the study or alternatively withdraw their consent at any point during the study and for any reason.

For peer review only - http://bmjopen.bmj.com/site/about/guidelines.xhtml

Barts and The London School of Medicine and Dentistry

If there is any further safety information, which may result in significant changes in the risk/benefit analysis, the PIS and Informed Consent Form (ICF) will be reviewed and updated accordingly. All participants who are actively enrolled on the study will be informed of the updated information and given a revised copy of the PIS/ICF in order to confirm their wish to continue on the study (if feasible), if it may change their willingness to participate. A copy of the consent form will be given to the participant; one will be kept in the hospital notes and the original will be placed in the Investigator Site File.

7.2. Screening and enrolment

New referrals and existing patients at the pelvic pain clinic are equally eligible. Through links with the Katherine Twining network and UCL partners we have established networks that can advertise recruitment. Based on these circumstances we are confident that we can achieve successful recruitment in the given timeframe.

Patients will be sent the Patient Information Sheet (PIS) in advance to ensure they have adequate time to consider the trial. The PIS will be accompanied with a letter from the PI informing patients that they may be approached about the study at their appointment.

At the appointment, the research practitioner will assess the women according to the inclusion/exclusion criteria detailed above and explain the nature of the intervention. The PIS will be reviewed and the patient will have the opportunity to ask any questions. All eligible participants willing to consent will be asked to sign the consent form. If a woman has not read or received the PIS before their appointment, the research team will go through the PIS with the individual in person. Women will be giving as much time as they want to consider the study before consent is taken. Women will be provided with the contact details of the researcher, and informed that they have the right to withdraw their consent at any stage.

For peer review only - http://bmjopen.bmj.com/site/about/guidelines.xhtml

Barts and The London
School of Medicine and Dentistry

7.3. Randomisation Procedures

After informed consent, patients will be randomised in a 1:1:1 ratio to one of the three treatment groups, using permuted blocks without stratification. Randomisation will be performed using a centralised internet service, hosted by the Pragmatic Clinical Trials Unit. The schedule of intervention with timeline is detailed below.

7.4. Blinding

When a participant is randomised the randomisation system will only display whether they have been allocated to an "app" treatment group (either the "Intervention" or "Active Control" group, but not which one) or the "Treatment as usual" group. If a participant is randomised to either "app" treatment group, then the randomisation system will supply an alphanumeric token which is redeemed when registering to receive the app. This will ensure that the correct content (mindfulness meditation or progressive muscle relaxation) is delivered to each participant. Therefore, the participant and recruiting staff will NOT be blinded to allocation of the "Treatment as usual" or "app" groups. However, at randomisation they will be blinded to whether allocation is to "Intervention" or "Active Control" group.

To preserve blinding of participants as much as possible, "Intervention" and "Active Control" groups will be using the same app, and hearing instructions for the same duration, delivered by the same narrator. Only the content of the instructions will differ. In addition, the Patient Information Sheet and consent form do not explicitly refer to "mindfulness meditation" or "progressive muscle relaxation".

Outcomes are collected in paper questionnaires completed by participants. The 6 month questionnaire includes a question to determine whether the participants randomised to the app have been unblinded to the "Intervention" app or "control" app. The researcher will answer a short questionnaire after recruiting each participant to determine if they have been unblinded to the "Intervention" app or "control" app, for participants randomised to an app.

Statisticians will be blinded to individual treatment allocations until required for the final analysis. If necessary, an independent statistician will perform any interim analysis which require unblinding of the data.

For peer review only - http://bmjopen.bmj.com/site/about/guidelines.xhtml

It is not anticipated that any emergency unbinding will be necessary.

7.5. Planned interventions

After eligible women have been allocated to one of the 3 groups, the participants in the Intervention and the Active Control group (progressive muscle relaxation app) will receive a face-to-face introduction to using the app. After that, the Intervention group will use the app over 60 days.

The meditation content is a structured and progressive course, layering in new techniques and concepts over successive sessions. The course was created and narrated by a former monk - Andy Puddicombe - drawing on a secularised version of the techniques he was taught over 10 years' experience in monasteries around the world.

The techniques used in the Intervention are shown in the table below. The first 30 days cover basic techniques, assuming no previous experience of meditation. The second 30 days focus specifically on the use of these techniques with respect to pain. The duration of individual sessions builds over time. Days 1-10 are 10 minutes in duration, days 11-20 are 15 minutes in duration, and days 21-60 are 20 minutes in duration.

The Active Control group will use the same app, but the app will be configured so that they will hear a series of non-meditative progressive muscle relaxation instructions, also narrated by Andy Puddicombe. These sessions will be identical every day, except that their duration will increase to mirror the increasing duration of the meditation content being listened to by the Intervention group.
In this way, both Intervention and Control groups will be using the same app, and hearing instructions for the same duration, delivered by the same narrator. Only the content of the instructions will differ.

For peer review only - http://bmjopen.bmj.com/site/about/guidelines.xhtml

Barts and The London School of Medicine and Dentistry

Series	Techniques involved
Take 10/Foundation 1 (first 10 days)	Open monitoring, body scan, breath as anchor
Foundation 2 (days 11-20)	As above, plus intention and altruism
Foundation 3 (days 21-30)	As above, plus integration of mindfulness with daily activities
Pain series (days, 31-60)	As above, plus visualisation and enquiry (insight/Tibetan vipassana)

7.6. Concomitant Medications

Patients are able to receive any concomitant medications that they would as part of usual care.

7.7. Reasons for non progression to full trial

- Insurmountable problems with recruitment
- Extremely high rates of loss-to-follow-up
- Extremely low rates of adherence to the intervention
- Unacceptability of intervention for patients

7.8. Key risks to delivering this research and contingencies:

- Recruitment of 90 patients between May 2016 and October 2016 not achieved – regular monitoring throughout recruitment period to identify and resolve problems (e.g. open new centres/extend recruitment period)
- We will monitor regularly if patients have not downloaded apps and offer further one-to-one support
- Data collection issues will be monitored and addressed early where possible; this will inform the full-scale RCT design

For peer review only - http://bmjopen.bmj.com/site/about/guidelines.xhtml

- Issues relating to the other milestones (ethics, personnel, app availability) and deliverables will be rectified, but potentially delay the start of MEMPHIS/full-scale trial. Contamination was not thought likely by the patient group

7.9. Procedure for Collecting Data

Patients will enter the data on paper questionnaires, which will be transferred into a purpose-built electronic database.

1.) Scales for clinical outcomes
2.) App satisfaction questionnaire, which includes open comment boxes and tick-boxes based on published questionnaires [30].

As an incentive to complete and return the patient questionnaires, a £5 shopping voucher will be sent in the post with each follow up questionnaire alongside a stamped addressed envelope.
In the case that a questionnaire is not received, participants will be sent a text reminder. Non-responders will then be contacted by telephone in order to collect a smaller dataset.

7.10. Including Case Report Forms (CRFs) and storage

In line with GCP guidance we will keep the data stored for 20 years following the close of the study to allow for verification and any further data sharing e.g. individual patient data meta-analysis.

We will follow the PCTU's standard operating procedures for legacy archiving.
Queen Mary University of London will act as custodians of the data.

7.11. Follow-up Procedures

Some of the participants will be asked for permission to elaborate on the open comment boxes about app satisfaction and also on clinical outcomes in two focus

For peer review only - http://bmjopen.bmj.com/site/about/guidelines.xhtml

groups to be held after the 6 month follow up point finisheswith participants asked to discuss and prioritise outcomes. Alternatively, participants unable to attend focus groups will be given the option to answer a questionnaire over the phone with a researcher.

7.12. Subject withdrawal (including data collection / retention for withdrawn participants)

A participant can be withdrawn from the trial if, in the opinion of the investigator or the care providing clinician or clinical team, it is medically necessary to do so.

With any post randomisation exclusions, the study personnel will make every effort to obtain, and record, information about the reasons for violation, any adverse events and to follow-up the women for all safety and efficacy outcomes, as appropriate. If a woman decides after randomisation she does not wish to participate any further in the MEMPHIS trial, she may withdraw herself from the trial. We will aim to document the reason for self-withdrawal. Clear distinction will be made as to whether the participant is withdrawing from trial whilst allowing further follow-up, or whether the participant refuses any follow-up. If a participant explicitly withdraws consent to have any further data recorded their decision will be respected and recorded on the final study form. All communication surrounding the withdrawal will be noted in the study records and no further data will be collected for that participant. They will be returned to the NHS standard practice for follow up care.

If a woman loses their ability to consent during participation in the trial, they will be withdrawn from the trial and no further data will be collected from the participant unless consent for this was explicitly obtained prior to the loss of capacity.

7.13. Continued app use after trial period and app use by treatment as usual group

It was decided to permit continued app use to the end of the study to reflect the situation in real life. Duration of use will be recorded through the app without using patient identifiable data.

For peer review only - http://bmjopen.bmj.com/site/about/guidelines.xhtml

Consideration was given to inform patients in the 'treatment as usual' arm at the beginning that they will be able to access the meditation app at the end of the study, but this was abandoned due to concerns that this could lead to bias. Research has shown [31] that in those circumstances patients may decide to 'wait' until the end of the intervention before trying to improve, and as a consequence, they tend to improve less, leading to overestimating the effect of the intervention. It is possible that without the offer of delayed app use recruitment may be slower, which is something we would like to determine in the feasibility study. However, if after close involvement with the PPI this appears to be not acceptable to patients as compromise such as telling control patients after the end of the study that they are now allowed to use the app may be offered.

7.14. Schedule of Assessment

Health outcome measures are collected at baseline. The delivery of the intervention or control will occur for 60 days. Health outcome measures are collected immediately after the intervention at 60 days, and again at 3 and 6 months. App satisfaction/usability measures will be collected immediately after the intervention at 60 days from app participants.

The usability and clinical outcome focus groups will take place after the 6 month follow up point.

Assessment	Baseline	During intervention	60 days post randomisation	3 months post randomisation	6 months post randomisation
Questions about participants pain	V				
History of pain treatment	V				
Personal details	V				
Adherence to app use		V			
Clinical outcome questionnaires	V		V	V	V

For peer review only - http://bmjopen.bmj.com/site/about/guidelines.xhtml

Barts and The London
School of Medicine and Dentistry

App satisfaction questionnaires			V		
Interview/focus group with recruiters, nurses, patients, other stakeholders on usability and integration into practice					V
HCP and patient focus groups on clinical outcomes					V

For peer review only - http://bmjopen.bmj.com/site/about/guidelines.xhtml

Barts and The London
School of Medicine and Dentistry

7.15. Criteria for Early Termination of the study

The nature of the intervention and follow−up makes it unlikely that any new information will impact an individual participant. If the TSC committee, REC, CI or sponsor determine it is within the best interests of the participants or trial to terminate the study, written notification will be given to the CI. This may be due to, but not limited to; safety concerns, proof of efficacy or non-compliance/serious breaches. If the study is terminated participants will be returned to the NHS normal follow up and routine care.

7.16. End of Study Definition

When the last enrolled participant has completed follow up, the REC will be notified of the trial completion. The final study report will be completed within 12 months after the trial completion.

8. STATISTICAL CONSIDERATIONS

8.1. Sample Size

30 participants will be recruited to each of the three treatment groups, giving a total of 90 participants. As this is a feasibility study, we have not performed a sample size calculation based upon the power to detect a significant treatment effect on a clinical outcome. However, 90 participants should provide a reliable estimate for the standard deviation of the primary clinical outcome (likely to be pain acceptance) [32, 33], which can be used to inform the sample size calculation of the main trial.

8.2. Statistical Analysis

A full analysis plan will be developed and agreed prior to any analysis or unblinding of the data.

For peer review only - http://bmjopen.bmj.com/site/about/guidelines.xhtml

Barts and The London
School of Medicine and Dentistry

Baseline

Baseline variables will be presented for each treatment group as the mean (SD) or median (IQR) for continuous variables, and the number (%) for categorical variables.

Analysis of Feasibility Outcomes

Feasibility outcomes will be presented for each treatment group as the mean (SD) or median (IQR) for continuous variables, and the number (%) for categorical variables.

Duration of recruitment will be calculated as the number of days from the beginning to the end of recruitment. The number of participants recruited per month will be presented.

The proportion of patients in each treatment group who have returned data at each follow-up time point (60 days, 3 months, and 6 months post-randomisation) will be presented. Summaries of baseline variables will be presented separately for patients who have and have not returned data at each at the 6 month time point.

Adherence outcomes will be summarised separately for the intervention and active control treatment groups. Adherence outcomes will be presented as the mean (SD) or median (IQR) for continuous variables, and the number (%) for categorical variables.

An estimate of the standard deviation of pain acceptance (CPAQ) in each treatment group at each follow up time point (60 days, 3 months, and 6 months) will be presented.

Analysis of Clinical Outcomes

For each clinical outcome we will present the following information:

- The number of patients in each treatment group with an observed outcome at each follow-up time point.

For peer review only - http://bmjopen.bmj.com/site/about/guidelines.xhtml

Barts and The London School of Medicine and Dentistry

- The mean (SD) in each treatment group at each follow-up time point.
- The estimated treatment effect at each follow-up time point, with a 95% confidence interval.

Estimates of treatment effect will be presented comparing the intervention group (mindfulness meditation app) to the control (treatment as usual) group, the intervention group to the active control (progressive muscle relaxation app) group, and the active control group to the control (treatment as usual) group. Outcomes will be analysed using linear mixed-effects models to account for the correlation between patient outcomes at different follow-up time points [34], and adjusted for baseline measure of the outcome [35]. Patient data will be analysed according to the treatment group to which they were randomised (intention-to-treat). All patients with an observed outcome for at least one of the three follow-up time points (60 days, 3 months, or 6 months) will be included in the analysis [36].

Analysis of usability and integration of app

- Obstacles to recruitment will be summarised
- The integration of the app into existing and emerging patient pathways will be investigated using questionnaires developed from social contagion theory and Normalisation Process Theory (NPT) as described in section 5.3. The maximum total score using NPT is 64. The maximum total score using the Diffusion of Innovations questionnaire is 200.
  The System Usability Scale (SUS) [28] has a maximum score of 50.

9. ETHICS

The Investigator to an Independent Research Ethics Committee will submit this protocol and any subsequent amendments, along with any accompanying material provided to the participant in addition to any advertising material. Written Approval from the Committee will be obtained and subsequently submitted to the JRMO to

For peer review only - http://bmjopen.bmj.com/site/about/guidelines.xhtml

Barts and The London School of Medicine and Dentistry

obtain Final R&D approval. The trial can only start after approval from a Research Ethics Committee and the local R&D “Sign-off” from the participating centre. If there is any further safety information, which may result in significant changes in the risk/benefit analysis, the Patient Information Sheet (PIS) and Informed Consent Form (ICF) will be amended accordingly and submitted to REC for revision and approval. All participants that are actively enrolled on the study will be informed of the updated information and given a revised copy of the PIS/ICF in order to confirm their wish to continue on the study (if feasible), if it may change their willingness to participate.

10. SAFETY CONSIDERATIONS

There are no known side effects arising from mindfulness meditation.

11. DATA HANDLING AND RECORD KEEPING

11.1. Confidentiality

Patient anonymity is protected and maintained. This applies to data collected on paper or via the headspace database.

We will ensure that patient identities are protected from any unauthorised parties. Information with regards to study patients will be kept confidential and managed in accordance with data Protection Act, NHS Caldicott Guardian, The research Governance Framework for Health and Social care and Research Ethics Committee Approval.

The trial will collect personal data and sensitive information about the participants either directly or from their clinical team. Participants will be informed about the transfer of this information to the study office and will be asked to consent to this. The data will be entered onto a secure computer database, either by trials unit staff or directly via a secure Internet connection. Any data to be processed will be anonymised. All personal information obtained for the trial will be held securely and treated as (strictly) confidential. All staff, at the hospital or the trials unit shares the

For peer review only - http://bmjopen.bmj.com/site/about/guidelines.xhtml

Barts and The London
School of Medicine and Dentistry

same duty of care to prevent unauthorised disclosure of personal information. No data that could be used to identify an individual will be published.

In relation to the data collected by Headspace the following applies:

Headspace will not collect any clinical data, but data on app usage. Details collected on the headspace database will be confidential. Details about the individual's use of Headspace tools will never be seen by or shared with anyone outside the research team and the company. Individual usage and demographic information will only be used by Headspace in accordance with the standard Headspace user terms and conditions. No data will be shared with any other organizations, unless with prior agreement, and all data is kept confidential. App usage data will be transferred to the research team via a securely encrypted file.

The Chief investigator, Miss Elizabeth Ball is the “custodian” of the data.

11.2. Required Study Documents

- A signed protocol and any subsequent amendments
- PCTU self-monitoring template for the trial team to complete on a regular basis as detailed by the Trial Monitoring section
- Current and Superseded Patient Information Sheets
- Current and Superseded Consent Forms
- Current and Superseded GP letters
- Current and Superseded Posters
- Current and Superseded CRFs
- Indemnity documentation from sponsor
- Conditions of Sponsorship from sponsor
- Conditional/Final R&D Approval
- Signed site agreements
- Ethics submissions/approvals/correspondence
- CVs and GCP certificates of CI and site staff

For peer review only - http://bmjopen.bmj.com/site/about/guidelines.xhtml

- Laboratory accreditation letter, certification and normal ranges for all laboratories to be utilised in the study
- Delegation log
- Staff training log
- Identification log
- Enrolment log
- Monitoring visit log
- Correspondence relating to the trial
- SAE reporting plan for the study

11.3. Record Retention and Archiving

During the course of research, all records are the responsibility of the Chief Investigator and must be kept in secure conditions. When the trial is complete, it is a requirement of the Research Governance Framework and Trust Policy that the records are kept for a further 20 years. For trials involving Barts Health Trust patients, undertaken by Trust staff, or sponsored by Barts Health trust or QMUL, the approved repository for long-term storage of local records is the Trust Modern Records centre, which is based at 9 Prescott Street.

12. PRODUCTS, DEVICES, TECHNIQUES AND TOOLS

12.1. Devices

The Medicines and Healthcare products Regulatory Agency (MHRA) states that some apps can be classified as medical devices. [37]

However, apps with software that provides general information but does not provide personalised advice, although it may be targeted to a particular user group, is unlikely to be considered a medical device. We believe that neither the mindfulness meditation nor the progressive muscle relaxation content in the app fulfil the criteria for medical devices.

For peer review only - http://bmjopen.bmj.com/site/about/guidelines.xhtml

12.2. Techniques and interventions

Intervention (mindfulness meditation content):

60 days of guided meditation content. The first 30 days cover basic techniques, assuming no previous experience of meditation. The second 30 days focus specifically on the use of these techniques with respect to pain. The duration of individual sessions builds over time. The first 10 days are each 10 minutes in duration. The next 10 days are each 15 minutes in duration. All following days are 20 minutes in duration. The minimum usage of app should be for at least 22 out of 60 days.

It was decided to permit continued app use to the end of the study to reflect the situation in real life. Duration of use will be recorded through the app without using patient identifiable data.

Control:

1) Treatment as usual (watch and wait, medication and/or surgery) to investigate if any app intervention makes a difference to wellbeing and to ascertain dropout rates for the full-scale trial in patients who perceive that they are getting no intervention

2) 60 days of progressive muscle relaxation content: This group will use the same app as the Intervention group, but the app will be configured so that they will hear a series of non-meditative progressive muscle relaxation instructions. These sessions will be identical every day, except that their duration will increase to mirror the increasing duration of the meditation content being listened to by the Intervention group (10 minutes a day for 10 days, then 15 minutes a day for 10 days, then 20 minutes a day thereafter.)

App satisfaction questionnaires

- Purpose made questionnaire (Carol Rivas)
- The System Usability Scale (SUS) [28]

For peer review only - http://bmjopen.bmj.com/site/about/guidelines.xhtml

Barts and The London School of Medicine and Dentistry

13. SAFETY REPORTING

13.1. Adverse Events (AE)

An AE is any untoward medical occurrence in a subject to whom a medicinal product has been administered, including occurrences which are not necessarily caused by or related to that product. An AE can therefore be any unfavourable and unintended sign (including an abnormal laboratory finding), symptom or disease temporarily associated with study activities.
We do not expect SAEs related to use of the mindfulness or the progressive muscle relaxation app.

Notification and reporting Adverse Events or Reactions
If the AE is not defined as SERIOUS, the AE is recorded in the study file and the participant is followed up by the research team. The AE is documented in the participants' medical notes (where appropriate) and the CRF.

13.2. Serious Adverse Event (SAE)

A serious adverse event (SAE) is defined as an untoward occurrence that:

(a) results in death;
(b) is life-threatening;
(c) requires hospitalisation or prolongation of existing hospitalisation;
(d) results in persistent or significant disability or incapacity;
(e) consists of a congenital anomaly or birth defect; or
(f) is otherwise considered medically significant by the investigator.

An SAE occurring to a research participant should be reported to the main REC where in the opinion of the Chief Investigator the event was:

• Related – that is, it resulted from administration of any of the research procedures, and

For peer review only - http://bmjopen.bmj.com/site/about/guidelines.xhtml

• Unexpected – that is, the type of event is not listed in the protocol as an expected occurrence.

Notification and Reporting of Serious Adverse Events

Serious Adverse Event (SAEs) that are considered to be 'related' and 'unexpected' are to be reported to the sponsor within 24 hours of learning of the event and to the Main REC within 15 days in line with the required timeframe. For further guidance on this matter, please refer to NRES website and JRMO SOPs

13.3. Urgent Safety Measures

The CI may take urgent safety measures to ensure the safety and protection of the clinical trial subjects from any immediate hazard to their health and safety,. The measures should be taken immediately. In this instance, the approval of the REC prior to implementing these safety measures is not required. However, it is the responsibility of the CI to inform the sponsor and Main Research Ethics Committee (via telephone) of this event immediately.

The CI has an obligation to inform both the Main REC in writing within 3 days, in the form of a substantial amendment. The sponsor (Joint Research Management Office (JRMO)) must be sent a copy of the correspondence with regards to this matter. For further guidance on this matter, please refer to NRES website and JRMO SOPs.

13.4. Annual Safety Reporting

The CI will send the Annual Progress Report to the main REC using the NRES template (the anniversary date is the date on the MREC "favourable opinion" letter from the MREC) and to the sponsor. Please see NRES website and JRMO SOP for further information

For peer review only - http://bmjopen.bmj.com/site/about/guidelines.xhtml

Barts and The London
School of Medicine and Dentistry

13. 5. Overview of the Safety Reporting responsibilities

The CI/PI has the overall pharmaco-vigilance oversight responsibility. The CI/PI has a duty to ensure that safety monitoring and reporting is conducted in accordance with the sponsor's requirements.

14. MONITORING & AUDITING

14.1. Auditing

Definition: "A systematic and independent examination of trial related activities and documents to determine whether the evaluated trial related activities were conducted, and the data were recorded, analysed and accurately reported according to the protocol, sponsor's standard operating procedures (SOPs), Good Clinical Practice (GCP), and the applicable regulatory requirement(s)."

A study may be identified for audit by any method listed below:

1. A project may be identified via the risk assessment process.
2. An individual investigator or department may request an audit.
3. A project may be identified via an allegation of research misconduct or fraud or a suspected breach of regulations.
4. Projects may be selected at random. The Department of Health states that Trusts should be auditing a minimum of 10% of all research projects.
5. Projects may be randomly selected for audit by an external organisation.

Internal audits may be conducted by a sponsor's or funder representative.

14.2. Summary Monitoring Plan

Investigators and their host Trusts will be required to permit study-related monitoring and audits to take place, providing direct access to source data and documents as requested. Trusts may also be subject to inspection by the Research and Development Manager and should do everything requested by the Chief Investigator in order to

For peer review only - http://bmjopen.bmj.com/site/about/guidelines.xhtml

prepare and contribute to any inspection or audit. Study participants will be made aware of the possibility of external audit of data they provide in the participant information sheet.

14.3. Compliance

The CI will ensure that the trial is conducted in compliance with the principles of the Declaration of Helsinki (1996), and in accordance with all applicable regulatory requirements including but not limited to the Research Governance Framework, GCP, Trust and Research Office policies and procedures and any subsequent amendments.

14.4. Non-Compliance

Definition: A noted systematic lack of both the CI and the study staff adhering to the principles of the Declaration of Helsinki (1996), applicable regulatory requirements including but not limited to the Research Governance Framework, GCP, Trust and Research Office policies and procedures and any subsequent amendments, which leads to prolonged collection of deviations, breaches or suspected fraud.

These non-compliances may be captured from a variety of different sources including monitoring visits, CRFs, communications and updates. The sponsor will maintain a log of the non-compliances to ascertain if there are any trends developing or escalating. The sponsor will assess the non-compliances and action a timeframe in which they need to be dealt with. Each action will be given a different timeframe dependent on the severity. If the actions are not dealt with accordingly, the sponsor will agree an appropriate action, including an on-site audit.

15. TRIAL COMMITTEES

15.1. Trial Steering Committee (TSC)

The TSC provides independent supervision for the trial, providing advice to the Chief and Co-Investigators and the Sponsor on all aspects of the trial and affording

For peer review only - http://bmjopen.bmj.com/site/about/guidelines.xhtml

Barts and The London School of Medicine and Dentistry

protection for patients by ensuring the trial is conducted according to the principles of Good Clinical Practice in Clinical Trials. If the Chief and Co-Investigators are unable to resolve any concern satisfactorily, Principal Investigators, and all others associated with the trial, may write through the Trial Unit to the chairman of the TSC, drawing attention to any concerns they may have about the possibility of particular side-effects, or of particular categories of patient requiring special study, or about any other matters thought relevant.

15.2. Trial Management Group (TMG)

The trial management group will meet regularly to discuss operational issues. This will include the chief investigator, trial co-ordinator, senior research manager, statistician, data manager, QA manager and research administrator.

15.3. Data Monitoring Committee (DMC)

Based on the short duration of recruitment (expected to be 6 months) and the safety profile of the intervention, a DMC will not be used.

16. FINANCE AND FUNDING

-This study is funded by the Research for Patients Benefit national programme (RfPB).
- Headspace is donating subscriptions at no charge as part of their research initiative.

17. INDEMNITY

Queen Mary, University of London will act as a Sponsor, as defined by the Research Governance Framework for Health and Social Care (April 2005) for the project. The project will also be covered by the sponsor's insurance brokers on a "No Faults Compensation for Clinical Trials and/or Human Volunteer Studies". This policy will

For peer review only - http://bmjopen.bmj.com/site/about/guidelines.xhtml

Barts and The London School of Medicine and Dentistry

indemnify/cover the insured in respect of their legal liabilities arising out of the insured's activities.

18. DISSEMINATION OF RESEARCH FINDINGS

The research findings of the feasibility study will be disseminated judiciously to avoid biasing the full-scale trial. In both trials we will disseminate our findings to:

1) Study participants through a dedicated website and newsletters at the end of the feasibility and full scale study, guided by our lay advisers

2) Participating health care professionals through the dedicated website and electronic newsletters

4) Professional groups via peer-reviewed journals and scientific meetings. Post-trial workshops run in collaboration with PPI group

5) Health service commissioners via the study website and an electronic newsletter

6) The wider public through local and national media and via dedicated website

7) Patients and relatives through PPI group

Applicants have links for dissemination via these organisations: Cochrane reviews, NICE, Pelvic pain support network (Judy Birch), Katherine Twining Network (KTN), BJOG (Khalid Khan), BSGE (Elizabeth Ball) Communications experts at our higher education institutions and the NIHR Collaboration for Leadership in Applied Health Research and Care North Thames will support our dissemination strategy through Twitter, Facebook and press coverage.

A particular strength of our application is our close links with:

For peer review only - http://bmjopen.bmj.com/site/about/guidelines.xhtml

Barts and The London
School of Medicine and Dentistry

1) KTN, dedicated to research and education in the UK and abroad via the East London International Women's Health Appeal, who will be able to disseminate this low cost-intervention in developing countries with high incidence of CPP [2]

2) UCL partners, whose focus is on patient-led population-focused delivery of research innovations.

For peer review only - http://bmjopen.bmj.com/site/about/guidelines.xhtml

Barts and The London School of Medicine and Dentistry

REFERENCES

1. Royal College of Obstetricians and Gynaecologists, green top guidelines (2012) Available at: https://www.rcog.org.uk/en/guidelines-research-services/guidelines/gtg41 (Accessed: 13 May 2015)

2. Latthe, P., Latthe, M., Say, L., Gülmezoglu, M., Khan, K.S., (2006), 'WHO systematic review of prevalence of chronic pelvic pain: a neglected reproductive health entity', BMC Public Health. , 6(6), pp. 177.

3. Howard, F.M., (1993), 'The role of laparoscopy in chronic pelvic pain: promise and pitfalls', Obstetrical and Gynecological Survey, 48(6), pp. 357-387.

4. Simoens, S., et al., (2012), 'The burden of endometriosis: costs and quality of life of women with endometriosis and treated in referral centres', Human Reproduction, 27(5) pp. 1292-1299

5. Stones, R.W. and Price, C., (2002), 'Health services for women with chronic pelvic pain', Journal of the Royal Society of Medicine, 95, pp. 531-535

6. Peters, A.A., et al., (1991), 'A randomized clinical trial to compare two different approaches in women with chronic pelvic pain', Obstetrics and Gynecology, 77(5), pp. 740-4

7. Warsi, A., et al., (2004), 'Self-management education programs in chronic disease: a systematic review and methodological critique of the literature', Archives of Internal Medicine, 164(15), pp. 1641-9.

8. Fox, S.D., Flynn, E., Allen, R.H., (2011), 'Mindfulness meditation for women with chronic pelvic pain: a pilot study', The Journal of reproductive medicine, 56(3-4), pp. 158-162.

9. Kold, M., et al., (2012), 'Mindfulness-based psychological intervention for coping with pain in endometriosis', Nordic Psychology, 64(1), pp. 2-16.

10. Cramer, H., et al., (2012), 'Mindfulness-based stress reduction for low back pain. A systematic review', BMC Complementary & Alternative Medicine, 12, pp. 162.

11. Kozasa, E.H., et al., (2012), 'The effects of meditation-based interventions on the treatment of fibromyalgia', Current Pain & Headache Reports, 16(5), pp. 383-7.

12. Veehof, M.M., et al., (2011), 'Acceptance-based interventions for the treatment of chronic pain: A systematic review and meta-analysis', Pain, 152(3), pp. 533-42

For peer review only - http://bmjopen.bmj.com/site/about/guidelines.xhtml

13. Higgins, J.P.T., Green, S., (2011) 'Cochrane handbook for systematic reviews of interventions', version 5.1.0. Cochrane Collaboration. Available at: www.cochrane-handbook.org (Accessed: 13 May 2015)

14. Brown, C.A. and Jones, A.K., (2013), 'Psychobiological correlates of improved mental health in patients with musculoskeletal pain after a mindfulness-based pain management program', Clinical Journal of Pain, 29(3), pp. 233-44.

15. Carson, J., et al., (2005), 'Loving-kindness meditation for chronic low back pain: results from a pilot trial', Journal of Holistic Nursing, 23(3), pp. 287-304.

16. Morone, N.E., Greco, C.M., Weiner, D.K., (2008), 'Mindfulness meditation for the treatment of chronic low back pain in older adults: a randomized controlled pilot study', Pain, 134(3), pp. 310-9.

17. Nash-Mc Feron, D.E., (2006), 'Mindfulness in the treatment of chronic headache pain', Dissertation Abstracts International: Section B: The Sciences and Engineering, 67(5-B), pp. 2841.

18. Plews-Ogan, M., et al., (2005), 'A pilot study evaluating mindfulness-based stress reduction and massage for the management of chronic pain', Journal of General Internal Medicine, 20(12), pp. 1136-8.

19. Sagula, D. and Rice, K.G., (2004), 'The effectiveness of mindfulness training on the grieving process and emotional well-being of chronic pain patients', Journal of Clinical Psychology in Medical Settings, 11(4), pp. 333-342.

20. Schmidt, S., et al., (2010), 'Assessing a dynamical EEG pattern related to chronic pain-Results from a controlled evaluation of back pain patients as well as from an observational trial with a mindfulness based intervention', European Journal of Integrative Medicine, 2 (4), pp. 196.

21. Sephton, S.E., et al., (2007), 'Mindfulness meditation alleviates depressive symptoms in women with fibromyalgia: Results of a randomized clinical trial', Arthritis Care and Research, 57(1), pp. 77-85.

22. Teixeira, E., (2010), 'The effect of mindfulness meditation on painful diabetic peripheral neuropathy in adults older than 50 years', Holistic Nursing Practice, 24(5), pp. 277-83.

23. Lakhan, S.E. and Schofield, K.L., (2014), 'Mindfulness-Based Therapies in the Treatment of Somatization Disorders: A Systematic Review and Meta-Analysis', PLOS One, 8(8): e71834

For peer review only - http://bmjopen.bmj.com/site/about/guidelines.xhtml

24. Goyal, M., et al., (2014), ‘Meditation Programs for Psychological Stress and Well-being’, JAMA Internal Medicine., 174(3), pp. 357-368

25. Krusche, A., Cyhlarova, E., Williams, J.M., (2013), ‘Mindfulness online: an evaluation of the feasibility of a web-based mindfulness course for stress, anxiety and depression’, BMJ Open., 3(11): pp. e003498.

26. http://bps.mapofmedicine.com/evidence/bps/chronic_pelvic_pain_for_men_and_women_1.html

27. May, C., Sibley, A., Hunt, K., (2013) ’The nursing work of hospital-based clinical practice guideline implementation: an explanatory systematic review using normalisation process theory’, International Journal of Nursing studies, 51(2), pp.289-99

28. Rogers, E.M., (2003), Diffusion of Innovations, Fifth Edition, Free Press, New York, pp.221

29. Measuring Usability With The System Usability Scale (SUS), (2011) Available at: http://www.measuringu.com/sus.php, (Accessed: 13 May 2015)

30. Spook, J.E., et al., (2013), ‘Monitoring Dietary Intake and Physical Activity Electronically: Feasibility, Usability, and Ecological Validity of a Mobile-Based Ecological Momentary Assessment Tool’, Journal of Medical Internet Research, 15(9), pp. e214.

31. Cunningham, J.A., Kypros, K., McCambridge, J., (2013), ‘Exploratory randomized controlled trial evaluating the impact of a waiting list control design’, BMC Medical Research Methodology, 13(150)

32. Lancaster, G.A., Dodd, S., Williamson, PR., (2004), ‘Design and analysis of pilot studies: recommendations for good practice’, Journal of Evaluation in Clinical Practice, 10(2), pp.307-12

33. Teare, MD., Dimairo, M., Shephard, N., Hayman, A., Whitehead, A., Walters, SJ., (2014), ´Sample size requirements to estimate key design parameters from external pilot randomised controlled trials: a simulation study´, Trials, 15(1), pp.264.

34. Rabe-Hesketh, S., Skrondal, A., (2012), Multilevel and Longitudinal Modeling Using Stata, Third Edition, College Station, Texas: Stata Press.

For peer review only - http://bmjopen.bmj.com/site/about/guidelines.xhtml

35. Kahan BC, Jairath V, Dore CJ, Morris TP., (2014), ´The risks and rewards of covariate adjustment in randomized trials: an assessment of 12 outcomes from 8 studies´, Trials, 15, pp.139.

36. White, IR., Horton, NJ., Carpenter, J., Pocock, SJ., (2011), ´Strategy for intention to treat analysis in randomised trials with missing outcome data`, BMJ, 342:d40.

37. Medicines and Healthcare products Regulatory Agency (2014) Medical device stand-alone software including apps. Available at: https://www.gov.uk/government/publications/medical-devices-software-applications-apps/medical-device-stand-alone-software-including-apps (Accessed: 13 May 2015)

For peer review only - http://bmjopen.bmj.com/site/about/guidelines.xhtml

Barts and The London
School of Medicine and Dentistry

APPENDICES

Appendix 1 – Information with regards to Safety Reporting in Non-CTIMP Research

	Who	When	How	To Whom
**SUSAR**	Chief Investigator	Report to the Sponsor, and QA manager within 24 hours<br>MREC within 15 days of learning of the event	SAE Report form for Non-CTIMPs, available from NRES website.	Sponsor and MREC
**Urgent Safety Measures**	Chief Investigator	Contact the Sponsor and MREC Immediately<br><br>Within 3 days	By phone<br><br>Substantial amendment form giving notice in writing setting out the reasons for the urgent safety measures and the plan for future action.	Main REC and Sponsor<br><br>Main REC with a copy also sent to the sponsor. The MREC will acknowledge this within 30 days of receipt.
**Progress Reports**	Chief Investigator	Annually (starting 12 months after the date of favourable opinion)	Annual Progress Report Form (non-CTIMPs) available from the NRES website	Main REC and Sponsor
**Declaration of the conclusion or early termination of the study**	Chief Investigator	Within 90 days (conclusion)<br><br>Within 15 days (early termination)<br><br>*The end of study should be defined in the protocol*	End of Study Declaration form available from the NRES website	Main REC with a copy to be sent to the sponsor
**Summary of final Report**	Chief Investigator	Within one year of conclusion of the Research	Where the study has met its objectives, the main findings and arrangements for publication or dissemination including feedback to participants	Main REC with a copy to be sent to the sponsor

For peer review only - http://bmjopen.bmj.com/site/about/guidelines.xhtml

Barts and The London School of Medicine and Dentistry

PCTU

MEMPHIS

MEMPHIS

Statistical Analysis Plan

Version: 3.0
Date: 26/Jan/2017

Person(s) contributing to the analysis plan	
Name(s) and position(s)	Neil Wright (Statistician)<br>Brennan Kahan (Statistician)<br>Elizabeth Ball (CI)<br>Gordon Forbes (Statistician)
**Authorisation**	
Position	**Chief or principal investigator**
Name	Elizabeth Ball
Signature	
Date	26/01/2017
Position	**Senior trial statistician**
Name	Brennan Kahan
Signature	
Date	26/01/2017
Position	**Independent statistician**
Name	Gian Luca Di Tanna
Tick once reviewed	✓
Date	30/01/2017

For peer review only - http://bmjopen.bmj.com/site/about/guidelines.xhtml

Barts and The London School of Medicine and Dentistry

PCTU

1. INTRODUCTION 3

2. STUDY METHODS 6

3. STUDY OUTCOMES 8

4. DATA COLLECTION 10

5. DERIVED VARIABLES 14

6. STATISTICAL ANALYSIS 15

7. GRAPHS AND FIGURES TO BE PRODUCED 19

8. REFERENCES 21

9. APPENDIX A: DERIVED AND COMPUTED VARIABLES 23

10. APPENDIX B: STATA CODE FOR GENERATING ADHERENCE OUTCOMES 29

11. APPENDIX C: STATA CODE FOR STATISTICAL ANALYSIS OF CLINICAL OUTCOMES 32

12. APPENDIX D: DRAFT TABLES 33

13. APPENDIX E: DATA / FILE MANAGEMENT 53

For peer review only - http://bmjopen.bmj.com/site/about/guidelines.xhtml

1. INTRODUCTION

1.1. Purpose of statistical analysis plan

The purpose of this document is to provide details of the statistical analyses and presentation of results to be reported within the principal paper(s) of the MEMPHIS trial. Any exploratory, post hoc or unplanned analyses will be clearly identified in the respective study analysis report. This document does not detail the qualitative analysis, and so aims and outcomes that are collected for qualitative analyses only are not included.

This document has been developed prior to examination of trial data and will not be implemented prior to final approval. Statisticians will be blinded to individual treatment allocations until this statistical analysis plan has been approved, all trial data has been collected and the trial is complete.

This document is based on protocol version 8.0 (December 2016)

1.2. Members of the writing committee

Neil Wright (Statistician) was primarily responsible for writing the Statistical Analysis Plan, with input from Brennan Kahan (Senior Statistician). Neil Wright was responsible for writing the computer code to implement the analysis strategy. Elizabeth Ball (CI) and Julie Dodds also contributed to this Statistical Analysis Plan.

1.3. Summary

Short Title	MEMPHIS
Methodology	A randomised feasibility trial
Research Sites	This trial will be conducted at the Royal London and Whipps Cross Hospitals
Objectives/Aims	The overall aim is to assess the feasibility of implementing a trial of a mindfulness meditation intervention delivered by a mobile phone app for patients with chronic pelvic pain (CPP). The primary objectives are:<br><br>To provide feasibility data for a large multicentre RCT aimed at rigorously testing mindfulness meditation in CPP<br><br>To determine whether this app can be seamlessly integrated into clinical practice, especially CPP pathways
Number of Participants/Patients	90 women with CPP will be recruited and each randomised into one of the three trial groups (meditation app, progressive muscle relaxation or no app).

Main Inclusion Criteria	To be eligible for the MEMPHIS study, the women must:<br><br>Be age 18 or over<br><br>Have either organic or non-organic chronic pelvic pain lasting for 6 months or more<br><br>Have access to a personal computer or smartphone.<br><br>Understand simple spoken English
Statistical Methodology and Analysis	Feasibility outcomes will be summarised using descriptive statistics. Clinical outcomes will be analysed using linear mixed-effects models, and results will be presented as a difference in means and a 95% confidence interval.

1.4. Changes from planned analysis in the protocol

- In the protocol, the dropout rate is a feasibility outcome but is not defined. In this analysis plan, we define two feasibility outcomes as "the number and proportion of participants who never return or answer a follow-up questionnaire at 6 months post-randomisation" and "the number and proportion of participants who do not return a follow-up questionnaire, but do answer the questionnaire by phone at 6 month post-randomisation".

- In the protocol, duration of recruitment is described as "the number of days from the beginning to the end of recruitment". In this analysis plan, duration of recruitment is defined as "the number of days from the day recruitment opens until the day the 90th patient is randomised (inclusive of both end days)".

- In the protocol, "Sexual Health Outcomes score (as measured by Sexual Health Outcomes in Women Questionnaire (SHOW-Q))" is given as a clinical outcome. In this analysis plan, this is replaced by the SHOW-Q global score, for sexually active participants, and by the SHOW-Q pelvic interference score, for all participants.

1.5. Changes from SAP v1.0

- In section 1.4 of version 1.0 of the SAP we stated *"In the protocol, "Quality of life score (as measured by the RAND Short form (36) Health Survey (SF-36))" is given as a clinical outcome. In this analysis plan, this is replaced by four of the RAND SF-36 subscales: physical functioning, general health, social functioning, and pain."* This has now been removed from the SAP as the protocol has been updated to reflect the change in the way quality of life score is being measured.

- The definition of app use has been changed from "having completed at least 50% of a session" to "having completed at least 90% of a session" (section 3.1). The change was

For peer review only - http://bmjopen.bmj.com/site/about/guidelines.xhtml

Barts and The London School of Medicine and Dentistry

PCTU

made due to Headspace, the data provider of the app usage data, only collecting data on sessions which were at least 90% complete.

1.6. Changes from SAP v2.0

- Added clarification to section 4.3 that data collected outside the recommended window for follow-up will still be included in analysis.
- In section 6.5.1, specified that the number of CRFs returned within the follow-up windows specified in section 4.3 will be summarised.
- Corrected scoring of CPAQ in Appendix A.
- Amended scoring of MYMOP in Appendix A so item scores are missing if the symptoms or activities are entered differently at follow up time points.

For peer review only - http://bmjopen.bmj.com/site/about/guidelines.xhtml

Barts and The London School of Medicine and Dentistry

PCTU

2. STUDY METHODS

2.1. Study objectives

The overall aim is to assess the feasibility of implementing a trial of a mindfulness meditation intervention delivered by a mobile phone app for patients with chronic pelvic pain (CPP). The primary objectives are:

- To provide feasibility data for a large multicentre RCT aimed at rigorously testing Mindfulness meditation in patients with CPP.
- To determine whether this app can be seamlessly integrated into clinical practice, especially CPP pathways.

2.2. Overall study design and plan

MEMPHIS is a randomised feasibility trial. Eligible women will be randomised to one of the three treatment groups:

- Intervention: 60 days of the app delivering mindfulness meditation content (in addition to usual care).
- Active control: 60 days of the app delivering progressive muscle relaxation content (in addition to usual care).
- Treatment as usual: Usual care

2.3. Selection of study population

2.3.1. Inclusion Criteria

To be eligible for the MEMPHIS study, the women must meet the following criteria:

- Aged 18 or over
- Women with organic and non-organic chronic pelvic pain lasting for six months or more
- Be capable of understanding the information provided, with use of an interpreter if required and being able to understand simple English as is used in the app
- Give written informed consent

2.3.2. Exclusion Criteria

Patients who meet the following criteria are ineligible to participate:

- No access to a Personal computer or smartphone

For peer review only - http://bmjopen.bmj.com/site/about/guidelines.xhtml

Barts and The London School of Medicine and Dentistry

PCTU

2.4. Method of treatment assignment and randomisation

After informed consent, patients will be randomised using a central, web-based system in a 1:1:1 ratio to one of the three treatment groups, using permuted blocks (of sizes 27, 30, 33) without stratification.

2.5. Sample size determination

30 participants will be recruited to each of the three treatment groups, giving a total of 90 participants. As this is a feasibility study, we have not performed a sample size calculation based upon the power to detect a significant treatment effect on a clinical outcome. However, 90 participants should provide a reliable estimate for the standard deviation of the primary clinical outcome (likely to be pain acceptance) [1, 2], which can be used to inform the sample size calculation of the main trial.

For peer review only - http://bmjopen.bmj.com/site/about/guidelines.xhtml

3. STUDY OUTCOMES

3.1. Feasibility outcomes

- Duration of recruitment (measured from the day recruitment opens until the day the 90th patient is randomised)
- Estimates to be used for the sample size calculation of the phase III RCT:
  - The estimated SD at 60 days, 3 months, and 6 months post-randomisation for pain acceptance (as measured by the Chronic Pain Acceptance Questionnaire (CPAQ-8))
  - The number and proportion of participants who never return or answer a follow-up questionnaire at 6 months post-randomisation.
  - The number and proportion of participants who do not return a follow-up questionnaire, but do answer the questionnaire by phone at 6 month post-randomisation.
- Patient adherence to app use measured by the following outcomes:
  - Number of days (within the first 60 days from randomisation) a patient has used the app (with app use defined as having completed at least 90%% of a session).
  - Whether the patient has used the app on 22 or more days within the first 60 days from randomisation.
  - Number of weeks (within the first eight weeks from randomisation) a patient has used the app on three or more days.
  - Whether the patient has used the app on three or more days in 6 or more weeks (within the first eight weeks from randomisation).
  - Whether the patient has used the app on 22 or more days within the first 60 days from randomisation, AND used the app on three or more days in 6 or more weeks within the first eight weeks from randomisation.

3.2. App satisfaction questionnaires

At 60 days post-randomisation:

- System Usability Scale (SUS) score (0 [worst] – 100 [best])
- Reponses to the purpose made app satisfaction questionnaire

For peer review only - http://bmjopen.bmj.com/site/about/guidelines.xhtml

Barts and The London School of Medicine and Dentistry PCTU

3.3. Clinical outcomes

The following clinical outcomes at 60 days, 3 months, and 6 months post-randomisation:

- Pain acceptance score (as measured by the Chronic Pain Acceptance Questionnaire (CPAQ-8)) (0 [worst] – 48 [best])
- RAND Short form (36) Health Survey (RAND SF-36) scales:
    - Physical functioning (0 [worst] – 100 [best])
    - Pain (0 [worst] – 100 [best])
    - General health (0 [worst] – 100 [best])
    - Social functioning (0 [worst] – 100 [best])
- Depression score (as measured by the Hospital Anxiety and Depression Scale (HADS)) (0 [best] – 21 [worst])
- Anxiety score (as measured by HADS) (0 [best] – 21 [worst])
- Mindfulness score (as measure by the Cognitive and Mindfulness - Revised (CAMS – R) scale) (12 [worst] – 48 [best])
- Pain related disability score (as measured by the Chronic Pain Grade (CPG) disability subscale) (0 [best] – 100 [worst])
- Self efficacy score (as measured by the Pain Self-Efficacy Questionnaire (PSEQ)) (0 [worst] – 60 [best])
- Sexual Health Outcomes scores (as measured by the Sexual Health Outcomes in Women Questionnaire (SHOW-Q)):
    - SHOW-Q global score, for sexually active participants (0 [worst] – 100 [best])
    - SHOW-Q pelvic interference score, for all participants (0 [best] – 100 [worst])
- Subjective outcome score (as measured by the Measure Yourself Medical Outcome Profile (MYMOP)) (0 [best] – 6 [worst])

The following qualitative outcomes are not included in the Statistical Analysis Plan:

- Reasons for patient non-adherence to app use
- Obstacles to recruitment from participants and recruiting staff
- Usability/integration etc
- Determining primary/secondary outcomes of interest
- App satisfaction questionnaires for service providers

For peer review only - http://bmjopen.bmj.com/site/about/guidelines.xhtml

Barts and The London School of Medicine and Dentistry PCTU

4. DATA COLLECTION

This section describes the variables that will be collected during the trial to be used in the analysis described by this plan.

4.1. Collected at baseline only

The following variables will be collected for each participant at baseline only.

Demographic:

- Age
- Weight
- Height
- Living arrangements (Alone, With others)
- Employment status (Employed (full or part time, including self-employment), Unemployed and looking for work, At school or in full time education, Unable to work due to long term sickness, Looking after your home/family, Retired from paid work, Other)
- Age left full time education (I did not receive a formal education, Age 12 or less, Age 13 to 16, Age 17 to 19, Age 20 or over, I am still in full time education, Other)
- Ethnic group (White, Black, Central Asian, Middle Eastern, Southern Asian, Mixed, Other ethnic group, Do not wish to say)
- Do you smoke (Yes, No)
- Number of cigarettes per week
- Do you drink alcohol (Yes, No)
- Number of alcohol units per week

Prior and concurrent treatment:

- Treatment used in last six months: Acupuncture; Gabapentin; Amitriptyline; Biofeedback; Botox injection; Contraceptive pills/patch/ring; Exercise, yoga or pilates; Injections to suppress ovaries (e.g. Prostap, Zoladex); Herbal Medicine; Meditation or relaxation exercises; Massage; Nutrition/diet; Codeine or Morphine type painkillers; Nerve blocks; Over the counter medication; Physiotherapy; Psychological (talking) therapy; Transcutaneous Electrical Nerve Stimulation (TENS); Surgery; Other. (One variable for each: Yes, No.)
- Currently using pain treatment (Yes, No)

Participants' pain:

For peer review only - http://bmjopen.bmj.com/site/about/guidelines.xhtml

- Length of pain (0-6 months, 7-12 months, 1-2 years, 3-5 years, 6-10 years, More than 10 years)
- Pain over the past week (0 [No pain] to 10 [Pain as bad as could be])

4.2. Randomisation details

The following variables for each participant will be held in the randomisation database.

- Date of randomisation
- Treatment group allocation

4.3. Collected at baseline and follow up

The following clinical outcome variables will be collected for each participant at baseline, 60 days, 3 months, and 6 months post-randomisation. We aim to collect 60 day follow up data between 46 and 74 days from randomisation, 3 month follow up date between 76 and 104 days and 6 month follow up data between 159 and 201 days. However, data collected outside these day ranges will be included in the analysis.

- Pain acceptance (as measured Chronic Pain Acceptance Questionnaire (CPAQ-8)) (4 variables)
- Short form (36) Health Survey (SF-36) (36 variables)
- Depression (as measured by the Hospital Anxiety and Depression Scale (HADS)) (7 variables)
- Anxiety (as measured by HADS) (7 variables)
- Mindfulness (as measure by the Cognitive and Mindfulness - Revised (CAMS – R) scale) (12 variables)
- Pain related disability (as measured by the Chronic Pain Grade (CPG) disability subscale) (3 variables)
- Self efficacy (as measured by the Pain Self-Efficacy Questionnaire (PSEQ)) (10 variables)
- Sexual Health Outcomes (as measured by Sexual Health Outcomes in Women Questionnaire (SHOW-Q)) (12 variables)
- Subjective outcome (as measured by Measure Yourself Medical Outcome Profile (MYMOP)) (4 variables)

For peer review only - http://bmjopen.bmj.com/site/about/guidelines.xhtml

Barts and The London School of Medicine and Dentistry PCTU

Date of visit / date completed and method of collection (return of postal questionnaire or via telephone) for each follow-up questionnaire will also be collected. When the follow-up questionnaire is answered via telephone, the variables for the Short form (36) Health Survey (SF-36), Self efficacy (as measured by the Pain Self-Efficacy Questionnaire (PSEQ)), and Sexual Health Outcomes (as measured by Sexual Health Outcomes in Women Questionnaire (SHOW-Q)) are not collected.

4.4. App usage data

App usage data will be received from Headspace, for all participants randomised to the Intervention or Active Control arms. The data will include variables for participant login token, duration of session, filename of session, date and time of completion. Each observation represents one user completing (at least 90% of) a mindfulness meditation or muscle relaxation session.

4.5. App satisfaction questionnaires

The following variables will be collected for participants randomised to an app arm, at 60 days post-randomisation:

- System Usability Scale (SUS) (10 variables)
- Purpose made questionnaire responses:
    - Nine statements with categorical response. (Totally disagree, Somewhat disagree, Neither agree nor disagree, Somewhat agree, Totally agree) (9 variables)
    - One question (Did you use the app every day? (Yes, No))

4.6. Unintentional unblinding of randomised treatment

After the participant has been randomised, the following variables will be collected from the researcher:

- Was the participant randomised to the app treatment arm? (Yes, No)
- If the participant was allocated to the app treatment arm, which app treatment do you believe the participant was randomised to? (Intervention app, Control app, Don't know)

At 6 months (between 159 and 201 days) post-randomisation, the following variables will be collected from the participant:

- Did you use the smartphone app for MEMPHIS? (Yes, No)

For peer review only - http://bmjopen.bmj.com/site/about/guidelines.xhtml

- Do you think you received the new treatment or comparison treatment? (New Treatment, Comparison Treatment, Don't Know)

For peer review only - http://bmjopen.bmj.com/site/about/guidelines.xhtml

5. DERIVED VARIABLES

5.1. Feasibility outcomes

A participant is counted as never having returned follow-up questionnaire at 6 months post-randomisation if date of visit / date completed and all other fields in the follow-up questionnaire are missing.

The patient adherence to app use outcomes listed in Section 3.1 will be calculated from the app usage data described in Section 4.4. Completing a session that is at least ten minutes on a day counts as having used the app on that day. Sample Stata code showing the calculation of these outcome variables is given in APPENDIX B: STATA CODE FOR GENERATING ADHERENCE OUTCOMES.

In the app usage data, date and timestamps will be provided in Coordinated Universal Time (UTC). These will be converted to UK time (BST/GMT as appropriate) before outcomes are derived.

5.2. Clinical outcomes

Details for how the clinical outcome scores list in Section 3.3 are derived from question responses (Section 4.2) are given in APPENDIX A: DERIVED AND COMPUTED VARIABLES.

5.3. System Usability Score (SUS) score

Details for how the System Usability Scale (SUS) score is derived from question responses is given in APPENDIX A: DERIVED AND COMPUTED VARIABLES.

For peer review only - http://bmjopen.bmj.com/site/about/guidelines.xhtml

6. STATISTICAL ANALYSIS

6.1. Analysis populations

All analyses will be carried out according to the intention-to-treat (ITT) principle: all patients with a non-missing outcome will be analysed according to the group to which they are randomised.

Summaries of patient adherence to app use will include all participants randomised to the intervention or active control treatment groups.

Sample means and SDs for clinical outcomes will include all participants with a non-missing outcome at that time point.

Analyses to estimate treatment effects for clinical outcomes (Section 6.4.2) will include all patients with a non-missing outcome for at least one of the three follow-up time points (60 days, 3 months, or 6 months) [3]. Patients with a missing outcome at all follow-up time points for a clinical outcome are excluded from the analysis of that clinical outcome. A clinical outcome is non-missing if there are recorded responses at that time point for all individual questions required for the derivation of the clinical outcome. (Note that for the Subjective outcome score (MYMOP profile score), only symptom 1 score and wellbeing score are required.)

6.2. Baseline variables

Demographic, prior and concurrent treatment, and participants' pain baseline variables are listed in Section 4.1. Each variable (plus body mass index instead of height and weight) will be summarised for each treatment group by the mean (SD) or median (IQR) for continuous variables, and the number (%) for categorical variables. Draft tables are given in APPENDIX D: DRAFT TABLES.

6.3. Analysis of feasibility outcomes

Duration of recruitment will be stated. It is the number of days from the day recruitment opens until the day the 90th patient is randomised (inclusive of both end days).

The number of participants randomised in each one month period from the day recruitment opens will be presented.

The estimated SD in each treatment group at each follow-up time point for pain acceptance (as measured by the Chronic Pain Acceptance Questionnaire (CPAQ-8)) will be presented.

Each patient adherence to app use outcome listed in Section 3.1 will be summarised separately for the intervention and active control treatment groups. Each outcome will be presented as the mean (SD) or median (IQR) for continuous variables, and the number (%) for categorical variables. Draft tables are given in APPENDIX D: DRAFT TABLES.

For peer review only - http://bmjopen.bmj.com/site/about/guidelines.xhtml

Barts and The London School of Medicine and Dentistry PCTU

6.4. Analysis of clinical outcomes

6.4.1. Descriptive statistics

For each clinical outcome listed in Section 3.3 we will present:

- The number of patients in each treatment group with a non-missing outcome at each time point.
- The mean (SD) in each treatment group at each time point.

6.4.2. Statistical analysis

For each clinical outcome we will present estimated treatment effects for each follow-up time point, with a 95% confidence interval. Estimates of treatment effects will be presented comparing the intervention group (mindfulness meditation app) to the control (treatment as usual) group, the intervention group to the active control (progressive muscle relaxation app) group, and the active control group to the control group.

Outcomes will be analysed using linear mixed-effects models with outcome measurement (at three follow-up time points) as the dependant variable. The model will include fixed time effects, a fixed effect for treatment, time treatment interactions for 3 months and 6 months follow-up time points, and an unstructured correlation matrix for the residuals [4]. The model will include baseline measure of the outcome as a covariate, assuming a linear relationship between baseline and outcome [5]. The model will be fitted using restricted maximum likelihood. Example Stata code for this analysis model is given in APPENDIX C: STATA CODE FOR STATISTICAL ANALYSIS OF CLINICAL OUTCOMES.

If there are missing values for baseline measure of a clinical outcome, they will be replaced by the mean of the observed baseline values for all participants in all treatment arms (mean imputation) [6]. Missing values of clinical outcomes at follow-up will not be imputed.

If the mixed effects models fail to converge, treatment effects will be estimated using separate linear regression models for each follow-up time point. Baseline measure of the outcome will be included as a covariate.

6.5. Other analyses

6.5.1. Comparison of losses to follow-up

The number and proportion of patients in each treatment group who have returned, answered by phone, or never returned the follow-up questionnaire will be presented for each follow-up time point (60 days, 3 months, and 6 months post-randomisation). A patient is counted as having returned data unless date of visit / date completed and all other fields in the follow-up questionnaire are missing. A draft table is given in APPENDIX D: DRAFT

For peer review only - http://bmjopen.bmj.com/site/about/guidelines.xhtml

TABLES.Summaries of the following baseline variables will be presented separately for patients who have returned, answered by phone, or never returned the follow-up questionnaire at the 6 month time point:

- Age at randomisation
- Body mass index
- Living arrangements
- Employment status
- Age left full time education
- Ethnic group
- Do you smoke
- Number of cigarettes per week
- Do you drink alcohol
- Number of units of alcohol per week
- Length of pain
- Pain over the past week
- Baseline values of clinical outcomes:
    - Pain acceptance score
    - Depression score
    - Anxiety score
    - Pain related disability score

6.5.2. Unintentional unblinding of randomised treatment

For each participants in the intervention and active control arm, researcher response to the question “If the participant was allocated to the app treatment arm, which app treatment do you believe the participant was randomised to?” will be summarised by number and percentage.

For participants in the intervention and active control arms, response to the question “Do you think you received the new treatment or comparison treatment?” will be summarised by number and percentage. A draft table is given in APPENDIX D: DRAFT TABLES.

For peer review only - http://bmjopen.bmj.com/site/about/guidelines.xhtml

Barts and The London School of Medicine and Dentistry PCTU

6.5.3. Summarising missing data in clinical outcomes

For each clinical outcome variable we will present the number and proportion of individuals for whom the outcome is complete for at least one of the three follow-up time points (60 days, 3 months, or 6 months).

For each clinical outcome variable, we will also present the number and proportion of individuals for whom the outcome is not completed (either because the questionnaire was not returned, or because the participant left all variables for that outcome blank), partially completed (one or more, but not all, variables used in its derivation are missing), or complete (no variables used in its derivation are missing) at each time point.

Completely missing and partially missing outcomes will be summarised separately according to whether follow-up was completed via the mail-in questionnaire or over the phone.

6.5.1. Summarising data returned outside of target follow up periods

The number and proportion of patients in each treatment group who had follow up questionnaires completed within the time periods specified in section 4.3 will be presented for each follow up point. These are between 46 and 74 days for 60 days follow up, between 76 and 104 days for 3 month follow up, and between 159 and 201 days for 6 month follow up.

6.5.2. App usability

The mean (SD) of the System Usability Scale (SUS) score will be presented separately for the treatment app and active control app arms.

The number and proportion of each response for each question in the purpose made app satisfaction questionnaire will be presented separated for the treatment app and active control app arms. The number and proportion responding "Yes" to the question "Did you use the app every day?" will also be presented for each app arm.

6.5.3. Serious adverse events

We will present the number of reported serious adverse events in each treatment arm.

6.6. Analysis software

The analysis will be carried out using Stata.

For peer review only - http://bmjopen.bmj.com/site/about/guidelines.xhtml

7. GRAPHS AND FIGURES TO BE PRODUCED

7.1. Participant flow

Participant throughput will be summarized in a CONSORT diagram:

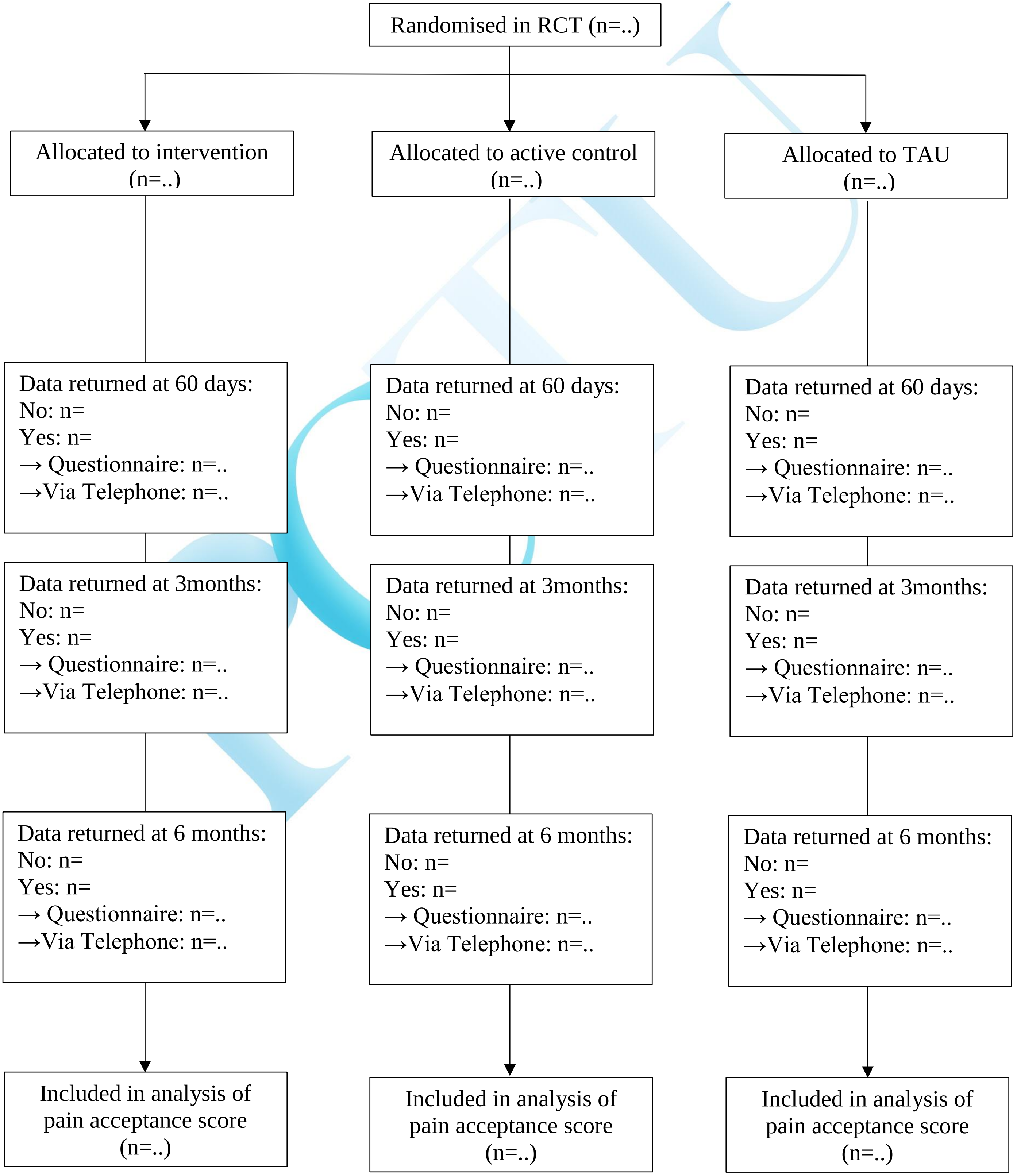

For peer review only - http://bmjopen.bmj.com/site/about/guidelines.xhtml

Barts and The London
School of Medicine and Dentistry

PCTU

7.2. Graphs

The following graphs will be created:

- Line graph showing mean CPAQ score at each time point for each treatment group. The graph will also include lines showing 95% confidence intervals for each mean CPAQ score.
- Line graph showing all estimated treatment effects (and 95% confidence intervals) on CPAQ score for each follow-up time point. (Estimates of treatment effects will be presented comparing the intervention group (mindfulness meditation app) to the control (treatment as usual) group, the intervention group to the active control (progressive muscle relaxation app) group, and the active control group to the control group.)
- Stacked bar chart showing the proportion of participants in each treatment group who have returned the follow-up questionnaire or answered the follow-up questionnaire by phone at each follow-up time point (60 days, 3 months, and 6 months post-randomisation).

For peer review only - http://bmjopen.bmj.com/site/about/guidelines.xhtml

Barts and The London School of Medicine and Dentistry PCTU

8. REFERENCES

[1] G. Lancaster, S. Dodd and P. Williamson, “Design and analysis of pilot studies: recommendations for good practice,” *Journal of Evaluation in Clinical Practice,* vol. 10, no. 2, pp. 307-12, 2004.

[2] M. Teare, M. Dimairo, N. Shephard, A. Hayman, A. Whitehead and S. Walters, “Sample size requirements to estimate key design parameters from external pilot randomised controlled trials: a simulation study,” *Trials*, vol. 15, p. 264, 2004.

[3] I. R. White, N. J. Horton, J. Carpenter and S. J. Pocock, “Strategy for intention to treat analysis in randomised trials with missing outcome data.,” *BMJ (Clinical research ed.),* vol. 342, p. d40, 2011.

[4] S. Rabe-Hesketh and A. Skrondal, Multilevel and Longitudinal Modeling Using Stata. Volume I: Continuous Responses, Stata Press, 2012.

[5] B. C. Kahan, V. Jairath, C. J. Doré and T. P. Morris, “The risks and rewards of covariate adjustment in randomized trials: an assessment of 12 outcomes from 8 studies,” *Trials,* vol. 15, p. 139, 2014.

[6] I. White and S. Thompson, “Adjusting for partially missing baseline measurements in randomized trials.,” *Statistics in Medicine,* vol. 24, no. 7, pp. 993-1007, 2005.

[7] “RAND 36-Item Short Form Survey (SF-36) Scoring Instructions,” [Online]. Available: https://www.rand.org/health/surveys_tools/mos/36-item-short-form/scoring.html.

[8] A. S. Zigmond and R. Snaith, “The hospital anxiety and depression scale.,” *Acta psychiatrica Scandinavica,* no. 6, pp. 361-370, 1983.

[9] G. Feldman, A. Hayes, S. Kumar, J. Greeson and J.-P. Laurenceau, “Mindfulness and Emotion Regulation: The Development and Initial Validation of the Cognitive and Affective Mindfulness Scale-Revised (CAMS-R),” *Journal of Psychopathology and Behavioral Assessment,* vol. 29, p. 177–190, 2007.

[10] M. V. Korff, J. Ormel, F. J. Keefe and S. F. Dworkin, “Grading the severity of chronic pain,” *Pain,* vol. 50, no. 2, pp. 133-149, 1992.

[11] M. K. Nicholas, “The pain self-efficacy questionnaire: Taking pain into account,” *European Journal of Pain,* vol. 11, p. 153–163, 2007.

[12] R. A. Fish, B. McGuire, M. Hogan, T. G. Morrison and I. Stewart, “Validation of the Chronic Pain Acceptance Questionnaire (CPAQ) in an Internet,” *Pain,* vol. 149, p. 435–443, 2010.

For peer review only - http://bmjopen.bmj.com/site/about/guidelines.xhtml

[13] [Online]. Available: http://www.bris.ac.uk/primaryhealthcare/resources/mymop/general-information/.

[14] [Online]. Available: https://www.usability.gov/how-to-and-tools/methods/system-usability-scale.html.

For peer review only - http://bmjopen.bmj.com/site/about/guidelines.xhtml

9. APPENDIX A: DERIVED AND COMPUTED VARIABLES

Unless otherwise stated, if an individual response variable used in the derivation of an outcome is missing then the outcome variable is missing.

Variables names used in the example code correspond to the field names specified in the trial database "Requirements Specification Document".

Body mass index

BMI is calculated as a person's weight (measured in kilograms) divided by the square of their height (measured in metres).

```
generate BMI = WEIGHT / ((HEIGHT / 100)^2)
```

RAND Short form (36) Health Survey (SF-36) scales scores [7]

Responses to individual questions are recoded as shown in the first table below. Each scale score is the average score for the questions in that scale, as shown in the second table below.

Item numbers	Original response code	Recode to
GH1, GH2, GH6, GH8, GH11b, GH11d	1	100
	2	75
	3	50
	4	25
	5	0
GH3a, GH3b, GH3c, GH3d, GH3e, GH3f, GH3g, GH3h, GH3i, GH3j	1	0
	2	50
	3	100
GH10, GH11a, GH11c	1	0
	2	25
	3	50
	4	75
	5	100
GH7	1	100
	2	80
	3	60
	4	40
	5	20
	6	0

Scale	After recoding, average the following items

Physical functioning	GH3a, GH3b, GH3c, GH3d, GH3e, GH3f, GH3g, GH3h, GH3i, GH3j
Pain	GH7, GH8
General health	GH1, GH11a, GH11b, GH11c, GH11d
Social functioning	GH6, GH10

```
recode GH1 GH2 GH6 GH8 GH11b GH11d (1=100) (2=75) (3=50) (4=25)
(5=0)
recode GH3a GH3b GH3c GH3d GH3e GH3f Gh3g GH3h GH3i Gh3j (1=0)
(2=50) (3=100)
recode GH10 GH11a GH11c (1=0) (2=25) (3=50) (4=75) (5=100)
recode GH7 (1=100) (2=80) (3=60) (4=40) (5=20) (6=0)
generate SF36_PHYSICALFUNC = (GH3a + GH3b + GH3c + GH3d + GH3e
+ GH3f + GH3g + GH3h + GH3i + GH3j) / 10
generate SF36_SOCIALFUNC = (GH6 + GH10) / 2
generate SF36_PAIN = (GH7 + GH8) / 2
generate SF36_GENERALHEALTH = (GH1 + GH11a + GH11b + GH11c +
GH11d) / 5
```

Depression score (as measured by the Hospital Anxiety and Depression Scale (HADS)) [7]

After appropriate recoding, the HADS depression score is the sum of scores for questions 2, 4, 6, 8, 10, 12 and 14.

```
recode HADS02 HADS04 HADS12 HADS14 (1=0) (2=1) (3=2) (4=3)
recode HADS06 HADS08 HADS10 (1=3) (2=2) (3=1) (4=0)
generate HADS_DEPRESSION = HADS02 + HADS04 +HADS06 + HADS08 +
HADS10 + HADS12 + HADS14
```

Anxiety score (as measured by HADS) [7]

After appropriate recoding, the HADS anxiety score is the sum of scores for questions 1, 3, 5, 7, 9, 11 and 13.

```
recode HADS01 HADS03 HADS05 HADS11 HADS13 (1=3) (2=2) (3=1)
(4=0)
recode HADS07 HADS09 (1=0) (2=1) (3=2) (4=3)
generate HADS_ANXIETY = HADS01 + HADS03 + HADS05 + HADS07 +
HADS09 + HADS11 + HADS13
```

Mindfulness score (as measure by the Cognitive and Mindfulness - Revised (CAMS – R) scale) [8]

For peer review only - http://bmjopen.bmj.com/site/about/guidelines.xhtml

After appropriate recording, the CAMS-R mindfulness score is the sum of scores for all questions 1 to 12.

```
recode CAMSR02 CAMSR06 CAMSR07 (1=4) (2=3) (3=2) (4=1)
generate CAMSR_SCORE = CAMSR01 + CAMSR02 + CAMSR03 + CAMSR04 +
CAMSR05 + CAMSR06 + CAMSR07 + CAMSR08 + CAMSR09 + CAMSR10 +
CAMSR11 + CAMSR12
```

Pain related disability score (as measured by the Chronic Pain Grade (CPG) disability subscale) [9]

THE CPG pain related disability score is the mean of the daily activities, social activities, and work activities scores, multiplied by 10.

```
generate CPG_DISABILITYSCORE = [(CPGd1 + CPGd2 + CPGd3) / 3 ] *
10
```

Self efficacy score (as measured by the Pain Self-Efficacy Questionnaire (PSEQ)) [10]

The PSEQ self efficacy score is the sum of scores for all questions 1 to 10.

```
generate PSEQ_SCORE = PSEQ01 + PSEQ02 + PSEQ03 + PSEQ04 + PSEQ05
+ PSEQ06 + PSEQ07 + PSEQ08 + PSEQ09 + PSEQ10
```

Pain acceptance score (as measured Chronic Pain Acceptance Questionnaire (CPAQ-8)) [12]

After reverse scoring, the CPAQ-8 pain willingness score is the sum of scores from questions 4, 5, 7 and 8. The CPAQ-8 activity engagement score is the sum of scores from questions 1, 2, 3, 5 and 6. The CPAQ-8 total score is the sum of the pain willingness score and the activity engagement score.

```
recode CPAQ CPAQ4 CPAQ5 CPAQ7 CPAQ8 (0=6) (1=5) (2=4) (3=3)
(4=2) (5=1) (6=0)
generate CPAQ_PAINWILL = CPAQ4 + CPAQ5 + CPAQ7 + CPAQ8
generate CPAQ_ACTIVITYENG = CPAQ1 + CPAQ2 + CPAQ3 + CPAQ6
generate CPAQ_TOTAL = CPAQ_PAINWILL + CPAQ_ACTIVITYENG
```

Sexual Health Outcomes score (as measured by Sexual Health Outcomes in Women

For peer review only - http://bmjopen.bmj.com/site/about/guidelines.xhtml

Questionnaire (SHOW-Q))

Each response is rescaled to a score 0 to 100, with higher scores reflecting higher sexual functioning or fewer sexual problems. For a 5 response item, the scores are 0, 25, 50, 75 or 100. For a 4 response item, the scores are 0, 33.3, 66.7 or 100. The scoring for each question is shown in the table below.

If a participant answers “I don’t have a partner” or “I don’t have sex without a partner” to question 2 or “I did not have sexual activity” to any of questions 3, 4, 6, 7 or 9, then the participant is classed as sexually inactive. Otherwise, the participant is classed as sexually active.

For sexually active participants, the SHOW-Q global score is calculated as the mean of all rescaled scores. Higher scores reflect higher sexual functioning or fewer sexual problems.

For all participants, the SHOW-Q pelvic problem interference score is the mean of response scores to questions 10, 11 and 12 after they are reverse scored. Higher scores reflect more interference.

**Item number**	**Response text**	**Original response code**	**Recode to**
SHOWQ01, SHOWQ02	Very satisfied	1	100
	Somewhat satisfied	2	75
	Neither satisfied nor dissatisfied	3	50
	Somewhat dissatisfied	4	25
	Very dissatisfied	5	0
SHOWQ10, SHOWQ11, SHOWQ12	Not at all	1	100
	Slightly	2	75
	Moderately	3	50
	Quite a bit	4	25
	Extremely	5	0
SHOWQ03, SHOWQ04	Never	1	0
	Rarely	2	25
	Sometimes	3	50
	Most of the time	4	75
	All of the time	5	100
SHOWQ08	Never	1	0
	Once or twice	2	25
	3-4 times	3	50
	5-6 times	4	75
	More than 6 times	5	100
SHOWQ05	Did not experience any orgasms	1	0
	Mild	2	33.3
	Moderate	3	66.7
	Strong	4	100
SHOWQ06,	Not a problem	1	100

For peer review only - http://bmjopen.bmj.com/site/about/guidelines.xhtml

SHOWQ07, SHOWQ09	Little of a problem	2	66.7
	Somewhat of a problem	3	33.3
	Very much of a problem	4	0

```
generate SHOWQ_ACTIVE = 1
replace SHOWQ_ACTIVE = 0 if SHOWQ02==6 | SHOWQ02==7 | SHOWQ03==6
| SHOW04==6 | SHOWQ06==5 | SHOWQ07==5 | SHOWQ09== 5
recode SHOWQ01 SHOWQ02 SHOW10 SHOWQ11 SHOWQ12 (1=100) (2=75)
(3=50) (4=25) (5=0)
recode SHOWQ03 SHOWQ04 SHOWQ08 (1=0) (2=25) (3=50) (4=75)
(5=100)
recode SHOWQ05 (1=0) (2=33.3) (3=66.7) (4=100)
recode SHOWQ06 SHOWQ07 SHOWQ09 (1=100) (2=66.7) (3=33.3) (4=0)
generate SHOWQ_GLOBAL = (SHOWQ01 + SHOWQ02 + SHOWQ03 + SHOWQ04
+ SHOWQ05 + SHOWQ06 + SHOWQ07 + SHOWQ08 + SHOWQ09 + SHOWQ10 +
SHOWQ11 + SHOWQ12)/12 if SHOWQ_ACTIVE == 1
generate SHOWQ_PELVPROBLEM = ((100 - SHOWQ10) + (100 - SHOWQ11)
+ (100 - SHOWQ12))/3
```

Subjective outcome score (as measured by Measure Yourself Medical Outcome Profile (MYMOP)) [12]

If the description for symptom 1, symptom 2, symptom 3 or activity does not match the description given for the corresponding symptom or activity at baseline then the score for that symptom or activity is missing.

If symptom 1 score or wellbeing score are missing, then MYMOP profile score is missing.The MYMOP profile score is the mean of the symptom 1 score, symptom 2 score, activity score, wellbeing score, and symptom 3 score. (Symptom 2 score, activity score and symptom 3 score are only included if they are not missing)

```
egen MYMOP_PROFILE = rowmean(SYMSCORE1, SYMSCORE2, ACTSCORE,
WELLBEING, SYMSCORE3)
```

System Usability Scale (SUS) score [13]

Barts and The London School of Medicine and Dentistry PCTU

For questions 1, 3, 5, 7, and 9 the score contribution is the response number minus 1. For questions 2, 4, 6, 8, and 10 the score contribution is 5 minus the response number. The SUS score is the sum of all score contributions multiplied by 2.5

```
recode SUS01 SUS03 SYS05 SUS07 SUS09 (1 = 0) (2 = 1) (3 = 2) (4
= 3) (5 = 4)
recode SUS02 SUS04 SUS06 SUS08 SUS10 (1 = 4) (2 = 3) (3 = 2) (4
= 1) (5 = 0)
generate SUS_SCORE  = 2.5 * (SUS01 + SUS02 + SUS03 + SUS04 +
SUS05 +SUS06 + SUS07 + SUS08 + SUS9 + SUS10)
```

Adherence outcomes

countin60days	Number of days (within the first 60 days from randomisation) a patient has used the app (with app use defined as having completed at least 90% of a session).	
numberofweeksthreeplus	Number of weeks (within the first eight weeks from randomisation) a patient has used the app on three or more days.	
adhere_countin60days	Whether the patient has used the app on 22 or more days within the first 60 days from randomisation.	1 = Yes<br>0 = No
adhere_numberofweeksthreeplus	Whether the patient has used the app on three or more days in 6 or more weeks (within the first eight weeks from randomisation).	1 = Yes<br>0 = No

Sample Stata code showing the calculation of these outcome variables is given in APPENDIX B: STATA CODE FOR GENERATING ADHERENCE OUTCOMES.

For peer review only - http://bmjopen.bmj.com/site/about/guidelines.xhtml

10. APPENDIX B: STATA CODE FOR GENERATING ADHERENCE OUTCOMES

Sample of Stata code for generating adherence outcomes from app usage data supplied by Headspace:

```
gen date_completed = date(datecompleted, "DMY")

format date_completed %td


gen date_rand = date(dateofrandomisation, "DMY")

format date_rand %td


gen date_fromrand = date_completed-date_rand


***


* Drop sessions which are not part of intervention (i.e. short duration)

drop if duration<5


* Remove multiple sessions in same day

duplicates report id date_fromrand
```

For peer review only - http://bmjopen.bmj.com/site/about/guidelines.xhtml

```
duplicates drop id date_fromrand , force


gen in60days = 1 if date_fromrand<61

bysort id: egen countin60days = count(in60days)


gen numberofweeksthreeplus = 0


forvalues week=1/8 {

     gen inweek`week' = 1 if date_fromrand>7*(`week'-1) & date_fromrand<7*`week'+1

     gen threeplusinweek`week' = 0

     bysort id: egen countinweek`week' = count(inweek`week')

     assert countinweek`week'<8

     bysort id: replace threeplusinweek`week' = 1 if countinweek`week'>2

     bysort id: replace numberofweeksthreeplus = numberofweeksthreeplus +1 if countinweek`week'>2

}
```

For peer review only - http://bmjopen.bmj.com/site/about/guidelines.xhtml

```
bysort id: keep if _n==1

keep id countin60days numberofweeksthreeplus threeplusinweek* countinweek*


gen adhere_countin60days = 0

replace adhere_countin60days = 1 if countin60days>21

gen adhere_numberofweeksthreeplus = 0

replace adhere_numberofweeksthreeplus = 1 if numberofweeksthreeplus>5


tab adhere_countin60days adhere_numberofweeksthreeplus
```

For peer review only - http://bmjopen.bmj.com/site/about/guidelines.xhtml

11. APPENDIX C: STATA CODE FOR STATISTICAL ANALYSIS OF CLINICAL OUTCOMES

The following Stata shows the model that will be used to estimate treatment effects on clinical outcomes:

```
xtmixed outcome time##treat baseline || id: , noconstant
residuals(unstructured, t(time)) var reml
```

Estimates of treatment effects for each treatment arm comparison and time point will then be obtained using:

```
lincom 1.treat + 1.time#1.treat

lincom 1.treat + 2.time#1.treat

lincom 1.treat + 3.time#1.treat

lincom 2.treat + 1.time#2.treat

lincom 2.treat + 2.time#2.treat

lincom 2.treat + 3.time#2.treat

lincom 2.treat + 1.time#2.treat - 1.treat + 1.time#1.treat

lincom 2.treat + 2.time#2.treat - 1.treat + 2.time#1.treat

lincom 2.treat + 3.time#2.treat - 1.treat + 3.time#1.treat
```

For peer review only - http://bmjopen.bmj.com/site/about/guidelines.xhtml

Barts and The London School of Medicine and Dentistry PCTU

12. APPENDIX D: DRAFT TABLES

12.1.1. Baseline demographics and medical history

Figures are mean (SD) unless stated otherwise.

	Intervention (n=…)	Active control (n=…)	Usual care (n=…)
**Demographics**			
Age at randomisation (Years)	XX (XX)	XX (XX)	XX (XX)
Body mass index	XX (XX)	XX (XX)	XX (XX)
Living arrangements – no. (%)			
Alone	XX (XX)	XX (XX)	XX (XX)
With others	XX (XX)	XX (XX)	XX (XX)
Employment status – no. (%)			
Employed	XX (XX)	XX (XX)	XX (XX)
Unemployed and looking for work	XX (XX)	XX (XX)	XX (XX)
At school or in full time education	XX (XX)	XX (XX)	XX (XX)
Unable to work due to long term sickness	XX (XX)	XX (XX)	XX (XX)
Look after your home/family	XX (XX)	XX (XX)	XX (XX)
Retired from paid work	XX (XX)	XX (XX)	XX (XX)
Other	XX (XX)	XX (XX)	XX (XX)
Age left full time education – no. (%)			
I did not receive a formal education	XX (XX)	XX (XX)	XX (XX)
Age 12 or less	XX (XX)	XX (XX)	XX (XX)
Age 13 to 16	XX (XX)	XX (XX)	XX (XX)
Age 17 to 19	XX (XX)	XX (XX)	XX (XX)
Age 20 or over	XX (XX)	XX (XX)	XX (XX)
I am still in full time education	XX (XX)	XX (XX)	XX (XX)
Other	XX (XX)	XX (XX)	XX (XX)
Ethnic group – no. (%)			
White	XX (XX)	XX (XX)	XX (XX)
Black	XX (XX)	XX (XX)	XX (XX)
Central Asian	XX (XX)	XX (XX)	XX (XX)
Middle Eastern	XX (XX)	XX (XX)	XX (XX)
Southern Asian	XX (XX)	XX (XX)	XX (XX)
Mixed	XX (XX)	XX (XX)	XX (XX)
Other ethnic group	XX (XX)	XX (XX)	XX (XX)
Do not wish to say	XX (XX)	XX (XX)	XX (XX)
Smoker – no. (%)			
Yes	XX (XX)	XX (XX)	XX (XX)
No	XX (XX)	XX (XX)	XX (XX)
If yes, number of cigarettes per week	XX (XX)	XX (XX)	XX (XX)
Drink alcohol – no. (%)			

For peer review only - http://bmjopen.bmj.com/site/about/guidelines.xhtml

Yes	XX	(XX)	XX	(XX)	XX	(XX)
No	XX	(XX)	XX	(XX)	XX	(XX)
If yes, number of units of alcohol per week	XX	(XX)	XX	(XX)	XX	(XX)
**Baseline medical history**						
Length of pain – no. (%)						
0-6 months	XX	(XX)	XX	(XX)	XX	(XX)
7-12 months	XX	(XX)	XX	(XX)	XX	(XX)
1-2 years	XX	(XX)	XX	(XX)	XX	(XX)
3-5 years	XX	(XX)	XX	(XX)	XX	(XX)
6-10 years	XX	(XX)	XX	(XX)	XX	(XX)
More than 10 years	XX	(XX)	XX	(XX)	XX	(XX)
Pain over the past week	XX	(XX)	XX	(XX)	XX	(XX)

12.1.2. Prior and concurrent treatment

Figures are number (percentage).

	Intervention (n=…)		Active control (n=…)		Usual care (n=…)	
Treatment used in last six months						
Acupuncture	XX	(XX)	XX	(XX)	XX	(XX)
Gabapentin	XX	(XX)	XX	(XX)	XX	(XX)
Amitriptyline	XX	(XX)	XX	(XX)	XX	(XX)
Biofeedback	XX	(XX)	XX	(XX)	XX	(XX)
Botox injection	XX	(XX)	XX	(XX)	XX	(XX)
Contraceptive pills/patch/ring	XX	(XX)	XX	(XX)	XX	(XX)
Exercise, yoga or pilates	XX	(XX)	XX	(XX)	XX	(XX)
Injections to suppress ovaries (e.g. Prostap, Zoladex)	XX	(XX)	XX	(XX)	XX	(XX)
Herbal Medicine	XX	(XX)	XX	(XX)	XX	(XX)
Meditation or relaxation exercises	XX	(XX)	XX	(XX)	XX	(XX)
Massage	XX	(XX)	XX	(XX)	XX	(XX)
Nutrition/diet	XX	(XX)	XX	(XX)	XX	(XX)
Codeine or Morphine type painkillers	XX	(XX)	XX	(XX)	XX	(XX)
Nerve blocks	XX	(XX)	XX	(XX)	XX	(XX)
Over the counter medication	XX	(XX)	XX	(XX)	XX	(XX)
Physiotherapy	XX	(XX)	XX	(XX)	XX	(XX)
Psychological (talking) therapy	XX	(XX)	XX	(XX)	XX	(XX)
Transcutaneous Electrical Nerve Stimulation (TENS)	XX	(XX)	XX	(XX)	XX	(XX)
Surgery	XX	(XX)	XX	(XX)	XX	(XX)

For peer review only - http://bmjopen.bmj.com/site/about/guidelines.xhtml

	Intervention	Active control	Usual care
Other	XX (XX)	XX (XX)	XX (XX)
Currently using pain treatment			
Yes	XX (XX)	XX (XX)	XX (XX)
No	XX (XX)	XX (XX)	XX (XX)

12.1.3. Baseline values of clinical outcomes

Figures are mean (SD) unless stated otherwise.

	Intervention (n=…)	Active control (n=…)	Usual care (n=…)
SF-36 scales:			
Physical functioning	XX (XX)	XX (XX)	XX (XX)
Pain	XX (XX)	XX (XX)	XX (XX)
General Health	XX (XX)	XX (XX)	XX (XX)
Social Functioning	XX (XX)	XX (XX)	XX (XX)
Depression score	XX (XX)	XX (XX)	XX (XX)
Anxiety score	XX (XX)	XX (XX)	XX (XX)
Mindfulness score	XX (XX)	XX (XX)	XX (XX)
Pain related disability score	XX (XX)	XX (XX)	XX (XX)
Self efficacy score	XX (XX)	XX (XX)	XX (XX)
Pain acceptance score	XX (XX)	XX (XX)	XX (XX)
Sexual health outcomes:			
SHOW-Q global score	XX (XX)	XX (XX)	XX (XX)
SHOW-Q pelvic problem interference score	XX (XX)	XX (XX)	XX (XX)
Subjective outcome score	XX (XX)	XX (XX)	XX (XX)

12.1.4. Loss to follow-up

	Intervention (n=…)	Active control (n=…)	Usual care (n=…)
Follow-up questionnaire returned – no. (%)			
60 days	XX (XX)	XX (XX)	XX (XX)
3 months	XX (XX)	XX (XX)	XX (XX)
6 months	XX (XX)	XX (XX)	XX (XX)
Follow-up questionnaire answered by phone – no. (%)			
60 days	XX (XX)	XX (XX)	XX (XX)
3 months	XX (XX)	XX (XX)	XX (XX)
6 months	XX (XX)	XX (XX)	XX (XX)
Follow-up questionnaire never returned – no. (%)			
60 days	XX (XX)	XX (XX)	XX (XX)
3 months	XX (XX)	XX (XX)	XX (XX)
6 months	XX (XX)	XX (XX)	XX (XX)

For peer review only - http://bmjopen.bmj.com/site/about/guidelines.xhtml

Barts and The London School of Medicine and Dentistry

PCTU

12.1.5. Loss to follow-up

Figures are mean (SD) unless stated otherwise.

	6 months follow-up questionnaire returned (n=…)	6 months follow-up questionnaire answered by phone (n=…)	6 months follow-up questionnaire never returned (n=…)
**Demographics**			
Age at randomisation (Years)	XX (XX)	XX (XX)	XX (XX)
Body mass index	XX (XX)	XX (XX)	XX (XX)
Living arrangements – no. (%)			
Alone	XX (XX)	XX (XX)	XX (XX)
With others	XX (XX)	XX (XX)	XX (XX)
Employment status – no. (%)			
Employed	XX (XX)	XX (XX)	XX (XX)
Unemployed and looking for work	XX (XX)	XX (XX)	XX (XX)
At school or in full time education	XX (XX)	XX (XX)	XX (XX)
Unable to work due to long term sickness	XX (XX)	XX (XX)	XX (XX)
Look after your home/family	XX (XX)	XX (XX)	XX (XX)
Retired from paid work	XX (XX)	XX (XX)	XX (XX)
Other	XX (XX)	XX (XX)	XX (XX)
Age left full time education – no. (%)			
I did not receive a formal education	XX (XX)	XX (XX)	XX (XX)
Age 12 or less	XX (XX)	XX (XX)	XX (XX)
Age 13 to 16	XX (XX)	XX (XX)	XX (XX)
Age 17 to 19	XX (XX)	XX (XX)	XX (XX)
Age 20 or over	XX (XX)	XX (XX)	XX (XX)
I am still in full time education	XX (XX)	XX (XX)	XX (XX)
Other	XX (XX)	XX (XX)	XX (XX)
Ethnic group – no. (%)			
White	XX (XX)	XX (XX)	XX (XX)
Black	XX (XX)	XX (XX)	XX (XX)
Central Asian	XX (XX)	XX (XX)	XX (XX)
Middle Eastern	XX (XX)	XX (XX)	XX (XX)
Southern Asian	XX (XX)	XX (XX)	XX (XX)
Mixed	XX (XX)	XX (XX)	XX (XX)
Other ethnic group	XX (XX)	XX (XX)	XX (XX)
Do not wish to say	XX (XX)	XX (XX)	XX (XX)
Smoker – no. (%)			

For peer review only - http://bmjopen.bmj.com/site/about/guidelines.xhtml

	Intervention	Active control	Usual care
Yes	XX (XX)	XX (XX)	XX (XX)
No	XX (XX)	XX (XX)	XX (XX)
If yes, number of cigarettes per week	XX (XX)	XX (XX)	XX (XX)
Drink alcohol – no. (%)			
Yes	XX (XX)	XX (XX)	XX (XX)
No	XX (XX)	XX (XX)	XX (XX)
If yes, number of units of alcohol per week	XX (XX)	XX (XX)	XX (XX)
**Baseline medical history**			
Length of pain – no. (%)			
0-6 months	XX (XX)	XX (XX)	XX (XX)
7-12 months	XX (XX)	XX (XX)	XX (XX)
1-2 years	XX (XX)	XX (XX)	XX (XX)
3-5 years	XX (XX)	XX (XX)	XX (XX)
6-10 years	XX (XX)	XX (XX)	XX (XX)
More than 10 years	XX (XX)	XX (XX)	XX (XX)
Pain over the past week	XX (XX)	XX (XX)	XX (XX)
**Baseline values of clinical outcomes**			
Pain acceptance score (CPAQ-8)	XX (XX)	XX (XX)	XX (XX)
Depression score (HADS)	XX (XX)	XX (XX)	XX (XX)
Anxiety score (HADS)	XX (XX)	XX (XX)	XX (XX)
Pain related disability score (CPG)	XX (XX)	XX (XX)	XX (XX)

12.1.6. Follow up within target follow up period

	Intervention (n=…)	Active control (n=…)	Usual care (n=…)
Follow-up questionnaire returned or answered by phone within target follow up period– no. (%)			
60 days (46 and 74days)	XX (XX)	XX (XX)	XX (XX)
3 months (76 and 104 days)	XX (XX)	XX (XX)	XX (XX)
6 months (159 and 201 days)	XX (XX)	XX (XX)	XX (XX)

12.1.7. Adherence to app use

Figures are mean (SD) unless stated otherwise.

	Intervention (n=…)	Active control (n=…)
Number of days (within the first 60 days from randomisation) a patient has used the app	XX (XX)	XX (XX)

For peer review only - http://bmjopen.bmj.com/site/about/guidelines.xhtml

Number of weeks (within the first eight weeks from randomisation) a patient has used the app on three or more days	XX (XX)	XX (XX)
Used the app on 22 or more days within the first 60 days from randomisation – no. (%)	XX (XX)	XX (XX)
Used the app on three or more days in 6 or more weeks (within the first eight weeks from randomisation) – no. (%)	XX (XX)	XX (XX)
Used the app on 22 or more days within the first 60 days from randomisation, AND used the app on three or more days in 6 or more weeks within the first eight weeks from randomisation – no. (%)	XX (XX)	XX (XX)

12.1.8. App usability questionnaire

Figures are number (percentage).

	Totally disagree	Somewhat disagree	Neither agree nor disagree	Somewhat agree	Totally agree	Not answered
It is easy to access the app whenever I wanted to use it						
Intervention:	XX (XX)	XX (XX)	XX (XX)	XX (XX)	XX (XX)	XX (XX)
Active control:	XX (XX)	XX (XX)	XX (XX)	XX (XX)	XX (XX)	XX (XX)
After being shown, I understood how the app would work						
Intervention:	XX (XX)	XX (XX)	XX (XX)	XX (XX)	XX (XX)	XX (XX)
Active control:	XX (XX)	XX (XX)	XX (XX)	XX (XX)	XX (XX)	XX (XX)
It was fun to work with the app						
Intervention:	XX (XX)	XX (XX)	XX (XX)	XX (XX)	XX (XX)	XX (XX)
Active control:	XX (XX)	XX (XX)	XX (XX)	XX (XX)	XX (XX)	XX (XX)
The app worked well						
Intervention:	XX (XX)	XX (XX)	XX (XX)	XX (XX)	XX (XX)	XX (XX)
Active control:	XX (XX)	XX (XX)	XX (XX)	XX (XX)	XX (XX)	XX (XX)
It was easy to work through the modules						
Intervention:	XX (XX)	XX (XX)	XX (XX)	XX (XX)	XX (XX)	XX (XX)
Active control:	XX (XX)	XX (XX)	XX (XX)	XX (XX)	XX (XX)	XX (XX)

For peer review only - http://bmjopen.bmj.com/site/about/guidelines.xhtml

The number of modules was annoying						
Intervention:	XX (XX)	XX (XX)	XX (XX)	XX (XX)	XX (XX)	XX (XX)
Active control:	XX (XX)	XX (XX)	XX (XX)	XX (XX)	XX (XX)	XX (XX)
The modules were well-displayed on my smartphone						
Intervention:	XX (XX)	XX (XX)	XX (XX)	XX (XX)	XX (XX)	XX (XX)
Active control:	XX (XX)	XX (XX)	XX (XX)	XX (XX)	XX (XX)	XX (XX)
Using the app was difficult because of my daily activities						
Intervention:	XX (XX)	XX (XX)	XX (XX)	XX (XX)	XX (XX)	XX (XX)
Active control:	XX (XX)	XX (XX)	XX (XX)	XX (XX)	XX (XX)	XX (XX)
Using the app took too long						
Intervention:	XX (XX)	XX (XX)	XX (XX)	XX (XX)	XX (XX)	XX (XX)
Active control:	XX (XX)	XX (XX)	XX (XX)	XX (XX)	XX (XX)	XX (XX)

For peer review only - http://bmjopen.bmj.com/site/about/guidelines.xhtml

Barts and The London School of Medicine and Dentistry

PCTU

12.1.9. Clinical outcomes

	Intervention (n=…)				Active control (n=…)				Usual care (n=…)			
	n	(%)	Mean	(SD)	n	(%)	Mean	(SD)	n	(%)	Mean	(SD)
**Pain acceptance score**												
Baseline	XX	(XX)	XX	(XX)	XX	(XX)	XX	(XX)	XX	(XX)	XX	(XX)
60 days	XX	(XX)	XX	(XX)	XX	(XX)	XX	(XX)	XX	(XX)	XX	(XX)
3 months	XX	(XX)	XX	(XX)	XX	(XX)	XX	(XX)	XX	(XX)	XX	(XX)
6 months	XX	(XX)	XX	(XX)	XX	(XX)	XX	(XX)	XX	(XX)	XX	(XX)
Included in analysis †	XX	(XX)			XX	(XX)			XX	(XX)		
**Depression score**												
Baseline	XX	(XX)	XX	(XX)	XX	(XX)	XX	(XX)	XX	(XX)	XX	(XX)
60 days	XX	(XX)	XX	(XX)	XX	(XX)	XX	(XX)	XX	(XX)	XX	(XX)
3 months	XX	(XX)	XX	(XX)	XX	(XX)	XX	(XX)	XX	(XX)	XX	(XX)
6 months	XX	(XX)	XX	(XX)	XX	(XX)	XX	(XX)	XX	(XX)	XX	(XX)
Included in analysis †	XX	(XX)			XX	(XX)			XX	(XX)		
**Anxiety score**												
Baseline	XX	(XX)	XX	(XX)	XX	(XX)	XX	(XX)	XX	(XX)	XX	(XX)
60 days	XX	(XX)	XX	(XX)	XX	(XX)	XX	(XX)	XX	(XX)	XX	(XX)
3 months	XX	(XX)	XX	(XX)	XX	(XX)	XX	(XX)	XX	(XX)	XX	(XX)
6 months	XX	(XX)	XX	(XX)	XX	(XX)	XX	(XX)	XX	(XX)	XX	(XX)
Included in analysis †	XX	(XX)			XX	(XX)			XX	(XX)		

For peer review only - http://bmjopen.bmj.com/site/about/guidelines.xhtml

Barts and The London School of Medicine and Dentistry PCTU

**Mindfulness score**												
Baseline	XX	(XX)	XX	(XX)	XX	(XX)	XX	(XX)	XX	(XX)	XX	(XX)
60 days	XX	(XX)	XX	(XX)	XX	(XX)	XX	(XX)	XX	(XX)	XX	(XX)
3 months	XX	(XX)	XX	(XX)	XX	(XX)	XX	(XX)	XX	(XX)	XX	(XX)
6 months	XX	(XX)	XX	(XX)	XX	(XX)	XX	(XX)	XX	(XX)	XX	(XX)
Included in analysis †	XX	(XX)			XX	(XX)			XX	(XX)		
**Pain related disability score**												
Baseline	XX	(XX)	XX	(XX)	XX	(XX)	XX	(XX)	XX	(XX)	XX	(XX)
60 days	XX	(XX)	XX	(XX)	XX	(XX)	XX	(XX)	XX	(XX)	XX	(XX)
3 months	XX	(XX)	XX	(XX)	XX	(XX)	XX	(XX)	XX	(XX)	XX	(XX)
6 months	XX	(XX)	XX	(XX)	XX	(XX)	XX	(XX)	XX	(XX)	XX	(XX)
Included in analysis †	XX	(XX)			XX	(XX)			XX	(XX)		
**Self efficacy score**												
Baseline	XX	(XX)	XX	(XX)	XX	(XX)	XX	(XX)	XX	(XX)	XX	(XX)
60 days	XX	(XX)	XX	(XX)	XX	(XX)	XX	(XX)	XX	(XX)	XX	(XX)
3 months	XX	(XX)	XX	(XX)	XX	(XX)	XX	(XX)	XX	(XX)	XX	(XX)
6 months	XX	(XX)	XX	(XX)	XX	(XX)	XX	(XX)	XX	(XX)	XX	(XX)
Included in analysis †	XX	(XX)			XX	(XX)			XX	(XX)		
**SHOW-Q global score, for sexually active participants**												
Baseline	XX	(XX)	XX	(XX)	XX	(XX)	XX	(XX)	XX	(XX)	XX	(XX)
60 days	XX	(XX)	XX	(XX)	XX	(XX)	XX	(XX)	XX	(XX)	XX	(XX)
3 months	XX	(XX)	XX	(XX)	XX	(XX)	XX	(XX)	XX	(XX)	XX	(XX)

For peer review only - http://bmjopen.bmj.com/site/about/guidelines.xhtml

6 months	XX	(XX)	XX	(XX)	XX	(XX)	XX	(XX)	XX	(XX)	XX	(XX)
Included in analysis †	XX	(XX)			XX	(XX)			XX	(XX)		
**SHOW-Q pelvic problem interference score, for all participants**												
Baseline	XX	(XX)	XX	(XX)	XX	(XX)	XX	(XX)	XX	(XX)	XX	(XX)
60 days	XX	(XX)	XX	(XX)	XX	(XX)	XX	(XX)	XX	(XX)	XX	(XX)
3 months	XX	(XX)	XX	(XX)	XX	(XX)	XX	(XX)	XX	(XX)	XX	(XX)
6 months	XX	(XX)	XX	(XX)	XX	(XX)	XX	(XX)	XX	(XX)	XX	(XX)
Included in analysis †	XX	(XX)			XX	(XX)			XX	(XX)		
**Subjective outcome score**												
Baseline	XX	(XX)	XX	(XX)	XX	(XX)	XX	(XX)	XX	(XX)	XX	(XX)
60 days	XX	(XX)	XX	(XX)	XX	(XX)	XX	(XX)	XX	(XX)	XX	(XX)
3 months	XX	(XX)	XX	(XX)	XX	(XX)	XX	(XX)	XX	(XX)	XX	(XX)
6 months	XX	(XX)	XX	(XX)	XX	(XX)	XX	(XX)	XX	(XX)	XX	(XX)
Included in analysis †	XX	(XX)			XX	(XX)			XX	(XX)		
**SF-36: Physical functioning**												
Baseline	XX	(XX)	XX	(XX)	XX	(XX)	XX	(XX)	XX	(XX)	XX	(XX)
60 days	XX	(XX)	XX	(XX)	XX	(XX)	XX	(XX)	XX	(XX)	XX	(XX)
3 months	XX	(XX)	XX	(XX)	XX	(XX)	XX	(XX)	XX	(XX)	XX	(XX)
6 months	XX	(XX)	XX	(XX)	XX	(XX)	XX	(XX)	XX	(XX)	XX	(XX)
Included in analysis †	XX	(XX)			XX	(XX)			XX	(XX)		
**SF-36: Pain**												
Baseline	XX	(XX)	XX	(XX)	XX	(XX)	XX	(XX)	XX	(XX)	XX	(XX)

PCTU only

For peer review only - http://bmjopen.bmj.com/site/about/guidelines.xhtml

60 days	XX	(XX)	XX	(XX)	XX	(XX)	XX	(XX)	XX	(XX)	XX	(XX)
3 months	XX	(XX)	XX	(XX)	XX	(XX)	XX	(XX)	XX	(XX)	XX	(XX)
6 months	XX	(XX)	XX	(XX)	XX	(XX)	XX	(XX)	XX	(XX)	XX	(XX)
Included in analysis †	XX	(XX)			XX	(XX)			XX	(XX)		
**SF-36: General Health**												
Baseline	XX	(XX)	XX	(XX)	XX	(XX)	XX	(XX)	XX	(XX)	XX	(XX)
60 days	XX	(XX)	XX	(XX)	XX	(XX)	XX	(XX)	XX	(XX)	XX	(XX)
3 months	XX	(XX)	XX	(XX)	XX	(XX)	XX	(XX)	XX	(XX)	XX	(XX)
6 months	XX	(XX)	XX	(XX)	XX	(XX)	XX	(XX)	XX	(XX)	XX	(XX)
Included in analysis †	XX	(XX)			XX	(XX)			XX	(XX)		
**SF-36: Social Functioning**												
Baseline	XX	(XX)	XX	(XX)	XX	(XX)	XX	(XX)	XX	(XX)	XX	(XX)
60 days	XX	(XX)	XX	(XX)	XX	(XX)	XX	(XX)	XX	(XX)	XX	(XX)
3 months	XX	(XX)	XX	(XX)	XX	(XX)	XX	(XX)	XX	(XX)	XX	(XX)
6 months	XX	(XX)	XX	(XX)	XX	(XX)	XX	(XX)	XX	(XX)	XX	(XX)
Included in analysis †	XX	(XX)			XX	(XX)			XX	(XX)		

(† Included in analysis if outcome is available for at least one follow-up time point.)

For peer review only - http://bmjopen.bmj.com/site/about/guidelines.xhtml

Barts and The London School of Medicine and Dentistry

PCTU

	Intervention vs. Active control Adjusted mean difference (95% CI)	Invention vs. Usual care Adjusted mean difference (95% CI)	Active control vs. Usual care Adjusted mean difference (95% CI)
**Pain acceptance score**			
60 days	XX (XX to XX)	XX (XX to XX)	XX (XX to XX)
3 months	XX (XX to XX)	XX (XX to XX)	XX (XX to XX)
6 months	XX (XX to XX)	XX (XX to XX)	XX (XX to XX)
**Depression score**			
60 days	XX (XX to XX)	XX (XX to XX)	XX (XX to XX)
3 months	XX (XX to XX)	XX (XX to XX)	XX (XX to XX)
6 months	XX (XX to XX)	XX (XX to XX)	XX (XX to XX)
**Anxiety score**			
60 days	XX (XX to XX)	XX (XX to XX)	XX (XX to XX)
3 months	XX (XX to XX)	XX (XX to XX)	XX (XX to XX)
6 months	XX (XX to XX)	XX (XX to XX)	XX (XX to XX)
**Mindfulness score**			
60 days	XX (XX to XX)	XX (XX to XX)	XX (XX to XX)
3 months	XX (XX to XX)	XX (XX to XX)	XX (XX to XX)
6 months	XX (XX to XX)	XX (XX to XX)	XX (XX to XX)
**Pain related disability score**			
60 days	XX (XX to XX)	XX (XX to XX)	XX (XX to XX)
3 months	XX (XX to XX)	XX (XX to XX)	XX (XX to XX)
6 months	XX (XX to XX)	XX (XX to XX)	XX (XX to XX)
**Self efficacy score**			
60 days	XX (XX to XX)	XX (XX to XX)	XX (XX to XX)
3 months	XX (XX to XX)	XX (XX to XX)	XX (XX to XX)
6 months	XX (XX to XX)	XX (XX to XX)	XX (XX to XX)
**SHOW-Q global score, for sexually active participants**			
60 days	XX (XX to XX)	XX (XX to XX)	XX (XX to XX)
3 months	XX (XX to XX)	XX (XX to XX)	XX (XX to XX)
6 months	XX (XX to XX)	XX (XX to XX)	XX (XX to XX)
**SHOW-Q pelvic problem interference score, for all participants**			
60 days	XX (XX to XX)	XX (XX to XX)	XX (XX to XX)
3 months	XX (XX to XX)	XX (XX to XX)	XX (XX to XX)

For peer review only - http://bmjopen.bmj.com/site/about/guidelines.xhtml

6 months	XX (XX to XX)	XX (XX to XX)	XX (XX to XX)
**Subjective outcome score**			
60 days	XX (XX to XX)	XX (XX to XX)	XX (XX to XX)
3 months	XX (XX to XX)	XX (XX to XX)	XX (XX to XX)
6 months	XX (XX to XX)	XX (XX to XX)	XX (XX to XX)
**SF-36: Physical Functioning**			
60 days	XX (XX to XX)	XX (XX to XX)	XX (XX to XX)
3 months	XX (XX to XX)	XX (XX to XX)	XX (XX to XX)
6 months	XX (XX to XX)	XX (XX to XX)	XX (XX to XX)
**SF-36: Pain**			
60 days	XX (XX to XX)	XX (XX to XX)	XX (XX to XX)
3 months	XX (XX to XX)	XX (XX to XX)	XX (XX to XX)
6 months	XX (XX to XX)	XX (XX to XX)	XX (XX to XX)
**SF-36: General Health**			
60 days	XX (XX to XX)	XX (XX to XX)	XX (XX to XX)
3 months	XX (XX to XX)	XX (XX to XX)	XX (XX to XX)
6 months	XX (XX to XX)	XX (XX to XX)	XX (XX to XX)
**SF-36: Social Functioning**			
60 days	XX (XX to XX)	XX (XX to XX)	XX (XX to XX)
3 months	XX (XX to XX)	XX (XX to XX)	XX (XX to XX)
6 months	XX (XX to XX)	XX (XX to XX)	XX (XX to XX)

12.1.10. Unintentional unblinding of randomised treatment

Figures are number (%) unless stated otherwise.

	**Intervention (n=…)**	**Active control (n=…)**
Researchers: Which app treatment do you believe the participant was randomised to?		
Intervention app	XX (XX)	XX (XX)
Control app	XX (XX)	XX (XX)
Don't know	XX (XX)	XX (XX)
Participants: Do you think you received the new treatment or comparison treatment?		
New treatment	XX (XX)	XX (XX)
Comparison treatment	XX (XX)	XX (XX)
Don't know	XX (XX)	XX (XX)

For peer review only - http://bmjopen.bmj.com/site/about/guidelines.xhtml

12.1.11. Partially missing clinical outcomes

	Not completed * n (%)		Partially Completed ** n (%)		Fully completed *** n (%)	
**Pain acceptance score**						
Baseline	XX	(XX)	XX	(XX)	XX	(XX)
60 days	XX	(XX)	XX	(XX)	XX	(XX)
3 months	XX	(XX)	XX	(XX)	XX	(XX)
6 months	XX	(XX)	XX	(XX)	XX	(XX)
**Depression score**						
Baseline	XX	(XX)	XX	(XX)	XX	(XX)
60 days	XX	(XX)	XX	(XX)	XX	(XX)
3 months	XX	(XX)	XX	(XX)	XX	(XX)
6 months	XX	(XX)	XX	(XX)	XX	(XX)
**Anxiety score**						
Baseline	XX	(XX)	XX	(XX)	XX	(XX)
60 days	XX	(XX)	XX	(XX)	XX	(XX)
3 months	XX	(XX)	XX	(XX)	XX	(XX)
6 months	XX	(XX)	XX	(XX)	XX	(XX)
**Mindfulness score**						
Baseline	XX	(XX)	XX	(XX)	XX	(XX)
60 days	XX	(XX)	XX	(XX)	XX	(XX)
3 months	XX	(XX)	XX	(XX)	XX	(XX)
6 months	XX	(XX)	XX	(XX)	XX	(XX)
**Pain related disability score**						
Baseline	XX	(XX)	XX	(XX)	XX	(XX)
60 days	XX	(XX)	XX	(XX)	XX	(XX)
3 months	XX	(XX)	XX	(XX)	XX	(XX)
6 months	XX	(XX)	XX	(XX)	XX	(XX)
**Self efficacy score**						
Baseline	XX	(XX)	XX	(XX)	XX	(XX)
60 days	XX	(XX)	XX	(XX)	XX	(XX)
3 months	XX	(XX)	XX	(XX)	XX	(XX)
6 months	XX	(XX)	XX	(XX)	XX	(XX)

For peer review only - http://bmjopen.bmj.com/site/about/guidelines.xhtml

**SHOW-Q global score, for sexually active participants**			
Baseline	XX (XX)	XX (XX)	XX (XX)
60 days	XX (XX)	XX (XX)	XX (XX)
3 months	XX (XX)	XX (XX)	XX (XX)
6 months	XX (XX)	XX (XX)	XX (XX)
**SHOW-Q pelvic problem interference score, for all participants**			
Baseline	XX (XX)	XX (XX)	XX (XX)
60 days	XX (XX)	XX (XX)	XX (XX)
3 months	XX (XX)	XX (XX)	XX (XX)
6 months	XX (XX)	XX (XX)	XX (XX)
**Subjective outcome score**			
Baseline	XX (XX)	XX (XX)	XX (XX)
60 days	XX (XX)	XX (XX)	XX (XX)
3 months	XX (XX)	XX (XX)	XX (XX)
6 months	XX (XX)	XX (XX)	XX (XX)
**SF-36: Physical Functioning**			
Baseline	XX (XX)	XX (XX)	XX (XX)
60 days	XX (XX)	XX (XX)	XX (XX)
3 months	XX (XX)	XX (XX)	XX (XX)
6 months	XX (XX)	XX (XX)	XX (XX)
**SF-36: Pain**			
Baseline	XX (XX)	XX (XX)	XX (XX)
60 days	XX (XX)	XX (XX)	XX (XX)
3 months	XX (XX)	XX (XX)	XX (XX)
6 months	XX (XX)	XX (XX)	XX (XX)
**SF-36: General Health**			
Baseline	XX (XX)	XX (XX)	XX (XX)
60 days	XX (XX)	XX (XX)	XX (XX)
3 months	XX (XX)	XX (XX)	XX (XX)
6 months	XX (XX)	XX (XX)	XX (XX)
**SF-36: Social Functioning**			
Baseline	XX (XX)	XX (XX)	XX (XX)
60 days	XX (XX)	XX (XX)	XX (XX)
3 months	XX (XX)	XX (XX)	XX (XX)
6 months	XX (XX)	XX (XX)	XX (XX)

For peer review only - http://bmjopen.bmj.com/site/about/guidelines.xhtml

Barts and The London School of Medicine and Dentistry

PCTU

* Questionnaire not answered or all variables used in the derivation of the outcome are missing.
** One or more, but not all, variables used in the derivation of the outcome are missing.
*** No variables used in the derivation of the outcome are missing.

For peer review only - http://bmjopen.bmj.com/site/about/guidelines.xhtml

Barts and The London School of Medicine and Dentistry

PCTU

	Questionnaire never returned		Questionnaire answered by telephone						Questionnaire returned					
			Not completed †		Partially completed ††		Fully completed †††		Not completed †		Partially completed ††		Fully completed †††	
	n	(%)	n	(%)	n	(%)	n	(%)	n	(%)	n	(%)	n	(%)
**Pain acceptance score**														
Baseline	XX	(XX)	n/a		n/a		n/a		XX	(XX)	XX	(XX)	XX	(XX)
60 days	XX	(XX)	XX	(XX)	XX	(XX)	XX	(XX)	XX	(XX)	XX	(XX)	XX	(XX)
3 months	XX	(XX)	XX	(XX)	XX	(XX)	XX	(XX)	XX	(XX)	XX	(XX)	XX	(XX)
6 months	XX	(XX)	XX	(XX)	XX	(XX)	XX	(XX)	XX	(XX)	XX	(XX)	XX	(XX)
**Depression score**														
Baseline	XX	(XX)	n/a		n/a		n/a		XX	(XX)	XX	(XX)	XX	(XX)
60 days	XX	(XX)	XX	(XX)	XX	(XX)	XX	(XX)	XX	(XX)	XX	(XX)	XX	(XX)
3 months	XX	(XX)	XX	(XX)	XX	(XX)	XX	(XX)	XX	(XX)	XX	(XX)	XX	(XX)
6 months	XX	(XX)	XX	(XX)	XX	(XX)	XX	(XX)	XX	(XX)	XX	(XX)	XX	(XX)
**Anxiety score**														
Baseline	XX	(XX)	n/a		n/a		n/a		XX	(XX)	XX	(XX)	XX	(XX)
60 days	XX	(XX)	XX	(XX)	XX	(XX)	XX	(XX)	XX	(XX)	XX	(XX)	XX	(XX)
3 months	XX	(XX)	XX	(XX)	XX	(XX)	XX	(XX)	XX	(XX)	XX	(XX)	XX	(XX)
6 months	XX	(XX)	XX	(XX)	XX	(XX)	XX	(XX)	XX	(XX)	XX	(XX)	XX	(XX)
**Mindfulness score**														
Baseline	XX	(XX)	n/a		n/a		n/a		XX	(XX)	XX	(XX)	XX	(XX)

PCTU only

Barts and The London School of Medicine and Dentistry PCTU

60 days	XX	(XX)	XX	(XX)	XX	(XX)	XX	(XX)	XX	(XX)	XX	(XX)	XX	(XX)
3 months	XX	(XX)	XX	(XX)	XX	(XX)	XX	(XX)	XX	(XX)	XX	(XX)	XX	(XX)
6 months	XX	(XX)	XX	(XX)	XX	(XX)	XX	(XX)	XX	(XX)	XX	(XX)	XX	(XX)
**Pain related disability score**														
Baseline	XX	(XX)	n/a		n/a		n/a		XX	(XX)	XX	(XX)	XX	(XX)
60 days	XX	(XX)	XX	(XX)	XX	(XX)	XX	(XX)	XX	(XX)	XX	(XX)	XX	(XX)
3 months	XX	(XX)	XX	(XX)	XX	(XX)	XX	(XX)	XX	(XX)	XX	(XX)	XX	(XX)
6 months	XX	(XX)	XX	(XX)	XX	(XX)	XX	(XX)	XX	(XX)	XX	(XX)	XX	(XX)
**Self efficacy score**														
Baseline	XX	(XX)	n/a		n/a		n/a		XX	(XX)	XX	(XX)	XX	(XX)
60 days	XX	(XX)	n/a		n/a		n/a		XX	(XX)	XX	(XX)	XX	(XX)
3 months	XX	(XX)	n/a		n/a		n/a		XX	(XX)	XX	(XX)	XX	(XX)
6 months	XX	(XX)	n/a		n/a		n/a		XX	(XX)	XX	(XX)	XX	(XX)
**SHOW-Q global score, for sexually active participants**														
Baseline	XX	(XX)	n/a		n/a		n/a		XX	(XX)	XX	(XX)	XX	(XX)
60 days	XX	(XX)	n/a		n/a		n/a		XX	(XX)	XX	(XX)	XX	(XX)
3 months	XX	(XX)	n/a		n/a		n/a		XX	(XX)	XX	(XX)	XX	(XX)
6 months	XX	(XX)	n/a		n/a		n/a		XX	(XX)	XX	(XX)	XX	(XX)
**SHOW-Q pelvic problem interference score, for all participants**														
Baseline	XX	(XX)	n/a		n/a		n/a		XX	(XX)	XX	(XX)	XX	(XX)
60 days	XX	(XX)	n/a		n/a		n/a		XX	(XX)	XX	(XX)	XX	(XX)
3 months	XX	(XX)	n/a		n/a		n/a		XX	(XX)	XX	(XX)	XX	(XX)

For peer review only - http://bmjopen.bmj.com/site/about/guidelines.xhtml

6 months	XX (XX)	n/a	n/a	n/a	XX (XX)	XX (XX)	XX (XX)
**Subjective outcome score**							
Baseline	XX (XX)	n/a	n/a	n/a	XX (XX)	XX (XX)	XX (XX)
60 days	XX (XX)	XX (XX)	XX (XX)	XX (XX)	XX (XX)	XX (XX)	XX (XX)
3 months	XX (XX)	XX (XX)	XX (XX)	XX (XX)	XX (XX)	XX (XX)	XX (XX)
6 months	XX (XX)	XX (XX)	XX (XX)	XX (XX)	XX (XX)	XX (XX)	XX (XX)
**SF-36: Physical Functioning**							
Baseline	XX (XX)	n/a	n/a	n/a	XX (XX)	XX (XX)	XX (XX)
60 days	XX (XX)	n/a	n/a	n/a	XX (XX)	XX (XX)	XX (XX)
3 months	XX (XX)	n/a	n/a	n/a	XX (XX)	XX (XX)	XX (XX)
6 months	XX (XX)	n/a	n/a	n/a	XX (XX)	XX (XX)	XX (XX)
**SF-36: Pain**							
Baseline	XX (XX)	n/a	n/a	n/a	XX (XX)	XX (XX)	XX (XX)
60 days	XX (XX)	n/a	n/a	n/a	XX (XX)	XX (XX)	XX (XX)
3 months	XX (XX)	n/a	n/a	n/a	XX (XX)	XX (XX)	XX (XX)
6 months	XX (XX)	n/a	n/a	n/a	XX (XX)	XX (XX)	XX (XX)
**SF-36: General Health**							
Baseline	XX (XX)	n/a	n/a	n/a	XX (XX)	XX (XX)	XX (XX)
60 days	XX (XX)	n/a	n/a	n/a	XX (XX)	XX (XX)	XX (XX)
3 months	XX (XX)	n/a	n/a	n/a	XX (XX)	XX (XX)	XX (XX)
6 months	XX (XX)	n/a	n/a	n/a	XX (XX)	XX (XX)	XX (XX)
**SF-36: Social Functioning**							

For peer review only - http://bmjopen.bmj.com/site/about/guidelines.xhtml

Barts and The London School of Medicine and Dentistry
PCTU

Baseline	XX	(XX)	n/a	n/a	n/a	XX	(XX)	XX	(XX)	XX	(XX)
60 days	XX	(XX)	n/a	n/a	n/a	XX	(XX)	XX	(XX)	XX	(XX)
3 months	XX	(XX)	n/a	n/a	n/a	XX	(XX)	XX	(XX)	XX	(XX)
6 months	XX	(XX)	n/a	n/a	n/a	XX	(XX)	XX	(XX)	XX	(XX)

† Questionnaire answered, but all variables used in the derivation of the outcome are missing.
†† One or more, but not all, variables used in the derivation of the outcome are missing.
††† No variables used in the derivation of the outcome are missing.

For peer review only - http://bmjopen.bmj.com/site/about/guidelines.xhtml

13. APPENDIX E: DATA / FILE MANAGEMENT

13.1.1. Sources of data

Copies of CRFs are included in the Statistics Master File. Data is entered from these into a PCTU database. Extracts from the database are supplied by the data manager onto a secure environment.

App usage data will be received from Headspace.

13.1.2. Programming plan

The trial folder on secure environment will contain a folder for each analysis.

An analysis folder should contain the following folders (and their contents):

- analysis data (saved Stata data files for analysis)
- do files (Stata do files for data preparation and analysis)
- log files (Stata log files)
- output (any files output e.g. produced tables and graphs)
- raw data (data as extracted from database)
- temp (any temporary files needed during data preparation or analysis)

Folders containing do files should include a text directory explaining the role of each do file.

13.1.3. Data dictionary

Field names specified in the database "Requirements Specification Document" will be the variable names in the data files. Where a variable is collect on more than one occasion, suffixes will be added to variables names (e.g. "_BASELINE", "_60DAYS", "_3MONTHS", "_6MONTHS").

Details of derived variables are given in Section 5, APPENDIX A: DERIVED AND COMPUTED VARIABLES, and APPENDIX B: STATA CODE FOR GENERATING ADHERENCE OUTCOMES.

A complete data dictionary will be produced for the final analysis data set.

For peer review only - http://bmjopen.bmj.com/site/about/guidelines.xhtml

Barts and The London School of Medicine and Dentistry

PCTU

Appendix 3: Supplementary tables

TABLE OF CONTENTS

Table 1. Prior and concurrent treatment 3
Table 2. Baseline values of clinical outcomes 4
Table 3. Baseline demographics of woman by 6 month questionnaire completion 5

1. FEASIBILITY OUTCOMES 7

1.1. FOLLOW-UP 7
Table 4. Losses to follow up 7
Figure 1. Figure 1: Proportion of participants answering follow up questionnaire 8
Table 5. Follow-up questionnaire returned or answered by phone within target follow up period 9
1.2. STANDARD DEVIATION OF CPAQ 10
Table 6. Estimated standard deviation of CPAQ 10
1.4. BLINDING 11
Table 7. Unintentional unbinding of randomised treatment 11

2. APP SATISFACTION QUESTIONNAIRES 12

Table 8. System usability scale 12
Table 9. App usability Questionnaire 13

3. CLINICAL OUTCOMES 14

3.1. RANGES OF CLINICAL OUTCOMES 14
3.2. COMPLETENESS OF CLINICAL DATA 15
Table 10. Partially missing clinical outcomes 15
Table 11. Partially missing clinical outcomes by method of questionnaire delivery 17
3.3. RESULTS OF ANALYSIS OF CLINICAL OUTCOMES 20
Table 12. Descriptive statistics for clinical outcomes 20
Table 13. Estimated treatment effects for clinical outcomes 23

For peer review only - http://bmjopen.bmj.com/site/about/guidelines.xhtml

Barts and The London
School of Medicine and Dentistry

PCTU

Figure 2. Figure 3: Estimated treatment effects and 95% confidence intervals for CPAQ....25

For peer review only

For peer review only - http://bmjopen.bmj.com/site/about/guidelines.xhtml

Barts and The London School of Medicine and Dentistry PCTU

Table 1. Prior and concurrent treatment

Figures are number (percentage).

	Summary measure			Missing data		
	Intervention (N=31)	Active control (N=30)	Usual care (N=29)	Intervention - no. (%)	Active control - no. (%)	Usual care - no. (%)
**Treatment used in the last six months**						
Acupuncture	2 (10.5)	5 (25.0)	1 (6.3)	12 (38.7)	10 (33.3)	13 (44.8)
Massage	11 (50.0)	8 (40.0)	7 (41.2)	9 (29.0)	10 (33.3)	12 (41.4)
Gabapentin	5 (26.3)	1 (5.9)	1 (6.3)	12 (38.7)	13 (43.3)	13 (44.8)
Nutrition/diet	14 (63.6)	14 (63.6)	18 (78.3)	9 (29.0)	8 (26.7)	6 (20.7)
Amitriptyline	5 (27.8)	4 (20.0)	4 (22.2)	13 (41.9)	10 (33.3)	11 (37.9)
Codeine or Morphine type painkillers	13 (56.5)	13 (59.1)	19 (76.0)	8 (25.8)	8 (26.7)	4 (13.8)
Biofeedback	0 (0.0)	0 (0.0)	0 (0.0)	13 (41.9)	12 (40.0)	13 (44.8)
Nerve blocks	0 (0.0)	2 (11.1)	0 (0.0)	14 (45.2)	12 (40.0)	12 (41.4)
Botox injection	0 (0.0)	0 (0.0)	0 (0.0)	14 (45.2)	13 (43.3)	13 (44.8)
Over the counter medication	17 (73.9)	9 (47.4)	17 (77.3)	8 (25.8)	11 (36.7)	7 (24.1)
Contraceptive pills/patch/ring	15 (68.2)	7 (36.8)	11 (52.4)	9 (29.0)	11 (36.7)	8 (27.6)
Physiotherapy	5 (26.3)	4 (20.0)	1 (6.7)	12 (38.7)	10 (33.3)	14 (48.3)
Exercise, yoga or Pilates	13 (59.1)	12 (60.0)	15 (78.9)	9 (29.0)	10 (33.3)	10 (34.5)
Psychological (talking) therapy	3 (16.7)	2 (11.1)	2 (13.3)	13 (41.9)	12 (40.0)	14 (48.3)
Injections to suppress ovaries (e.g. Prostap, Zoladex)	6 (33.3)	5 (25.0)	8 (38.1)	13 (41.9)	10 (33.3)	8 (27.6)
Transcutaneous Electrical Nerve Stimulation (TENS)	0 (0.0)	2 (11.1)	3 (17.6)	13 (41.9)	12 (40.0)	12 (41.4)
Herbal Medicine	4 (21.1)	5 (26.3)	8 (44.4)	12 (38.7)	11 (36.7)	11 (37.9)
Surgery	3 (16.7)	4 (23.5)	6 (31.6)	13 (41.9)	13 (43.3)	10 (34.5)
Meditation or relaxation exercises	11 (47.8)	7 (38.9)	10 (52.6)	8 (25.8)	12 (40.0)	10 (34.5)
Other	3 (37.5)	3 (33.3)	4 (44.4)	23 (74.2)	21 (70.0)	20 (69.0)
Currently using pain treatment				4 (12.9)	3 (10.0)	2 (6.9)
Yes	21 (77.8)	18 (66.7)	20 (74.1)			
No	6 (22.2)	9 (33.3)	7 (25.9)			

For peer review only - http://bmjopen.bmj.com/site/about/guidelines.xhtml

Barts and The London School of Medicine and Dentistry

PCTU

Table 2. Baseline values of clinical outcomes

Figures are mean (SD)

	Summary measure			Missing data		
	**Intervention (N=31)**	**Active control (N=30)**	**Usual care (N=29)**	**Intervention - no. (%)**	**Active control - no. (%)**	**Usual care - no. (%)**
CPAQ pain acceptance score	21.9 (9.5)	22.7 (8.4)	23.8 (8.5)	2 (6.5)	3 (10.0)	1 (3.4)
HADS depression score	8.7 (5.1)	8.6 (5.0)	7.4 (3.6)	1 (3.2)	3 (10.0)	2 (6.9)
HADS anxiety score	12.6 (5.3)	12.0 (5.3)	10.9 (3.9)	1 (3.2)	4 (13.3)	1 (3.4)
CAMS-R mindfulness score	28.6 (6.1)	28.8 (7.1)	30.3 (5.4)	3 (9.7)	5 (16.7)	3 (10.3)
CPG disability score	60.6 (24.4)	64.6 (19.6)	59.2 (24.4)	1 (3.2)	3 (10.0)	1 (3.4)
PSEQ Self efficacy score	29.1 (14.7)	27.9 (14.6)	35.5 (10.6)	1 (3.2)	3 (10.0)	2 (6.9)
Sexual health outcomes:						
SHOW-Q global score*	45.4 (20.3)	50.9 (20.9)	58.1 (22.2)	5 (16.1)	7 (23.3)	3 (10.3)
SHOW-Q pelvic problem interference score	47.1 (29.0)	49.0 (32.7)	56.4 (25.9)	8 (25.8)	6 (20.0)	3 (10.3)
MYMOP subjective outcome score	4.1 (1.2)	3.9 (1.3)	3.9 (1.1)	1 (3.2)	3 (10.0)	2 (6.9)
SF-36 Scales:						
SF36 - Physical functioning	56.3 (30.2)	55.8 (32.2)	66.5 (30.4)	3 (9.7)	4 (13.3)	2 (6.9)
SF36 - Pain	35.1 (17.5)	34.7 (20.6)	37.6 (20.6)	1 (3.2)	3 (10.0)	1 (3.4)
SF36 - General Health	39.1 (20.3)	42.0 (19.8)	37.9 (21.4)	2 (6.5)	3 (10.0)	1 (3.4)
SF36 - Social functioning	37.5 (19.1)	38.0 (28.3)	50.4 (25.3)	1 (3.2)	3 (10.0)	1 (3.4)

*Show-Q global is only applicable for sexually active participants. At baseline there are 17 sexually active women in the intervention group, 22 in the active control group and 19 in the usual care group.

For peer review only - http://bmjopen.bmj.com/site/about/guidelines.xhtml

Barts and The London School of Medicine and Dentistry

PCTU

Table 3. Baseline demographics of woman by 6 month questionnaire completion

Figures are mean (SD) unless stated otherwise.

	6 month follow-up questionnaire returned (N=33)	6 month follow-up questionnaire answered by phone (N=24)	6 month follow-up questionnaire never returned (N=33)
**Demographics**			
Age (Years)	35.8 (8.0)	36.6 (9.2)	33.1 (7.5)
Body mass index (kg/m^2)	27.4 (7.1)	27.7 (6.5)	25.9 (4.5)
Living arrangements - no. (%)			
Alone	2 (6.3)	1 (4.2)	3 (10.7)
With others	30 (93.8)	23 (95.8)	25 (89.3)
Employment status - no. (%)			
Employed	26 (78.8)	13 (54.2)	17 (60.7)
Unemployed and looking for work	1 (3.0)	1 (4.2)	1 (3.6)
At school or in full time education	1 (3.0)	2 (8.3)	4 (14.3)
Unable to work due to long term sickness	3 (9.1)	4 (16.7)	3 (10.7)
Looking after your home/family	2 (6.1)	3 (12.5)	3 (10.7)
Retired from paid work	0 (0.0)	1 (4.2)	0 (0.0)
Age left full time education - no. (%)			
Age 12 or less	0 (0.0)	3 (12.5)	0 (0.0)
Age 13 to 16	2 (6.1)	6 (25.0)	8 (29.6)
Age 17 to 19	7 (21.2)	2 (8.3)	5 (18.5)
Age 20 or over	23 (69.7)	9 (37.5)	10 (37.0)
Still in education	1 (3.0)	4 (16.7)	4 (14.8)
Ethnic group - no. (%)			
White	18 (58.1)	9 (40.9)	8 (30.8)
Black	7 (22.6)	4 (18.2)	2 (7.7)
Central Asian	0 (0.0)	0 (0.0)	2 (7.7)
Middle Eastern	0 (0.0)	0 (0.0)	1 (3.8)
Southern Asian	5 (16.1)	6 (27.3)	7 (26.9)
Mixed	1 (3.2)	0 (0.0)	1 (3.8)
Other ethnic group	0 (0.0)	2 (9.1)	4 (15.4)
Do not wish to say	0 (0.0)	1 (4.5)	1 (3.8)

For peer review only - http://bmjopen.bmj.com/site/about/guidelines.xhtml

Barts and The London School of Medicine and Dentistry

PCTU

	6 month follow-up questionnaire returned (N=33)	6 month follow-up questionnaire answered by phone (N=24)	6 month follow-up questionnaire never returned (N=33)
Smoker - no. (%)			
Yes	6 (18.8)	4 (18.2)	7 (25.9)
No	26 (81.3)	18 (81.8)	20 (74.1)
If yes, number of cigarettes per week	36.0 (24.1)	15.3 (12.5)	44.0 (30.8)
Drink alcohol - no. (%)			
Yes	18 (56.3)	6 (27.3)	10 (37.0)
No	14 (43.8)	16 (72.7)	17 (63.0)
If yes, number of units per week	8.9 (7.2)	5.8 (5.3)	5.2 (2.9)
**Baseline medical history**			
Duration of pain - no. (%)			
0 to 6 months	0 (0.0)	1 (4.2)	1 (3.6)
7 to 12 months	3 (9.1)	0 (0.0)	5 (17.9)
1 to 2 years	6 (18.2)	3 (12.5)	4 (14.3)
3 to 5 years	10 (30.3)	10 (41.7)	6 (21.4)
6 to 10 years	5 (15.2)	3 (12.5)	3 (10.7)
More than 10 years	9 (27.3)	7 (29.2)	9 (32.1)
Pain over the past week	6.0 (2.5)	6.0 (2.6)	7.5 (2.2)
**Baseline values of clinical outcomes**			
CPAQ pain acceptance score	25.3 (8.4)	20.8 (8.8)	21.4 (8.7)
HADS depression score	6.6 (3.6)	8.5 (4.9)	10.0 (4.9)
HADS anxiety score	10.3 (4.7)	12.2 (5.1)	13.5 (4.5)
CPG disability score	54.5 (18.8)	65.4 (20.7)	66.0 (27.4)

For peer review only - http://bmjopen.bmj.com/site/about/guidelines.xhtml

Barts and The London School of Medicine and Dentistry

PCTU

1. Feasibility Outcomes

1.1. Follow-up

Table 4. Losses to follow up

	Intervention (N=31)	Active control (N=30)	Usual care (N=29)
**Follow-up questionnaire returned - no (%)**			
60 days	15 (48.4)	9 (30.0)	18 (62.1)
3 months	17 (54.8)	12 (40.0)	17 (58.6)
6 months	11 (35.5)	10 (33.3)	12 (41.4)
**Follow-up questionnaire answered by phone - no (%)**			
60 days	1 (3.2)	8 (26.7)	1 (3.4)
3 months	3 (9.7)	7 (23.3)	3 (10.3)
6 months	10 (32.3)	6 (20.0)	8 (27.6)
**Follow-up questionnaire never returned - no (%)**			
60 days	15 (48.4)	13 (43.3)	10 (34.5)
3 months	11 (35.5)	11 (36.7)	9 (31.0)
6 months	10 (32.3)	14 (46.7)	9 (31.0)

For peer review only - http://bmjopen.bmj.com/site/about/guidelines.xhtml

Figure 1. Figure 1: Proportion of participants answering follow up questionnaire

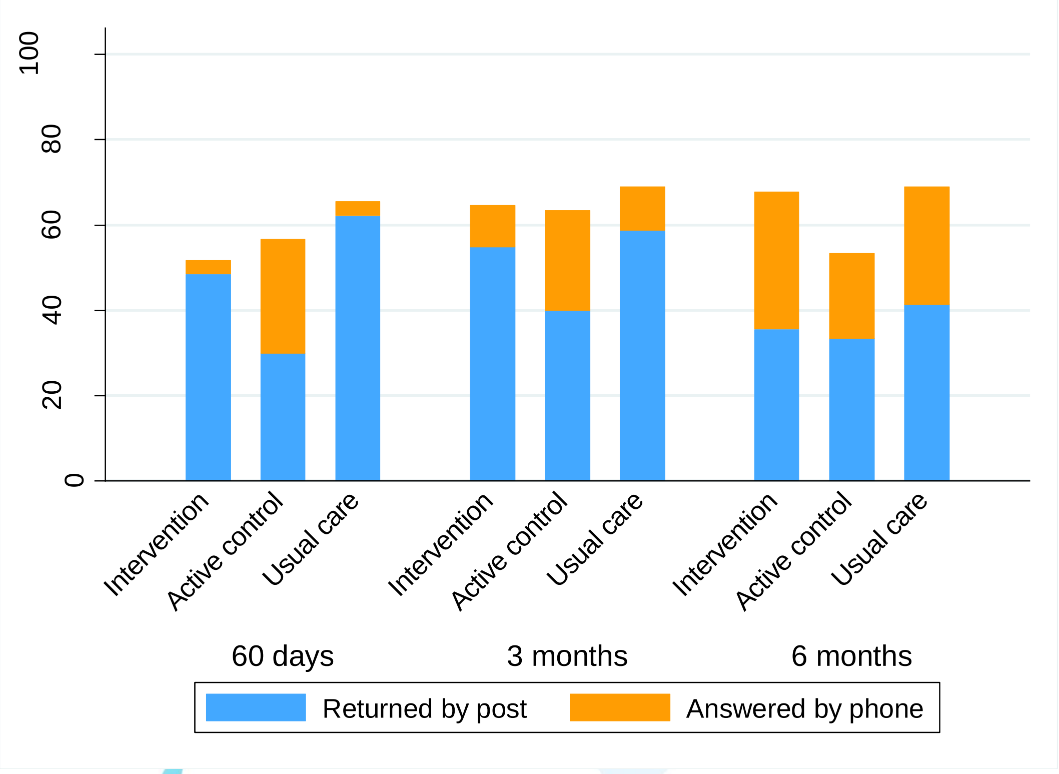

For peer review only - http://bmjopen.bmj.com/site/about/guidelines.xhtml

Table 5. Follow-up questionnaire returned or answered by phone within target follow up period

Figures are no returning data on time/no. returning data questionnaire answering by phone (%)*.

	Intervention (N=31)	Active control (N=30)	Usual care (N=29)
60 days (47 and 74 days)	7/16 (43.8)	6/17 (35.3)	11/19 (57.9)
3 months (76 and 104 days)	7/20 (35.0)	6/19 (31.6)	11/20 (55.0)
6 months (159 and 201 days)	7/21 (33.3)	6/16 (37.5)	11/20 (55.0)

*Denominator for percentage is number returning data questionnaire answering by phone

For peer review only - http://bmjopen.bmj.com/site/about/guidelines.xhtml

Barts and The London School of Medicine and Dentistry

PCTU

1.2. Standard deviation of CPAQ

Table 6. Estimated standard deviation of CPAQ

	Number with complete outcome	Estimated standard deviation
60 days	50	9.6
3 months	55	8.1
6 months	56	9.6

For peer review only - http://bmjopen.bmj.com/site/about/guidelines.xhtml

Barts and The London School of Medicine and Dentistry

PCTU

1.4. Blinding

Table 7. Unintentional unbinding of randomised treatment

Figures are number (%)

	Summary measure		Missing data	
	**Intervention (N=31)**	**Active control (N=30)**	**Intervention - no. (%)**	**Active control - no. (%)**
Researchers: Which app treatment do you believe the participant was randomised to?			2 (6.5)	3 (10.0)
Intervention app	0 (0.0)	1 (3.7)		
Control app	0 (0.0)	0 (0.0)		
Don't know	29 (100.0)	26 (96.3)		
Participants: Do you think you received the new treatment or comparison treatment?			15 (48.4)	19 (63.3)
New treatment	1 (6.3)	1 (9.1)		
Comparison treatment	0 (0.0)	1 (9.1)		
Don't know	15 (93.8)	9 (81.8)		

For peer review only - http://bmjopen.bmj.com/site/about/guidelines.xhtml

2. App satisfaction questionnaires

Table 8. System usability scale

Figures are mean (sd).

	Summary measure		Missing Data	
	Intervention (N=31)	Active control (N=30)	Intervention - no. (%)	Active control - no. (%)
System usability scale	50.7 (6.6)	46.0 (12.0)	16 (51.6)	18 (60.0)

For peer review only - http://bmjopen.bmj.com/site/about/guidelines.xhtml

Barts and The London School of Medicine and Dentistry

PCTU

Table 9. App usability Questionnaire

Figures are number (%).

	Totally disagree	Somewhat disagree	Neither agree nor disagree	Somewhat agree	Totally agree	Not answered
It is easy to use the app whenever I wanted to use it						
Intervention:	0 (0.0)	3 (9.7)	1 (3.2)	3 (9.7)	9 (29.0)	15 (48.4)
Active control:	0 (0.0)	0 (0.0)	0 (0.0)	4 (13.3)	8 (26.7)	18 (60.0)
After being shown, I understood how the app would work						
Intervention:	0 (0.0)	1 (3.2)	1 (3.2)	4 (12.9)	9 (29.0)	16 (51.6)
Active control:	0 (0.0)	2 (6.7)	0 (0.0)	2 (6.7)	8 (26.7)	18 (60.0)
It was fun to work with the app						
Intervention:	0 (0.0)	2 (6.5)	3 (9.7)	8 (25.8)	2 (6.5)	16 (51.6)
Active control:	0 (0.0)	3 (10.0)	3 (10.0)	5 (16.7)	1 (3.3)	18 (60.0)
The app worked well						
Intervention:	0 (0.0)	3 (9.7)	2 (6.5)	5 (16.1)	5 (16.1)	16 (51.6)
Active control:	0 (0.0)	2 (6.7)	2 (6.7)	4 (13.3)	4 (13.3)	18 (60.0)
It was easy to work through the modules						
Intervention:	0 (0.0)	4 (12.9)	0 (0.0)	5 (16.1)	6 (19.4)	16 (51.6)
Active control:	0 (0.0)	2 (6.7)	3 (10.0)	4 (13.3)	3 (10.0)	18 (60.0)
The number of modules was annoying						
Intervention:	1 (3.2)	4 (12.9)	6 (19.4)	5 (16.1)	0 (0.0)	15 (48.4)
Active control:	2 (6.7)	3 (10.0)	3 (10.0)	2 (6.7)	2 (6.7)	18 (60.0)
The modules were well-displayed on my smartphone						
Intervention:	0 (0.0)	2 (6.5)	1 (3.2)	8 (25.8)	5 (16.1)	15 (48.4)
Active control:	0 (0.0)	0 (0.0)	2 (6.7)	2 (6.7)	7 (23.3)	19 (63.3)
Using the app was difficult because of my daily activities						
Intervention:	2 (6.5)	2 (6.5)	3 (9.7)	9 (29.0)	0 (0.0)	15 (48.4)
Active control:	1 (3.3)	1 (3.3)	0 (0.0)	5 (16.7)	5 (16.7)	18 (60.0)
Using the app took too long						
Intervention:	2 (6.5)	4 (12.9)	3 (9.7)	7 (22.6)	0 (0.0)	15 (48.4)
Active control:	1 (3.3)	3 (10.0)	2 (6.7)	4 (13.3)	2 (6.7)	18 (60.0)

For peer review only - http://bmjopen.bmj.com/site/about/guidelines.xhtml

Barts and The London
School of Medicine and Dentistry

PCTU

3. Clinical Outcomes

3.1. Ranges of clinical outcomes

Pain acceptance score (as measured by the Chronic Pain Acceptance Questionnaire (CPAQ)):

- 0 (worst) – 48 (best)

Depression score (Hospital Anxiety and Depression Scale (HADS)):

- 0 (best) – 21 (worst)

Anxiety score (measured by HADS):

- 0 (best) – 21 (worst)

Mindfulness score (Cognitive and Mindfulness - Revised (CAMS – R) scale):

- 12 (worst) – 48 (best)

Pain related disability score (as measured by the Chronic Pain Grade (CPG) disability subscale):

- 0 (best) – 100 (worst)

Self efficacy score (as measured by the Pain Self-Efficacy Questionnaire (PSEQ)):

- 0 (worst) – 60 (best)

Sexual Health Outcomes scores (as measured by the Sexual Health Outcomes in Women Questionnaire (SHOW-Q))

- SHOW-Q global score, for sexually active participants: 0 (worst) – 100 (best)
- SHOW-Q pelvic interference score, for all participants: 0 (best) – 100 (worst)

Subjective outcome score (as measured by the Measure Yourself Medical Outcome Profile (MYMOP)):

- 0 (best) – 6)worst)

RAND Short form (36) Health Survey (RAND SF-36) scales:

- Physical functioning: 0 (worst) – 100)best)
- Pain: 0 (worst) – 100 (best)
- General health: 0 (worst) – 100 (best)
- Social functioning: 0 (worst) – 100 (best)

For peer review only - http://bmjopen.bmj.com/site/about/guidelines.xhtml

Barts and The London School of Medicine and Dentistry

PCTU

3.2. Completeness of clinical data

Table 10. Partially missing clinical outcomes

Figures are number (%)

	Not completed* no. (%)	Partially completed** no. (%)	Fully completed*** no. (%)
**CPAQ pain acceptance score**			
Baseline	5 (5.6)	1 (1.1)	84 (93.3)
60 days	40 (44.4)	0 (0.0)	50 (55.6)
3 months	32 (35.6)	3 (3.3)	55 (61.1)
6 months	34 (37.8)	0 (0.0)	56 (62.2)
**HADS depression score**			
Baseline	5 (5.6)	1 (1.1)	84 (93.3)
60 days	40 (44.4)	1 (1.1)	49 (54.4)
3 months	32 (35.6)	0 (0.0)	58 (64.4)
6 months	33 (36.7)	0 (0.0)	57 (63.3)
**HADS anxiety score**			
Baseline	5 (5.6)	1 (1.1)	84 (93.3)
60 days	40 (44.4)	0 (0.0)	50 (55.6)
3 months	32 (35.6)	0 (0.0)	58 (64.4)
6 months	33 (36.7)	0 (0.0)	57 (63.3)
**CAMS-R mindfulness score**			
Baseline	5 (5.6)	6 (6.7)	79 (87.8)
60 days	40 (44.4)	0 (0.0)	50 (55.6)
3 months	32 (35.6)	2 (2.2)	56 (62.2)
6 months	33 (36.7)	0 (0.0)	57 (63.3)
**CPG disability score**			
Baseline	5 (5.6)	0 (0.0)	85 (94.4)
60 days	40 (44.4)	0 (0.0)	50 (55.6)
3 months	33 (36.7)	0 (0.0)	57 (63.3)
6 months	34 (37.8)	0 (0.0)	56 (62.2)
**PSEQ Self efficacy score**			
Baseline	5 (5.6)	1 (1.1)	84 (93.3)
60 days	50 (55.6)	1 (1.1)	39 (43.3)
3 months	45 (50.0)	0 (0.0)	45 (50.0)
6 months	57 (63.3)	0 (0.0)	33 (36.7)

* Questionnaire not answered or all variables used in the derivation of the outcome are missing.
** One or more, but not all, variables used in the derivation of the outcome are missing.
*** No variables used in the derivation of the outcome are missing.

For peer review only - http://bmjopen.bmj.com/site/about/guidelines.xhtml

	Not completed* no. (%)	Partially completed** no. (%)	Fully completed*** no. (%)
**SHOW-Q global score**			
Baseline	5 (5.6)	15 (16.7)	70 (77.8)
60 days	50 (55.6)	6 (6.7)	34 (37.8)
3 months	47 (52.2)	8 (8.9)	35 (38.9)
6 months	58 (64.4)	5 (5.6)	27 (30.0)
**SHOW-Q pelvic problem interference score**			
Baseline	9 (10.0)	8 (8.9)	73 (81.1)
60 days	51 (56.7)	3 (3.3)	36 (40.0)
3 months	49 (54.4)	3 (3.3)	38 (42.2)
6 months	60 (66.7)	1 (1.1)	29 (32.2)
**MYMOP subjective outcome score**			
Baseline	5 (5.6)	1 (1.1)	84 (93.3)
60 days	38 (42.2)	11 (12.2)	41 (45.6)
3 months	33 (36.7)	10 (11.1)	47 (52.2)
6 months	33 (36.7)	6 (6.7)	51 (56.7)
**SF36 - General Health**			
Baseline	5 (5.6)	1 (1.1)	84 (93.3)
60 days	38 (42.2)	11 (12.2)	41 (45.6)
3 months	31 (34.4)	14 (15.6)	45 (50.0)
6 months	33 (36.7)	24 (26.7)	33 (36.7)
**SF36 - Physical functioning**			
Baseline	5 (5.6)	4 (4.4)	81 (90.0)
60 days	48 (53.3)	3 (3.3)	39 (43.3)
3 months	45 (50.0)	2 (2.2)	43 (47.8)
6 months	57 (63.3)	3 (3.3)	30 (33.3)
**SF36 - Pain**			
Baseline	5 (5.6)	0 (0.0)	85 (94.4)
60 days	48 (53.3)	0 (0.0)	42 (46.7)
3 months	45 (50.0)	0 (0.0)	45 (50.0)
6 months	57 (63.3)	0 (0.0)	33 (36.7)
**SF36 - Social functioning**			
Baseline	5 (5.6)	0 (0.0)	85 (94.4)
60 days	48 (53.3)	1 (1.1)	41 (45.6)
3 months	45 (50.0)	1 (1.1)	44 (48.9)
6 months	57 (63.3)	1 (1.1)	32 (35.6)

* Questionnaire not answered or all variables used in the derivation of the outcome are missing.
** One or more, but not all, variables used in the derivation of the outcome are missing.
*** No variables used in the derivation of the outcome are missing.

For peer review only - http://bmjopen.bmj.com/site/about/guidelines.xhtml

Table 11. Partially missing clinical outcomes by method of questionnaire delivery

Figures are number (%)

	Questionnaire never returned no. (%)	Questionnaire answered by telephone: Not completed* no. (%)	Questionnaire answered by telephone: Partially completed** no. (%)	Questionnaire answered by telephone: Fully completed*** no. (%)	Questionnaire returned: Not completed* no. (%)	Questionnaire returned: Partially completed** no. (%)	Questionnaire returned: Fully completed*** no. (%)
**CPAQ pain acceptance score**							
Baseline	5 (5.6)	n/a	n/a	n/a	0 (0.0)	1 (1.1)	84 (93.3)
60 days	38 (42.2)	1 (1.1)	0 (0.0)	9 (10.0)	1 (1.1)	0 (0.0)	41 (45.6)
3 months	31 (34.4)	0 (0.0)	0 (0.0)	13 (14.4)	1 (1.1)	3 (3.3)	42 (46.7)
6 months	33 (36.7)	1 (1.1)	0 (0.0)	23 (25.6)	0 (0.0)	0 (0.0)	33 (36.7)
**HADS depression score**							
Baseline	5 (5.6)	n/a	n/a	n/a	0 (0.0)	1 (1.1)	84 (93.3)
60 days	38 (42.2)	1 (1.1)	0 (0.0)	9 (10.0)	1 (1.1)	1 (1.1)	40 (44.4)
3 months	31 (34.4)	0 (0.0)	0 (0.0)	13 (14.4)	1 (1.1)	0 (0.0)	45 (50.0)
6 months	33 (36.7)	0 (0.0)	0 (0.0)	24 (26.7)	0 (0.0)	0 (0.0)	33 (36.7)
**HADS anxiety score**							
Baseline	5 (5.6)	n/a	n/a	n/a	0 (0.0)	1 (1.1)	84 (93.3)
60 days	38 (42.2)	1 (1.1)	0 (0.0)	9 (10.0)	1 (1.1)	0 (0.0)	41 (45.6)
3 months	31 (34.4)	0 (0.0)	0 (0.0)	13 (14.4)	1 (1.1)	0 (0.0)	45 (50.0)
6 months	33 (36.7)	0 (0.0)	0 (0.0)	24 (26.7)	0 (0.0)	0 (0.0)	33 (36.7)
**CAMS-R mindfulness score**							
Baseline	5 (5.6)	n/a	n/a	n/a	0 (0.0)	6 (6.7)	79 (87.8)
60 days	38 (42.2)	1 (1.1)	0 (0.0)	9 (10.0)	1 (1.1)	0 (0.0)	41 (45.6)
3 months	31 (34.4)	0 (0.0)	0 (0.0)	13 (14.4)	1 (1.1)	2 (2.2)	43 (47.8)
6 months	33 (36.7)	0 (0.0)	0 (0.0)	24 (26.7)	0 (0.0)	0 (0.0)	33 (36.7)
**CPG disability score**							
Baseline	5 (5.6)	n/a	n/a	n/a	0 (0.0)	0 (0.0)	85 (94.4)
60 days	38 (42.2)	1 (1.1)	0 (0.0)	9 (10.0)	1 (1.1)	0 (0.0)	41 (45.6)
3 months	31 (34.4)	0 (0.0)	0 (0.0)	13 (14.4)	2 (2.2)	0 (0.0)	44 (48.9)
6 months	33 (36.7)	1 (1.1)	0 (0.0)	23 (25.6)	0 (0.0)	0 (0.0)	33 (36.7)

* Questionnaire not answered or all variables used in the derivation of the outcome are missing.
** One or more, but not all, variables used in the derivation of the outcome are missing.
*** No variables used in the derivation of the outcome are missing.

For peer review only - http://bmjopen.bmj.com/site/about/guidelines.xhtml

Barts and The London School of Medicine and Dentistry

PCTU

	Questionnaire never returned no. (%)	Questionnaire answered by telephone			Questionnaire returned		
		Not completed* no. (%)	Partially completed** no. (%)	Fully completed*** no. (%)	Not completed* no. (%)	Partially completed** no. (%)	Fully completed*** no. (%)
**PSEQ Self efficacy score**							
Baseline	5 (5.6)	n/a	n/a	n/a	0 (0.0)	1 (1.1)	84 (93.3)
60 days	38 (42.2)	10 (11.1)	0 (0.0)	0 (0.0)	2 (2.2)	1 (1.1)	39 (43.3)
3 months	31 (34.4)	13 (14.4)	0 (0.0)	0 (0.0)	1 (1.1)	0 (0.0)	45 (50.0)
6 months	33 (36.7)	24 (26.7)	0 (0.0)	0 (0.0)	0 (0.0)	0 (0.0)	33 (36.7)
**SHOW-Q global score**							
Baseline	5 (5.6)	n/a	n/a	n/a	0 (0.0)	15 (16.7)	70 (77.8)
60 days	38 (42.2)	10 (11.1)	0 (0.0)	0 (0.0)	2 (2.2)	6 (6.7)	34 (37.8)
3 months	31 (34.4)	13 (14.4)	0 (0.0)	0 (0.0)	3 (3.3)	8 (8.9)	35 (38.9)
6 months	33 (36.7)	24 (26.7)	0 (0.0)	0 (0.0)	1 (1.1)	5 (5.6)	27 (30.0)
**SHOW-Q pelvic problem interference score**							
Baseline	5 (5.6)	n/a	n/a	n/a	4 (4.4)	8 (8.9)	73 (81.1)
60 days	38 (42.2)	10 (11.1)	0 (0.0)	0 (0.0)	3 (3.3)	3 (3.3)	36 (40.0)
3 months	31 (34.4)	13 (14.4)	0 (0.0)	0 (0.0)	5 (5.6)	3 (3.3)	38 (42.2)
6 months	33 (36.7)	24 (26.7)	0 (0.0)	0 (0.0)	3 (3.3)	1 (1.1)	29 (32.2)
**MYMOP subjective outcome score**							
Baseline	5 (5.6)	n/a	n/a	n/a	0 (0.0)	1 (1.1)	84 (93.3)
60 days	38 (42.2)	0 (0.0)	1 (1.1)	9 (10.0)	0 (0.0)	10 (11.1)	32 (35.6)
3 months	31 (34.4)	0 (0.0)	1 (1.1)	12 (13.3)	2 (2.2)	9 (10.0)	35 (38.9)
6 months	33 (36.7)	0 (0.0)	2 (2.2)	22 (24.4)	0 (0.0)	4 (4.4)	29 (32.2)

* Questionnaire not answered or all variables used in the derivation of the outcome are missing.
** One or more, but not all, variables used in the derivation of the outcome are missing.
*** No variables used in the derivation of the outcome are missing.

For peer review only - http://bmjopen.bmj.com/site/about/guidelines.xhtml
PCTU only

	Questionnaire never returned no. (%)	Questionnaire answered by telephone			Questionnaire returned		
		Not completed* no. (%)	Partially completed** no. (%)	Fully completed*** no. (%)	Not completed* no. (%)	Partially completed** no. (%)	Fully completed*** no. (%)
**SF36 - General Health**							
Baseline	5 (5.6)	n/a	n/a	n/a	0 (0.0)	1 (1.1)	84 (93.3)
60 days	38 (42.2)	10 (11.1)	0 (0.0)	0 (0.0)	0 (0.0)	1 (1.1)	41 (45.6)
3 months	31 (34.4)	13 (14.4)	0 (0.0)	0 (0.0)	0 (0.0)	1 (1.1)	45 (50.0)
6 months	33 (36.7)	24 (26.7)	0 (0.0)	0 (0.0)	0 (0.0)	0 (0.0)	33 (36.7)
**SF36 - Physical functioning**							
Baseline	5 (5.6)	n/a	n/a	n/a	0 (0.0)	4 (4.4)	81 (90.0)
60 days	38 (42.2)	10 (11.1)	0 (0.0)	0 (0.0)	0 (0.0)	3 (3.3)	39 (43.3)
3 months	31 (34.4)	13 (14.4)	0 (0.0)	0 (0.0)	1 (1.1)	2 (2.2)	43 (47.8)
6 months	33 (36.7)	24 (26.7)	0 (0.0)	0 (0.0)	0 (0.0)	3 (3.3)	30 (33.3)
**SF36 - Pain**							
Baseline	5 (5.6)	n/a	n/a	n/a	0 (0.0)	0 (0.0)	85 (94.4)
60 days	38 (42.2)	10 (11.1)	0 (0.0)	0 (0.0)	0 (0.0)	0 (0.0)	42 (46.7)
3 months	31 (34.4)	13 (14.4)	0 (0.0)	0 (0.0)	1 (1.1)	0 (0.0)	45 (50.0)
6 months	33 (36.7)	24 (26.7)	0 (0.0)	0 (0.0)	0 (0.0)	0 (0.0)	33 (36.7)
**SF36 - Social functioning**							
Baseline	5 (5.6)	n/a	n/a	n/a	0 (0.0)	0 (0.0)	85 (94.4)
60 days	38 (42.2)	10 (11.1)	0 (0.0)	0 (0.0)	0 (0.0)	1 (1.1)	41 (45.6)
3 months	31 (34.4)	13 (14.4)	0 (0.0)	0 (0.0)	1 (1.1)	1 (1.1)	44 (48.9)
6 months	33 (36.7)	24 (26.7)	0 (0.0)	0 (0.0)	0 (0.0)	1 (1.1)	32 (35.6)

* Questionnaire not answered or all variables used in the derivation of the outcome are missing.
** One or more, but not all, variables used in the derivation of the outcome are missing.
*** No variables used in the derivation of the outcome are missing.

For peer review only - http://bmjopen.bmj.com/site/about/guidelines.xhtml

3.3. Results of analysis of clinical outcomes

Table 12. Descriptive statistics for clinical outcomes

	Intervention (N=31)		Active control (N=30)		Usual care (N=29)	
	no. (%)	mean (sd)	no. (%)	mean (sd)	no. (%)	mean (sd)
**CPAQ pain acceptance score**						
Baseline	29 (93.5)	21.9 (9.5)	27 (90.0)	22.7 (8.4)	28 (96.6)	23.8 (8.5)
60 days	15 (48.4)	21.5 (10.2)	16 (53.3)	22.9 (8.5)	19 (65.5)	24.3 (10.2)
3 months	18 (58.1)	20.8 (7.2)	18 (60.0)	22.9 (8.5)	19 (65.5)	25.0 (8.4)
6 months	21 (67.7)	22.7 (10.1)	16 (53.3)	24.0 (11.2)	19 (65.5)	25.8 (7.6)
Included in analysis*	27 (87.1)		23 (76.7)		25 (86.2)	
**HADS depression score**						
Baseline	30 (96.8)	8.7 (5.1)	27 (90.0)	8.6 (5.0)	27 (93.1)	7.4 (3.6)
60 days	14 (45.2)	7.1 (5.2)	16 (53.3)	8.4 (4.0)	19 (65.5)	8.2 (2.9)
3 months	20 (64.5)	8.7 (3.9)	19 (63.3)	8.2 (5.0)	19 (65.5)	6.8 (3.6)
6 months	21 (67.7)	7.0 (4.9)	16 (53.3)	6.1 (4.4)	20 (69.0)	7.0 (4.6)
Included in analysis*	27 (87.1)		23 (76.7)		26 (89.7)	
**HADS anxiety score**						
Baseline	30 (96.8)	12.6 (5.3)	26 (86.7)	12.0 (5.3)	28 (96.6)	10.9 (3.9)
60 days	15 (48.4)	12.5 (5.6)	16 (53.3)	9.5 (4.1)	19 (65.5)	10.7 (4.1)
3 months	20 (64.5)	12.2 (4.1)	19 (63.3)	9.7 (5.6)	19 (65.5)	10.2 (4.0)
6 months	21 (67.7)	10.1 (4.9)	16 (53.3)	8.4 (5.5)	20 (69.0)	9.1 (4.7)
Included in analysis*	27 (87.1)		23 (76.7)		26 (89.7)	
**CAMS-R mindfulness score**						
Baseline	28 (90.3)	28.6 (6.1)	25 (83.3)	28.8 (7.1)	26 (89.7)	30.3 (5.4)
60 days	15 (48.4)	27.4 (5.6)	16 (53.3)	30.6 (8.4)	19 (65.5)	29.7 (7.6)
3 months	19 (61.3)	29.2 (5.2)	19 (63.3)	30.9 (8.8)	18 (62.1)	31.4 (6.4)
6 months	21 (67.7)	29.0 (7.6)	16 (53.3)	31.0 (7.3)	20 (69.0)	32.0 (8.5)
Included in analysis*	27 (87.1)		23 (76.7)		26 (89.7)	

* Included in analysis if outcome is available for at least one follow-up time point.

For peer review only - http://bmjopen.bmj.com/site/about/guidelines.xhtml

Barts and The London School of Medicine and Dentistry

PCTU

	Intervention (N=31)		Active control (N=30)		Usual care (N=29)	
	no. (%)	mean (sd)	no. (%)	mean (sd)	no. (%)	mean (sd)
**CPG disability score**						
Baseline	30 (96.8)	60.6 (24.4)	27 (90.0)	64.6 (19.6)	28 (96.6)	59.2 (24.4)
60 days	15 (48.4)	56.7 (19.8)	16 (53.3)	54.8 (25.0)	19 (65.5)	54.7 (22.9)
3 months	19 (61.3)	61.1 (17.3)	19 (63.3)	52.5 (27.5)	19 (65.5)	52.8 (23.5)
6 months	21 (67.7)	48.3 (28.1)	16 (53.3)	48.5 (24.4)	19 (65.5)	54.2 (23.7)
Included in analysis*	27 (87.1)		23 (76.7)		25 (86.2)	
**PSEQ Self efficacy score**						
Baseline	30 (96.8)	29.1 (14.7)	27 (90.0)	27.9 (14.6)	27 (93.1)	35.5 (10.6)
60 days	14 (45.2)	32.4 (13.9)	9 (30.0)	30.9 (15.9)	16 (55.2)	34.5 (13.1)
3 months	17 (54.8)	28.9 (11.8)	12 (40.0)	30.2 (14.2)	16 (55.2)	39.3 (9.7)
6 months	11 (35.5)	34.3 (12.5)	10 (33.3)	33.7 (17.7)	12 (41.4)	40.2 (13.1)
Included in analysis*	21 (67.7)		18 (60.0)		21 (72.4)	
**SHOW-Q global score**						
Baseline	17 (54.8)	45.4 (20.3)	20 (66.7)	50.9 (20.9)	19 (65.5)	58.1 (22.2)
60 days	4 (12.9)	69.3 (13.3)	8 (26.7)	54.1 (18.0)	13 (44.8)	53.7 (24.5)
3 months	5 (16.1)	51.1 (26.6)	11 (36.7)	44.9 (19.4)	10 (34.5)	61.2 (24.8)
6 months	7 (22.6)	52.3 (15.6)	4 (13.3)	60.9 (14.3)	7 (24.1)	58.5 (26.4)
Included in analysis*	9 (29.0)		14 (46.7)		16 (55.2)	
**SHOW-Q pelvic problem interference score**						
Baseline	23 (74.2)	47.1 (29.0)	24 (80.0)	49.0 (32.7)	26 (89.7)	56.4 (25.9)
60 days	12 (38.7)	60.4 (33.7)	9 (30.0)	60.2 (27.9)	15 (51.7)	51.7 (28.9)
3 months	12 (38.7)	54.9 (34.0)	11 (36.7)	50.0 (25.3)	15 (51.7)	69.4 (32.8)
6 months	9 (29.0)	65.7 (22.2)	9 (30.0)	59.3 (33.4)	11 (37.9)	57.6 (32.8)
Included in analysis*	16 (51.6)		17 (56.7)		20 (69.0)	
**MYMOP subjective outcome score**						
Baseline	30 (96.8)	4.1 (1.2)	27 (90.0)	3.9 (1.3)	27 (93.1)	3.9 (1.1)
60 days	13 (41.9)	3.2 (1.4)	14 (46.7)	3.5 (1.3)	14 (48.3)	3.6 (1.2)
3 months	15 (48.4)	3.4 (1.3)	16 (53.3)	3.1 (1.6)	16 (55.2)	2.9 (1.4)
6 months	18 (58.1)	3.0 (1.4)	15 (50.0)	3.0 (1.5)	18 (62.1)	3.1 (1.5)
Included in analysis*	25 (80.6)		21 (70.0)		24 (82.8)	

* Included in analysis if outcome is available for at least one follow-up time point.

For peer review only - http://bmjopen.bmj.com/site/about/guidelines.xhtml

Barts and The London School of Medicine and Dentistry PCTU

	Intervention (N=31)		Active control (N=30)		Usual care (N=29)	
	no. (%)	mean (sd)	no. (%)	mean (sd)	no. (%)	mean (sd)
**SF36 - General Health**						
Baseline	29 (93.5)	39.1 (20.3)	27 (90.0)	42.0 (19.8)	28 (96.6)	37.9 (21.4)
60 days	15 (48.4)	45.0 (21.2)	9 (30.0)	51.1 (19.2)	17 (58.6)	37.6 (19.9)
3 months	17 (54.8)	44.1 (21.7)	12 (40.0)	42.1 (23.2)	16 (55.2)	40.3 (19.4)
6 months	11 (35.5)	54.5 (19.0)	10 (33.3)	54.5 (24.2)	12 (41.4)	40.0 (27.8)
Included in analysis*	21 (67.7)		18 (60.0)		22 (75.9)	
**SF36 - Physical functioning**						
Baseline	28 (90.3)	56.3 (30.2)	26 (86.7)	55.8 (32.2)	27 (93.1)	66.5 (30.4)
60 days	13 (41.9)	61.2 (27.1)	9 (30.0)	60.6 (25.7)	17 (58.6)	66.5 (30.0)
3 months	15 (48.4)	58.3 (24.0)	12 (40.0)	54.6 (30.7)	16 (55.2)	69.1 (27.5)
6 months	10 (32.3)	66.0 (26.5)	10 (33.3)	72.0 (28.6)	10 (34.5)	63.5 (37.4)
Included in analysis*	20 (64.5)		18 (60.0)		22 (75.9)	
**SF36 - Pain**						
Baseline	30 (96.8)	35.1 (17.5)	27 (90.0)	34.7 (20.6)	28 (96.6)	37.6 (20.6)
60 days	15 (48.4)	39.0 (19.2)	9 (30.0)	43.1 (33.0)	18 (62.1)	40.0 (24.5)
3 months	17 (54.8)	43.7 (17.6)	12 (40.0)	46.7 (22.7)	16 (55.2)	49.5 (25.9)
6 months	11 (35.5)	50.0 (17.8)	10 (33.3)	61.0 (19.9)	12 (41.4)	48.3 (24.8)
Included in analysis*	21 (67.7)		18 (60.0)		22 (75.9)	
**SF36 - Social functioning**						
Baseline	30 (96.8)	37.5 (19.1)	27 (90.0)	38.0 (28.3)	28 (96.6)	50.4 (25.3)
60 days	15 (48.4)	45.8 (27.4)	9 (30.0)	55.6 (29.4)	17 (58.6)	51.5 (28.9)
3 months	17 (54.8)	50.7 (20.9)	12 (40.0)	49.0 (30.4)	15 (51.7)	57.5 (29.0)
6 months	11 (35.5)	54.5 (21.8)	10 (33.3)	56.3 (27.8)	11 (37.9)	59.1 (34.0)
Included in analysis*	21 (67.7)		18 (60.0)		22 (75.9)	

* Included in analysis if outcome is available for at least one follow-up time point.

For peer review only - http://bmjopen.bmj.com/site/about/guidelines.xhtml

Table 13. Estimated treatment effects for clinical outcomes

	Intervention vs Active control adjusted mean difference (95% CI)	Intervention vs Usual care adjusted mean difference (95% CI)	Active control vs Usual care adjusted mean difference (95% CI)
**CPAQ pain acceptance score**			
60 days	-2.3 (-6.6, 2.0)	-4.0 (-8.1, 0.1)	-1.7 (-5.8, 2.4)
3 months	-3.0 (-6.8, 0.7)	-4.5 (-8.2, -0.9)	-1.5 (-5.2, 2.2)
6 months	-1.4 (-5.8, 3.0)	-4.0 (-8.2, 0.2)	-2.5 (-7.0, 2.0)
**HADS depression score**			
60 days	-0.7 (-2.7, 1.2)	-1.2 (-3.1, 0.6)	-0.5 (-2.3, 1.3)
3 months	0.5 (-1.6, 2.6)	1.2 (-0.9, 3.4)	0.8 (-1.4, 2.9)
6 months	0.5 (-1.7, 2.6)	0.4 (-1.7, 2.4)	-0.1 (-2.3, 2.1)
**HADS anxiety score**			
60 days	2.0 (-0.1, 4.1)	1.0 (-1.1, 3.0)	-1.0 (-3.0, 1.0)
3 months	1.9 (-0.3, 4.0)	1.5 (-0.6, 3.6)	-0.4 (-2.5, 1.7)
6 months	0.1 (-2.3, 2.5)	0.3 (-2.0, 2.6)	0.2 (-2.2, 2.6)
**CAMS-R mindfulness score**			
60 days	-3.5 (-7.3, 0.4)	-2.2 (-5.9, 1.4)	1.2 (-2.5, 4.9)
3 months	-2.5 (-5.8, 0.8)	-2.3 (-5.5, 1.0)	0.2 (-3.1, 3.5)
6 months	-1.4 (-4.9, 2.2)	-2.9 (-6.3, 0.4)	-1.6 (-5.1, 2.0)
**CPG disability score**			
60 days	5.1 (-7.2, 17.5)	3.8 (-8.1, 15.7)	-1.4 (-13.1, 10.4)
3 months	8.8 (-3.4, 21.0)	7.6 (-4.5, 19.7)	-1.2 (-13.4, 10.9)
6 months	1.9 (-12.1, 16.0)	1.0 (-12.6, 14.5)	-1.0 (-15.3, 13.4)
**PSEQ Self efficacy score**			
60 days	0.1 (-8.2, 8.4)	-0.2 (-7.4, 6.9)	-0.3 (-8.4, 7.8)
3 months	-3.6 (-9.8, 2.6)	-7.1 (-12.9, -1.2)	-3.5 (-9.8, 2.9)
6 months	-5.9 (-14.8, 3.0)	-8.7 (-17.1, -0.2)	-2.8 (-11.6, 5.9)

For peer review only - http://bmjopen.bmj.com/site/about/guidelines.xhtml

Barts and The London School of Medicine and Dentistry PCTU

	Intervention vs Active control adjusted mean difference (95% CI)	Intervention vs Usual care adjusted mean difference (95% CI)	Active control vs Usual care adjusted mean difference (95% CI)
**SHOW-Q global score**			
60 days	7.0 (-7.2, 21.2)	8.3 (-5.2, 21.8)	1.3 (-9.8, 12.4)
3 months	3.5 (-13.9, 20.9)	-4.8 (-22.0, 12.3)	-8.3 (-23.2, 6.6)
6 months	-11.5 (-27.7, 4.8)	-10.7 (-25.8, 4.3)	0.7 (-14.5, 15.9)
**SHOW-Q pelvic problem interference score**			
60 days	-7.2 (-28.0, 13.5)	3.6 (-14.7, 21.9)	10.9 (-8.9, 30.7)
3 months	-1.2 (-25.1, 22.8)	-10.2 (-32.5, 12.1)	-9.0 (-31.9, 13.8)
6 months	3.3 (-21.3, 27.9)	4.7 (-18.7, 28.1)	1.4 (-22.1, 24.8)
**MYMOP subjective outcome score**			
60 days	0.0 (-0.7, 0.8)	-0.3 (-1.1, 0.4)	-0.4 (-1.1, 0.4)
3 months	0.6 (-0.2, 1.5)	0.6 (-0.2, 1.4)	-0.0 (-0.9, 0.8)
6 months	-0.2 (-1.1, 0.7)	0.2 (-0.7, 1.1)	0.4 (-0.6, 1.3)
**SF36 - General Health**			
60 days	-8.8 (-19.4, 1.8)	-0.9 (-10.0, 8.3)	7.9 (-2.5, 18.3)
3 months	2.0 (-7.3, 11.3)	-5.6 (-14.5, 3.3)	-7.6 (-17.1, 1.9)
6 months	-4.6 (-18.2, 8.9)	-1.9 (-14.9, 11.0)	2.7 (-10.8, 16.2)
**SF36 - Physical functioning**			
60 days	0.1 (-16.0, 16.2)	-6.5 (-20.9, 7.9)	-6.6 (-22.2, 9.0)
3 months	-4.9 (-19.0, 9.3)	-7.7 (-20.8, 5.4)	-2.8 (-16.8, 11.1)
6 months	-2.4 (-24.7, 19.9)	6.3 (-15.7, 28.2)	8.6 (-13.6, 30.9)
**SF36 - Pain**			
60 days	-3.7 (-19.8, 12.3)	0.5 (-12.9, 13.9)	4.2 (-11.4, 19.8)
3 months	-6.4 (-20.7, 7.9)	-7.3 (-20.8, 6.2)	-0.9 (-15.3, 13.6)
6 months	-8.5 (-22.8, 5.8)	0.7 (-13.0, 14.4)	9.2 (-5.0, 23.4)
**SF36 - Social functioning**			
60 days	-17.1 (-33.4, -0.7)	5.2 (-8.8, 19.1)	22.2 (5.7, 38.8)
3 months	-8.2 (-26.5, 10.1)	4.3 (-13.2, 21.8)	12.5 (-6.5, 31.5)
6 months	0.3 (-18.9, 19.6)	3.9 (-15.0, 22.8)	3.5 (-16.0, 23.1)

For peer review only - http://bmjopen.bmj.com/site/about/guidelines.xhtml

Figure 2. Figure 3: Estimated treatment effects and 95% confidence intervals for CPAQ

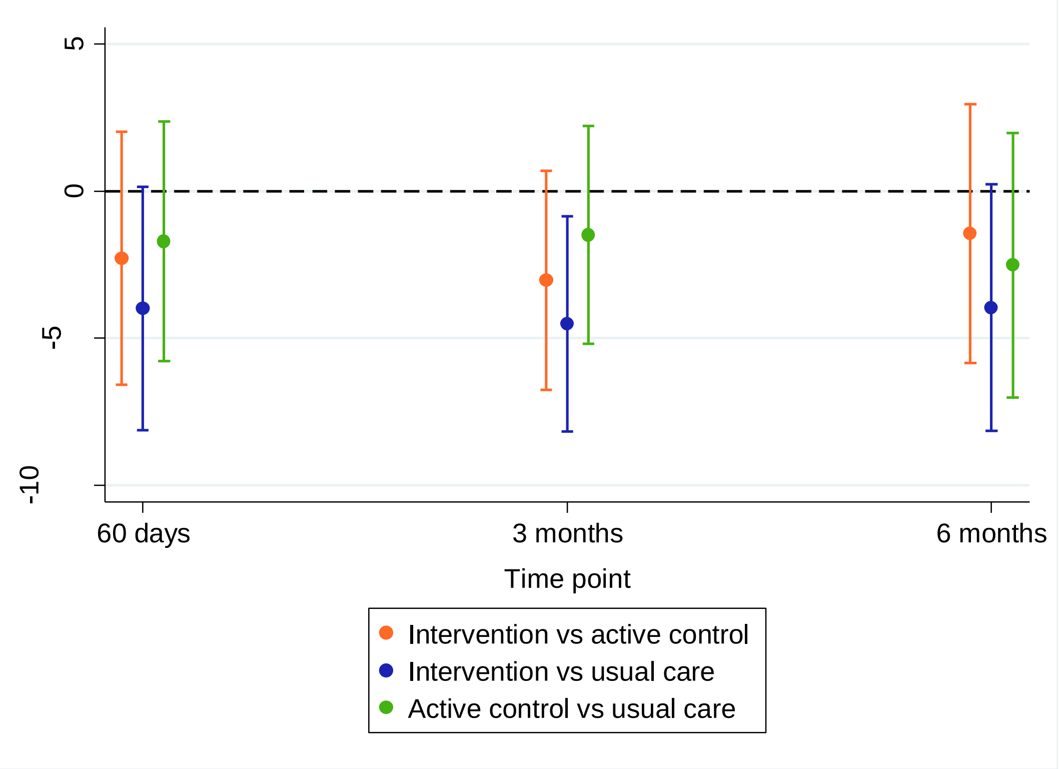

CONSORT 2010 checklist of information to include when reporting a pilot or feasibility trial*

Section/Topic	Item No	Checklist item	Reported on page No
**Title and abstract**			
	1a	Identification as a pilot or feasibility randomised trial in the title	1
	1b	Structured summary of pilot trial design, methods, results, and conclusions (for specific guidance see CONSORT abstract extension for pilot trials)	2
**Introduction**			
Background and objectives	2a	Scientific background and explanation of rationale for future definitive trial, and reasons for randomised pilot trial	4
	2b	Specific objectives or research questions for pilot trial	4
**Methods**			
Trial design	3a	Description of pilot trial design (such as parallel, factorial) including allocation ratio	5
	3b	Important changes to methods after pilot trial commencement (such as eligibility criteria), with reasons	n/a
Participants	4a	Eligibility criteria for participants	5
	4b	Settings and locations where the data were collected	6
	4c	How participants were identified and consented	5
Interventions	5	The interventions for each group with sufficient details to allow replication, including how and when they were actually administered	5
Outcomes	6a	Completely defined prespecified assessments or measurements to address each pilot trial objective specified in 2b, including how and when they were assessed	6-7
	6b	Any changes to pilot trial assessments or measurements after the pilot trial commenced, with reasons	7
	6c	If applicable, prespecified criteria used to judge whether, or how, to proceed with future definitive trial	n/a
Sample size	7a	Rationale for numbers in the pilot trial	8
	7b	When applicable, explanation of any interim analyses and stopping guidelines	n/a
Randomisation:			
Sequence generation	8a	Method used to generate the random allocation sequence	5
	8b	Type of randomisation(s); details of any restriction (such as blocking and block size)	5
Allocation concealment mechanism	9	Mechanism used to implement the random allocation sequence (such as sequentially numbered containers), describing any steps taken to conceal the sequence until interventions were assigned	5

For peer review only

Implementation	10	Who generated the random allocation sequence, who enrolled participants, and who assigned participants to interventions	5
Blinding	11a	If done, who was blinded after assignment to interventions (for example, participants, care providers, those assessing outcomes) and how	6
	11b	If relevant, description of the similarity of interventions	5
Statistical methods	12	Methods used to address each pilot trial objective whether qualitative or quantitative	8
**Results**			
Participant flow (a diagram is strongly recommended)	13a	For each group, the numbers of participants who were approached and/or assessed for eligibility, randomly assigned, received intended treatment, and were assessed for each objective	13
	13b	For each group, losses and exclusions after randomisation, together with reasons	13
Recruitment	14a	Dates defining the periods of recruitment and follow-up	8
	14b	Why the pilot trial ended or was stopped	8
Baseline data	15	A table showing baseline demographic and clinical characteristics for each group	15
Numbers analysed	16	For each objective, number of participants (denominator) included in each analysis. If relevant, these numbers should be by randomised group	Supplementary tables
Outcomes and estimation	17	For each objective, results including expressions of uncertainty (such as 95% confidence interval) for any estimates. If relevant, these results should be by randomised group	Supplementary tables
Ancillary analyses	18	Results of any other analyses performed that could be used to inform the future definitive trial	n/a
Harms	19	All important harms or unintended effects in each group (for specific guidance see CONSORT for harms)	Supplementary tables
	19a	If relevant, other important unintended consequences	n/a
**Discussion**			
Limitations	20	Pilot trial limitations, addressing sources of potential bias and remaining uncertainty about feasibility	9
Generalisability	21	Generalisability (applicability) of pilot trial methods and findings to future definitive trial and other studies	9
Interpretation	22	Interpretation consistent with pilot trial objectives and findings, balancing potential benefits and harms, and considering other relevant evidence	9
	22a	Implications for progression from pilot to future definitive trial, including any proposed amendments	9
**Other information**			
Registration	23	Registration number for pilot trial and name of trial registry	3
Protocol	24	Where the pilot trial protocol can be accessed, if available	Supplementary material
Funding	25	Sources of funding and other support (such as supply of drugs), role of funders	3

For peer review only

	26	Ethical approval or approval by research review committee, confirmed with reference number	8

Citation: Eldridge SM, Chan CL, Campbell MJ, Bond CM, Hopewell S, Thabane L, et al. CONSORT 2010 statement: extension to randomised pilot and feasibility trials. BMJ. 2016;355.

*We strongly recommend reading this statement in conjunction with the CONSORT 2010, extension to randomised pilot and feasibility trials, Explanation and Elaboration for important clarifications on all the items. If relevant, we also recommend reading CONSORT extensions for cluster randomised trials, non-inferiority and equivalence trials, non-pharmacological treatments, herbal interventions, and pragmatic trials. Additional extensions are forthcoming: for those and for up to date references relevant to this checklist, see www.consort-statement.org.

For peer review only

BMJ Open

A smartphone app using psychological approaches for women with chronic pelvic pain presenting to gynaecology clinics (MEMPHIS): a randomised feasibility trial

Journal:	*BMJ Open*
Manuscript ID	bmjopen-2019-030164.R2
Article Type:	Original research
Date Submitted by the Author:	22-Nov-2019
Complete List of Authors:	Forbes, Gordon; King's College London, IoPPN<br>Newton, Sian; Queen Mary University of London, Centre for Primary Care and Population Health<br>Cantalapiedra Calvete, Clara; Barts Health NHS Trust, United Kingdom, Department of Obstetrics and Gynaecology<br>Birch, Judy; Pelvic Pain Support Network<br>Dodds, Julie; Barts and The London School of Medicine and Dentistry, Women's Health Research Unit<br>Steed, Liz; Queen Mary University of London, Centre for Primary Care and Population Health<br>Rivas, Carol; University of Southamptom, Faculty of Health Sciences<br>Khan, Khalid; Barts and The London School of Medicine and Dentistry, Women's Health Research Unit<br>Röhricht, Frank; East London NHS Foundation Trust<br>Taylor, Stephanie; Queen Mary University of London, Centre for Primary Care and Public Health<br>Kahan, Brennan; Queen Mary University of London, Pragmatic Clinical Trials Unit<br>Ball, Elizabeth; Barts Health NHS Trust, Department of Obstetrics and Gynaecology
**Primary Subject Heading**:	Obstetrics and gynaecology
Secondary Subject Heading:	Obstetrics and gynaecology
Keywords:	chronic pain, meditation, mindfulness, mobile applications, pelvic pain, randomized controlled trial

SCHOLARONE™
Manuscripts

I, the Submitting Author has the right to grant and does grant on behalf of all authors of the Work (as defined in the below author licence), an exclusive licence and/or a non-exclusive licence for contributions from authors who are: i) UK Crown employees; ii) where BMJ has agreed a CC-BY licence shall apply, and/or iii) in accordance with the terms applicable for US Federal Government officers or employees acting as part of their official duties; on a worldwide, perpetual, irrevocable, royalty-free basis to BMJ Publishing Group Ltd ("BMJ") its licensees and where the relevant Journal is co-owned by BMJ to the co-owners of the Journal, to publish the Work in this journal and any other BMJ products and to exploit all rights, as set out in our licence.

The Submitting Author accepts and understands that any supply made under these terms is made by BMJ to the Submitting Author unless you are acting as an employee on behalf of your employer or a postgraduate student of an affiliated institution which is paying any applicable article publishing charge ("APC") for Open Access articles. Where the Submitting Author wishes to make the Work available on an Open Access basis (and intends to pay the relevant APC), the terms of reuse of such Open Access shall be governed by a Creative Commons licence – details of these licences and which Creative Commons licence will apply to this Work are set out in our licence referred to above.

Other than as permitted in any relevant BMJ Author's Self Archiving Policies, I confirm this Work has not been accepted for publication elsewhere, is not being considered for publication elsewhere and does not duplicate material already published. I confirm all authors consent to publication of this Work and authorise the granting of this licence.

For peer review only

A smartphone app using psychological approaches for women with chronic pelvic pain presenting to gynaecology clinics (MEMPHIS): a randomised feasibility trial

Gordon Forbes
King's College London, IoPPN, London, UK

Newton, Sian
Centre for Primary Care and Population Health, Queen Mary University of London, United Kingdom

Cantalapiedra, Clara
Department of Obstetrics and Gynaecology, Barts Health NHS Trust, United Kingdom

Birch, Judy
Pelvic Pain Support Network, UK

Dodds, Julie
Women's Health Research Unit, Barts and the London School of Medicine and Dentistry, Queen Mary University of London, United Kingdom

Steed, Liz
Centre for Primary Care and Population Health, Queen Mary University of London, United Kingdom

Rivas, Carol
Social Science Research Unit, University College London, United Kingdom

Khan, Khalid S.
Women's Health Research Unit, Barts and the London School of Medicine and Dentistry, Queen Mary University of London, United Kingdom

Rohricht, Frank
East London NHS Foundation Trust, United Kingdom

Taylor, Stephanie JC
Centre for Primary Care and Population Health, Queen Mary University of London, United Kingdom

Brennan C, Kahan
Pragmatic Clinical Trials Unit, Centre for Primary Care and Population Health, Queen Mary University of London, United Kingdom

Ball, Elizabeth
Department of Obstetrics and Gynaecology, Barts Health NHS Trust, United Kingdom

Corresponding Author: elizabeth.ball9@nhs.net

Objectives

To evaluate the feasibility of a randomised trial of a modified, pre-existing, mindfulness meditation smartphone app for women with chronic pelvic pain.

Design

Three arm randomised feasibility trial.

Setting

Women were recruited at two gynaecology clinics in the UK. Interventions were delivered via smartphone or computer at a location of participants choosing.

Participants

Women were eligible for the study if they were over 18, had been experiencing organic or non-organic chronic pelvic pain for six months or more, and had access to a computer or smartphone. 90 women were randomised.

Interventions

Daily mindfulness meditation delivered by smartphone app, an active control app which delivered muscle relaxation techniques, and usual care without app. Interventions were delivered over 60-days.

Primary and secondary outcome measures

Outcomes included length of recruitment, follow up rates, adherence to the app interventions, and clinical outcomes measured at baseline, two, three and six months.

Results

The target sample size was recruited in 145 days. Adherence to the app interventions was extremely low (mean app use 1.8 days mindfulness meditation group, 7.0 days active control). Fifty-seven (63%) women completed 6-month follow-up, and 75 (83%) women completed at least one post-randomisation follow-up. The 95% confidence intervals for clinical outcomes were consistent with no benefit from the mindfulness meditation app; for example, mean differences in pain acceptance scores at 60 days (higher scores are better) were -2.3 (mindfulness meditation vs. usual care, 95% CI: -6.6, 2.0) and -4.0 (mindfulness meditation vs. active control, 95% CI: -8.1, 0.1).

Conclusions

Despite high recruitment and adequate follow-up rates, demonstrating feasibility, the extremely low adherence suggests a definitive randomised trial of the mindfulness meditation app used in this study is not warranted. Future research should focus on improving patient engagement.

ClinicalTrials.gov registration: NCT02721108, ISRCTN 10925965

Funding: This research was supported by the UK National Institute of Health Research, Research for Patient Benefit programme (RfPB PB-PG-1013-32025).

Strengths and limitations of this study

- This is a randomised feasibility study designed specifically to test whether evaluation of the intervention is viable in a full scale randomised trial
- The trial achieved target recruitment demonstrating feasibility of recruiting patients to trials of apps for women experiencing chronic pelvic pain.
- Measures of adherence to the app interventions were robust and complete as they relied on system generated data
- This trial evaluated only one app provided by a leading developer of mindfulness meditation apps

BACKGROUND

Chronic pelvic pain in women is defined as intermittent or constant pain in the lower abdomen or pelvis for six or more months, and affects more than 24% of women worldwide (1). It has considerable impact on patients' quality of life, including their mental health and their income due to loss of working days and diminished work capacity (2). Chronic pelvic pain may or may not have an identifiable pathology and has both physical and psychological contributors (3). Chronic pelvic pain is difficult to treat but health outcomes can be improved by psychological and lifestyle interventions (4, 5). However these are often not received (6, 7) due to difficulties in access or service shortages.

Systematic reviews of randomised controlled trials evaluating mindfulness meditation have shown benefit in chronic pain conditions (positive effects on depression, quality of life and pain symptoms (8, 9). Mindfulness is a form of meditation where the client attempts to maintain attention on the present moment, for example by focusing their attention on their breathing. Whenever attention wanders from the present moment to thoughts and feelings, the client will simply take notice of them and let them go as attention is returned to the present. There is an emphasis on simply taking notice of whatever the mind happens to wander to and accepting each object without making judgements about it or elaborating on its implications additional meaning or need for action. The client is further encouraged to use the same general approach outside of their formal meditation practice, bringing awareness back to the here and now, whenever they notice a general lack of awareness or that attention has become focused on streams of thoughts and worries (10). So far no randomised controlled trials of mindfulness meditation exist in chronic pelvic pain in women, but results from uncontrolled studies comparing pre- and post-treatment outcomes have suggested there may be a benefit (such as increased ability to control pain, improvements in mental health, emotional well-being, work and family life and social functioning) (11, 12).

Mindfulness meditation can be resource-intensive and typically requires multiple face-to-face visits over a period of weeks or months (13). If effective, delivery of mindfulness meditation via smartphone app to women with chronic pelvic pain could provide a new treatment option for this patient group, requiring a minimal increase in resources for healthcare systems. No studies have evaluated mindfulness mediation via smartphone app for women with chronic pelvic pain. We therefore conducted a randomised feasibility trial to assess the feasibility of a future full scale, multi-centre randomised trial of a

mindfulness meditation intervention delivered by the Headspace smartphone app (Headspace Ltd) for patients with chronic pelvic pain.

The primary objective of the study was to assess the feasibility of implementing a randomised trial of a mindfulness meditation intervention delivered by a smartphone app for women with chronic pelvic pain. Specifically, we assessed feasibility of recruitment, levels of adherence to the intervention, and estimated parameters required for the sample size calculation for a full trial. Secondary objectives were to measure the clinical outcomes that may be used in a future full scale trial and make estimates for the effect of the intervention. We examined a variety of clinical outcomes assessing pain acceptance and self efficacy, pain related disability, mental health, mindfulness, and sexual health, and quality of life. No primary outcome was specified because this was a feasibility study (14), however it was anticipated that chronic pain acceptance would be the primary outcome for any future study assessing effectiveness. Pain acceptance was chosen by the study group with input from pain patients and clinicians because has been shown to be a meaningful clinical outcome that was improved by mindfulness mediation in other pain conditions (8). This article reports quantitative findings; qualitative findings will be published separately (15).

METHODS

Study design and participants

This three arm parallel group randomised feasibility trial was conducted at two gynecology clinics within Barts Health NHS trust. Eligible patients were aged 18 years or over, had been experiencing chronic pelvic pain with or without identifiable pathology (i.e. organic or non-organic chronic pelvic pain) for six months or more, and understood simple English. Patients were excluded from the trial if they had no access to a personal computer or smartphone, or were current users of the publicly available Headspace app. Patients were recruited via pelvic pain or endometriosis clinics at participating sites as well as at other routine appointments. Prior to randomisation, all participants were provided with a patient information sheet and provided written informed consent. The study protocol has been published (16) and the final version is given in Appendix 1.

Interventions

Full details of the interventions are available in the published protocol (16). Patients were randomised to receive mindfulness meditation, an active control, or usual care only. All participants received usual care, which included watch and wait, medication and/or surgery.

Women in the mindfulness meditation group received access to a 60-day progressive mindfulness meditation course delivered via the Headspace app. The intervention consisted of daily, audio guided, mindfulness meditation sessions. The first 10 days of the course taught basics of mindfulness meditation. Following this, participants were able to access the module on meditation which was targeted at chronic pain. This module was specifically designed for the MEMPHIS trial. Session length was 10 minutes for the first 10 days, 15 minutes up to day 20 and 20 minutes up to day 60.

The active control group received access to a series of muscle relaxation sessions. These sessions were identical every day, except that their duration increased to mirror the increasing duration of the meditation content being listened to by the intervention group.

Women in the mindfulness mediation group and active control group were given instructions on how to install the app. No further face-to-face induction was given on how to carry out the techniques taught in the apps. To maintain blinding between the mindfulness meditation group and active control, both groups accessed their intervention via the same app, and received instructions for the same duration, delivered by the same narrator. Only the content of the instructions differed.

We chose to evaluate an existing commercial app teaching mindfulness by guided meditation (Headspace Ltd) as this approach was expected to save time and money compared to designing a new app from scratch. The Headspace app was adapted for use by chronic pelvic pain patients by augmenting the existing app with a novel module on chronic pain, which could be accessed after completing ten days of basic training in mindfulness meditation.

Randomisation and blinding

Women were randomly allocated 1:1:1 to the active intervention app, active control app, or treatment as usual using random permuted blocks (block size 27, 30, 33) without stratification using a centralised web based service with allocation concealment. The randomisation list was generated using the Pragmatic Clinical Trials Unit’s randomisation system using a random number generator. Following

randomisation, participants, recruiting staff, and researchers conducting follow-up interviews were not blinded to whether allocation was to the treatment as usual group or to one of the app groups (mindfulness meditation or active control); however, for allocation to an app group they were blinded to which specific app group this was (mindfulness meditation or active control). The trial statisticians remained blinded to allocation until the statistical analysis plan had been signed off, all data collection was completed, and the dataset was finalised.

Data collection

Data on patient adherence to the app was collected by Headspace Ltd. Data collection was performed automatically by the app and recorded every time a participant completed more than 90% of a session with the app. No data was collected on sessions that were less than 90% complete. Headspace provided the trial team with a list of codes, which were linked to the randomisation system, and given to trial participants to access the app. At the end of the trial, data on completed sessions were transferred via a secure file transfer protocol (SFTP) from Headspace to the trial team. No data which could identify participants were included in this transfer. Clinical outcome measures were collected in person at baseline prior to randomisation and via postal questionnaires or telephone at 2, 3 and 6 months post-randomisation. App satisfaction and usability questionnaires were collected via postal questionnaires or telephone. Shopping vouchers (£5), text reminders and phone calls were introduced to improve follow up rates three months after recruitment began: shopping vouchers were sent in the post with each follow up questionnaire; participants were sent text reminders and up to three attempts were made to contact participants by phone if questionnaire responses were not received within 10 days.

Outcomes

Feasibility outcomes were: time to recruit 90 patients to the study; standard deviation of chronic pain acceptance questionnaire (CPAQ-8) (17) (as this was likely to be the primary outcome for a future full-scale trial); proportion of participants completing a follow-up questionnaire at 6 months post randomisation; and proportion of participants not returning a follow up questionnaire by post but who answered a telephone questionnaire at 6 months. Standard deviation of CPAQ was included as an outcome as this information would be required for the sample size calculation for a full trial. App usability was measured using the system usability scale (18) and a purpose made, non-validated questionnaire developed from PPI group discussion. Adherence to the app interventions was measured in the following ways:

For peer review only - http://bmjopen.bmj.com/site/about/guidelines.xhtml

(a) number of days a patient has used the app within 60 days of randomisation;

(b) Number of weeks a patient has used the app on three or more days within the first eight weeks from randomisation;

(c) whether the patient has used the app on at least 22 days within 60 days of randomisation (binary outcome);

(d) whether the patient has used the app on three or more days in 6 or more weeks within the first eight weeks of randomisation (binary outcome);

(e) whether the patient has used the app on 22 or more days within the first 60 days from randomisation and used the app on three or more days in 6 or more weeks within the first eight weeks from randomisation (binary outcome).

Measures of app use were chosen following discussion within the trial management group and trial steering group to give a complete picture of how participants were using the app. App use was defined as having completed at least 90% of a session. This definition of app use was changed after the trial started recruiting but before any data were analysed due to a change in the way data on app use were collected by Headspace. The original definition of app use was for patients to have completed at least 50% of a session.

The following clinical outcomes were measured at baseline, 60 days, 3 months and 6 months post randomisation:

a) Pain acceptance score (measured by the chronic pain acceptance questionnaire [CPAQ-8]) (17);

b) pain related disability (chronic pain grade [CPG] – disability subscale) (19);

c) quality of life subscales (measured by the RAND short form 36 health survey [SF-36]): social functioning subscale, pain functioning subscale, and general health subscale (20);

d) the depression and anxiety subscales of the Hospital Anxiety and Depression Scale [HADS] (21)

e) mindfulness (cognitive and mindfulness - revised scale [CAMS-R]) (22);

f) self-efficacy (pain self-efficacy questionnaire [PSEQ]) (23);

g) sexual health amongst sexually active participants (sexual health outcomes in women questionnaire [SHOW-Q]) (24);

h) sexual health pelvic problem interference score (SHOW-Q pelvic problem subscale) (24);

i) an individualised outcome (Measure yourself medical outcome profile [MYMOP]) (25).

Statistical analysis

A sample size of 90 participants was chosen as it would provide a precise estimate for the standard deviation of the primary clinical outcome (likely to be pain acceptance) (26, 27), which could be used to inform the sample size calculation of a subsequent full-scale trial. This sample size is also adequate to provide estimates of proportions for binary outcomes (27).

Feasibility outcomes and baseline data were summarised using descriptive statistics. Clinical outcomes were analysed using a linear mixed-effects models with outcome measurement (at three follow-up time points) as the dependent variable and an unstructured correlation matrix for the residuals (28). The model included fixed effects for time, treatment arm, time-by-treatment interactions and baseline measure of the outcome (29). Analysis was by intention-to-treat; all patients with an observed outcome for at least one of the three follow-up time points were included in the analysis (30), and were analysed according to their randomised group. Missing baseline clinical measures were handled using mean imputation (31). See appendix 2 for a full statistical analysis plan.

Patient and Public Involvement (PPI).

The study design and intervention was discussed with a PPI group formed of 15 women who attended the recruiting clinics. A basic version of the app by Headspace Ltd. was made available to the group for testing. A patient, who bought their own experience and acted as a representative for a charity supporting those with CPP, sat on the trial management group which oversaw the conduct of the trial.

Ethical Approval

Ethics approval was granted by Camden and Kings Cross Research Ethics Committee on 1st February 2016.

RESULTS

Feasibility Outcomes

Ninety women were recruited to the trial in 145 days between May 2016 and September 2016. A CONSORT diagram is shown in figure 1 and baseline characteristics are shown in table 1, with additional

baseline data given in appendix 3, tables 1 and 2. Follow up at 6 months was 68% in the mindfulness meditation group, 53% in the active control group and 69% in the usual care group. Follow up rates by method of follow up (phone or questionnaire), at different time points, and a comparison of baseline characteristics by questionnaire completion are given in appendix 3, tables 3-5 and appendix 3 figure 1. The standard deviation for CPAQ can be found in appendix 3, table 6. Unintentional unblinding of treatment for either participants or researchers collecting data was rare (Appendix 3, table 7).

App use was low in both groups, but was higher in the active control group than the intervention group (app used on mean 1.8 days intervention vs 7.0 active control – table 2). Few women used the app on more than 22 days within 60 days of randomisation (0 intervention vs 2 active control). Adherence to the app intervention was low or entirely absent across all other measures of app use (table 2). Daily app use within 60 days of randomisation is summarised in figure 2. The results from the app usability questionnaire are shown in appendix 3, tables 8 and 9.

Clinical outcomes

We included 27 (87%) women from the intervention group, 23 (77%) from the active control group and 25 (86%) from the usual care group in the analysis of pain acceptance score. The 95% confidence intervals for CPAQ (figure 3) rule out any strong benefit of the intervention compared to either the active control group, or usual care group at any time point (higher CPAQ corresponds to better outcomes). The results for other clinical outcomes are consistent with no effect of the intervention (full results of clinical outcomes are shown in appendix 3 tables 10-13 & figure 2).

DISCUSSION

This trial shows that it is feasible to recruit women to a trial of a mindfulness meditation app. Follow up rates were adequate and including data across all time points meant that a relatively a high proportion of participants could be included in the analysis. This study provides estimates to inform sample size calculations for future research.

Most participants either did not complete any sessions on the apps or used them extremely infrequently. The analyses of clinical outcomes are consistent with no differences in health outcome between the three study arms. For pain acceptance, which was considered to be a likely outcome for a future effectiveness trial, our results suggest a meaningful effect of the mindfulness meditation app, delivered

as it is in this trial, is unlikely. An effective intervention requires both engagement from those receiving it and the ability to change the targeted clinical outcome (32). As engagement with the mindfulness meditation app evaluated in this study was very low it is unlikely it would be an effective intervention in the routine clinical setting for women with chronic pelvic pain, unless delivered as part of an intervention which significantly enhanced rates of engagement.

In addition to the work described in this paper we carried out in-depth qualitative interviews in order to examine the reasons for low levels of user engagement. Suggestions are given for improving the intervention such as co-development, an approach to intervention that involves the users in the design of the intervention The findings are published in the companion paper describing the qualitative arm of this study (15). The length of the intervention in this study (60 days) may also have been a barrier to participation and future work may want to explore different treatment lengths for remote based mindfulness interventions.

An important lesson from this trial for future researchers was that intermediate follow up points allowed for more participants to be included in the analysis of clinical outcomes than were followed up at the final time point. This demonstrates that utilising intermediate follow up time points may help to minimise potential bias from missing data in trials.

Strengths of this study include randomisation of participants, which eliminates bias inherent in other designs such as before-after studies. We also blinded patients, recruiters, and data collectors to which app group patients were allocated to. We used system generated app data and therefore were able to obtain complete adherence data for all participants. One drawback to this method of data collection was that sessions of the app were only recorded as being complete if a participant listened to 90% of the session. This means this study may have underestimated app use if participants were only partially completing sessions. Levels of app use were so low however that this is unlikely to have had a material impact on the study's results. A second limitation is that recruitment was limited to two hospitals in one area of London, this may limit the generalisability of the results to settings where there is higher engagement with smartphone apps.

In conclusion, this study had high recruitment and adequate follow-up rates, demonstrating that it is feasible to conduct randomised trials in this patient population. However, due to extremely low adherence, further randomised trials to evaluate the benefit of the Headspace mindfulness meditation app for women with chronic pelvic pain are not warranted, unless additional steps to improve engagement with the app are included in the intervention. Further discussion of reasons for low engagement and what could be done to improve engagement may be found in the qualitative part of this study (15).

Data availability statements
Anonymised participant data is available upon reasonable request. Please contact pctu-data-sharing@qmul.ac.uk with any data sharing requests.

Competing interests
None declared

Author contributions
EB conceived of the research and lead the study. GF conducted the statistical analysis under supervision from BCK. GF drafted the manuscript. GF, SN, CC, JB, JD, LS, CR, KSK, FR, SJCT, BCK, and EB contributed to the design and conduct of the study, and discussed and reviewed the final manuscript.

References

1. Latthe P, Latthe M, Say L, Gulmezoglu M, Khan KS. WHO systematic review of prevalence of chronic pelvic pain: a neglected reproductive health morbidity. BMC Public Health. 2006;6:177.
2. Zondervan KT, Yudkin PL, Vessey MP, Jenkinson CP, Dawes MG, Barlow DH, et al. Chronic pelvic pain in the community - Symptoms, investigations, and diagnoses. Am J Obstet Gynecol. 2001;184(6):1149-55.
3. Daniels JP, Khan KS. Chronic pelvic pain in women. BMJ. 2010;341:c4834.
4. Moore SJ KS. Green Top Guideline No 41: the initial management of Chronic Pelvic Pain. In: Gynaecologists TrcoOa, editor. 2012.
5. Peters AA, van Dorst E, Jellis B, van Zuuren E, Hermans J, Trimbos JB. A randomized clinical trial to compare two different approaches in women with chronic pelvic pain. Obstet Gynecol. 1991;77(5):740-4.
6. Romao APMS, Gorayeb R, Romao GS, Poli-Neto OB, dos Reis FJC, Rosa-e-Silva JC, et al. High levels of anxiety and depression have a negative effect on quality of life of women with chronic pelvic pain. Int J Clin Pract. 2009;63(5):707-11.
7. Stones RW, Price C. Health services for women with chronic pelvic pain. J R Soc Med. 2002;95(11):531-5.
8. Ball EF, Sharizan ENSM, Franklin G, Rogozinska E. Does mindfulness meditation improve chronic pain? A systematic review. Curr Opin Obstet Gynecol. 2017;29(6):359-66.
9. Hilton L, Hempel S, Ewing BA, Apaydin E, Xenakis L, Newberry S, et al. Mindfulness Meditation for Chronic Pain: Systematic Review and Meta-analysis. Ann Behav Med. 2017;51(2):199-213.
10. Bishop SR, Lau M, Shapiro S, Carlson L, Anderson ND, Carmody J, et al. Mindfulness: A proposed operational definition. Clinical Psychology-Science and Practice. 2004;11(3):230-41.
11. Kold M, Hansen T, Vedsted-Hansen H, Forman A. Mindfulness-based psychological intervention for coping with pain in endometriosis. Nordic Psychology. 2012;64(1):2-16.
12. Fox SD, Flynn E, Allen RH. Mindfulness Meditation for Women with Chronic Pelvic Pain A Pilot Study. J Reprod Med. 2011;56(3-4):158-62.
13. Salmon P, Sephton S, Weissbecker I, Hoover K, Ulmer C, Studts JL. Mindfulness meditation in clinical practice. Cogn Behav Pract. 2004;11(4):434-46.
14. Eldridge SM, Chan CL, Campbell MJ, Bond CM, Hopewell S, Thabane L, et al. CONSORT 2010 statement: extension to randomised pilot and feasibility trials. Pilot Feasibility Stud. 2016;2:64.
15. Ball E, Newton S, Kahan BC, Forbes G, Rohricht F, Steed E, et al. Using a Mindfulness App for women with chronic pelvic pain: Qualitative data of user experience and lessons learnt Submitted. 2018.
16. Ball E, Newton S, Kahan BC, Forbes G, Wright N, Calvete CC, et al. Smartphone App Using Mindfulness Meditation for Women With Chronic Pelvic Pain (MEMPHIS): Protocol for a Randomized Feasibility Trial. Jmir Research Protocols. 2018;7(1).
17. Fish RA, McGuire B, Hogan M, Morrison TG, Stewart I. Validation of the Chronic Pain Acceptance Questionnaire (CPAQ) in an Internet sample and development and preliminary validation of the CPAQ-8. Pain. 2010;149(3):435-43.
18. Brooke J. SUS-A quick and dirty usability scale. Usability evaluation in industry. 1996;189(194):4-7.
19. Von Korff M, Ormel J, Keefe FJ, Dworkin SF. Grading the severity of chronic pain. Pain. 1992;50(2):133-49.
20. Ware JE, Sherbourne CD. The Mos 36-Item Short-Form Health Survey (Sf-36) .1. Conceptual-Framework and Item Selection. Med Care. 1992;30(6):473-83.

21. Zigmond AS, Snaith RP. The Hospital Anxiety and Depression Scale. Acta Psychiatr Scand. 1983;67(6):361-70.
22. Feldman G, Hayes A, Kumar S, Greeson J, Laurenceau JP. Mindfulness and emotion regulation: The development and initial validation of the Cognitive and Affective Mindfulness Scale-Revised (CAMS-R). Journal of Psychopathology and Behavioral Assessment. 2007;29(3):177-90.
23. Nicholas MK. The pain self-efficacy questionnaire: Taking pain into account. European Journal of Pain. 2007;11(2):153-63.
24. Learman LA, Huang AJ, Nakagawa S, Gregorich SE, Kuppermann M. Development and validation of a sexual functioning measure for use in diverse women's health outcome studies. Am J Obstet Gynecol. 2008;198(6).
25. Paterson C. Measuring outcomes in primary care: A patient generated measure, MYMOP, compared with the SF-36 health survey. Br Med J. 1996;312(7037):1016-20.
26. Lancaster GA, Dodd S, Williamson PR. Design and analysis of pilot studies: recommendations for good practice. J Eval Clin Pract. 2004;10(2):307-12.
27. Teare MD, Dimairo M, Shephard N, Hayman A, Whitehead A, Walters SJ. Sample size requirements to estimate key design parameters from external pilot randomised controlled trials: a simulation study. Trials. 2014;15.
28. Rabe-Hesketh S, Skrondal A. Multilevel and longitudinal modeling using Stata. 3rd ed. College Station, Tex.: Stata Press Publication; 2012.
29. Kahan BC, Jairath V, Dore CJ, Morris TP. The risks and rewards of covariate adjustment in randomized trials: an assessment of 12 outcomes from 8 studies. Trials. 2014;15.
30. White IR, Horton NJ, Carpenter J, Pocock SJ. Strategy for intention to treat analysis in randomised trials with missing outcome data. Bmj-British Medical Journal. 2011;342.
31. White IR, Thompson SG. Adjusting for partially missing baseline measurements in randomized trials. Stat Med. 2005;24(7):993-1007.
32. Barrera M, Castro FG. A heuristic framework for the cultural adaptation of interventions. Clinical Psychology-Science and Practice. 2006;13(4):311-6.

Figures

Figure 1: CONSORT diagram

Figure 2: Daily app use (defined as completing ≥90% of a session) within 60 days of randomisation in the intervention and active control groups.

Figure 3: Mean (95% CI) chronic pain acceptance score (CPAQ) and estimated treatment effect (95% CI) at each follow-up time point. (CPAQ). Higher scores indicate better health outcomes.

Tables

Table 1: Baseline demographics and medical history. Figures are mean (SD) unless stated otherwise.

	Summary measure		
	**Intervention (N=31)**	**Active control (N=30)**	**Usual care (N=29)**
**Demographics**			
Age (Years)	34.8 (9.9)	35.7 (5.7)	35.0 (8.6)
Body mass index (kg/m^2)	28.7 (7.0)	26.2 (5.5)	26.6 (6.3)
Living arrangements - no. (%)			
Alone	1 (3.3)	2 (7.4)	3 (11.1)
With others	29 (96.7)	25 (92.6)	24 (88.9)
Employment status - no. (%)			
Employed	19 (63.3)	18 (66.7)	19 (67.9)
Unemployed and looking for work	2 (6.7)	0 (0.0)	1 (3.6)
At school or in full time education	2 (6.7)	1 (3.7)	4 (14.3)
Unable to work due to long term sickness	4 (13.3)	5 (18.5)	1 (3.6)
Looking after your home/family	3 (10.0)	3 (11.1)	2 (7.1)
Retired from paid work	0 (0.0)	0 (0.0)	1 (3.6)
Age left full time education - no. (%)			
Age 12 or less	1 (3.3)	1 (3.8)	1 (3.6)
Age 13 to 16	9 (30.0)	4 (15.4)	3 (10.7)
Age 17 to 19	6 (20.0)	5 (19.2)	3 (10.7)
Age 20 or over	11 (36.7)	15 (57.7)	16 (57.1)
Still in education	3 (10.0)	1 (3.8)	5 (17.9)
Ethnic group - no. (%)			
White	10 (35.7)	10 (43.5)	15 (53.6)
Black	6 (21.4)	4 (17.4)	3 (10.7)
Cetral Asian	1 (3.6)	1 (4.3)	0 (0.0)
Middle Eastern	0 (0.0)	0 (0.0)	1 (3.6)
Southern Asian	8 (28.6)	7 (30.4)	3 (10.7)
Mixed	0 (0.0)	0 (0.0)	2 (7.1)
Other ethnic group	2 (7.1)	1 (4.3)	3 (10.7)
Do not wish to say	1 (3.6)	0 (0.0)	1 (3.6)
Smoker - no. (%)			
Yes	8 (27.6)	3 (12.5)	6 (21.4)
No	21 (72.4)	21 (87.5)	22 (78.6)
If yes, number of cigarettes per week	23.9 (20.3)	40.0 (20.0)	47.6 (35.6)
Drink alcohol - no. (%)			
Yes	10 (34.5)	9 (36.0)	15 (55.6)
No	19 (65.5)	16 (64.0)	12 (44.4)

For peer review only - http://bmjopen.bmj.com/site/about/guidelines.xhtml

If yes, number of units per week	5.7 (5.3)	8.3 (4.7)	7.7 (7.2)
**Baseline medical history**			
Duration of pain - no. (%)			
0 to 6 months	2 (6.7)	0 (0.0)	0 (0.0)
7 to 12 months	2 (6.7)	4 (14.8)	2 (7.1)
1 to 2 years	3 (10.0)	5 (18.5)	5 (17.9)
3 to 5 years	13 (43.3)	7 (25.9)	6 (21.4)
6 to 10 years	4 (13.3)	4 (14.8)	3 (10.7)
More than 10 years	6 (20.0)	7 (25.9)	12 (42.9)
Pain over the past week (scale of 0 to 10)	6.9 (2.3)	5.8 (2.8)	6.8 (2.3)

Table 2: App use

Figures are mean (SD) unless stated otherwise.

	**Intervention (N=31)**	**Active control (N=28)***
Number of days a patient has used the app (within 60 days of randomisation)	1.8 (4.3)	7.0 (10.5)
Number of weeks a patient has used the app on three or more days (within the first eight weeks from randomisation)	0.3 (0.8)	1.0 (1.6)
Used the app on 22 or more days within the first 60 days from randomisation - no. (%)	0 (0.0)	2 (7.1)
Used the app on three or more days in 6 or more weeks (within the first eight weeks from randomisation) - no. (%)	0 (0.0)	0 (0.0)
Used the app on 22 or more days within the first 60 days AND used the app on three or more days in 6 or more weeks within the first eight weeks from randomisation - no. (%)	0 (0.0)	0 (0.0)

*2 participants in the active control group withdrew permission for their data to be used and are excluded from this analysis.

Appendix 1: Final study protocol

Appendix 2: Statistical analysis plan

Appendix 3: Supplementary tables

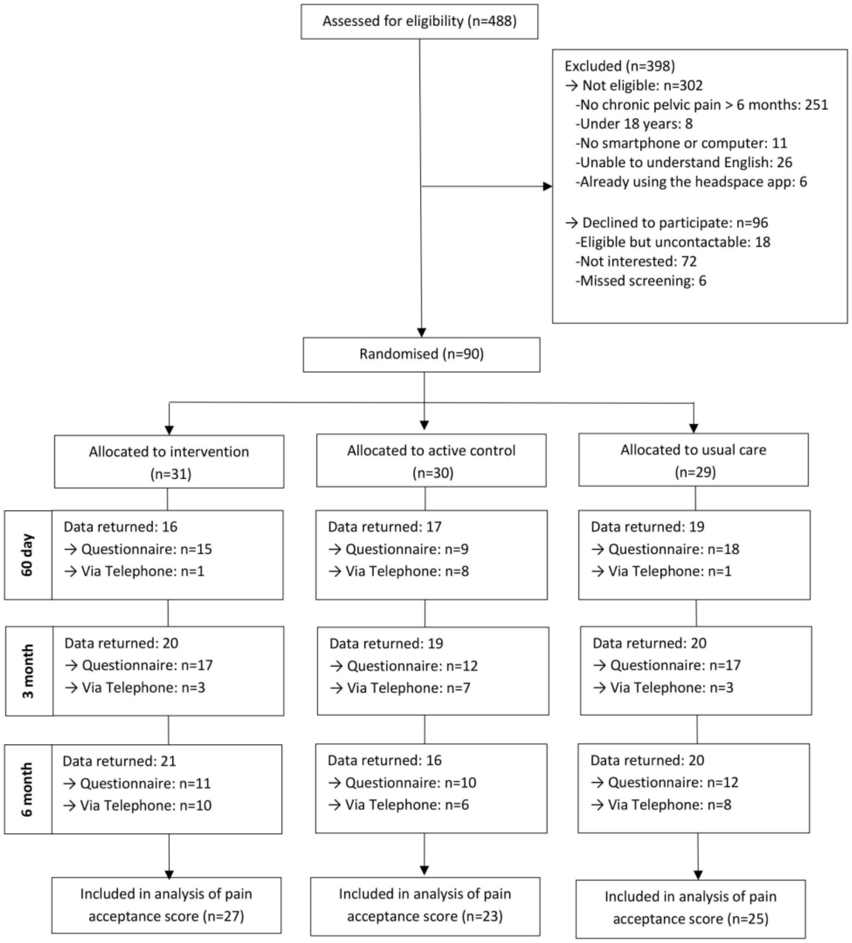

Figure 1: CONSORT diagram

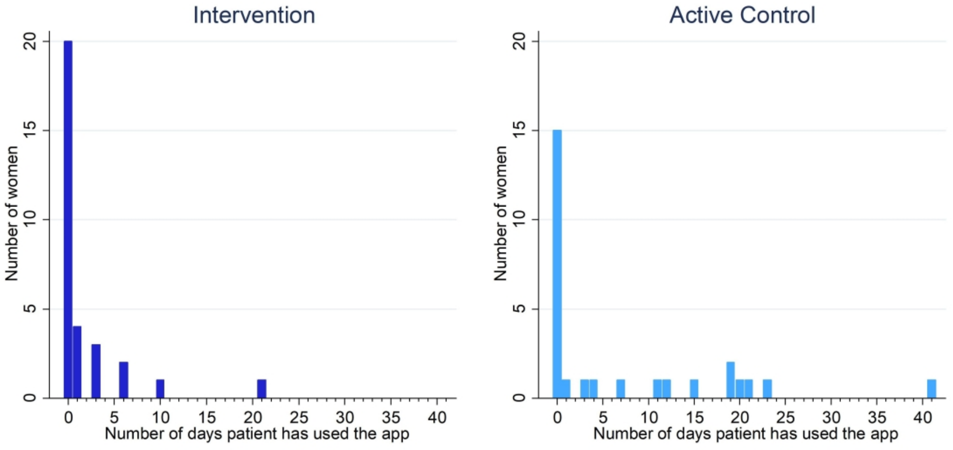

Figure 2: Daily app use (defined as completing >90% of a session) within 60 days of randomisation in the intervention and active control groups.

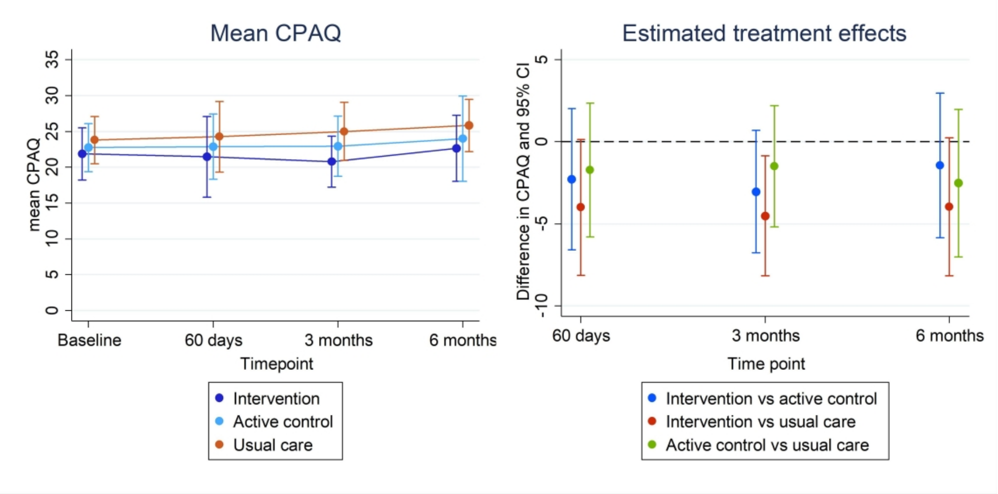

Figure 3: Mean (95% CI) chronic pain acceptance score (CPAQ) and estimated treatment effect (95% CI) at each follow-up time point. (CPAQ). Higher scores indicate better health outcomes.

A smartphone app using psychological approaches for women with chronic pelvic pain (MEMPHIS): a randomised feasibility trial

**Short Title/Acronym**	MEMPHIS
**Sponsor**	Barts Health NHS Trust<br><br>Contact person of the above sponsor organisations is:<br><br>Dr Sally Burtles<br>Director of Research Services & Business Development<br>Joint Research Management Office<br>5 Walden Street<br>London<br>E1 2EF<br>Phone: 020 7882 7260<br>Email: sponsorsrep@bartshealth.nhs.uk
**REC Reference**	15/LO/1967
**Chief Investigator**	**Miss Elizabeth Ball**<br>Women and Children Department<br>Royal London Hospital<br>Barts Health NHS Trust<br>elizabeth.ball@bartshealth.nhs.uk<br>eball69@gmail.com

For peer review only - http://bmjopen.bmj.com/site/about/guidelines.xhtml

Pragmatic Clinical Trials Unit

Blizard Institute, Centre for Primary Care and Public Health

Bart's and The London School Medicine and Dentistry

Queen Mary University of London

Yvonne Carter Building, 58 Turner St

Whitechapel, E1 2AB

PCTU Senior Trial statistician:

Mr Brennan Kahan

+44 207 882 2483

b.kahan@qmul.ac.uk

PCTU Junior Trial Statistician

Mr Neil Wright

+44 207 882 6564

n.wright@qmul.ac.uk

PCTU Data Manager:

Mr Mike Waring

+44 207 882 6914

m.r.waring@qmul.ac.uk

PCTU QA Manager:

Ms Anitha Manivannan

+44 207 882 6312

a.manivannan@qmul.ac.uk

Protocol development

Miss Elizabeth Ball

Chief Investigator

For peer review only - http://bmjopen.bmj.com/site/about/guidelines.xhtml

Barts and The London School of Medicine and Dentistry

elizabeth.ball@bartshealth.nhs.uk

eball69@gmail.com

Dr Julie Dodds

Senior Research Manager

j.dodds@qmul.ac.uk

Mr Brennan Kahan

PCTU Senior Trial Statistician

b.kahan@qmul.ac.uk

Mr Neil Wright

PCTU Junior Trial Statistician

n.wright@qmul.ac.uk

Dr Carol Rivas

Senior Research Fellow

C.A.Rivas@soton.ac.uk

Miss Ewelina Rogozinska

Co-investigator

e.a.rogozinska@qmul.ac.uk

PCTU QA Manager:

Ms Anitha Manivannan

+44 207 882 6312

a.manivannan@qmul.ac.uk

For peer review only - http://bmjopen.bmj.com/site/about/guidelines.xhtml

Barts and The London
School of Medicine and Dentistry

CONTENTS PAGE

1.GLOSSARY OF TERMS AND ABBREVIATIONS 7
2.SIGNATURE PAGES 8
3.SUMMARY/SYNOPSIS 9
4.INTRODUCTION 11
4.1. Background 11
4.2. Effect of mindfulness meditation in chronic pain patients 12
4.3. On-going studies 12
4.4. Implications for the further development of clinical or public health practice 13
4.5. Potential impact on local policy making and improvement in service delivery 14
5.TRIAL OBJECTIVES 14
5.1. Aims and Objectives 14
5.2. Feasibility outcomes 15
5.3. App satisfaction questionnaires 16
5.4. Clinical Outcomes 17
6.METHODOLOGY 18
6.1. Inclusion Criteria 18
6.2. Exclusion Criteria 18
6.3. Study Design 19
6.4. Study Scheme Diagram 20
7. STUDY PROCEDURES 20
7.1.Informed Consent Procedures 20
7.2. Screening and enrolment 21
7.3. Randomisation Procedures 22
7.4 Blinding 22
7.5. Planned interventions 23
7.6. Concomitant Medications 24
7.7. Reasons for non progression to full trial 24

For peer review only - http://bmjopen.bmj.com/site/about/guidelines.xhtml

Barts and The London
School of Medicine and Dentistry

7.8. Key risks to delivering this research and contingencies 24
7.9. Procedure for Collecting Data 25
7.10. Including Case Report Forms (CRFs) and storage 25
7.11. Follow-up Procedures 25
7.12. Subject withdrawal (including data collection / retention for withdrawn participants) 25
7.13. Continued app use after trial period and app use by treatment as usual group 26
7.14. Schedule of Assessment 27
7.15. Criteria for Early Termination of the study 28
7.16. End of Study Definition 28
8. STATISTICAL CONSIDERATIONS 28
8.1. Sample Size 28
8.2. Statistical Analysis 28
9. ETHICS 30
10. SAFETY CONSIDERATIONS 31
11. DATA HANDLING AND RECORD KEEPING 31
11.1 Confidentiality 31
11.2. Required Study Documents 32
11.3. Record Retention and Archiving 33
12. PRODUCTS, DEVICES, TECHNIQUES AND TOOLS 33
12.1. Devices 33
12.2. Techniques and intervention 34
13. SAFETY REPORTING 35
13.1. Adverse Events (AE) 35
13.2. Serious Adverse Event (SAE) 35
13.3. Urgent Safety Measures 36
13.4. Annual Safety Reporting 36
13.5. Overview of the Safety Reporting Responsibilities 37
14. MONITORING & AUDITING 37
14.1. Auditing 37
14.2. Summary Monitoring Plan 37

For peer review only - http://bmjopen.bmj.com/site/about/guidelines.xhtml

14.3. Compliance 38
14.4. Non-Compliance 38
15. TRIAL COMMITTEES 39
15.1. Trial Steering Committee (TSC) 38
15.2. Trial Management Group (TMG) 39
15.3. Data Monitoring Committee (DMC) 39
16. FINANCE AND FUNDING 39
17. INDEMNITY 39
18. DISSEMINATION OF RESEARCH FINDINGS 40
REFERENCES 42
APPENDICES 46

For peer review only - http://bmjopen.bmj.com/site/about/guidelines.xhtml

Barts and The London
School of Medicine and Dentistry

1. GLOSSARY of Terms and Abbreviations

AE	Adverse Event
CI	Chief Investigator
CPP	Chronic Pelvic Pain
CRF	Case Report Form
DMC	Data Monitoring Committee
GCP	Good Clinical Practice
HCP	Health Care Professional
ICF	Informed Consent Form
JRMO	Joint Research Management Office
KTN	Katherine Twining Network
MHRA	Medicines and Healthcare products Regulatory Agency
NHS REC	National Health Service Research Ethics Committee
NHS R&D	National Health Service Research & Development
NPT	Normalization Process Theory
Participant	An individual who takes part in a clinical trial
PCTU	Pragmatic Clinical Trial Unit
PI	Principal Investigator
PIS	Participant Information Sheet
PSM	Patient Self-Management
QOL	Quality Of Life
QC	Quality Control
RCT	Randomised Controlled Trial
REC	Research Ethics Committee
RfPB	Research for Patients Benefit
SAE	Serious Adverse Event
SOP	Standard Operating Procedure
SUS	System Usability Scale
TAU	Treatment As Usual
TMG	Trial Management Group
TSC	Trial Steering Committee

For peer review only - http://bmjopen.bmj.com/site/about/guidelines.xhtml

2. SIGNATURE PAGES

Chief Investigator/Principal Investigator Agreement

The clinical study as detailed within this research protocol **(Version V8.0, dated 22 12 2016)**, or any subsequent amendments will be conducted in accordance with the Research Governance Framework for Health & Social Care (2005), the World Medical Association Declaration of Helsinki (1996) and the current applicable regulatory requirements and any subsequent amendments of the appropriate regulations.

Chief Investigator Name: Miss Elizabeth Ball
Chief Investigator Site: Barts and the London School of Medicine and Dentistry, Queen Mary University of London

Signature and Date: **22.12.2016**

Statistician Agreement

The clinical study as detailed within this research protocol **(Version V8.0, 22 12 2016)**, or any subsequent amendments will be conducted in accordance with the Research Governance Framework for Health & Social Care (2005), the World Medical Association Declaration of Helsinki (1996) and the current applicable regulatory requirements and any subsequent amendments of the appropriate regulations.

Statistician Name: Mr Brennan Kahan
Statistician Site: Pragmatic Clinical Trials Unit, Queen Mary University of London

Signature and Date: **22.12.2016**

For peer review only - http://bmjopen.bmj.com/site/about/guidelines.xhtml

Barts and The London
School of Medicine and Dentistry

3. SUMMARY/SYNOPSIS

**Short Title**	MEMPHIS
**Methodology**	A randomised feasibility trial
**Research Sites**	This trial will be conducted at the Royal London and Whipps Cross Hospitals
**Objectives/Aims**	The overall aim is to assess the feasibility of implementing a trial of a mindfulness meditation intervention delivered by a mobile phone app for patients with chronic pelvic pain (CPP). The primary objectives are:<br>1) To provide feasibility data for a large multicentre RCT aimed at rigorously testing mindfulness meditation in CPP<br>2) To determine whether this app can be seamlessly integrated into clinical practice, especially CPP pathways
**Number of Participants/Patients**	90 women with CPP will be recruited and each randomised into one of the three trial groups (meditation app, progressive muscle relaxation or no app).
**Main Inclusion Criteria**	To be eligible for the MEMPHIS study, the women must:<br>● Be age 18 or over<br>● Have either organic or non-organic chronic pelvic pain lasting for 6 months or more<br>● Have access to a personal computer or smartphone.<br>● Understand simple spoken English
**Statistical Methodology and Analysis (if applicable)**	Feasibility outcomes will be summarised using descriptive statistics. Clinical outcomes will be analysed using linear mixed-effects models, and results will be presented as a difference in means and a 95% confidence interval.

For peer review only - http://bmjopen.bmj.com/site/about/guidelines.xhtml

	Usability and integration into clinical practice will be explored in focus groups or via telephone interviews with participants.<br>Some participants will be asked to elaborate about app satisfaction and also on clinical outcomes. Results will be analysed using content analysis including both thematic and text word analysis.
**Proposed Start Date**	November 2015
**Proposed End Date**	August 2017
**Study Duration**	22 months

For peer review only - http://bmjopen.bmj.com/site/about/guidelines.xhtml

Barts and The London
School of Medicine and Dentistry

4. INTRODUCTION

4.1. Background

Chronic pelvic pain (CPP) is defined as intermittent or constant pain in the lower abdomen or pelvis of a woman for at least 6 months, not exclusively associated with menstruation, intercourse and not associated with pregnancy [1].

It affects up to 24% women worldwide [2], accounts for 20% of UK gynaecological clinic referrals [3], and has a considerable impact on patients' quality of life and their income. CPP costs the NHS € 3.3bn per year [4]. Despite costly interventions, CPP is often resistant to surgical and medical treatment. Multifactorial psychological and somatic causes require a multidimensional approach, which is not routinely offered in gynaecology clinics [5]. Evidence from randomised controlled trials (RCTs) suggests that psychological interventions may be superior to primary surgery [6]. Although psychological treatment is provided across the NHS, mostly in the context of the primary care programme *Improving Access to Psychological Therapies* there are problems with capacity, waiting times, and the overall number of patients being able to access services. Alternatively, patient self-management (PSM) is now recognised as a tool empowering patients to cope better with their condition [7]. Mindfulness meditation is a potentially valuable PSM tool in CPP. We conducted a systematic search of literature (07/2013, updated 12/2013) and found no RCTs of mindfulness meditation in CPP. However, we identified two small, non-randomised pilot trials investigating the effect of mindfulness meditation on pain (one in women with CPP and one in women with endometriosis) both of which showed promising results [8,9].

Because we identified no RCTs on mindfulness meditation in CPP in our systematic review, we included other chronic pain conditions which may have a similar patho-mechanism to pelvic pain, such as back pain, headache, fibromyalgia and diabetic neuropathy. We assume that any benefits of mindfulness meditation in these conditions may also be seen in CPP.

We found previous systematic reviews in these conditions had a number of limitations, such as not reporting effect sizes [10-12].

For peer review only - http://bmjopen.bmj.com/site/about/guidelines.xhtml

Barts and The London
School of Medicine and Dentistry

Our systematic review conducted in lines with current standards [13] identified 472 relevant citations. Nine RCTs met fully the review's inclusion criteria [14,15,16-22]. Most studies were of moderate quality; but sample sizes were generally small (from 65 women for quality of life in mental health domain to 259 women for depression).

4.2. Effect of Mindfulness based meditation in chronic pain patients

Our results showed Mindfulness based meditation reduced depression levels in chronic pain patients (standardised mean difference (SMD) -0.28; 95% CI -0.53, -0.03; p = 0.03)). Patients who received Mindfulness meditation tended to cope better with anxiety (SMD -0.16, 95% CI -0.47, 0.15) and affective pain (the emotional reaction to pain) (SMD -0.13, 95% CI -0.42, 0.16). Women in the intervention arm had also higher Quality of life (QOL) scores (especially the mental health component SMD 0.65, 95% CI -0.27, 1.58) and higher pain acceptance (SMD 0.53, 95% CI -0.13, 1.19); although these results were not statistically significant. Only one of the included studies reported the important measure of pain acceptance.

Currently Mindfulness-based therapy is creating lively research interest. Two recent systematic reviews report positive effects on somatisation disorders [23] and psychological stress [24].

4.3. On-going studies

Although there are currently no on-going studies of Mindfulness in patients with CPP that we are aware of, there are other NIHR funded studies with overlapping themes**.**

Self help in CPP

The RFPB-funded study SUPPORT, which is currently in follow- up (MREC 10/H1005/24), is investigating an evidence-based self-care guidance in general practice for women with CPP. GPs received training to use the guidance in their consultations. Women were randomised to either receive the facilitated self-care guide or usual care. Results from SUPPORT will provide valuable information on how best to integrate a new patient self-help intervention into an existing patient pathway.

For peer review only - http://bmjopen.bmj.com/site/about/guidelines.xhtml

Interactive mobile phone application to modify patient behaviour

The recently closed RFPB-funded feasibility study STARFISH (MREC 12/WS/0309) investigated the acceptability of a smartphone app that encourages stroke patients to become more physically active. The number of steps taken per day by the individual is monitored. Patients work in small groups and different goals can be set for different individuals in the group, along with goals for the whole group. It will be interesting to compare the reported obstacles and facilitators to using the app with MEMPHIS.

Web-based delivery of an intervention

Of particular interest, due to the similarities in study design to MEMPHIS, is a recently closed pilot study, MIMS (UKCRN ID 13105) that investigated adjustment to multiple sclerosis.

In MIMS, meditation teaching was delivered by videoconference. Web-based delivery has also been explored and shown to be feasible for reducing stress, anxiety and depression [25]; both options are lacking the flexibility of a smartphone app, which we are proposing.

4.4. Implications for the further development of clinical or public health practice

Our co-investigator Judy Birch is closely involved with the committee that produces national guidelines for CPP patient care pathways, which she helps to develop [26]. If the app were proven to be effective in a phase III trial, it would be possible for it to be incorporated in this pathway.

One outcome measure of MEMPHIS is to determine whether this app can be integrated into clinical practice, especially CPP pathways. If this is the case there would be benefit from studying how to extend the app to other pain conditions, such as headache, back pain and irritable bowel syndrome, in which face-to face delivered mindfulness meditation has had positive effects [23].

For peer review only - http://bmjopen.bmj.com/site/about/guidelines.xhtml

If this app is shown to be effective in a phase III trial, we will collaborate closely with Headspace, our local Health and Education Cluster and Queen Mary to implement this app both locally and nationally.

4.5. Potential impact on local policy making and improvement in service delivery

Chronic pelvic pain patients would benefit from multiple treatment approaches [6] but currently most gynaecological departments only offer medical and/or surgical treatment [5]. Although psychological treatment is provided across the NHS, mostly in the context of the primary care programme *Improving Access to Psychological Therapies* there are problems with capacity, waiting times, and the overall number of patients being able to access services. If the app is proven to be useful in a phase III RCT this gap could be filled, without having to employ more psychologists, because the interventions would be largely app delivered. Locally this would help our concerns about access to psychological treatment for CPP. Given the ubiquity of the app, greater compliance with treatment and less wastage from patients not attending appointments is expected. The use of the app in local primary, secondary and tertiary care settings would be introduced in collaboration with GP commissioning groups through local guidelines and protocols.

5. TRIAL OBJECTIVES

5.1. Aims and Objectives

The overall aim is to assess the feasibility of implementing a trial of a mindfulness meditation intervention delivered by a mobile phone app for patients with chronic pelvic pain (CPP). The primary objectives are:

- To provide feasibility data for a large multicentre RCT aimed at rigorously testing Mindfulness meditation in patients with CPP. The full-scale trial will assess the effectiveness of the mindfulness meditation app in patients with chronic pelvic pain in a national multicentre RCT

For peer review only - http://bmjopen.bmj.com/site/about/guidelines.xhtml

- To determine whether this app can be seamlessly integrated into clinical practice, especially CPP pathways. In cooperation with the Pelvic Pain Support Network, which is instrumental in the initiative on implementing nationwide pathways for patients with CPP, we will review the data on feasibility, especially the patient feedback and process analysis to answer this question to find out if the app, if it has been shown to be effective could be incorporated straight away into a national clinical pathway for CPP patients

5.2. Feasibility outcomes

5.2.1. Feasibility outcomes collected from participants

- Duration of recruitment (measured from the day recruitment opens until the day the 90th patient is randomised).
- Estimates to be used for the sample size calculation of the phase III RCT (the estimated SD for pain acceptance, and the dropout rate).
- Patient adherence to app use will be measured by the following outcomes:
    - Number of days (within the first 60 days from randomisation) a patient has used the app (with app use defined as having completed at least 90% of a session).
    - Whether the patient has used the app on 22 or more days within the first 60 days from randomisation.
    - Number of weeks (within the first eight weeks from randomisation) a patient has used the app on three or more days.
    - Whether the patient has used the app on three or more days in 6 or more weeks (within the first eight weeks from randomisation).
    - Whether the patient has used the app on 22 or more days within the first 60 days from randomisation, AND used the app on three or more days in 6 or more weeks within the first eight weeks from randomisation.
- Reasons for patient non-adherence to app use.

For peer review only - http://bmjopen.bmj.com/site/about/guidelines.xhtml

Barts and The London
School of Medicine and Dentistry

5.2.2. Feasibility outcomes collected from participant focus groups

- Usability and integration into clinical practice will be explored in two focus groups post-intervention with approximately 15 app participants, who have completed the 60 day follow up. Alternatively, participants unable to attend focus groups will be given the chance to answer a questionnaire over the phone with a research nurse.
- Discussions will be recorded and literal themes on integration and usability will be evaluated for in depth information. This information will be considered as well as adherence to the app as an indirect measure of acceptability. In cooperation with the Pelvic Pain Support Network, which is instrumental in the initiative on implementing nationwide pathways for patients with CPP, we will review the data on feasibility, especially the patient feedback and process analysis to answer this question to find out if the app, If it has been shown to be effective could be incorporated straight away into a national clinical pathway for CPP patients.
- We will determine primary and secondary outcomes of interest from the perspective of patients, for a full-scale trial. This will involve asking participants who were randomised to the app groups to discuss and prioritise outcomes.
- Obstacles to recruitment will also be explored.

5.2.3. Feasibility outcomes collected from health care practitioner focus groups

- A purpose made topic guide will be used to structure a focus group with service providers and based on the NPT toolkit [27] and the Diffusion of Innovations Theory [28] as a prompt for the facilitator.

The service providers will be asked to consider their role and their organisation and to suggest and discuss any issues to integration, and also – unlike conventional qualitative research focus groups – to suggest potential solutions. Discussions will be based around Diffusion of Innovations Theory, that is, we will consider:

- Relative advantage vs. existing practices

For peer review only - http://bmjopen.bmj.com/site/about/guidelines.xhtml

- Compatibility with existing practices
- Simplicity and ease of integration
- Trialability and reinvention of the process
- Feedback (e.g. can clinicians see that patients benefit?)
- Peer to peer networking

We will use our findings to develop our integration approach to be further explored in the subsequent full trial.

- Obstacles to recruitment will also be explored.

5.3. Clinical outcomes

- Quality of life score, Physical Functioning subscale (as measured by the RAND Short Form (36) Health Survey (SF-36))
- Quality of life score, Social Functioning subscale (as measured by the RAND SF-36)
- Quality of life score, Pain subscale (as measured by the RAND SF-36)
- Quality of life score, General Health subscale (as measured by the RAND SF-36)
- Depression score (as measured by the Hospital Anxiety and Depression Scale (HADS))
- Anxiety score (as measured by HADS)
- Mindfulness score (as measure by the Cognitive and Mindfulness - Revised (CAMS – R) scale)
- Pain related disability score (as measured by the Chronic Pain Grade (CPG) disability subscale)
- Self efficacy score (as measured by the Pain Self-Efficacy Questionnaire (PSEQ))
- Pain acceptance score (as measured Chronic Pain Acceptance Questionnaire (CPAQ-8))
- Sexual Health Outcomes score (as measured by Sexual Health Outcomes in

For peer review only - http://bmjopen.bmj.com/site/about/guidelines.xhtml

Barts and The London
School of Medicine and Dentistry

Women Questionnaire (SHOW-Q))

- Subjective outcome score (as measured by Measure Yourself Medical Outcome Profile (MYMOP))

All clinical outcomes will be analysed at 60 days, 3 months, and 6 months post-randomisation.

6. METHODOLOGY

6.1. Inclusion Criteria

To be eligible for the MEMPHIS study, the women must meet the following criteria:

- Aged 18 or over
- Women with organic and non-organic chronic pelvic pain lasting for six months or more
- Be capable of understanding the information provided, with use of an interpreter if required and being able to understand simple English as is used in the app
- Give written informed consent

6.2. Exclusion Criteria

Patients who mee**t the following criteria are** ineligible to participate:

- No access to a Personal computer or smartphone
- Current users of the Headspace app content available to the public

6.3. Study Design

MEMPHIS is a randomised, single centre feasibility trial. All eligible women referred to the chronic pelvic pain clinics at the Royal London and Whipps Cross Hospitals (both new and existing patients) will be approached to take part in the study. A study leaflet will be given to them, providing brief information of the study and informing them that they are invited to participate. After informed consent, we will randomise

For peer review only - http://bmjopen.bmj.com/site/about/guidelines.xhtml

eligible women in a 1:1:1 ratio (30 participants in each group) to one of the three treatment groups:

Group A - “Intervention”: 60 days of the app delivering mindfulness meditation content (in addition to usual care). See section 7.4 for a detailed description.

Group B - “Active control”: 60 days of the app delivering progressive muscle relaxation content (in addition to usual care). See section 7.4 for a detailed description.

Group C - Treatment as usual (TAU): Usual care

Setting: NHS Tertiary care hospital

6.4. Study Scheme Diagram

Figure 1

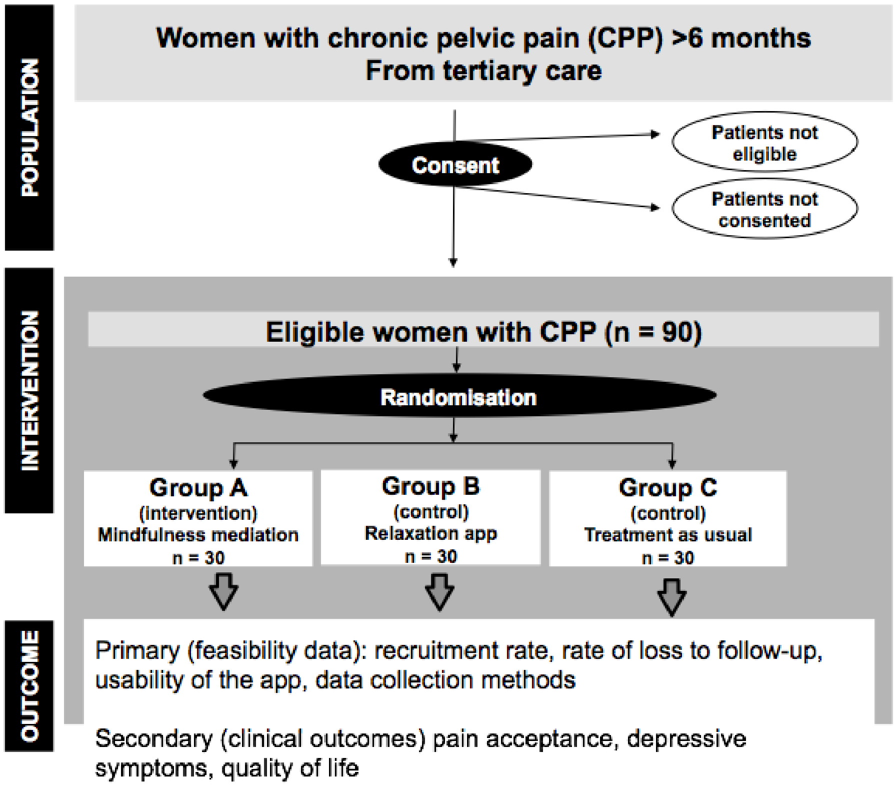

For peer review only - http://bmjopen.bmj.com/site/about/guidelines.xhtml

7. STUDY PROCEDURES

7.1. Informed Consent Procedures

Women will be made aware of the study by a health care professional and through promotional material. Potentially eligible patients will receive the PIS along with their hospital appointment invitation to ensure they have adequate time (at least 24 hours) to consider the trial. The PIS will be accompanied with a letter from the PI informing the women that they may be approached about the study at their appointment. Eligible patients who are seen in clinics other than pelvic pain and endometriosis clinics will be given the PIS and contact details for the research practitioner so they can benefit from participating in MEMPHIS should they wish so.

The PIS will be reviewed and the patient will have the opportunity to ask any questions. All eligible participants willing to consent will be asked to sign the consent form. Women will be provided with the contact details of the researcher, and informed that they have the right to withdraw their consent at any stage. Some women may be asked for permission to be contacted by a research practitioner at a later stage for enrolment if there are time constraints.

Only those on the delegation log will be able to consent for the intervention. The consenting staff will have thorough knowledge of research governance issues surrounding consent and will be fully conversant with the protocol.

If they are eligible but do not wish to consent, this will be recorded. For the full scale trial we need to understand how many eligible patients need to be approached to reach the recruitment target. We also would like to identify if eligible women opt out of the study due to a rectifiable issue.
Women who give their approval will be randomised. The investigator (or another qualified person) will explain to the potential participant that they are free to refuse any involvement within the study or alternatively withdraw their consent at any point during the study and for any reason.

For peer review only - http://bmjopen.bmj.com/site/about/guidelines.xhtml

If there is any further safety information, which may result in significant changes in the risk/benefit analysis, the PIS and Informed Consent Form (ICF) will be reviewed and updated accordingly. All participants who are actively enrolled on the study will be informed of the updated information and given a revised copy of the PIS/ICF in order to confirm their wish to continue on the study (if feasible), if it may change their willingness to participate. A copy of the consent form will be given to the participant; one will be kept in the hospital notes and the original will be placed in the Investigator Site File.

7.2. Screening and enrolment

New referrals and existing patients at the pelvic pain clinic are equally eligible. Through links with the Katherine Twining network and UCL partners we have established networks that can advertise recruitment. Based on these circumstances we are confident that we can achieve successful recruitment in the given timeframe.

Patients will be sent the Patient Information Sheet (PIS) in advance to ensure they have adequate time to consider the trial. The PIS will be accompanied with a letter from the PI informing patients that they may be approached about the study at their appointment.

At the appointment, the research practitioner will assess the women according to the inclusion/exclusion criteria detailed above and explain the nature of the intervention. The PIS will be reviewed and the patient will have the opportunity to ask any questions. All eligible participants willing to consent will be asked to sign the consent form. If a woman has not read or received the PIS before their appointment, the research team will go through the PIS with the individual in person. Women will be giving as much time as they want to consider the study before consent is taken. Women will be provided with the contact details of the researcher, and informed that they have the right to withdraw their consent at any stage.

For peer review only - http://bmjopen.bmj.com/site/about/guidelines.xhtml

Barts and The London
School of Medicine and Dentistry

7.3. Randomisation Procedures

After informed consent, patients will be randomised in a 1:1:1 ratio to one of the three treatment groups, using permuted blocks without stratification. Randomisation will be performed using a centralised internet service, hosted by the Pragmatic Clinical Trials Unit. The schedule of intervention with timeline is detailed below.

7.4. Blinding

When a participant is randomised the randomisation system will only display whether they have been allocated to an "app" treatment group (either the "Intervention" or "Active Control" group, but not which one) or the "Treatment as usual" group. If a participant is randomised to either "app" treatment group, then the randomisation system will supply an alphanumeric token which is redeemed when registering to receive the app. This will ensure that the correct content (mindfulness meditation or progressive muscle relaxation) is delivered to each participant. Therefore, the participant and recruiting staff will NOT be blinded to allocation of the "Treatment as usual" or "app" groups. However, at randomisation they will be blinded to whether allocation is to "Intervention" or "Active Control" group.

To preserve blinding of participants as much as possible, "Intervention" and "Active Control" groups will be using the same app, and hearing instructions for the same duration, delivered by the same narrator. Only the content of the instructions will differ. In addition, the Patient Information Sheet and consent form do not explicitly refer to "mindfulness meditation" or "progressive muscle relaxation".

Outcomes are collected in paper questionnaires completed by participants. The 6 month questionnaire includes a question to determine whether the participants randomised to the app have been unblinded to the "Intervention" app or "control" app. The researcher will answer a short questionnaire after recruiting each participant to determine if they have been unblinded to the "Intervention" app or "control" app, for participants randomised to an app.

Statisticians will be blinded to individual treatment allocations until required for the final analysis. If necessary, an independent statistician will perform any interim analysis which require unblinding of the data.

For peer review only - http://bmjopen.bmj.com/site/about/guidelines.xhtml

Barts and The London
School of Medicine and Dentistry

It is not anticipated that any emergency unbinding will be necessary.

7.5. Planned interventions

After eligible women have been allocated to one of the 3 groups, the participants in the Intervention and the Active Control group (progressive muscle relaxation app) will receive a face-to-face introduction to using the app. After that, the Intervention group will use the app over 60 days.

The meditation content is a structured and progressive course, layering in new techniques and concepts over successive sessions. The course was created and narrated by a former monk - Andy Puddicombe - drawing on a secularised version of the techniques he was taught over 10 years' experience in monasteries around the world.

The techniques used in the Intervention are shown in the table below. The first 30 days cover basic techniques, assuming no previous experience of meditation. The second 30 days focus specifically on the use of these techniques with respect to pain. The duration of individual sessions builds over time. Days 1-10 are 10 minutes in duration, days 11-20 are 15 minutes in duration, and days 21-60 are 20 minutes in duration.

The Active Control group will use the same app, but the app will be configured so that they will hear a series of non-meditative progressive muscle relaxation instructions, also narrated by Andy Puddicombe. These sessions will be identical every day, except that their duration will increase to mirror the increasing duration of the meditation content being listened to by the Intervention group.
In this way, both Intervention and Control groups will be using the same app, and hearing instructions for the same duration, delivered by the same narrator. Only the content of the instructions will differ.

For peer review only - http://bmjopen.bmj.com/site/about/guidelines.xhtml

Barts and The London
School of Medicine and Dentistry

Series	Techniques involved
Take 10/Foundation 1 (first 10 days)	Open monitoring, body scan, breath as anchor
Foundation 2 (days 11-20)	As above, plus intention and altruism
Foundation 3 (days 21-30)	As above, plus integration of mindfulness with daily activities
Pain series (days, 31-60)	As above, plus visualisation and enquiry (insight/Tibetan vipassana)

7.6. Concomitant Medications

Patients are able to receive any concomitant medications that they would as part of usual care.

7.7. Reasons for non progression to full trial

- Insurmountable problems with recruitment
- Extremely high rates of loss-to-follow-up
- Extremely low rates of adherence to the intervention
- Unacceptability of intervention for patients

7.8. Key risks to delivering this research and contingencies:

- Recruitment of 90 patients between May 2016 and October 2016 not achieved – regular monitoring throughout recruitment period to identify and resolve problems (e.g. open new centres/extend recruitment period)
- We will monitor regularly if patients have not downloaded apps and offer further one-to-one support
- Data collection issues will be monitored and addressed early where possible; this will inform the full-scale RCT design

For peer review only - http://bmjopen.bmj.com/site/about/guidelines.xhtml

Barts and The London School of Medicine and Dentistry

- Issues relating to the other milestones (ethics, personnel, app availability) and deliverables will be rectified, but potentially delay the start of MEMPHIS/full-scale trial. Contamination was not thought likely by the patient group

7.9. Procedure for Collecting Data

Patients will enter the data on paper questionnaires, which will be transferred into a purpose-built electronic database.

1.) Scales for clinical outcomes
2.) App satisfaction questionnaire, which includes open comment boxes and tick-boxes based on published questionnaires [30].

As an incentive to complete and return the patient questionnaires, a £5 shopping voucher will be sent in the post with each follow up questionnaire alongside a stamped addressed envelope.
In the case that a questionnaire is not received, participants will be sent a text reminder. Non-responders will then be contacted by telephone in order to collect a smaller dataset.

7.10. Including Case Report Forms (CRFs) and storage

In line with GCP guidance we will keep the data stored for 20 years following the close of the study to allow for verification and any further data sharing e.g. individual patient data meta-analysis.

We will follow the PCTU's standard operating procedures for legacy archiving.
Queen Mary University of London will act as custodians of the data.

7.11. Follow-up Procedures

Some of the participants will be asked for permission to elaborate on the open comment boxes about app satisfaction and also on clinical outcomes in two focus

For peer review only - http://bmjopen.bmj.com/site/about/guidelines.xhtml

Barts and The London
School of Medicine and Dentistry

groups to be held after the 6 month follow up point finisheswith participants asked to discuss and prioritise outcomes. Alternatively, participants unable to attend focus groups will be given the option to answer a questionnaire over the phone with a researcher.

7.12. Subject withdrawal (including data collection / retention for withdrawn participants)

A participant can be withdrawn from the trial if, in the opinion of the investigator or the care providing clinician or clinical team, it is medically necessary to do so.

With any post randomisation exclusions, the study personnel will make every effort to obtain, and record, information about the reasons for violation, any adverse events and to follow-up the women for all safety and efficacy outcomes, as appropriate. If a woman decides after randomisation she does not wish to participate any further in the MEMPHIS trial, she may withdraw herself from the trial. We will aim to document the reason for self-withdrawal. Clear distinction will be made as to whether the participant is withdrawing from trial whilst allowing further follow-up, or whether the participant refuses any follow-up. If a participant explicitly withdraws consent to have any further data recorded their decision will be respected and recorded on the final study form. All communication surrounding the withdrawal will be noted in the study records and no further data will be collected for that participant. They will be returned to the NHS standard practice for follow up care.

If a woman loses their ability to consent during participation in the trial, they will be withdrawn from the trial and no further data will be collected from the participant unless consent for this was explicitly obtained prior to the loss of capacity.

7.13. Continued app use after trial period and app use by treatment as usual group

It was decided to permit continued app use to the end of the study to reflect the situation in real life. Duration of use will be recorded through the app without using patient identifiable data.

For peer review only - http://bmjopen.bmj.com/site/about/guidelines.xhtml

Barts and The London
School of Medicine and Dentistry

Consideration was given to inform patients in the ’treatment as usual’ arm at the beginning that they will be able to access the meditation app at the end of the study, but this was abandoned due to concerns that this could lead to bias. Research has shown [31] that in those circumstances patients may decide to ‘wait’ until the end of the intervention before trying to improve, and as a consequence, they tend to improve less, leading to overestimating the effect of the intervention. It is possible that without the offer of delayed app use recruitment may be slower, which is something we would like to determine in the feasibility study. However, if after close involvement with the PPI this appears to be not acceptable to patients as compromise such as telling control patients after the end of the study that they are now allowed to use the app may be offered.

7.14. Schedule of Assessment

Health outcome measures are collected at baseline. The delivery of the intervention or control will occur for 60 days. Health outcome measures are collected immediately after the intervention at 60 days, and again at 3 and 6 months. App satisfaction/usability measures will be collected immediately after the intervention at 60 days from app participants.

The usability and clinical outcome focus groups will take place after the 6 month follow up point.

Assessment	Baseline	During intervention	60 days post randomisation	3 months post randomisation	6 months post randomisation
Questions about participants pain	V				
History of pain treatment	V				
Personal details	V				
Adherence to app use		V			
Clinical outcome questionnaires	V		V	V	V

For peer review only - http://bmjopen.bmj.com/site/about/guidelines.xhtml

Barts and The London
School of Medicine and Dentistry

App satisfaction questionnaires			V		
Interview/focus group with recruiters, nurses, patients, other stakeholders on usability and integration into practice					V
HCP and patient focus groups on clinical outcomes					V

For peer review only - http://bmjopen.bmj.com/site/about/guidelines.xhtml

Barts and The London
School of Medicine and Dentistry

7.15. Criteria for Early Termination of the study

The nature of the intervention and follow−up makes it unlikely that any new information will impact an individual participant. If the TSC committee, REC, CI or sponsor determine it is within the best interests of the participants or trial to terminate the study, written notification will be given to the CI. This may be due to, but not limited to; safety concerns, proof of efficacy or non-compliance/serious breaches. If the study is terminated participants will be returned to the NHS normal follow up and routine care.

7.16. End of Study Definition

When the last enrolled participant has completed follow up, the REC will be notified of the trial completion. The final study report will be completed within 12 months after the trial completion.

8. STATISTICAL CONSIDERATIONS

8.1. Sample Size

30 participants will be recruited to each of the three treatment groups, giving a total of 90 participants. As this is a feasibility study, we have not performed a sample size calculation based upon the power to detect a significant treatment effect on a clinical outcome. However, 90 participants should provide a reliable estimate for the standard deviation of the primary clinical outcome (likely to be pain acceptance) [32, 33], which can be used to inform the sample size calculation of the main trial.

8.2. Statistical Analysis

A full analysis plan will be developed and agreed prior to any analysis or unblinding of the data.

For peer review only - http://bmjopen.bmj.com/site/about/guidelines.xhtml

Barts and The London School of Medicine and Dentistry

Baseline

Baseline variables will be presented for each treatment group as the mean (SD) or median (IQR) for continuous variables, and the number (%) for categorical variables.

Analysis of Feasibility Outcomes

Feasibility outcomes will be presented for each treatment group as the mean (SD) or median (IQR) for continuous variables, and the number (%) for categorical variables.

Duration of recruitment will be calculated as the number of days from the beginning to the end of recruitment. The number of participants recruited per month will be presented.

The proportion of patients in each treatment group who have returned data at each follow-up time point (60 days, 3 months, and 6 months post-randomisation) will be presented. Summaries of baseline variables will be presented separately for patients who have and have not returned data at each at the 6 month time point.

Adherence outcomes will be summarised separately for the intervention and active control treatment groups. Adherence outcomes will be presented as the mean (SD) or median (IQR) for continuous variables, and the number (%) for categorical variables.

An estimate of the standard deviation of pain acceptance (CPAQ) in each treatment group at each follow up time point (60 days, 3 months, and 6 months) will be presented.

Analysis of Clinical Outcomes

For each clinical outcome we will present the following information:

- The number of patients in each treatment group with an observed outcome at each follow-up time point.

For peer review only - http://bmjopen.bmj.com/site/about/guidelines.xhtml

- The mean (SD) in each treatment group at each follow-up time point.
- The estimated treatment effect at each follow-up time point, with a 95% confidence interval.

Estimates of treatment effect will be presented comparing the intervention group (mindfulness meditation app) to the control (treatment as usual) group, the intervention group to the active control (progressive muscle relaxation app) group, and the active control group to the control (treatment as usual) group. Outcomes will be analysed using linear mixed-effects models to account for the correlation between patient outcomes at different follow-up time points [34], and adjusted for baseline measure of the outcome [35]. Patient data will be analysed according to the treatment group to which they were randomised (intention-to-treat). All patients with an observed outcome for at least one of the three follow-up time points (60 days, 3 months, or 6 months) will be included in the analysis [36].

Analysis of usability and integration of app

- Obstacles to recruitment will be summarised
- The integration of the app into existing and emerging patient pathways will be investigated using questionnaires developed from social contagion theory and Normalisation Process Theory (NPT) as described in section 5.3. The maximum total score using NPT is 64. The maximum total score using the Diffusion of Innovations questionnaire is 200.
  The System Usability Scale (SUS) [28] has a maximum score of 50.

9. ETHICS

The Investigator to an Independent Research Ethics Committee will submit this protocol and any subsequent amendments, along with any accompanying material provided to the participant in addition to any advertising material. Written Approval from the Committee will be obtained and subsequently submitted to the JRMO to

For peer review only - http://bmjopen.bmj.com/site/about/guidelines.xhtml

Barts and The London School of Medicine and Dentistry

obtain Final R&D approval. The trial can only start after approval from a Research Ethics Committee and the local R&D "Sign-off" from the participating centre. If there is any further safety information, which may result in significant changes in the risk/benefit analysis, the Patient Information Sheet (PIS) and Informed Consent Form (ICF) will be amended accordingly and submitted to REC for revision and approval. All participants that are actively enrolled on the study will be informed of the updated information and given a revised copy of the PIS/ICF in order to confirm their wish to continue on the study (if feasible), if it may change their willingness to participate.

10. SAFETY CONSIDERATIONS

There are no known side effects arising from mindfulness meditation.

11. DATA HANDLING AND RECORD KEEPING

11.1. Confidentiality

Patient anonymity is protected and maintained. This applies to data collected on paper or via the headspace database.

We will ensure that patient identities are protected from any unauthorised parties. Information with regards to study patients will be kept confidential and managed in accordance with data Protection Act, NHS Caldicott Guardian, The research Governance Framework for Health and Social care and Research Ethics Committee Approval.

The trial will collect personal data and sensitive information about the participants either directly or from their clinical team. Participants will be informed about the transfer of this information to the study office and will be asked to consent to this. The data will be entered onto a secure computer database, either by trials unit staff or directly via a secure Internet connection. Any data to be processed will be anonymised. All personal information obtained for the trial will be held securely and treated as (strictly) confidential. All staff, at the hospital or the trials unit shares the

For peer review only - http://bmjopen.bmj.com/site/about/guidelines.xhtml

same duty of care to prevent unauthorised disclosure of personal information. No data that could be used to identify an individual will be published.

In relation to the data collected by Headspace the following applies:

Headspace will not collect any clinical data, but data on app usage. Details collected on the headspace database will be confidential. Details about the individual's use of Headspace tools will never be seen by or shared with anyone outside the research team and the company. Individual usage and demographic information will only be used by Headspace in accordance with the standard Headspace user terms and conditions. No data will be shared with any other organizations, unless with prior agreement, and all data is kept confidential. App usage data will be transferred to the research team via a securely encrypted file.

The Chief investigator, Miss Elizabeth Ball is the "custodian" of the data.

11.2. Required Study Documents

- A signed protocol and any subsequent amendments
- PCTU self-monitoring template for the trial team to complete on a regular basis as detailed by the Trial Monitoring section
- Current and Superseded Patient Information Sheets
- Current and Superseded Consent Forms
- Current and Superseded GP letters
- Current and Superseded Posters
- Current and Superseded CRFs
- Indemnity documentation from sponsor
- Conditions of Sponsorship from sponsor
- Conditional/Final R&D Approval
- Signed site agreements
- Ethics submissions/approvals/correspondence
- CVs and GCP certificates of CI and site staff

For peer review only - http://bmjopen.bmj.com/site/about/guidelines.xhtml

Barts and The London School of Medicine and Dentistry

- Laboratory accreditation letter, certification and normal ranges for all laboratories to be utilised in the study
- Delegation log
- Staff training log
- Identification log
- Enrolment log
- Monitoring visit log
- Correspondence relating to the trial
- SAE reporting plan for the study

11.3. Record Retention and Archiving

During the course of research, all records are the responsibility of the Chief Investigator and must be kept in secure conditions. When the trial is complete, it is a requirement of the Research Governance Framework and Trust Policy that the records are kept for a further 20 years. For trials involving Barts Health Trust patients, undertaken by Trust staff, or sponsored by Barts Health trust or QMUL, the approved repository for long-term storage of local records is the Trust Modern Records centre, which is based at 9 Prescott Street.

12. PRODUCTS, DEVICES, TECHNIQUES AND TOOLS

12.1. Devices

The Medicines and Healthcare products Regulatory Agency (MHRA) states that some apps can be classified as medical devices. [37]

However, apps with software that provides general information but does not provide personalised advice, although it may be targeted to a particular user group, is unlikely to be considered a medical device. We believe that neither the mindfulness meditation nor the progressive muscle relaxation content in the app fulfil the criteria for medical devices.

For peer review only - http://bmjopen.bmj.com/site/about/guidelines.xhtml

Barts and The London
School of Medicine and Dentistry

12.2. Techniques and interventions

Intervention (mindfulness meditation content):

60 days of guided meditation content. The first 30 days cover basic techniques, assuming no previous experience of meditation. The second 30 days focus specifically on the use of these techniques with respect to pain. The duration of individual sessions builds over time. The first 10 days are each 10 minutes in duration. The next 10 days are each 15 minutes in duration. All following days are 20 minutes in duration. The minimum usage of app should be for at least 22 out of 60 days.

It was decided to permit continued app use to the end of the study to reflect the situation in real life. Duration of use will be recorded through the app without using patient identifiable data.

Control:

1) Treatment as usual (watch and wait, medication and/or surgery) to investigate if any app intervention makes a difference to wellbeing and to ascertain dropout rates for the full-scale trial in patients who perceive that they are getting no intervention

2) 60 days of progressive muscle relaxation content: This group will use the same app as the Intervention group, but the app will be configured so that they will hear a series of non-meditative progressive muscle relaxation instructions. These sessions will be identical every day, except that their duration will increase to mirror the increasing duration of the meditation content being listened to by the Intervention group (10 minutes a day for 10 days, then 15 minutes a day for 10 days, then 20 minutes a day thereafter.)

App satisfaction questionnaires

- Purpose made questionnaire (Carol Rivas)
- The System Usability Scale (SUS) [28]

For peer review only - http://bmjopen.bmj.com/site/about/guidelines.xhtml

Barts and The London
School of Medicine and Dentistry

13. SAFETY REPORTING

13.1. Adverse Events (AE)

An AE is any untoward medical occurrence in a subject to whom a medicinal product has been administered, including occurrences which are not necessarily caused by or related to that product. An AE can therefore be any unfavourable and unintended sign (including an abnormal laboratory finding), symptom or disease temporarily associated with study activities.
We do not expect SAEs related to use of the mindfulness or the progressive muscle relaxation app.

Notification and reporting Adverse Events or Reactions

If the AE is not defined as SERIOUS, the AE is recorded in the study file and the participant is followed up by the research team. The AE is documented in the participants' medical notes (where appropriate) and the CRF.

13.2. Serious Adverse Event (SAE)

A serious adverse event (SAE) is defined as an untoward occurrence that:

(a) results in death;
(b) is life-threatening;
(c) requires hospitalisation or prolongation of existing hospitalisation;
(d) results in persistent or significant disability or incapacity;
(e) consists of a congenital anomaly or birth defect; or
(f) is otherwise considered medically significant by the investigator.

An SAE occurring to a research participant should be reported to the main REC where in the opinion of the Chief Investigator the event was:

• Related – that is, it resulted from administration of any of the research procedures, and

For peer review only - http://bmjopen.bmj.com/site/about/guidelines.xhtml

• Unexpected – that is, the type of event is not listed in the protocol as an expected occurrence.

Notification and Reporting of Serious Adverse Events

Serious Adverse Event (SAEs) that are considered to be 'related' and 'unexpected' are to be reported to the sponsor within 24 hours of learning of the event and to the Main REC within 15 days in line with the required timeframe. For further guidance on this matter, please refer to NRES website and JRMO SOPs

13.3. Urgent Safety Measures

The CI may take urgent safety measures to ensure the safety and protection of the clinical trial subjects from any immediate hazard to their health and safety,. The measures should be taken immediately. In this instance, the approval of the REC prior to implementing these safety measures is not required. However, it is the responsibility of the CI to inform the sponsor and Main Research Ethics Committee (via telephone) of this event immediately.

The CI has an obligation to inform both the Main REC in writing within 3 days, in the form of a substantial amendment. The sponsor (Joint Research Management Office (JRMO)) must be sent a copy of the correspondence with regards to this matter. For further guidance on this matter, please refer to NRES website and JRMO SOPs.

13.4. Annual Safety Reporting

The CI will send the Annual Progress Report to the main REC using the NRES template (the anniversary date is the date on the MREC "favourable opinion" letter from the MREC) and to the sponsor. Please see NRES website and JRMO SOP for further information

For peer review only - http://bmjopen.bmj.com/site/about/guidelines.xhtml

Barts and The London School of Medicine and Dentistry

13. 5. Overview of the Safety Reporting responsibilities

The CI/PI has the overall pharmaco-vigilance oversight responsibility. The CI/PI has a duty to ensure that safety monitoring and reporting is conducted in accordance with the sponsor's requirements.

14. MONITORING & AUDITING

14.1. Auditing

Definition: "A systematic and independent examination of trial related activities and documents to determine whether the evaluated trial related activities were conducted, and the data were recorded, analysed and accurately reported according to the protocol, sponsor's standard operating procedures (SOPs), Good Clinical Practice (GCP), and the applicable regulatory requirement(s)."

A study may be identified for audit by any method listed below:

1. A project may be identified via the risk assessment process.
2. An individual investigator or department may request an audit.
3. A project may be identified via an allegation of research misconduct or fraud or a suspected breach of regulations.
4. Projects may be selected at random. The Department of Health states that Trusts should be auditing a minimum of 10% of all research projects.
5. Projects may be randomly selected for audit by an external organisation.

Internal audits may be conducted by a sponsor's or funder representative.

14.2. Summary Monitoring Plan

Investigators and their host Trusts will be required to permit study-related monitoring and audits to take place, providing direct access to source data and documents as requested. Trusts may also be subject to inspection by the Research and Development Manager and should do everything requested by the Chief Investigator in order to

For peer review only - http://bmjopen.bmj.com/site/about/guidelines.xhtml

prepare and contribute to any inspection or audit. Study participants will be made aware of the possibility of external audit of data they provide in the participant information sheet.

14.3. Compliance

The CI will ensure that the trial is conducted in compliance with the principles of the Declaration of Helsinki (1996), and in accordance with all applicable regulatory requirements including but not limited to the Research Governance Framework, GCP, Trust and Research Office policies and procedures and any subsequent amendments.

14.4. Non-Compliance

Definition: A noted systematic lack of both the CI and the study staff adhering to the principles of the Declaration of Helsinki (1996), applicable regulatory requirements including but not limited to the Research Governance Framework, GCP, Trust and Research Office policies and procedures and any subsequent amendments, which leads to prolonged collection of deviations, breaches or suspected fraud.

These non-compliances may be captured from a variety of different sources including monitoring visits, CRFs, communications and updates. The sponsor will maintain a log of the non-compliances to ascertain if there are any trends developing or escalating. The sponsor will assess the non-compliances and action a timeframe in which they need to be dealt with. Each action will be given a different timeframe dependent on the severity. If the actions are not dealt with accordingly, the sponsor will agree an appropriate action, including an on-site audit.

15. TRIAL COMMITTEES

15.1. Trial Steering Committee (TSC)

The TSC provides independent supervision for the trial, providing advice to the Chief and Co-Investigators and the Sponsor on all aspects of the trial and affording

For peer review only - http://bmjopen.bmj.com/site/about/guidelines.xhtml

Barts and The London School of Medicine and Dentistry

protection for patients by ensuring the trial is conducted according to the principles of Good Clinical Practice in Clinical Trials. If the Chief and Co-Investigators are unable to resolve any concern satisfactorily, Principal Investigators, and all others associated with the trial, may write through the Trial Unit to the chairman of the TSC, drawing attention to any concerns they may have about the possibility of particular side-effects, or of particular categories of patient requiring special study, or about any other matters thought relevant.

15.2. Trial Management Group (TMG)

The trial management group will meet regularly to discuss operational issues. This will include the chief investigator, trial co-ordinator, senior research manager, statistician, data manager, QA manager and research administrator.

15.3. Data Monitoring Committee (DMC)

Based on the short duration of recruitment (expected to be 6 months) and the safety profile of the intervention, a DMC will not be used.

16. FINANCE AND FUNDING

-This study is funded by the Research for Patients Benefit national programme (RfPB).
- Headspace is donating subscriptions at no charge as part of their research initiative.

17. INDEMNITY

Queen Mary, University of London will act as a Sponsor, as defined by the Research Governance Framework for Health and Social Care (April 2005) for the project. The project will also be covered by the sponsor's insurance brokers on a "No Faults Compensation for Clinical Trials and/or Human Volunteer Studies". This policy will

For peer review only - http://bmjopen.bmj.com/site/about/guidelines.xhtml

indemnify/cover the insured in respect of their legal liabilities arising out of the insured's activities.

18. DISSEMINATION OF RESEARCH FINDINGS

The research findings of the feasibility study will be disseminated judiciously to avoid biasing the full-scale trial. In both trials we will disseminate our findings to:

1) Study participants through a dedicated website and newsletters at the end of the feasibility and full scale study, guided by our lay advisers

2) Participating health care professionals through the dedicated website and electronic newsletters

4) Professional groups via peer-reviewed journals and scientific meetings. Post-trial workshops run in collaboration with PPI group

5) Health service commissioners via the study website and an electronic newsletter

6) The wider public through local and national media and via dedicated website

7) Patients and relatives through PPI group

Applicants have links for dissemination via these organisations: Cochrane reviews, NICE, Pelvic pain support network (Judy Birch), Katherine Twining Network (KTN), BJOG (Khalid Khan), BSGE (Elizabeth Ball) Communications experts at our higher education institutions and the NIHR Collaboration for Leadership in Applied Health Research and Care North Thames will support our dissemination strategy through Twitter, Facebook and press coverage.

A particular strength of our application is our close links with:

For peer review only - http://bmjopen.bmj.com/site/about/guidelines.xhtml

Barts and The London
School of Medicine and Dentistry

1) KTN, dedicated to research and education in the UK and abroad via the East London International Women's Health Appeal, who will be able to disseminate this low cost-intervention in developing countries with high incidence of CPP [2]

2) UCL partners, whose focus is on patient-led population-focused delivery of research innovations.

For peer review only - http://bmjopen.bmj.com/site/about/guidelines.xhtml

Barts and The London School of Medicine and Dentistry

REFERENCES

1. Royal College of Obstetricians and Gynaecologists, green top guidelines (2012) Available at: https://www.rcog.org.uk/en/guidelines-research-services/guidelines/gtg41 (Accessed: 13 May 2015)

2. Latthe, P., Latthe, M., Say, L., Gülmezoglu, M., Khan, K.S., (2006), 'WHO systematic review of prevalence of chronic pelvic pain: a neglected reproductive health entity', BMC Public Health. , 6(6), pp. 177.

3. Howard, F.M., (1993), 'The role of laparoscopy in chronic pelvic pain: promise and pitfalls', Obstetrical and Gynecological Survey, 48(6), pp. 357-387.

4. Simoens, S., et al., (2012), 'The burden of endometriosis: costs and quality of life of women with endometriosis and treated in referral centres', Human Reproduction, 27(5) pp. 1292-1299

5. Stones, R.W. and Price, C., (2002), 'Health services for women with chronic pelvic pain', Journal of the Royal Society of Medicine, 95, pp. 531-535

6. Peters, A.A., et al., (1991), 'A randomized clinical trial to compare two different approaches in women with chronic pelvic pain', Obstetrics and Gynecology, 77(5), pp. 740-4

7. Warsi, A., et al., (2004), 'Self-management education programs in chronic disease: a systematic review and methodological critique of the literature', Archives of Internal Medicine, 164(15), pp. 1641-9.

8. Fox, S.D., Flynn, E., Allen, R.H., (2011), 'Mindfulness meditation for women with chronic pelvic pain: a pilot study', The Journal of reproductive medicine, 56(3-4), pp. 158-162.

9. Kold, M., et al., (2012), 'Mindfulness-based psychological intervention for coping with pain in endometriosis', Nordic Psychology, 64(1), pp. 2-16.

10. Cramer, H., et al., (2012), 'Mindfulness-based stress reduction for low back pain. A systematic review', BMC Complementary & Alternative Medicine, 12, pp. 162.

11. Kozasa, E.H., et al., (2012), 'The effects of meditation-based interventions on the treatment of fibromyalgia', Current Pain & Headache Reports, 16(5), pp. 383-7.

12. Veehof, M.M., et al., (2011), 'Acceptance-based interventions for the treatment of chronic pain: A systematic review and meta-analysis', Pain, 152(3), pp. 533-42

For peer review only - http://bmjopen.bmj.com/site/about/guidelines.xhtml

Barts and The London
School of Medicine and Dentistry

13. Higgins, J.P.T., Green, S., (2011) ‘Cochrane handbook for systematic reviews of interventions’, version 5.1.0. Cochrane Collaboration. Available at: www.cochrane-handbook.org (Accessed: 13 May 2015)

14. Brown, C.A. and Jones, A.K., (2013), ‘Psychobiological correlates of improved mental health in patients with musculoskeletal pain after a mindfulness-based pain management program’, Clinical Journal of Pain, 29(3), pp. 233-44.

15. Carson, J., et al., (2005), ‘Loving-kindness meditation for chronic low back pain: results from a pilot trial’, Journal of Holistic Nursing, 23(3), pp. 287-304.

16. Morone, N.E., Greco, C.M., Weiner, D.K., (2008), ‘Mindfulness meditation for the treatment of chronic low back pain in older adults: a randomized controlled pilot study’, Pain, 134(3), pp. 310-9.

17. Nash-Mc Feron, D.E., (2006), ‘Mindfulness in the treatment of chronic headache pain’, Dissertation Abstracts International: Section B: The Sciences and Engineering, 67(5-B), pp. 2841.

18. Plews-Ogan, M., et al., (2005), ‘A pilot study evaluating mindfulness-based stress reduction and massage for the management of chronic pain’, Journal of General Internal Medicine, 20(12), pp. 1136-8.

19. Sagula, D. and Rice, K.G., (2004), ‘The effectiveness of mindfulness training on the grieving process and emotional well-being of chronic pain patients’, Journal of Clinical Psychology in Medical Settings, 11(4), pp. 333-342.

20. Schmidt, S., et al., (2010), ‘Assessing a dynamical EEG pattern related to chronic pain-Results from a controlled evaluation of back pain patients as well as from an observational trial with a mindfulness based intervention’, European Journal of Integrative Medicine, 2 (4), pp. 196.

21. Sephton, S.E., et al., (2007), ‘Mindfulness meditation alleviates depressive symptoms in women with fibromyalgia: Results of a randomized clinical trial’, Arthritis Care and Research, 57(1), pp. 77-85.

22. Teixeira, E., (2010), ‘The effect of mindfulness meditation on painful diabetic peripheral neuropathy in adults older than 50 years’, Holistic Nursing Practice, 24(5), pp. 277-83.

23. Lakhan, S.E. and Schofield, K.L., (2014), ‘Mindfulness-Based Therapies in the Treatment of Somatization Disorders: A Systematic Review and Meta-Analysis’, PLOS One, 8(8): e71834

For peer review only - http://bmjopen.bmj.com/site/about/guidelines.xhtml

Barts and The London
School of Medicine and Dentistry

24. Goyal, M., et al., (2014), 'Meditation Programs for Psychological Stress and Well-being', JAMA Internal Medicine., 174(3), pp. 357-368

25. Krusche, A., Cyhlarova, E., Williams, J.M., (2013), 'Mindfulness online: an evaluation of the feasibility of a web-based mindfulness course for stress, anxiety and depression', BMJ Open., 3(11): pp. e003498.

26. http://bps.mapofmedicine.com/evidence/bps/chronic_pelvic_pain_for_men_and_women_1.html

27. May, C., Sibley, A., Hunt, K., (2013) 'The nursing work of hospital-based clinical practice guideline implementation: an explanatory systematic review using normalisation process theory', International Journal of Nursing studies, 51(2), pp.289-99

28. Rogers, E.M., (2003), Diffusion of Innovations, Fifth Edition, Free Press, New York, pp.221

29. Measuring Usability With The System Usability Scale (SUS), (2011) Available at: http://www.measuringu.com/sus.php, (Accessed: 13 May 2015)

30. Spook, J.E., et al., (2013), 'Monitoring Dietary Intake and Physical Activity Electronically: Feasibility, Usability, and Ecological Validity of a Mobile-Based Ecological Momentary Assessment Tool', Journal of Medical Internet Research, 15(9), pp. e214.

31. Cunningham, J.A., Kypros, K., McCambridge, J., (2013), 'Exploratory randomized controlled trial evaluating the impact of a waiting list control design', BMC Medical Research Methodology, 13(150)

32. Lancaster, G.A., Dodd, S., Williamson, PR., (2004), 'Design and analysis of pilot studies: recommendations for good practice', Journal of Evaluation in Clinical Practice, 10(2), pp.307-12

33. Teare, MD., Dimairo, M., Shephard, N., Hayman, A., Whitehead, A., Walters, SJ., (2014), ´Sample size requirements to estimate key design parameters from external pilot randomised controlled trials: a simulation study´, Trials, 15(1), pp.264.

34. Rabe-Hesketh, S., Skrondal, A., (2012), Multilevel and Longitudinal Modeling Using Stata, Third Edition, College Station, Texas: Stata Press.

For peer review only - http://bmjopen.bmj.com/site/about/guidelines.xhtml

Barts and The London
School of Medicine and Dentistry

35. Kahan BC, Jairath V, Dore CJ, Morris TP., (2014), ´The risks and rewards of covariate adjustment in randomized trials: an assessment of 12 outcomes from 8 studies´, Trials, 15, pp.139.

36. White, IR., Horton, NJ., Carpenter, J., Pocock, SJ., (2011), ´Strategy for intention to treat analysis in randomised trials with missing outcome data`, BMJ, 342:d40.

37. Medicines and Healthcare products Regulatory Agency (2014) Medical device stand-alone software including apps. Available at: https://www.gov.uk/government/publications/medical-devices-software-applications-apps/medical-device-stand-alone-software-including-apps (Accessed: 13 May 2015)

For peer review only - http://bmjopen.bmj.com/site/about/guidelines.xhtml

Barts and The London School of Medicine and Dentistry

APPENDICES

Appendix 1 – Information with regards to Safety Reporting in Non-CTIMP Research

	Who	When	How	To Whom
**SUSAR**	Chief Investigator	Report to the Sponsor, and QA manager within 24 hours<br>MREC within 15 days of learning of the event	SAE Report form for Non-CTIMPs, available from NRES website.	Sponsor and MREC
**Urgent Safety Measures**	Chief Investigator	Contact the Sponsor and MREC Immediately<br><br>Within 3 days	By phone<br><br>Substantial amendment form giving notice in writing setting out the reasons for the urgent safety measures and the plan for future action.	Main REC and Sponsor<br><br>Main REC with a copy also sent to the sponsor. The MREC will acknowledge this within 30 days of receipt.
**Progress Reports**	Chief Investigator	Annually (starting 12 months after the date of favourable opinion)	Annual Progress Report Form (non-CTIMPs) available from the NRES website	Main REC and Sponsor
**Declaration of the conclusion or early termination of the study**	Chief Investigator	Within 90 days (conclusion)<br><br>Within 15 days (early termination)<br><br>*The end of study should be defined in the protocol*	End of Study Declaration form available from the NRES website	Main REC with a copy to be sent to the sponsor
**Summary of final Report**	Chief Investigator	Within one year of conclusion of the Research	Where the study has met its objectives, the main findings and arrangements for publication or dissemination including feedback to participants	Main REC with a copy to be sent to the sponsor

For peer review only - http://bmjopen.bmj.com/site/about/guidelines.xhtml

Barts and The London School of Medicine and Dentistry

PCTU

MEMPHIS

MEMPHIS

Statistical Analysis Plan

Version: 3.0
Date: 26/Jan/2017

Person(s) contributing to the analysis plan	
Name(s) and position(s)	Neil Wright (Statistician)<br>Brennan Kahan (Statistician)<br>Elizabeth Ball (CI)<br>Gordon Forbes (Statistician)
**Authorisation**	
Position	**Chief or principal investigator**
Name	Elizabeth Ball
Signature	Elizabeth Ball
Date	26/01/2017
Position	**Senior trial statistician**
Name	Brennan Kahan
Signature	[illegible]
Date	26/01/2017
Position	**Independent statistician**
Name	Gian Luca Di Tanna
Tick once reviewed	✓
Date	30/01/2017

For peer review only - http://bmjopen.bmj.com/site/about/guidelines.xhtml

Barts and The London School of Medicine and Dentistry

PCTU

1. INTRODUCTION 3
2. STUDY METHODS 6
3. STUDY OUTCOMES 8
4. DATA COLLECTION 10
5. DERIVED VARIABLES 14
6. STATISTICAL ANALYSIS 15
7. GRAPHS AND FIGURES TO BE PRODUCED 19
8. REFERENCES 21
9. APPENDIX A: DERIVED AND COMPUTED VARIABLES 23
10. APPENDIX B: STATA CODE FOR GENERATING ADHERENCE OUTCOMES 29
11. APPENDIX C: STATA CODE FOR STATISTICAL ANALYSIS OF CLINICAL OUTCOMES ... 32
12. APPENDIX D: DRAFT TABLES 33
13. APPENDIX E: DATA / FILE MANAGEMENT 53

For peer review only - http://bmjopen.bmj.com/site/about/guidelines.xhtml

1. INTRODUCTION

1.1. Purpose of statistical analysis plan

The purpose of this document is to provide details of the statistical analyses and presentation of results to be reported within the principal paper(s) of the MEMPHIS trial. Any exploratory, post hoc or unplanned analyses will be clearly identified in the respective study analysis report. This document does not detail the qualitative analysis, and so aims and outcomes that are collected for qualitative analyses only are not included.

This document has been developed prior to examination of trial data and will not be implemented prior to final approval. Statisticians will be blinded to individual treatment allocations until this statistical analysis plan has been approved, all trial data has been collected and the trial is complete.

This document is based on protocol version 8.0 (December 2016)

1.2. Members of the writing committee

Neil Wright (Statistician) was primarily responsible for writing the Statistical Analysis Plan, with input from Brennan Kahan (Senior Statistician). Neil Wright was responsible for writing the computer code to implement the analysis strategy. Elizabeth Ball (CI) and Julie Dodds also contributed to this Statistical Analysis Plan.

1.3. Summary

Short Title	MEMPHIS
Methodology	A randomised feasibility trial
Research Sites	This trial will be conducted at the Royal London and Whipps Cross Hospitals
Objectives/Aims	The overall aim is to assess the feasibility of implementing a trial of a mindfulness meditation intervention delivered by a mobile phone app for patients with chronic pelvic pain (CPP). The primary objectives are:<br><br>To provide feasibility data for a large multicentre RCT aimed at rigorously testing mindfulness meditation in CPP<br><br>To determine whether this app can be seamlessly integrated into clinical practice, especially CPP pathways
Number of Participants/Patients	90 women with CPP will be recruited and each randomised into one of the three trial groups (meditation app, progressive muscle relaxation or no app).

For peer review only - http://bmjopen.bmj.com/site/about/guidelines.xhtml

Main Inclusion Criteria	To be eligible for the MEMPHIS study, the women must:<br>Be age 18 or over<br>Have either organic or non-organic chronic pelvic pain lasting for 6 months or more<br>Have access to a personal computer or smartphone.<br>Understand simple spoken English
Statistical Methodology and Analysis	Feasibility outcomes will be summarised using descriptive statistics. Clinical outcomes will be analysed using linear mixed-effects models, and results will be presented as a difference in means and a 95% confidence interval.

1.4. Changes from planned analysis in the protocol

- In the protocol, the dropout rate is a feasibility outcome but is not defined. In this analysis plan, we define two feasibility outcomes as “the number and proportion of participants who never return or answer a follow-up questionnaire at 6 months post-randomisation” and “the number and proportion of participants who do not return a follow-up questionnaire, but do answer the questionnaire by phone at 6 month post-randomisation”.

- In the protocol, duration of recruitment is described as “the number of days from the beginning to the end of recruitment”. In this analysis plan, duration of recruitment is defined as “the number of days from the day recruitment opens until the day the 90th patient is randomised (inclusive of both end days)”.

- In the protocol, “Sexual Health Outcomes score (as measured by Sexual Health Outcomes in Women Questionnaire (SHOW-Q))” is given as a clinical outcome. In this analysis plan, this is replaced by the SHOW-Q global score, for sexually active participants, and by the SHOW-Q pelvic interference score, for all participants.

1.5. Changes from SAP v1.0

- In section 1.4 of version 1.0 of the SAP we stated *“In the protocol, “Quality of life score (as measured by the RAND Short form (36) Health Survey (SF-36))” is given as a clinical outcome. In this analysis plan, this is replaced by four of the RAND SF-36 subscales: physical functioning, general health, social functioning, and pain.”* This has now been removed from the SAP as the protocol has been updated to reflect the change in the way quality of life score is being measured.

- The definition of app use has been changed from “having completed at least 50% of a session” to “having completed at least 90% of a session” (section 3.1). The change was

made due to Headspace, the data provider of the app usage data, only collecting data on sessions which were at least 90% complete.

1.6. Changes from SAP v2.0

- Added clarification to section 4.3 that data collected outside the recommended window for follow-up will still be included in analysis.
- In section 6.5.1, specified that the number of CRFs returned within the follow-up windows specified in section 4.3 will be summarised.
- Corrected scoring of CPAQ in Appendix A.
- Amended scoring of MYMOP in Appendix A so item scores are missing if the symptoms or activities are entered differently at follow up time points.

For peer review only - http://bmjopen.bmj.com/site/about/guidelines.xhtml

2. STUDY METHODS

2.1. Study objectives

The overall aim is to assess the feasibility of implementing a trial of a mindfulness meditation intervention delivered by a mobile phone app for patients with chronic pelvic pain (CPP). The primary objectives are:

- To provide feasibility data for a large multicentre RCT aimed at rigorously testing Mindfulness meditation in patients with CPP.
- To determine whether this app can be seamlessly integrated into clinical practice, especially CPP pathways.

2.2. Overall study design and plan

MEMPHIS is a randomised feasibility trial. Eligible women will be randomised to one of the three treatment groups:

- Intervention: 60 days of the app delivering mindfulness meditation content (in addition to usual care).
- Active control: 60 days of the app delivering progressive muscle relaxation content (in addition to usual care).
- Treatment as usual: Usual care

2.3. Selection of study population

2.3.1. Inclusion Criteria

To be eligible for the MEMPHIS study, the women must meet the following criteria:

- Aged 18 or over
- Women with organic and non-organic chronic pelvic pain lasting for six months or more
- Be capable of understanding the information provided, with use of an interpreter if required and being able to understand simple English as is used in the app
- Give written informed consent

2.3.2. Exclusion Criteria

Patients who meet the following criteria are ineligible to participate:

- No access to a Personal computer or smartphone

For peer review only - http://bmjopen.bmj.com/site/about/guidelines.xhtml

2.4. Method of treatment assignment and randomisation

After informed consent, patients will be randomised using a central, web-based system in a 1:1:1 ratio to one of the three treatment groups, using permuted blocks (of sizes 27, 30, 33) without stratification.

2.5. Sample size determination

30 participants will be recruited to each of the three treatment groups, giving a total of 90 participants. As this is a feasibility study, we have not performed a sample size calculation based upon the power to detect a significant treatment effect on a clinical outcome. However, 90 participants should provide a reliable estimate for the standard deviation of the primary clinical outcome (likely to be pain acceptance) [1, 2], which can be used to inform the sample size calculation of the main trial.

For peer review only - http://bmjopen.bmj.com/site/about/guidelines.xhtml

3. STUDY OUTCOMES

3.1. Feasibility outcomes

- Duration of recruitment (measured from the day recruitment opens until the day the 90th patient is randomised)
- Estimates to be used for the sample size calculation of the phase III RCT:
    - The estimated SD at 60 days, 3 months, and 6 months post-randomisation for pain acceptance (as measured by the Chronic Pain Acceptance Questionnaire (CPAQ-8))
    - The number and proportion of participants who never return or answer a follow-up questionnaire at 6 months post-randomisation.
    - The number and proportion of participants who do not return a follow-up questionnaire, but do answer the questionnaire by phone at 6 month post-randomisation.
- Patient adherence to app use measured by the following outcomes:
    - Number of days (within the first 60 days from randomisation) a patient has used the app (with app use defined as having completed at least 90%% of a session).
    - Whether the patient has used the app on 22 or more days within the first 60 days from randomisation.
    - Number of weeks (within the first eight weeks from randomisation) a patient has used the app on three or more days.
    - Whether the patient has used the app on three or more days in 6 or more weeks (within the first eight weeks from randomisation).
    - Whether the patient has used the app on 22 or more days within the first 60 days from randomisation, AND used the app on three or more days in 6 or more weeks within the first eight weeks from randomisation.

3.2. App satisfaction questionnaires

At 60 days post-randomisation:

- System Usability Scale (SUS) score (0 [worst] – 100 [best])
- Reponses to the purpose made app satisfaction questionnaire

For peer review only - http://bmjopen.bmj.com/site/about/guidelines.xhtml

3.3. Clinical outcomes

The following clinical outcomes at 60 days, 3 months, and 6 months post-randomisation:

- Pain acceptance score (as measured by the Chronic Pain Acceptance Questionnaire (CPAQ-8)) (0 [worst] – 48 [best])
- RAND Short form (36) Health Survey (RAND SF-36) scales:
    - Physical functioning (0 [worst] – 100 [best])
    - Pain (0 [worst] – 100 [best])
    - General health (0 [worst] – 100 [best])
    - Social functioning (0 [worst] – 100 [best])
- Depression score (as measured by the Hospital Anxiety and Depression Scale (HADS)) (0 [best] – 21 [worst])
- Anxiety score (as measured by HADS) (0 [best] – 21 [worst])
- Mindfulness score (as measure by the Cognitive and Mindfulness - Revised (CAMS – R) scale) (12 [worst] – 48 [best])
- Pain related disability score (as measured by the Chronic Pain Grade (CPG) disability subscale) (0 [best] – 100 [worst])
- Self efficacy score (as measured by the Pain Self-Efficacy Questionnaire (PSEQ)) (0 [worst] – 60 [best])
- Sexual Health Outcomes scores (as measured by the Sexual Health Outcomes in Women Questionnaire (SHOW-Q)):
    - SHOW-Q global score, for sexually active participants (0 [worst] – 100 [best])
    - SHOW-Q pelvic interference score, for all participants (0 [best] – 100 [worst])
- Subjective outcome score (as measured by the Measure Yourself Medical Outcome Profile (MYMOP)) (0 [best] – 6 [worst])

The following qualitative outcomes are not included in the Statistical Analysis Plan:

- Reasons for patient non-adherence to app use
- Obstacles to recruitment from participants and recruiting staff
- Usability/integration etc
- Determining primary/secondary outcomes of interest
- App satisfaction questionnaires for service providers

For peer review only - http://bmjopen.bmj.com/site/about/guidelines.xhtml

4. DATA COLLECTION

This section describes the variables that will be collected during the trial to be used in the analysis described by this plan.

4.1. Collected at baseline only

The following variables will be collected for each participant at baseline only.

Demographic:

- Age
- Weight
- Height
- Living arrangements (Alone, With others)
- Employment status (Employed (full or part time, including self-employment), Unemployed and looking for work, At school or in full time education, Unable to work due to long term sickness, Looking after your home/family, Retired from paid work, Other)
- Age left full time education (I did not receive a formal education, Age 12 or less, Age 13 to 16, Age 17 to 19, Age 20 or over, I am still in full time education, Other)
- Ethnic group (White, Black, Central Asian, Middle Eastern, Southern Asian, Mixed, Other ethnic group, Do not wish to say)
- Do you smoke (Yes, No)
- Number of cigarettes per week
- Do you drink alcohol (Yes, No)
- Number of alcohol units per week

Prior and concurrent treatment:

- Treatment used in last six months: Acupuncture; Gabapentin; Amitriptyline; Biofeedback; Botox injection; Contraceptive pills/patch/ring; Exercise, yoga or pilates; Injections to suppress ovaries (e.g. Prostap, Zoladex); Herbal Medicine; Meditation or relaxation exercises; Massage; Nutrition/diet; Codeine or Morphine type painkillers; Nerve blocks; Over the counter medication; Physiotherapy; Psychological (talking) therapy; Transcutaneous Electrical Nerve Stimulation (TENS); Surgery; Other. (One variable for each: Yes, No.)
- Currently using pain treatment (Yes, No)

Participants' pain:

For peer review only - http://bmjopen.bmj.com/site/about/guidelines.xhtml

- Length of pain (0-6 months, 7-12 months, 1-2 years, 3-5 years, 6-10 years, More than 10 years)
- Pain over the past week (0 [No pain] to 10 [Pain as bad as could be])

4.2. Randomisation details

The following variables for each participant will be held in the randomisation database.

- Date of randomisation
- Treatment group allocation

4.3. Collected at baseline and follow up

The following clinical outcome variables will be collected for each participant at baseline, 60 days, 3 months, and 6 months post-randomisation. We aim to collect 60 day follow up data between 46 and 74 days from randomisation, 3 month follow up date between 76 and 104 days and 6 month follow up data between 159 and 201 days. However, data collected outside these day ranges will be included in the analysis.

- Pain acceptance (as measured Chronic Pain Acceptance Questionnaire (CPAQ-8)) (4 variables)
- Short form (36) Health Survey (SF-36) (36 variables)
- Depression (as measured by the Hospital Anxiety and Depression Scale (HADS)) (7 variables)
- Anxiety (as measured by HADS) (7 variables)
- Mindfulness (as measure by the Cognitive and Mindfulness - Revised (CAMS – R) scale) (12 variables)
- Pain related disability (as measured by the Chronic Pain Grade (CPG) disability subscale) (3 variables)
- Self efficacy (as measured by the Pain Self-Efficacy Questionnaire (PSEQ)) (10 variables)
- Sexual Health Outcomes (as measured by Sexual Health Outcomes in Women Questionnaire (SHOW-Q)) (12 variables)
- Subjective outcome (as measured by Measure Yourself Medical Outcome Profile (MYMOP)) (4 variables)

For peer review only - http://bmjopen.bmj.com/site/about/guidelines.xhtml

Date of visit / date completed and method of collection (return of postal questionnaire or via telephone) for each follow-up questionnaire will also be collected. When the follow-up questionnaire is answered via telephone, the variables for the Short form (36) Health Survey (SF-36), Self efficacy (as measured by the Pain Self-Efficacy Questionnaire (PSEQ)), and Sexual Health Outcomes (as measured by Sexual Health Outcomes in Women Questionnaire (SHOW-Q)) are not collected.

4.4. App usage data

App usage data will be received from Headspace, for all participants randomised to the Intervention or Active Control arms. The data will include variables for participant login token, duration of session, filename of session, date and time of completion. Each observation represents one user completing (at least 90% of) a mindfulness meditation or muscle relaxation session.

4.5. App satisfaction questionnaires

The following variables will be collected for participants randomised to an app arm, at 60 days post-randomisation:

- System Usability Scale (SUS) (10 variables)
- Purpose made questionnaire responses:
    - Nine statements with categorical response. (Totally disagree, Somewhat disagree, Neither agree nor disagree, Somewhat agree, Totally agree) (9 variables)
    - One question (Did you use the app every day? (Yes, No))

4.6. Unintentional unblinding of randomised treatment

After the participant has been randomised, the following variables will be collected from the researcher:

- Was the participant randomised to the app treatment arm? (Yes, No)
- If the participant was allocated to the app treatment arm, which app treatment do you believe the participant was randomised to? (Intervention app, Control app, Don't know)

At 6 months (between 159 and 201 days) post-randomisation, the following variables will be collected from the participant:

- Did you use the smartphone app for MEMPHIS? (Yes, No)

For peer review only - http://bmjopen.bmj.com/site/about/guidelines.xhtml

Barts and The London School of Medicine and Dentistry

PCTU

- Do you think you received the new treatment or comparison treatment? (New Treatment, Comparison Treatment, Don't Know)

For peer review only - http://bmjopen.bmj.com/site/about/guidelines.xhtml

5. DERIVED VARIABLES

5.1. Feasibility outcomes

A participant is counted as never having returned follow-up questionnaire at 6 months post-randomisation if date of visit / date completed and all other fields in the follow-up questionnaire are missing.

The patient adherence to app use outcomes listed in Section 3.1 will be calculated from the app usage data described in Section 4.4. Completing a session that is at least ten minutes on a day counts as having used the app on that day. Sample Stata code showing the calculation of these outcome variables is given in APPENDIX B: STATA CODE FOR GENERATING ADHERENCE OUTCOMES.

In the app usage data, date and timestamps will be provided in Coordinated Universal Time (UTC). These will be converted to UK time (BST/GMT as appropriate) before outcomes are derived.

5.2. Clinical outcomes

Details for how the clinical outcome scores list in Section 3.3 are derived from question responses (Section 4.2) are given in APPENDIX A: DERIVED AND COMPUTED VARIABLES.

5.3. System Usability Score (SUS) score

Details for how the System Usability Scale (SUS) score is derived from question responses is given in APPENDIX A: DERIVED AND COMPUTED VARIABLES.

For peer review only - http://bmjopen.bmj.com/site/about/guidelines.xhtml

6. STATISTICAL ANALYSIS

6.1. Analysis populations

All analyses will be carried out according to the intention-to-treat (ITT) principle: all patients with a non-missing outcome will be analysed according to the group to which they are randomised.

Summaries of patient adherence to app use will include all participants randomised to the intervention or active control treatment groups.

Sample means and SDs for clinical outcomes will include all participants with a non-missing outcome at that time point.

Analyses to estimate treatment effects for clinical outcomes (Section 6.4.2) will include all patients with a non-missing outcome for at least one of the three follow-up time points (60 days, 3 months, or 6 months) [3]. Patients with a missing outcome at all follow-up time points for a clinical outcome are excluded from the analysis of that clinical outcome. A clinical outcome is non-missing if there are recorded responses at that time point for all individual questions required for the derivation of the clinical outcome. (Note that for the Subjective outcome score (MYMOP profile score), only symptom 1 score and wellbeing score are required.)

6.2. Baseline variables

Demographic, prior and concurrent treatment, and participants' pain baseline variables are listed in Section 4.1. Each variable (plus body mass index instead of height and weight) will be summarised for each treatment group by the mean (SD) or median (IQR) for continuous variables, and the number (%) for categorical variables. Draft tables are given in APPENDIX D: DRAFT TABLES.

6.3. Analysis of feasibility outcomes

Duration of recruitment will be stated. It is the number of days from the day recruitment opens until the day the 90th patient is randomised (inclusive of both end days).

The number of participants randomised in each one month period from the day recruitment opens will be presented.

The estimated SD in each treatment group at each follow-up time point for pain acceptance (as measured by the Chronic Pain Acceptance Questionnaire (CPAQ-8)) will be presented.

Each patient adherence to app use outcome listed in Section 3.1 will be summarised separately for the intervention and active control treatment groups. Each outcome will be presented as the mean (SD) or median (IQR) for continuous variables, and the number (%) for categorical variables. Draft tables are given in APPENDIX D: DRAFT TABLES.

For peer review only - http://bmjopen.bmj.com/site/about/guidelines.xhtml

Barts and The London School of Medicine and Dentistry

PCTU

6.4. Analysis of clinical outcomes

6.4.1. Descriptive statistics

For each clinical outcome listed in Section 3.3 we will present:

- The number of patients in each treatment group with a non-missing outcome at each time point.
- The mean (SD) in each treatment group at each time point.

6.4.2. Statistical analysis

For each clinical outcome we will present estimated treatment effects for each follow-up time point, with a 95% confidence interval. Estimates of treatment effects will be presented comparing the intervention group (mindfulness meditation app) to the control (treatment as usual) group, the intervention group to the active control (progressive muscle relaxation app) group, and the active control group to the control group.

Outcomes will be analysed using linear mixed-effects models with outcome measurement (at three follow-up time points) as the dependant variable. The model will include fixed time effects, a fixed effect for treatment, time treatment interactions for 3 months and 6 months follow-up time points, and an unstructured correlation matrix for the residuals [4]. The model will include baseline measure of the outcome as a covariate, assuming a linear relationship between baseline and outcome [5]. The model will be fitted using restricted maximum likelihood. Example Stata code for this analysis model is given in APPENDIX C: STATA CODE FOR STATISTICAL ANALYSIS OF CLINICAL OUTCOMES.

If there are missing values for baseline measure of a clinical outcome, they will be replaced by the mean of the observed baseline values for all participants in all treatment arms (mean imputation) [6]. Missing values of clinical outcomes at follow-up will not be imputed.

If the mixed effects models fail to converge, treatment effects will be estimated using separate linear regression models for each follow-up time point. Baseline measure of the outcome will be included as a covariate.

6.5. Other analyses

6.5.1. Comparison of losses to follow-up

The number and proportion of patients in each treatment group who have returned, answered by phone, or never returned the follow-up questionnaire will be presented for each follow-up time point (60 days, 3 months, and 6 months post-randomisation). A patient is counted as having returned data unless date of visit / date completed and all other fields in the follow-up questionnaire are missing. A draft table is given in APPENDIX D: DRAFT

TABLES.Summaries of the following baseline variables will be presented separately for patients who have returned, answered by phone, or never returned the follow-up questionnaire at the 6 month time point:

- Age at randomisation
- Body mass index
- Living arrangements
- Employment status
- Age left full time education
- Ethnic group
- Do you smoke
- Number of cigarettes per week
- Do you drink alcohol
- Number of units of alcohol per week
- Length of pain
- Pain over the past week
- Baseline values of clinical outcomes:
    - Pain acceptance score
    - Depression score
    - Anxiety score
    - Pain related disability score

6.5.2. Unintentional unblinding of randomised treatment

For each participants in the intervention and active control arm, researcher response to the question "If the participant was allocated to the app treatment arm, which app treatment do you believe the participant was randomised to?" will be summarised by number and percentage.

For participants in the intervention and active control arms, response to the question "Do you think you received the new treatment or comparison treatment?" will be summarised by number and percentage. A draft table is given in APPENDIX D: DRAFT TABLES.

For peer review only - http://bmjopen.bmj.com/site/about/guidelines.xhtml

6.5.3. Summarising missing data in clinical outcomes

For each clinical outcome variable we will present the number and proportion of individuals for whom the outcome is complete for at least one of the three follow-up time points (60 days, 3 months, or 6 months).

For each clinical outcome variable, we will also present the number and proportion of individuals for whom the outcome is not completed (either because the questionnaire was not returned, or because the participant left all variables for that outcome blank), partially completed (one or more, but not all, variables used in its derivation are missing), or complete (no variables used in its derivation are missing) at each time point.

Completely missing and partially missing outcomes will be summarised separately according to whether follow-up was completed via the mail-in questionnaire or over the phone.

6.5.1. Summarising data returned outside of target follow up periods

The number and proportion of patients in each treatment group who had follow up questionnaires completed within the time periods specified in section 4.3 will be presented for each follow up point. These are between 46 and 74 days for 60 days follow up, between 76 and 104 days for 3 month follow up, and between 159 and 201 days for 6 month follow up.

6.5.2. App usability

The mean (SD) of the System Usability Scale (SUS) score will be presented separately for the treatment app and active control app arms.

The number and proportion of each response for each question in the purpose made app satisfaction questionnaire will be presented separated for the treatment app and active control app arms. The number and proportion responding "Yes" to the question "Did you use the app every day?" will also be presented for each app arm.

6.5.3. Serious adverse events

We will present the number of reported serious adverse events in each treatment arm.

6.6. Analysis software

The analysis will be carried out using Stata.

7. GRAPHS AND FIGURES TO BE PRODUCED

7.1. Participant flow

Participant throughput will be summarized in a CONSORT diagram:

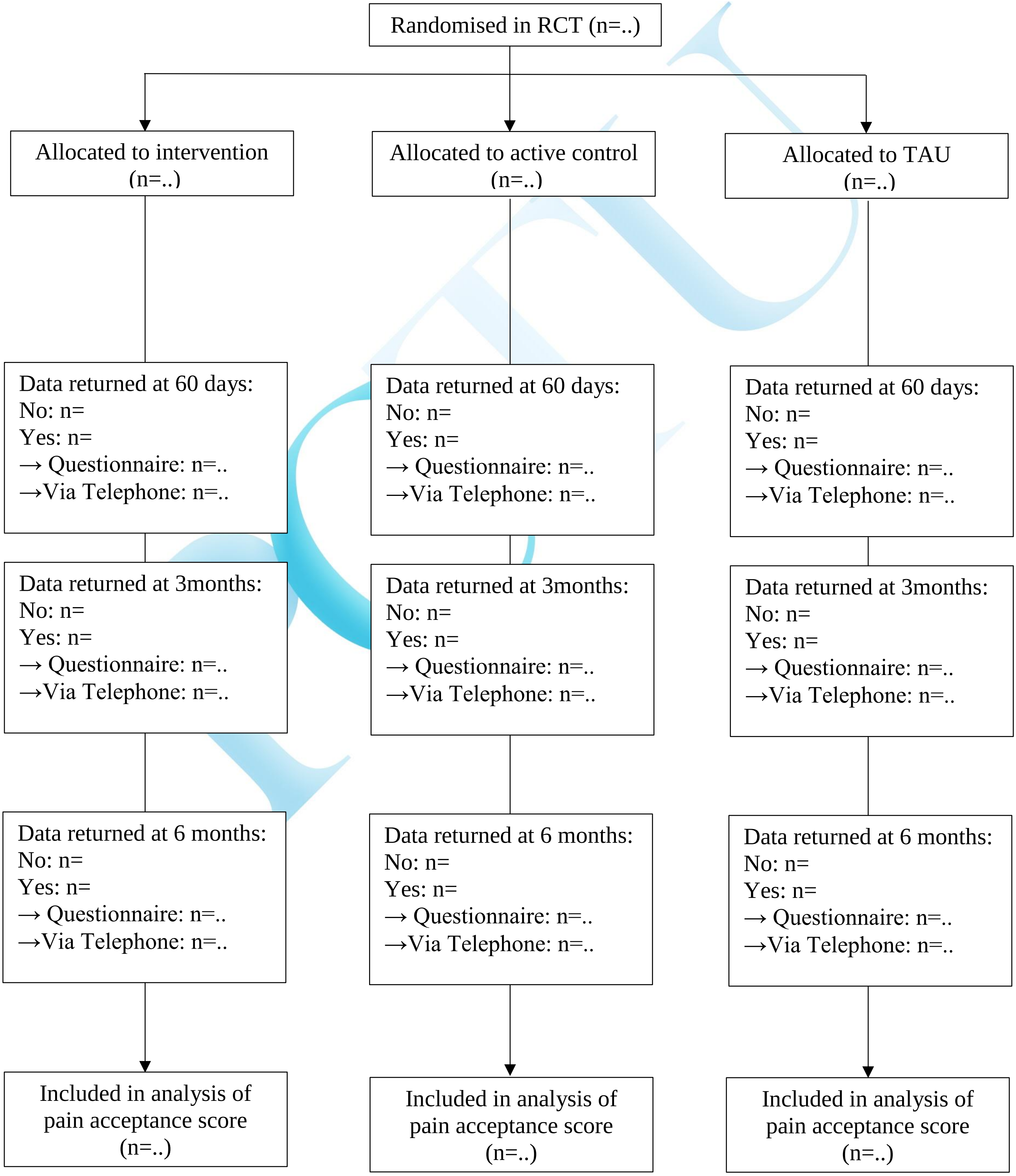

7.2. Graphs

The following graphs will be created:

- Line graph showing mean CPAQ score at each time point for each treatment group. The graph will also include lines showing 95% confidence intervals for each mean CPAQ score.
- Line graph showing all estimated treatment effects (and 95% confidence intervals) on CPAQ score for each follow-up time point. (Estimates of treatment effects will be presented comparing the intervention group (mindfulness meditation app) to the control (treatment as usual) group, the intervention group to the active control (progressive muscle relaxation app) group, and the active control group to the control group.)
- Stacked bar chart showing the proportion of participants in each treatment group who have returned the follow-up questionnaire or answered the follow-up questionnaire by phone at each follow-up time point (60 days, 3 months, and 6 months post-randomisation).

For peer review only - http://bmjopen.bmj.com/site/about/guidelines.xhtml

8. REFERENCES

[1] G. Lancaster, S. Dodd and P. Williamson, "Design and analysis of pilot studies: recommendations for good practice," *Journal of Evaluation in Clinical Practice,* vol. 10, no. 2, pp. 307-12, 2004.

[2] M. Teare, M. Dimairo, N. Shephard, A. Hayman, A. Whitehead and S. Walters, "Sample size requirements to estimate key design parameters from external pilot randomised controlled trials: a simulation study," *Trials*, vol. 15, p. 264, 2004.

[3] I. R. White, N. J. Horton, J. Carpenter and S. J. Pocock, "Strategy for intention to treat analysis in randomised trials with missing outcome data.," *BMJ (Clinical research ed.),* vol. 342, p. d40, 2011.

[4] S. Rabe-Hesketh and A. Skrondal, Multilevel and Longitudinal Modeling Using Stata. Volume I: Continuous Responses, Stata Press, 2012.

[5] B. C. Kahan, V. Jairath, C. J. Doré and T. P. Morris, "The risks and rewards of covariate adjustment in randomized trials: an assessment of 12 outcomes from 8 studies," *Trials,* vol. 15, p. 139, 2014.

[6] I. White and S. Thompson, "Adjusting for partially missing baseline measurements in randomized trials.," *Statistics in Medicine,* vol. 24, no. 7, pp. 993-1007, 2005.

[7] "RAND 36-Item Short Form Survey (SF-36) Scoring Instructions," [Online]. Available: https://www.rand.org/health/surveys_tools/mos/36-item-short-form/scoring.html.

[8] A. S. Zigmond and R. Snaith, "The hospital anxiety and depression scale.," *Acta psychiatrica Scandinavica,* no. 6, pp. 361-370, 1983.

[9] G. Feldman, A. Hayes, S. Kumar, J. Greeson and J.-P. Laurenceau, "Mindfulness and Emotion Regulation: The Development and Initial Validation of the Cognitive and Affective Mindfulness Scale-Revised (CAMS-R)," *Journal of Psychopathology and Behavioral Assessment,* vol. 29, p. 177–190, 2007.

[10] M. V. Korff, J. Ormel, F. J. Keefe and S. F. Dworkin, "Grading the severity of chronic pain," *Pain,* vol. 50, no. 2, pp. 133-149, 1992.

[11] M. K. Nicholas, "The pain self-efficacy questionnaire: Taking pain into account," *European Journal of Pain,* vol. 11, p. 153–163, 2007.

[12] R. A. Fish, B. McGuire, M. Hogan, T. G. Morrison and I. Stewart, "Validation of the Chronic Pain Acceptance Questionnaire (CPAQ) in an Internet," *Pain,* vol. 149, p. 435–443, 2010.

For peer review only - http://bmjopen.bmj.com/site/about/guidelines.xhtml

Barts and The London School of Medicine and Dentistry

PCTU

[13] [Online]. Available: http://www.bris.ac.uk/primaryhealthcare/resources/mymop/general-information/.

[14] [Online]. Available: https://www.usability.gov/how-to-and-tools/methods/system-usability-scale.html.

For peer review only - http://bmjopen.bmj.com/site/about/guidelines.xhtml

9. APPENDIX A: DERIVED AND COMPUTED VARIABLES

Unless otherwise stated, if an individual response variable used in the derivation of an outcome is missing then the outcome variable is missing.

Variables names used in the example code correspond to the field names specified in the trial database "Requirements Specification Document".

Body mass index

BMI is calculated as a person's weight (measured in kilograms) divided by the square of their height (measured in metres).

```
generate BMI = WEIGHT / ((HEIGHT / 100)^2)
```

RAND Short form (36) Health Survey (SF-36) scales scores [7]

Responses to individual questions are recoded as shown in the first table below. Each scale score is the average score for the questions in that scale, as shown in the second table below.

Item numbers	Original response code	Recode to
GH1, GH2, GH6, GH8, GH11b, GH11d	1	100
	2	75
	3	50
	4	25
	5	0
GH3a, GH3b, GH3c, GH3d, GH3e, GH3f, GH3g, GH3h, GH3i, GH3j	1	0
	2	50
	3	100
GH10, GH11a, GH11c	1	0
	2	25
	3	50
	4	75
	5	100
GH7	1	100
	2	80
	3	60
	4	40
	5	20
	6	0

Scale	After recoding, average the following items

Barts and The London School of Medicine and Dentistry PCTU

Physical functioning	GH3a, GH3b, GH3c, GH3d, GH3e, GH3f, GH3g, GH3h, GH3i, GH3j
Pain	GH7, GH8
General health	GH1, GH11a, GH11b, GH11c, GH11d
Social functioning	GH6, GH10

```
recode GH1 GH2 GH6 GH8 GH11b GH11d (1=100) (2=75) (3=50) (4=25)
(5=0)
recode GH3a GH3b GH3c GH3d GH3e GH3f Gh3g GH3h GH3i Gh3j (1=0)
(2=50) (3=100)
recode GH10 GH11a GH11c (1=0) (2=25) (3=50) (4=75) (5=100)
recode GH7 (1=100) (2=80) (3=60) (4=40) (5=20) (6=0)
generate SF36_PHYSICALFUNC = (GH3a + GH3b + GH3c + GH3d + GH3e
+ GH3f + GH3g + GH3h + GH3i + GH3j) / 10
generate SF36_SOCIALFUNC = (GH6 + GH10) / 2
generate SF36_PAIN = (GH7 + GH8) / 2
generate SF36_GENERALHEALTH = (GH1 + GH11a + GH11b + GH11c +
GH11d) / 5
```

Depression score (as measured by the Hospital Anxiety and Depression Scale (HADS)) [7]

After appropriate recoding, the HADS depression score is the sum of scores for questions 2, 4, 6, 8, 10, 12 and 14.

```
recode HADS02 HADS04 HADS12 HADS14 (1=0) (2=1) (3=2) (4=3)
recode HADS06 HADS08 HADS10 (1=3) (2=2) (3=1) (4=0)
generate HADS_DEPRESSION = HADS02 + HADS04 +HADS06 + HADS08 +
HADS10 + HADS12 + HADS14
```

Anxiety score (as measured by HADS) [7]

After appropriate recoding, the HADS anxiety score is the sum of scores for questions 1, 3, 5, 7, 9, 11 and 13.

```
recode HADS01 HADS03 HADS05 HADS11 HADS13 (1=3) (2=2) (3=1)
(4=0)
recode HADS07 HADS09 (1=0) (2=1) (3=2) (4=3)
generate HADS_ANXIETY = HADS01 + HADS03 + HADS05 + HADS07 +
HADS09 + HADS11 + HADS13
```

Mindfulness score (as measure by the Cognitive and Mindfulness - Revised (CAMS – R) scale) [8]

For peer review only - http://bmjopen.bmj.com/site/about/guidelines.xhtml

After appropriate recording, the CAMS-R mindfulness score is the sum of scores for all questions 1 to 12.

```
recode CAMSR02 CAMSR06 CAMSR07 (1=4) (2=3) (3=2) (4=1)
generate CAMSR_SCORE = CAMSR01 + CAMSR02 + CAMSR03 + CAMSR04 +
CAMSR05 + CAMSR06 + CAMSR07 + CAMSR08 + CAMSR09 + CAMSR10 +
CAMSR11 + CAMSR12
```

<u>Pain related disability score (as measured by the Chronic Pain Grade (CPG) disability subscale)</u> [9]

THE CPG pain related disability score is the mean of the daily activities, social activities, and work activities scores, multiplied by 10.

```
generate CPG_DISABILITYSCORE = [(CPGd1 + CPGd2 + CPGd3) / 3 ] *
10
```

<u>Self efficacy score (as measured by the Pain Self-Efficacy Questionnaire (PSEQ))</u> [10]

The PSEQ self efficacy score is the sum of scores for all questions 1 to 10.

```
generate PSEQ_SCORE = PSEQ01 + PSEQ02 + PSEQ03 + PSEQ04 + PSEQ05
+ PSEQ06 + PSEQ07 + PSEQ08 + PSEQ09 + PSEQ10
```

<u>Pain acceptance score (as measured Chronic Pain Acceptance Questionnaire (CPAQ-8))</u> [12]

After reverse scoring, the CPAQ-8 pain willingness score is the sum of scores from questions 4, 5, 7 and 8. The CPAQ-8 activity engagement score is the sum of scores from questions 1, 2, 3, 5 and 6. The CPAQ-8 total score is the sum of the pain willingness score and the activity engagement score.

```
recode CPAQ CPAQ4 CPAQ5 CPAQ7 CPAQ8 (0=6) (1=5) (2=4) (3=3)
(4=2) (5=1) (6=0)
generate CPAQ_PAINWILL = CPAQ4 + CPAQ5 + CPAQ7 + CPAQ8
generate CPAQ_ACTIVITYENG = CPAQ1 + CPAQ2 + CPAQ3 + CPAQ6
generate CPAQ_TOTAL = CPAQ_PAINWILL + CPAQ_ACTIVITYENG
```

<u>Sexual Health Outcomes score (as measured by Sexual Health Outcomes in Women</u>

For peer review only - http://bmjopen.bmj.com/site/about/guidelines.xhtml

Barts and The London School of Medicine and Dentistry PCTU

Questionnaire (SHOW-Q))

Each response is rescaled to a score 0 to 100, with higher scores reflecting higher sexual functioning or fewer sexual problems. For a 5 response item, the scores are 0, 25, 50, 75 or 100. For a 4 response item, the scores are 0, 33.3, 66.7 or 100. The scoring for each question is shown in the table below.

If a participant answers “I don’t have a partner” or “I don’t have sex without a partner” to question 2 or “I did not have sexual activity” to any of questions 3, 4, 6, 7 or 9, then the participant is classed as sexually inactive. Otherwise, the participant is classed as sexually active.

For sexually active participants, the SHOW-Q global score is calculated as the mean of all rescaled scores. Higher scores reflect higher sexual functioning or fewer sexual problems.

For all participants, the SHOW-Q pelvic problem interference score is the mean of response scores to questions 10, 11 and 12 after they are reverse scored. Higher scores reflect more interference.

Item number	Response text	Original response code	Recode to
SHOWQ01, SHOWQ02	Very satisfied	1	100
	Somewhat satisfied	2	75
	Neither satisfied nor dissatisfied	3	50
	Somewhat dissatisfied	4	25
	Very dissatisfied	5	0
SHOWQ10, SHOWQ11, SHOWQ12	Not at all	1	100
	Slightly	2	75
	Moderately	3	50
	Quite a bit	4	25
	Extremely	5	0
SHOWQ03, SHOWQ04	Never	1	0
	Rarely	2	25
	Sometimes	3	50
	Most of the time	4	75
	All of the time	5	100
SHOWQ08	Never	1	0
	Once or twice	2	25
	3-4 times	3	50
	5-6 times	4	75
	More than 6 times	5	100
SHOWQ05	Did not experience any orgasms	1	0
	Mild	2	33.3
	Moderate	3	66.7
	Strong	4	100
SHOWQ06,	Not a problem	1	100

For peer review only - http://bmjopen.bmj.com/site/about/guidelines.xhtml

Barts and The London School of Medicine and Dentistry PCTU

SHOWQ07, SHOWQ09	Little of a problem	2	66.7
	Somewhat of a problem	3	33.3
	Very much of a problem	4	0

```
generate SHOWQ_ACTIVE = 1
replace SHOWQ_ACTIVE = 0 if SHOWQ02==6 | SHOWQ02==7 | SHOWQ03==6
| SHOW04==6 | SHOWQ06==5 | SHOWQ07==5 | SHOWQ09== 5
recode SHOWQ01 SHOWQ02 SHOW10 SHOWQ11 SHOWQ12 (1=100) (2=75)
(3=50) (4=25) (5=0)
recode SHOWQ03 SHOWQ04 SHOWQ08 (1=0) (2=25) (3=50) (4=75)
(5=100)
recode SHOWQ05 (1=0) (2=33.3) (3=66.7) (4=100)
recode SHOWQ06 SHOWQ07 SHOWQ09 (1=100) (2=66.7) (3=33.3) (4=0)
generate SHOWQ_GLOBAL = (SHOWQ01 + SHOWQ02 + SHOWQ03 + SHOWQ04
+ SHOWQ05 + SHOWQ06 + SHOWQ07 + SHOWQ08 + SHOWQ09 + SHOWQ10 +
SHOWQ11 + SHOWQ12)/12 if SHOWQ_ACTIVE == 1
generate SHOWQ_PELVPROBLEM = ((100 - SHOWQ10) + (100 - SHOWQ11)
+ (100 - SHOWQ12))/3
```

<u>Subjective outcome score (as measured by Measure Yourself Medical Outcome Profile (MYMOP)) [12]</u>

If the description for symptom 1, symptom 2, symptom 3 or activity does not match the description given for the corresponding symptom or activity at baseline then the score for that symptom or activity is missing.

If symptom 1 score or wellbeing score are missing, then MYMOP profile score is missing.The MYMOP profile score is the mean of the symptom 1 score, symptom 2 score, activity score, wellbeing score, and symptom 3 score. (Symptom 2 score, activity score and symptom 3 score are only included if they are not missing)

```
egen MYMOP_PROFILE = rowmean(SYMSCORE1, SYMSCORE2, ACTSCORE,
WELLBEING, SYMSCORE3)
```

<u>System Usability Scale (SUS) score [13]</u>

For questions 1, 3, 5, 7, and 9 the score contribution is the response number minus 1. For questions 2, 4, 6, 8, and 10 the score contribution is 5 minus the response number. The SUS score is the sum of all score contributions multiplied by 2.5

```
recode SUS01 SUS03 SYS05 SUS07 SUS09 (1 = 0) (2 = 1) (3 = 2) (4 = 3) (5 = 4)
recode SUS02 SUS04 SUS06 SUS08 SUS10 (1 = 4) (2 = 3) (3 = 2) (4 = 1) (5 = 0)
generate SUS_SCORE  = 2.5 * (SUS01 + SUS02 + SUS03 + SUS04 + SUS05 +SUS06 + SUS07 + SUS08 + SUS9 + SUS10)
```

Adherence outcomes

countin60days	Number of days (within the first 60 days from randomisation) a patient has used the app (with app use defined as having completed at least 90% of a session).	
numberofweeksthreeplus	Number of weeks (within the first eight weeks from randomisation) a patient has used the app on three or more days.	
adhere_countin60days	Whether the patient has used the app on 22 or more days within the first 60 days from randomisation.	1 = Yes<br>0 = No
adhere_numberofweeksthreeplus	Whether the patient has used the app on three or more days in 6 or more weeks (within the first eight weeks from randomisation).	1 = Yes<br>0 = No

Sample Stata code showing the calculation of these outcome variables is given in APPENDIX B: STATA CODE FOR GENERATING ADHERENCE OUTCOMES.

For peer review only - http://bmjopen.bmj.com/site/about/guidelines.xhtml

10. APPENDIX B: STATA CODE FOR GENERATING ADHERENCE OUTCOMES

Sample of Stata code for generating adherence outcomes from app usage data supplied by Headspace:

```
gen date_completed = date(datecompleted, "DMY")

format date_completed %td


gen date_rand = date(dateofrandomisation, "DMY")

format date_rand %td


gen date_fromrand = date_completed-date_rand


***


* Drop sessions which are not part of intervention (i.e. short duration)

drop if duration<5


* Remove multiple sessions in same day

duplicates report id date_fromrand
```

For peer review only - http://bmjopen.bmj.com/site/about/guidelines.xhtml

```
duplicates drop id date_fromrand , force


gen in60days = 1 if date_fromrand<61

bysort id: egen countin60days = count(in60days)


gen numberofweeksthreeplus = 0


forvalues week=1/8 {

     gen inweek`week' = 1 if date_fromrand>7*(`week'-1) & date_fromrand<7*`week'+1

     gen threeplusinweek`week' = 0

     bysort id: egen countinweek`week' = count(inweek`week')

     assert countinweek`week'<8

     bysort id: replace threeplusinweek`week' = 1 if countinweek`week'>2

     bysort id: replace numberofweeksthreeplus = numberofweeksthreeplus +1 if countinweek`week'>2

}
```

For peer review only - http://bmjopen.bmj.com/site/about/guidelines.xhtml

```
bysort id: keep if _n==1

keep id countin60days numberofweeksthreeplus threeplusinweek* countinweek*


gen adhere_countin60days = 0

replace adhere_countin60days = 1 if countin60days>21

gen adhere_numberofweeksthreeplus = 0

replace adhere_numberofweeksthreeplus = 1 if numberofweeksthreeplus>5


tab adhere_countin60days adhere_numberofweeksthreeplus
```

For peer review only - http://bmjopen.bmj.com/site/about/guidelines.xhtml

11. APPENDIX C: STATA CODE FOR STATISTICAL ANALYSIS OF CLINICAL OUTCOMES

The following Stata shows the model that will be used to estimate treatment effects on clinical outcomes:

```
xtmixed outcome time##treat baseline || id: , noconstant
residuals(unstructured, t(time)) var reml
```

Estimates of treatment effects for each treatment arm comparison and time point will then be obtained using:

```
lincom 1.treat + 1.time#1.treat

lincom 1.treat + 2.time#1.treat

lincom 1.treat + 3.time#1.treat

lincom 2.treat + 1.time#2.treat

lincom 2.treat + 2.time#2.treat

lincom 2.treat + 3.time#2.treat

lincom 2.treat + 1.time#2.treat - 1.treat + 1.time#1.treat

lincom 2.treat + 2.time#2.treat - 1.treat + 2.time#1.treat

lincom 2.treat + 3.time#2.treat - 1.treat + 3.time#1.treat
```

For peer review only - http://bmjopen.bmj.com/site/about/guidelines.xhtml

12. APPENDIX D: DRAFT TABLES

12.1.1. Baseline demographics and medical history

Figures are mean (SD) unless stated otherwise.

	Intervention (n=…)	Active control (n=…)	Usual care (n=…)
**Demographics**			
Age at randomisation (Years)	XX (XX)	XX (XX)	XX (XX)
Body mass index	XX (XX)	XX (XX)	XX (XX)
Living arrangements – no. (%)			
Alone	XX (XX)	XX (XX)	XX (XX)
With others	XX (XX)	XX (XX)	XX (XX)
Employment status – no. (%)			
Employed	XX (XX)	XX (XX)	XX (XX)
Unemployed and looking for work	XX (XX)	XX (XX)	XX (XX)
At school or in full time education	XX (XX)	XX (XX)	XX (XX)
Unable to work due to long term sickness	XX (XX)	XX (XX)	XX (XX)
Look after your home/family	XX (XX)	XX (XX)	XX (XX)
Retired from paid work	XX (XX)	XX (XX)	XX (XX)
Other	XX (XX)	XX (XX)	XX (XX)
Age left full time education – no. (%)			
I did not receive a formal education	XX (XX)	XX (XX)	XX (XX)
Age 12 or less	XX (XX)	XX (XX)	XX (XX)
Age 13 to 16	XX (XX)	XX (XX)	XX (XX)
Age 17 to 19	XX (XX)	XX (XX)	XX (XX)
Age 20 or over	XX (XX)	XX (XX)	XX (XX)
I am still in full time education	XX (XX)	XX (XX)	XX (XX)
Other	XX (XX)	XX (XX)	XX (XX)
Ethnic group – no. (%)			
White	XX (XX)	XX (XX)	XX (XX)
Black	XX (XX)	XX (XX)	XX (XX)
Central Asian	XX (XX)	XX (XX)	XX (XX)
Middle Eastern	XX (XX)	XX (XX)	XX (XX)
Southern Asian	XX (XX)	XX (XX)	XX (XX)
Mixed	XX (XX)	XX (XX)	XX (XX)
Other ethnic group	XX (XX)	XX (XX)	XX (XX)
Do not wish to say	XX (XX)	XX (XX)	XX (XX)
Smoker – no. (%)			
Yes	XX (XX)	XX (XX)	XX (XX)
No	XX (XX)	XX (XX)	XX (XX)
If yes, number of cigarettes per week	XX (XX)	XX (XX)	XX (XX)
Drink alcohol – no. (%)			

For peer review only - http://bmjopen.bmj.com/site/about/guidelines.xhtml

Barts and The London School of Medicine and Dentistry PCTU

Yes	XX	(XX)	XX	(XX)	XX	(XX)
No	XX	(XX)	XX	(XX)	XX	(XX)
If yes, number of units of alcohol per week	XX	(XX)	XX	(XX)	XX	(XX)
**Baseline medical history**						
Length of pain – no. (%)						
0-6 months	XX	(XX)	XX	(XX)	XX	(XX)
7-12 months	XX	(XX)	XX	(XX)	XX	(XX)
1-2 years	XX	(XX)	XX	(XX)	XX	(XX)
3-5 years	XX	(XX)	XX	(XX)	XX	(XX)
6-10 years	XX	(XX)	XX	(XX)	XX	(XX)
More than 10 years	XX	(XX)	XX	(XX)	XX	(XX)
Pain over the past week	XX	(XX)	XX	(XX)	XX	(XX)

12.1.2. Prior and concurrent treatment

Figures are number (percentage).

	Intervention (n=…)		Active control (n=…)		Usual care (n=…)	
Treatment used in last six months						
Acupuncture	XX	(XX)	XX	(XX)	XX	(XX)
Gabapentin	XX	(XX)	XX	(XX)	XX	(XX)
Amitriptyline	XX	(XX)	XX	(XX)	XX	(XX)
Biofeedback	XX	(XX)	XX	(XX)	XX	(XX)
Botox injection	XX	(XX)	XX	(XX)	XX	(XX)
Contraceptive pills/patch/ring	XX	(XX)	XX	(XX)	XX	(XX)
Exercise, yoga or pilates	XX	(XX)	XX	(XX)	XX	(XX)
Injections to suppress ovaries (e.g. Prostap, Zoladex)	XX	(XX)	XX	(XX)	XX	(XX)
Herbal Medicine	XX	(XX)	XX	(XX)	XX	(XX)
Meditation or relaxation exercises	XX	(XX)	XX	(XX)	XX	(XX)
Massage	XX	(XX)	XX	(XX)	XX	(XX)
Nutrition/diet	XX	(XX)	XX	(XX)	XX	(XX)
Codeine or Morphine type painkillers	XX	(XX)	XX	(XX)	XX	(XX)
Nerve blocks	XX	(XX)	XX	(XX)	XX	(XX)
Over the counter medication	XX	(XX)	XX	(XX)	XX	(XX)
Physiotherapy	XX	(XX)	XX	(XX)	XX	(XX)
Psychological (talking) therapy	XX	(XX)	XX	(XX)	XX	(XX)
Transcutaneous Electrical Nerve Stimulation (TENS)	XX	(XX)	XX	(XX)	XX	(XX)
Surgery	XX	(XX)	XX	(XX)	XX	(XX)

For peer review only - http://bmjopen.bmj.com/site/about/guidelines.xhtml

	Intervention	Active control	Usual care
Other	XX (XX)	XX (XX)	XX (XX)
Currently using pain treatment			
Yes	XX (XX)	XX (XX)	XX (XX)
No	XX (XX)	XX (XX)	XX (XX)

12.1.3. Baseline values of clinical outcomes

Figures are mean (SD) unless stated otherwise.

	Intervention (n=…)	Active control (n=…)	Usual care (n=…)
SF-36 scales:			
Physical functioning	XX (XX)	XX (XX)	XX (XX)
Pain	XX (XX)	XX (XX)	XX (XX)
General Health	XX (XX)	XX (XX)	XX (XX)
Social Functioning	XX (XX)	XX (XX)	XX (XX)
Depression score	XX (XX)	XX (XX)	XX (XX)
Anxiety score	XX (XX)	XX (XX)	XX (XX)
Mindfulness score	XX (XX)	XX (XX)	XX (XX)
Pain related disability score	XX (XX)	XX (XX)	XX (XX)
Self efficacy score	XX (XX)	XX (XX)	XX (XX)
Pain acceptance score	XX (XX)	XX (XX)	XX (XX)
Sexual health outcomes:			
SHOW-Q global score	XX (XX)	XX (XX)	XX (XX)
SHOW-Q pelvic problem interference score	XX (XX)	XX (XX)	XX (XX)
Subjective outcome score	XX (XX)	XX (XX)	XX (XX)

12.1.4. Loss to follow-up

	Intervention (n=…)	Active control (n=…)	Usual care (n=…)
Follow-up questionnaire returned – no. (%)			
60 days	XX (XX)	XX (XX)	XX (XX)
3 months	XX (XX)	XX (XX)	XX (XX)
6 months	XX (XX)	XX (XX)	XX (XX)
Follow-up questionnaire answered by phone – no. (%)			
60 days	XX (XX)	XX (XX)	XX (XX)
3 months	XX (XX)	XX (XX)	XX (XX)
6 months	XX (XX)	XX (XX)	XX (XX)
Follow-up questionnaire never returned – no. (%)			
60 days	XX (XX)	XX (XX)	XX (XX)
3 months	XX (XX)	XX (XX)	XX (XX)
6 months	XX (XX)	XX (XX)	XX (XX)

For peer review only - http://bmjopen.bmj.com/site/about/guidelines.xhtml

12.1.5. Loss to follow-up

Figures are mean (SD) unless stated otherwise.

	6 months follow-up questionnaire returned (n=…)	6 months follow-up questionnaire answered by phone (n=…)	6 months follow-up questionnaire never returned (n=…)
**Demographics**			
Age at randomisation (Years)	XX (XX)	XX (XX)	XX (XX)
Body mass index	XX (XX)	XX (XX)	XX (XX)
Living arrangements – no. (%)			
Alone	XX (XX)	XX (XX)	XX (XX)
With others	XX (XX)	XX (XX)	XX (XX)
Employment status – no. (%)			
Employed	XX (XX)	XX (XX)	XX (XX)
Unemployed and looking for work	XX (XX)	XX (XX)	XX (XX)
At school or in full time education	XX (XX)	XX (XX)	XX (XX)
Unable to work due to long term sickness	XX (XX)	XX (XX)	XX (XX)
Look after your home/family	XX (XX)	XX (XX)	XX (XX)
Retired from paid work	XX (XX)	XX (XX)	XX (XX)
Other	XX (XX)	XX (XX)	XX (XX)
Age left full time education – no. (%)			
I did not receive a formal education	XX (XX)	XX (XX)	XX (XX)
Age 12 or less	XX (XX)	XX (XX)	XX (XX)
Age 13 to 16	XX (XX)	XX (XX)	XX (XX)
Age 17 to 19	XX (XX)	XX (XX)	XX (XX)
Age 20 or over	XX (XX)	XX (XX)	XX (XX)
I am still in full time education	XX (XX)	XX (XX)	XX (XX)
Other	XX (XX)	XX (XX)	XX (XX)
Ethnic group – no. (%)			
White	XX (XX)	XX (XX)	XX (XX)
Black	XX (XX)	XX (XX)	XX (XX)
Central Asian	XX (XX)	XX (XX)	XX (XX)
Middle Eastern	XX (XX)	XX (XX)	XX (XX)
Southern Asian	XX (XX)	XX (XX)	XX (XX)
Mixed	XX (XX)	XX (XX)	XX (XX)
Other ethnic group	XX (XX)	XX (XX)	XX (XX)
Do not wish to say	XX (XX)	XX (XX)	XX (XX)
Smoker – no. (%)			

For peer review only - http://bmjopen.bmj.com/site/about/guidelines.xhtml

Yes	XX (XX)	XX (XX)	XX (XX)
No	XX (XX)	XX (XX)	XX (XX)
If yes, number of cigarettes per week	XX (XX)	XX (XX)	XX (XX)
Drink alcohol – no. (%)			
Yes	XX (XX)	XX (XX)	XX (XX)
No	XX (XX)	XX (XX)	XX (XX)
If yes, number of units of alcohol per week	XX (XX)	XX (XX)	XX (XX)
**Baseline medical history**			
Length of pain – no. (%)			
0-6 months	XX (XX)	XX (XX)	XX (XX)
7-12 months	XX (XX)	XX (XX)	XX (XX)
1-2 years	XX (XX)	XX (XX)	XX (XX)
3-5 years	XX (XX)	XX (XX)	XX (XX)
6-10 years	XX (XX)	XX (XX)	XX (XX)
More than 10 years	XX (XX)	XX (XX)	XX (XX)
Pain over the past week	XX (XX)	XX (XX)	XX (XX)
**Baseline values of clinical outcomes**			
Pain acceptance score (CPAQ-8)	XX (XX)	XX (XX)	XX (XX)
Depression score (HADS)	XX (XX)	XX (XX)	XX (XX)
Anxiety score (HADS)	XX (XX)	XX (XX)	XX (XX)
Pain related disability score (CPG)	XX (XX)	XX (XX)	XX (XX)

12.1.6. Follow up within target follow up period

	Intervention (n=…)	Active control (n=…)	Usual care (n=…)
Follow-up questionnaire returned or answered by phone within target follow up period– no. (%)			
60 days (46 and 74days)	XX (XX)	XX (XX)	XX (XX)
3 months (76 and 104 days)	XX (XX)	XX (XX)	XX (XX)
6 months (159 and 201 days)	XX (XX)	XX (XX)	XX (XX)

12.1.7. Adherence to app use

Figures are mean (SD) unless stated otherwise.

	Intervention (n=…)	Active control (n=…)
Number of days (within the first 60 days from randomisation) a patient has used the app	XX (XX)	XX (XX)

For peer review only - http://bmjopen.bmj.com/site/about/guidelines.xhtml

Number of weeks (within the first eight weeks from randomisation) a patient has used the app on three or more days	XX (XX)	XX (XX)
Used the app on 22 or more days within the first 60 days from randomisation – no. (%)	XX (XX)	XX (XX)
Used the app on three or more days in 6 or more weeks (within the first eight weeks from randomisation) – no. (%)	XX (XX)	XX (XX)
Used the app on 22 or more days within the first 60 days from randomisation, AND used the app on three or more days in 6 or more weeks within the first eight weeks from randomisation – no. (%)	XX (XX)	XX (XX)

12.1.8. App usability questionnaire

Figures are number (percentage).

	Totally disagree	Somewhat disagree	Neither agree nor disagree	Somewhat agree	Totally agree	Not answered
It is easy to access the app whenever I wanted to use it						
Intervention:	XX (XX)	XX (XX)	XX (XX)	XX (XX)	XX (XX)	XX (XX)
Active control:	XX (XX)	XX (XX)	XX (XX)	XX (XX)	XX (XX)	XX (XX)
After being shown, I understood how the app would work						
Intervention:	XX (XX)	XX (XX)	XX (XX)	XX (XX)	XX (XX)	XX (XX)
Active control:	XX (XX)	XX (XX)	XX (XX)	XX (XX)	XX (XX)	XX (XX)
It was fun to work with the app						
Intervention:	XX (XX)	XX (XX)	XX (XX)	XX (XX)	XX (XX)	XX (XX)
Active control:	XX (XX)	XX (XX)	XX (XX)	XX (XX)	XX (XX)	XX (XX)
The app worked well						
Intervention:	XX (XX)	XX (XX)	XX (XX)	XX (XX)	XX (XX)	XX (XX)
Active control:	XX (XX)	XX (XX)	XX (XX)	XX (XX)	XX (XX)	XX (XX)
It was easy to work through the modules						
Intervention:	XX (XX)	XX (XX)	XX (XX)	XX (XX)	XX (XX)	XX (XX)
Active control:	XX (XX)	XX (XX)	XX (XX)	XX (XX)	XX (XX)	XX (XX)

For peer review only - http://bmjopen.bmj.com/site/about/guidelines.xhtml

Barts and The London School of Medicine and Dentistry PCTU

The number of modules was annoying						
Intervention:	XX (XX)	XX (XX)	XX (XX)	XX (XX)	XX (XX)	XX (XX)
Active control:	XX (XX)	XX (XX)	XX (XX)	XX (XX)	XX (XX)	XX (XX)
The modules were well-displayed on my smartphone						
Intervention:	XX (XX)	XX (XX)	XX (XX)	XX (XX)	XX (XX)	XX (XX)
Active control:	XX (XX)	XX (XX)	XX (XX)	XX (XX)	XX (XX)	XX (XX)
Using the app was difficult because of my daily activities						
Intervention:	XX (XX)	XX (XX)	XX (XX)	XX (XX)	XX (XX)	XX (XX)
Active control:	XX (XX)	XX (XX)	XX (XX)	XX (XX)	XX (XX)	XX (XX)
Using the app took too long						
Intervention:	XX (XX)	XX (XX)	XX (XX)	XX (XX)	XX (XX)	XX (XX)
Active control:	XX (XX)	XX (XX)	XX (XX)	XX (XX)	XX (XX)	XX (XX)

PCTU

Barts and The London School of Medicine and Dentistry PCTU

12.1.9. Clinical outcomes

	Intervention (n=…)				Active control (n=…)				Usual care (n=…)			
	n	(%)	Mean	(SD)	n	(%)	Mean	(SD)	n	(%)	Mean	(SD)
**Pain acceptance score**												
Baseline	XX	(XX)	XX	(XX)	XX	(XX)	XX	(XX)	XX	(XX)	XX	(XX)
60 days	XX	(XX)	XX	(XX)	XX	(XX)	XX	(XX)	XX	(XX)	XX	(XX)
3 months	XX	(XX)	XX	(XX)	XX	(XX)	XX	(XX)	XX	(XX)	XX	(XX)
6 months	XX	(XX)	XX	(XX)	XX	(XX)	XX	(XX)	XX	(XX)	XX	(XX)
Included in analysis †	XX	(XX)			XX	(XX)			XX	(XX)		
**Depression score**												
Baseline	XX	(XX)	XX	(XX)	XX	(XX)	XX	(XX)	XX	(XX)	XX	(XX)
60 days	XX	(XX)	XX	(XX)	XX	(XX)	XX	(XX)	XX	(XX)	XX	(XX)
3 months	XX	(XX)	XX	(XX)	XX	(XX)	XX	(XX)	XX	(XX)	XX	(XX)
6 months	XX	(XX)	XX	(XX)	XX	(XX)	XX	(XX)	XX	(XX)	XX	(XX)
Included in analysis †	XX	(XX)			XX	(XX)			XX	(XX)		
**Anxiety score**												
Baseline	XX	(XX)	XX	(XX)	XX	(XX)	XX	(XX)	XX	(XX)	XX	(XX)
60 days	XX	(XX)	XX	(XX)	XX	(XX)	XX	(XX)	XX	(XX)	XX	(XX)
3 months	XX	(XX)	XX	(XX)	XX	(XX)	XX	(XX)	XX	(XX)	XX	(XX)
6 months	XX	(XX)	XX	(XX)	XX	(XX)	XX	(XX)	XX	(XX)	XX	(XX)
Included in analysis †	XX	(XX)			XX	(XX)			XX	(XX)		

For peer review only - http://bmjopen.bmj.com/site/about/guidelines.xhtml

**Mindfulness score**												
Baseline	XX	(XX)	XX	(XX)	XX	(XX)	XX	(XX)	XX	(XX)	XX	(XX)
60 days	XX	(XX)	XX	(XX)	XX	(XX)	XX	(XX)	XX	(XX)	XX	(XX)
3 months	XX	(XX)	XX	(XX)	XX	(XX)	XX	(XX)	XX	(XX)	XX	(XX)
6 months	XX	(XX)	XX	(XX)	XX	(XX)	XX	(XX)	XX	(XX)	XX	(XX)
Included in analysis †	XX	(XX)			XX	(XX)			XX	(XX)		
**Pain related disability score**												
Baseline	XX	(XX)	XX	(XX)	XX	(XX)	XX	(XX)	XX	(XX)	XX	(XX)
60 days	XX	(XX)	XX	(XX)	XX	(XX)	XX	(XX)	XX	(XX)	XX	(XX)
3 months	XX	(XX)	XX	(XX)	XX	(XX)	XX	(XX)	XX	(XX)	XX	(XX)
6 months	XX	(XX)	XX	(XX)	XX	(XX)	XX	(XX)	XX	(XX)	XX	(XX)
Included in analysis †	XX	(XX)			XX	(XX)			XX	(XX)		
**Self efficacy score**												
Baseline	XX	(XX)	XX	(XX)	XX	(XX)	XX	(XX)	XX	(XX)	XX	(XX)
60 days	XX	(XX)	XX	(XX)	XX	(XX)	XX	(XX)	XX	(XX)	XX	(XX)
3 months	XX	(XX)	XX	(XX)	XX	(XX)	XX	(XX)	XX	(XX)	XX	(XX)
6 months	XX	(XX)	XX	(XX)	XX	(XX)	XX	(XX)	XX	(XX)	XX	(XX)
Included in analysis †	XX	(XX)			XX	(XX)			XX	(XX)		
**SHOW-Q global score, for sexually active participants**												
Baseline	XX	(XX)	XX	(XX)	XX	(XX)	XX	(XX)	XX	(XX)	XX	(XX)
60 days	XX	(XX)	XX	(XX)	XX	(XX)	XX	(XX)	XX	(XX)	XX	(XX)
3 months	XX	(XX)	XX	(XX)	XX	(XX)	XX	(XX)	XX	(XX)	XX	(XX)

For peer review only - http://bmjopen.bmj.com/site/about/guidelines.xhtml

Barts and The London School of Medicine and Dentistry

PCTU

6 months	XX	(XX)	XX	(XX)	XX	(XX)	XX	(XX)	XX	(XX)	XX	(XX)
Included in analysis †	XX	(XX)			XX	(XX)			XX	(XX)		
**SHOW-Q pelvic problem interference score, for all participants**												
Baseline	XX	(XX)	XX	(XX)	XX	(XX)	XX	(XX)	XX	(XX)	XX	(XX)
60 days	XX	(XX)	XX	(XX)	XX	(XX)	XX	(XX)	XX	(XX)	XX	(XX)
3 months	XX	(XX)	XX	(XX)	XX	(XX)	XX	(XX)	XX	(XX)	XX	(XX)
6 months	XX	(XX)	XX	(XX)	XX	(XX)	XX	(XX)	XX	(XX)	XX	(XX)
Included in analysis †	XX	(XX)			XX	(XX)			XX	(XX)		
**Subjective outcome score**												
Baseline	XX	(XX)	XX	(XX)	XX	(XX)	XX	(XX)	XX	(XX)	XX	(XX)
60 days	XX	(XX)	XX	(XX)	XX	(XX)	XX	(XX)	XX	(XX)	XX	(XX)
3 months	XX	(XX)	XX	(XX)	XX	(XX)	XX	(XX)	XX	(XX)	XX	(XX)
6 months	XX	(XX)	XX	(XX)	XX	(XX)	XX	(XX)	XX	(XX)	XX	(XX)
Included in analysis †	XX	(XX)			XX	(XX)			XX	(XX)		
**SF-36: Physical functioning**												
Baseline	XX	(XX)	XX	(XX)	XX	(XX)	XX	(XX)	XX	(XX)	XX	(XX)
60 days	XX	(XX)	XX	(XX)	XX	(XX)	XX	(XX)	XX	(XX)	XX	(XX)
3 months	XX	(XX)	XX	(XX)	XX	(XX)	XX	(XX)	XX	(XX)	XX	(XX)
6 months	XX	(XX)	XX	(XX)	XX	(XX)	XX	(XX)	XX	(XX)	XX	(XX)
Included in analysis †	XX	(XX)			XX	(XX)			XX	(XX)		
**SF-36: Pain**												
Baseline	XX	(XX)	XX	(XX)	XX	(XX)	XX	(XX)	XX	(XX)	XX	(XX)

PCTU only

For peer review only - http://bmjopen.bmj.com/site/about/guidelines.xhtml

60 days	XX (XX)	XX (XX)	XX (XX)	XX (XX)	XX (XX)	XX (XX)
3 months	XX (XX)	XX (XX)	XX (XX)	XX (XX)	XX (XX)	XX (XX)
6 months	XX (XX)	XX (XX)	XX (XX)	XX (XX)	XX (XX)	XX (XX)
Included in analysis †	XX (XX)		XX (XX)		XX (XX)	
**SF-36: General Health**						
Baseline	XX (XX)	XX (XX)	XX (XX)	XX (XX)	XX (XX)	XX (XX)
60 days	XX (XX)	XX (XX)	XX (XX)	XX (XX)	XX (XX)	XX (XX)
3 months	XX (XX)	XX (XX)	XX (XX)	XX (XX)	XX (XX)	XX (XX)
6 months	XX (XX)	XX (XX)	XX (XX)	XX (XX)	XX (XX)	XX (XX)
Included in analysis †	XX (XX)		XX (XX)		XX (XX)	
**SF-36: Social Functioning**						
Baseline	XX (XX)	XX (XX)	XX (XX)	XX (XX)	XX (XX)	XX (XX)
60 days	XX (XX)	XX (XX)	XX (XX)	XX (XX)	XX (XX)	XX (XX)
3 months	XX (XX)	XX (XX)	XX (XX)	XX (XX)	XX (XX)	XX (XX)
6 months	XX (XX)	XX (XX)	XX (XX)	XX (XX)	XX (XX)	XX (XX)
Included in analysis †	XX (XX)		XX (XX)		XX (XX)	

(† Included in analysis if outcome is available for at least one follow-up time point.)

For peer review only - http://bmjopen.bmj.com/site/about/guidelines.xhtml

	Intervention vs. Active control Adjusted mean difference (95% CI)	Invention vs. Usual care Adjusted mean difference (95% CI)	Active control vs. Usual care Adjusted mean difference (95% CI)
**Pain acceptance score**			
60 days	XX (XX to XX)	XX (XX to XX)	XX (XX to XX)
3 months	XX (XX to XX)	XX (XX to XX)	XX (XX to XX)
6 months	XX (XX to XX)	XX (XX to XX)	XX (XX to XX)
**Depression score**			
60 days	XX (XX to XX)	XX (XX to XX)	XX (XX to XX)
3 months	XX (XX to XX)	XX (XX to XX)	XX (XX to XX)
6 months	XX (XX to XX)	XX (XX to XX)	XX (XX to XX)
**Anxiety score**			
60 days	XX (XX to XX)	XX (XX to XX)	XX (XX to XX)
3 months	XX (XX to XX)	XX (XX to XX)	XX (XX to XX)
6 months	XX (XX to XX)	XX (XX to XX)	XX (XX to XX)
**Mindfulness score**			
60 days	XX (XX to XX)	XX (XX to XX)	XX (XX to XX)
3 months	XX (XX to XX)	XX (XX to XX)	XX (XX to XX)
6 months	XX (XX to XX)	XX (XX to XX)	XX (XX to XX)
**Pain related disability score**			
60 days	XX (XX to XX)	XX (XX to XX)	XX (XX to XX)
3 months	XX (XX to XX)	XX (XX to XX)	XX (XX to XX)
6 months	XX (XX to XX)	XX (XX to XX)	XX (XX to XX)
**Self efficacy score**			
60 days	XX (XX to XX)	XX (XX to XX)	XX (XX to XX)
3 months	XX (XX to XX)	XX (XX to XX)	XX (XX to XX)
6 months	XX (XX to XX)	XX (XX to XX)	XX (XX to XX)
**SHOW-Q global score, for sexually active participants**			
60 days	XX (XX to XX)	XX (XX to XX)	XX (XX to XX)
3 months	XX (XX to XX)	XX (XX to XX)	XX (XX to XX)
6 months	XX (XX to XX)	XX (XX to XX)	XX (XX to XX)
**SHOW-Q pelvic problem interference score, for all participants**			
60 days	XX (XX to XX)	XX (XX to XX)	XX (XX to XX)
3 months	XX (XX to XX)	XX (XX to XX)	XX (XX to XX)

For peer review only - http://bmjopen.bmj.com/site/about/guidelines.xhtml

6 months	XX (XX to XX)	XX (XX to XX)	XX (XX to XX)
**Subjective outcome score**			
60 days	XX (XX to XX)	XX (XX to XX)	XX (XX to XX)
3 months	XX (XX to XX)	XX (XX to XX)	XX (XX to XX)
6 months	XX (XX to XX)	XX (XX to XX)	XX (XX to XX)
**SF-36: Physical Functioning**			
60 days	XX (XX to XX)	XX (XX to XX)	XX (XX to XX)
3 months	XX (XX to XX)	XX (XX to XX)	XX (XX to XX)
6 months	XX (XX to XX)	XX (XX to XX)	XX (XX to XX)
**SF-36: Pain**			
60 days	XX (XX to XX)	XX (XX to XX)	XX (XX to XX)
3 months	XX (XX to XX)	XX (XX to XX)	XX (XX to XX)
6 months	XX (XX to XX)	XX (XX to XX)	XX (XX to XX)
**SF-36: General Health**			
60 days	XX (XX to XX)	XX (XX to XX)	XX (XX to XX)
3 months	XX (XX to XX)	XX (XX to XX)	XX (XX to XX)
6 months	XX (XX to XX)	XX (XX to XX)	XX (XX to XX)
**SF-36: Social Functioning**			
60 days	XX (XX to XX)	XX (XX to XX)	XX (XX to XX)
3 months	XX (XX to XX)	XX (XX to XX)	XX (XX to XX)
6 months	XX (XX to XX)	XX (XX to XX)	XX (XX to XX)

12.1.10. Unintentional unblinding of randomised treatment

Figures are number (%) unless stated otherwise.

	Intervention (n=…)	Active control (n=…)
Researchers: Which app treatment do you believe the participant was randomised to?		
Intervention app	XX (XX)	XX (XX)
Control app	XX (XX)	XX (XX)
Don't know	XX (XX)	XX (XX)
Participants: Do you think you received the new treatment or comparison treatment?		
New treatment	XX (XX)	XX (XX)
Comparison treatment	XX (XX)	XX (XX)
Don't know	XX (XX)	XX (XX)

For peer review only - http://bmjopen.bmj.com/site/about/guidelines.xhtml

Barts and The London School of Medicine and Dentistry

PCTU

12.1.11. Partially missing clinical outcomes

	Not completed *		Partially Completed **		Fully completed ***	
	n	(%)	n	(%)	n	(%)
**Pain acceptance score**						
Baseline	XX	(XX)	XX	(XX)	XX	(XX)
60 days	XX	(XX)	XX	(XX)	XX	(XX)
3 months	XX	(XX)	XX	(XX)	XX	(XX)
6 months	XX	(XX)	XX	(XX)	XX	(XX)
**Depression score**						
Baseline	XX	(XX)	XX	(XX)	XX	(XX)
60 days	XX	(XX)	XX	(XX)	XX	(XX)
3 months	XX	(XX)	XX	(XX)	XX	(XX)
6 months	XX	(XX)	XX	(XX)	XX	(XX)
**Anxiety score**						
Baseline	XX	(XX)	XX	(XX)	XX	(XX)
60 days	XX	(XX)	XX	(XX)	XX	(XX)
3 months	XX	(XX)	XX	(XX)	XX	(XX)
6 months	XX	(XX)	XX	(XX)	XX	(XX)
**Mindfulness score**						
Baseline	XX	(XX)	XX	(XX)	XX	(XX)
60 days	XX	(XX)	XX	(XX)	XX	(XX)
3 months	XX	(XX)	XX	(XX)	XX	(XX)
6 months	XX	(XX)	XX	(XX)	XX	(XX)
**Pain related disability score**						
Baseline	XX	(XX)	XX	(XX)	XX	(XX)
60 days	XX	(XX)	XX	(XX)	XX	(XX)
3 months	XX	(XX)	XX	(XX)	XX	(XX)
6 months	XX	(XX)	XX	(XX)	XX	(XX)
**Self efficacy score**						
Baseline	XX	(XX)	XX	(XX)	XX	(XX)
60 days	XX	(XX)	XX	(XX)	XX	(XX)
3 months	XX	(XX)	XX	(XX)	XX	(XX)
6 months	XX	(XX)	XX	(XX)	XX	(XX)

For peer review only - http://bmjopen.bmj.com/site/about/guidelines.xhtml

**SHOW-Q global score, for sexually active participants**			
Baseline	XX (XX)	XX (XX)	XX (XX)
60 days	XX (XX)	XX (XX)	XX (XX)
3 months	XX (XX)	XX (XX)	XX (XX)
6 months	XX (XX)	XX (XX)	XX (XX)
**SHOW-Q pelvic problem interference score, for all participants**			
Baseline	XX (XX)	XX (XX)	XX (XX)
60 days	XX (XX)	XX (XX)	XX (XX)
3 months	XX (XX)	XX (XX)	XX (XX)
6 months	XX (XX)	XX (XX)	XX (XX)
**Subjective outcome score**			
Baseline	XX (XX)	XX (XX)	XX (XX)
60 days	XX (XX)	XX (XX)	XX (XX)
3 months	XX (XX)	XX (XX)	XX (XX)
6 months	XX (XX)	XX (XX)	XX (XX)
**SF-36: Physical Functioning**			
Baseline	XX (XX)	XX (XX)	XX (XX)
60 days	XX (XX)	XX (XX)	XX (XX)
3 months	XX (XX)	XX (XX)	XX (XX)
6 months	XX (XX)	XX (XX)	XX (XX)
**SF-36: Pain**			
Baseline	XX (XX)	XX (XX)	XX (XX)
60 days	XX (XX)	XX (XX)	XX (XX)
3 months	XX (XX)	XX (XX)	XX (XX)
6 months	XX (XX)	XX (XX)	XX (XX)
**SF-36: General Health**			
Baseline	XX (XX)	XX (XX)	XX (XX)
60 days	XX (XX)	XX (XX)	XX (XX)
3 months	XX (XX)	XX (XX)	XX (XX)
6 months	XX (XX)	XX (XX)	XX (XX)
**SF-36: Social Functioning**			
Baseline	XX (XX)	XX (XX)	XX (XX)
60 days	XX (XX)	XX (XX)	XX (XX)
3 months	XX (XX)	XX (XX)	XX (XX)
6 months	XX (XX)	XX (XX)	XX (XX)

For peer review only - http://bmjopen.bmj.com/site/about/guidelines.xhtml

Barts and The London School of Medicine and Dentistry

PCTU

* Questionnaire not answered or all variables used in the derivation of the outcome are missing.
** One or more, but not all, variables used in the derivation of the outcome are missing.
*** No variables used in the derivation of the outcome are missing.

For peer review only - http://bmjopen.bmj.com/site/about/guidelines.xhtml

	Questionnaire never returned	Questionnaire answered by telephone			Questionnaire returned		
		Not completed †	Partially completed ††	Fully completed †††	Not completed †	Partially completed ††	Fully completed †††
	n (%)	n (%)	n (%)	n (%)	n (%)	n (%)	n (%)
**Pain acceptance score**							
Baseline	XX (XX)	n/a	n/a	n/a	XX (XX)	XX (XX)	XX (XX)
60 days	XX (XX)	XX (XX)	XX (XX)	XX (XX)	XX (XX)	XX (XX)	XX (XX)
3 months	XX (XX)	XX (XX)	XX (XX)	XX (XX)	XX (XX)	XX (XX)	XX (XX)
6 months	XX (XX)	XX (XX)	XX (XX)	XX (XX)	XX (XX)	XX (XX)	XX (XX)
**Depression score**							
Baseline	XX (XX)	n/a	n/a	n/a	XX (XX)	XX (XX)	XX (XX)
60 days	XX (XX)	XX (XX)	XX (XX)	XX (XX)	XX (XX)	XX (XX)	XX (XX)
3 months	XX (XX)	XX (XX)	XX (XX)	XX (XX)	XX (XX)	XX (XX)	XX (XX)
6 months	XX (XX)	XX (XX)	XX (XX)	XX (XX)	XX (XX)	XX (XX)	XX (XX)
**Anxiety score**							
Baseline	XX (XX)	n/a	n/a	n/a	XX (XX)	XX (XX)	XX (XX)
60 days	XX (XX)	XX (XX)	XX (XX)	XX (XX)	XX (XX)	XX (XX)	XX (XX)
3 months	XX (XX)	XX (XX)	XX (XX)	XX (XX)	XX (XX)	XX (XX)	XX (XX)
6 months	XX (XX)	XX (XX)	XX (XX)	XX (XX)	XX (XX)	XX (XX)	XX (XX)
**Mindfulness score**							
Baseline	XX (XX)	n/a	n/a	n/a	XX (XX)	XX (XX)	XX (XX)

PCTU only

60 days	XX (XX)	XX (XX)	XX (XX)	XX (XX)	XX (XX)	XX (XX)	XX (XX)
3 months	XX (XX)	XX (XX)	XX (XX)	XX (XX)	XX (XX)	XX (XX)	XX (XX)
6 months	XX (XX)	XX (XX)	XX (XX)	XX (XX)	XX (XX)	XX (XX)	XX (XX)
**Pain related disability score**							
Baseline	XX (XX)	n/a	n/a	n/a	XX (XX)	XX (XX)	XX (XX)
60 days	XX (XX)	XX (XX)	XX (XX)	XX (XX)	XX (XX)	XX (XX)	XX (XX)
3 months	XX (XX)	XX (XX)	XX (XX)	XX (XX)	XX (XX)	XX (XX)	XX (XX)
6 months	XX (XX)	XX (XX)	XX (XX)	XX (XX)	XX (XX)	XX (XX)	XX (XX)
**Self efficacy score**							
Baseline	XX (XX)	n/a	n/a	n/a	XX (XX)	XX (XX)	XX (XX)
60 days	XX (XX)	n/a	n/a	n/a	XX (XX)	XX (XX)	XX (XX)
3 months	XX (XX)	n/a	n/a	n/a	XX (XX)	XX (XX)	XX (XX)
6 months	XX (XX)	n/a	n/a	n/a	XX (XX)	XX (XX)	XX (XX)
**SHOW-Q global score, for sexually active participants**							
Baseline	XX (XX)	n/a	n/a	n/a	XX (XX)	XX (XX)	XX (XX)
60 days	XX (XX)	n/a	n/a	n/a	XX (XX)	XX (XX)	XX (XX)
3 months	XX (XX)	n/a	n/a	n/a	XX (XX)	XX (XX)	XX (XX)
6 months	XX (XX)	n/a	n/a	n/a	XX (XX)	XX (XX)	XX (XX)
**SHOW-Q pelvic problem interference score, for all participants**							
Baseline	XX (XX)	n/a	n/a	n/a	XX (XX)	XX (XX)	XX (XX)
60 days	XX (XX)	n/a	n/a	n/a	XX (XX)	XX (XX)	XX (XX)
3 months	XX (XX)	n/a	n/a	n/a	XX (XX)	XX (XX)	XX (XX)

For peer review only - http://bmjopen.bmj.com/site/about/guidelines.xhtml

Barts and The London
School of Medicine and Dentistry

PCTU

6 months	XX (XX)	n/a	n/a	n/a	XX (XX)	XX (XX)	XX (XX)
**Subjective outcome score**							
Baseline	XX (XX)	n/a	n/a	n/a	XX (XX)	XX (XX)	XX (XX)
60 days	XX (XX)	XX (XX)	XX (XX)	XX (XX)	XX (XX)	XX (XX)	XX (XX)
3 months	XX (XX)	XX (XX)	XX (XX)	XX (XX)	XX (XX)	XX (XX)	XX (XX)
6 months	XX (XX)	XX (XX)	XX (XX)	XX (XX)	XX (XX)	XX (XX)	XX (XX)
**SF-36: Physical Functioning**							
Baseline	XX (XX)	n/a	n/a	n/a	XX (XX)	XX (XX)	XX (XX)
60 days	XX (XX)	n/a	n/a	n/a	XX (XX)	XX (XX)	XX (XX)
3 months	XX (XX)	n/a	n/a	n/a	XX (XX)	XX (XX)	XX (XX)
6 months	XX (XX)	n/a	n/a	n/a	XX (XX)	XX (XX)	XX (XX)
**SF-36: Pain**							
Baseline	XX (XX)	n/a	n/a	n/a	XX (XX)	XX (XX)	XX (XX)
60 days	XX (XX)	n/a	n/a	n/a	XX (XX)	XX (XX)	XX (XX)
3 months	XX (XX)	n/a	n/a	n/a	XX (XX)	XX (XX)	XX (XX)
6 months	XX (XX)	n/a	n/a	n/a	XX (XX)	XX (XX)	XX (XX)
**SF-36: General Health**							
Baseline	XX (XX)	n/a	n/a	n/a	XX (XX)	XX (XX)	XX (XX)
60 days	XX (XX)	n/a	n/a	n/a	XX (XX)	XX (XX)	XX (XX)
3 months	XX (XX)	n/a	n/a	n/a	XX (XX)	XX (XX)	XX (XX)
6 months	XX (XX)	n/a	n/a	n/a	XX (XX)	XX (XX)	XX (XX)
**SF-36: Social Functioning**							

PCTU only

For peer review only - http://bmjopen.bmj.com/site/about/guidelines.xhtml

Baseline	XX (XX)	n/a	n/a	n/a	XX (XX)	XX (XX)	XX (XX)
60 days	XX (XX)	n/a	n/a	n/a	XX (XX)	XX (XX)	XX (XX)
3 months	XX (XX)	n/a	n/a	n/a	XX (XX)	XX (XX)	XX (XX)
6 months	XX (XX)	n/a	n/a	n/a	XX (XX)	XX (XX)	XX (XX)

† Questionnaire answered, but all variables used in the derivation of the outcome are missing.
†† One or more, but not all, variables used in the derivation of the outcome are missing.
††† No variables used in the derivation of the outcome are missing.

For peer review only - http://bmjopen.bmj.com/site/about/guidelines.xhtml

13. APPENDIX E: DATA / FILE MANAGEMENT

13.1.1. Sources of data

Copies of CRFs are included in the Statistics Master File. Data is entered from these into a PCTU database. Extracts from the database are supplied by the data manager onto a secure environment.

App usage data will be received from Headspace.

13.1.2. Programming plan

The trial folder on secure environment will contain a folder for each analysis.

An analysis folder should contain the following folders (and their contents):

- analysis data (saved Stata data files for analysis)
- do files (Stata do files for data preparation and analysis)
- log files (Stata log files)
- output (any files output e.g. produced tables and graphs)
- raw data (data as extracted from database)
- temp (any temporary files needed during data preparation or analysis)

Folders containing do files should include a text directory explaining the role of each do file.

13.1.3. Data dictionary

Field names specified in the database "Requirements Specification Document" will be the variable names in the data files. Where a variable is collect on more than one occasion, suffixes will be added to variables names (e.g. "_BASELINE", "_60DAYS", "_3MONTHS", "_6MONTHS").

Details of derived variables are given in Section 5, APPENDIX A: DERIVED AND COMPUTED VARIABLES, and APPENDIX B: STATA CODE FOR GENERATING ADHERENCE OUTCOMES.

A complete data dictionary will be produced for the final analysis data set.

For peer review only - http://bmjopen.bmj.com/site/about/guidelines.xhtml

Barts and The London School of Medicine and Dentistry

PCTU

Appendix 3: Supplementary tables

TABLE OF CONTENTS

Table 1. Prior and concurrent treatment ... 3
Table 2. Baseline values of clinical outcomes ... 4
Table 3. Baseline demographics of woman by 6 month questionnaire completion ... 5
1. FEASIBILITY OUTCOMES ... 7
1.1. FOLLOW-UP ... 7
Table 4. Losses to follow up ... 7
Figure 1. Proportion of participants answering follow up questionnaire ... 8
Table 5. Follow-up questionnaire returned or answered by phone within target follow up period ... 9
1.2. STANDARD DEVIATION OF CPAQ ... 10
Table 6. Estimated standard deviation of CPAQ ... 10
1.4. BLINDING ... 11
Table 7. Unintentional unbinding of randomised treatment ... 11
2. APP SATISFACTION QUESTIONNAIRES ... 12
Table 8. System usability scale ... 12
Table 9. App usability Questionnaire ... 13
3. CLINICAL OUTCOMES ... 14
3.1. RANGES OF CLINICAL OUTCOMES ... 14
3.2. COMPLETENESS OF CLINICAL DATA ... 15
Table 10. Partially missing clinical outcomes ... 15
Table 11. Partially missing clinical outcomes by method of questionnaire delivery ... 17
3.3. RESULTS OF ANALYSIS OF CLINICAL OUTCOMES ... 20
Table 12. Descriptive statistics for clinical outcomes ... 20
Table 13. Estimated treatment effects for clinical outcomes ... 23

For peer review only - http://bmjopen.bmj.com/site/about/guidelines.xhtml

Figure 2. Estimated treatment effects and 95% confidence intervals for CPAQ 25

For peer review only - http://bmjopen.bmj.com/site/about/guidelines.xhtml

Barts and The London School of Medicine and Dentistry

PCTU

Table 1. Prior and concurrent treatment

Figures are number (percentage).

	Summary measure			Missing data		
	Intervention (N=31)	Active control (N=30)	Usual care (N=29)	Intervention - no. (%)	Active control - no. (%)	Usual care - no. (%)
**Treatment used in the last six months**						
Acupuncture	2 (10.5)	5 (25.0)	1 (6.3)	12 (38.7)	10 (33.3)	13 (44.8)
Massage	11 (50.0)	8 (40.0)	7 (41.2)	9 (29.0)	10 (33.3)	12 (41.4)
Gabapentin	5 (26.3)	1 (5.9)	1 (6.3)	12 (38.7)	13 (43.3)	13 (44.8)
Nutrition/diet	14 (63.6)	14 (63.6)	18 (78.3)	9 (29.0)	8 (26.7)	6 (20.7)
Amitriptyline	5 (27.8)	4 (20.0)	4 (22.2)	13 (41.9)	10 (33.3)	11 (37.9)
Codeine or Morphine type painkillers	13 (56.5)	13 (59.1)	19 (76.0)	8 (25.8)	8 (26.7)	4 (13.8)
Biofeedback	0 (0.0)	0 (0.0)	0 (0.0)	13 (41.9)	12 (40.0)	13 (44.8)
Nerve blocks	0 (0.0)	2 (11.1)	0 (0.0)	14 (45.2)	12 (40.0)	12 (41.4)
Botox injection	0 (0.0)	0 (0.0)	0 (0.0)	14 (45.2)	13 (43.3)	13 (44.8)
Over the counter medication	17 (73.9)	9 (47.4)	17 (77.3)	8 (25.8)	11 (36.7)	7 (24.1)
Contraceptive pills/patch/ring	15 (68.2)	7 (36.8)	11 (52.4)	9 (29.0)	11 (36.7)	8 (27.6)
Physiotherapy	5 (26.3)	4 (20.0)	1 (6.7)	12 (38.7)	10 (33.3)	14 (48.3)
Exercise, yoga or Pilates	13 (59.1)	12 (60.0)	15 (78.9)	9 (29.0)	10 (33.3)	10 (34.5)
Psychological (talking) therapy	3 (16.7)	2 (11.1)	2 (13.3)	13 (41.9)	12 (40.0)	14 (48.3)
Injections to suppress ovaries (e.g. Prostap, Zoladex)	6 (33.3)	5 (25.0)	8 (38.1)	13 (41.9)	10 (33.3)	8 (27.6)
Transcutaneous Electrical Nerve Stimulation (TENS)	0 (0.0)	2 (11.1)	3 (17.6)	13 (41.9)	12 (40.0)	12 (41.4)
Herbal Medicine	4 (21.1)	5 (26.3)	8 (44.4)	12 (38.7)	11 (36.7)	11 (37.9)
Surgery	3 (16.7)	4 (23.5)	6 (31.6)	13 (41.9)	13 (43.3)	10 (34.5)
Meditation or relaxation exercises	11 (47.8)	7 (38.9)	10 (52.6)	8 (25.8)	12 (40.0)	10 (34.5)
Other	3 (37.5)	3 (33.3)	4 (44.4)	23 (74.2)	21 (70.0)	20 (69.0)
Currently using pain treatment				4 (12.9)	3 (10.0)	2 (6.9)
Yes	21 (77.8)	18 (66.7)	20 (74.1)			
No	6 (22.2)	9 (33.3)	7 (25.9)			

For peer review only - http://bmjopen.bmj.com/site/about/guidelines.xhtml

Barts and The London
School of Medicine and Dentistry

PCTU

Table 2. Baseline values of clinical outcomes

Figures are mean (SD)

	Summary measure			Missing data		
	Intervention (N=31)	Active control (N=30)	Usual care (N=29)	Intervention - no. (%)	Active control - no. (%)	Usual care - no. (%)
CPAQ pain acceptance score	21.9 (9.5)	22.7 (8.4)	23.8 (8.5)	2 (6.5)	3 (10.0)	1 (3.4)
HADS depression score	8.7 (5.1)	8.6 (5.0)	7.4 (3.6)	1 (3.2)	3 (10.0)	2 (6.9)
HADS anxiety score	12.6 (5.3)	12.0 (5.3)	10.9 (3.9)	1 (3.2)	4 (13.3)	1 (3.4)
CAMS-R mindfulness score	28.6 (6.1)	28.8 (7.1)	30.3 (5.4)	3 (9.7)	5 (16.7)	3 (10.3)
CPG disability score	60.6 (24.4)	64.6 (19.6)	59.2 (24.4)	1 (3.2)	3 (10.0)	1 (3.4)
PSEQ Self efficacy score	29.1 (14.7)	27.9 (14.6)	35.5 (10.6)	1 (3.2)	3 (10.0)	2 (6.9)
Sexual health outcomes:						
SHOW-Q global score*	45.4 (20.3)	50.9 (20.9)	58.1 (22.2)	5 (16.1)	7 (23.3)	3 (10.3)
SHOW-Q pelvic problem interference score	47.1 (29.0)	49.0 (32.7)	56.4 (25.9)	8 (25.8)	6 (20.0)	3 (10.3)
MYMOP subjective outcome score	4.1 (1.2)	3.9 (1.3)	3.9 (1.1)	1 (3.2)	3 (10.0)	2 (6.9)
SF-36 Scales:						
SF36 - Physical functioning	56.3 (30.2)	55.8 (32.2)	66.5 (30.4)	3 (9.7)	4 (13.3)	2 (6.9)
SF36 - Pain	35.1 (17.5)	34.7 (20.6)	37.6 (20.6)	1 (3.2)	3 (10.0)	1 (3.4)
SF36 - General Health	39.1 (20.3)	42.0 (19.8)	37.9 (21.4)	2 (6.5)	3 (10.0)	1 (3.4)
SF36 - Social functioning	37.5 (19.1)	38.0 (28.3)	50.4 (25.3)	1 (3.2)	3 (10.0)	1 (3.4)

*Show-Q global is only applicable for sexually active participants. At baseline there are 17 sexually active women in the intervention group, 22 in the active control group and 19 in the usual care group.

For peer review only - http://bmjopen.bmj.com/site/about/guidelines.xhtml

Barts and The London School of Medicine and Dentistry
PCTU

Table 3. Baseline demographics of woman by 6 month questionnaire completion

Figures are mean (SD) unless stated otherwise.

	6 month follow-up questionnaire returned (N=33)	6 month follow-up questionnaire answered by phone (N=24)	6 month follow-up questionnaire never returned (N=33)
**Demographics**			
Age (Years)	35.8 (8.0)	36.6 (9.2)	33.1 (7.5)
Body mass index (kg/m^2)	27.4 (7.1)	27.7 (6.5)	25.9 (4.5)
Living arrangements - no. (%)			
Alone	2 (6.3)	1 (4.2)	3 (10.7)
With others	30 (93.8)	23 (95.8)	25 (89.3)
Employment status - no. (%)			
Employed	26 (78.8)	13 (54.2)	17 (60.7)
Unemployed and looking for work	1 (3.0)	1 (4.2)	1 (3.6)
At school or in full time education	1 (3.0)	2 (8.3)	4 (14.3)
Unable to work due to long term sickness	3 (9.1)	4 (16.7)	3 (10.7)
Looking after your home/family	2 (6.1)	3 (12.5)	3 (10.7)
Retired from paid work	0 (0.0)	1 (4.2)	0 (0.0)
Age left full time education - no. (%)			
Age 12 or less	0 (0.0)	3 (12.5)	0 (0.0)
Age 13 to 16	2 (6.1)	6 (25.0)	8 (29.6)
Age 17 to 19	7 (21.2)	2 (8.3)	5 (18.5)
Age 20 or over	23 (69.7)	9 (37.5)	10 (37.0)
Still in education	1 (3.0)	4 (16.7)	4 (14.8)
Ethnic group - no. (%)			
White	18 (58.1)	9 (40.9)	8 (30.8)
Black	7 (22.6)	4 (18.2)	2 (7.7)
Central Asian	0 (0.0)	0 (0.0)	2 (7.7)
Middle Eastern	0 (0.0)	0 (0.0)	1 (3.8)
Southern Asian	5 (16.1)	6 (27.3)	7 (26.9)
Mixed	1 (3.2)	0 (0.0)	1 (3.8)
Other ethnic group	0 (0.0)	2 (9.1)	4 (15.4)
Do not wish to say	0 (0.0)	1 (4.5)	1 (3.8)

For peer review only - http://bmjopen.bmj.com/site/about/guidelines.xhtml

	6 month follow-up questionnaire returned (N=33)	6 month follow-up questionnaire answered by phone (N=24)	6 month follow-up questionnaire never returned (N=33)
Smoker - no. (%)			
Yes	6 (18.8)	4 (18.2)	7 (25.9)
No	26 (81.3)	18 (81.8)	20 (74.1)
If yes, number of cigarettes per week	36.0 (24.1)	15.3 (12.5)	44.0 (30.8)
Drink alcohol - no. (%)			
Yes	18 (56.3)	6 (27.3)	10 (37.0)
No	14 (43.8)	16 (72.7)	17 (63.0)
If yes, number of units per week	8.9 (7.2)	5.8 (5.3)	5.2 (2.9)
**Baseline medical history**			
Duration of pain - no. (%)			
0 to 6 months	0 (0.0)	1 (4.2)	1 (3.6)
7 to 12 months	3 (9.1)	0 (0.0)	5 (17.9)
1 to 2 years	6 (18.2)	3 (12.5)	4 (14.3)
3 to 5 years	10 (30.3)	10 (41.7)	6 (21.4)
6 to 10 years	5 (15.2)	3 (12.5)	3 (10.7)
More than 10 years	9 (27.3)	7 (29.2)	9 (32.1)
Pain over the past week	6.0 (2.5)	6.0 (2.6)	7.5 (2.2)
**Baseline values of clinical outcomes**			
CPAQ pain acceptance score	25.3 (8.4)	20.8 (8.8)	21.4 (8.7)
HADS depression score	6.6 (3.6)	8.5 (4.9)	10.0 (4.9)
HADS anxiety score	10.3 (4.7)	12.2 (5.1)	13.5 (4.5)
CPG disability score	54.5 (18.8)	65.4 (20.7)	66.0 (27.4)

For peer review only - http://bmjopen.bmj.com/site/about/guidelines.xhtml

Barts and The London School of Medicine and Dentistry

PCTU

1. Feasibility Outcomes

1.1. Follow-up

Table 4. Losses to follow up

	Intervention (N=31)	Active control (N=30)	Usual care (N=29)
**Follow-up questionnaire returned - no (%)**			
60 days	15 (48.4)	9 (30.0)	18 (62.1)
3 months	17 (54.8)	12 (40.0)	17 (58.6)
6 months	11 (35.5)	10 (33.3)	12 (41.4)
**Follow-up questionnaire answered by phone - no (%)**			
60 days	1 (3.2)	8 (26.7)	1 (3.4)
3 months	3 (9.7)	7 (23.3)	3 (10.3)
6 months	10 (32.3)	6 (20.0)	8 (27.6)
**Follow-up questionnaire never returned - no (%)**			
60 days	15 (48.4)	13 (43.3)	10 (34.5)
3 months	11 (35.5)	11 (36.7)	9 (31.0)
6 months	10 (32.3)	14 (46.7)	9 (31.0)

For peer review only - http://bmjopen.bmj.com/site/about/guidelines.xhtml

Barts and The London
School of Medicine and Dentistry

PCTU

Figure 1. *Proportion of participants answering follow up questionnaire*

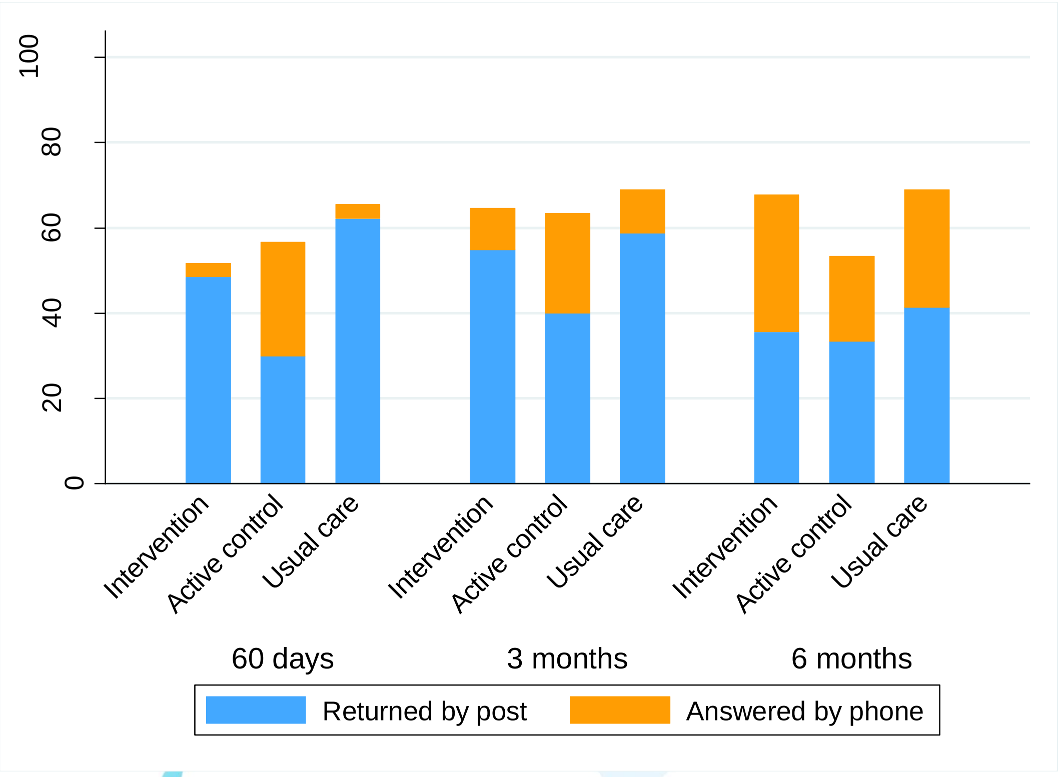

For peer review only - http://bmjopen.bmj.com/site/about/guidelines.xhtml

Barts and The London
School of Medicine and Dentistry

PCTU

Table 5. Follow-up questionnaire returned or answered by phone within target follow up period

Figures are no returning data on time/no. returning data questionnaire answering by phone (%)*.

	Intervention (N=31)	Active control (N=30)	Usual care (N=29)
60 days (47 and 74 days)	7/16 (43.8)	6/17 (35.3)	11/19 (57.9)
3 months (76 and 104 days)	7/20 (35.0)	6/19 (31.6)	11/20 (55.0)
6 months (159 and 201 days)	7/21 (33.3)	6/16 (37.5)	11/20 (55.0)

*Denominator for percentage is number returning data questionnaire answering by phone

For peer review only - http://bmjopen.bmj.com/site/about/guidelines.xhtml

Barts and The London School of Medicine and Dentistry

PCTU

1.2. Standard deviation of CPAQ

Table 6. Estimated standard deviation of CPAQ

	Number with complete outcome	Estimated standard deviation
60 days	50	9.6
3 months	55	8.1
6 months	56	9.6

For peer review only - http://bmjopen.bmj.com/site/about/guidelines.xhtml

Barts and The London School of Medicine and Dentistry PCTU

1.4. Blinding

Table 7. Unintentional unbinding of randomised treatment

Figures are number (%)

	Summary measure		Missing data	
	**Intervention (N=31)**	**Active control (N=30)**	**Intervention - no. (%)**	**Active control - no. (%)**
Researchers: Which app treatment do you believe the participant was randomised to?			2 (6.5)	3 (10.0)
Intervention app	0 (0.0)	1 (3.7)		
Control app	0 (0.0)	0 (0.0)		
Don't know	29 (100.0)	26 (96.3)		
Participants: Do you think you received the new treatment or comparison treatment?			15 (48.4)	19 (63.3)
New treatment	1 (6.3)	1 (9.1)		
Comparison treatment	0 (0.0)	1 (9.1)		
Don't know	15 (93.8)	9 (81.8)		

For peer review only - http://bmjopen.bmj.com/site/about/guidelines.xhtml

2. App satisfaction questionnaires

Table 8. System usability scale

Figures are mean (sd).

	Summary measure		Missing Data	
	Intervention (N=31)	Active control (N=30)	Intervention - no. (%)	Active control - no. (%)
System usability scale	50.7 (6.6)	46.0 (12.0)	16 (51.6)	18 (60.0)

For peer review only - http://bmjopen.bmj.com/site/about/guidelines.xhtml

Barts and The London School of Medicine and Dentistry

PCTU

Table 9. App usability Questionnaire

Figures are number (%).

	Totally disagree	Somewhat disagree	Neither agree nor disagree	Somewhat agree	Totally agree	Not answered
It is easy to use the app whenever I wanted to use it						
Intervention:	0 (0.0)	3 (9.7)	1 (3.2)	3 (9.7)	9 (29.0)	15 (48.4)
Active control:	0 (0.0)	0 (0.0)	0 (0.0)	4 (13.3)	8 (26.7)	18 (60.0)
After being shown, I understood how the app would work						
Intervention:	0 (0.0)	1 (3.2)	1 (3.2)	4 (12.9)	9 (29.0)	16 (51.6)
Active control:	0 (0.0)	2 (6.7)	0 (0.0)	2 (6.7)	8 (26.7)	18 (60.0)
It was fun to work with the app						
Intervention:	0 (0.0)	2 (6.5)	3 (9.7)	8 (25.8)	2 (6.5)	16 (51.6)
Active control:	0 (0.0)	3 (10.0)	3 (10.0)	5 (16.7)	1 (3.3)	18 (60.0)
The app worked well						
Intervention:	0 (0.0)	3 (9.7)	2 (6.5)	5 (16.1)	5 (16.1)	16 (51.6)
Active control:	0 (0.0)	2 (6.7)	2 (6.7)	4 (13.3)	4 (13.3)	18 (60.0)
It was easy to work through the modules						
Intervention:	0 (0.0)	4 (12.9)	0 (0.0)	5 (16.1)	6 (19.4)	16 (51.6)
Active control:	0 (0.0)	2 (6.7)	3 (10.0)	4 (13.3)	3 (10.0)	18 (60.0)
The number of modules was annoying						
Intervention:	1 (3.2)	4 (12.9)	6 (19.4)	5 (16.1)	0 (0.0)	15 (48.4)
Active control:	2 (6.7)	3 (10.0)	3 (10.0)	2 (6.7)	2 (6.7)	18 (60.0)
The modules were well-displayed on my smartphone						
Intervention:	0 (0.0)	2 (6.5)	1 (3.2)	8 (25.8)	5 (16.1)	15 (48.4)
Active control:	0 (0.0)	0 (0.0)	2 (6.7)	2 (6.7)	7 (23.3)	19 (63.3)
Using the app was difficult because of my daily activities						
Intervention:	2 (6.5)	2 (6.5)	3 (9.7)	9 (29.0)	0 (0.0)	15 (48.4)
Active control:	1 (3.3)	1 (3.3)	0 (0.0)	5 (16.7)	5 (16.7)	18 (60.0)
Using the app took too long						
Intervention:	2 (6.5)	4 (12.9)	3 (9.7)	7 (22.6)	0 (0.0)	15 (48.4)
Active control:	1 (3.3)	3 (10.0)	2 (6.7)	4 (13.3)	2 (6.7)	18 (60.0)

For peer review only - http://bmjopen.bmj.com/site/about/guidelines.xhtml

Barts and The London School of Medicine and Dentistry PCTU

3. Clinical Outcomes

3.1. Ranges of clinical outcomes

Pain acceptance score (as measured by the Chronic Pain Acceptance Questionnaire (CPAQ)):

- 0 (worst) – 48 (best)

Depression score (Hospital Anxiety and Depression Scale (HADS)):

- 0 (best) – 21 (worst)

Anxiety score (measured by HADS):

- 0 (best) – 21 (worst)

Mindfulness score (Cognitive and Mindfulness - Revised (CAMS – R) scale):

- 12 (worst) – 48 (best)

Pain related disability score (as measured by the Chronic Pain Grade (CPG) disability subscale):

- 0 (best) – 100 (worst)

Self efficacy score (as measured by the Pain Self-Efficacy Questionnaire (PSEQ)):

- 0 (worst) – 60 (best)

Sexual Health Outcomes scores (as measured by the Sexual Health Outcomes in Women Questionnaire (SHOW-Q))

- SHOW-Q global score, for sexually active participants: 0 (worst) – 100 (best)
- SHOW-Q pelvic interference score, for all participants: 0 (best) – 100 (worst)

Subjective outcome score (as measured by the Measure Yourself Medical Outcome Profile (MYMOP)):

- 0 (best) – 6)worst)

RAND Short form (36) Health Survey (RAND SF-36) scales:

- Physical functioning: 0 (worst) – 100)best)
- Pain: 0 (worst) – 100 (best)
- General health: 0 (worst) – 100 (best)
- Social functioning: 0 (worst) – 100 (best)

For peer review only - http://bmjopen.bmj.com/site/about/guidelines.xhtml

Barts and The London School of Medicine and Dentistry

PCTU

3.2. Completeness of clinical data

Table 10. Partially missing clinical outcomes

Figures are number (%)

	Not completed* no. (%)	Partially completed** no. (%)	Fully completed*** no. (%)
**CPAQ pain acceptance score**			
Baseline	5 (5.6)	1 (1.1)	84 (93.3)
60 days	40 (44.4)	0 (0.0)	50 (55.6)
3 months	32 (35.6)	3 (3.3)	55 (61.1)
6 months	34 (37.8)	0 (0.0)	56 (62.2)
**HADS depression score**			
Baseline	5 (5.6)	1 (1.1)	84 (93.3)
60 days	40 (44.4)	1 (1.1)	49 (54.4)
3 months	32 (35.6)	0 (0.0)	58 (64.4)
6 months	33 (36.7)	0 (0.0)	57 (63.3)
**HADS anxiety score**			
Baseline	5 (5.6)	1 (1.1)	84 (93.3)
60 days	40 (44.4)	0 (0.0)	50 (55.6)
3 months	32 (35.6)	0 (0.0)	58 (64.4)
6 months	33 (36.7)	0 (0.0)	57 (63.3)
**CAMS-R mindfulness score**			
Baseline	5 (5.6)	6 (6.7)	79 (87.8)
60 days	40 (44.4)	0 (0.0)	50 (55.6)
3 months	32 (35.6)	2 (2.2)	56 (62.2)
6 months	33 (36.7)	0 (0.0)	57 (63.3)
**CPG disability score**			
Baseline	5 (5.6)	0 (0.0)	85 (94.4)
60 days	40 (44.4)	0 (0.0)	50 (55.6)
3 months	33 (36.7)	0 (0.0)	57 (63.3)
6 months	34 (37.8)	0 (0.0)	56 (62.2)
**PSEQ Self efficacy score**			
Baseline	5 (5.6)	1 (1.1)	84 (93.3)
60 days	50 (55.6)	1 (1.1)	39 (43.3)
3 months	45 (50.0)	0 (0.0)	45 (50.0)
6 months	57 (63.3)	0 (0.0)	33 (36.7)

* Questionnaire not answered or all variables used in the derivation of the outcome are missing.
** One or more, but not all, variables used in the derivation of the outcome are missing.
*** No variables used in the derivation of the outcome are missing.

For peer review only - http://bmjopen.bmj.com/site/about/guidelines.xhtml

Barts and The London
School of Medicine and Dentistry

PCTU

	Not completed* no. (%)	Partially completed** no. (%)	Fully completed*** no. (%)
**SHOW-Q global score**			
Baseline	5 (5.6)	15 (16.7)	70 (77.8)
60 days	50 (55.6)	6 (6.7)	34 (37.8)
3 months	47 (52.2)	8 (8.9)	35 (38.9)
6 months	58 (64.4)	5 (5.6)	27 (30.0)
**SHOW-Q pelvic problem interference score**			
Baseline	9 (10.0)	8 (8.9)	73 (81.1)
60 days	51 (56.7)	3 (3.3)	36 (40.0)
3 months	49 (54.4)	3 (3.3)	38 (42.2)
6 months	60 (66.7)	1 (1.1)	29 (32.2)
**MYMOP subjective outcome score**			
Baseline	5 (5.6)	1 (1.1)	84 (93.3)
60 days	38 (42.2)	11 (12.2)	41 (45.6)
3 months	33 (36.7)	10 (11.1)	47 (52.2)
6 months	33 (36.7)	6 (6.7)	51 (56.7)
**SF36 - General Health**			
Baseline	5 (5.6)	1 (1.1)	84 (93.3)
60 days	38 (42.2)	11 (12.2)	41 (45.6)
3 months	31 (34.4)	14 (15.6)	45 (50.0)
6 months	33 (36.7)	24 (26.7)	33 (36.7)
**SF36 - Physical functioning**			
Baseline	5 (5.6)	4 (4.4)	81 (90.0)
60 days	48 (53.3)	3 (3.3)	39 (43.3)
3 months	45 (50.0)	2 (2.2)	43 (47.8)
6 months	57 (63.3)	3 (3.3)	30 (33.3)
**SF36 - Pain**			
Baseline	5 (5.6)	0 (0.0)	85 (94.4)
60 days	48 (53.3)	0 (0.0)	42 (46.7)
3 months	45 (50.0)	0 (0.0)	45 (50.0)
6 months	57 (63.3)	0 (0.0)	33 (36.7)
**SF36 - Social functioning**			
Baseline	5 (5.6)	0 (0.0)	85 (94.4)
60 days	48 (53.3)	1 (1.1)	41 (45.6)
3 months	45 (50.0)	1 (1.1)	44 (48.9)
6 months	57 (63.3)	1 (1.1)	32 (35.6)

* Questionnaire not answered or all variables used in the derivation of the outcome are missing.
** One or more, but not all, variables used in the derivation of the outcome are missing.
*** No variables used in the derivation of the outcome are missing.

For peer review only - http://bmjopen.bmj.com/site/about/guidelines.xhtml

Barts and The London School of Medicine and Dentistry

PCTU

Table 11. Partially missing clinical outcomes by method of questionnaire delivery

Figures are number (%)

	Questionnaire never returned no. (%)	Questionnaire answered by telephone: Not completed* no. (%)	Partially completed** no. (%)	Fully completed*** no. (%)	Questionnaire returned: Not completed* no. (%)	Partially completed** no. (%)	Fully completed*** no. (%)
**CPAQ pain acceptance score**							
Baseline	5 (5.6)	n/a	n/a	n/a	0 (0.0)	1 (1.1)	84 (93.3)
60 days	38 (42.2)	1 (1.1)	0 (0.0)	9 (10.0)	1 (1.1)	0 (0.0)	41 (45.6)
3 months	31 (34.4)	0 (0.0)	0 (0.0)	13 (14.4)	1 (1.1)	3 (3.3)	42 (46.7)
6 months	33 (36.7)	1 (1.1)	0 (0.0)	23 (25.6)	0 (0.0)	0 (0.0)	33 (36.7)
**HADS depression score**							
Baseline	5 (5.6)	n/a	n/a	n/a	0 (0.0)	1 (1.1)	84 (93.3)
60 days	38 (42.2)	1 (1.1)	0 (0.0)	9 (10.0)	1 (1.1)	1 (1.1)	40 (44.4)
3 months	31 (34.4)	0 (0.0)	0 (0.0)	13 (14.4)	1 (1.1)	0 (0.0)	45 (50.0)
6 months	33 (36.7)	0 (0.0)	0 (0.0)	24 (26.7)	0 (0.0)	0 (0.0)	33 (36.7)
**HADS anxiety score**							
Baseline	5 (5.6)	n/a	n/a	n/a	0 (0.0)	1 (1.1)	84 (93.3)
60 days	38 (42.2)	1 (1.1)	0 (0.0)	9 (10.0)	1 (1.1)	0 (0.0)	41 (45.6)
3 months	31 (34.4)	0 (0.0)	0 (0.0)	13 (14.4)	1 (1.1)	0 (0.0)	45 (50.0)
6 months	33 (36.7)	0 (0.0)	0 (0.0)	24 (26.7)	0 (0.0)	0 (0.0)	33 (36.7)
**CAMS-R mindfulness score**							
Baseline	5 (5.6)	n/a	n/a	n/a	0 (0.0)	6 (6.7)	79 (87.8)
60 days	38 (42.2)	1 (1.1)	0 (0.0)	9 (10.0)	1 (1.1)	0 (0.0)	41 (45.6)
3 months	31 (34.4)	0 (0.0)	0 (0.0)	13 (14.4)	1 (1.1)	2 (2.2)	43 (47.8)
6 months	33 (36.7)	0 (0.0)	0 (0.0)	24 (26.7)	0 (0.0)	0 (0.0)	33 (36.7)
**CPG disability score**							
Baseline	5 (5.6)	n/a	n/a	n/a	0 (0.0)	0 (0.0)	85 (94.4)
60 days	38 (42.2)	1 (1.1)	0 (0.0)	9 (10.0)	1 (1.1)	0 (0.0)	41 (45.6)
3 months	31 (34.4)	0 (0.0)	0 (0.0)	13 (14.4)	2 (2.2)	0 (0.0)	44 (48.9)
6 months	33 (36.7)	1 (1.1)	0 (0.0)	23 (25.6)	0 (0.0)	0 (0.0)	33 (36.7)

* Questionnaire not answered or all variables used in the derivation of the outcome are missing.
** One or more, but not all, variables used in the derivation of the outcome are missing.
*** No variables used in the derivation of the outcome are missing.

For peer review only - http://bmjopen.bmj.com/site/about/guidelines.xhtml

Barts and The London School of Medicine and Dentistry PCTU

	Questionnaire never returned no. (%)	Questionnaire answered by telephone Not completed* no. (%)	Partially completed** no. (%)	Fully completed*** no. (%)	Questionnaire returned Not completed* no. (%)	Partially completed** no. (%)	Fully completed*** no. (%)
**PSEQ Self efficacy score**							
Baseline	5 (5.6)	n/a	n/a	n/a	0 (0.0)	1 (1.1)	84 (93.3)
60 days	38 (42.2)	10 (11.1)	0 (0.0)	0 (0.0)	2 (2.2)	1 (1.1)	39 (43.3)
3 months	31 (34.4)	13 (14.4)	0 (0.0)	0 (0.0)	1 (1.1)	0 (0.0)	45 (50.0)
6 months	33 (36.7)	24 (26.7)	0 (0.0)	0 (0.0)	0 (0.0)	0 (0.0)	33 (36.7)
**SHOW-Q global score**							
Baseline	5 (5.6)	n/a	n/a	n/a	0 (0.0)	15 (16.7)	70 (77.8)
60 days	38 (42.2)	10 (11.1)	0 (0.0)	0 (0.0)	2 (2.2)	6 (6.7)	34 (37.8)
3 months	31 (34.4)	13 (14.4)	0 (0.0)	0 (0.0)	3 (3.3)	8 (8.9)	35 (38.9)
6 months	33 (36.7)	24 (26.7)	0 (0.0)	0 (0.0)	1 (1.1)	5 (5.6)	27 (30.0)
**SHOW-Q pelvic problem interference score**							
Baseline	5 (5.6)	n/a	n/a	n/a	4 (4.4)	8 (8.9)	73 (81.1)
60 days	38 (42.2)	10 (11.1)	0 (0.0)	0 (0.0)	3 (3.3)	3 (3.3)	36 (40.0)
3 months	31 (34.4)	13 (14.4)	0 (0.0)	0 (0.0)	5 (5.6)	3 (3.3)	38 (42.2)
6 months	33 (36.7)	24 (26.7)	0 (0.0)	0 (0.0)	3 (3.3)	1 (1.1)	29 (32.2)
**MYMOP subjective outcome score**							
Baseline	5 (5.6)	n/a	n/a	n/a	0 (0.0)	1 (1.1)	84 (93.3)
60 days	38 (42.2)	0 (0.0)	1 (1.1)	9 (10.0)	0 (0.0)	10 (11.1)	32 (35.6)
3 months	31 (34.4)	0 (0.0)	1 (1.1)	12 (13.3)	2 (2.2)	9 (10.0)	35 (38.9)
6 months	33 (36.7)	0 (0.0)	2 (2.2)	22 (24.4)	0 (0.0)	4 (4.4)	29 (32.2)

* Questionnaire not answered or all variables used in the derivation of the outcome are missing.
** One or more, but not all, variables used in the derivation of the outcome are missing.
*** No variables used in the derivation of the outcome are missing.

For peer review only - http://bmjopen.bmj.com/site/about/guidelines.xhtml

Barts and The London School of Medicine and Dentistry

PCTU

	Questionnaire never returned no. (%)	Questionnaire answered by telephone: Not completed* no. (%)	Partially completed** no. (%)	Fully completed*** no. (%)	Questionnaire returned: Not completed* no. (%)	Partially completed** no. (%)	Fully completed*** no. (%)
**SF36 - General Health**							
Baseline	5 (5.6)	n/a	n/a	n/a	0 (0.0)	1 (1.1)	84 (93.3)
60 days	38 (42.2)	10 (11.1)	0 (0.0)	0 (0.0)	0 (0.0)	1 (1.1)	41 (45.6)
3 months	31 (34.4)	13 (14.4)	0 (0.0)	0 (0.0)	0 (0.0)	1 (1.1)	45 (50.0)
6 months	33 (36.7)	24 (26.7)	0 (0.0)	0 (0.0)	0 (0.0)	0 (0.0)	33 (36.7)
**SF36 - Physical functioning**							
Baseline	5 (5.6)	n/a	n/a	n/a	0 (0.0)	4 (4.4)	81 (90.0)
60 days	38 (42.2)	10 (11.1)	0 (0.0)	0 (0.0)	0 (0.0)	3 (3.3)	39 (43.3)
3 months	31 (34.4)	13 (14.4)	0 (0.0)	0 (0.0)	1 (1.1)	2 (2.2)	43 (47.8)
6 months	33 (36.7)	24 (26.7)	0 (0.0)	0 (0.0)	0 (0.0)	3 (3.3)	30 (33.3)
**SF36 - Pain**							
Baseline	5 (5.6)	n/a	n/a	n/a	0 (0.0)	0 (0.0)	85 (94.4)
60 days	38 (42.2)	10 (11.1)	0 (0.0)	0 (0.0)	0 (0.0)	0 (0.0)	42 (46.7)
3 months	31 (34.4)	13 (14.4)	0 (0.0)	0 (0.0)	1 (1.1)	0 (0.0)	45 (50.0)
6 months	33 (36.7)	24 (26.7)	0 (0.0)	0 (0.0)	0 (0.0)	0 (0.0)	33 (36.7)
**SF36 - Social functioning**							
Baseline	5 (5.6)	n/a	n/a	n/a	0 (0.0)	0 (0.0)	85 (94.4)
60 days	38 (42.2)	10 (11.1)	0 (0.0)	0 (0.0)	0 (0.0)	1 (1.1)	41 (45.6)
3 months	31 (34.4)	13 (14.4)	0 (0.0)	0 (0.0)	1 (1.1)	1 (1.1)	44 (48.9)
6 months	33 (36.7)	24 (26.7)	0 (0.0)	0 (0.0)	0 (0.0)	1 (1.1)	32 (35.6)

* Questionnaire not answered or all variables used in the derivation of the outcome are missing.
** One or more, but not all, variables used in the derivation of the outcome are missing.
*** No variables used in the derivation of the outcome are missing.

For peer review only - http://bmjopen.bmj.com/site/about/guidelines.xhtml

3.3. Results of analysis of clinical outcomes

Table 12. Descriptive statistics for clinical outcomes

	Intervention (N=31)		Active control (N=30)		Usual care (N=29)	
	no. (%)	mean (sd)	no. (%)	mean (sd)	no. (%)	mean (sd)
**CPAQ pain acceptance score**						
Baseline	29 (93.5)	21.9 (9.5)	27 (90.0)	22.7 (8.4)	28 (96.6)	23.8 (8.5)
60 days	15 (48.4)	21.5 (10.2)	16 (53.3)	22.9 (8.5)	19 (65.5)	24.3 (10.2)
3 months	18 (58.1)	20.8 (7.2)	18 (60.0)	22.9 (8.5)	19 (65.5)	25.0 (8.4)
6 months	21 (67.7)	22.7 (10.1)	16 (53.3)	24.0 (11.2)	19 (65.5)	25.8 (7.6)
Included in analysis*	27 (87.1)		23 (76.7)		25 (86.2)	
**HADS depression score**						
Baseline	30 (96.8)	8.7 (5.1)	27 (90.0)	8.6 (5.0)	27 (93.1)	7.4 (3.6)
60 days	14 (45.2)	7.1 (5.2)	16 (53.3)	8.4 (4.0)	19 (65.5)	8.2 (2.9)
3 months	20 (64.5)	8.7 (3.9)	19 (63.3)	8.2 (5.0)	19 (65.5)	6.8 (3.6)
6 months	21 (67.7)	7.0 (4.9)	16 (53.3)	6.1 (4.4)	20 (69.0)	7.0 (4.6)
Included in analysis*	27 (87.1)		23 (76.7)		26 (89.7)	
**HADS anxiety score**						
Baseline	30 (96.8)	12.6 (5.3)	26 (86.7)	12.0 (5.3)	28 (96.6)	10.9 (3.9)
60 days	15 (48.4)	12.5 (5.6)	16 (53.3)	9.5 (4.1)	19 (65.5)	10.7 (4.1)
3 months	20 (64.5)	12.2 (4.1)	19 (63.3)	9.7 (5.6)	19 (65.5)	10.2 (4.0)
6 months	21 (67.7)	10.1 (4.9)	16 (53.3)	8.4 (5.5)	20 (69.0)	9.1 (4.7)
Included in analysis*	27 (87.1)		23 (76.7)		26 (89.7)	
**CAMS-R mindfulness score**						
Baseline	28 (90.3)	28.6 (6.1)	25 (83.3)	28.8 (7.1)	26 (89.7)	30.3 (5.4)
60 days	15 (48.4)	27.4 (5.6)	16 (53.3)	30.6 (8.4)	19 (65.5)	29.7 (7.6)
3 months	19 (61.3)	29.2 (5.2)	19 (63.3)	30.9 (8.8)	18 (62.1)	31.4 (6.4)
6 months	21 (67.7)	29.0 (7.6)	16 (53.3)	31.0 (7.3)	20 (69.0)	32.0 (8.5)
Included in analysis*	27 (87.1)		23 (76.7)		26 (89.7)	

* Included in analysis if outcome is available for at least one follow-up time point.

For peer review only - http://bmjopen.bmj.com/site/about/guidelines.xhtml

Barts and The London School of Medicine and Dentistry
PCTU

	Intervention (N=31) no. (%)	Intervention (N=31) mean (sd)	Active control (N=30) no. (%)	Active control (N=30) mean (sd)	Usual care (N=29) no. (%)	Usual care (N=29) mean (sd)
**CPG disability score**						
Baseline	30 (96.8)	60.6 (24.4)	27 (90.0)	64.6 (19.6)	28 (96.6)	59.2 (24.4)
60 days	15 (48.4)	56.7 (19.8)	16 (53.3)	54.8 (25.0)	19 (65.5)	54.7 (22.9)
3 months	19 (61.3)	61.1 (17.3)	19 (63.3)	52.5 (27.5)	19 (65.5)	52.8 (23.5)
6 months	21 (67.7)	48.3 (28.1)	16 (53.3)	48.5 (24.4)	19 (65.5)	54.2 (23.7)
Included in analysis*	27 (87.1)		23 (76.7)		25 (86.2)	
**PSEQ Self efficacy score**						
Baseline	30 (96.8)	29.1 (14.7)	27 (90.0)	27.9 (14.6)	27 (93.1)	35.5 (10.6)
60 days	14 (45.2)	32.4 (13.9)	9 (30.0)	30.9 (15.9)	16 (55.2)	34.5 (13.1)
3 months	17 (54.8)	28.9 (11.8)	12 (40.0)	30.2 (14.2)	16 (55.2)	39.3 (9.7)
6 months	11 (35.5)	34.3 (12.5)	10 (33.3)	33.7 (17.7)	12 (41.4)	40.2 (13.1)
Included in analysis*	21 (67.7)		18 (60.0)		21 (72.4)	
**SHOW-Q global score**						
Baseline	17 (54.8)	45.4 (20.3)	20 (66.7)	50.9 (20.9)	19 (65.5)	58.1 (22.2)
60 days	4 (12.9)	69.3 (13.3)	8 (26.7)	54.1 (18.0)	13 (44.8)	53.7 (24.5)
3 months	5 (16.1)	51.1 (26.6)	11 (36.7)	44.9 (19.4)	10 (34.5)	61.2 (24.8)
6 months	7 (22.6)	52.3 (15.6)	4 (13.3)	60.9 (14.3)	7 (24.1)	58.5 (26.4)
Included in analysis*	9 (29.0)		14 (46.7)		16 (55.2)	
**SHOW-Q pelvic problem interference score**						
Baseline	23 (74.2)	47.1 (29.0)	24 (80.0)	49.0 (32.7)	26 (89.7)	56.4 (25.9)
60 days	12 (38.7)	60.4 (33.7)	9 (30.0)	60.2 (27.9)	15 (51.7)	51.7 (28.9)
3 months	12 (38.7)	54.9 (34.0)	11 (36.7)	50.0 (25.3)	15 (51.7)	69.4 (32.8)
6 months	9 (29.0)	65.7 (22.2)	9 (30.0)	59.3 (33.4)	11 (37.9)	57.6 (32.8)
Included in analysis*	16 (51.6)		17 (56.7)		20 (69.0)	
**MYMOP subjective outcome score**						
Baseline	30 (96.8)	4.1 (1.2)	27 (90.0)	3.9 (1.3)	27 (93.1)	3.9 (1.1)
60 days	13 (41.9)	3.2 (1.4)	14 (46.7)	3.5 (1.3)	14 (48.3)	3.6 (1.2)
3 months	15 (48.4)	3.4 (1.3)	16 (53.3)	3.1 (1.6)	16 (55.2)	2.9 (1.4)
6 months	18 (58.1)	3.0 (1.4)	15 (50.0)	3.0 (1.5)	18 (62.1)	3.1 (1.5)
Included in analysis*	25 (80.6)		21 (70.0)		24 (82.8)	

* Included in analysis if outcome is available for at least one follow-up time point.

PCTU only

For peer review only - http://bmjopen.bmj.com/site/about/guidelines.xhtml

Barts and The London School of Medicine and Dentistry

PCTU

	Intervention (N=31)		Active control (N=30)		Usual care (N=29)	
	no. (%)	mean (sd)	no. (%)	mean (sd)	no. (%)	mean (sd)
**SF36 - General Health**						
Baseline	29 (93.5)	39.1 (20.3)	27 (90.0)	42.0 (19.8)	28 (96.6)	37.9 (21.4)
60 days	15 (48.4)	45.0 (21.2)	9 (30.0)	51.1 (19.2)	17 (58.6)	37.6 (19.9)
3 months	17 (54.8)	44.1 (21.7)	12 (40.0)	42.1 (23.2)	16 (55.2)	40.3 (19.4)
6 months	11 (35.5)	54.5 (19.0)	10 (33.3)	54.5 (24.2)	12 (41.4)	40.0 (27.8)
Included in analysis*	21 (67.7)		18 (60.0)		22 (75.9)	
**SF36 - Physical functioning**						
Baseline	28 (90.3)	56.3 (30.2)	26 (86.7)	55.8 (32.2)	27 (93.1)	66.5 (30.4)
60 days	13 (41.9)	61.2 (27.1)	9 (30.0)	60.6 (25.7)	17 (58.6)	66.5 (30.0)
3 months	15 (48.4)	58.3 (24.0)	12 (40.0)	54.6 (30.7)	16 (55.2)	69.1 (27.5)
6 months	10 (32.3)	66.0 (26.5)	10 (33.3)	72.0 (28.6)	10 (34.5)	63.5 (37.4)
Included in analysis*	20 (64.5)		18 (60.0)		22 (75.9)	
**SF36 - Pain**						
Baseline	30 (96.8)	35.1 (17.5)	27 (90.0)	34.7 (20.6)	28 (96.6)	37.6 (20.6)
60 days	15 (48.4)	39.0 (19.2)	9 (30.0)	43.1 (33.0)	18 (62.1)	40.0 (24.5)
3 months	17 (54.8)	43.7 (17.6)	12 (40.0)	46.7 (22.7)	16 (55.2)	49.5 (25.9)
6 months	11 (35.5)	50.0 (17.8)	10 (33.3)	61.0 (19.9)	12 (41.4)	48.3 (24.8)
Included in analysis*	21 (67.7)		18 (60.0)		22 (75.9)	
**SF36 - Social functioning**						
Baseline	30 (96.8)	37.5 (19.1)	27 (90.0)	38.0 (28.3)	28 (96.6)	50.4 (25.3)
60 days	15 (48.4)	45.8 (27.4)	9 (30.0)	55.6 (29.4)	17 (58.6)	51.5 (28.9)
3 months	17 (54.8)	50.7 (20.9)	12 (40.0)	49.0 (30.4)	15 (51.7)	57.5 (29.0)
6 months	11 (35.5)	54.5 (21.8)	10 (33.3)	56.3 (27.8)	11 (37.9)	59.1 (34.0)
Included in analysis*	21 (67.7)		18 (60.0)		22 (75.9)	

* Included in analysis if outcome is available for at least one follow-up time point.

For peer review only - http://bmjopen.bmj.com/site/about/guidelines.xhtml

Barts and The London School of Medicine and Dentistry PCTU

Table 13. Estimated treatment effects for clinical outcomes

	Intervention vs Active control adjusted mean difference (95% CI)	Intervention vs Usual care adjusted mean difference (95% CI)	Active control vs Usual care adjusted mean difference (95% CI)
**CPAQ pain acceptance score**			
60 days	-2.3 (-6.6, 2.0)	-4.0 (-8.1, 0.1)	-1.7 (-5.8, 2.4)
3 months	-3.0 (-6.8, 0.7)	-4.5 (-8.2, -0.9)	-1.5 (-5.2, 2.2)
6 months	-1.4 (-5.8, 3.0)	-4.0 (-8.2, 0.2)	-2.5 (-7.0, 2.0)
**HADS depression score**			
60 days	-0.7 (-2.7, 1.2)	-1.2 (-3.1, 0.6)	-0.5 (-2.3, 1.3)
3 months	0.5 (-1.6, 2.6)	1.2 (-0.9, 3.4)	0.8 (-1.4, 2.9)
6 months	0.5 (-1.7, 2.6)	0.4 (-1.7, 2.4)	-0.1 (-2.3, 2.1)
**HADS anxiety score**			
60 days	2.0 (-0.1, 4.1)	1.0 (-1.1, 3.0)	-1.0 (-3.0, 1.0)
3 months	1.9 (-0.3, 4.0)	1.5 (-0.6, 3.6)	-0.4 (-2.5, 1.7)
6 months	0.1 (-2.3, 2.5)	0.3 (-2.0, 2.6)	0.2 (-2.2, 2.6)
**CAMS-R mindfulness score**			
60 days	-3.5 (-7.3, 0.4)	-2.2 (-5.9, 1.4)	1.2 (-2.5, 4.9)
3 months	-2.5 (-5.8, 0.8)	-2.3 (-5.5, 1.0)	0.2 (-3.1, 3.5)
6 months	-1.4 (-4.9, 2.2)	-2.9 (-6.3, 0.4)	-1.6 (-5.1, 2.0)
**CPG disability score**			
60 days	5.1 (-7.2, 17.5)	3.8 (-8.1, 15.7)	-1.4 (-13.1, 10.4)
3 months	8.8 (-3.4, 21.0)	7.6 (-4.5, 19.7)	-1.2 (-13.4, 10.9)
6 months	1.9 (-12.1, 16.0)	1.0 (-12.6, 14.5)	-1.0 (-15.3, 13.4)
**PSEQ Self efficacy score**			
60 days	0.1 (-8.2, 8.4)	-0.2 (-7.4, 6.9)	-0.3 (-8.4, 7.8)
3 months	-3.6 (-9.8, 2.6)	-7.1 (-12.9, -1.2)	-3.5 (-9.8, 2.9)
6 months	-5.9 (-14.8, 3.0)	-8.7 (-17.1, -0.2)	-2.8 (-11.6, 5.9)

For peer review only - http://bmjopen.bmj.com/site/about/guidelines.xhtml

	Intervention vs Active control adjusted mean difference (95% CI)	Intervention vs Usual care adjusted mean difference (95% CI)	Active control vs Usual care adjusted mean difference (95% CI)
**SHOW-Q global score**			
60 days	7.0 (-7.2, 21.2)	8.3 (-5.2, 21.8)	1.3 (-9.8, 12.4)
3 months	3.5 (-13.9, 20.9)	-4.8 (-22.0, 12.3)	-8.3 (-23.2, 6.6)
6 months	-11.5 (-27.7, 4.8)	-10.7 (-25.8, 4.3)	0.7 (-14.5, 15.9)
**SHOW-Q pelvic problem interference score**			
60 days	-7.2 (-28.0, 13.5)	3.6 (-14.7, 21.9)	10.9 (-8.9, 30.7)
3 months	-1.2 (-25.1, 22.8)	-10.2 (-32.5, 12.1)	-9.0 (-31.9, 13.8)
6 months	3.3 (-21.3, 27.9)	4.7 (-18.7, 28.1)	1.4 (-22.1, 24.8)
**MYMOP subjective outcome score**			
60 days	0.0 (-0.7, 0.8)	-0.3 (-1.1, 0.4)	-0.4 (-1.1, 0.4)
3 months	0.6 (-0.2, 1.5)	0.6 (-0.2, 1.4)	-0.0 (-0.9, 0.8)
6 months	-0.2 (-1.1, 0.7)	0.2 (-0.7, 1.1)	0.4 (-0.6, 1.3)
**SF36 - General Health**			
60 days	-8.8 (-19.4, 1.8)	-0.9 (-10.0, 8.3)	7.9 (-2.5, 18.3)
3 months	2.0 (-7.3, 11.3)	-5.6 (-14.5, 3.3)	-7.6 (-17.1, 1.9)
6 months	-4.6 (-18.2, 8.9)	-1.9 (-14.9, 11.0)	2.7 (-10.8, 16.2)
**SF36 - Physical functioning**			
60 days	0.1 (-16.0, 16.2)	-6.5 (-20.9, 7.9)	-6.6 (-22.2, 9.0)
3 months	-4.9 (-19.0, 9.3)	-7.7 (-20.8, 5.4)	-2.8 (-16.8, 11.1)
6 months	-2.4 (-24.7, 19.9)	6.3 (-15.7, 28.2)	8.6 (-13.6, 30.9)
**SF36 - Pain**			
60 days	-3.7 (-19.8, 12.3)	0.5 (-12.9, 13.9)	4.2 (-11.4, 19.8)
3 months	-6.4 (-20.7, 7.9)	-7.3 (-20.8, 6.2)	-0.9 (-15.3, 13.6)
6 months	-8.5 (-22.8, 5.8)	0.7 (-13.0, 14.4)	9.2 (-5.0, 23.4)
**SF36 - Social functioning**			
60 days	-17.1 (-33.4, -0.7)	5.2 (-8.8, 19.1)	22.2 (5.7, 38.8)
3 months	-8.2 (-26.5, 10.1)	4.3 (-13.2, 21.8)	12.5 (-6.5, 31.5)
6 months	0.3 (-18.9, 19.6)	3.9 (-15.0, 22.8)	3.5 (-16.0, 23.1)

For peer review only - http://bmjopen.bmj.com/site/about/guidelines.xhtml

Barts and The London
School of Medicine and Dentistry

PCTU

Figure 2. Estimated treatment effects and 95% confidence intervals for CPAQ

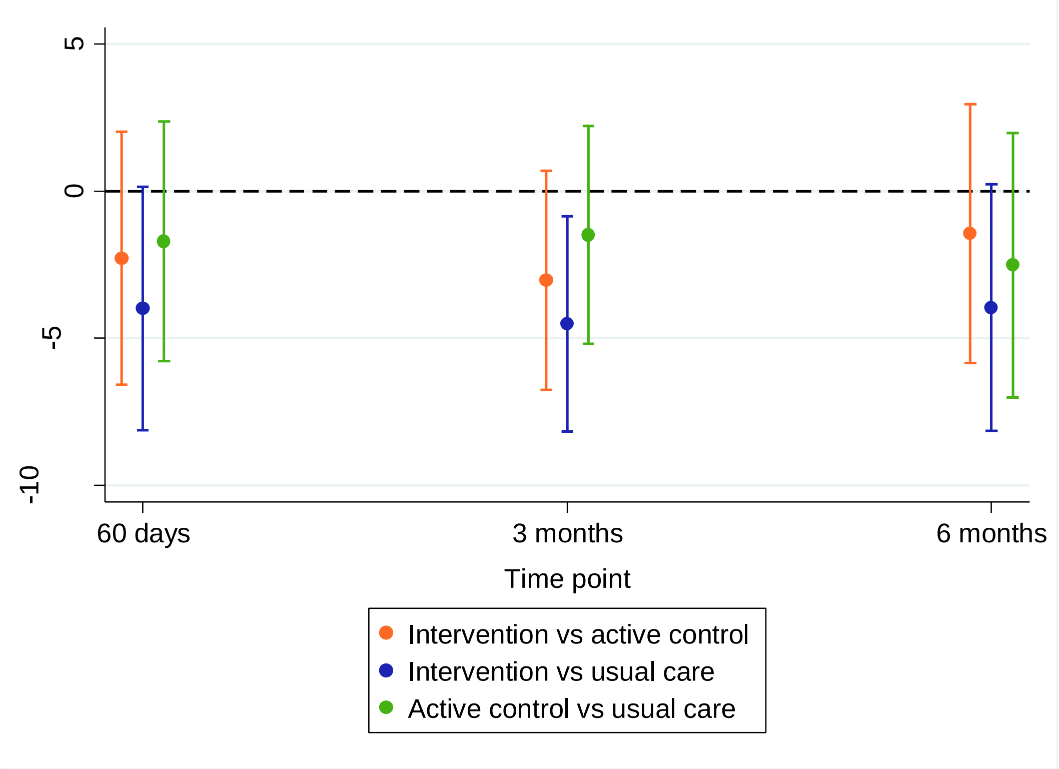

For peer review only - http://bmjopen.bmj.com/site/about/guidelines.xhtml

CONSORT 2010 checklist of information to include when reporting a pilot or feasibility trial*

Section/Topic	Item No	Checklist item	Reported on page No
**Title and abstract**			
	1a	Identification as a pilot or feasibility randomised trial in the title	1
	1b	Structured summary of pilot trial design, methods, results, and conclusions (for specific guidance see CONSORT abstract extension for pilot trials)	2
**Introduction**			
Background and objectives	2a	Scientific background and explanation of rationale for future definitive trial, and reasons for randomised pilot trial	4
	2b	Specific objectives or research questions for pilot trial	4
**Methods**			
Trial design	3a	Description of pilot trial design (such as parallel, factorial) including allocation ratio	5
	3b	Important changes to methods after pilot trial commencement (such as eligibility criteria), with reasons	n/a
Participants	4a	Eligibility criteria for participants	5
	4b	Settings and locations where the data were collected	6
	4c	How participants were identified and consented	5
Interventions	5	The interventions for each group with sufficient details to allow replication, including how and when they were actually administered	5
Outcomes	6a	Completely defined prespecified assessments or measurements to address each pilot trial objective specified in 2b, including how and when they were assessed	6-7
	6b	Any changes to pilot trial assessments or measurements after the pilot trial commenced, with reasons	7
	6c	If applicable, prespecified criteria used to judge whether, or how, to proceed with future definitive trial	n/a
Sample size	7a	Rationale for numbers in the pilot trial	8
	7b	When applicable, explanation of any interim analyses and stopping guidelines	n/a
Randomisation:			
Sequence generation	8a	Method used to generate the random allocation sequence	5
	8b	Type of randomisation(s); details of any restriction (such as blocking and block size)	5
Allocation concealment mechanism	9	Mechanism used to implement the random allocation sequence (such as sequentially numbered containers), describing any steps taken to conceal the sequence until interventions were assigned	5

For peer review only

Implementation	10	Who generated the random allocation sequence, who enrolled participants, and who assigned participants to interventions	5
Blinding	11a	If done, who was blinded after assignment to interventions (for example, participants, care providers, those assessing outcomes) and how	6
	11b	If relevant, description of the similarity of interventions	5
Statistical methods	12	Methods used to address each pilot trial objective whether qualitative or quantitative	8
**Results**			
Participant flow (a diagram is strongly recommended)	13a	For each group, the numbers of participants who were approached and/or assessed for eligibility, randomly assigned, received intended treatment, and were assessed for each objective	13
	13b	For each group, losses and exclusions after randomisation, together with reasons	13
Recruitment	14a	Dates defining the periods of recruitment and follow-up	8
	14b	Why the pilot trial ended or was stopped	8
Baseline data	15	A table showing baseline demographic and clinical characteristics for each group	15
Numbers analysed	16	For each objective, number of participants (denominator) included in each analysis. If relevant, these numbers should be by randomised group	Supplementary tables
Outcomes and estimation	17	For each objective, results including expressions of uncertainty (such as 95% confidence interval) for any estimates. If relevant, these results should be by randomised group	Supplementary tables
Ancillary analyses	18	Results of any other analyses performed that could be used to inform the future definitive trial	n/a
Harms	19	All important harms or unintended effects in each group (for specific guidance see CONSORT for harms)	Supplementary tables
	19a	If relevant, other important unintended consequences	n/a
**Discussion**			
Limitations	20	Pilot trial limitations, addressing sources of potential bias and remaining uncertainty about feasibility	9
Generalisability	21	Generalisability (applicability) of pilot trial methods and findings to future definitive trial and other studies	9
Interpretation	22	Interpretation consistent with pilot trial objectives and findings, balancing potential benefits and harms, and considering other relevant evidence	9
	22a	Implications for progression from pilot to future definitive trial, including any proposed amendments	9
**Other information**			
Registration	23	Registration number for pilot trial and name of trial registry	3
Protocol	24	Where the pilot trial protocol can be accessed, if available	Supplementary material
Funding	25	Sources of funding and other support (such as supply of drugs), role of funders	3

For peer review only

	26	Ethical approval or approval by research review committee, confirmed with reference number	8

Citation: Eldridge SM, Chan CL, Campbell MJ, Bond CM, Hopewell S, Thabane L, et al. CONSORT 2010 statement: extension to randomised pilot and feasibility trials. BMJ. 2016;355.
*We strongly recommend reading this statement in conjunction with the CONSORT 2010, extension to randomised pilot and feasibility trials, Explanation and Elaboration for important clarifications on all the items. If relevant, we also recommend reading CONSORT extensions for cluster randomised trials, non-inferiority and equivalence trials, non-pharmacological treatments, herbal interventions, and pragmatic trials. Additional extensions are forthcoming: for those and for up to date references relevant to this checklist, see www.consort-statement.org.

For peer review only
